# α-Amido Sulfonium Salts Provide a Platform for Photocatalytic Metal-Free Carbon–Carbon Bond Formation in Amides

**DOI:** 10.1021/acscatal.5c02029

**Published:** 2025-05-05

**Authors:** Leendert van Dalsen, Shibo Zhang, Wei Tian, Ben W. Joynson, Ciro Romano, David J. Procter

**Affiliations:** Department of Chemistry, University of Manchester, Oxford Road, Manchester M13 9PL, U.K.

**Keywords:** photocatalysis, sulfonium salts, amides, alkylation, alkenylation, heteroarylation

## Abstract

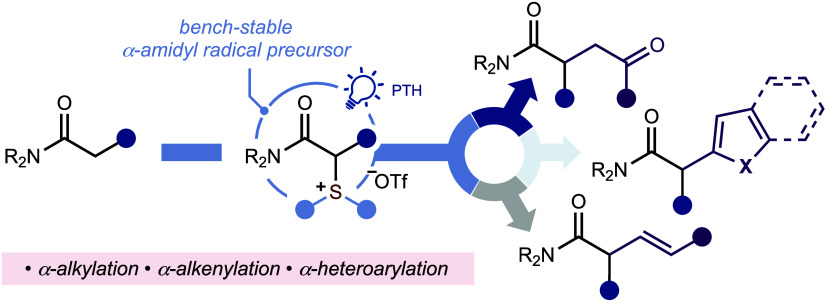

α-Amido sulfonium salts are bench-stable precursors of α-amidyl radicals, prepared directly from tertiary amides. These largely unexplored sulfonium salts engage in metal-free photocatalytic C(sp^3^)–C(sp^2^) and C(sp^3^)–C(sp^3^) bond formation in cross-coupling reactions that realize the formal α-alkylation, α-alkenylation, and α-arylation of amides. Fine-tuning the photocatalytic conditions allows divergent access to important 1,4-dicarbonyl compounds, skipped unsaturated amides, and α-heteroarylated amides, thus showcasing the role of α-amido sulfonium salts in establishing a novel platform for amide functionalization. Preliminary mechanistic experiments support a photocatalytic cycle in which the sulfonium salts are reduced to form α-amidyl radicals that couple with various electron-rich partners in a chemodivergent approach for the introduction of complexity in amides.

## Introduction

**Scheme 1:**
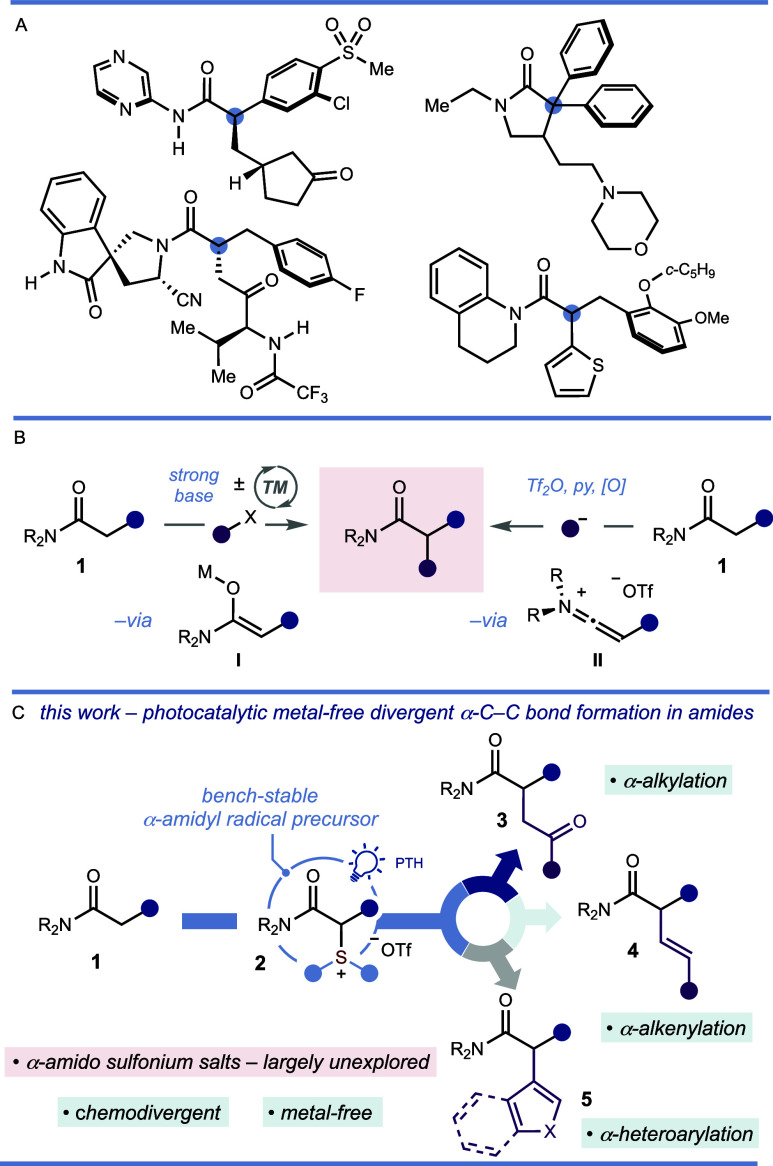
(A) Bioactive Amides with α-branching; (B) Established Methods for the Synthesis of α-substituted Amides Involving Either Enolate or Ketiminium Ion Intermediates; (C) Chemodivergent Photocatalytic Strategy for the Metal-Free Construction of C–C Bonds at the α-position of Amides TM = transition metal; Tf = 1,1,1-trifluoromethylsulfonyl; Py = pyridine; PTH = 10-phenylphenothiazine.

As a privileged compound class, amides play essential roles in our lives and in Life itself.^1^ For example, amide motifs are a critical repeat unit in protein scaffolds and synthetic materials and a ubiquitous component of medicines. As such, precision construction and elaboration of amide motifs remain a vital goal for synthetic method development.^2−4^ The high frequency with which branching α- to the amide carbonyl group is found – for example, in biologically relevant amides (Scheme 1A)^5^ – necessitates the development of new methods for the construction of C–C bonds α- to amide carbonyl groups.

Classically, the introduction of branching in amides through addition of α-carbon substituents relies on deprotonation of simple amides **1** and engagement of the resultant metal enolates **I** with carbon electrophiles (Scheme 1B, left),^6,7^ or on substitution of a preinstalled α-functional group.^8^ In the former, stoichiometric deprotonation by strong base (amide p*K*_a_ ∼35 in DMSO)^9^ and quenching with reactive electrophiles raises issues of chemoselectivity and functional group tolerance. While the use of transition metal catalysts greatly facilitates carbon–carbon bond formation in amides,^10^ strong base is still required for enolate formation, and the use of transition metals can raise issues around cost and product contamination.^11^

Recent advances in amide α-functionalization^12^ often involve the *in situ* generation of ketiminium ions **II** from tertiary amides **1** and trapping with an oxidant followed by a nucleophile (Scheme 1B, right). For example, work by Maulide and others has led to methods for α-arylation^13^ and α-allylation/allenylation^14^ in which the oxidant and nucleophile are combined and the trapping is intramolecular. Of note, Maulide has described the harnessing of ketiminiums **II** derived from tertiary amides in intermolecular couplings with an oxidant and a preformed metal enolate nucleophile.^15^ Formation of α-C–X bonds in amides **1** is also possible;^16^ for example, Maulide has reported the α-oxytriflation of tertiary amides **1** and the direct use of the triflates in asymmetric Ni-catalyzed cross-coupling with aryl boranes.^17^

Turning to radical approaches involving the formation and engagement of α-amidyl radicals, oxidative methods also typically require the formation of metal enolates **I**,^18^ while reductive methods require access to relatively hard to reduce α-haloamides^8,19^ or Katritzky salts^20^ derived from α-amino acid derivatives rather than simple amides **1**.^21^

Given the interest in the development of formal photocatalytic C(sp^2^)–H functionalization strategies, driven by the formation of sulfonium salt intermediates,^22^ using sulfoxide activators,^23^ we reasoned that a similar approach could be used for the formal α-C(sp^3^)–H manipulation of amides **1**. We posited that α-amido sulfonium salts **2** would be bench-stable, typically crystalline, easy to handle, radical precursors, capable of liberating α-amidyl radicals on demand using an organic photocatalyst and light (Scheme 1C). A divergent platform would be established, and several sought-after cross-coupling reactions achieved, if multiple coupling partners could be employed to intercept the α-amidyl radical intermediates. Here, we disclose the direct synthesis of a library of α-amido sulfonium salts **2**, a study of their physical and photophysical properties, and their first exploitation as α-amidyl radical precursors. The resulting photocatalytic, metal-free processes constitute a divergent platform for the conversion of simple amides into high-value, branched amides **3**–**5** by the construction of various α-C–C bonds.

## Methods

In a single, isolated report, Movassaghi described an α-sulfenylation of amides that proceeds first via ketiminium ions **II**, then α-amido sulfonium salts, followed by *in situ* demethylation.^24^ In three examples, intermediate α-amido sulfonium salts were isolated and characterized. Further study of the properties of the sulfonium salts was not reported.

**Scheme 2:**
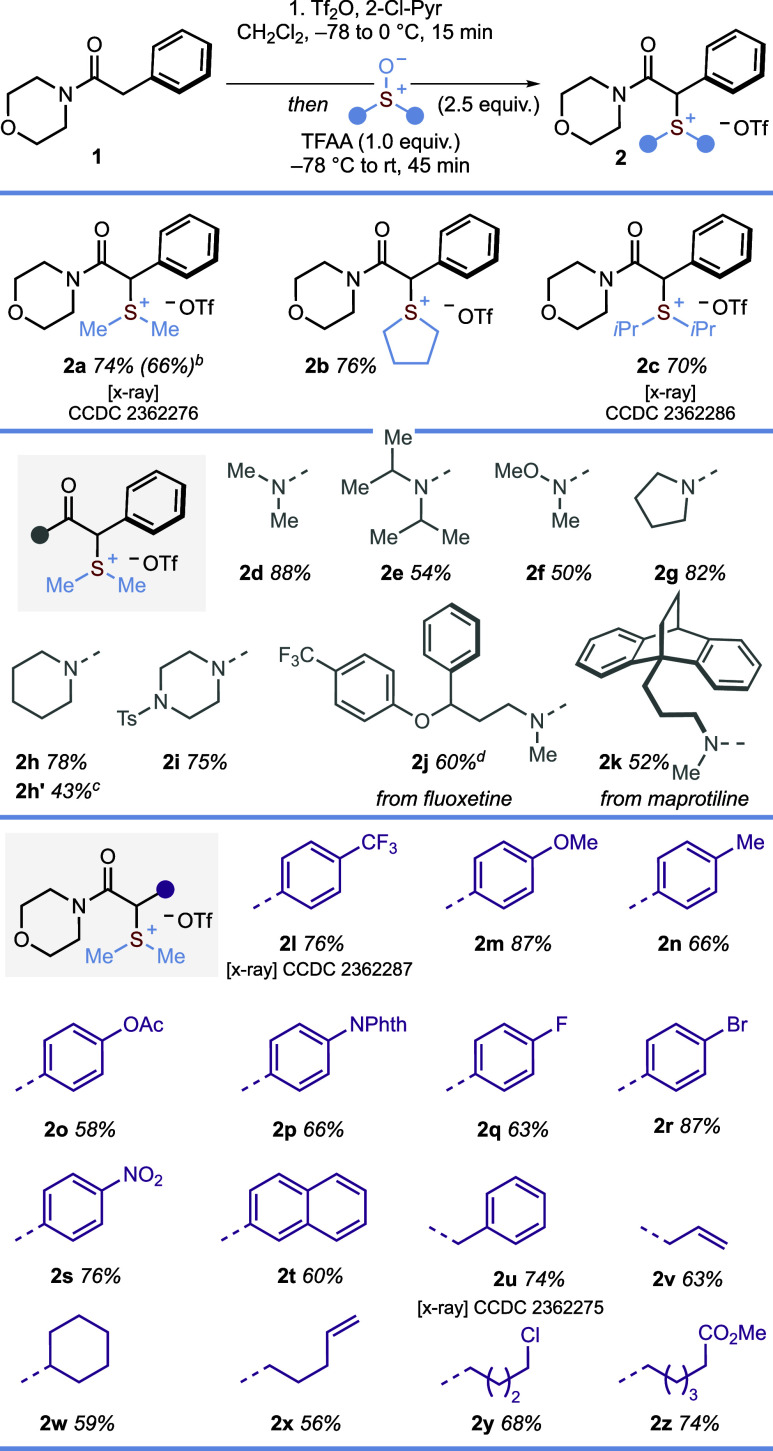
α-Amido Sulfonium Salt Synthesis Reaction conditions: **1** (1.0 mmol), 2-chloropyridine (2.0 mmol), Tf_2_O (1.1 mmol) in CH_2_Cl_2_ (3.5 mL) at −78 °C → 0 °C for 15 min followed by sulfoxide (2.5 mmol) in CH_2_Cl_2_ (0.5 mL) and TFAA (1.0 mmol) at −78 °C → 0 °C then 0 °C → rt for 45 min. On a 10 mmol scale. **2h**′ bears an α-(4-CF_3_)-phenyl group instead of the α-phenyl substituent. On a 2.0 mmol scale.

Adapting the conditions of Movassaghi and co-workers,^24^ a collection of α-amido sulfonium salts was prepared, with the products isolated either by trituration from hexane followed by recrystallization from ethyl acetate or by column chromatography (Scheme 2). In contrast to amide functionalization protocols based on deprotonation using strong bases, selective installation of the sulfonium salt moiety is achieved under mild conditions by using a pyridine base. Varying the sulfoxide activator delivered sulfonium salts **2a**–**c** bearing different *S*-substituents in good yield. With the atom economy of future processes in mind, for the rest of our studies, we opted to use DMSO as an inexpensive activator of amides to deliver dimethyl sulfonium motifs with the lowest molecular weight. Concerning the amino fragment in amides, aliphatic, acyclic tertiary amides were tolerated (**2d** and **2e**), including those derived from the drug molecules fluoxetine and maprotiline (**2j** and **2k**). Cyclic amino motifs of different ring sizes, including morpholinyl (**2a**), pyrrolidinyl (**2g**), piperidinyl (**2h** and **2h**′), and piperazinyl (**2i**) – were well tolerated. Finally, compatibility with the *N*-methoxy *N*-methyl motif allowed access to the Weinreb amide sulfonium salt (**2f**) in moderate yield. Varying the α-substituent, we found that aryl substituents bearing both electron-donating (**2m**–**p**) and electron-withdrawing groups (**2l**, **2q**–**s**) are well accommodated, as was a naphthyl substituent (**2t**). Despite the potential for elimination to form α,β-unsaturated amides, α-amido sulfonium salts bearing benzyl (**2u**), allyl (**2v**), cyclohexyl (**2w**), and functionalized alkyl groups featuring alkenyl (**2x**), chloro (**2y**), and carbomethoxy (**2z**) groups were prepared in moderate yield and were found to be stable. The structures of **2a**, **2c**, **2l**, and **2u** were confirmed by X-ray crystallography.^25^ In line with known methods for amide activation involving ketiminium ion formation, the method is currently suited to the activation of tertiary amides.^12−17^

**Scheme 3:**
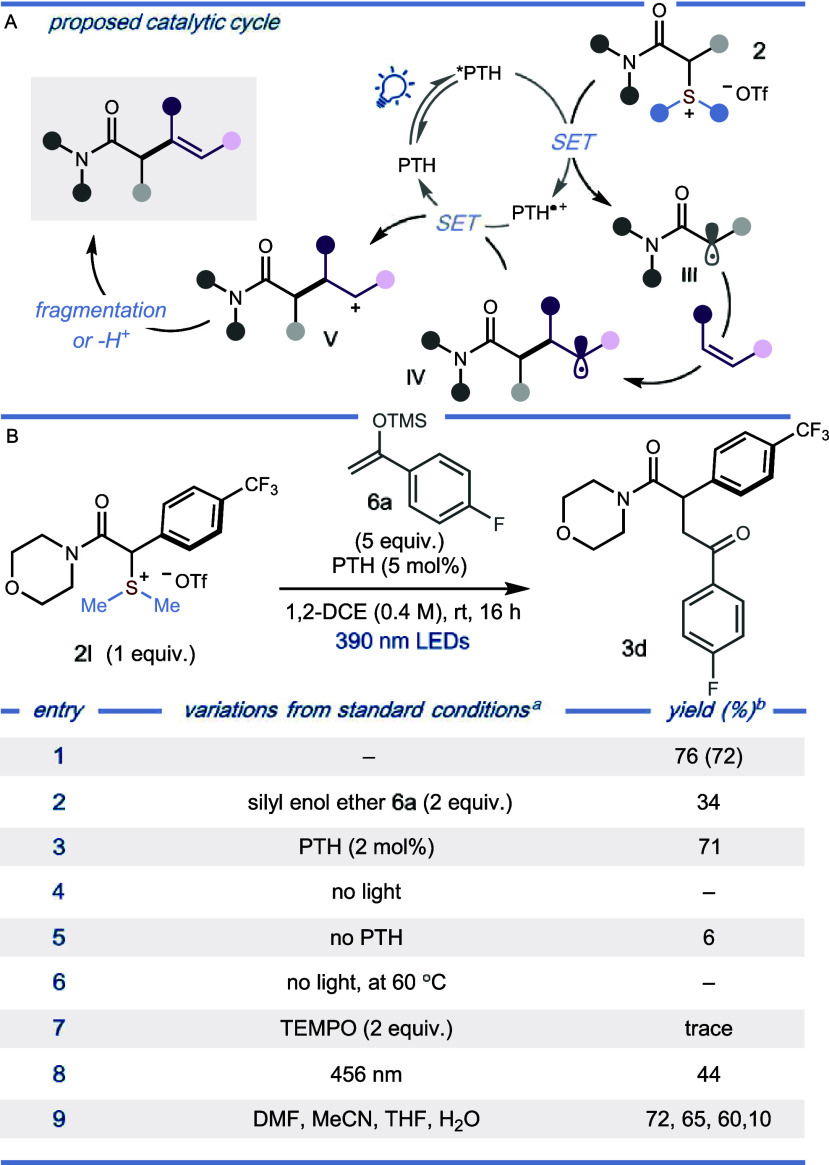
(A) Proposed General Photocatalytic Cycle; (B) Optimization of Reaction Conditions for α-alkylation of α-amido Sulfonium Salt **2l** with Silyl Enol Ether **6a** Reaction conditions: **2l** (0.10 mmol), **6a** (0.50 mmol), PTH (0.005 mmol) in 1,2-DCE (0.25 mL) irradiated at 390 nm (Purple Kessil Lamp) overnight. Yields were determined by ^1^H qNMR with MeNO_2_ as the internal standard. Yield of isolated product in parentheses.

In developing a suite of metal-free photocatalytic protocols that leverage α-amido sulfonium salts, we envisaged a mechanism whereby salts **2** would undergo SET reduction in the presence of an appropriate photocatalyst, triggering fragmentation to give α-amidyl radicals **III**. These electrophilic radical species would then be intercepted by electron-rich traps to give radical adducts **IV**. Finally, a single electron oxidation step would deliver the desired product – via fragmentation or deprotonation of carbocation intermediates **V** – and regenerate the photocatalyst (Scheme 3A). CV measurements gave irreversible reduction curves, with similar reduction potentials measured for α-amido sulfonium salts **2a** and **2b**, bearing different *S*-motifs (*E*_1/2red_ = −1.49 and −1.48 V vs SCE, respectively), but a more negative reduction potential in the case of the α-alkyl-substituted derivative **2u** (*E*_1/2red_ = −1.70 V vs SCE). PTH (10-phenylphenothiazine) (PTH^•+^/*PTH = −2.10 V vs SCE) was therefore selected as a photocatalyst able to reduce all α-amido sulfonium salts. Of note, the reduction potential of **9a** – the α-bromo amide analogue of **2u** (see Scheme 6C) – has a much lower reduction potential (<−2.5 eV vs SCE), outside the reduction range of PTH. Thus, α-bromo amides are ineffective substrates in the following couplings.

We began by optimizing the coupling of α-amido sulfonium salt **2l** with silyl enol ether **6a** to give the product of amide α-alkylation, 1,4-dicarbonyl **3d** (Scheme 3B). The use of 5 equiv of the silyl enol ether radical trap worked well; decreasing the stoichiometry to 2 equiv negatively affected the yield of **3d** (Entry 2). Although the PTH loading could be reduced to 2 mol % without significantly altering the yield, we opted to use 5 mol % of this inexpensive photocatalyst so that a single set of reaction conditions could be applied across the scope (Entry 3).

Control experiments confirmed the need for photocatalyst and light (Entries 4 and 5); a moderate yield of **3d** was obtained by irradiation with 456 nm light, while addition of the radical scavenger TEMPO shut down product formation, supporting the proposed radical mechanism (Entries 7 and 8). The cross-coupling was found to proceed well in several aprotic solvents, with the best results obtained in 1,2-DCE (Entry 9).

**Scheme 4:**
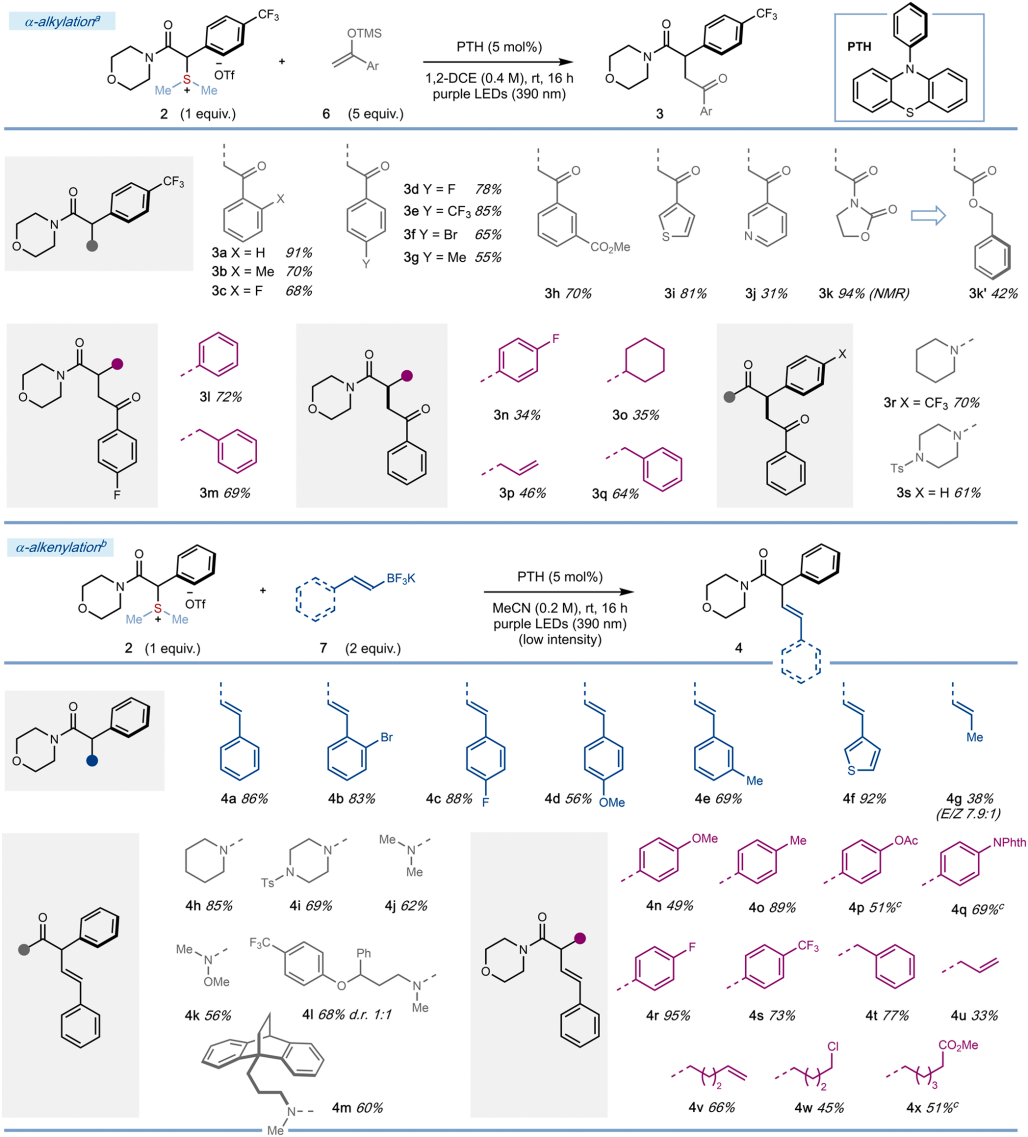
Reaction Scope of the α-alkylation (top) and α-alkenylation (bottom) of α-amido Sulfonium Salts with Silyl Enol Ethers **6** and Vinyl Trifluoroborate Salts **7** as Coupling Partners, Respectively Reaction conditions: **2** (0.10 mmol), **6** (0.50 mmol), PTH (0.005 mmol) in 1,2-DCE (0.25 mL) irradiated at 390 nm (Purple Kessil lamp) overnight. Reaction conditions: **2** (0.10 mmol), **7** (0.20 mmol), PTH (0.005 mmol) in MeCN (0.5 mL) irradiated at 390 nm (Tuna Blue Kessil lamp – providing low-intensity 390 nm light) overnight. Irradiated at 390 nm (Purple Kessil lamp), 25% intensity.

We next investigated the generality of the photocatalytic α-alkylation of α-amido sulfonium salts to give important 1,4-dicarbonyl products **3** (Scheme 4). Silyl enol ethers **6** bearing electron-withdrawing groups on the aromatic ring were well tolerated – halogens (**3c**, **3d**, **3f**), trifluoromethyl (**3e**), ester (**3h**). Substitution in the *ortho, meta, and para*-positions of the silyl enol ethers did not impede the reaction. Sigma electron-donating methyl groups were also compatible with cross-coupling (**3b** and **3g**). In addition, silyl enol ethers derived from heteroaryl ketones were also viable substrates (**3i** and **3j**). Engaging a radical trap derived from 3-acetyl-2-oxazolidinone gave **3k** in 94% NMR yield; attempts to purify the product led to decomposition, but we could isolate the corresponding ester **3k**′ after LiOBn addition. The exchange of an α-phenyl substituent (**3l**, 72%) for an α-benzyl substituent (**3m**, 69% and **3q**, 64%) did not impact negatively the reaction outcome. Salts containing simple aliphatic (**3o**) and allyl (**3p**) α-substituents were also compatible with the photocatalytic coupling, although yields were moderate. Variation of the amide motif in the salts delivered piperidinyl and piperazinyl products **3r** and **3s**, respectively.

α-Amido sulfonium salts also underwent efficient coupling with readily accessible potassium alkenyl trifluoroborate salts **7**, delivering skipped unsaturated amide products of metal-free α-alkenylation **4**. The use of acetonitrile as a solvent and more dilute conditions were the only changes made to the easily tunable protocol. We observed that a higher *E/Z* ratio of the product alkenes (8:1 to >20:1) was obtained when irradiation was switched to low-intensity 390 nm light; *E*-to-*Z* photoisomerization of the starting alkenyl trifluoroborate salt **7** takes place under irradiation with high intensity 390 nm light. The stoichiometry of the coupling partner can be reduced (2 equiv) without compromising yields, and control experiments confirmed the necessity for photocatalyst and light.

Varying the trifluoroborate salt coupling partner, aryl substituents bearing electron-withdrawing (**4b** and **4c**) and electron-donating (**4d** and **4e**) groups were well accommodated in the *ortho*-, *meta*-, and *para*- positions. Use of thiophene-containing trifluoroborate salts gave **4f** in excellent 92% yield. Use of 1-propenyl trifluoroborate salt furnished the desired product **4g** in modest yield, presumably due to the lower stabilization of the radical generated upon trapping. The amino fragment in the sulfonium salts could be varied, with cyclic morpholinyl (**4a**), piperidinyl (**4h**), piperazinyl (**4i**), and acyclic motifs (**4j** and **4k**) – including those derived from fluoxetine and maprotiline (**4l** and **4m**) – accommodated.

The Weinreb amide product of metal-free alkenylation **4k** stands out as a potentially useful building block for synthesis. Electron-donating (**4n**-**4q**) and electron-withdrawing (**4r** and **4s**) groups were tolerated on the α-aryl substituents of the sulfonium salts. In addition, α-benzyl (**4t**) and allyl (**4u**) substituents proved compatible as did α-alkyl units bearing alkenyl (**4v**), chloro (**4w**), and carbomethoxy (**4x**) groups.

Interestingly, alkene-containing product **4v** was obtained exclusively, with no trace of products arising from 5-*exo*-trig cyclization. This suggests that intermolecular trapping with an alkenyl trifluoroborate salt is more facile than cyclization,^26^ possibly due to preassociation of the positively charged sulfonium salt and negatively charged trifluoroborate salt leading to enhanced rates of cross-coupling.^27^

**Scheme 5:**
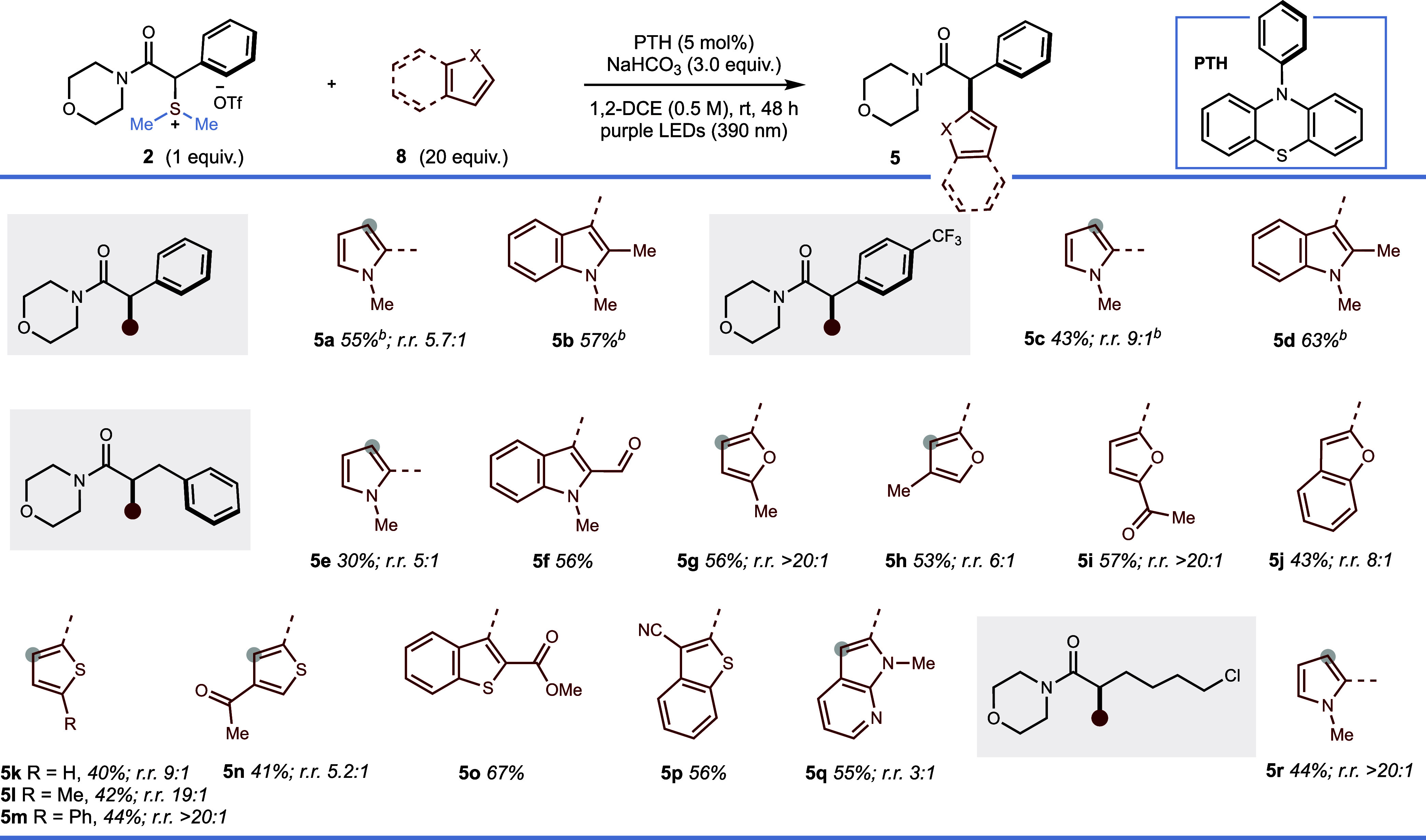
Reaction Scope for the Cross-Coupling of α-amido Sulfonium Salts **2** with Heteroaryl Partners **8** Reaction conditions: **2** (0.10 mmol), **8** (2.0 mmol), NaHCO_3_ (0.3 mmol), PTH (0.005 mmol) in 1,2-DCE (0.20 mL) irradiated at 390 nm (Purple Kessil lamp). Reaction time of 16 h.

α-Aryl carbonyl motifs are prevalent substructures in drugs and natural products.^5a,28^ After minimal retuning of reaction conditions, metal-free cross-coupling of α-amido sulfonium salts **2** and heteroaryl partners **8** was found to proceed in moderate to good yield (Scheme 5). Of note, heterocyclic partners are challenging substrates for transition metal-catalyzed α-arylation protocols due to their poisoning of the metal catalyst.^29^ Furthermore, our approach utilizes nonprefunctionalized heteroaromatic partners in formal C–H functionalization processes.^29c^ The coupling of α-amido sulfonium salts, bearing both α-aryl and α-alkyl groups, with *N*-methyl pyrrole **8a** gave products in up to 55% yield and up to >20:1 regioisomeric ratio in favor of addition to the 2-position (**5a**, **5c**, **5e, 5r**). Coupling with 1,2-dimethyl-1*H*-indole gave products of coupling at the 3-position in improved yields of up to 63% (**5b**, **5d**). Working with an α-alkyl-substituted α-amido sulfonium salt, we further explored the scope regarding the heteroaryl coupling partner. In addition to pyrrole and indole (**5f**), the protocol embraces several other heteroaromatic architectures of medicinal relevance; furan (**5g**–**i**), benzofuran (**5j**), thiophene (**5k**–**n**), benzothiophene (**5o** and **5p**), and azaindole (**5q**).

**Scheme 6:**
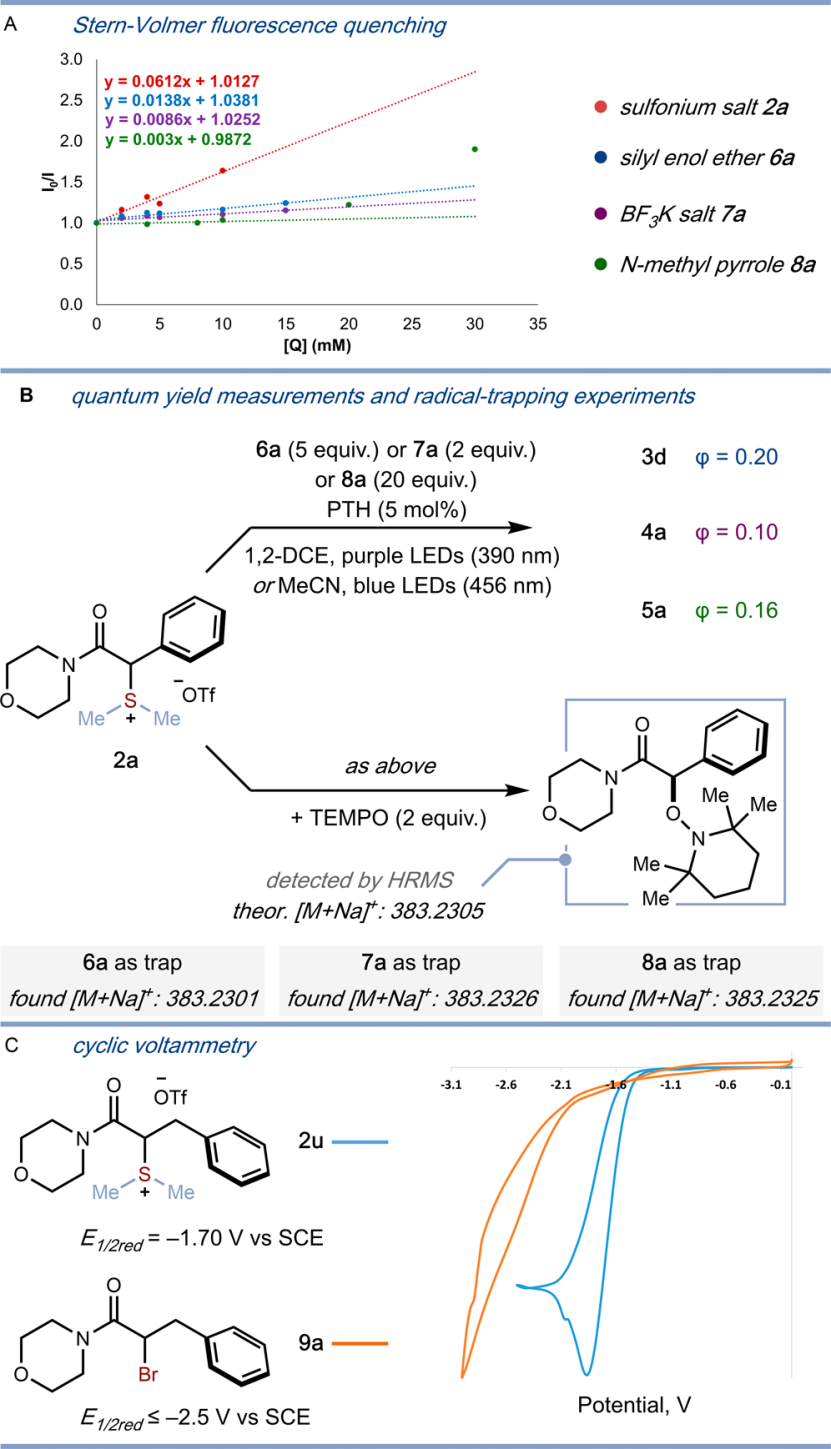
(A) Preliminary Mechanistic Studies on the α-alkylation, α-alkenylation, and α-arylation of α-amido Sulfonium Salts **2**; Stern–Volmer Quenching Studies of PTH Fluorescence by α-amido Sulfonium Salt **2a**, Silyl Enol Ether **6a**, Styrenyl Trifluoroborate Salt **7a**, and *N*-methyl Pyrrole **8a;** (B) Quantum Yield Measurements (top) and Radical-Trapping Experiments (bottom); (C) Normalized CV Spectra Comparing the Reduction Potentials of the α-amido Sulfonium Salt **2u** and the Corresponding α-bromo Amide **9a**

Stern–Volmer quenching studies support SET from the excited PTH photocatalyst to the sulfonium salt (Scheme 6A); in line with the relative reduction potentials, PTH fluorescence is quenched more rapidly by the α-amido sulfonium salt **2a** than by the silyl enol ether (**6a**), alkenyl trifluoroborate salt (**7a**), and heteroaromatic (**8a**) coupling partners. The proposed, closed photocatalytic cycle set out in Scheme 3A (rather than a radical chain mechanism) is supported by quantum yield measurements; φ = 0.20, 0.10, and 0.16 have been measured for the α-alkylation, α-alkenylation, and α-arylation reactions, respectively (Scheme 6B, top). The radical nature of the process is supported by the suppression of any reactivity, in all three transformations, when the reactions are conducted in the presence of the radical scavenger TEMPO (Scheme 6B, bottom). A comparison between the reduction potentials of α-amido sulfonium salt **2a** and the corresponding α-bromo amide **9a** highlights the ability of largely unexplored α-amido sulfonium salts to engage in chemodivergent α-functionalization under mild, metal-free photocatalytic conditions (Scheme 6C).

## Conclusions

In summary, α-amido sulfonium salts, prepared directly from tertiary amides, are bench-stable precursors of α-amidyl radicals under mild metal-free photocatalytic conditions. These largely unexplored sulfonium salts engage in C(sp^3^)–C(sp^2^) and C(sp^3^)–C(sp^3^) bond formation in cross-coupling reactions that realize the formal α-alkylation, α-alkenylation, and α-heteroarylation of tertiary amides. Preliminary mechanistic experiments support a photocatalytic cycle in which the sulfonium salts are reduced to form α-amidyl radicals, which couple with various electron-rich partners in a chemodivergent metal-free platform for the introduction of complexity in amides.

## Supporting Information Available

Additional experiments, experimental procedures, characterization data, X-ray data, and NMR spectra for all new compounds. (PDF)

## References

[ref1] Pitzer J.; Steiner K. Amides in Nature and Biocatalysis. J. Biotechnol. 2016, 235 (10), 32–46. 10.1016/j.jbiotec.2016.03.023. 26995609

[ref2] Pattabiraman V. R.; Bode J. W. Rethinking Amide Bond Synthesis. Nature 2011, 480, 471–479. 10.1038/nature10702. 22193101

[ref3] Seavill P. W.; Wilden J. D. The Preparation and Applications of Amides Using Electrosynthesis. Green Chem. 2020, 22, 7737–7759. 10.1039/D0GC02976A.

[ref4] Massolo E.; Pirola M.; Benaglia M. Amide Bond Formation Strategies: Latest Advances on a Dateless Transformation. Eur. J. Org. Chem. 2020, 2020, 4641–4651. 10.1002/ejoc.202000080.

[ref5] aSarabu R.; Bizzarro F. T.; Corbett W. L.; Dvorozniak M. T.; Geng W.; Grippo J. F.; Haynes N.-E.; Hutchings S.; Garofalo L.; Guertin K. R.; Hillard D. W.; Kabat M.; Kester R. F.; Ka W.; Liang Z.; Mahaney P. E.; Marcus L.; Matschinsky F. M.; Moore D.; Racha J.; Radinov R.; Ren Y.; Qi L.; Pignatello M.; Spence C. L.; Steele T.; Tengi J.; Grimsby J. Discovery of Piragliatin—First Glucokinase Activator Studied in Type 2 Diabetic Patients. J. Med. Chem. 2012, 55 (16), 7021–7036. 10.1021/jm3008689. 22809456

[ref6] Oare D. A.; Henderson M. A.; Sanner M. A.; Heathcock C. H. Acyclic Stereoselection. 46. Stereochemistry of the Michael Addition of *N*,*N*-disubstituted Amide and Thioamide Enolates to α,β-Unsaturated Ketones. J. Org. Chem. 1990, 55 (1), 132–157. 10.1021/jo00288a027.

[ref7] Rablen P. R.; Bentrup K. H. Are the Enolates of Amides and Esters Stabilized by Electrostatics?. J. Am. Chem. Soc. 2003, 125 (8), 2142–2147. 10.1021/ja029102a. 12590542

[ref8] aFantinati A.; Zanirato V.; Marchetti P.; Trapella C. The Fascinating Chemistry of α-Haloamides. ChemistryOpen 2020, 9 (2), 100–170. 10.1002/open.201900220. 32025460 PMC6996577

[ref9] Bordwell F. G.; Fried H. E. Acidities of the hydrogen-carbon protons in carboxylic esters, amides, and nitriles. J. Org. Chem. 1981, 46 (22), 4327–4331. 10.1021/jo00335a001.

[ref10] aShaughnessy K. H.; Hamann B. C.; Hartwig J. F. Palladium-Catalyzed Inter- and Intramolecular α-Arylation of Amides. Application of Intramolecular Amide Arylation to the Synthesis of Oxindoles. J. Org. Chem. 1998, 63 (19), 6546–6553. 10.1021/jo980611y.

[ref11] aObligacion J. V.; Hopmann K. H. Our Cup of Tea: Sustainable Organometallic Chemistry. Organometallics 2022, 41 (14), 1739–1742. 10.1021/acs.organomet.2c00294.

[ref12] aKaiser D.; Bauer A.; Lemmerer M.; Maulide N. Amide Activation: an Emerging Tool for Chemoselective Synthesis. Chem. Soc. Rev. 2018, 47 (21), 7899–7925. 10.1039/C8CS00335A. 30152510

[ref13] aPeng B.; Geerdink D.; Farès C.; Maulide N. Chemoselective Intermolecular α-Arylation of Amides. Angew. Chem., Int. Ed. 2014, 53 (21), 5462–5466. 10.1002/anie.201402229. 24740762

[ref14] Niu Z.-J.; Li L.-H.; Li X.-S.; Liu H.-C.; Shi W.-Y.; Liang Y.-M. Formation of *o*-Allyl- and Allenyl-Modified Amides via Intermolecular Claisen Rearrangement. Org. Lett. 2021, 23 (4), 1315–1320. 10.1021/acs.orglett.0c04300. 33534590

[ref15] Kaiser D.; Teskey C. J.; Adler P.; Maulide N. Chemoselective Intermolecular Cross-Enolate-Type Coupling of Amides. J. Am. Chem. Soc. 2017, 139 (45), 16040–16043. 10.1021/jacs.7b08813. 29099184 PMC5691317

[ref16] aGonçalves C. R.; Lemmerer M.; Teskey C. J.; Adler P.; Kaiser D.; Maryasin B.; Gonzáles L.; Maulide N. Unified Approach to the Chemoselective α-Functionalization of Amides with Heteroatom Nucleophiles. J. Am. Chem. Soc. 2019, 141 (46), 18437–18443. 10.1021/jacs.9b06956. 31714077 PMC6879173

[ref17] Li J.; Berger M.; Zawodny W.; Simaan M.; Maulide N. A Chemoselective α-Oxytriflation Enables the Direct Asymmetric Arylation of Amides. Chem 2019, 5 (7), 1883–1891. 10.1016/j.chempr.2019.05.006.

[ref18] aRathke M. W.; Lindert A. Reaction of ester enolates with copper(II) salts. Synthesis of substituted succinate esters. J. Am. Chem. Soc. 1971, 93 (18), 4605–4606. 10.1021/ja00747a051.

[ref19] aWang L.; Wei X.-J.; Jia W.-L.; Zhong J.-J.; Wu L.-Z.; Liu Q. Visible-Light-Driven Difluoroacetamidation of Unactive Arenes and Heteroarenes by Direct C–H Functionalization at Room Temperature. Org. Lett. 2014, 16 (22), 5842–5845. 10.1021/ol502676y. 25369540

[ref20] aBasch C. H.; Liao J.; Jianyu L.; Xu J.; Piane J. J.; Watson M. P. Harnessing Alkyl Amines as Electrophiles for Nickel-Catalyzed Cross Couplings via C–N Bond Activation. J. Am. Chem. Soc. 2017, 139 (15), 5313–5316. 10.1021/jacs.7b02389. 28359153 PMC5480296

[ref21] aZhu Z.-F.; Chen G.-L.; Liu F. Ruthenium-Catalysed *meta*-Selective C_Ar_–H Bond Alkylation via a Deaminative Strategy. Chem. Commun. 2021, 57 (27), 3411–3414. 10.1039/D1CC00039J. 33687414

[ref22] aPéter Á.; Perry G. J. P.; Procter D. J. Radical C–C bond formation using sulfonium salts and light. Adv. Synth. Catal. 2020, 362 (11), 2135–2142. 10.1002/adsc.202000220.

[ref23] aAukland M. H.; Šiaučiulis M.; West A.; Perry G. J. P.; Procter D. J. Metal-free Photoredox-Catalysed Formal C–H/C–H Coupling of Arenes Enabled by Interrupted Pummerer Activation. Nat. Catal. 2020, 3, 163–169. 10.1038/s41929-019-0415-3.

[ref24] Leypold M.; D’Angelo K. A.; Movassaghi M. Chemoselective α-Sulfidation of Amides Using Sulfoxide Reagents. Org. Lett. 2020, 22 (22), 8802–8807. 10.1021/acs.orglett.0c03160. 33048547 PMC7680396

[ref25] Deposition numbers 2362276 (for **2a**), 2362286 (for **2c**), 2362287 (for **2l**), and 2362275 (for **2u**) contain the supplementary crystallographic data for this paper. These data are provided free of charge by the joint Cambridge Crystallographic Data Centre and Fachinformationszentrum Karlsruhe Access Structures service.

[ref26] aBeckwith A. L. J.; Schiesser C. H. Regio- and Stereo-Selectivity of Alkenyl Radical Ring Closure: a Theoretical Study. Tetrahedron 1985, 41 (19), 3925–3941. 10.1016/S0040-4020(01)97174-1.

[ref27] aGillespie J. E.; Fanourakis A.; Phipps R. J. Strategies That Utilize Ion Pairing Interactions to Exert Selectivity Control in the Functionalization of C–H Bonds. J. Am. Chem. Soc. 2022, 144 (40), 18195–18211. 10.1021/jacs.2c08752. 36178308 PMC9562467

[ref28] Shen T. Y. Perspectives in Nonsteroidal Anti-inflammatory Agents. Angew. Chem., Int. Ed. 1972, 11 (6), 460–472. 10.1002/anie.197204601. 4626261

[ref29] aLiu Y.-J.; Xu H.; Kong W.-J.; Shang M.; Dai H.-X.; Yu J.-Q. Overcoming the Limitations of Directed C–H Functionalizations of Heterocycles. Nature 2014, 515, 389–393. 10.1038/nature13885. 25383516 PMC4248606

