# Supplementary material for: α-Amido Sulfonium Salts Provide a Platform for Photocatalytic Metal-Free Carbon–Carbon Bond Formation in Amides
Source: ACS Catal. 2025 May 5;15(10):8345–52. doi: 10.1021/acscatal.5c02029 (PMC12090209; doi:10.1021/acscatal.5c02029)
Supplement: Supplementary file 1 — cs5c02029_si_001.pdf [file cs5c02029_si_001.pdf]

α-Amido Sulfonium Salts Provide a Platform for Photocatalytic Metal-free Carbon-Carbon Bond Formation in Amides

*Leendert van Dalsen, Shibo Zhang, Wei Tian†, Ben W. Joynson†, Ciro Romano and David J. Procter**

†Authors Contributed Equally

Department of Chemistry, University of Manchester, Oxford Road, Manchester, M13 9PL (UK)

**Corresponding author. email: david.j.procter@manchester.ac.uk*

Supporting Information

1 General Information 3

2 Reaction Optimization 4

2.1 Synthesis of α-Amido Sulfonium Salts 4

2.2 Optimization of Photochemical Alkylation 6

2.3 Optimization of Photochemical Alkenylation 9

2.4 Optimization of Photochemical (Hetero)arylation 14

3 Synthesis of Starting Materials 20

3.1 General Procedure 1 (GP-1): Amide coupling *via* acid chlorides 20

3.2 General Procedure 2 (GP-2): Amide coupling *via* carboxylic acids 20

3.3 General Procedure 3 (GP-3): Amide coupling with medicinally-relevant amines 21

3.4 Synthesis of Sulfoxides 46

3.5 Synthesis of Photocatalyst 47

3.6 Synthesis of Silyl Enol Ethers 48

3.7 Synthesis of Alkenyl Trifluoroborates 49

4 Synthesis of Amido Sulfonium Salts 55

4.1 General Procedure 4 (GP-4): Synthesis of Amido Sulfonium Salts 55

5 Functionalization of α-Amido Sulfonium Salts 84

5.1 General Procedure 5 (GP-5): Photochemical α-Alkylation 84

5.2 General Procedure 6 (GP-6): Photocatalytic α-Alkenylation 104

5.3 General Procedure 7 (GP-7): Photochemical α-(Hetero)arylation 130

6 Reaction Limitations 149

7 Mechanistic Investigations 151

7.1 Cyclic Voltammetry 151

7.2 Quantum Yield Measurements 155

7.3 Stern-Volmer Fluorescence Quenching 165

8 X-Ray Structures 172

9 References 181

10 NMR Spectra 183

1 General Information

Reagents were purchased from commercial suppliers and used as received. Procedures requiring inert conditions were conducted in flame- or oven-dried glassware under an atmosphere of anhydrous dinitrogen using standard Schlenk techniques. All solvents were purchased as 99.8% purity. Anhydrous solvents were purchased from Sigma-Aldrich and used as supplied. Deuterated chloroform was stored over 4 Å molecular sieves. Photochemical reactions were subjected to irradiation from either 34 W 390 nm Kessil PR160L or Tuna Blue Kessil A160WE lamps, with the reaction vessel placed approximately 5 cm from the light source with fan cooling.

NMR spectra were recorded at 298 K (^{1}H, 500 / 400 MHz; ^{13}C, 125 / 101 MHz; ^{19}F, 471 / 376 MHz). Chemical shifts (δ) are reported in ppm; coupling constants, *J*, are reported in Hz. Signals are reported as singlet (s), doublet (d), triplet (t), quartet (q), septet (sept), multiplet (m), broad (br), apparent (app) and combinations thereof. Chemical shifts are reported relative to tetramethylsilane (TMS) and referenced to the appropriate residual solvent peaks for ^{1}H and ^{13}C{^{1}H} NMR respectively:

$CDCl_3$: 7.26 ppm, 77.16 ppm.

CD_3CN: 1.94 ppm, 1.32 ppm.

High-resolution mass spectra were obtained using electron impact ionization (EI) and chemical ionization (CI), or electrospray ionization (ESI). Column chromatography was accomplished using silica gel 60 Å (40-60 μm particle size) used as purchased from Sigma-Aldrich. Analytical thin-layer chromatography was carried out on aluminium-backed silica gel plates (Merck/EMD Millipore, 60 Å pore size, precoated with a 254 nm responsive fluorescent dye). Compounds were visualized by exposure to UV radiation (254 nm) or by staining with potassium permanganate followed by heating.

2 *Reaction Optimization*

2.1 Synthesis of α-Amido Sulfonium Salts

Purification of sulfonium salts

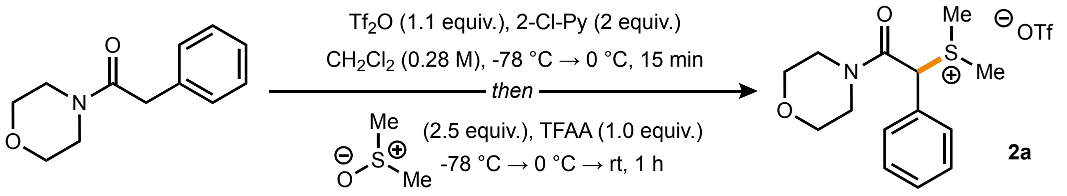

Entry	Work-up	Purification	Isolated Yield / %
1	None	Column Chromatography	52
2	K_2CO_3 plug, then hexane trituration	Column Chromatography	46
3	NaOAc plug, then hexane trituration	Column Chromatography	45
4	K_2CO_3 plug, then hexane trituration	Silica plug, then recrystallization (EtOAc)	38
5	K_2CO_3 plug, then hexane trituration	Recrystallization (EtOAc)	54
6	Aq. $NaHCO_3$, then hexane trituration	Recrystallization (EtOAc)	59
7	Aq. $NaHCO_3$	Column Chromatography	74

Supplementary Table S1: *Optimization of the work-up and purification in the formation of amido sulfonium salts.*

Discussion: In general, column chromatography was utilized to achieve the highest yield and purity, though trituration from hexanes followed by recrystallization also achieved high purity at the expense of yield. Sulfonium salts bearing more electron-withdrawing substituents were in general more crystalline and could be isolated by trituration/recrystallization with higher isolated yields.

Reaction Robustness

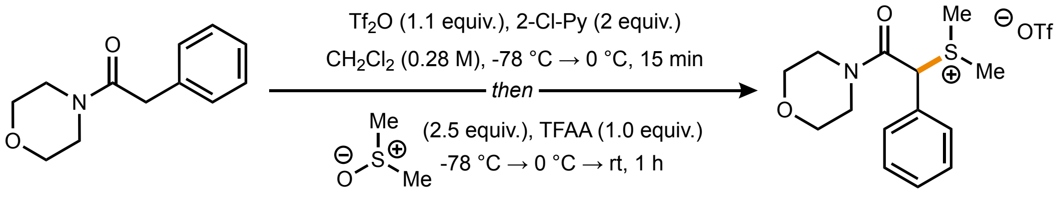

2a

Entry	Conditions	Yield / %[a]
1	Anhydrous CH_2Cl_2, inert atmosphere	88
2	Winchester-grade CH_2Cl_2, inert atmosphere	80
3	Winchester-grade CH_2Cl_2, air	60

Supplementary Table S2: *Solvent grade tests for α-amido sulfonium salt synthesis. [a]Determined by ^1H NMR spectroscopy using CH_2Br_2 as an internal standard.*

2.2 Optimization of Photochemical Alkylation

Solvent Screen

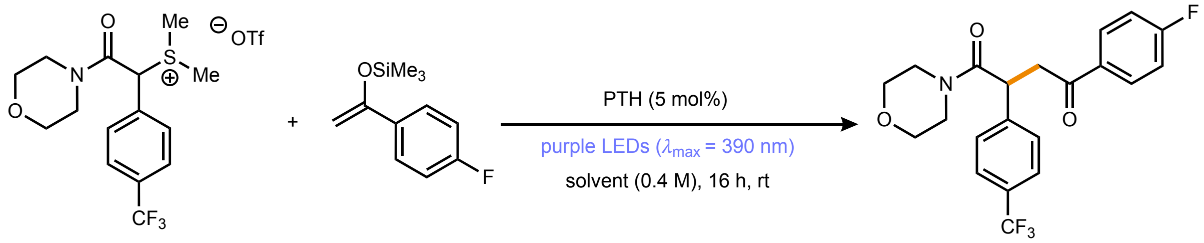

2l **6a** (5 equiv.) **3d**

Entry	Solvent	Yield of 3d / %[a]
1	MeCN	65
2	DMF	72 (70)
3	DMA	62
4	DMSO	47
5	CH_2Cl_2	66
6	1,2-DME	68
7	EtOAc	69
8	THF	60
9	1,4-dioxane	67
10	1,2-DCE	76 (72)
11	Acetone	<5
12	MeOH	<5
13	$CHCl_3$	22
14	H_2O	10

Supplementary Table S3: *Solvent optimization of the photochemical alkylation of amides. [a]Determined by ^1H NMR spectroscopy using $MeNO_2$ as an internal standard. Isolated yields in parentheses.*

Further Optimization

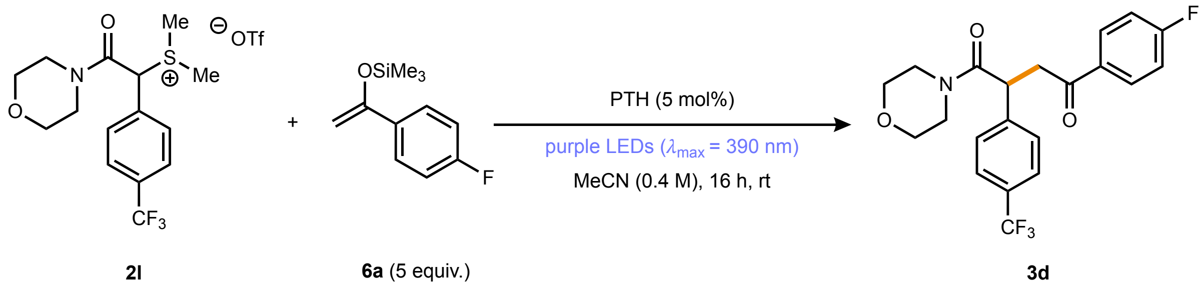

Entry	$[2l]_0$ / M	Equivs. of 6a	PTH loading	Yield of 3d / %[a]
1	0.4	5	5 mol%	76 (72)
2	0.1	5	5 mol%	60
3	0.2	5	5 mol%	64
4	0.3	5	5 mol%	65
5	0.5	5	5 mol%	70
6	0.4	1	5 mol%	23
7	0.4	2	5 mol%	34
8	0.4	10	5 mol%	78
9	0.4	5	2 mol%	71
10	0.4	5	10 mol%	57
11	0.4	5	25 mol%	36

Supplementary Table S4: *Further optimization of the photochemical alkylation of amides. [a]Determined by ^1H NMR spectroscopy using $MeNO_2$ as an internal standard. Isolated yields in parentheses.*

Control Reactions

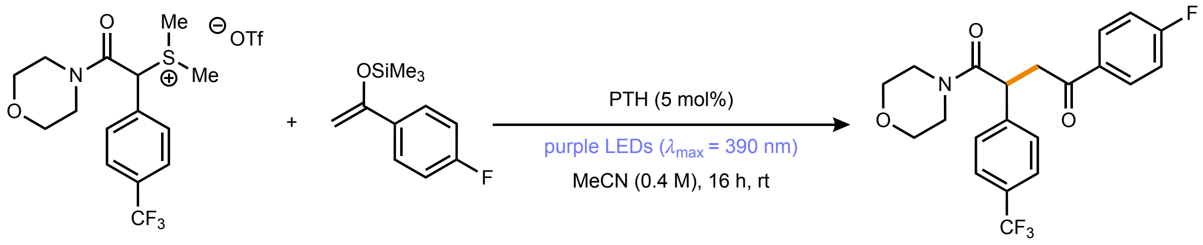

2l **6a** (5 equiv.) **3d**

Entry	Deviation from above	2l remaining / %[a]	Yield of 3d / %[a]
1	No photocatalyst	86	6
2	No light	99	-
3	No light, 60 °C	95	-
4	2 equiv. TEMPO	<5	<5
5	Irradiation (456 nm)	26	44

Supplementary Table S5: *Control experiments for the photochemical alkylation of amides.* [a]*Determined by* ^{1}H *NMR spectroscopy using* $MeNO_2$ *as an internal standard. Isolated yields in parentheses.*

2.3 Optimization of Photochemical Alkenylation

Investigation of Light-mediated Alkene Isomerization

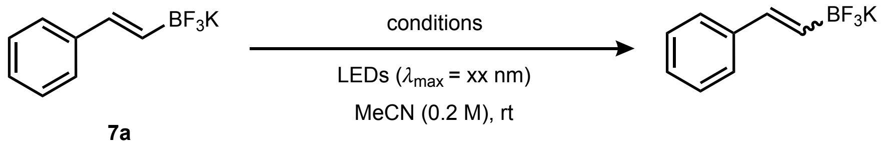

7a

Entry	Irradiation wavelength / nm	Conditions	*E*:*Z* ratio of 7a[a]
1	-	As purchased	>20:1
2	390	16 h, no PTH	11.5:1
3	390	16 h, 10 mol% PTH	9:1
4	456[b]	16 h, no PTH	>20:1
5	456[b]	16 h, 10 mol% PTH	>20:1
6	390, 25% intensity	16 h, 5 mol% PTH	>20:1

Supplementary Table S6: *Alkenyl BF_3K salt isomerization studies for the photochemical alkenylation of amides. [a]Determined by ^1H NMR spectroscopy using $MeNO_2$ as an internal standard. [b]Using broad wavelength Kessil Tuna Blue LEDs.*

Discussion:

The use of Kessil Tuna Blue LED lamps in the presence of the PTH photocatalyst resulted in little to no isomerization of the BF_3K salt starting material, leading to >20:1 E/Z ratios in the final products. The broad emission spectrum of the Tuna Blue lamps includes a small emission at 390 nm, resulting in photoexcitation of the PTH photocatalyst.

As Tuna Blue lamps are less prevalent in laboratory setting, 390 nm lamps can also be used but result in lower E/Z ratios in some cases. Typically, E/Z ratios can be improved by lowering the intensity of the 390 nm lamp to 25%, somewhat mimicking the small emission of the Tuna Blue lamps at this wavelength.

Alkenyl trifluoroborate Stoichiometry and Additive Optimization

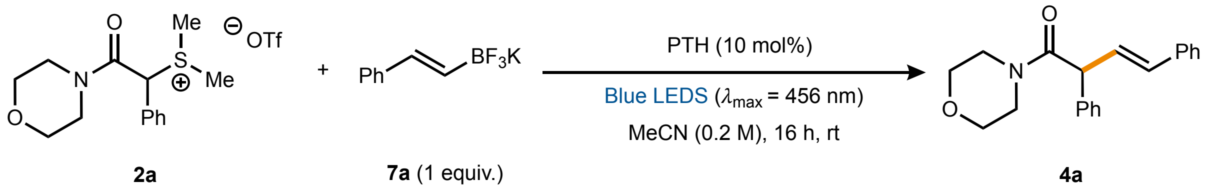

Entry	Deviations from above	Yield of 4a / %[a]	*E*:*Z* ratio of 4a[a]
1	None	59	8.8:1
2	1.1 equiv. **7a** + 1 equiv. KF	25	4:1
3	1.1 equiv. **7a** + 2 equiv. KF	<5	-
4	2 equiv. **7a**	84 (86)	18.5:1
5	3 equiv. **7a**	91	9.1:1

Supplementary Table S7: *Additive and trifluoroborate salt stoichiometry studies for the photochemical alkenylation of amides. [a]Determined by ^{1}H NMR spectroscopy using $MeNO_2$ as an internal standard. Isolated yields in parentheses.*

Solvent Optimization

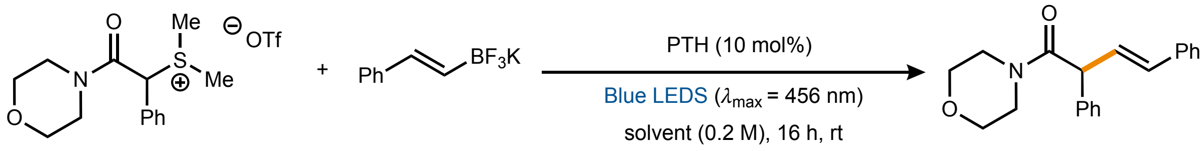

2a **7a** (2 equiv.) **4a**

Entry	Solvent	Yield of 4a / %[a]	*E*:*Z* ratio of 4a[a]
1	MeCN	84 (86)	18.5:1
2	DMF	38	1.9:1
3	DMA	53	2.8:1
4	DMSO	28	1:1
5	CH_2Cl_2	72	4.5:1
6	1,2-DME	41	4.9:1
7	EtOAc	31	>20:1
8	THF	63	1.2:1
9	1,2-DCE	73	6.1:1
10	Acetone	44	>20:1
11	MeOH	15	1.5:1

Supplementary Table S8: *Solvent optimization experiments for the photochemical alkenylation of amides.*
[a]Determined by ^1H NMR spectroscopy using $MeNO_2$ as an internal standard. Isolated yields in parentheses.

Concentration and Photocatalyst Optimization

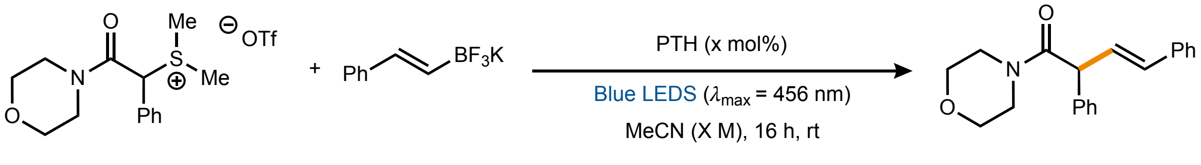

Entry	$[2a]_0$ / M	PTH loading	Yield of 4a / %[a]	*E*:*Z* ratio of 4a[a]
1	0.2	10 mol%	84 (86)	18.5:1
2	0.1	10 mol%	74	12.6:1
3	0.4	10 mol%	70	>20:1
4	0.2	5 mol%	85 (86)	12:1
5	0.2	25 mol%	67	18:1

Supplementary Table S9: *Reaction concentration and photocatalyst loading optimization for the alkenylation of amides. [a]Determined by ^1H NMR spectroscopy using $MeNO_2$ as an internal standard. Isolated yields in parentheses.*

Control Reactions

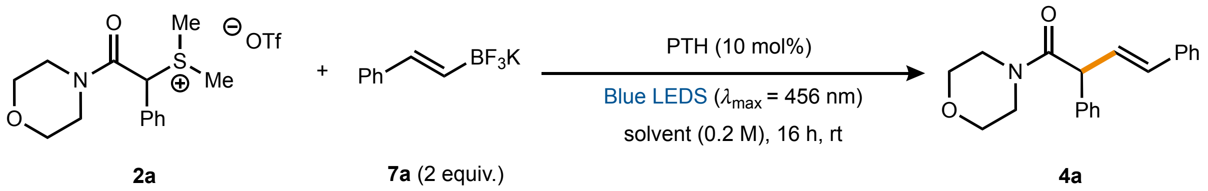

Entry	Deviation from above	2a remaining / %[a]	Yield of 4a / %[a]
1	No photocatalyst	89	<5
2	No light	98	<5
3	No light, 60 °C	94	<5
4	2 equiv. TEMPO	<5	<5
5	**2a** stored for 1 month in darkness	<5	74
6	**2a** stored for 1 week in ambient light[b]	<5	80

Supplementary Table S10: *Control experiments for the photochemical alkenylation of amides.* [a]*Determined by* ^{1}*H NMR spectroscopy using* $MeNO_2$ *as an internal standard. Isolated yields in parentheses.* [b]Sample of **2a** stored next to an east-facing window at 53.4668° N, 2.2339° W (Manchester, UK) during 03/07/24-10/07/24.

2.4 Optimization of Photochemical (Hetero)arylation

Initial Optimization

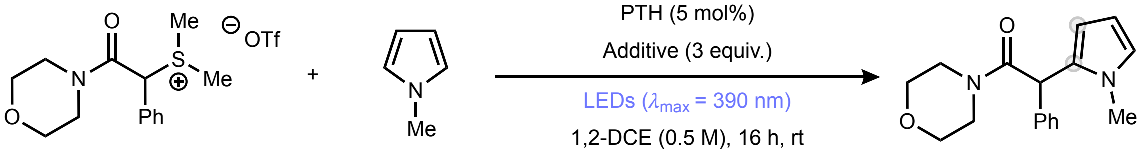

Entry	LED wavelength / nm	PTH Loading	Additive	Yield of 5a / %[a]
1	456	25 mol%	-	31
2	456	10 mol%	-	23
3	456	5 mol%	-	14
4	390	25 mol%	-	29
5	390	10 mol%	-	34
6	390	5 mol%	-	36
7	390	5 mol%	2,6-lutidine	35
8	390	5 mol%	NEt_3	5
9	390	5 mol%	NaOAc	55

Supplementary Table S11: *Reaction optimization for the photochemical heteroarylation of amides. [a]Determined by ^1H NMR spectroscopy using $MeNO_2$ as an internal standard. Isolated yields in parentheses.*

Additive Screen

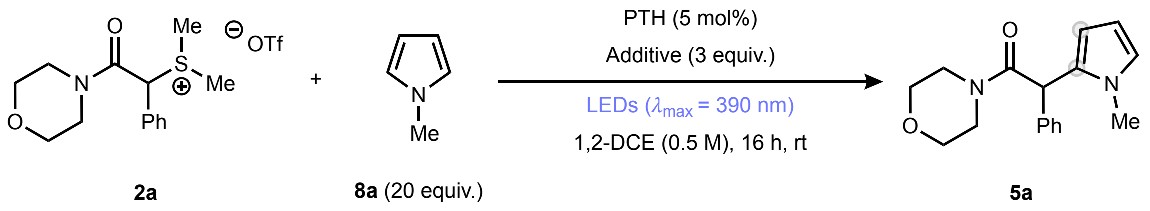

Entry	Additive	Yield of 5a / %[a]	Regioisomeric ratio (r.r.)[a]
1	K_2CO_3	50	3.5:1
2	Na_2CO_3	67	3.8:1
3	$NaHCO_3$	71 (58)	3.4:1
4	K_3PO_4	64	4.3:1

Supplementary Table S12: *Inorganic base screen for the photochemical heteroarylation of amides. [a]Determined by ^1H NMR spectroscopy using $MeNO_2$ as an internal standard. Isolated yields in parentheses.*

Solvent Optimization

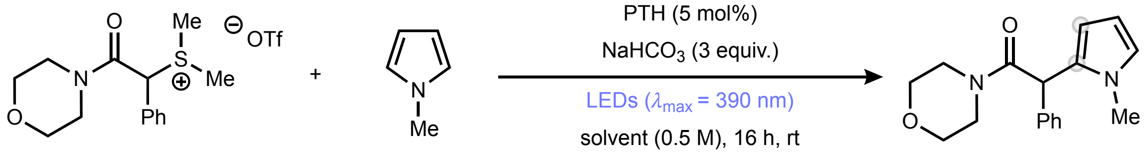

Entry	Solvent	Yield of 5a / %[a]	Regioisomeric ratio (r.r.)[a]
1	MeCN	54	4.9:1
2	1,2-DCE	60 (55)	3.8:1
3	CH_2Cl_2	48	3.4:1
4	EtOAc	46	4.3:1

Supplementary Table S13: *Solvent optimization for photochemical heteroarylation of amides. [a]Determined by ^{1}H NMR spectroscopy using $MeNO_2$ as an internal standard. Isolated yields in parentheses.*

Further Optimization

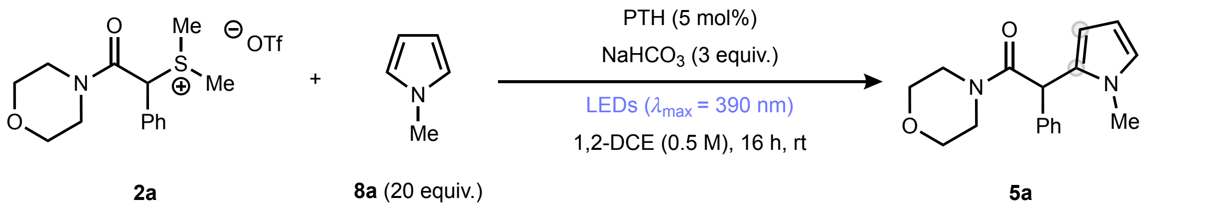

Entry	$[2a]_0$ / M	Equivs. of 8a	Equivs. of $NaHCO_3$	Yield of 5a / %[a]	Regioisomeric ratio (r.r.)[a]
1	0.4	20	3	60 (55)	5:1 (4.6:1 r.r.)
2	0.2	20	3	31	5.2:1
3	0.1	20	3	27	5.8:1
4	0.4	20	1	38	2.8:1
5	0.4	20	2	48	3.4:1
6	0.4	20	5	52	2.7:1
7	0.4	2	3	23	2.8:1
8	0.4	5	3	41	3.1:1
9	0.4	10	3	45	3.5:1

Supplementary Table S14: *Concentration and stoichiometry investigations for the photochemical heteroarylation of amides. [a]Determined by ^1H NMR spectroscopy using $MeNO_2$ as an internal standard. Isolated yields in parentheses.*

Sulfonium Salt Variation

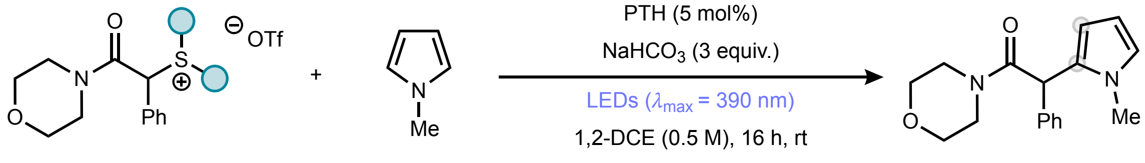

8a (20 equiv.) **5a**

Entry	Sulfur substituents	Yield of 5a / %[a]	Regioisomeric ratio (r.r.)[a]
1	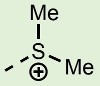 **(2a)**	60 (55)	5:1 (4.6:1 r.r.)
2	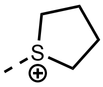 **(2b)**	32	3.7:1

Supplementary Table S15: *Investigation of the sulfur group for the photochemical heteroarylation of amides.*
[a]*Determined by* [1]*H NMR spectroscopy using* $MeNO_2$ *as an internal standard. Isolated yields in parentheses.*

While the sulfonium salts **2a** and **2b** possess similar reduction potentials (see Section 7.1), the tetrahydrothiophenium (THT) salt **2b** proved less efficient in the heteroarylation reaction with *N*-methylpyrrole. In addition to this, the THT salt **2b** also proved more difficult to handle and was often obtained as a viscous foam with greater hygroscopicity compared to the crystalline, stable salt **2a**.

Control Reactions

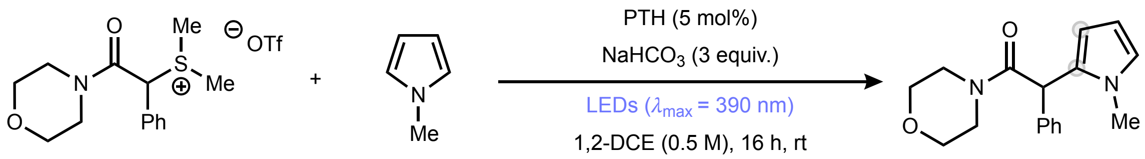

Entry	Deviation from above	2a remaining / %[a]	Yield of 5a / %[a]
1	No photocatalyst	94	<5
2	No light	90	<5
3	No light, 60 °C	87	<5
4	2 equiv. TEMPO	<5	<5

Supplementary Table S16: *Control experiments for the photochemical heteroarylation of amides. [a]Determined by ^1H NMR spectroscopy using $MeNO_2$ as an internal standard. Isolated yields in parentheses.*

3.1 General Procedure 1 (GP-1): Amide coupling *via* acid chlorides

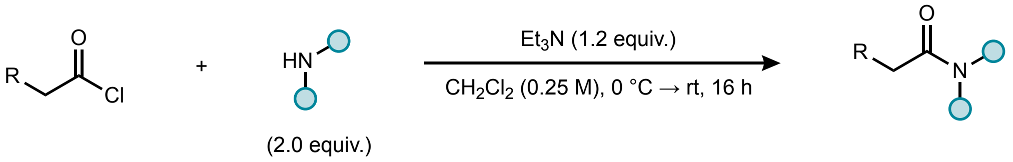

To a solution of secondary amine (10.0 mmol) and Et_3N (0.84 mL, 6.0 mmol) in CH_2Cl_2 (20 mL, 0.25 M) cooled to 0 °C (ice/water bath) was added the acid chloride (5.0 mmol) drop-wise. After complete addition, the reaction was allowed to warm to rt and stirred overnight. The reaction was quenched by the addition of 1 M HCl (10 mL) and the CH_2Cl_2 layer was separated. The aqueous layer was extracted with CH_2Cl_2 (3 × 10 mL) and the combined organics were washed with saturated aqueous $NaHCO_3$ (50 mL), dried over Na_2SO_4, and concentrated *in vacuo*. If required, purification by column chromatography with eluents given below afforded the pure product.

3.2 General Procedure 2 (GP-2): Amide coupling *via* carboxylic acids

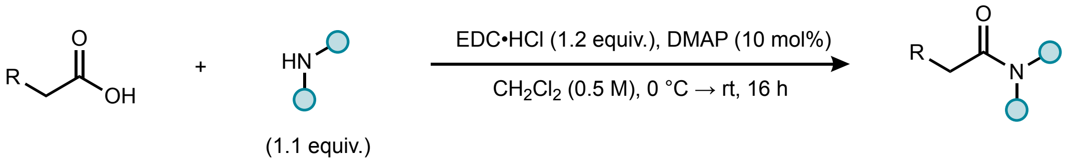

To a solution of carboxylic acid (5.0 mmol) in CH_2Cl_2 (10 mL, 0.5 M) cooled to 0 °C (ice/water bath) was added secondary amine (5.5 mmol), EDC•HCl (1.15 g, 6.0 mmol), and DMAP (61 mg, 0.5 mmol), sequentially. The reaction was allowed to warm to rt and stirred overnight. The reaction was quenched by the addition of 1 M HCl (10 mL) and the CH_2Cl_2 layer was separated. The aqueous layer was extracted with CH_2Cl_2 (3 × 10 mL) and the combined organics were washed with saturated aqueous $NaHCO_3$ (50 mL), dried over Na_2SO_4, and concentrated *in vacuo*. If required, purification by column chromatography with eluents given below afforded the pure product.

3.3 General Procedure 3 (GP-3): Amide coupling with medicinally-relevant amines

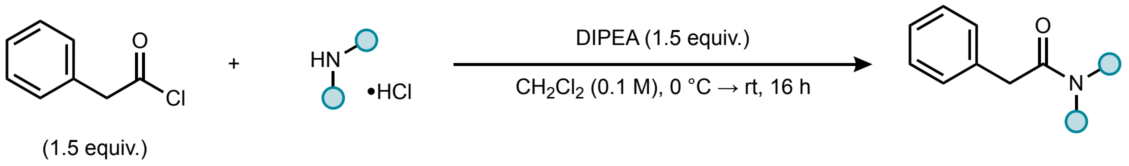

According to the modified literature procedure,[1] to a solution of amine hydrochloride (2.0 mmol) in CH_2Cl_2 (20 mL, 0.1 M) cooled to 0 °C (ice/water bath) was added DIPEA (0.52 mL, 3.0 mmol) followed by phenylacetyl chloride (0.40 mL, 3.0 mmol) drop-wise. The reaction was allowed to warm to rt and stirred overnight. The reaction was quenched by the addition of either 1 M HCl (10 mL) or saturated aqueous $NaHCO_3$ (10 mL) for amides with basic functional groups, and the CH_2Cl_2 layer was separated. The aqueous layer was extracted with CH_2Cl_2 (3 × 10 mL) and the combined organics were washed with saturated aqueous $NaHCO_3$ (50 mL), dried over Na_2SO_4, and concentrated *in vacuo*. Purification by column chromatography with eluents given below afforded the pure product.

2-Phenyl-1-morpholinoethan-1-one

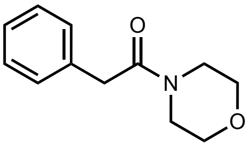

$C_{12}H_{15}NO_2$
MW: 205.26

Synthesised according to **GP-1** from 2-phenylacetyl chloride (6.6 mL, 50.0 mmol), morpholine (8.6 mL, 100.0 mmol), and Et_3N (8.4 mL, 60.0 mmol) in CH_2Cl_2 (150 mL). Purification by column chromatography (silica gel; EtOAc) afforded the product as a white solid (9.22 g, 44.9 mmol, 90%).

^{1}H NMR (400 MHz, $CDCl_3$): δ 7.35 – 7.30 (m, 2H, Ar *H*), 7.28 – 7.22 (m, 3H, Ar *H*), 3.74 (s, 2H, PhCH_2), 3.65 (s, 4H, 2 x CH_2), 3.50 – 3.41 (m, 4H, 2 x CH_2).

^{13}C{^{1}H} NMR (101 MHz, $CDCl_3$): δ 169.8 (*C*=O), 134.9 (Ar *C*), 129.0 (Ar *C*H), 128.7 (Ar *C*H), 127.1 (Ar *C*H), 67.0 (*C*H_2), 66.6 (*C*H_2), 46.7 (*C*H_2), 42.3 (*C*H_2), 41.0 (Ph*C*H_2).

HRMS: calcd. for $C_{12}H_{15}NO_2Na$ $[M+Na]^+$: 228.0995; found (ESI$^+$): 228.0996.

Characterization data are consistent with literature values.[2]

2-(4-Trifluoromethylphenyl)-1-morpholinoethan-1-one

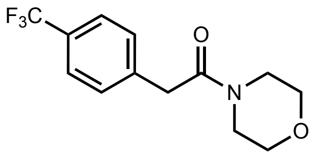

$C_{13}H_{14}F_3NO_2$
MW: 273.26

Synthesised according to **GP-1** from 2-(4-trifluoromethylphenyl)acetyl chloride (1.32 mL, 10.0 mmol), morpholine (1.73 mL, 10.0 mmol), and Et_3N (1.67 mL, 12.0 mmol) in CH_2Cl_2 (80 mL). Purification by column chromatography (silica gel; EtOAc) afforded the product as a white solid (1.77 g, 8.02 mmol, 80%).

^{1}H NMR (400 MHz, $CDCl_3$): δ 7.59 (d, *J* = 8.0 Hz, 2H, Ar *H*), 7.36 (d, *J* = 8.0 Hz, 2H, Ar *H*), 3.77 (s, 2H, ArC*H*$_2$), 3.69 – 3.61 (m, 4H, 2 x C*H*$_2$), 3.59 – 3.41 (m, 4H, 2 x C*H*$_2$).

^{13}C{^{1}H} NMR (101 MHz, $CDCl_3$): δ 168.9 (*C*=O), 139.0 (Ar *C*), 129.3 (Ar *C*H), 129.5 (q, *J* = 32.8 Hz, Ar *C*), 125.8 (q, *J* = 3.8 Hz, Ar *C*H), 124.2 (q, *J* = 272.1 Hz, *C*F_3), 66.9 (*C*H_2), 66.6 (*C*H_2), 46.6 (*C*H_2), 42.4 (*C*H_2), 40.4 (Ar*C*H_2).

^{19}F NMR (376 MHz, $CDCl_3$): δ -62.53 (s, C*F*$_3$).

HRMS: calcd. for $C_{13}H_{14}NO_2F_3Na$ $[M+Na]^+$: 296.0869; found (ESI$^+$): 296.0864.

Characterization data are consistent with literature values.[3]

2-(4-Fluorophenyl)-1-morpholinoethan-1-one

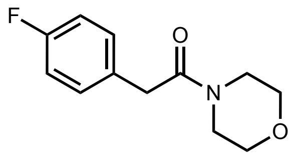

$C_{12}H_{14}FNO_2$
MW: 223.25

Synthesised according to **GP-1** from 2-(4-fluorophenyl)acetyl chloride (0.69 mL, 5.0 mmol), morpholine (0.86 mL, 10.0 mmol), and Et_3N (0.84 mL, 6.0 mmol) in CH_2Cl_2 (20 mL). The pure product was afforded without further purification as a white solid (1.04 g, 4.67 mmol, 93%).

^{1}H NMR (400 MHz, $CDCl_3$): δ 7.23 – 7.17 (m, 2H, Ar *H*), 7.06 – 6.97 (m, 2H, Ar *H*), 3.69 (s, 2H, ArCH_2), 3.69 – 3.63 (m, 4H, 2 × CH_2), 3.52 (dd, *J* = 5.6, 3.7 Hz, 2H, CH_2), 3.44 (dd, *J* = 5.6, 3.7 Hz, 2H, CH_2).

^{13}C{^{1}H} NMR (101 MHz, $CDCl_3$): δ 169.6 (*C*=O), 162.0 (d, *J* = 245.5 Hz, Ar *C*F), 130.6 (d, *J* = 3.4 Hz, Ar *C*), 130.3 (d, *J* = 8.1 Hz, Ar *C*H), 115.8 (d, *J* = 21.4 Hz, Ar *C*H), 66.9 (*C*H_2), 66.6 (*C*H_2), 46.6 (*C*H_2), 42.3 (*C*H_2), 39.9 (Ar*C*H_2).

^{19}F NMR (376 MHz, $CDCl_3$): δ -115.84 (tt, *J* = 13.9, 5.3 Hz).

HRMS: calcd. for $C_{12}H_{14}FNO_2Na$ $[M+Na]^+$: 246.0901; found (ESI$^+$): 246.0905.

Characterization data are consistent with literature values.[3]

2-(4-Methoxyphenyl)-1-morpholinoethan-1-one

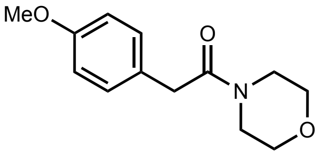

$C_{13}H_{17}NO_3$
MW: 235.28

Synthesised according to **GP-2** from 2-(4-methoxyphenyl)acetic acid (3.32 g, 20.0 mmol), morpholine (1.90 mL, 22.0 mmol), EDC•HCl (4.60 g, 24.0 mmol), and DMAP (244 mg, 2.0 mmol) in CH_2Cl_2 (80 mL). Purification by column chromatography (silica gel; 20-50% EtOAc in hexanes) afforded the product as a white solid (4.21 g, 17.9 mmol, 90%).

^{1}H NMR (400 MHz, $CDCl_3$): δ 7.14 (d, J = 8.7 Hz, 2H, Ar H), 6.85 (d, J = 8.7 Hz, 2H, Ar H), 3.79 (s, 3H, OCH_3), 3.66 (s, 2H, ArCH_2), 3.63 (br s, 4H, 2 x CH_2), 3.51 – 3.39 (m, 4H, 2 x CH_2).

^{13}C{^{1}H} NMR (101 MHz, $CDCl_3$): δ 170.1 (C=O), 158.6 (Ar C), 129.7 (Ar CH), 126.9 (Ar C), 114.3 (Ar CH), 66.9 (CH$_2$), 66.6 (CH$_2$), 55.4 (OCH$_3$) 46.6 (CH$_2$), 42.4 (CH$_2$), 40.0 (ArCH$_2$).

HRMS: calcd. for $C_{13}H_{17}NO_3Na$ $[M+Na]^+$: 258.1106; found (ESI$^+$): 258.1101.

Characterization data are consistent with literature values.[3]

2-(4-Methylphenyl)-1-morpholinoethan-1-one

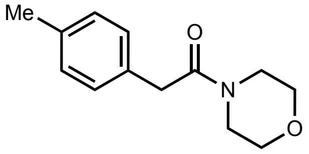

$C_{13}H_{17}NO_2$
MW: 219.28

Synthesised according to **GP-2** from 2-(4-methylphenyl)acetic acid (751 mg, 5.0 mmol), morpholine (0.47 mL, 5.5 mmol), EDC•HCl (1.15 g, 6.0 mmol), and DMAP (61 mg, 0.5 mmol) in CH_2Cl_2 (10 mL). The pure product was afforded without further purification as a white solid (1.08 g, 4.94 mmol, 98%).

^{1}H NMR (400 MHz, $CDCl_3$): δ 7.15 – 7.09 (m, 4H, Ar *H*), 3.68 (s, 2H ArC*H*$_2$), 3.67 – 3.63 (m, 4H, 2 × C*H*$_2$), 3.50 – 3.44 (m, 2H, C*H*$_2$), 3.44 – 3.37 (m, 2H, C*H*$_2$), 2.32 (s, 3H, C*H*$_3$).

^{13}C{^{1}H} NMR (101 MHz, $CDCl_3$): δ 169.9 (*C*=O), 136.6 (Ar *C*), 131.8 (Ar *C*), 129.6 (Ar *C*H), 128.5 (Ar *C*H), 66.9 (*C*H$_2$), 66.6 (*C*H$_2$), 46.6 (*C*H$_2$), 42.2 (*C*H$_2$), 40.6 (Ar*C*H$_2$), 21.1 (*C*H$_3$).

HRMS: calcd. for $C_{13}H_{17}NO_2Na$ $[M+Na]^+$: 242.1151; found (ESI$^+$): 242.1159.

Characterization data are consistent with literature values.[4]

2-(4-Bromophenyl)-1-morpholinoethan-1-one

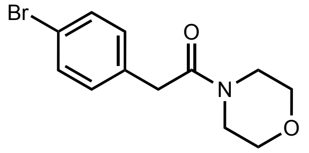

$C_{12}H_{14}BrNO_2$
MW: 284.15

Synthesised according to **GP-2** from 2-(4-bromophenyl)acetic acid (1.08 g, 5.0 mmol), morpholine (0.48 mL, 5.5 mmol), EDC•HCl (1.15 g, 6.0 mmol), and DMAP (61 mg, 0.5 mmol) in CH_2Cl_2 (20 mL). Purification by column chromatography (silica gel; 20-50% EtOAc in hexanes) afforded the product as a white solid (1.27 g, 4.5 mmol, 90%).

^{1}H NMR (500 MHz, $CDCl_3$): δ 7.48 – 7.42 (m, 2H, Ar *H*), 7.15 – 7.08 (m, 2H, Ar *H*), 3.68 – 3.60 (m, 6H, CH_2), 3.55 – 3.50 (m, 2H, CH_2), 3.45 – 3.40 (m, 2H, CH_2).

^{13}C{^{1}H} NMR (126 MHz, $CDCl_3$): δ 169.2 (*C*=O), 133.9 (Ar *C*), 132.0 (Ar *C*H), 130.5 (Ar *C*H), 121.0 (Ar *C*), 66.9 (*C*H$_2$), 66.6 (*C*H$_2$), 46.6 (*C*H$_2$), 42.3 (*C*H$_2$), 40.1 (Ar*C*H$_2$).

HRMS: calcd. for $C_{12}H_{15}BrNO_2$ $[M+H]^+$: 284.0281; found (ESI$^+$): 284.0288.

Characterization data are consistent with literature values.[3]

2-(4-Nitrophenyl)-1-morpholinoethan-1-one

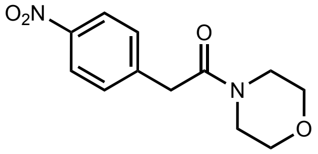

$C_{12}H_{14}N_2O_4$
MW: 250.25

Synthesised according to **GP-2** from 2-(4-nitrophenyl)acetic acid (0.91 g, 5.0 mmol), morpholine (0.48 mL, 5.5 mmol), EDC•HCl (1.15 g, 6.0 mmol), and DMAP (61 mg, 0.5 mmol) in CH_2Cl_2 (20 mL). Purification by column chromatography (silica gel; 20-50% EtOAc in hexanes) afforded the product as a white solid (1.13 g, 4.5 mmol, 90%).

^{1}H NMR (500 MHz, $CDCl_3$): δ 8.23 – 8.16 (m, 2H, Ar *H*), 7.45 – 7.38 (m, 2H, Ar *H*), 3.81 (s, 2H, CH_2), 3.71 – 3.64 (m, 4H, CH_2), 3.61 – 3.59 (m, 2H, CH_2), 3.50 – 3.45 (m, 2H, CH_2).

^{13}C{^{1}H} NMR (126 MHz, $CDCl_3$): δ 168.3 (*C*=O), 147.2 (Ar *C*), 142.5 (Ar *C*), 130.1 (Ar *C*H), 124.0 (Ar *C*H), 66.9 (*C*H_2), 66.6 (*C*H_2), 46.5 (*C*H_2), 42.4 (*C*H_2), 40.2 (Ar*C*H_2).

HRMS: calcd. for $C_{12}H_{15}N_2O_4$ [M+H]+: 251.1026; found (ESI+): 251.1029.

Characterization data are consistent with literature values.[3]

1-Morpholino-2-(naphthalen-2-yl)ethan-1-one

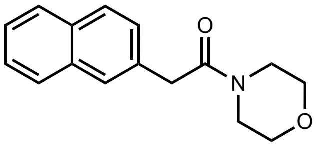

$C_{16}H_{17}NO_2$

MW: 255.32

Synthesised according to **GP-2** from 2-(2-naphthyl)acetic acid (931 mg, 5.0 mmol), morpholine (0.47 mL, 5.5 mmol), EDC•HCl (1.15 g, 6.0 mmol), and DMAP (61 mg, 0.5 mmol) in CH_2Cl_2 (10 mL). Purification by column chromatography (silica gel; 50% EtOAc in hexanes) afforded the product as a white solid (888 mg, 3.48 mmol, 70%).

^{1}H NMR (400 MHz, $CDCl_3$): δ 7.84 – 7.76 (m, 3H, Ar *H*), 7.68 (d, *J* = 1.8 Hz, 1H, Ar *H*), 7.50 – 7.43 (m, 2H, Ar *H*), 7.38 (dd, *J* = 8.5, 1.9 Hz, 1H, Ar *H*), 3.90 (s, 2H, ArCH_2), 3.70 – 3.63 (m, 4H, 2 x CH_2), 3.46 (s, 4H, 2 x CH_2).

^{13}C{^{1}H} NMR (101 MHz, $CDCl_3$): δ 169.7 (*C*=O), 133.7 (Ar *C*), 132.5 (Ar *C*), 132.4 (Ar *C*), 128.7 (Ar *C*H), 127.8 (Ar *C*H), 127.7 (Ar *C*H), 127.1 (Ar *C*H), 126.8 (Ar *C*H), 126.4 (Ar *C*H), 126.0 (Ar *C*H), 66.9 (CH_2), 66.6 (CH_2), 46.7 (CH_2), 42.3 (CH_2), 41.2 (ArCH_2).

HRMS: calcd. for $C_{16}H_{17}NO_2Na$ $[M+H]^+$: 278.1152; found (ESI$^+$): 278.1143.

Characterization data are consistent with literature values.[5]

4-(2-Morpholino-2-oxoethyl)phenyl acetate

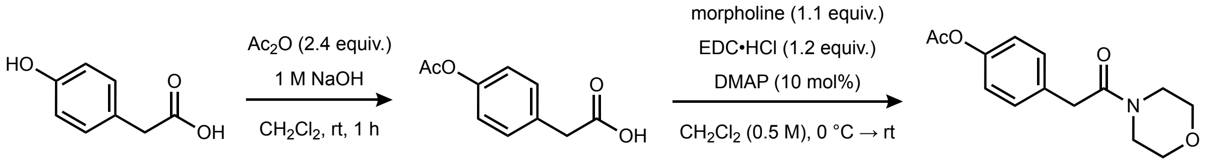

$C_{14}H_{17}NO_4$
MW: 263.29

Step 1: According to the literature procedure,[6] 4-hydroxyphenylacetic acid (761 mg, 5.0 mmol) was dissolved in a biphasic mixture of 1 M aqueous NaOH (15 mL) and CH_2Cl_2 (15 mL). Ac_2O (1.1 mL, 12.0 mmol) was added drop-wise and the reaction mixture stirred vigorously for 1 h. 2 M HCl (*ca.* 15 mL) was then added until pH 2-3 was achieved. The organic layer was separated and the aqueous layer extracted with CH_2Cl_2 (3 × 10 mL) and 5% MeOH in EtOAc (2 × 10 mL). The combined organics were dried over $MgSO_4$, filtered, and concentrated *in vacuo*. After drying under high vacuum for 1 h, the resulting white solid was used directly in the next step.

Step 2: Synthesised according to **GP-2** from 4-(acetoxy)phenylacetic acid (971 mg, 5.0 mmol), morpholine (0.47 mL, 5.5 mmol), EDC•HCl (1.15 g, 6.0 mmol), and DMAP (61 mg, 0.5 mmol) in CH_2Cl_2 (10 mL). Purification by column chromatography (silica gel; 70-100% EtOAc in hexane) afforded the product as a white solid (683 mg, 2.60 mmol, 52% over 2 steps).

^{1}H NMR (400 MHz, $CDCl_3$): δ 7.27 – 7.23 (m, 2H, Ar *H*), 7.08 – 7.03 (m, 2H, Ar *H*), 3.71 (s, 2H, Ar C*H₂*), 3.68 – 3.61 (m, 4H, 2 × C*H₂*), 3.56 – 3.49 (m, 2H, C*H₂*), 3.49 – 3.41 (m, 2H, C*H₂*), 2.29 (s, 3H, C*H₃*).

^{13}C{^{1}H} NMR (101 MHz, $CDCl_3$): δ 169.6 (*C*=O), 169.5 (*C*=O), 149.7 (Ar *C*), 132.4 (Ar *C*), 129.7 (Ar *C*H), 122.0 (Ar *C*H), 66.9 (*C*H_2), 66.6 (*C*H_2), 46.6 (*C*H_2), 42.3 (*C*H_2), 40.1 (Ar*C*H_2), 21.3 (*C*H_3).

HRMS: calcd. for $C_{14}H_{17}NO_4Na$ $[M+Na]^+$: 286.1050; found (ESI^+): 286.1040.

m.p.: 85-86 °C.

2-(4-(2-Morpholino-2-oxoethyl)phenyl)isoindoline-1,3-dione

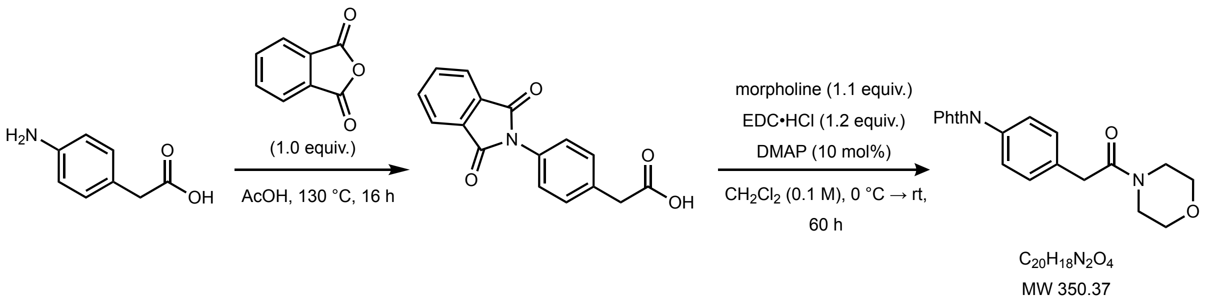

Step 1: To a suspension of 4-aminophenylacetic acid (1.75 g, 5.0 mmol) in AcOH (12.5 mL) was added phthalic anhydride (741 mg, 5.0 mmol) in one portion. The suspension was heated to reflux and stirred overnight. After cooling to rt, the resulting precipitate was isolated by filtration and the filter cake washed with cold EtOH (5 mL) and dried under reduced pressure to afford the pure product which was used directly in the next step.

^{1}H NMR (400 MHz, $CDCl_3$): δ 7.96 (dd, *J* = 5.5, 3.1 Hz, 2H, Ar *H*), 7.80 (dd, *J* = 5.5, 3.1 Hz, 2H, Ar *H*), 7.53 – 7.37 (m, 4H, Ar *H*), 3.72 (s, 2H, ArC*H*$_2$).

Step 2: Synthesised according to a modified **GP-2** from morpholine (0.47 mL, 5.5 mmol), EDC•HCl (1.15 g, 6.0 mmol), and DMAP (61 mg, 0.5 mmol) in CH_2Cl_2 (50 mL) for 60 h to afford the pure product without any further purification (1.39 g, 3.95 mmol, 79%). *Note: starting acid is poorly soluble in CH_2Cl_2 but dissolves over the course of the reaction.*

^{1}H NMR (400 MHz, $CDCl_3$): δ 7.95 (dd, *J* = 5.5, 3.1 Hz, 2H), 7.79 (dd, *J* = 5.5, 3.1 Hz, 2H), 7.43 (d, *J* = 8.6 Hz, 2H), 7.39 (d, *J* = 8.6 Hz, 2H), 3.78 (s, 2H), 3.70 – 3.63 (m, 4H), 3.59 – 3.53 (m, 2H), 3.50 – 3.44 (m, 2H).

^{13}C NMR (101 MHz, $CDCl_3$): δ 169.3 (*C*=O), 167.3 (*C*=O), 134.8 (Ar *C*), 134.6 (Ar *C*H), 131.9 (Ar *C*), 130.7 (Ar *C*), 129.4 (Ar *C*H), 126.9 (Ar *C*H), 123.9 (Ar *C*H), 67.0 (*C*H$_2$), 66.6 (*C*H$_2$), 46.7 (*C*H$_2$), 42.3 (*C*H$_2$), 40.5 (Ar*C*H$_2$).

HRMS: calcd. for $C_{20}H_{18}N_2O_4Na$ $[M+Na]^+$: 373.1159; found (ESI$^+$): 373.1150.

m.p.: 224-225 °C.

1-Morpholino-3-phenylpropan-1-one

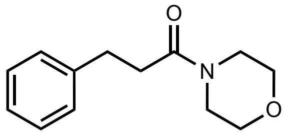

$C_{13}H_{17}NO_2$
MW: 219.28

Synthesised according to **GP-1** from 3-phenylpropionyl chloride (0.74 mL, 5.0 mmol), morpholine (0.86 mL, 10.0 mmol), and Et_3N (0.84 mL, 6.0 mmol) in CH_2Cl_2 (20 mL). Purification by column chromatography (silica gel; 20-50% EtOAc in hexanes) afforded the product as a colourless oil (1.02 g, 4.63 mmol, 93%).

^{1}H NMR (400 MHz, $CDCl_3$): δ 7.32 – 7.26 (m, 2H, Ar *H*), 7.24 – 7.18 (m, 3H, Ar *H*), 3.64 – 3.58 (m, 4H, 2 x CH_2), 3.52 – 3.48 (m, 2H, CH_2), 3.37 – 3.33 (m, 2H, CH_2), 3.00 – 2.95 (m, 2H, CH_2), 2.64 – 2.58 (m, 2H, CH_2).

^{13}C{^{1}H} NMR (101 MHz, $CDCl_3$): δ 171.0 (*C*=O), 141.1 (Ar *C*), 128.7 (Ar *C*H), 128.6 (Ar *C*H), 126.4 (Ar *C*H), 67.0 (*C*H_2), 66.6 (*C*H_2), 46.0 (*C*H_2), 42.0 (*C*H_2), 34.9 (*C*H_2), 31.6 (*C*H_2).

HRMS: calcd. for $C_{13}H_{18}FNO_2$ $[M+H]^+$: 220.1332; found (ESI$^+$): 220.1336.

Characterization data are consistent with literature values.[7]

1-Morpholino-2-cyclohexylethan-1-one

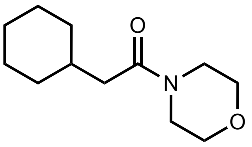

$C_{12}H_{21}NO_2$
MW: 211.31

Synthesised according to **GP-2** from 2-cyclohexylacetic acid (711 mg, 5.0 mmol), morpholine (0.47 mL, 5.5 mmol), EDC•HCl (1.15 g, 6.0 mmol), and DMAP (61 mg, 0.5 mmol) in CH_2Cl_2 (10 mL). The pure product was afforded without further purification as a white solid (990 mg, 4.69 mmol, 94%).

^{1}H NMR (400 MHz, $CDCl_3$): δ 3.70 – 3.57 (m, 6H, 3 × CH_2), 3.50 – 3.43 (m, 2H, CH_2), 2.19 (d, J = 6.7 Hz, 2H, CH_2Cy), 1.85 – 1.59 (m, 6H, CH, 2 × CH_2, CH_{2A}), 1.35 – 1.20 (m, 2H, CH_2), 1.20 – 1.05 (m, 1H, CH_{2B}), 1.03 – 0.87 (m, 2H, CH_2).

^{13}C{^{1}H} NMR (101 MHz, $CDCl_3$): δ 171.3 (*C*=O), 67.2 (*C*H_2), 66.9 (*C*H_2), 46.5 (*C*H_2), 42.0 (*C*H_2), 40.6 (*C*H_2Cy), 35.2 (*C*H), 33.5 (*C*H_2), 26.34 (*C*H_2), 26.26 (*C*H_2).

HRMS: calcd. for $C_{12}H_{15}FNO_2$ $[M+H]^+$: 212.1645; found (ESI$^+$): 212.1652.

ν_{max} (neat/cm^{-1}): 846, 1028, 1107, 1229, 1422, 1628, 2847, 2916.

m.p.: 71-72 °C.

1-Morpholinopent-4-en-1-one

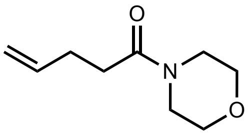

$C_9H_{11}NO_2$
MW: 169.22

Synthesised according to **GP-2** from 4-pentenoic acid (0.51 mL, 5.0 mmol), morpholine (0.47 mL, 5.5 mmol), EDC•HCl (1.15 g, 6.0 mmol), and DMAP (61 mg, 0.5 mmol) in CH_2Cl_2 (10 mL). The pure product was afforded without further purification as a colourless oil (732 mg, 4.32 mmol, 86%).

^{1}H NMR (400 MHz, $CDCl_3$): δ 5.92 – 5.80 (m, 1H, C*H*), 5.06 (dd, *J* = 17.0, 1.1 Hz, 1H, CH=CH_{trans}), 5.00 (dd, *J* = 10.1, 1.7 Hz, 1H, CH=CH_{cis}), 3.69 – 3.64 (m, 4H, 2 x CH_2), 3.64 – 3.60 (m, 2H, CH_2), 3.49 – 3.43 (m, 2H, CH_2), 2.41 – 2.38 (m, 4H, 2 x CH_2).

^{13}C{^{1}H} NMR (101 MHz, $CDCl_3$): δ 171.0 (*C*=O), 137.4 (H_2C=*C*H), 115.4 (H_2*C*=CH), 67.0 (*C*H_2), 66.7 (*C*H_2), 46.0 (*C*H_2), 42.0 (*C*H_2), 32.4 (*C*H_2), 29.2 (*C*H_2).

HRMS: calcd. for $C_9H_{11}NO_2Na$ $[M+Na]^+$: 192.0995; found (ESI^+): 192.1001.

Characterization data are consistent with literature values.[8]

1-Morpholinohept-6-en-1-one

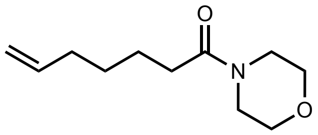

$C_{11}H_{19}NO_2$
MW: 197.28

Synthesised according to **GP-2** from 6-heptenoic acid (0.68 mL, 5.0 mmol), morpholine (0.47 mL, 5.5 mmol), EDC•HCl (1.15 g, 6.0 mmol), and DMAP (61 mg, 0.5 mmol) in CH_2Cl_2 (10 mL). Purification by column chromatography (silica gel; 50% EtOAc in hexanes) afforded the product as a colourless oil (861 mg, 4.38 mmol, 87%).

^{1}H NMR (400 MHz, CDCl$_3$): δ 5.80 (ddt, J = 17.0, 10.2, 6.7 Hz, 1H, CH=CH$_2$), 5.00 (app dq, J = 17.0, 1.7 Hz, 1H, CH=CH_{trans}), 4.95 (ddt, J = 10.2, 2.3, 1.2 Hz, 1H, CH=CH_{cis}), 3.69 – 3.63 (m, 4H, 2 × CH_2), 3.63 – 3.58 (m, 2H, CH_2), 3.48 – 3.43 (m, 2H, CH_2), 2.34 – 2.27 (m, 2H, CH_2), 2.12 – 2.04 (m, 2H, CH_2), 1.70 – 1.60 (m, 2H, CH_2), 1.50 – 1.39 (m, 2H, CH_2).

^{13}C{^{1}H} NMR (101 MHz, CDCl$_3$): δ 171.7 (C=O), 138.5 (H$_2$C=CH), 114.7 (H$_2$$C$=CH), 67.0 ($CH_2$), 66.7, ($CH_2$), 46.1, ($CH_2$), 41.9, ($CH_2$), 33.5, ($CH_2$), 32.7, ($CH_2$), 28.7, ($CH_2$), 24.7, ($CH_2$).

HRMS: calcd. for $C_{11}H_{19}NO_2Na$ $[M+Na]^+$: 220.1308; found (ESI$^+$): 220.1316.

Characterization data are consistent with literature values.[9]

6-Chloro-1-morpholinohexan-1-one

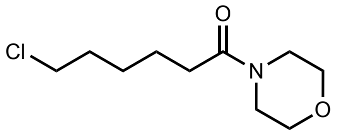

$C_{10}H_{18}ClNO_2$
MW: 219.71

Synthesised according to **GP-2** from 6-chlorohexanoic acid (753 mg, 5.0 mmol), morpholine (0.47 mL, 5.5 mmol), EDC•HCl (1.15 g, 6.0 mmol), and DMAP (61 mg, 0.5 mmol) in CH_2Cl_2 (10 mL). The pure product was afforded without further purification as a colourless oil (798 mg, 3.63 mmol, 73%).

^{1}H NMR (400 MHz, $CDCl_3$): δ 3.68 – 3.64 (m, 4H, 2 × CH_2), 3.63 – 3.58 (m, 2H, CH_2), 3.54 (t, J = 6.6 Hz, 2H, CH_2Cl), 3.45 (t, J = 4.8 Hz, 2H, CH_2), 2.32 (t, J = 7.5 Hz, 2H, CH_2(CO)), 1.80 (app dq, J = 8.0, 6.7 Hz, 2H, CH_2), 1.73 – 1.60 (m, 2H, CH_2), 1.55 – 1.43 (m, 2H, CH_2).

^{13}C NMR (101 MHz, $CDCl_3$): δ 171.5 (*C*=O), 67.0 (*C*H_2), 66.8 (*C*H_2), 46.0 (*C*H_2), 45.0 (*C*H_2Cl), 42.0 (*C*H_2), 32.9 (*C*H_2(CO)), 32.5 (*C*H_2), 26.8 (*C*H_2), 24.5 (*C*H_2).

HRMS: calcd. for $C_{10}H_{18}ClNO_2Na$ $[M+Na]^+$: 242.0918; found (ESI$^+$): 242.0910.

Methyl 8-morpholino-8-oxooctanoate

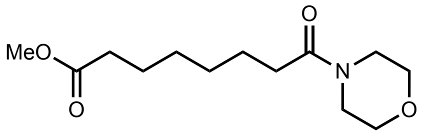

$C_{13}H_{23}NO_2$

MW: 257.33

Synthesised according to **GP-2** from 8-methoxy-8-oxooctanoic acid (941 mg, 5.0 mmol), morpholine (0.47 mL, 5.5 mmol), EDC•HCl (1.15 g, 6.0 mmol), and DMAP (61 mg, 0.5 mmol) in CH_2Cl_2 (10 mL). The pure product was afforded without further purification as a white solid (1.13 g, 4.39 mmol, 88%).

^{1}H NMR (400 MHz, $CDCl_3$): δ 3.67 – 3.62 (m, 7H, 2 × CH_2, CH_3), 3.62 – 3.56 (m, 2H, CH_2), 3.44 (t, J = 4.8 Hz, 2H, CH_2), 2.29 (t, J = 7.5 Hz, 4H, 2 × CH_2), 1.67 – 1.55 (m, 4H, 2 × CH_2), 1.38 – 1.28 (m, 4H, 2 × CH_2).

^{13}C{^{1}H} NMR (101 MHz, $CDCl_3$): δ 174.3 (*C*=O), 171.8 (*C*=O), 67.1 (CH_2), 66.8 (CH_2), 51.6 (CH_3), 46.1 (CH_2), 42.0 (CH_2), 34.1 (CH_2(CO)), 33.1 (CH_2(CO)), 29.2 (CH_2), 29.0 (CH_2), 25.1 (CH_2), 24.9 (CH_2).

HRMS: calcd. for $C_{13}H_{23}NO_4Na$ $[M+Na]^+$: 280.1519; found (ESI$^+$): 280.1511.

m.p.: 31-32 °C.

_N,N_-Dimethyl-2-phenylacetamide

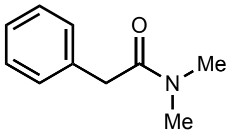

$C_{10}H_{13}NO$

MW: 163.22

Synthesised according to **GP-1** from 2-phenylacetyl chloride (0.68 mL, 5.0 mmol), dimethylamine hydrochloride (820 mg, 10.0 mmol), and Et_3N (0.84 mL, 6.0 mmol) in CH_2Cl_2 (20 mL). Purification by column chromatography (silica gel; 20-50% EtOAc in hexanes) afforded the product as a white solid (500 mg, 3.08 mmol, 62%).

^{1}H NMR (400 MHz, $CDCl_3$): δ 7.35 – 7.30 (m, 2H, Ar *H*), 7.28 – 7.22 (m, 3H, Ar *H*), 3.73 (s, 2H, PhCH_2), 3.00 (s, 3H, CH_3), 2.97 (s, 3H, CH_3).

^{13}C{^{1}H} NMR (101 MHz, $CDCl_3$): δ 171.2 (*C*=O), 135.1 (Ar *C*), 128.8 (Ar *C*H), 128.7 (Ar *C*H), 126.8 (Ar *C*H), 41.1 (Ph*C*H_2), 37.9 (*C*H_3), 35.7 (*C*H_3).

HRMS: calcd. for $C_{10}H_{14}NO$ $[M+H]^+$: 164.1070; found (ESI$^+$): 164.1071.

Characterization data are consistent with literature values.[10]

2-Phenyl-1-(piperidn-1-yl)ethan-1-one

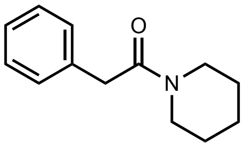

$C_{13}H_{17}NO$
MW: 203.29

Synthesised according to **GP-1** from 2-phenylacetyl chloride (0.68 mL, 5.0 mmol), piperidine (0.99 mL, 10.0 mmol), and Et_3N (0.84 mL, 6.0 mmol) in CH_2Cl_2 (20 mL). Purification by column chromatography (silica gel; 50-70% EtOAc in hexanes) afforded the product as a white solid (1.00 g, 4.94 mmol, 99%).

^{1}H NMR (400 MHz, $CDCl_3$): δ 7.35 – 7.29 (m, 2H, Ar *H*), 7.28 – 7.21 (m, 3H, Ar *H*), 3.74 (s, 2H, PhC*H*$_2$), 3.61 – 3.56 (m, 2H, C*H*$_2$), 3.40 – 3.35 (m, 2H, C*H*$_2$), 1.62 – 1.49 (m, 4H, 2 x C*H*$_2$), 1.39 – 1.31 (m, 2H, C*H*$_2$).

^{13}C{^{1}H} NMR (101 MHz, $CDCl_3$): δ 169.3 (*C*=O), 135.5 (Ar *C*), 128.7 (Ar *C*H), 128.7 (Ar *C*H), 126.7 (Ar *C*H), 47.3 (*C*H$_2$), 42.9 (*C*H$_2$), 41.3 (Ph*C*H$_2$), 26.3 (*C*H$_2$), 25.6 (*C*H$_2$), 24.5 (*C*H$_2$).

HRMS: calcd. for $C_{13}H_{18}NO$ $[M+H]^+$: 204.1388; found (ESI$^+$): 204.1382.

Characterization data are consistent with literature values.[11]

2-Phenyl-1-(pyrrolidin-1-yl)ethan-1-one

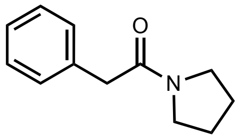

$C_{12}H_{15}NO$
MW: 189.26

Synthesised according to **GP-1** from 2-phenylacetyl chloride (0.68 mL, 5.0 mmol), pyrrolidine (0.82 mL, 10.0 mmol), and Et_3N (0.84 mL, 6.0 mmol) in CH_2Cl_2 (20 mL). Purification by column chromatography (silica gel; 50-70% EtOAc in hexanes) afforded the product as a white solid (902 mg, 4.77 mmol, 95%).

^{1}H NMR (400 MHz, $CDCl_3$): δ 7.34 – 7.21 (m, 5H, Ar *H*), 3.66 (s, 2H, PhCH_2), 3.49 (t, *J* = 6.7 Hz, 2H, CH_2), 3.42 (t, *J* = 6.7 Hz, 2H, CH_2), 1.95 – 1.79 (m, 4H, 2 x CH_2).

^{13}C{^{1}H} NMR (101 MHz, $CDCl_3$): δ 169.7 (*C*=O), 135.1 (Ar *C*), 129.1 (Ar *C*H), 128.7 (Ar *C*H), 126.8 (Ar *C*H), 47.0 (*C*H_2), 46.1 (*C*H_2), 42.5 (Ph*C*H_2), 26.3 (*C*H_2), 24.5 (*C*H_2).

HRMS: calcd. for $C_{12}H_{16}NO$ $[M+H]^+$: 190.1232; found (ESI$^+$): 190.1229.

Characterization data are consistent with literature values.[11]

2-Phenyl-1-(4-tosylpiperazin-1-yl)ethan-1-one

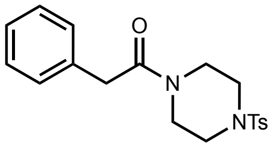

$C_{19}H_{22}N_2O_3S$
MW: 358.46

Synthesised according to **GP-1** from 2-phenylacetyl chloride (0.68 mL, 5.0 mmol), *N*-tosylpiperazine (2.40 g, 10.0 mmol), and Et_3N (0.84 mL, 6.0 mmol) in CH_2Cl_2 (30 mL). Purification by column chromatography (silica gel; 0-5% MeOH in CH_2Cl_2) afforded the product as a white solid (840 mg, 2.34 mmol, 47%).

^{1}H NMR (400 MHz, $CDCl_3$): δ 7.57 – 7.53 (m, 2H, Ar *H*), 7.32 (d, *J* = 7.9 Hz, 2H, Ar *H*), 7.25 – 7.18 (m, 3H, Ar *H*), 7.13 (dd, *J* = 7.9, 1.7 Hz, 2H, Ar *H*), 3.70 (t, *J* = 5.2 Hz, 2H, NCH_2), 3.66 (s, 2H, PhCH_2), 3.49 (t, *J* = 5.1 Hz, 2H, NCH_2), 2.93 (t, *J* = 5.2 Hz, 2H, NCH_2), 2.72 (t, *J* = 5.1 Hz, 2H, NCH_2), 2.45 (s, 3H, CH_3).

^{13}C{^{1}H} NMR (101 MHz, $CDCl_3$): δ 169.6 (*C*=O), 144.2 (Ar *C*), 134.5 (Ar *C*), 132.2 (Ar *C*), 129.9 (Ar *C*H), 128.9 (Ar *C*H), 128.6 (Ar *C*H), 127.9 (Ar *C*H), 127.1 (Ar *C*H), 45.9 (*C*H_2), 45.8 (*C*H_2), 45.6 (*C*H_2), 41.2 (*C*H_2), 41.1 (*C*H_2), 21.7 (*C*H_3).

HRMS: calcd. for $C_{19}H_{22}N_2O_3SNa$ $[M+H]^+$: 381.1243; found (ESI$^+$): 381.1260.

ν_{max} (neat/cm^{-1}): 696, 730, 947, 1111, 1159, 1233, 1342, 1412, 1655, 2847.

m.p.: 173-175 °C.

***N,N*-Diisopropyl-2-phenylacetamide**

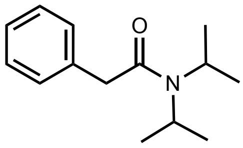

$C_{14}H_{21}NO$
MW: 219.33

Synthesised according to **GP-1** from 2-phenylacetyl chloride (0.68 mL, 5.0 mmol), diisopropylamine (1.4 mL, 10.0 mmol), and Et_3N (0.84 mL, 6.0 mmol) in CH_2Cl_2 (20 mL). Purification by column chromatography (silica gel; 50% EtOAc in hexanes) afforded the product as a white solid (780 mg, 3.54 mmol, 71%).

^{1}H NMR (400 MHz, $CDCl_3$): δ 7.34 – 7.21 (m, 5H, Ar *H*), 4.00 – 3.92 (m, 1H, C*H*), 3.69 (s, 2H, PhCH_2), 3.42 – 3.31 (m, 1H, C*H*), 1.41 (d, *J* = 6.8 Hz, 6H, 2 x CH_3), 1.00 (d, *J* = 6.8 Hz, 6H, 2 x CH_3).

^{13}C{^{1}H} NMR (101 MHz, $CDCl_3$): δ 170.0 (*C*=O), 135.8 (Ar *C*), 128.6 (Ar *C*H), 128.5 (Ar *C*H), 126.5 (Ar *C*H), 49.5 (*C*H), 45.9 (*C*H), 43.5 (Ph*C*H_2), 20.6 (2 × *C*H_3), 20.5 (2 × *C*H_3).

HRMS: calcd. for $C_{14}H_{21}NONa$ $[M+Na]^+$: 242.1515; found (ESI$^+$): 242.1509.

Characterization data are consistent with literature values.[10]

***N*-Methoxy-*N*-methyl-2-phenylacetamide**

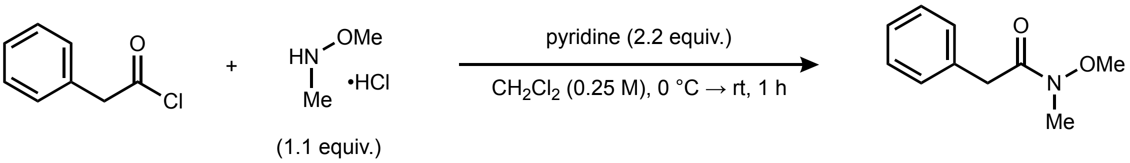

$C_{10}H_{13}NO_2$
MW: 179.22

According to the literature procedure,[12] phenylacetyl chloride (0.66 mL, 5.0 mmol) was added to a suspension of *N,O*-dimethylhydroxylamine hydrochloride (585 mg, 6.0 mmol) in CH_2Cl_2 (20 mL) cooled to 0 °C (ice/water bath). Pyridine (0.89 mL, 11.0 mmol) was then added drop-wise, and the resulting solution was stirred at 0 °C for 5 min then warmed to rt and stirred for 1 h. The reaction was quenched by the addition of 1 M HCl (10 mL) and the CH_2Cl_2 layer was separated. The aqueous layer was extracted with CH_2Cl_2 (3 × 10 mL) and the combined organics were washed with saturated aqueous $NaHCO_3$ (50 mL), dried over Na_2SO_4, and concentrated *in vacuo*. The pure product was afforded without further purification as a colourless oil (650 mg, 3.63 mmol, 73%).

^{1}H NMR (400 MHz, $CDCl_3$): δ 7.34 – 7.27 (m, 4H, Ar*H*), 7.27 – 7.21 (m, 1H, Ar*H*), 3.78 (s, 2H, PhCH_2), 3.60 (s, 3H, OCH_3), 3.19 (s, 3H, NCH_3).

^{13}C{^{1}H} NMR (101 MHz, $CDCl_3$): δ 172.5 (*C*=O), 135.0 (Ar *C*), 129.4 (Ar *C*H), 128.6 (Ar *C*H), 126.8 (Ar *C*H), 61.3 (O*C*H_3), 39.5 (Ph*C*H_2), 32.3 (N*C*H_3).

HRMS: calcd. for $C_{10}H_{13}NO_2Na$ $[M+Na]^+$: 202.0838; found (ESI+): 202.0845.

Characterization data are consistent with literature values.[12]

***N*-Methyl-2-phenyl-*N*-(3-phenyl-3-(4-(trifluoromethyl)phenoxy)propyl)acetamide**

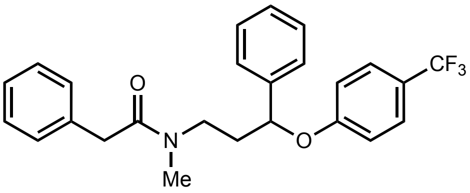

$C_{25}H_{24}F_3NO_2$
MW: 427.47

Synthesised according to **GP-3** from fluoxetine hydrochloride (1.73 g, 5.0 mmol), DIPEA (1.31 mL, 7.5 mmol), and phenylacetyl chloride (0.99 mL, 7.5 mmol) in CH_2Cl_2 (50 mL). Purification by column chromatography (silica gel; 20-40% EtOAc) in hexanes) afforded the product as a colourless viscous oil (1.94 g, 4.53 mmol, 91%).

A 1.2:1 mixture of rotamers was observed at 298 K in $CDCl_3$.

^{1}H NMR (400 MHz, $CDCl_3$): δ 7.45 (d, *J* = 8.8 Hz, 0.8H, Ar *H*), 7.41 (d, *J* = 8.8 Hz, 1.2H, Ar *H*), 7.39 – 7.19 (m, 9H, Ar *H*), 7.11 (dd, *J* = 6.9, 1.8 Hz, 0.8H, Ar *H*), 6.87 (d, *J* = 8.5 Hz, 0.8H, Ar *H*), 6.83 (d, *J* = 8.7 Hz, 1.2H, Ar *H*), 5.13 (dd, *J* = 8.7, 4.3 Hz, 0.6H, OC*H*Ph), 5.10 (dd, *J* = 8.7, 3.8 Hz, 0.4H, OC*H*Ph), 3.70 – 3.51 (m, 3.5H, CH_2, C$H_{2A,B}$), 3.44 (ddd, *J* = 14.4, 9.3, 4.4 Hz, 0.5H, CH_{2B}), 2.96 (s, 1.6H, NCH_3), 2.95 (s, 1.4H, NCH_3), 2.24 – 2.07 (m, 1.6H, CH_2), 2.04 – 1.98 (m, 0.4H, CH_2).

^{13}C{^{1}H} NMR (101 MHz, $CDCl_3$): δ 171.23 (*C*=O), 171.15 (*C*=O), 160.4 (Ar *C*), 160.1 (Ar *C*), 140.8 (Ar *C*), 140.0 (Ar *C*), 135.2 (Ar *C*), 135.0 (Ar *C*), 129.2 (Ar *C*H), 128.93 (Ar *C*H), 128.88 (Ar *C*H), 128.82 (Ar *C*H), 128.79 (Ar *C*H), 128.4 (Ar *C*H), 128.1 (Ar *C*H), 127.1 (q, *J* = 3.8 Hz, Ar *C*H), 126.9 (q, *J* = 4.0 Hz, Ar *C*H), 125.8 (Ar *C*H), 125.7 (Ar *C*H), 123.4 (q, *J* = 32.8 Hz, Ar *C*), 123.0 (q, *J* = 32.5 Hz, Ar *C*), 115.8 (Ar *C*H), 115.8 (Ar *C*H), 78.3 (O*C*HPh), 77.4 (O*C*HPh), 46.7 (*C*H_2), 45.6 (*C*H_2), 41.5 (*C*H_2), 40.9 (*C*H_2), 37.3 (*C*H_2), 36.6 (N*C*H_3), 36.3 (*C*H_2), 33.6 (N*C*H_3). *The quaternary carbon corresponding to the CF_3 was not observed, though its presence was confirmed by ^{19}F NMR spectroscopy.*

^{19}F NMR (471 MHz, $CDCl_3$): δ -61.58 (s, 1.7F), -61.65 (s, 1.3F).

HRMS: calcd. for $C_{25}H_{24}F_3NO_2Na$ $[M+Na]^+$: 450.1651; found (ESI$^+$): 450.1671.

ν_{max} (neat/cm^{-1}): 698, 835, 1066, 1107, 1158, 1244, 1322, 1453, 1516, 1613, 1639, 2930.

N-(3-(9,10-Ethanoanthracen-9(10H)-yl)propyl)-N-methyl-2-phenylacetamide

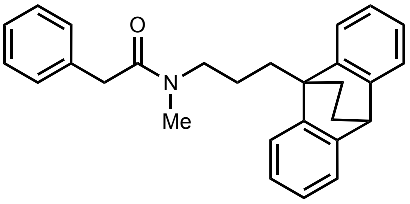

$C_{28}H_{29}NO$
MW: 395.55

Synthesised according to **GP-3** from maprotiline hydrochloride (629 mg, 2.0 mmol), DIPEA (0.52 mL, 3.0 mmol), and phenylacetyl chloride (0.40 mL, 3.0 mmol) in CH_2Cl_2 (20 mL). Purification by column chromatography (silica gel; 30% EtOAc) in hexanes) afforded the product as a white solid (683 mg, 1.72 mmol, 86%).

A 1.5:1 mixture of rotamers was observed at 298 K in $CDCl_3$.

^{1}H NMR (400 MHz, $CDCl_3$): δ 7.38 – 7.17 (m, 8H, Ar *H*), 7.14 – 7.04 (m, 5H, Ar *H*), 4.35 – 4.26 (m, 1H, C*H*), 3.87 (s, 0.8H, CH_2), 3.78 (s, 1.2H, CH_2), 3.69 (t, J = 7.4 Hz, 1.2H, NCH_2), 3.58 (t, J = 7.8 Hz, 0.8H, NCH_2), 3.08 (s, 1.7H, NCH_3), 3.06 (s, 1.3H, NCH_3), 2.48 – 2.39 (m, 1.2H, CH_2), 2.35 – 2.25 (m, 0.8H, CH_2), 2.07 – 1.96 (m, 1.2H, CH_2), 1.96 – 1.86 (m, 0.8H, CH_2), 1.82 (ddd, J = 10.4, 4.3, 2.8 Hz, 2H, HC-CH_2), 1.60 – 1.51 (m, 1.2H, HC-CH_2CH_2), 1.51 – 1.44 (m, 0.8H, HC-CH_2CH_2).

^{13}C NMR (101 MHz, $CDCl_3$): δ 171.2 (*C*=O), 171.0 (*C*=O), 145.4 (Ar *C*), 145.1 (Ar *C*), 145.0 (Ar *C*), 135.5 (Ar *C*), 135.2 (Ar *C*), 128.94 (Ar *C*H), 128.87 (Ar *C*H), 128.85 (Ar *C*H), 128.8 (Ar *C*H), 127.0 (Ar *C*H), 126.9 (Ar *C*H), 125.5 (Ar *C*H), 125.44 (Ar *C*H), 125.42 (Ar *C*H), 125.35 (Ar *C*H), 123.6 (Ar *C*H), 123.5 (Ar *C*H), 121.3 (Ar *C*H), 121.0 (Ar *C*H), 51.3 (N*C*H_2), 48.9 (N*C*H_2), 44.8 (*C*H), 44.62 (*C*H), 44.55 (*C*H), 41.6 (*C*H_3), 41.3 (*C*H_3), 36.1 (*C*H_3), 33.7 (*C*H_3), 29.7 (*C*H_2), 28.14 (*C*H_2), 28.08 (*C*H_2), 27.8 (*C*H_2), 27.6 (*C*H_2), 24.0 (*C*H_2), 22.9 (*C*H_2).

HRMS: $C_{25}H_{24}F_3NO_2Na$ $[M+Na]^+$: 418.2141; found (ESI^+): 418.2155.

ν_{max} (neat/cm^{-1}): 725, 749, 762, 1115, 1401, 1450, 1628, 2936.

m.p.: 100-102 °C

3.4 Synthesis of Sulfoxides

Diisopropylsulfoxide

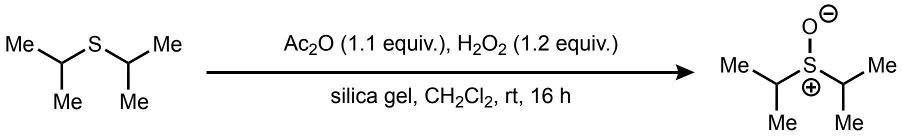

To a mixture of isopropylsulfide (0.73 mL, 5 mmol), Ac_2O (0.52 mL, 5.5 mmol), and silica gel (1.0 g, 230–400 mesh) in CH_2Cl_2 (25 mL) was added H_2O_2 (50 wt% in water, 0.34 mL, 6.0 mmol) in one portion. The reaction mixture was stirred for 16 h then filtered and washed with saturated aqueous $NaHSO_3$ (50 mL), saturated aqueous $NaHCO_3$ (50 mL), and brine (50 mL). The organic layer was separated, dried over Na_2SO_4 and concentrated *in vacuo*. Purification by column chromatography (silica gel; EtOAc) afforded the product as a colourless liquid (288 mg, 2.15 mmol, 43%).

^{1}H NMR (400 MHz, $CDCl_3$): δ 2.76 (hept, J = 6.9 Hz, 2H, C*H*), 1.30 (d, J = 6.9 Hz, 6H, CH_3), 1.25 (d, J = 6.9 Hz, 6H, CH_3).

^{13}C{^{1}H} NMR (101 MHz, $CDCl_3$): δ 46.8 (*C*H), 17.4 (*C*H_3), 14.3 (*C*H_3).

HRMS: calcd. for $C_6H_{15}OS$ $[M+H]^+$: 135.0844; found (ESI^+): 135.0839.

Characterization data are consistent with literature values.[13]

3.5 Synthesis of Photocatalyst

10-Phenyl-10*H*-phenothiazine (PTH)

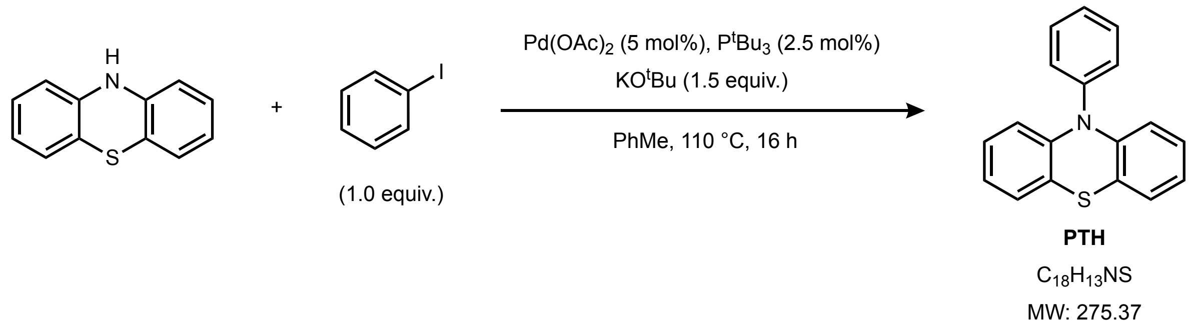

An oven-dried flask was charged with phenothiazine (1.99 g, 10.0 mmol), $Pd(OAc)_2$ (112 mg, 0.5 mmol, 5 mol%), tri-*tert*-butylphosphine (1 M in PhMe, 0.25 mL, 0.25 mmol), KO^tBu (1.68 g, 15.0 mmol), iodobenzene (1.1 mL, 10.0 mmol), and PhMe (90 mL). The flask was fitted with a condenser and the reaction mixture was heated to 110 °C and stirred overnight. After cooling to rt, the reaction mixture was diluted with CH_2Cl_2 and washed with water. The organics were dried over $MgSO_4$, filtered, and concentrated *in vacuo*. Purification by column chromatography (silica gel; 10% $CHCl_3$ in hexanes) afforded the product as a pale yellow solid (2.28 g, 8.30 mmol, 83%).

^{1}H NMR (400 MHz, $CDCl_3$): δ 7.63 – 7.57 (m, 2H, Ar *H*), 7.50 – 7.45 (m, 1H, Ar *H*), 7.42 – 7.37 (m, 2H, Ar *H*), 7.04 – 6.99 (m, 2H, Ar *H*), 6.88 – 6.77 (m, 4H, Ar *H*), 6.23 – 6.17 (m, 2H, Ar *H*).

^{13}C{^{1}H} NMR (101 MHz, $CDCl_3$): δ 144.4 (Ar *C*), 141.2 (Ar *C*), 131.0 (Ar *C*H), 130.9 (Ar *C*H), 128.3 (Ar *C*H), 127.0 (Ar *C*H), 126.9 (Ar *C*H), 122.6 (Ar *C*H), 120.3 (Ar *C*), 116.2 (Ar *C*H).

HRMS: calcd. for $C_{18}H_{13}NS$ $[M]^+$: 275.0769; found (ESI^+): 275.0763.

Characterization data are consistent with literature values.[14]

3.6 Synthesis of Silyl Enol Ethers

Silyl enol ethers were prepared according to the literature procedure.[15]

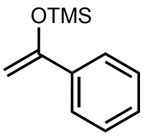

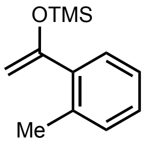

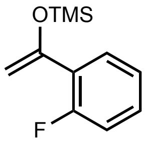

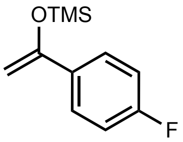

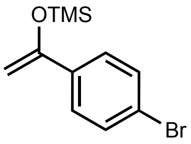

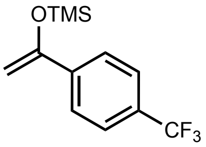

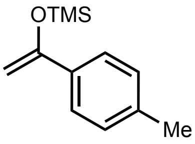

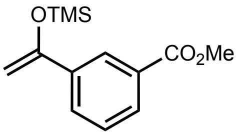

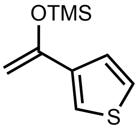

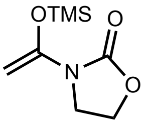

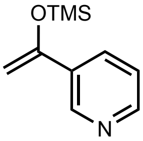

3.7 Synthesis of Alkenyl Trifluoroborates

Potassium (*E*)-(-4-fluorostyryl)trifluoroborate

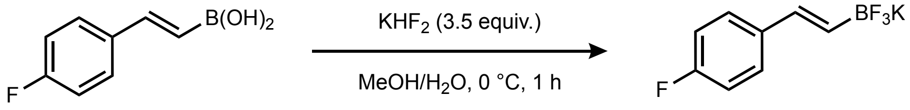

$C_8H_6BF_4K$

MW: 228.04

To a solution of 4-fluorostyrylboronic acid (249 mg, 1.5 mmol) in a minimal amount of MeOH cooled to 0 °C (ice/water bath) was added KHF_2 (4.5 M in H_2O, 1.2 mL, 5.25 mmol) drop-wise. The reaction mixture was stirred for 1 h and then frozen and lyophilized. The dried residue was then triturated with hot acetone and filtered. The resulting filtrate was concentrated and re-dissolved in a minimal amount of hot acetone. Et_2O was then added, and the resulting precipitate filtered and dried over a flow of air to afford the pure product as a white solid (237 mg, 1.04 mmol, 69%).

^{1}H NMR (400 MHz, acetone-d$_6$): 7.40 – 7.30 (m, 2H, Ar *H*), 7.03 – 6.93 (m, 2H, Ar *H*), 6.61 (d, *J* = 18.2 Hz, 1H, C*H*), 6.25 (dq, *J* = 18.2, 3.6 Hz, 1H, C*H*).

^{13}C NMR (101 MHz, acetone-d$_6$): 162.2 (d, *J* = 242.0 Hz, Ar *C*F), 138.2 (Ar *C*), 133.6 (q, *J* = 4.8 Hz, *C*H), 128.0 (d, *J* = 7.7 Hz, Ar *C*H), 115.5 (d, *J* = 21.4 Hz, Ar *C*H). *The carbon atom adjacent to boron was not observed due to quadrupolar relaxation.*

HRMS: calcd. for $C_8H_6BF_4$ $[M-K]^-$: 189.0504; found (ESI$^-$): 189.0510.

Characterization data are consistent with literature values.[16]

Potassium (*E*)-(4-methoxystyryl)trifluoroborate

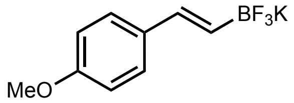

$C_9H_9BF_3OK$

MW: 240.07

Synthesised according to the above procedure from (*E*)-(4-methoxystyryl)boronic acid (267 mg, 1.5 mmol) and KHF_2 (4.5 M in H_2O, 1.2 mL, 5.25 mmol) to afford the pure product as a white solid (68 mg, 0.28 mmol, 19%).

^{1}H NMR (400 MHz, acetone-d$_6$): 7.30 – 7.21 (m, 2H, Ar *H*), 6.85 – 6.76 (m, 2H, Ar *H*), 6.58 (d, *J* = 18.2 Hz, 1H, C*H*), 6.16 (dq, *J* = 18.2, 3.6 Hz, 1H, C*H*), 3.75 (s, 3H, C*H*$_3$).

^{13}C NMR (101 MHz, acetone-d$_6$): 159.0 (Ar *C*O), 135.0 (Ar *C*), 133.6 (d, *J* = 4.7 Hz, *C*H), 127.4 (Ar *C*H), 114.3 (Ar *C*H), 55.4 (*C*H$_3$). *The carbon atom adjacent to boron was not observed due to quadrupolar relaxation.*

HRMS: calcd. for $C_9H_9OBF_4$ $[M-K]^-$: 201.0704; found (ESI$^-$): 201.0701.

Characterization data are consistent with literature values.[17]

Potassium (*E*)-(prop-1-en-1-yl)-trifluoroborate

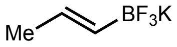

$C_3H_5BF_3K$

MW: 147.98

Synthesised according to the above procedure from (*E*)-(prop-1-enyl)boronic acid pinacol ester (252 mg, 1.5 mmol) and KHF_2 (4.5 M in H_2O, 1.2 mL, 5.25 mmol) to afford the pure product as a white solid (220 mg, 1.20 mmol, 80%).

^{1}H NMR (400 MHz, acetone-d$_6$): 5.58 (dd, *J* = 16.8, 6.4 Hz, 1H, C*H*), 5.31 (dqd, *J* = 17.4, 3.7, 1.9 Hz, 1H, C*H*), 1.53 – 1.48 (m, 3H, C*H*$_3$).

^{13}C NMR (101 MHz, acetone-d$_6$): 130.09 (*C*H), 21.49 (*C*H$_3$). *The carbon atom adjacent to boron was not observed due to quadrupolar relaxation.*

^{19}F NMR (376 MHz, acetone-d$_6$): -141.46 (s, B*F*$_3$).

^{11}B NMR (128 MHz, acetone-d$_6$): 3.74 – 1.48 (m, *B*F$_3$).

HRMS: calcd. for $C_3H_5BF_3$ [M-K]$^-$: 109.0442; found (ESI$^-$): 109.0449.

ν_{max} (neat/cm^{-1}): 591, 735, 911, 1103, 1280, 1644, 2996.

Potassium (*E*)-(3-methylstyryl)trifluoroborate

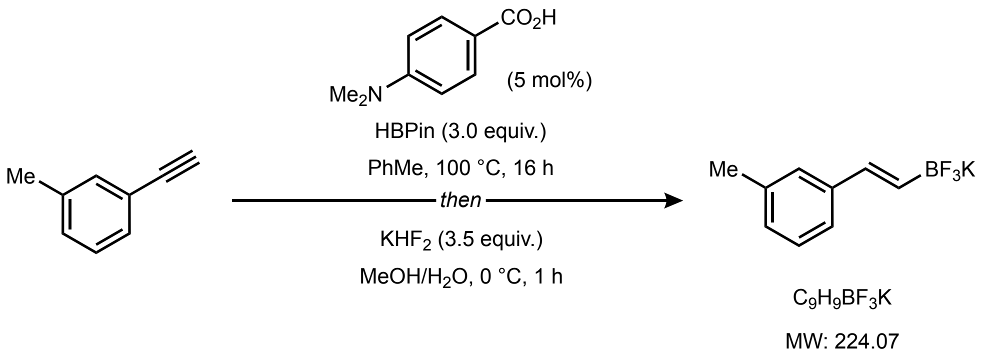

To a solution of 3-methylphenylacetylene (232 mg, 2.0 mmol) and 4-(dimethylamino)benzoic acid (16.5 mg, 0.1 mmol) in toluene (1.0 M, 2.0 mL) was added HBPin (768 mg, 6.0 mmol). The reaction mixture was heated to 100 °C and stirred overnight. After cooling to rt, the resulting solids were filtered and washed with heptane. The volatiles were removed *in vacuo* to afford the crude pinacol ester that was used without further purification. The boronic ester was dissolved in a minimal amount of MeOH and cooled to 0 °C (ice/water bath). KHF_2 (4.5 M in H_2O, 1.6 mL, 7.0 mmol) was added drop-wise and the reaction mixture was stirred for 1 h and then frozen and lyophilized. The dried residue was then triturated with hot acetone and filtered. The resulting filtrate was concentrated and re-dissolved in a minimal amount of hot acetone. Et_2O was then added, and the resulting precipitate filtered and dried over a flow of air to afford the pure product as a white solid (189 mg, 0.84 mmol 42%).

^{1}H NMR (400 MHz, acetone-d$_6$): 7.18 – 7.07 (m, 3H, Ar *H*), 6.92 (d, *J* = 6.9 Hz, 1H, Ar *H*), 6.64 (d, *J* = 18.4 Hz, 1H, C*H*), 6.30 (dq, J = 18.3, 3.8 Hz, 1H, C*H*), 2.27 (s, 3H, CH_3).

^{13}C NMR (101 MHz, acetone-d$_6$): 141.7 (Ar *C*), 138.1 (*C*H), 135.1 (q, *J* = 4.8 Hz, *C*H), 128.8 (Ar *C*H), 127.4 (Ar *C*H), 127.3 (Ar *C*H), 123.7 (Ar *C*H), 21.4 (CH_3).

^{19}F NMR (376 MHz, acetone-d$_6$): -141.39 (s, BF_3).

^{11}B NMR (128 MHz, acetone-d$_6$): 3.74 – 1.48 (m, *B*F_3).

HRMS: calcd. for $C_9H_9BF_3$ [M-K]$^-$: 185.0755; found (ESI$^-$): 185.0763.

ν_{max} (neat/cm^{-1}): 540, 730, 924, 1098, 1224, 1627, 2993.

Potassium (E)-(thiophen-3-yl)trifluoroborate

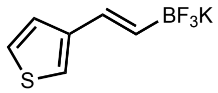

$C_6H_5BF_3SK$

MW: 216.07

Synthesised according to the above procedure from 3-ethynylthiophene (0.20 mL, 2.0 mmol), 4-(dimethylamino)benzoic acid (16.5 mg, 0.1 mmol) in toluene (1.0 M, 2.0 mL) followed by KHF_2 (4.5 M in H_2O, 1.2 mL, 5.25 mmol) to afford the pure product as a white solid (223 mg, 1.04 mmol, 52%).

^{1}H NMR (400 MHz, acetone-d$_6$): 7.28 (dd, *J* = 5.1, 2.9 Hz, 1H, thiophene *H*), 7.22 (dd, *J* = 5.1, 1.3 Hz, 1H, thiophene *H*), 7.02 (d, *J* = 2.9 Hz, 1H, thiophene *H*), 6.65 (d, *J* = 18.2 Hz, 1H, C*H*), 6.10 (dq, *J* = 18.2, 3.7 Hz, 1H, C*H*).

^{13}C NMR (101 MHz, acetone-d$_6$): 145.5 (thiophene *C*), 129.0 (q, *J* = 4.8 Hz, *C*H), 126.1 (thiophene *C*H), 125.6 (thiophene *C*H), 119.4 (thiophene *C*H). *The carbon atom adjacent to boron was not observed due to quadrupolar relaxation.*

HRMS: calcd. for $C_6H_5BF_3S$ $[M-K]^-$: 177.0163; found (ESI$^-$): 177.0172.

Characterization data are consistent with literature values.[18]

Potassium (*E*)-(2-bromostyryl)trifluoroborate

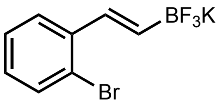

$C_8H_6BrBF_3K$

MW: 288.94

Synthesised according to the above procedure from 1-ethynyl-2-bromobenzene (362 mg, 2.0 mmol), 4-(dimethylamino)benzoic acid (16.5 mg, 0.1 mmol) in toluene (1.0 M, 2.0 mL) followed by KHF_2 (4.5 M in H_2O, 1.2 mL, 5.25 mmol) to afford the pure product as a white solid (156 mg, 0.54 mmol, 27%).

^{1}H NMR (400 MHz, acetone-d$_6$): 7.61 (dd, *J* = 7.9, 1.7 Hz, 1H, Ar *H*), 7.49 (dd, *J* = 8.0, 1.3 Hz, 1H, Ar *H*), 7.26 (ddt, *J* = 8.6, 7.9, 1.0 Hz, 1H, Ar *H*), 7.08 – 7.00 (m, 1H, Ar *H*), 6.99 (d, *J* = 17.8 Hz, 1H, C*H*), 6.34 (dq, *J* = 18.0, 3.6 Hz, 1H, C*H*).

^{13}C NMR (101 MHz, acetone-d$_6$): 141.1 (Ar *C*Br), 133.4 (Ar *C*H), 132.8 (q, *J* = 4.6 Hz, *C*H), 128.2 (Ar *C*H), 127.5 (Ar *C*H), 123.5 (Ar *C*), 119.6 (Ar *C*H).

^{19}F NMR (376 MHz, acetone-d$_6$): -141.83 (s, B*F*$_3$).

^{11}B NMR (128 MHz, acetone-d$_6$): 2.74 (m, *B*F$_3$).

HRMS: calcd. for $C_9H_9BF_3$ [M-K]$^-$: 248.9703; found (ESI$^-$): 248.9709.

ν_{max} (neat/cm^{-1}): 729, 944, 1239, 1289, 1434, 1465, 1622.

4.1 General Procedure 4 (GP-4): Synthesis of Amido Sulfonium Salts

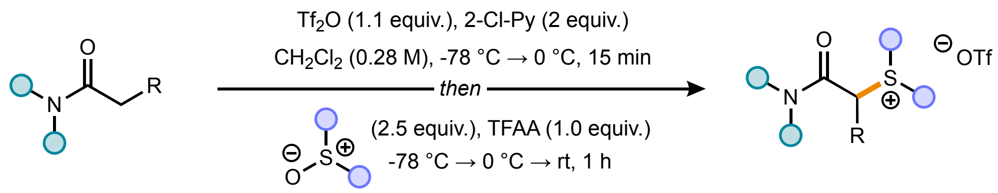

According to the modified literature procedure,[19] an oven- or flame-dried flask under an atmosphere of dinitrogen was charged with amide (1.0 mmol), 2-chloropyridine (0.19 mL, 2.0 mmol) and anhydrous CH_2Cl_2 (0.28 M, 3.5 mL). The solution was cooled to -78 °C (dry ice/acetone bath) and Tf_2O (0.18 mL, 1.1 mmol) was added drop-wise. After complete addition, the reaction was warmed to 0 °C (ice/water bath) and stirred for 15 min. After cooling once more to -78 °C, the sulfoxide (2.5 mmol) in anhydrous CH_2Cl_2 (0.5 mL) was added drop-wise, followed by drop-wise addition of TFAA (0.14 mL, 1.0 mmol). The reaction mixture was warmed to 0 °C and stirred for 15 min and at rt for 45 min. The reaction was quenched by addition of saturated aqueous $NaHCO_3$ (5 mL/mmol) followed by vigorous stirring for 5 min. The organic layer was separated and the aqueous layer extracted with CH_2Cl_2 (3 × 10 mL). The combined organics were dried over $MgSO_4$, filtered, and concentrated *in vacuo*. Purification by column chromatography using eluents given below afforded the pure product. In general, the products could be purified further by recrystallization from EtOAc if required.

Dimethyl(2-morpholino-2-oxo-1-phenylethyl)sulfonium trifluoromethanesulfonate (2a)

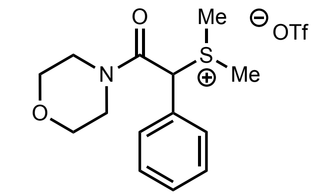

$C_{15}H_{20}F_3NO_5S_2$
MW: 415.44

Synthesised according to **GP-4** from 2-phenyl-1-morpholinoethan-1-one (205 mg, 1.0 mmol), Tf_2O (0.18 mL, 1.1 mmol), and 2-chloropyridine (0.19 mL, 2.0 mmol) in CH_2Cl_2 (3.5 mL) *then* DMSO (0.18 mL in 0.5 mL CH_2Cl_2, 2.5 mmol) and TFAA (0.14 mL, 1.0 mmol). Purification by column chromatography (silica gel; 0-10% MeOH in EtOAc) afforded the product **2a** as an off-white solid (309 mg, 0.74 mmol, 74%).

^{1}H NMR (400 MHz, CDCl$_3$): δ 7.64 – 7.58 (m, 2H, Ar *H*), 7.57 – 7.51 (m, 3H, Ar *H*), 6.76 (s, 1H, C*H*), 3.78 – 3.46 (m, 6H, 2 x CH_2, 2 x CH_{2A}), 3.22 – 3.08 (m, 5H, 2 x CH_{2B}, SCH_3), 2.70 (s, 3H, SCH_3).

^{13}C{^{1}H} NMR (101 MHz, CDCl$_3$): δ 163.5 (*C*=O), 131.9 (Ar *C*H), 130.8 (Ar *C*H), 129.7 (Ar *C*H), 127.0 (Ar *C*), 67.2 (*C*H), 66.4 (CH_2), 66.2 (CH_2), 46.6 (CH_2), 43.3 (CH_2), 26.1 (CH_3), 22.1 (CH_3).

^{19}F NMR (376 MHz, CDCl$_3$): δ -78.37 (s, SO_2CF_3).

HRMS: calcd. for $C_{14}H_{20}NO_2S$ [M-OTf]$^+$: 266.1209; found (ESI$^+$): 266.1207.

ν_{max} (neat/cm^{-1}): 639, 1029, 1164, 1255, 1276, 1449, 1642, 2862, 2935, 3024, 3482.

m.p.: (recrystallized from EtOAc) 138-140 °C.

*The synthesis of **2a** was also carried out according to a **modified GP-4** on a 10 mmol scale as follows:*

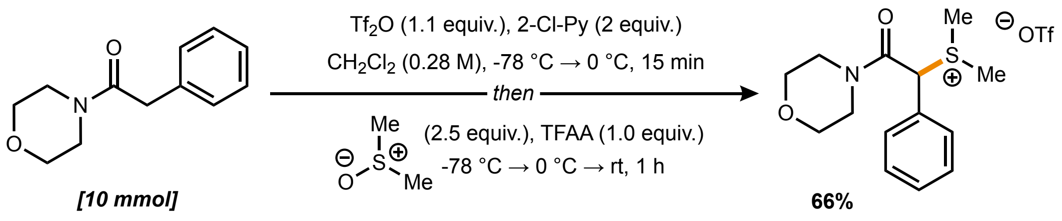

According to the modified literature procedure,[19] an oven-dried flask under an atmosphere of dinitrogen was charged with 2-phenyl-1-morpholinoethan-1-one (2.05 g, 10.0 mmol), 2-chloropyridine (1.89 mL, 20.0 mmol) and anhydrous CH_2Cl_2 (0.28 M, 35 mL). The solution was cooled to -78 °C (dry ice/acetone bath) and Tf_2O (1.85 mL, 11.0 mmol) was added drop-wise over 10 min. After complete addition, the reaction was warmed to 0 °C (ice/water bath) and stirred for 15 min. After cooling once more to -78 °C, DMSO (1.78 mL, 25.0 mmol) in anhydrous CH_2Cl_2 (5 mL) was added drop-wise, followed by drop-wise addition of TFAA (1.39 mL, 10.0 mmol). The reaction mixture was warmed to 0 °C and stirred for 15 min and at rt for 45 min. The reaction was poured onto a vigorously stirred sat. aqueous $NaHCO_3$ solution (50 mL) followed by vigorous stirring for 10 min. The organic layer was separated and the aqueous layer extracted with CH_2Cl_2 (3 × 50 mL). The combined organics were dried over $MgSO_4$, filtered, and concentrated *in vacuo*. Purification by column chromatography (silica gel; 0-5% MeOH in EtOAc) afforded the product **2a** as an off-white solid (2.76 g, 6.64 mmol, 66%).

Characterization data were identical to those listed above.

1-(2-Morpholino-2-oxo-1-phenylethyl)tetrahydro-1*H*-thiophen-1-ium trifluoromethanesulfonate (2b)

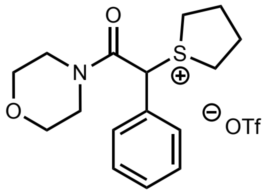

$C_{17}H_{22}F_3NO_5S_2$
MW: 441.48

Synthesised according to **GP-4** from 2-phenyl-1-morpholinoethan-1-one (205 mg, 1.0 mmol), Tf_2O (0.18 mL, 1.1 mmol), and 2-chloropyridine (0.19 mL, 2.0 mmol) in CH_2Cl_2 (3.5 mL) *then* tetrahydrothiophene *S*-oxide (260 mg in 0.5 mL CH_2Cl_2, 2.5 mmol) and TFAA (0.14 mL, 1.0 mmol). Purification by column chromatography (silica gel; 0-10% MeOH in EtOAc) afforded the product **2b** as an off white solid (337 mg, 0.76 mmol, 76%).

^{1}H NMR (400 MHz, CD_3CN): δ 7.63 – 7.57 (m, 2H, Ar *H*), 7.54 – 7.50 (m, 3H, Ar *H*), 6.65 (s, 1H, C*H*S(CH_2)$_2$), 3.79 – 3.47 (m, 8H, 3 x CH_2, 2 x CH_{2A}), 3.35 – 3.25 (m, 1H, 1 x CH_{2A}), 3.14 – 3.02 (m, 3H, 3 x CH_{2B}), 2.49 – 2.37 (m, 2H, CH_2), 2.24 – 2.07 (m, 2H, CH_2).

^{13}C{^{1}H} NMR (101 MHz, CD_3CN): δ 164.3 (*C*=O), 131.7 (Ar *C*H), 130.8 (Ar *C*H), 129.6 (Ar *C*H), 128.4 (Ar *C*), 120.5 (q, J = 319.4 Hz, *C*F_3), 67.0 (*C*HS(CH_2)$_2$), 66.3 (*C*H_2), 66.0 (*C*H_2), 46.6 (*C*H_2), 44.8 (*C*H_2), 43.4 (*C*H_2), 39.2 (*C*H_2), 28.57 (*C*H_2), 28.55 (*C*H_2).

^{19}F NMR (376 MHz, CD_3CN): δ -78.39 (s, SO_2CF_3).

HRMS: calcd. for $C_{16}H_{22}NO_2S$ [M-OTf]$^+$: 292.1366; found (ESI$^+$): 292.1371.

ν_{max} (neat/cm^{-1}): 638, 1030, 1256, 1448, 1639, 1726, 2861, 2946, 3520.

m.p.: (recrystallized from EtOAc) 135-138 °C.

(2-Morpholino-2-oxo-1-phenylethyl)diisopropylsulfonium trifluoromethanesulfonate (2c)

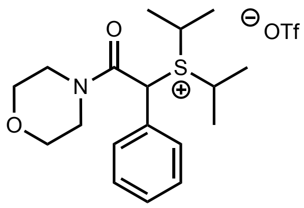

$C_{19}H_{28}F_3NO_5S_2$
MW: 471.55

Synthesised according to **GP-4** from 2-phenyl-1-morpholinoethan-1-one (1.03 g, 5.0 mmol), Tf_2O (0.93 mL, 5.5 mmol), and 2-chloropyridine (0.95 mL, 10.0 mmol) in CH_2Cl_2 (35 mL) *then* diisopropyl sulfoxide (805 mg in 5 mL CH_2Cl_2, 12.5 mmol) and TFAA (0.70 mL, 5.0 mmol). Purification by column chromatography (silica gel; 0-10% MeOH in EtOAc) afforded the product **2c** as an off white solid (1.64 g, 3.48 mmol, 70%).

^{1}H NMR (400 MHz, CD_3CN): δ 7.60 – 7.52 (m, 5H, Ar *H*), 5.90 (s, 1H, C*H*S(iPr)$_2$), 3.88 (pent, *J* = 6.9 Hz, 1H, SC*H*), 3.69 – 3.42 (m, 6H, 2 x CH_2, 2 x CH_{2A}), 3.21 – 3.09 (m, 3H, SC*H*, 2 x CH_{2B}), 1.63 (d, *J* = 6.9 Hz, 3H, CH_3), 1.56 (d, *J* = 6.9 Hz, 3H, CH_3), 1.37 (d, *J* = 7.1 Hz, 3H, CH_3), 1.26 (d, *J* = 6.9 Hz, 3H, CH_3).

^{13}C{^{1}H} NMR (101 MHz, CD_3CN): δ 164.6 (*C*=O), 132.3 (Ar *C*H), 131.5 (Ar *C*H), 130.4 (Ar *C*H), 128.4 (Ar *C*), 122.1 (q, *J* = 320.6 Hz, *C*F$_3$), 66.9 (*C*H$_2$), 66.7 (*C*H$_2$), 61.5 (C*H*S(iPr)$_2$), 47.2 (*C*H$_2$), 45.8 (*C*H), 44.9 (*C*H), 44.1 (*C*H$_2$), 22.0 (*C*H$_3$), 19.7 (*C*H$_3$), 19.6 (*C*H$_3$), 17.4 (*C*H$_3$).

^{19}F NMR (376 MHz, $CDCl_3$): δ -79.35 (s, SO$_2$C*F$_3$*).

HRMS: calcd. for $C_{18}H_{28}NO_2S$ [M-OTf]$^+$: 322.1855; found (ESI$^+$): 322.1849.

ν_{max} (neat/cm^{-1}): 643, 752, 1055, 1149, 1257, 1434, 1608, 2889, 2930, 3062.

m.p.: (recrystallized from EtOAc) 113-115 °C.

(2-(Dimethylamino)-2-oxo-1-phenylethyl)dimethylsulfonium trifluoromethanesulfonate (2d)

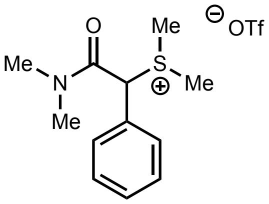

$C_{13}H_{14}F_3NO_4S_2$
MW: 373.41

Synthesised according to **GP-4** from *N*,*N*-dimethyl-2-phenylacetamide (163 mg, 1.0 mmol), Tf_2O (0.18 mL, 1.1 mmol), and 2-chloropyridine (0.19 mL, 2.0 mmol) in CH_2Cl_2 (3.5 mL) *then* DMSO (0.18 mL in 0.5 mL CH_2Cl_2, 2.5 mmol) and TFAA (0.14 mL, 1.0 mmol). Purification by column chromatography (silica gel; 0-10% MeOH in EtOAc) afforded the product **2d** as an off brown oil (330 mg, 0.88 mmol, 88%).

^{1}H NMR (400 MHz, CD_3CN): δ 7.61 – 7.54 (m, 3H, Ar *H*), 7.48 – 7.43 (m, 2H, Ar *H*), 5.87 (s, 1H, C*H*), 2.93 (s, 3H, C*H*3), 2.80 (s, 3H, C*H*3), 2.76 (s, 3H, C*H*3), 2.48 (s, 3H, C*H*3).

^{13}C{^{1}H} NMR (101 MHz, CD_3CN): δ 165.7 (*C*=O), 132.4 (Ar *C*H), 131.2 (Ar *C*H), 130.8 (Ar *C*H), 127.2 (Ar *C*), 121.9 (q, *J* = 320.3 Hz, *C*F$_3$), 67.5 (*C*H), 37.7 (*C*H$_3$), 36.5 (*C*H$_3$), 26.0 (*C*H$_3$), 23.2 (*C*H$_3$).

^{19}F NMR (376 MHz, $CDCl_3$): δ -79.37 (s, SO_2CF_3).

HRMS: calcd. for $C_{18}H_{28}NO_2S$ [M-OTf]$^+$: 224.1104; found (ESI$^+$): 224.1111.

ν_{max} (neat/cm^{-1}): 639, 1030, 1157, 1257, 1648, 2858, 2935, 3031.

(1-(Diisopropylamino)-2-oxo-1-phenylethyl)dimethylsulfonium trifluoromethanesulfonate (2e)

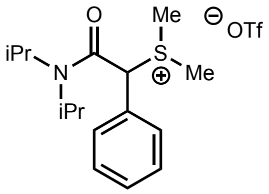

$C_{17}H_{26}F_3NO_4S_2$
MW: 429.51

Synthesised according to **GP-4** from *N,N*-diisopropyl-2-phenylacetamide (219 mg, 1.0 mmol), Tf_2O (0.18 mL, 1.1 mmol), and 2-chloropyridine (0.19 mL, 2.0 mmol) in CH_2Cl_2 (3.5 mL) *then* DMSO (0.18 mL in 0.5 mL CH_2Cl_2, 2.5 mmol) and TFAA (0.14 mL, 1.0 mmol). Purification by column chromatography (silica gel; 0-10% MeOH in EtOAc) afforded the product **2e** as a brown oil (198 mg, 0.54 mmol, 54%).

^{1}H NMR (400 MHz, CD_3CN): δ 7.59 – 7.54 (m, 3H, Ar *H*), 7.48 – 7.44 (m, 2H, Ar *H*), 5.96 (s, 1H, C*H*SMe$_2$), 3.74 (sept, *J* = 6.7 Hz, 1H, NC*H*), 3.46 (sept, *J* = 6.7 Hz, 1H, NC*H*), 2.77 (s, 3H, C*H*$_3$), 2.46 (s, 3H, C*H*$_3$), 1.42 (d, *J* = 6.8 Hz, 3H, C*H*$_3$), 1.33 (d, *J* = 6.8 Hz, 3H, C*H*$_3$), 1.19 (d, *J* = 6.5 Hz, 3H, C*H*$_3$), 0.44 (d, *J* = 6.5 Hz, 3H, C*H*$_3$).

^{13}C{^{1}H} NMR (101 MHz, CD_3CN): δ 164.6 (*C*=O), 132.2 (Ar *C*H), 131.1 (Ar *C*H), 130.8 (Ar *C*H), 127.8 (Ar *C*), 69.3 (*C*HSMe$_2$), 51.6 (*C*H), 47.7 (*C*H), 25.9 (S*C*H$_3$), 23.2 (S*C*H$_3$), 20.6 (*C*H$_3$), 20.5 (*C*H$_3$), 19.6 (*C*H$_3$), 19.0 (*C*H$_3$). *The quaternary carbon corresponding to the CF_3 in the triflate counter anion was not observed, though its presence was confirmed by ^{19}F NMR spectroscopy.*

^{19}F NMR (376 MHz, CD_3CN): -79.33 (s, SO$_2$C*F*$_3$)

HRMS: calcd. for $C_{16}H_{26}NOS$ [M-OTf]$^+$: 280.1730; found (ESI$^+$): 280.1720.

ν_{max} (neat/cm^{-1}): 637, 757, 1029, 1155, 1250, 1451, 1632, 2938, 2974, 3465.

(2-(Methoxy(methyl)amino)-2-oxo-1-phenylethyl)dimethylsulfonium trifluoromethanesulfonate (2f)

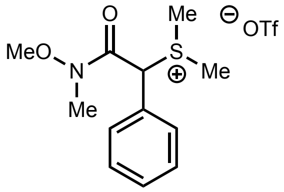

$C_{13}H_{18}F_3NO_5S_2$
MW: 389.40

Synthesised according to **GP-4** from *N*-methoxy-*N*-methyl-2-phenylacetamide (179 mg, 1.0 mmol), Tf_2O (0.18 mL, 1.1 mmol), and 2-chloropyridine (0.19 mL, 2.0 mmol) in CH_2Cl_2 (3.5 mL) *then* DMSO (0.18 mL in 0.5 mL CH_2Cl_2, 2.5 mmol) and TFAA (0.14 mL, 1.0 mmol). Purification by column chromatography (silica gel; 0-2% MeOH in EtOAc) afforded the product **2d** as a viscous yellow oil (196 mg, 0.504 mmol, 50%).

^{1}H NMR (400 MHz, $CDCl_3$): δ 7.63 – 7.58 (m, 2H, Ar *H*), 7.56 – 7.51 (m, 3H, Ar *H*), 6.64 (s, 1H, C*H*SMe$_2$), 3.61 (s, 3H, OC*H$_3$*), 3.25 (s, 3H, SC*H$_3$*), 3.20 (s, 3H, SC*H$_3$*), 2.76 (s, 3H, NC*H$_3$*).

^{13}C{^{1}H} NMR (101 MHz, $CDCl_3$): δ 165.3 (*C*=O), 131.4 (Ar *C*), 130.3 (Ar *C*H), 129.7 (Ar *C*H), 127.4 (Ar *C*H), 120.7 (q, *J* = 319.5 Hz, SO$_2$*C*F$_3$), 66.5 (*C*HSMe$_2$), 62.0 (O*C*H$_3$), 32.6 (N*C*H$_3$), 25.4 (S*C*H$_3$), 21.8 (S*C*H$_3$).

^{19}F NMR (376 MHz, $CDCl_3$): δ -78.41 (s, SO$_2$C*F$_3$*).

HRMS: calcd. for $C_{12}H_{18}NO_2S$ [M-OTf]$^+$: 240.1058; found (ESI$^+$): 240.1052.

ν_{max} (neat/cm^{-1}): 638, 1030, 1157, 1256, 1432, 1656, 2940, 3022.

(2-(Pyrrolidin-1-yl)-2-oxo-1-phenylethyl)dimethylsulfonium trifluoromethanesulfonate (2g)

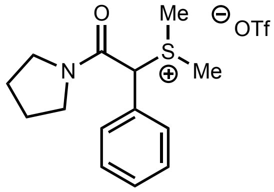

$C_{15}H_{20}F_3NO_4S_2$
MW: 399.44

Synthesised according to **GP-4** from 2-phenyl-1-(pyrrolidin-1-yl)ethan-1-one (189 mg, 1.0 mmol), Tf_2O (0.18 mL, 1.1 mmol), and 2-chloropyridine (0.19 mL, 2.0 mmol) in CH_2Cl_2 (3.5 mL) *then* DMSO (0.18 mL in 0.5 mL CH_2Cl_2, 2.5 mmol) and TFAA (0.14 mL, 1.0 mmol). Purification by column chromatography (silica gel; 0-10% MeOH in EtOAc) afforded the product **2g** as a brown oil (327 mg, 0.82 mmol, 82%).

^{1}H NMR (400 MHz, CD_3CN): δ 7.60 – 7.53 (m, 3H, Ar *H*), 7.49 – 7.44 (m, 2H, Ar *H*), 5.64 (s, 1H, C*H*SMe$_2$), 3.52 – 3.42 (m, 2H, 2 x CH_{2A}), 3.41 – 3.34 (m, 1H, CH_{2B}), 2.80 (s, 3H, CH_3), 2.78 – 2.71 (m, 1H, CH_{2B}), 2.48 (s, 3H, CH_3), 1.90 – 1.80 (m, 2H, C$H_{2A,}$ CH_{2B}), 1.79 – 1.67 (m, 2H, C$H_{2A,}$ CH_{2B}).

^{13}C{^{1}H} NMR (101 MHz, CD_3CN): δ 163.7 (*C*=O), 132.3 (Ar *C*H), 131.1 (Ar *C*H), 131.0 (Ar *C*H), 127.1 (Ar *C*), 67.6 (*C*HSMe$_2$), 47.7 (*C*H$_2$), 47.5 (*C*H$_2$), 26.5 (*C*H$_2$), 26.0 (*C*H$_3$), 24.6 (*C*H$_2$), 23.1 (*C*H$_3$). *The quaternary carbon corresponding to the CF_3 in the triflate counter anion was not observed, though its presence was confirmed by ^{19}F NMR spectroscopy.*

^{19}F NMR (376 MHz, CD_3CN): -79.30 (s, SO$_2$C*F$_3$*).

HRMS: calcd. for $C_{14}H_{20}NOS$ [M-OTf]$^+$: 250.1260; found (ESI$^+$): 250.1265.

v_{max} (neat/cm^{-1}): 639, 1032, 1163, 1260, 1449, 1640, 2849, 2917, 3024.

(2-(Piperidin-1-yl)-2-oxo-1-phenylethyl)dimethylsulfonium trifluoromethanesulfonate (2h)

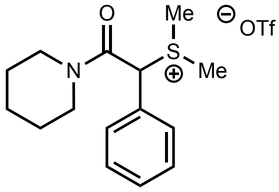

$C_{16}H_{22}F_3NO_4S_2$
MW: 413.47

Synthesised according to **GP-4** from 1-(piperidin-1-yl)-2-phenylethan-1-one (xx mg, 1.0 mmol), Tf_2O (0.18 mL, 1.1 mmol), and 2-chloropyridine (0.19 mL, 2.0 mmol) in CH_2Cl_2 (3.5 mL) *then* DMSO (0.18 mL in 0.5 mL CH_2Cl_2, 2.5 mmol) and TFAA (0.14 mL, 1.0 mmol). Purification by column chromatography (silica gel; 0-10% MeOH in EtOAc) afforded the product **2h** as a pale yellow solid (321 mg, 0.78 mmol, 78%).

^{1}H NMR (400 MHz, CD_3CN): 7.63 – 7.53 (m, 3H, Ar *H*), 7.48 – 7.43 (m, 2H, Ar *H*), 5.87 (s, 1H, C*H*), 3.77 – 3.69 (m, 1H, CH_{2A}), 3.37 – 3.29 (m, 1H, CH_{2B}), 3.25 – 3.11 (m, 2H, CH_2), 2.77 (s, 3H, CH_3), 2.47 (s, 3H, CH_3), 1.60 – 1.47 (m, 3H, CH_2 + CH_{2A}), 1.44 – 1.31 (m, 2H, CH_2), 0.81 – 0.71 (m, 1H, CH_{2B}).

^{13}C{^{1}H} NMR (101 MHz, CD_3CN): 163.8 (*C*=O), 132.3 (Ar *C*H), 131.2 (Ar *C*H), 130.8 (Ar *C*H), 127.6 (Ar *C*), 122.0 (q, J = 320.3 Hz, SO_2CF_3), 67.7 (*C*H), 47.9 (*C*H_2), 44.6 (*C*H_2), 26.0 (*C*H_2), 25.9 (*C*H_3 + *C*H_2), 24.4 (*C*H_2), 23.3 (*C*H_3).

^{19}F NMR (376 MHz, CD_3CN): -79.35 (s, SO_2CF_3)

HRMS: calcd. for $C_{15}H_{22}NOS$ [M-OTf]$^+$: 264.1417; found (ESI$^+$): 264.1416.

ν_{max} (neat/cm^{-1}): 639, 893, 1032, 1256, 1455, 1637, 2861, 2941, 3025, 3337.

(2-(Piperidin-1-yl)-2-oxo-1-((4-trifluoromethyl)phenyl)ethyl)dimethylsulfonium trifluoromethanesulfonate (2h′)

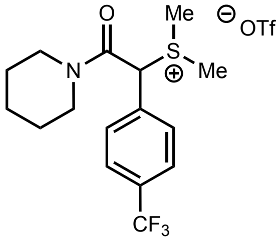

$C_{17}H_{21}F_6NO_4S_2$
MW: 481.47

Synthesised according to **GP-4** from 1-(piperidin-1-yl)-2-(4-(trifluoromethyl)phenyl)ethan-1-one (269 mg, 1.0 mmol), Tf_2O (0.18 mL, 1.1 mmol), and 2-chloropyridine (0.19 mL, 2.0 mmol) in CH_2Cl_2 (3.5 mL) *then* DMSO (0.18 mL in 0.5 mL CH_2Cl_2, 2.5 mmol) and TFAA (0.14 mL, 1.0 mmol). Purification by column chromatography (silica gel; 0-10% MeOH in EtOAc) afforded the product **2h′** as a brown oil (105 mg, 0.20 mmol, 20%).

^{1}H NMR (400 MHz, CD_3CN): 7.91 – 7.84 (m, 2H, Ar *H*), 7.69 – 7.62 (m, 2H, Ar *H*), 6.00 (s, 1H, C*H*SMe_2), 3.68 (ddd, *J* = 13.1, 6.3, 3.7 Hz, 1H, C*H*$_2$), 3.44 – 3.33 (m, 1H, C*H*$_2$), 3.23 (ddd, *J* = 13.7, 8.5, 3.5 Hz, 1H, C*H*$_2$), 3.12 (ddd, *J* = 13.7, 6.4, 3.9 Hz, 1H, C*H*$_2$), 2.81 (s, 3H, C*H*$_3$), 2.51 (s, 3H, C*H*$_3$), 1.63 – 1.33 (m, 5H, C*H*$_2$), 0.86 – 0.73 (m, 1H, C*H*$_2$).

^{13}C{^{1}H} NMR (101 MHz, CD_3CN): 163.3 (*C*=O), 133.4 (q, *J* = 32.8 Hz, Ar *C*), 132.0 (Ar *C*H), 131.7 (Ar *C*), 128.1 (q, *J* = 3.9 Hz, Ar *C*H), 124.8 (q, *J* = 271.9 Hz, *C*F_3), 66.5 (*C*HSMe_2), 48.0 (*C*H_2), 44.6 (*C*H_2), 26.1 (*C*H_2), 26.0 (*C*H_2), 25.9 (*C*H_2), 24.3 (S*C*H_3), 23.3 (S*C*H_3). *The quaternary carbon corresponding to the CF_3 in the triflate counter anion was not observed, though its presence was confirmed by ^{19}F NMR spectroscopy.*

^{19}F NMR (376 MHz, CD_3CN): -63.55 (s, C*F*$_3$), -79.34 (s, SO_2C*F*$_3$).

HRMS: calcd. for $C_{16}H_{21}F_3NOS$ [M-OTf]$^+$: 332.1290; found (ESI$^+$): 332.1298.

ν_{max} (neat/cm^{-1}): 646, 857, 1033, 1160, 1259, 1665, 1720, 2859, 3487.

(2-Oxo-1-phenyl-2-(4-tosylpiperazin-1-yl)ethyl)dimethylsulfonium trifluoromethanesulfonate (2i)

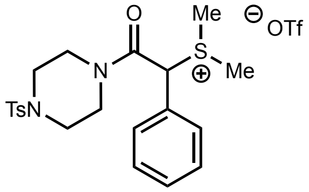

$C_{22}H_{27}F_3N_2O_6S_3$
MW: 568.64

Synthesised according to **GP-4** from 2-phenyl-1-(4-tosylpiperazin-1-yl)ethan-1-one (358 mg, 1.0 mmol), Tf_2O (0.18 mL, 1.1 mmol), and 2-chloropyridine (0.19 mL, 2.0 mmol) in CH_2Cl_2 (3.5 mL) *then* DMSO (0.18 mL in 0.5 mL CH_2Cl_2, 2.5 mmol) and TFAA (0.14 mL, 1.0 mmol). Purification by column chromatography (silica gel; 0-2% MeOH in EtOAc) afforded the product **2i** as a yellow solid (426 mg, 0.748 mmol, 75%).

^{1}H NMR (400 MHz, CD_3CN): δ 7.56 – 7.47 (m, 3H, Ar *H*), 7.46 – 7.37 (m, 4H, Ar *H*), 7.37 – 7.32 (m, 2H, Ar *H*), 5.91 (s, 1H, C*H*SMe_2), 3.74 (ddd, *J* = 13.4, 6.3, 3.2 Hz, 1H, NCH_2), 3.54 (ddd, *J* = 13.4, 7.8, 3.3 Hz, 1H, NCH_2), 3.39 (ddd, *J* = 14.0, 7.8, 3.3 Hz, 1H, NCH_2), 3.19 (dddd, *J* = 14.0, 6.3, 3.3, 1.0 Hz, 1H, NCH_2), 3.06 (dddd, *J* = 11.8, 6.3, 3.3, 1.0 Hz, 1H, NCH_2), 2.86 (dddd, *J* = 11.8, 6.3, 3.3, 1.0 Hz, 1H, NCH_2), 2.76 (s, 3H), 2.70 (ddd, *J* = 11.8, 7.8, 3.3 Hz, 1H, NCH_2), 2.46 (s, 3H), 2.45 (s, 3H), 2.01 – 1.95 (m, 1H, NCH_2).

^{13}C{^{1}H} NMR (101 MHz, CD_3CN): δ 164.5 (C=O), 145.5 (Ar *C*), 132.8 (Ar *C*), 132.4 (Ar *C*), 131.2 (Ar *C*H), 130.9 (Ar *C*H), 130.8 (Ar *C*H), 128.6 (Ar *C*H), 127.0 (Ar *C*H), 122.1 (q, *J* = 320.8 Hz, SO_2CF_3), 66.8 (*C*$HSMe_2$), 46.3 (*C*H_2), 46.1 (*C*H_2), 45.9 (*C*H_2), 42.9 (*C*H_2), 25.9 (S*C*H_3), 23.1 (S*C*H_3), 21.5 (*C*H_3).

^{19}F NMR (376 MHz, $CDCl_3$): δ -78.40 (s, SO_2CF_3).

HRMS: calcd. for $C_{21}H_{27}N_2O_3S_2$ [M-OTf]$^+$: 419.1463; found (ESI$^+$): 419.1480.

ν_{max} (neat/cm^{-1}): 724, 913, 1028, 1163, 1223, 1254, 1348, 1452, 1639, 2938.

m.p.: (recrystallized from EtOAc) 149-150 °C.

(2-(Methyl(3-phenyl-3-(4-(trifluoromethyl)phenoxy)propyl)amino)-2-oxo-1-phenylethyl) dimethylsulfonium trifluoromethanesulfonate (2j)

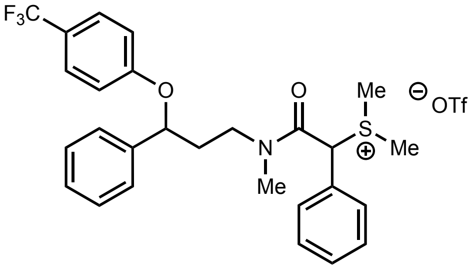

$C_{28}H_{29}F_6NO_5S_2$
MW: 637.65

Synthesised according to **GP-4** from *N*-(2-phenylacetyl)fluoxetine (855 mg, 2.0 mmol), Tf_2O (0.37 mL, 2.2 mmol), and 2-chloropyridine (0.38 mL, 4.0 mmol) in CH_2Cl_2 (7 mL) *then* DMSO (0.36 mL in 1 mL CH_2Cl_2, 5.0 mmol) and TFAA (0.28 mL, 2.0 mmol). Purification by column chromatography (silica gel; 50-100% EtOAc in hexanes then 0-1% MeOH in EtOAc) afforded the product **2j** as a yellow foam as an inseparable mixture of diastereomers and rotamers (771 mg, 1.21 mmol, 60%).

^{1}H NMR (400 MHz, $CDCl_3$): δ 7.69 – 7.20 (m, 12H, Ar *H*), 6.97 – 6.81 (m, 2H, Ar *H*), 6.79 – 6.73 (m, 1H, C*H*SMe$_2$), 5.30 – 5.06 (m, 1H, OC*H*), 4.27 – 4.06 (m, 0.5H, C*H$_2$*), 3.79 – 3.59 (m, 0.5H, C*H$_2$*), 3.57 – 3.21 (m, 1H, C*H$_2$*), 3.21 – 2.77 (m, 6H, SC*H$_3$*, NC*H$_3$*), 2.76 – 2.60 (m, 3H, SC*H$_3$*), 2.24 – 2.04 (m, 2H, C*H$_2$*).

^{13}C{^{1}H} NMR (101 MHz, $CDCl_3$): δ 165.1 (*C*=O), 164.9 (*C*=O), 164.7 (*C*=O), 140.42 (Ar *C*), 140.39 (Ar *C*), 140.1 (Ar *C*), 139.8 (Ar *C*), 131.7 (Ar *C*H), 131.64 (Ar *C*H), 131.60 (Ar *C*H), 130.64 (Ar *C*H), 130.60 (Ar *C*H), 129.1 (Ar *C*H), 129.98 (Ar *C*H), 128.95 (Ar *C*H), 128.2 (Ar *C*H), 128.10 (Ar *C*H), 127.4 (Ar *C*H), 127.3 (Ar *C*H), 127.1 – 126.8 (m, Ar *C*H), 126.7 (Ar *C*H), 125.8 (q, *J* = 4.3 Hz, Ar *C*H), 123.5 – 122.4 (m, Ar *C*), 120.7 (q, *J* = 319.7 Hz, *C*F$_3$), 116.0 (Ar *C*H), 115.9 (Ar *C*H), 77.3 (OC*H*), 76.5 (OC*H*), 67.9 (*C*HSMe$_2$), 67.6 (*C*HSMe$_2$), 67.51 (*C*HSMe$_2$), 67.48 (*C*HSMe$_2$), 47.3 (*C*H$_2$), 46.6 (*C*H$_2$), 45.4 (*C*H$_2$), 36.4 (N*C*H$_3$), 36.3 (N*C*H$_3$), 36.1 (N*C*H$_3$), 35.70 (CH$_2$), 35.65 (N*C*H$_3$), 35.4 (N*C*H$_3$), 34.2 (N*C*H$_3$), 34.1 (N*C*H$_3$), 26.0 (S*C*H$_3$), 25.79 (S*C*H$_3$), 25.75 (S*C*H$_3$), 25.7 (S*C*H$_3$), 22.0 (S*C*H$_3$), 21.9 (S*C*H$_3$). *The quaternary carbon corresponding to one CF$_3$ was not observed, though its presence was confirmed by ^{19}F NMR spectroscopy.*

^{19}F NMR (376 MHz, CD_3CN): δ -61.54 (C*F$_3$*), -61.59 (C*F$_3$*), -61.61 (C*F$_3$*), -78.32, (SO$_2$C*F$_3$*).

HRMS: calcd. for $C_{27}H_{29}F_3NO_2S$ [M-OTf]$^+$: 488.1871; found (ESI$^+$): 488.1891.

ν_{max} (neat/cm^{-1}): 1027, 1109, 1151, 1223, 1241, 1274, 1451, 1639, 2932.

(2-((3-(9,10-Ethanoanthracen-9(10*H*)-yl)propyl)(methyl)amino)-2-oxo-1-phenylethyl)dimethylsulfonium trifluoromethanesulfonate (2k)

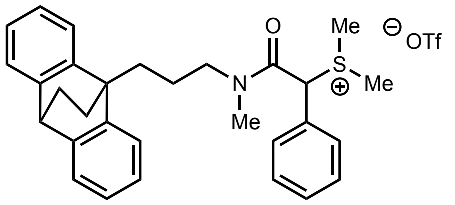

Synthesised according to **GP-4** from *N*-(2-phenylacetyl)maprotiline (427 mg, 1.0 mmol), Tf_2O (0.18 mL, 1.1 mmol), and 2-chloropyridine (0.19 mL, 2.0 mmol) in CH_2Cl_2 (3.5 mL) *then* DMSO (0.18 mL in 0.5 mL CH_2Cl_2, 2.5 mmol) and TFAA (0.14 mL, 1.0 mmol). Purification by column chromatography (silica gel; 50-100% EtOAc in hexanes then 0-2% MeOH in EtOAc) afforded the product **2k** as a yellow foam (314 mg, 0.519 mmol, 52%).

A 2:1 mixture of rotamers was observed at 298 K in $CDCl_3$.

^{1}H NMR (400 MHz, $CDCl_3$): δ 7.81 – 7.65 (m, 2H, Ar *H*), 7.60 – 7.44 (m, 3H, Ar *H*), 7.32 – 7.03 (m, 8H, Ar *H*), 6.97 (s, 0.33H, C*H*SMe$_2$), 6.96 – 6.83 (m, 0.66H, Ar *H*), 6.79 (s, 0.66H, C*H*SMe$_2$), 4.33 – 4.20 (m, 1H, C*H*), 4.07 – 3.81 (m, 1H, NCH_{2A}), 3.52 – 3.35 (m, 1H, NCH_{2B}), 3.29 – 3.15 (m, 3H, SCH_3), 3.13 – 3.03 (m, 3H, NCH_3), 2.76 – 2.69 (m, 3H, SCH_3), 2.50 – 2.32 (m, 1.33H, CH_2), 2.12 – 1.93 (m, 1.66H, CH_2), 1.91 – 1.70 (m, 2H, CH_2), 1.62 – 1.44 (m, 1.33H, CH_2), 1.25 – 1.17 (m, 0.33H, CH_2), 1.12 – 0.95 (m, 0.33H, CH_2).

^{13}C NMR (101 MHz, $CDCl_3$): δ 165.1 (*C*=O), 164.6 (*C*=O), 145.1 (Ar *C*), 145.03 (Ar *C*), 145.02 (Ar *C*), 144.9 (Ar *C*), 144.8 (Ar *C*), 144.6 (Ar *C*), 131.84 (Ar *C*H), 131.75 (Ar *C*H), 130.8 (Ar *C*H), 130.7 (Ar *C*H), 129.8 (Ar *C*H), 127.9 (Ar *C*), 126.9 (Ar *C*H), 125.52 (Ar *C*H), 125.50 (Ar *C*H), 125.50 (Ar *C*H), 125.43 (Ar *C*H), 125.41 (Ar *C*H), 123.64 (Ar *C*H), 123.61 (Ar *C*H), 123.57 (Ar *C*H), 123.4 (Ar *C*H), 120.7 (q, J = 319.9 Hz, SO$_2$CF_3), 121.14 (Ar *C*H), 121.07 (Ar *C*H), 121.0 (Ar *C*H), 68.0 (*C*HSMe$_2$), 67.9 (*C*HSMe$_2$), 50.9 (N*C*H$_2$), 50.0 (N*C*H$_2$), 44.7 (*C*H), 44.53 (*C*H), 44.45 (*C*H), 44.4 (*C*H), 35.7 (N*C*H$_3$), 33.9 (N*C*H$_3$), 29.6 (*C*H$_2$), 28.0 (*C*H$_2$), 27.64 (*C*H$_2$), 27.55 (*C*H$_2$), 27.5 (*C*H$_2$), 26.4 (S*C*H$_3$), 26.2 (S*C*H$_3$), 23.0 (*C*H$_2$), 22.5 (*C*H$_2$), 22.0 (S*C*H$_3$), 21.8 (S*C*H$_3$).

^{19}F NMR (376 MHz, CD_3CN): δ -78.31 (s, SO$_2$CF_3).

HRMS: calcd. for $C_{30}H_{34}NOS$ [M-OTf]$^+$: 456.2361; found (ESI$^+$): 456.2377.

ν_{max} (neat/cm^{-1}): 1027, 1150, 1223, 1240, 1276, 1450, 1646, 2862, 2931.

(1-(4-Trifluoromethylphenyl)-2-morpholino-2-oxoethyl)dimethylsulfonium trifluoromethanesulfonate (2l)

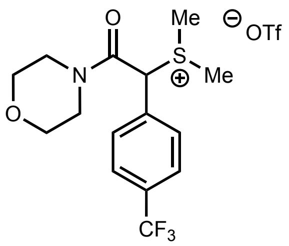

$C_{16}H_{19}F_6NO_5S_2$
MW: 483.44

Synthesised according to **GP-4** from 2-(4-trifluoromethylphenyl)-1-morpholinoethan-1-one (273 mg, 1.0 mmol), Tf_2O (0.18 mL, 1.1 mmol), and 2-chloropyridine (0.19 mL, 2.0 mmol) in CH_2Cl_2 (3.5 mL) *then* DMSO (0.18 mL in 0.5 mL CH_2Cl_2, 2.5 mmol) and TFAA (0.14 mL, 1.0 mmol). Purification by column chromatography (silica gel; 10% MeOH in EtOAc) afforded the product **2l** as an off-white solid (367 mg, 0.76 mmol, 76%).

^{1}H NMR (400 MHz, CD_3CN): δ 7.88 (d, J = 8.1 Hz, 2H, Ar *H*), 7.66 (d, J = 8.1 Hz, 2H, Ar *H*), 6.03 (s, 1H, C*H*SMe$_2$), 3.67 – 3.47 (m, 5H, 2 x CH_2, 1 x CH_{2A}), 3.39 – 3.31 (m, 1H, CH_{2A}), 3.12 – 3.06 (m, 1H, CH_{2B}), 3.05 – 2.98 (m, 1H, CH_{2B}) 2.84 (s, 3H, CH_3), 2.53 (s, 3H, CH_3).

^{13}C{^{1}H} NMR (101 MHz, CD_3CN): δ 164.0 (*C*=O), 133.4 (q, J = 32.8 Hz, Ar *C*), 131.8 (Ar *C*H), 131.5 (q, J = 1.3 Hz, Ar *C*), 128.2 (q, J = 3.8 Hz, Ar *C*H), 124.8 (q, J = 271.9 Hz, *C*F$_3$), 122.1 (q, J = 320.7 Hz, SO$_2$*C*F$_3$), 66.8 (*C*H$_2$), 66.4 (*C*H$_2$), 65.8 (*C*HSMe$_2$), 47.2 (*C*H$_2$), 43.9 (*C*H$_2$), 25.9 (*C*H$_3$), 23.3 (*C*H$_3$).

^{19}F NMR (376 MHz, CD_3CN): δ -63.57 (s, 3F, CF_3), -79.32 (s, 3F, SO$_2$CF_3).

HRMS: calcd. for $C_{15}H_{19}F_3NO_2S$ [M-OTf]$^+$: 334.1083; found (ESI$^+$): 334.1092.

ν_{max} (neat/cm^{-1}): 639, 857, 1030, 1164, 1253, 1447, 1643, 1721, 2861, 2935, 3515.

m.p.: (recrystallized from EtOAc) 186–190 °C.

(1-(4-Methoxyphenyl)-2-morpholino-2-oxoethyl)dimethylsulfonium trifluoromethanesulfonate (2m)

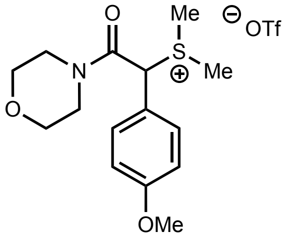

$C_{16}H_{22}F_3NO_6S_2$
MW: 445.47

Synthesised according to **GP-4** from 2-(4-methoxyphenyl)-1-morpholinoethan-1-one (235 mg, 1.0 mmol), Tf_2O (0.18 mL, 1.1 mmol), and 2-chloropyridine (0.19 mL, 2.0 mmol) in CH_2Cl_2 (3.5 mL) *then* DMSO (0.18 mL in 0.5 mL CH_2Cl_2, 2.5 mmol) and TFAA (0.14 mL, 1.0 mmol). Purification by column chromatography (silica gel; 10% MeOH in EtOAc) afforded the product **2m** as a brown oil (385 mg, 0.87 mmol, 87%).

^{1}H NMR (400 MHz, CD_3CN): δ 7.38 (d, J = 8.9 Hz, 2H, Ar *H*), 7.09 (d, J = 8.9 Hz, 2H, Ar *H*), 5.90 (s, 1H, C*H*SMe$_2$), 3.84 (s, 3H, OC*H*$_3$), 3.66 – 3.46 (m, 5H, 2 x C*H*$_2$, 1 x C*H*$_{2A}$), 3.38 – 3.31 (m, 1H, C*H*$_{2A}$) 3.10 – 3.00 (m, 2H, 2 x C*H*$_{2B}$), 2.76 (s, 3H, C*H*$_3$), 2.48 (s, 3H, C*H*$_3$).

^{13}C{^{1}H} NMR (101 MHz, CD_3CN): δ 164.8 (*C*=O), 162.9 (Ar *C*), 132.5 (Ar *C*H), 118.5 (Ar *C*), 116.5 (Ar *C*H), 67.0 (*C*HSMe$_2$), 66.9 (*C*H$_2$), 66.5 (*C*H$_2$), 56.4 (O*C*H$_3$), 47.2 (*C*H$_2$), 43.8 (*C*H$_2$), 25.6 (*C*H$_3$), 23.1 (*C*H$_3$). *The quaternary carbon corresponding to the CF$_3$ in the triflate counter anion was not observed, though its presence was confirmed by ^{19}F NMR spectroscopy.*

^{19}F NMR (376 MHz, CD_3CN): -79.31 (s, SO$_2$C*F*$_3$).

HRMS: calcd. for $C_{15}H_{22}NO_3S$ [M+H]$^+$: 296.1315; found (ESI$^+$): 296.1323.

ν_{max} (neat/cm^{-1}): 639, 762, 1031, 1166, 1260, 1447, 1514, 1642, 2932, 3496.

(1-(4-Methylphenyl)-2-morpholino-2-oxoethyl)dimethylsulfonium trifluoromethanesulfonate (2n)

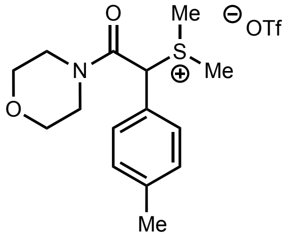

$C_{16}H_{22}F_3NO_5S_2$
MW: 429.47

Synthesised according to **GP-4** from 2-(4-methylphenyl)-1-morpholinoethan-1-one (235 mg, 1.0 mmol), Tf_2O (0.18 mL, 1.1 mmol), and 2-chloropyridine (0.19 mL, 2.0 mmol) in CH_2Cl_2 (3.5 mL) *then* DMSO (0.18 mL in 0.5 mL CH_2Cl_2, 2.5 mmol) and TFAA (0.14 mL, 1.0 mmol). Purification by column chromatography (silica gel; 0-10% MeOH in EtOAc) afforded the product **2n** as a tan solid (285 mg, 0.663 mmol, 66%).

^{1}H NMR (400 MHz, $CDCl_3$): δ 7.47 (d, J = 8.0 Hz, 2H, Ar *H*), 7.32 (d, J = 8.0 Hz, 2H, Ar *H*), 6.72 (s, 1H, C*H*SMe$_2$), 3.78 – 3.53 (m, 5H, 2 × CH_2, CH_{2A}), 3.49 (ddd, J = 12.8, 6.9, 3.3 Hz, 1H, CH_{2B}), 3.21 – 3.06 (m, 2H, CH_2), 3.12 (s, 3H, SCH_3), 2.68 (s, 3H, SCH_3), 2.41 (s, 3H, CH_3).

^{13}C{^{1}H} NMR (101 MHz, $CDCl_3$): δ 163.7 (*C*=O), 142.5 (Ar *C*), 131.4 (Ar *C*), 129.6 (Ar *C*H), 123.8 (Ar *C*H), 120.7 (q, J = 319.4 Hz, SO$_2$*C*F$_3$), 67.1 (*C*H), 66.4 (*C*H$_2$), 66.2 (*C*H$_2$), 46.6 (*C*H$_2$), 43.3 (*C*H$_2$), 25.9 (S*C*H$_3$), 22.0 (S*C*H$_3$), 21.6 (*C*H$_3$).

^{19}F NMR (376 MHz, CD_3CN): δ -78.41 (s, SO$_2$CF_3).

HRMS: calcd. for $C_{15}H_{22}NO_2S$ [M-OTf]$^+$: 280.1371; found (ESI$^+$): 280.1384.

ν_{max} (neat/cm^{-1}): 847, 1027, 1113, 1152, 1219, 1256, 1448, 1464, 1635, 2862, 2934.

m.p.: (recrystallized from EtOAc) 149-150 °C.

(1-(4-Acetoxyphenyl)-2-morpholino-2-oxoethyl)dimethylsulfonium trifluoromethanesulfonate (2o)

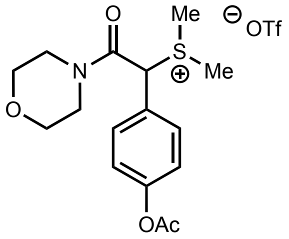

$C_{17}H_{22}F_3NO_7S_2$
MW: 473.48

Synthesised according to **GP-4** from 2-(4-acetoxyphenyl)-1-morpholinoethan-1-one (263 mg, 1.0 mmol), Tf_2O (0.18 mL, 1.1 mmol), and 2-chloropyridine (0.19 mL, 2.0 mmol) in CH_2Cl_2 (3.5 mL) *then* DMSO (0.18 mL in 0.5 mL CH_2Cl_2, 2.5 mmol) and TFAA (0.14 mL, 1.0 mmol). Purification by column chromatography (silica gel; 0-10% MeOH in EtOAc) afforded the product **2o** as a yellow foam (275 mg, 0.58 mmol, 58%).

^{1}H NMR (400 MHz, CD_3CN): δ 7.53 – 7.47 (m, 2H, Ar *H*), 7.36 – 7.28 (m, 2H, Ar *H*), 5.96 (s, 1H, C*H*SMe_2), 3.67 – 3.45 (m, 5H, CH_2, CH_{2A}), 3.42 – 3.31 (m, 1H, CH_{2B}), 3.15 – 3.00 (m, 2H, CH_2), 2.81 (s, 3H, SCH_3), 2.51 (s, 3H, SCH_3), 2.27 (s, 3H, COCH_3).

^{13}C NMR (101 MHz, CD_3CN): δ 170.0 (*C*=O), 164.4 (*C*=O), 154.2 (Ar *C*), 132.3 (Ar *C*H), 124.7 (Ar *C*H), 124.4 (Ar *C*), 122.1 (d, *J* = 320.4 Hz, SO_2CF_3), 66.8 (*C*H_2), 66.4 (*C*H_2), 66.3 (*C*HSMe_2), 47.3 (*C*H_2), 43.9 (*C*H_2), 25.8 (S*C*H_3), 23.3 (S*C*H_3), 21.3 (*C*H_3).

^{19}F NMR (376 MHz, CD_3CN): δ -79.27 (s, SO_2CF_3).

HRMS: calcd. for $C_{16}H_{22}NO_4S$ $[M-OTf]^+$: 324.1270; found (ESI^+): 324.1277.

(1-(4-(1,3-Dioxoisoindolin-2-yl)phenyl)-2-morpholino-2-oxoethyl)dimethylsulfonium trifluoromethanesulfonate (2p)

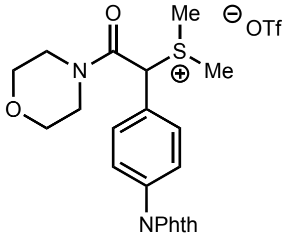

$C_{23}H_{23}F_3N_2O_7S_2$
MW: 560.56

Synthesised according to **GP-4** from 2-(4-(2-morpholino-2-oxoethyl)phenyl)isoindoline-1,3-dione (350 mg, 1.0 mmol), Tf_2O (0.18 mL, 1.1 mmol), and 2-chloropyridine (0.19 mL, 2.0 mmol) in CH_2Cl_2 (3.5 mL) *then* DMSO (0.18 mL in 0.5 mL CH_2Cl_2, 2.5 mmol) and TFAA (0.14 mL, 1.0 mmol). Purification by column chromatography (silica gel; 0-10% MeOH in EtOAc) afforded the product **2p** as a yellow foam (314 mg, 0.56 mmol, 56%).

^{1}H NMR (400 MHz, CD_3CN): δ 7.99 – 7.92 (m, 2H, Ar *H*), 7.91 – 7.86 (m, 2H, Ar *H*), 7.74 – 7.70 (m, 2H, Ar *H*), 7.65 – 7.60 (m, 2H, Ar *H*), 5.99 (s, 1H, C*H*SMe$_2$), 3.70 – 3.48 (m, 5H, CH_2, CH_{2A}), 3.41 (ddd, J = 13.5, 6.2, 3.1 Hz, 1H, CH_{2B}), 3.17 (ddd, J = 11.6, 6.2, 3.1 Hz, 1H, CH_{2A}), 3.09 (ddd, J = 13.5, 6.8, 3.1 Hz, 1H, CH_{2B}), 2.84 (s, 3H, SCH_3), 2.56 (s, 3H, SCH_3).

^{13}C NMR (101 MHz, CD_3CN): δ 168.0 (*C*=O), 164.3 (*C*=O), 136.1 (Ar *C*), 135.9 (Ar *C*H), 132.8 (Ar *C*), 131.5 (Ar *C*H), 129.1 (Ar *C*H), 126.3 (Ar *C*), 124.5 (Ar *C*H), 66.9 (*C*H$_2$), 66.5 (*C*H$_2$), 66.4 (*C*HSMe$_2$), 47.3 (*C*H$_2$), 43.9 (*C*H$_2$), 26.0 (S*C*H$_3$), 23.4 (S*C*H$_3$). *The quaternary carbon corresponding to the CF_3 in the triflate counter anion was not observed, though its presence was confirmed by ^{19}F NMR spectroscopy.*

^{19}F NMR (376 MHz, CD_3CN): δ -79.28 (s, SO$_2$CF_3).

HRMS: calcd. for $C_{22}H_{23}NO_4S$ [M-OTf]$^+$: 411.1379; found (ESI$^+$): 411.1379.

(1-(4-Fluorophenyl)-2-morpholino-2-oxoethyl)dimethylsulfonium trifluoromethanesulfonate (2q)

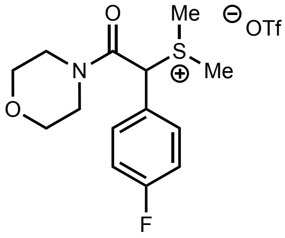

$C_{15}H_{29}F_4NO_5S_2$
MW: 433.43

Synthesised according to **GP-4** from 2-(4-fluorophenyl)-1-morpholinoethan-1-one (223 mg, 1.0 mmol), Tf_2O (0.18 mL, 1.1 mmol), and 2-chloropyridine (0.19 mL, 2.0 mmol) in CH_2Cl_2 (3.5 mL) *then* DMSO (0.18 mL in 0.5 mL CH_2Cl_2, 2.5 mmol) and TFAA (0.14 mL, 1.0 mmol). Purification by column chromatography (silica gel; 0-10% MeOH in EtOAc) afforded the product **2q** as a white solid (271 mg, 0.63 mmol, 63%).

^{1}H NMR (400 MHz, $CDCl_3$): δ 7.69 – 7.63 (m, 2H, Ar *H*), 7.23 (dd, *J* = 9.0, 8.1 Hz, 2H, Ar *H*), 6.84 (s, 1H, C*H*SMe$_2$), 3.77 (ddd, *J* = 13.3, 5.8, 3.2 Hz, 1H, C*H$_2$*), 3.70 – 3.57 (m, 4H, C*H$_2$*), 3.47 (ddd, *J* = 13.3, 7.3, 3.4 Hz, 1H, C*H$_2$*), 3.25 (ddd, *J* = 12.5, 6.0, 3.2 Hz, 1H, C*H$_2$*), 3.16 (s, 3H, SC*H$_3$*), 3.11 (ddd, *J* = 13.3, 7.6, 3.2 Hz, 1H, C*H$_2$*), 2.72 (s, 3H, SC*H$_3$*).

^{13}C{^{1}H} NMR (101 MHz, $CDCl_3$): δ 164.5 (d, *J* = 254.3 Hz, Ar *C*F), 164.3 (*C*=O), 131.9 (d, *J* = 8.7 Hz, Ar *C*H), 122.9 (d, *J* = 3.4 Hz, Ar *C*), 120.6 (q, *J* = 319.4 Hz, SO$_2$*C*F$_3$), 118.1 (d, *J* = 22.1 Hz, Ar *C*H), 66.4 (*C*H$_2$), 66.2 (*C*H$_2$), 66.1 (*C*HSMe$_2$), 46.5 (*C*H$_2$), 43.3 (*C*H$_2$), 25.9 (S*C*H$_3$), 21.9 (S*C*H$_3$).

^{19}F NMR (376 MHz, $CDCl_3$): δ -78.41 (s, 3F, O$_3$SC*F$_3$*), -106.22 (tt, *J* = 8.4, 4.4 Hz, 1F, Ar*F*).

HRMS: calcd. for $C_{14}H_{19}FNO_2S$ [M-OTf]$^+$: 284.1121; found (ESI$^+$): 284.1134.

v_{max} (neat/cm^{-1}): 854, 1028, 1111, 1151, 1230, 1256, 1446, 1465, 1508, 1632, 3025.

m.p.: (recrystallized from EtOAc) 158-160 °C.

(1-(4-Bromophenyl)-2-morpholino-2-oxoethyl)dimethylsulfonium trifluoromethanesulfonate (2r)

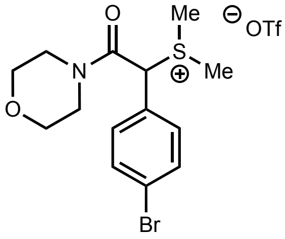

$C_{15}H_{19}BrF_3NO_5S_2$
MW: 494.34

Synthesised according to **GP-4** from 2-(4-bromophenyl)-1-morpholinoethan-1-one (xx, 1.0 mmol), Tf_2O (0.18 mL, 1.1 mmol), and 2-chloropyridine (0.19 mL, 2.0 mmol) in CH_2Cl_2 (3.5 mL) *then* DMSO (0.18 mL in 0.5 mL CH_2Cl_2, 2.5 mmol) and TFAA (0.14 mL, 1.0 mmol). Purification by column chromatography (silica gel; 0-10% MeOH in EtOAc) afforded the product **2r** as a yellow solid (430 mg, 0.87 mmol, 87%)

^{1}H NMR (400 MHz, $CDCl_3$): δ 7.78 – 7.70 (m, 2H, Ar *H*), 7.42 – 7.33 (m, 2H, Ar *H*), 6.02 – 5.83 (m, 1H, C*H*), 3.67 – 3.46 (m, 5H, 2 × CH_2, CH_{2A}), 3.38 – 3.28 (m, 1H, CH_{2B}), 3.16 – 2.98 (m, 2H, CH_2), 2.80 (s, 3H, SCH_3), 2.52 (s, 3H, SCH_3).

^{13}C{^{1}H} NMR (101 MHz, $CDCl_3$): δ 164.1 (*C*=O), 134.4 (Ar *C*), 132.7 (Ar *C*H), 126.5 (Ar *C*H), 126.4 (Ar *C*), 66.8 (*C*HSMe$_2$), 66.4 (*C*H$_2$), 66.1 (*C*H$_2$), 47.2 (*C*H$_2$), 43.8 (*C*H$_2$), 25.8 (S*C*H$_3$), 23.2 (S*C*H$_3$).

^{19}F NMR (376 MHz, $CDCl_3$): δ -79.30 (s, SO$_2$C*F*$_3$).

HRMS: calcd. for $C_{14}H_{19}BrNO_2S$ [M-OTf]$^+$: 344.0314; found (ESI$^+$): 344.0309.

v_{max} (neat/cm^{-1}): 677, 1051, 1239, 1476, 1688, 1723, 2891, 2951, 3577.

m.p.: (recrystallized from EtOAc) 135-138 °C.

(1-(4-Nitrophenyl)-2-morpholino-2-oxoethyl)dimethylsulfonium trifluoromethanesulfonate (2s)

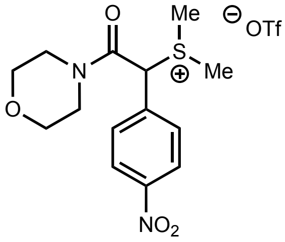

$C_{15}H_{19}F_3N_2O_7S_2$
MW: 460.44

Synthesised according to **GP-4** from 1-morpholino-2-(4-nitrophenyl)ethan-1-one (250 mg, 1.0 mmol), Tf_2O (0.18 mL, 1.1 mmol), and 2-chloropyridine (0.19 mL, 2.0 mmol) in CH_2Cl_2 (3.5 mL) *then* DMSO (0.18 mL in 0.5 mL CH_2Cl_2, 2.5 mmol) and TFAA (0.14 mL, 1.0 mmol). Purification by column chromatography (silica gel; 0-10% MeOH in EtOAc) afforded the product **2s** as an off yellow solid (337 mg, 0.76 mmol, 76%).

^{1}H NMR (400 MHz, $CDCl_3$): δ 8.40 – 8.32 (m, 2H, Ar *H*), 7.76 – 7.67 (m, 2H, Ar *H*), 6.11 (s, 1H, C*H*), 3.69 – 3.45 (m, 5H, 2 × CH_2, CH_{2A}), 3.41 – 3.31 (m, 1H, CH_{2B}), 3.15 – 2.97 (m, 2H, CH_2), 2.87 (s, 3H, SCH_3), 2.56 (s, 3H, SCH_3).

^{13}C{^{1}H} NMR (101 MHz, $CDCl_3$): δ 163.7 (*C*=O), 133.9 (Ar *C*), 132.3 (Ar *C*H), 127.2 (Ar *C*), 126.2 (Ar *C*H), 66.8 (*C*HSMe$_2$), 66.4 (*C*H$_2$), 65.4 (*C*H$_2$), 47.2 (*C*H$_2$), 43.9 (*C*H$_2$), 26.0 (S*C*H$_3$), 23.3 (S*C*H$_3$). *The quaternary carbon corresponding to the CF_3 in the triflate counter anion was not observed, though its presence was confirmed by ^{19}F NMR spectroscopy.*

^{19}F NMR (376 MHz, $CDCl_3$): δ -79.30 (s, SO_2CF_3).

HRMS: calcd. for $C_{14}H_{19}N_2O_4S$ [M-OTf]$^+$: 311.1060; found (ESI$^+$): 311.1065.

ν_{max} (neat/cm^{-1}): 641, 1028, 1251, 1463, 1665, 1714, 2877, 2931, 3550.

m.p.: (recrystallized from EtOAc) 131-133 °C.

(1-(Naphthalen-2-yl)-2-morpholino-2-oxoethyl)dimethylsulfonium trifluoromethanesulfonate (2t)

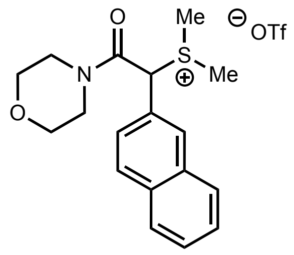

$C_{19}H_{22}F_3NO_5S_2$
MW: 465.50

Synthesised according to **GP-4** from 2-(naphthalen-2-yl)-1-morpholinoethan-1-one (95 mg, 0.37 mmol), Tf_2O (68 μL, 1.1 mmol), and 2-chloropyridine (70 μL, 2.0 mmol) in CH_2Cl_2 (1.3 mL) *then* DMSO (66 μL in 0.2 mL CH_2Cl_2, 0.93 mmol) and TFAA (0.14 mL, 1.0 mmol). Purification by column chromatography (silica gel; 0-10% MeOH in EtOAc) afforded the product **2t** as a yellow solid (102 mg, 0.22 mmol, 60%).

^{1}H NMR (400 MHz, CD_3CN): δ 8.09 (d, *J* = 8.5 Hz, 1H, Ar *H*), 8.05 – 7.98 (m, 3H, Ar *H*), 7.70 – 7.64 (m, 2H, Ar *H*), 7.48 (dd, *J* = 8.5, 2.0 Hz, 1H, Ar *H*), 6.05 (s, 1H, C*H*SMe$_2$), 3.67 – 3.49 (m, 4H, 2 x CH_2), 3.48 – 3.34 (m, 2H, 2 x CH_{2A}) 3.09 – 2.96 (m, 2H, 2 x CH_{2B}), 2.84 (s, 3H, SCH_3), 2.52 (s, 3H, SCH_3).

^{13}C{^{1}H} NMR (101 MHz, CD_3CN) δ 164.5 (*C*=O), 135.2 (Ar *C*), 134.2 (Ar *C*), 131.8 (Ar *C*H), 131.3 (Ar *C*H), 129.6 (Ar *C*H), 129.3 (Ar *C*H), 128.9 (Ar *C*H), 128.6 (Ar *C*H), 126.5 (Ar *C*H), 124.4 (Ar *C*), 122.0 (q, *J* = 320.3 Hz, *C*F$_3$), 67.4 (*C*HSMe$_2$), 66.9 (*C*H$_2$), 66.4 (*C*H$_2$), 47.3 (*C*H$_2$), 43.8 (*C*H$_2$), 25.9 (*C*H$_3$), 23.4 (*C*H$_3$). *The quaternary carbon corresponding to the CF_3 in the triflate counter anion was not observed, though its presence was confirmed by ^{19}F NMR spectroscopy.*

^{19}F NMR (376 MHz, CD_3CN): -79.33 (s, SO$_2$C*F$_3$*).

HRMS: calcd. for $C_{18}H_{22}NO_2S$ [M-OTf]$^+$: 316.1362; found (ESI$^+$): 316.1366.

ν_{max} (neat/cm^{-1}): 639, 750, 1031, 1258, 1443, 1643, 2864, 2928, 3354.

(1-Morpholino-1-oxophenylpropan-2-yl)dimethylsulfonium trifluoromethanesulfonate (2u)

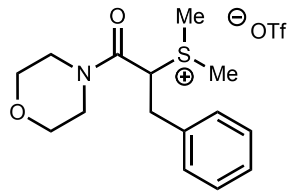

$C_{16}H_{22}F_3NO_5S_2$
MW: 429.47

Synthesised according to **GP-4** from 1-morpholinopent-4-en-1-one (219 mg, 1.0 mmol), Tf_2O (0.18 mL, 1.1 mmol), and 2-chloropyridine (0.19 mL, 2.0 mmol) in CH_2Cl_2 (3.5 mL) *then* DMSO (0.18 mL in 0.5 mL CH_2Cl_2, 2.5 mmol) and TFAA (0.14 mL, 1.0 mmol). Purification by column chromatography (silica gel; 0-10% MeOH in EtOAc) afforded the product **2u** as an off-white solid (318 mg, 0.74 mmol, 74%).

^{1}H NMR (400 MHz, CD_3CN): δ 7.42 – 7.34 (m, 3H, Ar *H*), 7.30 – 7.26 (m, 2H, Ar *H*), 4.89 (dd, J = 10.1, 4.8 Hz, 1H, C*H*SMe$_2$), 3.58 – 3.33 (m, 6H, 2 x CH_2, 2 x CH_{2A}), 3.27 – 3.19 (m, 2H, C$H_{2A,}$ CH_{2B}), 2.94 (s, 3H, CH_3), 2.87 (s, 3H, CH_3), 2.80 – 2.73 (m, 2H, CH_2).

^{13}C{^{1}H} NMR (101 MHz, CD_3CN): δ 164.9 (*C*=O), 134.8 (Ar *C*), 130.8 (Ar *C*H), 130.2 (Ar *C*H), 129.3 (Ar *C*H), 122.1 (q, J = 320.8 Hz, *C*F$_3$), 66.8 (*C*H$_2$), 66.5 (*C*H$_2$), 58.1 (*C*HSMe$_2$), 47.5 (*C*H$_2$), 43.5 (*C*H$_2$), 35.2 (*C*H$_2$), 24.9 (*C*H$_3$), 23.7 (*C*H$_3$).

^{19}F NMR (376 MHz, CD_3CN): -79.33 (s, SO$_2$CF_3)

HRMS: calcd. for $C_{15}H_{22}NO_2S$ [M-OTf]$^+$: 280.1366; found (ESI$^+$): 280.1363.

ν_{max} (neat/cm^{-1}): 634, 750, 1033, 1262, 1270, 1632, 2851, 2929, 2994, 3333.

(1-Morpholino-1-oxopent-4-en-2-yl)dimethylsulfonium trifluoromethanesulfonate (2v)

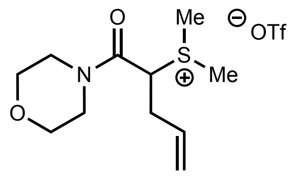

$C_{12}H_{20}F_3NO_5S_2$
MW: 379.41

Synthesised according to **GP-4** from 1-morpholinopent-4-en-1-one (169 mg, 1.0 mmol), Tf_2O (0.18 mL, 1.1 mmol), and 2-chloropyridine (0.19 mL, 2.0 mmol) in CH_2Cl_2 (3.5 mL) *then* DMSO (0.18 mL in 0.5 mL CH_2Cl_2, 2.5 mmol) and TFAA (0.14 mL, 1.0 mmol). Purification by column chromatography (silica gel; 0-2% MeOH in EtOAc) afforded the product **2v** as a colourless oil (239 mg, 0.631 mmol, 63%).

^{1}H NMR (400 MHz, $CDCl_3$): δ 5.78 (ddd, *J* = 17.0, 11.7, 7.2 Hz, 1H, $H_2C=CH$), 5.67 (t, *J* = 5.6 Hz, 1H, C*H*SMe$_2$), 5.37 – 5.31 (m, 2H, $H_2C=CH$) 3.88 – 3.69 (m, 5H, CH_2), 3.66 (ddd, *J* = 11.7, 7.7, 3.0 Hz, 1H, CH_2), 3.57 – 3.50 (m, 1H, CH_2), 3.47 (ddd, *J* = 13.4, 7.7, 3.3 Hz, 1H, CH_2), 3.05 (s, 3H, SCH_3), 3.03 (s, 3H, SCH_3), 2.87 – 2.78 (m, 2H, CH_2).

^{13}C{^{1}H} NMR (126 MHz, $CDCl_3$): δ 164.1 (*C*=O), 129.3 ($H_2C=CH$), 122.3 ($H_2C=CH$), 120.6 (q, *J* = 319.4 Hz, SO_2CF_3), 66.9 (CH_2), 66.7 (CH_2), 59.2 (*C*HSMe$_2$), 47.1 (CH_2), 43.2 (CH_2), 32.9 (CH_2), 24.5 (SCH_3), 22.1 (SCH_3).

^{19}F NMR (376 MHz, $CDCl_3$): δ -78.49 (s, SO_2CF_3).

HRMS: calcd. for $C_{11}H_{20}NO_2S$ [M-OTf]$^+$: 230.1215; found (ESI$^+$): 230.1226.

ν_{max} (neat/cm^{-1}): 932, 1027, 1108, 1152, 1222, 1256, 1438, 1468, 1633, 2925, 2964.

m.p.: (recrystallized from EtOAc) 99-100 °C.

(1-Cyclohexyl-2-morpholino-2-oxoethyl)dimethylsulfonium trifluoromethanesulfonate (2w)

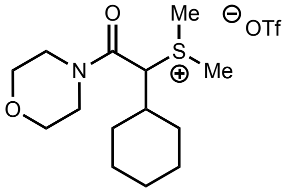

$C_{15}H_{26}F_3NO_5S_2$
MW: 421.49

Synthesised according to **GP-4** from 2-cyclohexyl-1-morpholinoethan-1-one (221 mg, 1.0 mmol), Tf_2O (0.18 mL, 1.1 mmol), and 2-chloropyridine (0.19 mL, 2.0 mmol) in CH_2Cl_2 (3.5 mL) *then* DMSO (0.18 mL in 0.5 mL CH_2Cl_2, 2.5 mmol) and TFAA (0.14 mL, 1.0 mmol). Purification by column chromatography (silica gel; 0-4% MeOH in EtOAc) afforded the product **2w** as a white solid (247 mg, 0.59 mmol, 59%).

^{1}H NMR (400 MHz, $CDCl_3$): δ 5.17 (d, *J* = 7.2 Hz, 1H, C*H*SMe$_2$), 3.96 – 3.83 (m, 2H, C*H$_2$*), 3.81 – 3.72 (m, 2H, C*H$_2$*), 3.72 – 3.52 (m, 3H, C*H$_2$*, C*H$_{2A}$*), 3.48 (ddd, *J* = 13.4, 8.0, 3.2 Hz, 1H, C*H$_{2B}$*), 3.01 (s, 3H, SC*H$_3$*), 3.00 (s, 3H, SC*H$_3$*), 2.14 – 1.55 (m, 6H, C*H*, 2 × C*H$_2$*, C*H$_{2A}$*), 1.38 – 1.07 (m, 5H, 2 × C*H$_2$*, C*H$_{2B}$*).

^{13}C{^{1}H} NMR (101 MHz, $CDCl_3$): δ 163.8 (*C*=O), 120.6 (q, *J* = 319.6 Hz, SO$_2$*C*F$_3$), 67.1 (*C*H$_2$), 67.0 (*C*H$_2$), 59.4 (*C*HSMe$_2$), 47.6 (*C*H$_2$), 43.2 (*C*H$_2$), 38.7 (*C*H), 30.4 (*C*H$_2$), 30.0 (*C*H$_2$), 25.92 (*C*H$_2$), 25.85 (*C*H$_2$), 25.4 (*C*H$_2$), 23.2 (S*C*H$_3$), 22.7 (S*C*H$_3$).

^{19}F NMR (376 MHz, $CDCl_3$): δ -78.50 (s, SO_2CF_3).

HRMS: calcd. for $C_{14}H_{26}NO_2S$ [M-OTf]$^+$: 272.1684; found (ESI$^+$): 272.1694.

ν_{max} (neat/cm^{-1}): 636, 1027, 1110, 1150, 1223, 1238, 1282, 1449, 1650, 2861, 2931.

m.p.: (recrystallized from EtOAc) 168-170 °C.

(1-Morpholino-1-oxohept-6-en-2-yl)dimethylsulfonium trifluoromethanesulfonate (2x)

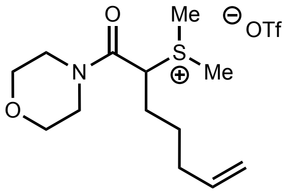

$C_{14}H_{24}F_3NO_5S_2$
MW: 407.46

Synthesised according to **GP-4** from 1-morpholinohept-6-en-1-one (197 mg, 1.0 mmol), Tf_2O (0.18 mL, 1.1 mmol), and 2-chloropyridine (0.19 mL, 2.0 mmol) in CH_2Cl_2 (3.5 mL) *then* DMSO (0.18 mL in 0.5 mL CH_2Cl_2, 2.5 mmol) and TFAA (0.14 mL, 1.0 mmol). Purification by column chromatography (silica gel; 0-5% MeOH in EtOAc) afforded the product **2x** as a white solid (227 mg, 0.558 mmol, 56%).

^{1}H NMR (400 MHz, $CDCl_3$): δ 5.79 – 5.65 (m, 1H, C*H*=CH_2), 5.57 (t, *J* = 5.5 Hz, 1H, C*H*SMe_2), 5.09 – 5.04 (m, 2H, *H_2*C=CH) 3.89 – 3.78 (m, 2H, C*H_2*), 3.78 – 3.71 (m, 3H, C*H_2*), 3.66 (ddd, *J* = 11.6, 7.8, 3.0 Hz, 1H, C*H_2*), 3.53 (ddd, *J* = 13.0, 6.3, 4.4 Hz, 1H, C*H_2*), 3.47 (ddd, *J* = 13.3, 7.8, 3.4 Hz, 1H, C*H_2*), 3.05 (s, 3H, SC*H_3*), 3.02 (s, 3H, SC*H_3*), 2.19 – 2.06 (m, 2H, C*H_2*), 2.07 – 1.97 (m, 2H, C*H_2*), 1.59 – 1.47 (m, 2H, C*H_2*).

^{13}C{^{1}H} NMR (101 MHz, $CDCl_3$): δ 164.3 (*C*=O), 136.5 (H_2C=*C*H), 120.6 (q, *J* = 319.6 Hz, SO_2*C*F_3), 117.0 (H_2*C*=CH), 67.0 (*C*H_2), 66.8 (*C*H_2), 60.2 (*C*HSMe_2), 47.1 (*C*H_2), 43.2 (*C*H_2), 32.9 (*C*H_2), 27.4 (*C*H_2), 24.7 (*C*H_3), 24.3 (*C*H_2), 22.2 (*C*H_3).

^{19}F NMR (376 MHz, $CDCl_3$): δ -78.48 (s, SO_2C*F_3*).

HRMS: calcd. for $C_{13}H_{24}NO_2S$ $[M\text{-}OTf]^+$: 258.1528; found (ESI+): 258.1541.

ν_{max} (neat/cm^{-1}): 921, 1028, 1110, 1157, 1223, 1252, 1438, 1626, 2864, 2944.

m.p.: (recrystallized from EtOAc) 121-122 °C.

(6-Chloro-1-morpholino-1-oxohexan-2-yl)dimethylsulfonium trifluoromethanesulfonate (2y)

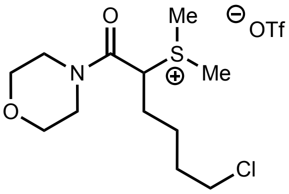

$C_{13}H_{23}ClF_3NO_5S_2$
MW: 429.89

Synthesised according to **GP-4** from 6-chloro-1-morpholinohexan-1-one (219 mg, 1.0 mmol), Tf_2O (0.18 mL, 1.1 mmol), and 2-chloropyridine (0.19 mL, 2.0 mmol) in CH_2Cl_2 (3.5 mL) *then* DMSO (0.18 mL in 0.5 mL CH_2Cl_2, 2.5 mmol) and TFAA (0.14 mL, 1.0 mmol). Purification by column chromatography (silica gel; 0-5% MeOH in EtOAc) afforded the product as a white waxy solid (346 mg, 0.74 mmol, 74%). Purification by column chromatography (silica gel; 0-10% MeOH in EtOAc) afforded the product **2y** as a white solid (292 mg, 0.68 mmol, 68%).

^{1}H NMR (400 MHz, $CDCl_3$): δ 5.61 (t, *J* = 5.5 Hz, 1H, C*H*SMe$_2$), 3.87 – 3.79 (m, 2H, C*H$_2$*), 3.79 – 3.72 (m, 3H, C*H$_2$*, C*H$_{2A}$*), 3.67 (ddd, *J* = 11.6, 7.7, 3.0 Hz, 1H, C*H$_{2B}$*), 3.62 – 3.54 (m, 3H, C*H$_2$*, C*H$_{2A}$*), 3.50 (ddd, *J* = 13.1, 7.6, 3.3 Hz, 1H, C*H$_{2B}$*), 3.06 (s, 3H, SC*H$_3$*), 3.05 (s, 3H, SC*H$_3$*), 2.12 – 2.03 (m, 2H, C*H$_2$*), 1.91 – 1.79 (m, 2H, C*H$_2$*), 1.70 – 1.56 (m, 2H, C*H$_2$*).

^{13}C NMR (101 MHz, $CDCl_3$): δ 164.2 (*C*=O), 120.6 (q, *J* = 318.5 Hz, SO$_2$*C*F$_3$), 66.9 (*C*H$_2$), 66.8 (*C*H$_2$), 59.5 (*C*HSMe$_2$), 47.1 (*C*H$_2$), 44.1 (*C*H$_2$), 43.3 (*C*H$_2$), 31.4 (*C*H$_2$), 27.3 (*C*H$_2$), 24.6 (S*C*H$_3$), 22.5 (*C*H$_2$), 22.3 (S*C*H$_3$).

^{19}F NMR (376 MHz, $CDCl_3$): δ -78.41 (s, SO$_2$C*F$_3$*).

HRMS: calcd. for $C_{12}H_{23}ClNO_2S$ [M-OTf]$^+$: 280.1133; found (ESI$^+$): 280.1131.

m.p.: 108-109 °C.

(8-Methoxy-1-morpholino-1,8-dioxooctan-2-yl)dimethylsulfonium trifluoromethanesulfonate (2z)

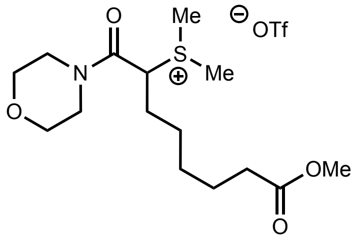

$C_{16}H_{28}F_3NO_7S_2$
MW: 467.52

Synthesised according to **GP-4** from methyl 8-morpholino-8-oxooctanoate (257 mg, 1.0 mmol), Tf_2O (0.18 mL, 1.1 mmol), and 2-chloropyridine (0.19 mL, 2.0 mmol) in CH_2Cl_2 (3.5 mL) *then* DMSO (0.18 mL in 0.5 mL CH_2Cl_2, 2.5 mmol) and TFAA (0.14 mL, 1.0 mmol). Purification by column chromatography (silica gel; 0-5% MeOH in EtOAc) afforded the product **2z** as a white waxy solid (346 mg, 0.74 mmol, 74%).

^{1}H NMR (400 MHz, $CDCl_3$): δ 5.53 (t, J = 5.6 Hz, 1H, C*H*SMe$_2$), 3.91 – 3.70 (m, 5H, C*H$_2$,* C*H$_{2A}$*), 3.70 – 3.60 (m, 4H, C*H$_{2B}$*, OC*H$_3$*), 3.58 – 3.50 (m, 1H, C*H$_{2A}$*), 3.46 (ddd, J = 13.3, 7.9, 3.3 Hz, 1H, C*H$_{2B}$*), 3.04 (s, 3H, SC*H$_3$*), 3.03 (s, 3H, SC*H$_3$*), 2.31 (t, J = 7.2 Hz, 2H, C*H$_2$*CO$_2$Me), 2.07 – 1.97 (m, 2H, C*H$_2$*), 1.63 (app pent, J = 7.2 Hz, 2H, C*H$_2$*), 1.50 – 1.33 (m, 4H, 2 × C*H$_2$*).

^{13}C NMR (101 MHz, $CDCl_3$): δ 173.9 (*C*=O), 164.3 (*C*=O), 122.2 (d, J = 319.9 Hz, SO$_2$*C*F$_3$), 67.0 (*C*H$_2$) 66.8 (*C*H$_2$), 59.6 (*C*HSMe$_2$), 51.8 (O*C*H$_3$), 47.1 (*C*H$_2$), 43.2 (*C*H$_2$), 33.7 (*C*H$_2$CO$_2$Me), 28.8 (*C*H$_2$), 28.1 (*C*H$_2$), 25.1 (*C*H$_2$), 24.6 (S*C*H$_3$), 24.3 (*C*H$_2$), 22.3 (S*C*H$_3$).

^{19}F NMR (376 MHz, $CDCl_3$): δ -78.43 (s, SO$_2$C*F$_3$*).

HRMS: calcd. for $C_{15}H_{28}NO_4S$ [M-OTf]$^+$: 318.1734; found (ESI$^+$): 318.1739.
m.p.: 44-46 °C.

5.1 General Procedure 5 (GP-5): Photochemical α-Alkylation

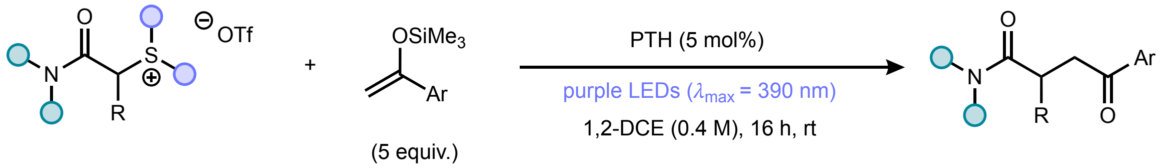

An oven-dried microwave vial was charged with sulfonium salt (0.1 mmol) and 10-phenylphenothiazine (**PTH**, 1.4 mg, 0.005 mmol) and then sealed and evacuated and back-filled with dinitrogen 3 times. Anhydrous DCE (0.25 mL, 0.4 M) was then added, followed by the silyl enol ether (0.5 mmol). The sample was stirred under constant irradiation (390 nm Kessil lamp, 34 W, 5 cm from light source) with fan cooling overnight. The crude material was purified directly by column chromatography using eluents given below to afford the pure product.

1-Morpholino-4-phenyl-2-(4-(trifluoromethyl)phenyl)butane-1,4-dione (3a)

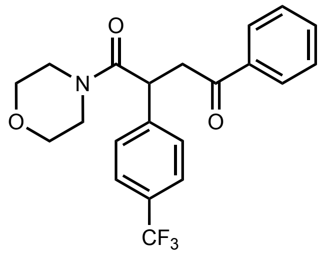

$C_{21}H_{20}F_3NO_3$
MW: 391.39

Synthesised according to **GP-5** from **2l** (48 mg, 0.1 mmol), trimethyl((1-phenylvinyl)oxy)silane (96 mg, 0.5 mmol), and PTH (1.4 mg, 0.005 mmol) in 1,2-DCE (0.25 mL). Purification by column chromatography (silica gel; 0-40% EtOAc in Hexane) afforded the product **3a** as an off yellow oil (35.5 mg, 0.091 mmol, 91%).

^{1}H NMR (400 MHz, $CDCl_3$): δ 7.97 (d, *J* = 7.2 Hz, 2H, Ar *H*), 7.62 (d, *J* = 8.1 Hz, 2H, Ar *H*), 7.56 (t, *J* = 7.4 Hz, 1H, Ar *H*), 7.49 – 7.42 (m, 4H, Ar *H*), 4.62 (dd, *J* = 9.7, 3.8 Hz, 1H, Ar C-C*H*), 4.13 (dd, *J* = 17.8, 9.7 Hz, 1H, CH_2C(O)), 3.73 – 3.60 (m, 5H, 4 × CH_2, CH_{2A}), 3.59 – 3.52 (m, 1H, CH_{2B}), 3.42 – 3.33 (m, 1H, CH_{2A}), 3.31 – 3.22 (m, 1H, CH_{2B}), 3.10 (dd, *J* = 17.9, 3.8 Hz, 1H, CH_2C(O))

^{13}C{^{1}H} NMR (101 MHz, $CDCl_3$): δ 198.1 (*C*=O), 170.3 (*C*=O), 143.5 (Ar *C*), 136.4 (Ar *C*), 133.5 (Ar *C*H), 129.9 (q, *J* = 32.7 Hz, Ar *C*-CF_3), 128.7 (Ar *C*H), 128.2-128.4 (m, CF_3-ArC- Ar *C*H- Ar *C*H), 126.3 (q, *J* = 3.7 Hz, Ar C*H*), 124.1 (q, *J* = 272.4 Hz, *C*F_3), 66.9 (*C*H_2), 66.5 (*C*H_2), 46.3 (*C*H_2), 44.2 (*C*H_2), 43.7 (*C*H), 42.8 (*C*H_2).

^{19}F NMR (471 MHz, $CDCl_3$): δ -62.57 (s, C*F$_3$*).

HRMS: calcd. for $C_{21}H_{21}F_3NO_3$ $[M+H]^+$: 392.1468; found (ESI$^+$): 392.1461.

ν_{max} (neat/cm^{-1}): 647, 1019, 1234, 1447, 1581, 1641, 1683, 2855, 2922.

1-Morpholino-4-(2-methylphenyl)-2-(4-(trifluoromethyl)phenyl)butane-1,4-dione (3b)

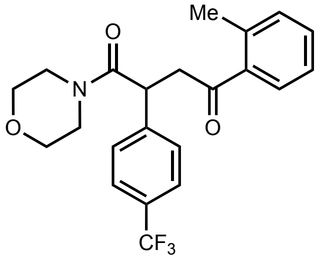

$C_{22}H_{22}F_3NO_3$
MW: 405.42

Synthesised according to **GP-5** from **2l** (48.3 mg, 0.1 mmol), ((1-(2-methylphenyl)vinyl)oxy)trimethylsilane (103 mg, 0.5 mmol), and PTH (1.4 mg, 0.005 mmol) in 1,2-DCE (0.25 mL). Purification by column chromatography (silica gel; 0-50% EtOAc in hexane) afforded the product **3b** as a yellow oil (28.4 mg, 0.070 mmol, 70%).

^{1}H NMR (400 MHz, CDCl$_3$): δ 7.80 – 7.74 (m, 1H, Ar *H*), 7.63 (d, *J* = 8.1 Hz, 2H, Ar *H*), 7.48 (d, *J* = 8.1 Hz, 2H, Ar *H*), 7.38 (td, *J* = 7.5, 1.4 Hz, 1H, Ar *H*), 7.30 – 7.23 (m, 2H, Ar *H*), 4.63 (dd, *J* = 9.9, 3.9 Hz, 1H, C*H*), 4.05 (dd, *J* = 17.7, 9.8 Hz, 1H, CH_2C(O)), 3.74 – 3.61 (m, 5H, CH_2), 3.61 – 3.53 (m, 1H, CH_2), 3.43 – 3.35 (m, 1H, CH_2), 3.29 – 3.22 (m, 1H, CH_2), 3.01 (dd, *J* = 17.7, 4.0 Hz, 1H, CH_2C(O)), 2.50 (s, 3H, CH_3).

^{13}C{^{1}H} NMR (101 MHz, CDCl$_3$): δ 201.9 (*C*=O), 170.3 (*C*=O), 143.5 (Ar *C*), 138.4 (Ar *C*), 137.4 (Ar *C*), 132.0 (Ar *C*H), 131.7 (Ar *C*H), 129.9 (q, *J* = 32.6 Hz, Ar *C*), 129.0 (Ar *C*H), 128.3 (Ar *C*H), 126.3 (q, *J* = 3.8 Hz, Ar *C*H), 125.9 (Ar *C*H), 124.0 (q, *J* = 272.0 Hz, *C*F_3), 66.9 (*C*H_2), 66.5 (*C*H_2), 46.8 (*C*H_2C(O)), 46.4 (*C*H_2), 44.1 (*C*H), 42.8 (*C*H_2), 21.4 (*C*H_3).

^{19}F NMR (376 MHz, CDCl$_3$): δ -62.58 (s, CF_3).

HRMS: calcd. for $C_{22}H_{22}F_3NO_3Na$ [M+Na]$^+$: 428.1444; found (ESI$^+$): 428.1439.

ν_{max} (neat/cm^{-1}): 749, 810, 1066, 1323, 1444, 1467, 1680, 1702, 2857.

1-Morpholino-4-(2-fluorophenyl)-2-(4-(trifluoromethyl)phenyl)butane-1,4-dione (3c)

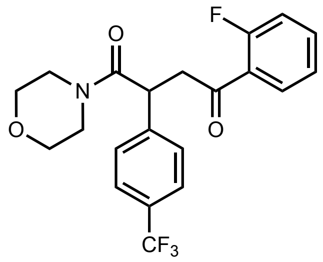

$C_{21}H_{19}F_4NO_3$
MW: 409.38

Synthesised according to **GP-5** from **2l** (48.3 mg, 0.1 mmol), ((1-(2-fluorophenyl)vinyl)oxy)trimethylsilane (105 mg, 0.5 mmol), and PTH (1.4 mg, 0.005 mmol) in 1,2-DCE (0.25 mL). Purification by column chromatography (silica gel; 0-50% EtOAc in hexane) afforded the product **3c** as a yellow oil (27.8 mg, 0.068 mmol, 68%).

^{1}H NMR (400 MHz, CDCl$_3$): δ 7.89 (td, *J* = 7.6, 1.9 Hz, 1H, Ar *H*), 7.61 (d, *J* = 8.1 Hz, 2H, Ar *H*), 7.51 (dddd, *J* = 8.6, 7.1, 5.0, 1.9 Hz, 1H, Ar *H*), 7.44 (d, *J* = 8.0 Hz, 2H, Ar *H*), 7.21 (td, *J* = 7.6, 1.1 Hz, 1H, Ar *H*), 7.11 (ddd, *J* = 11.4, 8.3, 1.0 Hz, 1H, Ar *H*), 4.59 (dd, *J* = 10.2, 3.5 Hz, 1H, C*H*), 4.01 (ddd, *J* = 18.6, 10.2, 3.2 Hz, 1H, C*H$_2$*C(O)), 3.75 – 3.59 (m, 5H, C*H$_2$*), 3.58 – 3.50 (m, 1H, C*H$_2$*), 3.41 – 3.32 (m, 1H, C*H$_2$*), 3.30 – 3.20 (m, 1H, C*H$_2$*), 3.13 (dt, *J* = 18.6, 3.4 Hz, 1H, C*H$_2$*C(O)).

^{13}C{^{1}H} NMR (101 MHz, CDCl$_3$): δ 196.2 (d, *J* = 3.9 Hz, *C*=O), 170.2 (*C*=O), 162.3 (d, *J* = 255.4 Hz, Ar *C*F), 143.3 (Ar *C*), 135.0 (d, *J* = 9.2 Hz, Ar *C*H), 130.8 (d, *J* = 2.4 Hz, Ar *C*H), 129.9 (q, J = 32.7 Hz, Ar *C*), 128.3 (Ar *C*H), 126.3 (q, *J* = 3.8 Hz, Ar *C*H), 125.0 (d, *J* = 12.4 Hz, Ar *C*), 124.6 (d, *J* = 3.4 Hz, Ar *C*H), 124.1 (q, *J* = 272.1 Hz, *C*F$_3$), 116.9 (d, *J* = 23.6 Hz, Ar *C*H), 66.9 (*C*H$_2$), 66.5 (*C*H$_2$), 48.7 (d, *J* = 8.3 Hz, *C*H$_2$C(O)), 46.3 (*C*H$_2$), 43.8 (d, *J* = 2.3 Hz, *C*H), 42.8 (*C*H$_2$).

^{19}F NMR (376 MHz, CDCl$_3$): δ -62.54 (s, 3F, C*F$_3$*), -108.44 – -108.54 (m, 1F, Ar C*F*).

HRMS: calcd. for $C_{21}H_{19}F_4NO_3Na$ [M+Na]$^+$: 432.1193; found (ESI$^+$): 431.1199.

ν_{max} (neat/cm^{-1}): 733, 817, 1113, 1235, 1608, 1643, 1727, 2856, 2920.

4-(4-Fluorophenyl)-1-morpholino-2-((4-trifluoromethyl)phenyl)butane-1,4-dione (3d)

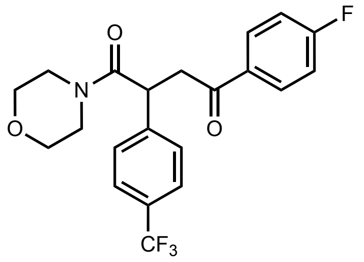

$C_{21}H_{19}F_4NO_3$
MW: 409.38

Synthesised according to **GP-5** from **2l** (48.3 mg, 0.1 mmol), ((1-(4-fluorophenyl)vinyl)oxy)trimethylsilane (105 mg, 0.5 mmol), and PTH (1.4 mg, 0.005 mmol) in 1,2-DCE (0.25 mL). Purification by column chromatography (silica gel; 0-0% EtOAc in hexane) afforded the product **3d** as a pale yellow oil (31.8 mg, 0.08 mmol, 78%).

^{1}H NMR (400 MHz, $CDCl_3$): δ 8.00 (dd, *J* = 8.9, 5.4 Hz, 2H, Ar *H*), 7.61 (d, *J* = 8.1 Hz, 2H, Ar *H*), 7.46 (d, *J* = 8.1 Hz, 2H, Ar *H*), 7.11 (app t, *J* = 8.6 Hz, 2H, Ar *H*), 4.60 (dd, *J* = 9.8, 3.8 Hz, 1H, C*H*), 4.10 (dd, *J* = 17.8, 9.8 Hz, 1H, CH_2C(O)), 3.73 – 3.50 (m, 6H, 2 x CH_2, 2 x CH_{2A}), 3.40 – 3.32 (m, 1H, CH_{2B}), 3.29 – 3.21 (m, 1H, CH_{2B}), 3.04 (dd, *J* = 17.8, 3.8 Hz, 1H, CH_2C(O)).

^{13}C{^{1}H} NMR (101 MHz, $CDCl_3$): δ 196.5 (*C*=O), 170.2 (*C*=O), 166.1 (d, *J* = 255.1 Hz, Ar *C*F), 143.3 (Ar *C*), 132.9 (d, *J* = 3.0 Hz, Ar *C*), 131.0 (d, *J* = 9.3 Hz, Ar *C*H), 130.0 (q, *J* = 32.7 Hz, Ar *C*), 128.2 (Ar *C*H), 126.3 (q, *J* = 3.8 Hz, Ar *C*H), 124.1 (q, *J* = 272.1 Hz, *C*F_3), 115.9 (d, *J* = 21.9 Hz, Ar *C*H), 66.9 (*C*H_2), 66.5 (*C*H_2), 46.3 (*C*H_2), 44.0 (*C*H_2), 43.8 (*C*H), 42.8 (*C*H_2).

^{19}F NMR (376 MHz, $CDCl_3$): δ -62.59 (s, 3F, CF_3), -104.65 - -104.72 (m, 1F, Ar *F*).

HRMS: calcd. for $C_{20}H_{20}FNO_3Na$ $[M+Na]^+$: 432.1193; found (ESI^+): 432.1214.

***ν*$_{max}$ (neat/cm^{-1}):** 2923, 2855, 1724, 1644, 1324, 1267, 1238, 1115, 731.

1-Morpholino-2,4-di(4-(trifluoromethyl)phenyl)butane-1,4-dione (3e)

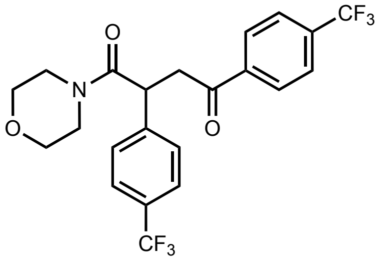

$C_{22}H_{19}F_6NO_3$
MW: 459.39

Synthesised according to **GP-5** from **2l** (48.3 mg, 0.1 mmol), trimethyl((1-(4-(trifluoromethyl)phenyl)vinyl)oxy)silane (130 mg, 0.5 mmol), and PTH (1.4 mg, 0.005 mmol) in 1,2-DCE (0.25 mL). Purification by column chromatography (silica gel; 0-40% EtOAc in hexane) afforded the product **3e** as an off yellow oil (39 mg, 0.085 mmol, 85%).

^{1}H NMR (400 MHz, CDCl$_3$): δ 8.08 (d, *J* = 8.1 Hz, 2H, Ar *H*), 7.72 (d, *J* = 8.1 Hz, 2H, Ar *H*), 7.63 (d, *J* = 8.1 Hz, 2H, Ar *H*), 7.47 (d, *J* = 8.1 Hz, 2H, Ar *H*), 4.62 (dd, *J* = 9.9, 3.8 Hz, 1H, C*H*), 4.14 (dd, *J* = 17.8, 9.9 Hz, 1H, CH_2C(O)), 3.75 – 3.53 (m, 6H, 3 × CH_2), 3.42 – 3.31 (m, 1H, CH_{2A}), 3.29 – 3.21 (m, 1H, CH_{2B}), 3.06 (dd, J = 17.8, 3.8 Hz, 1H, CH_2C(O)).

^{13}C{^{1}H} NMR (101 MHz, CDCl$_3$): δ 197.3 (CH*C*=O), 170.0 (CH$_2$*C*=O), 143.1 (Ar *C*), 139.1, (Ar *C*), 134.8 (q, *J* = 32.7 Hz, Ar *C*), 130.1 (q, *J* = 32.6 Hz, Ar *C*), 128.7 (Ar *C*H), 128.2 (Ar *C*H), 126.4 (q, *J* = 3.7 Hz, Ar *C*H), 125.9 (q, *J* = 3.7 Hz, Ar *C*H), 124.00 (q, *J* = 272.1 Hz, *C*F$_3$), 123.7 (q, *J* = 272.9 Hz, *C*F$_3$), 66.9 (*C*H$_2$), 66.5 (*C*H$_2$), 46.3 (*C*H$_2$), 44.4 (*C*H$_2$C(O)), 44.0 (*C*H), 42.9 (*C*H$_2$).

^{19}F NMR (376 MHz, CDCl$_3$): δ -62.62 (s, 3F, CF_3), -63.16 (s, 3F, CF_3).

HRMS: calcd. for $C_{22}H_{20}F_6NO_3$ [M+H]$^+$: 460.1342; found (ESI$^+$): 460.1348.

ν_{max} (neat/cm^{-1}): 633, 1018, 1241, 1442, 1493, 1645, 1722, 2852, 2918, 3275.

1-Morpholino-4-(4-bromophenyl)-2-(4-(trifluoromethyl)phenyl)butane-1,4-dione (3f)

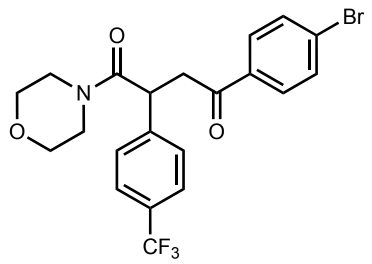

$C_{21}H_{19}BrF_3NO_3$
MW: 470.29

Synthesised according to **GP-5** from **2l** (48.3 mg, 0.1 mmol), ((1-(4-bromophenyl)vinyl)oxy)trimethylsilane (135 mg, 0.5 mmol), and PTH (1.4 mg, 0.005 mmol) in 1,2-DCE (0.25 mL). Purification by column chromatography (silica gel; 0-50% EtOAc in hexanes) afforded the product **3f** as an off yellow oil (30.4 mg, 0.065 mmol, 65%).

^{1}H NMR (400 MHz, $CDCl_3$): δ 7.89 – 7.79 (m, 2H, Ar *H*), 7.65 – 7.55 (m, 4H, Ar *H*), 7.46 (d, *J* = 8.0 Hz, 2H, Ar *H*), 4.59 (dd, *J* = 9.8, 3.7 Hz, 1H, Ar C-C*H*), 4.08 (dd, *J* = 17.8, 9.8 Hz, 1H, $CH_2C(O)$), 3.73 – 3.51 (m, 6H, 3 × CH_2), 3.41 – 3.31 (m, 1H, CH_{2A}), 3.30 – 3.21 (m, 1H, CH_{2B}), 3.02 (dd, *J* = 17.8, 3.7 Hz, 1H, $CH_2C(O)$).

^{13}C{^{1}H} NMR (101 MHz, $CDCl_3$): δ 197.1 (*C*=O), 170.1 (*C*=O), 143.3 (Ar *C*), 135.2 (Ar *C*), 132.1 (Ar *C*H), 130.0 (d, *J* = 32.8 Hz, CF_3-Ar *C*), 129.8 (Ar *C*H), 128.8 (Ar *C*), 128.2 (Ar *C*H), 126.3 (q, *J* = 3.7 Hz, Ar *C*H), 124.1 (q, *J* = 272.3 Hz, CF_3), 66.9 (CH_2) 66.5 (CH_2), 46.3 (CH_2), 44.1 ($CH_2C(O)$), 43.8 (*C*H), 42.9 (CH_2).

^{19}F NMR (376 MHz, $CDCl_3$): δ -62.60 (s, CF_3).

HRMS: calcd. for $C_{21}H_{20}BrF_3NO_3$ $[M+H]^+$: 470.0573; found (ESI$^+$): 470.0569.

ν_{max} (neat/cm^{-1}): 614, 1018, 1235, 1436, 1484, 1643, 1685, 2856, 2920.

1-Morpholino-4-(4-methylphenyl)-2-(4-(trifluoromethyl)phenyl)butane-1,4-dione (3g)

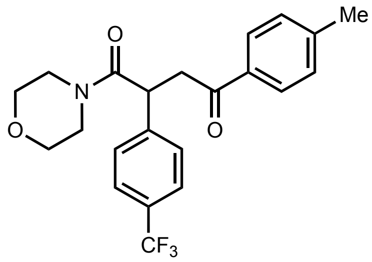

$C_{22}H_{22}F_3NO_3$
MW: 405.42

Synthesised according to **GP-5** from **2l** (48.3 mg, 0.1 mmol), ((1-(4-methylphenyl)vinyl)oxy)trimethylsilane (135 mg, 0.5 mmol), and PTH (1.4 mg, 0.005 mmol) in 1,2-DCE (0.25 mL). Purification by column chromatography (silica gel; 0-50% EtOAc in hexanes) afforded the product **3g** as an off yellow oil (22.3 mg, 0.055 mmol, 55%).

^{1}H NMR (400 MHz, $CDCl_3$): δ 7.86 (d, *J* = 8.2 Hz, 2H, Ar *H*), 7.61 (d, *J* = 8.1 Hz, 2H, Ar *H*), 7.47 (d, *J* = 8.1 Hz, 2H, Ar *H*), 7.23 (d, *J* = 8.0 Hz, 2H, Ar *H*), 4.61 (dd, *J* = 9.6, 3.8 Hz, 1H, C*H*), 4.10 (dd, *J* = 17.8, 9.7 Hz, 1H, $CH_2C(O)$), 3.77 – 3.50 (m, 6H, 3 × CH_2), 3.45 – 3.22 (m, 2H, CH_2), 3.08 (dd, *J* = 17.8, 3.8 Hz, 1H, $CH_2C(O)$), 2.39 (s, 3H, CH_3).

^{13}C{^{1}H} NMR (101 MHz, $CDCl_3$): δ 198.0 (*C*=O), 170.7 (*C*=O), 144.7 (Ar *C*), 143.9 (Ar *C*), 134.3 (Ar *C*), 130.3 (q, J = 32.7 Hz, Ar *C*), 129.7 (Ar *C*H), 128.7 (Ar *C*H), 128.6 (Ar *C*H), 126.6 (q, *J* = 3.7 Hz, Ar *C*H), 124.4 (q, *J* = 272.0 Hz, CF_3), 67.2 (CH_2), 66.8 (CH_2), 46.7 (CH_2), 44.3 ($CH_2C(O)$), 44.0 (*C*H), 43.1 (CH_2), 22.1 (CH_3).

^{19}F NMR (376 MHz, $CDCl_3$): δ -62.56 (s, CF_3).

HRMS: calcd. for $C_{22}H_{22}F_3NO_3Na$ $[M+Na]^+$: 428.1444; found (ESI^+): 427.1440.

ν_{max} (neat/cm^{-1}): 753, 818, 1068, 1112, 1323, 1436, 1458, 1643, 1680, 2856.

Methyl 3-(4-morpholino-4-oxo-3-(4-(trifluoromethyl)phenyl)butanoyl)benzoate (3h)

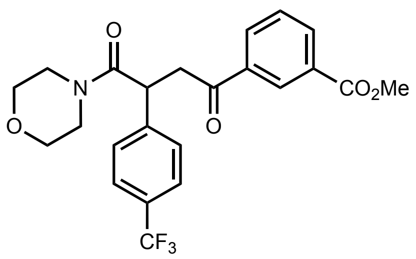

$C_{23}H_{22}F_3NO_5$
MW: 449.43

Synthesised according to **GP-5** from **2l** (48.3 mg, 0.1 mmol), methyl 3-(1-((trimethylsilyl)oxy)vinyl)benzoate (125 mg, 0.5 mmol), and PTH (1.4 mg, 0.005 mmol) in 1,2-DCE (0.25 mL). Purification by column chromatography (silica gel; 0-50% EtOAc in hexanes) afforded the product **3h** as a pale yellow oil (31.0 mg, 0.070 mmol, 70%).

^{1}H NMR (400 MHz, CDCl$_3$): δ 8.60 (d, *J* = 1.9 Hz, 1H, Ar *H*), 8.22 (d, *J* = 7.5 Hz, 1H, Ar *H*), 8.16 (d, *J* = 7.9 Hz, 1H, Ar *H*), 7.63 (d, *J* = 7.9 Hz, 2H, Ar *H*), 7.54 (app t, *J* = 7.8 Hz, 1H, Ar *H*), 7.47 (d, *J* = 7.9 Hz, 2H, Ar *H*), 4.62 (dd, *J* = 9.8, 3.8 Hz, 1H, C*H*), 4.16 (dd, *J* = 17.9, 9.8 Hz, 1H, CH_2C(O)), 3.93 (s, 3H, CH_3), 3.74 – 3.50 (m, 6H, CH_2), 3.42 – 3.21 (m, 2H, CH_2), 3.11 (dd, *J* = 17.9, 3.8 Hz, 1H, CH_2C(O)).

^{13}C{^{1}H} NMR (101 MHz, CDCl$_3$): δ 197.3 (*C*=O), 170.1 (*C*=O), 166.3 (*C*=O), 143.2 (Ar *C*), 136.6 (Ar *C*), 134.3 (Ar *C*H), 132.4 (Ar *C*H), 130.9 (Ar *C*), 130.0 (q, *J* = 32.7 Hz, Ar *C*), 129.5 (Ar *C*H), 129.0 (Ar *C*H), 128.3 (Ar *C*H), 126.3 (q, *J* = 3.8 Hz, Ar *C*H), 124.0 (q, *J* = 272.0 Hz, *C*F$_3$), 66.9 (*C*H$_2$), 66.5 (*C*H$_2$), 52.5 (*C*H$_3$), 46.3 (*C*H$_2$), 44.2 (*C*H$_2$C(O)), 43.8 (*C*H), 42.9 (*C*H$_2$).

^{19}F NMR (376 MHz, CDCl$_3$): δ -62.59 (s, CF_3).

HRMS: calcd. for $C_{23}H_{22}F_3NO_5Na$ [M+Na]$^+$: 472.1342; found (ESI$^+$): 472.1350.

ν_{max} (neat/cm^{-1}): 764, 815, 1073, 1126, 1320, 1439, 1615, 1692, 1725, 2856.

1-Morpholino-4-(thiophen-3-yl)-2-(4-(trifluoromethyl)phenyl)butane-1,4-dione (3i)

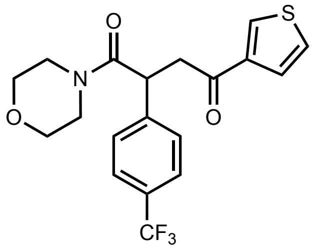

$C_{19}H_{18}F_3NO_3S$
MW: 391.41

Synthesised according to **GP-5** from **2l** (48.3 mg, 0.1 mmol), trimethyl((1-(thiophen-3-yl)vinyl)oxy)silane (99 mg, 0.5 mmol), and PTH (1.4 mg, 0.005 mmol) in 1,2-DCE (0.25 mL). Purification by column chromatography (silica gel; 0-50% EtOAc in hexanes) afforded the product **3i** as a brown oil (32.1 mg, 0.081 mmol, 81%).

^{1}H NMR (400 MHz, $CDCl_3$): δ 8.09 (dd, *J* = 2.9, 1.2 Hz, 1H, Ar *H*), 7.61 (d, *J* = 8.1 Hz, 2H, Ar *H*), 7.53 (dd, *J* = 5.1, 1.3 Hz, 1H, Ar *H*), 7.46 (d, *J* = 8.1 Hz, 2H, Ar *H*), 7.30 (dd, *J* = 5.1, 2.9 Hz, 1H, Ar *H*), 4.59 (dd, *J* = 9.6, 4.0 Hz, 1H, C*H*), 4.01 (dd, *J* = 17.6, 9.6 Hz, 1H, $CH_2C(O)$), 3.72 – 3.51 (m, 6H, 3 × CH_2), 3.40 – 3.21 (m, 2H, CH_2), 3.04 (dd, *J* = 17.6, 4.0 Hz, 1H, $CH_2C(O)$).

^{13}C{^{1}H} NMR (101 MHz, $CDCl_3$): δ 192.3 (*C*=O), 170.2 (*C*=O), 143.4 (Ar *C*), 141.6 (Ar *C*), 132.6 (Ar *C*H), 129.9 (q, *J* = 32.5 Hz, Ar *C*), 128.3 (Ar *C*H), 126.9 (Ar *C*H), 126.6 (Ar *C*H), 126.3 (q, *J* = 3.7 Hz, Ar *C*H), 124.1 (q, *J* = 272.0 Hz, CF_3), 66.9 (CH_2), 66.5 (CH_2), 46.3 (CH_2), 45.1 ($CH_2C(O)$), 43.5 (*C*H), 42.8 (CH_2).

^{19}F NMR (376 MHz, $CDCl_3$): δ -62.57 (s, CF_3).

HRMS: calcd. for $C_{19}H_{18}F_3NO_3Na$ $[M+Na]^+$: 420.0852; found (ESI^+): 420.0846.

ν_{max} (neat/cm^{-1}): 736, 842, 925, 1116, 1409, 1597, 1612, 1683, 2857.

1-Morpholino-4-(pyridin-3-yl)-2-(4-(trifluoromethyl)phenyl)butane-1,4-dione (3j)

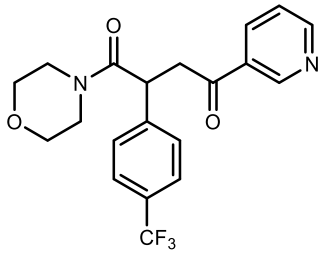

$C_{20}H_{19}F_3N_2O_3$
MW: 392.38

Synthesised according to **GP-5** from **2l** (48.3 mg, 0.1 mmol), 2-(1-((trimethylsilyl)oxy)vinyl)pyridine (96 mg, 0.5 mmol), and PTH (1.4 mg, 0.005 mmol) in 1,2-DCE (0.25 mL). Purification by column chromatography (silica gel; 0-50% EtOAc in hexanes) afforded the product as an off yellow oil (18.1 mg, 0.031 mmol, 31%).

^{1}H NMR (400 MHz, $CDCl_3$): δ 9.19 (dd, *J* = 2.3, 0.9 Hz, 1H, Ar H), 8.78 (dd, *J* = 4.9, 1.7 Hz, 1H, Ar H), 8.24 (app dt, *J* = 8.0, 2.0 Hz, 1H, Ar H), 7.64 (d, *J* = 8.1 Hz, 2H, Ar H), 7.47 (d, *J* = 8.1 Hz, 2H, Ar H), 7.41 (dd, *J* = 8.0, 4.8 Hz, 1H, Ar H), 4.62 (dd, *J* = 9.9, 3.7 Hz, 1H, CH), 4.13 (dd, *J* = 17.8, 9.9 Hz, 1H, $CH_2C(O)$), 3.73 – 3.51 (m, 6H, CH_2), 3.40 – 3.30 (m, 1H, CH_2), 3.29 – 3.22 (m, 1H, CH_2), 3.06 (dd, *J* = 17.8, 3.7 Hz, 1H, $CH_2C(O)$).

$^{13}C\{^1H\}$ NMR (101 MHz, $CDCl_3$): δ 197.1 (*C*=O), 170.0 (*C*=O), 153.9 (Ar *C*H), 149.9 (Ar *C*H), 143.0 (Ar *C*) 135.6 (Ar *C*H), 131.8 (Ar *C*), 130.2 (q, *J* = 32.6 Hz, Ar *C*), 128.2 (Ar *C*H), 126.8 (q, *J* = 283.7 Hz, CF_3), 126.4 (q, *J* = 4.0 Hz, Ar *C*H), 123.7 (Ar *C*H), 66.9 (CH_2), 66.5 (CH_2), 46.3 (CH_2), 44.3 ($CH_2C(O)$), 43.8 (CH_2), 42.9 (CH_2).

^{19}F NMR (376 MHz, $CDCl_3$): δ -62.62 (s, 3F, CF_3)

HRMS: calcd. for $C_{20}H_{20}F_3N_2O_3$ $[M+H]^+$: 393.1421; found (ESI^+): 393.1426.

ν_{max} (neat/cm^{-1}): 677, 1042, 1249, 1455, 1491, 1642, 1671, 1699, 2855, 2921.

1-Morpholino-4-(2-oxopyrrolidin-1-yl)-2-(4-(trifluoromethyl)phenyl)butane-1,4-dione (3k) and benzyl 4-morpholino-4-oxo-3-(4-(trifluoromethyl)phenyl)butanoate (3k′)

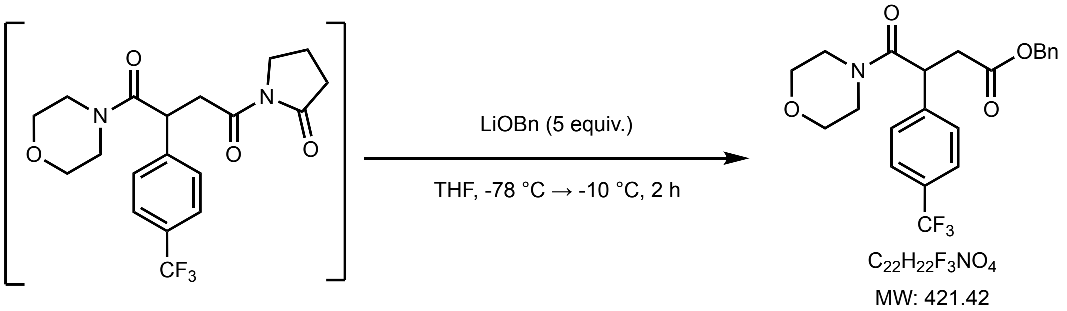

3k synthesised according to **GP-5** from **2l** (48 mg, 0.1 mmol), 1-(1-((trimethylsilyl)oxy)vinyl)pyrrolidin-2-one (100 mg, 0.5 mmol), and PTH (1.4 mg, 0.005 mmol) in 1,2-DCE (0.25 mL). Quantitative ^{1}H NMR spectroscopic analysis afforded the product **3k** in a 94% yield. The solvent was removed *in vacuo* and anhydrous THF (1 mL) was added. In a separate flask, a solution of benzyl alcohol (62 μL, 0.6 mmol) in anhydrous THF (1 mL) was cooled to -78 °C (dry ice/acetone) and *n*-BuLi (2.5 M in hexanes, 0.2 mL, 0.5 mmol) was added drop-wise. The reaction was stirred for 10 min and then the contents of the first flask were added to the LiOBn/BnOH solution drop-wise. The reaction was stirred for 1 h, then warmed to -10 °C and stirred for an additional 1 h. The reaction was quenched by the addition of water (1 mL) and brine (1 mL), and EtOAc (5 mL) was added. The organic layer was separated and the aqueous layer extracted with EtOAc (3 × 5 mL). The combined organics were dried over $MgSO_4$, filtered, and concentrated *in vacuo*. Purification by column chromatography (silica gel; 20-40% EtOAc in hexane) afforded the product **3k′** as a colourless oil (17.6 mg, 0.042 mmol, 42%).

^{1}H NMR (400 MHz, $CDCl_3$): δ 7.57 (d, *J* = 8.0 Hz, 2H, Ar *H*), 7.40 – 7.27 (m, 7H, Ar *H*), 5.16 (d, *J* = 12.3 Hz, 1H, PhCH_{2A}), 5.05 (d, *J* = 12.3 Hz, 1H, PhCH_{2B}), 4.34 (dd, *J* = 9.4, 5.1 Hz, 1H), 3.72 – 3.41 (m, 6H, 3 × CH_2), 3.32 (dd, *J* = 16.9, 9.4 Hz, 1H, CH_2C(O)), 3.28 – 3.22 (m, 1H, CH_{2A}), 3.21 – 3.13 (m, 1H, CH_{2B}), 2.64 (dd, *J* = 16.9, 5.1 Hz, 1H, CH_2C(O)).

^{13}C{^{1}H} NMR (101 MHz, $CDCl_3$): δ 171.7 (*C*=O), 169.9 (*C*=O), 142.58 (Ar *C*), 135.78 (Ar *C*), 130.1 (q, *J* = 32.5 Hz, Ar *C*), 128.7 (Ar *C*H), 128.5 (Ar *C*H), 128.3 (Ar *C*H), 128.2 (Ar *C*H), 126.3 (q, *J* = 3.9 Hz, Ar *C*H), 124.0 (q, *J* = 272.0 Hz, CF_3), 66.83 (*C*H_2), 66.78 (Ph*C*H_2), 66.4 (*C*H_2), 46.2 (*C*H_2), 44.6 (*C*H_2), 42.8 (*C*H), 39.4 (CH_2C(O)).

^{19}F NMR (471 MHz, $CDCl_3$): δ -62.63 (s, CF_3).

HRMS: calcd. for $C_{22}H_{22}F_3NO_4Na$ $[M+Na]^+$: 444.1393; found (ESI^+): 444.1415.

4-(4-Fluorophenyl)-1-morpholino-2-phenylbutane-1,4-dione (3l)

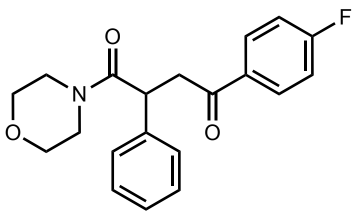

$C_{20}H_{20}FNO_3$
MW: 341.38

Synthesised according to **GP-5** from **2a** (41.5 mg, 0.1 mmol), ((1-(4-fluorophenyl)vinyl)oxy)trimethylsilane (105 mg, 0.5 mmol), and PTH (1.4 mg, 0.005 mmol) in 1,2-DCE (0.2 mL). Purification by column chromatography (silica gel; 0-50% EtOAc in hexanes) afforded the product **3l** as a pale yellow oil (24.6 mg, 0.07 mmol, 72%).

^{1}H NMR (400 MHz, CDCl$_3$): δ 8.04 – 7.98 (m, 2H, Ar *H*), 7.38 – 7.25 (m, 5H, Ar *H*), 7.10 (t, *J* = 8.6 Hz, 2H, Ar *H*), 4.52 (dd, *J* = 10.0, 3.6 Hz, 1H, C*H*), 4.10 (dd, *J* = 17.6, 10.0 Hz, 1H, C*H*$_2$C(O)), 3.76 – 3.49 (m, 6H, 2 x C*H*$_2$, 2 x C*H*$_{2A}$), 3.43 – 3.36 (m, 1H, C*H*$_{2B}$), 3.19 – 3.12 (m, 1H, C*H*$_{2B}$), 3.01 (dd, *J* = 17.6, 3.6 Hz, 1H, C*H*$_2$C(O)).

^{13}C{^{1}H} NMR (101 MHz, CDCl$_3$) δ 197.1 (*C*=O), 170.8 (*C*=O), 165.9 (d, *J* = 254.8 Hz, Ar *C*F), 139.3 (Ar *C*), 133.2 (d, *J* = 2.9 Hz, Ar *C*), 131.0 (d, *J* = 9.5 Hz, Ar *C*H), 129.4 (Ar *C*H), 127.8 (Ar *C*H), 127.6 (Ar *C*H), 115.7 (d, *J* = 21.9 Hz, Ar *C*H), 66.9 (*C*H$_2$), 66.4 (*C*H$_2$), 46.3 (*C*H$_2$), 44.3 (*C*H), 43.2 (*C*H$_2$), 42.8 (*C*H$_2$).

^{19}F NMR (376 MHz, CDCl$_3$): δ -105.11 - -105.20 (m).

HRMS: calcd. for $C_{20}H_{20}FNO_3Na$ [M+Na]$^+$: 364.1319; found (ESI$^+$): 364.1336.

ν_{max} (neat/cm^{-1}): 731, 839, 1115, 1234, 1598, 1643, 1724, 2855, 2924.

2-Benzyl-4-(4-fluorophenyl)-1-morpholinobutane-1,4-dione (3m)

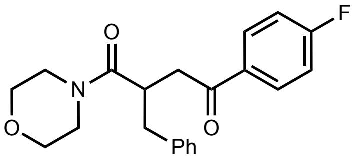

$C_{21}H_{22}FNO_3$
MW: 355.41

Synthesised according to **GP-5** from **2u** (43.0 mg, 0.1 mmol), ((1-(4-fluorophenyl)vinyl)oxy)trimethylsilane (105 mg, 0.5 mmol), and PTH (1.4 mg, 0.005 mmol) in 1,2-DCE (0.2 mL). Purification by column chromatography (silica gel; 0-50% EtOAc in hexanes) afforded the product **3m** as a colourless oil (24.5 mg, 0.07 mmol, 69%).

^{1}H NMR (400 MHz, CDCl$_3$): δ 7.98 (dd, *J* = 8.9, 5.4 Hz, 2H, Ar *H*), 7.34 – 7.19 (m, 5H, Ar *H*), 7.12 (t, *J* = 8.6 Hz, 2H, Ar *H*), 3.75 (dd, *J* = 17.8, 9.6 Hz, 1H, CH_2C(O)), 3.65 – 3.54 (m, 4H, 3 x CH_{2A}, C*H*), 3.53 – 3.34 (m, 3H, CH_{2A}, 2 x CH_{2B}), 3.19 – 3.12 (m, 1H, CH_{2B}), 3.03 (dd, *J* = 17.8, 3.5 Hz, 1H, CH_2C(O)), 2.96 (dd, *J* = 13.1, 9.7 Hz, 1H, PhCH_2), 2.95 – 2.88 (m, 1H, CH_{2B}), 2.82 (dd, *J* = 13.1, 5.9 Hz, 1H, PhCH_2).

^{13}C{^{1}H} NMR (101 MHz, CDCl$_3$): δ 197.3 (*C*=O), 173.2 (*C*=O), 166.0 (d, *J* = 254.9 Hz, Ar *C*F), 138.9 (Ar *C*), 133.1 (d, *J* = 3.0 Hz, Ar *C*), 130.8 (d, *J* = 9.3 Hz, Ar *C*H), 129.2 (Ar *C*H), 128.7 (Ar *C*H), 127.0 (Ar *C*H), 115.8 (d, *J* = 21.8 Hz, Ar *C*H), 66.7 (*C*H$_2$), 66.4 (*C*H$_2$), 46.4 (*C*H$_2$), 42.3 (*C*H$_2$), 42.1 (*C*H$_2$), 39.5 (*C*H$_2$), 38.2 (*C*H).

^{19}F NMR (376 MHz, CDCl$_3$): -104.88 - -104.97 (m).

HRMS: calcd. for $C_{21}H_{22}FNO_3Na$ $[M+Na]^+$: 378.1476; found (ESI$^+$): 378.1491.

ν_{max} (neat/cm^{-1}): 702, 839, 1115, 1231, 1410, 1598, 1633, 1683, 2857, 2919, 2962.

2-(4-Fluorophenyl)-1-morpholino-4-phenylbutane-1,4-dione (3n)

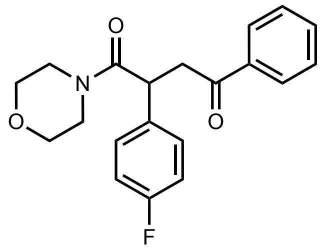

$C_{20}H_{20}FNO_3$
MW: 341.38

Synthesised according to **GP-5** from **2q** (43.4 mg, 0.1 mmol), trimethyl((1-phenylvinyl)oxy)silane (96 mg, 0.5 mmol), and PTH (1.4 mg, 0.005 mmol) in 1,2-DCE (0.2 mL). Purification by column chromatography (silica gel; 0-50% EtOAc in hexanes) afforded the product **3n** as a pale yellow oil (11.6 mg, 0.034 mmol, 34%).

^{1}H NMR (400 MHz, $CDCl_3$): δ 8.01 – 7.94 (m, 2H, Ar *H*), 7.59 – 7.50 (m, 1H, Ar *H*), 7.47 - 7.40 (m, 2H, Ar *H*), 7.36 – 7.28 (m, 2H, Ar *H*), 7.09 – 7.00 (m, 2H, Ar *H*), 4.53 (dd, J = 9.7, 3.9 Hz, 1H, C*H*), 4.10 (dd, J = 17.8, 9.7 Hz, 1H, CH_2C(O)), 3.80 – 3.48 (m, 6H, 3 × CH_2), 3.46 – 3.17 (m, 2H, CH_2), 3.07 (dd, J = 17.8, 3.9 Hz, 1H, CH_2C(O)).

^{13}C{^{1}H} NMR (101 MHz, $CDCl_3$): δ 198.5 (*C*=O), 170.9 (*C*=O), 162.1 (d, *J* = 246.4 Hz, Ar *C*F), 136.6 (Ar *C*), 135.1 (d, *J* = 3.3 Hz, Ar *C*), 133.4 (Ar *C*H), 129.4 (d, *J* = 7.9 Hz, Ar *C*H), 128.7 (Ar *C*H), 128.3 (Ar *C*H), 116.2 (d, *J* = 21.5 Hz, Ar *C*H), 66.9 (CH_2), 66.5 (CH_2), 46.3 (CH_2), 44.4 (CH_2C(O)), 43.2 (*C*H), 42.8 (CH_2).

^{19}F NMR (376 MHz, $CDCl_3$): δ -114.77 – -114.87 (m).

HRMS: calcd. for $C_{20}H_{20}FNO_3Na$ [M+Na]$^+$: 364.1319; found (ESI$^+$): 364.1325.

ν_{max} (neat/cm^{-1}): 690, 785, 841, 1029, 1113, 1224, 1507, 1637, 1682, 2855, 2919.

2-Cyclohexyl-1-morpholino-4-phenylbutane-1,4-dione (3o)

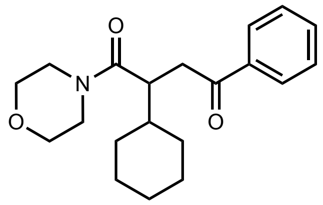

$C_{20}H_{27}FNO_3$
MW: 329.44

Synthesised according to **GP-5** from **2w** (42.2 mg, 0.1 mmol), trimethyl((1-phenylvinyl)oxy)silane (96 mg, 0.5 mmol), and PTH (1.4 mg, 0.005 mmol) in 1,2-DCE (0.2 mL). Purification by column chromatography (silica gel; 0-50% EtOAc in hexanes) afforded the product **3o** as a pale yellow oil (11.5 mg, 0.035 mmol, 35%).

^{1}H NMR (400 MHz, $CDCl_3$): δ 7.96 (d, J = 7.4 Hz, 2H, Ar H), 7.54 (t, J = 7.3 Hz, 1H, Ar H), 7.44 (app t, J = 7.6 Hz, 2H, Ar H), 3.91 – 3.78 (m, 2H, CH_2), 3.78 – 3.59 (m, 6H, 3 × CH_2), 3.55 (dt, J = 12.9, 4.9 Hz, 1H, CH_{2A}), 3.16 (ddd, J = 10.3, 7.6, 3.1 Hz, 1H, CH), 3.06 (dd, J = 17.8, 3.0 Hz, 1H, CH_2C(O)), 1.88 – 1.54 (m, 6H, 3 x CH_2), 1.33 – 0.95 (m, 5H, 1 x CH, 2 x CH_2).

^{13}C{^{1}H} NMR (101 MHz, $CDCl_3$): δ 199.8 (C=O), 174.0 (C=O), 136.9 (Ar C), 133.3 (Ar CH), 128.6 (Ar CH), 128.2 (Ar CH), 67.2 (CH_2), 66.9 (CH_2), 47.0 (CH_2), 42.4 (CH_2), 41.5 (CH), 40.5 (CH_2), 39.5 (CH_2), 31.5 (CH_2), 30.7 (CH_2), 26.5 (CH_2), 26.5 (CH_2), 26.4 (CH_2).

HRMS: calcd. for $C_{20}H_{27}NO_3Na$ $[M+Na]^+$: 352.1883; found (ESI$^+$): 352.1876.

ν_{max} (neat/cm^{-1}): 691, 1114, 1215, 1448, 1632, 1683, 2851, 2823.

2-(Prop-2-enyl)-1-morpholino-4-phenylbutane-1,4-dione (3p)

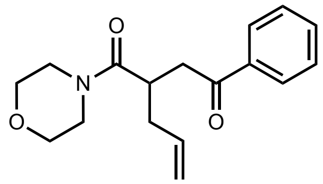

$C_{17}H_{21}FNO_3$
MW: 287.36

Synthesised according to **GP-5** from **2v** (38.0 mg, 0.1 mmol), trimethyl((1-phenylvinyl)oxy)silane (96 mg, 0.5 mmol), and PTH (1.4 mg, 0.005 mmol) in 1,2-DCE (0.2 mL). Purification by column chromatography (silica gel; 0-50% EtOAc in hexanes) afforded the product **3p** as a yellow oil (13.3 mg, 0.046 mmol, 46%).

^{1}H NMR (400 MHz, $CDCl_3$): δ δ 7.99 – 7.92 (m, 2H, Ar *H*), 7.59 – 7.50 (m, 1H, Ar *H*), 7.44 (t, *J* = 7.7 Hz, 2H, Ar *H*), 5.77 (ddt, *J* = 17.2, 10.0, 7.1 Hz, 1H, C*H*=CH$_2$), 5.18 – 5.05 (m, 2H, CH=C*H*$_2$), 3.89 – 3.80 (m, 1H, C*H*$_2$), 3.80 – 3.56 (m, 8H, C*H*$_2$C(O), C*H*$_2$), 3.48 – 3.38 (m, 1H, C*H*), 3.02 (dd, *J* = 17.9, 3.6 Hz, 1H, C*H*$_2$C(O)), 2.48 – 2.36 (m, 1H, C*H*$_2$), 2.32 – 2.21 (m, 1H, C*H*$_2$).

^{13}C{^{1}H} NMR (101 MHz, $CDCl_3$): δ 199.1 (*C*=O), 173.5 (*C*=O), 136.8 (Ar *C*), 135.0 (*C*H=CH$_2$), 133.4 (Ar *C*H), 128.7 (Ar *C*H), 128.2 (Ar *C*H), 117.9 (CH=*C*H$_2$), 67.1 (*C*H$_2$), 67.0 (*C*H$_2$), 46.6 (*C*H$_2$), 42.5 (*C*H$_2$), 41.2 (*C*H$_2$C(O)), 37.0 (*C*H$_2$), 35.8 (*C*H).

HRMS: calcd. for $C_{17}H_{21}NO_3Na$ [M+Na]$^+$: 310.1414; found (ESI$^+$): 310.1406.

ν_{max} (neat/cm^{-1}):

2-Benzyl-1-morpholino-4-phenylbutane-1,4-dione (3q)

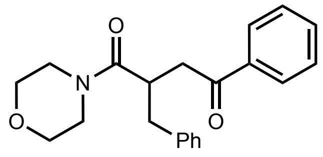

$C_{21}H_{23}NO_3$
MW: 337.42

Synthesised according to **GP-5** from **2u** (43.0 mg, 0.1 mmol), trimethyl((1-phenylvinyl)oxy)silane (96 mg, 0.5 mmol), and PTH (1.4 mg, 0.005 mmol) in 1,2-DCE (0.2 mL). Purification by column chromatography (silica gel; 0-50% EtOAc in hexanes) afforded the product **3q** as a yellow oil (21.6 mg, 0.064 mmol, 64%).

^{1}H NMR (400 MHz, CDCl$_3$): δ 7.99 – 7.90 (m, 2H, Ar *H*), 7.60 – 7.53 (m, 1H, Ar *H*), 7.45 (dd, J = 8.4, 7.0 Hz, 2H, Ar *H*), 7.34 – 7.18 (m, 5H, Ar *H*), 3.76 (dd, *J* = 17.9, 9.5 Hz, 1H, CH_2C(O)), 3.66 – 3.33 (m, 7H, 3 x CH_2, CH_{2A}), 3.20 – 3.11 (m, 1H, CH_2), 3.07 (dd, *J* = 17.9, 3.6 Hz, 1H, CH_2C(O)), 3.01 – 2.87 (m, 2H, CH_{2A}, CH_{2B}), 2.82 (dd, *J* = 13.0, 5.8 Hz, 1H, CH_2).

^{13}C{^{1}H} NMR (101 MHz, CDCl$_3$) δ 199.0 (*C*=O), 173.3 (*C*=O), 139.1 (Ar *C*), 136.7 (Ar *C*), 133.4 (Ar *C*H), 129.2 (Ar *C*H), 128.7 (Ar *C*H), 128.7 (Ar *C*H), 128.2 (Ar *C*H), 126.9 (Ar *C*H), 66.8 (*C*H$_2$), 66.4 (*C*H$_2$), 46.4 (*C*H$_2$), 42.3 *C*H$_2$C(O)), 42.3 (*C*H$_2$), 39.6 (*C*H$_2$), 38.2 (*C*H).

HRMS: calcd. for $C_{21}H_{23}NO_3Na$ [M+Na]$^+$: 360.1570; found (ESI$^+$): 360.1577.

ν_{max} (neat/cm^{-1}): 700, 737, 752, 1113, 1214, 1447, 1632, 1682, 2856.

4-Phenyl-1-(piperidin-1-yl)-2-(4-(trifluoromethyl)phenyl)butane-1,4-dione (3r)

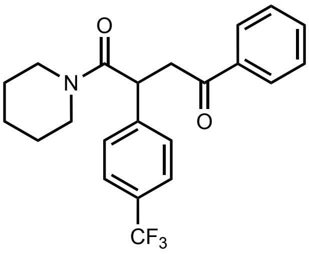

$C_{21}H_{22}F_3NO_2$
MW: 389.42

Synthesised according to **GP-5** from **2h′** (47.9 mg, 0.1 mmol), trimethyl((1-phenylvinyl)oxy)silane (96 mg, 0.5 mmol), and PTH (1.4 mg, 0.005 mmol) in 1,2-DCE (0.2 mL). Purification by column chromatography (silica gel; 0-50% EtOAc in hexanes) afforded the product **3r** as a yellow oil (27.2 mg, 0.070 mmol, 70%).

^{1}H NMR (400 MHz, $CDCl_3$): δ 8.00 – 7.93 (m, 2H, Ar *H*), 7.60 (d, *J* = 8.1 Hz, 2H, Ar *H*), 7.57 – 7.50 (m, 1H, Ar *H*), 7.48 (d, *J* = 8.1 Hz, 2H, Ar *H*), 7.43 (t, *J* = 7.8 Hz, 2H, Ar *H*), 4.66 (dd, *J* = 9.5, 4.0 Hz, 1H, C*H*), 4.12 (dd, *J* = 17.8, 9.5 Hz, 1H, $CH_2C(O)$), 3.65 – 3.57 (m, 1H, CH_2), 3.55 – 3.45 (m, 2H, CH_2), 3.40 – 3.32 (m, 1H, CH_2), 3.06 (dd, *J* = 17.8, 4.1 Hz, 1H, $CH_2C(O)$), 1.64 – 1.48 (m, 4H, CH_2), 1.47 – 1.37 (m, 1H, CH_{2A}), 1.10 – 0.99 (m, 1H, CH_{2B}).

^{13}C{^{1}H} NMR (101 MHz, $CDCl_3$): 198.3 (*C*=O), 169.6 (*C*=O), 144.2 (Ar *C*), 136.6 (Ar *C*), 133.4 (Ar *C*H), 129.6 (q, *J* = 32.7 Hz, Ar *C*), 128.7 (Ar *C*H), 128.33 (Ar *C*H), 128.30 (Ar *C*H), 126.1 (q, *J* = 3.7 Hz, Ar *C*H), 124.2 (q, *J* = 271.9 Hz, CF_3), 46.9 ($CH_2C(O)$), 44.2 (CH_2), 43.9 (*C*H), 43.6 (CH_2), 26.1 (CH_2), 25.6 (CH_2), 24.6 (CH_2).

^{19}F NMR (376 MHz, $CDCl_3$): δ -62.51 (s, CF_3).

HRMS: calcd. for $C_{22}H_{22}F_3NO_2Na$ $[M+Na]^+$: 412.1495; found (ESI^+): 412.1492.

ν_{max} (neat/cm^{-1}): 706, 734, 1017, 1067, 1121, 1163, 1322, 1443, 1635, 1684, 2936.

2,4-Diphenyl-1-(4-tosylpiperazin-1-yl)butane-1,4-dione (3s)

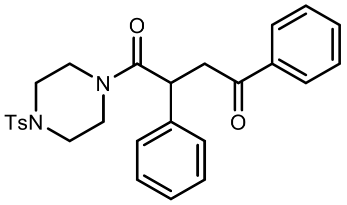

$C_{27}H_{28}N_2O_4S$
MW: 476.59

Synthesised according to **GP-5** from **2l** (48.3 mg, 0.1 mmol), trimethyl((1-phenylvinyl)oxy)silane (96 mg, 0.5 mmol), and PTH (1.4 mg, 0.005 mmol) in 1,2-DCE (0.2 mL). Purification by column chromatography (silica gel; 0-50% EtOAc in hexanes) afforded the product as an off yellow oil (33.2 mg, 0.061 mmol, 61%).

^{1}H NMR (400 MHz, $CDCl_3$): δ 7.95 – 7.87 (m, 2H, Ar *H*), 7.57 – 7.46 (m, 3H, Ar *H*), 7.45 – 7.37 (m, 2H, Ar *H*), 7.35 – 7.31 (m, 2H, Ar *H*), 7.26 – 7.15 (m, 5H, Ar *H*), 4.45 (dd, J = 10.0, 3.6 Hz, 1H, C*H*), 4.04 (dd, J = 17.9, 10.0 Hz, 1H, CH_2C(O)), 3.78 – 3.59 (m, 3H, CH_2), 3.50 – 3.40 (m, 1H, CH_2), 3.06 – 2.89 (m, 3H, CH_2C(O) + CH_2), 2.87 – 2.76 (m, 1H, CH_2), 2.47 (s, 3H, CH_3), 2.41 – 2.30 (m, 1H).

^{13}C{^{1}H} NMR (101 MHz, $CDCl_3$): δ 198.5 (*C*=O), 170.9 (*C*=O), 144.0 (Ar *C*), 139.0 (Ar *C*), 136.5 (Ar *C*), 133.4 (Ar *C*H), 132.4 (Ar *C*), 129.9 (Ar *C*H), 129.3 (Ar *C*H), 128.7 (Ar *C*H), 128.2 (Ar *C*H), 127.9 (Ar *C*H), 127.7 (Ar *C*H), 127.5 (Ar *C*H), 45.8 (*C*H_2), 45.6 (*C*H_2), 45.2 (*C*H_2), 44.3 (*C*H_2C(O)), 44.2 (*C*H_2), 41.7 (*C*H_2), 21.7 (*C*H_3).

HRMS: calcd. for $C_{27}H_{29}N_2O_4S$ $[M+H]^+$: 477.1843; found (ESI^+): 477.1849.

ν_{max} (neat/cm^{-1}): 629, 1031, 1239, 1445, 1480, 1637, 1691, 2849, 2933.

5.2 General Procedure 6 (GP-6): Photocatalytic α-Alkenylation

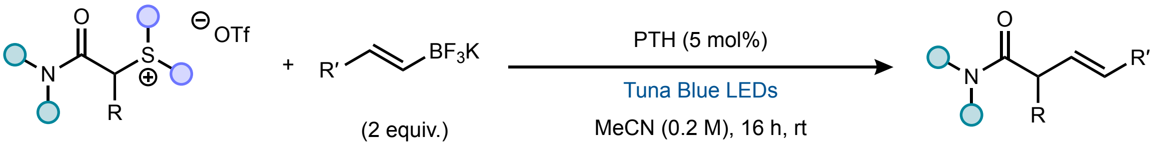

An oven-dried microwave vial was charged with sulfonium salt (0.1 mmol), potassium trifluoroborate salt (0.2 mmol), and 10-phenylphenothiazine (**PTH**, 1.4 mg, 0.005 mmol) and then sealed and evacuated and back-filled with dinitrogen 3 times. Anhydrous MeCN (0.5 mL, 0.2 M) was then added. The sample was stirred under constant irradiation (broad wavelength *Tuna Blue* Kessil lamp, 5 cm from light source) with fan cooling overnight. The crude material was purified directly by column chromatography using eluents given below to afford the pure product.

(*E*)-1-Morpholino-2,4-diphenylbut-3-en-1-one (4a)

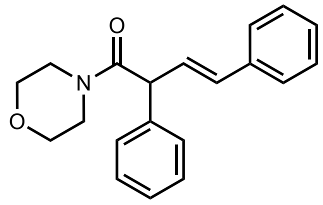

$C_{20}H_{21}NO_2$
MW: 307.39

Synthesised according to **GP-6** from **2a** (179 mg, 0.1 mmol), potassium (*E*)-styryl trifluoroborate (42.0 mg, 0.2 mmol), and PTH (1.4 mg, 0.005 mmol) in MeCN (0.5 mL). Purification by column chromatography (silica gel; 0-50% EtOAc in hexanes) afforded the product **4a** as a pale yellow oil (26.5 mg, 0.086 mmol, 86%, 18.5:1 *E*/*Z* ratio).

^{1}H NMR (400 MHz, $CDCl_3$): δ 7.41 – 7.25 (m, 9H, Ar *H*), 7.24 – 7.17 (m, 1H, Ar *H*), 6.67 (dd, *J* = 15.9, 7.9 Hz, 1H, CH-C*H*=CH), 6.39 (d, *J* = 15.9 Hz, 1H, CH-CH=C*H*), 4.61 (d, *J* = 8.0 Hz, 1H, C*H*), 3.84 – 3.73 (m, 1H, CH_{2A}), 3.73 – 3.65 (m, 1H, CH_{2B}), 3.65 – 3.44 (m, 4H, 2 × CH_2), 3.45 – 3.36 (m, 1H CH_{2A}), 3.33 – 3.22 (m, 1H, CH_{2B}).

^{13}C{^{1}H} NMR (101 MHz, $CDCl_3$): 170.6 (*C*=O), 138.8 (Ar *C*), 136.9 (Ar *C*), 131.3 (CH-CH=*C*H), 129.2 (Ar *C*H), 129.1 (CH-*C*H=CH), 128.6 (Ar *C*H), 128.0 (Ar *C*H), 127.6 (Ar *C*H), 127.4 (Ar *C*H), 126.6 (Ar *C*H), 66.8 (CH_2), 66.5 (CH_2), 52.8 (*C*H), 46.3 (CH_2), 42.5 (CH_2).

HRMS: calcd. for $C_{20}H_{21}NO_2Na$ $[M+Na]^+$: 330.1465; found (ESI$^+$): 330.1471.

ν_{max} (neat/cm^{-1}): 700, 1030, 1115, 1431, 1449, 1641, 2854, 2919, 2964, 3026.

(*E*)-4-(2-Bromophenyl)-1-morpholino-2-phenylbut-3-en-1-one (4b)

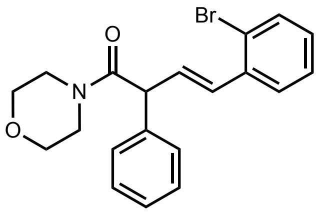

$C_{20}H_{20}BrNO_2$
MW: 386.29

Synthesised according to **GP-6** from **2a** (41.5 mg, 0.1 mmol), potassium (*E*)-2-bromostyryl trifluoroborate (57.8 mg, 0.2 mmol), and PTH (1.4 mg, 0.005 mmol) in MeCN (0.5 mL). Purification by column chromatography (silica gel; 0-50% EtOAc in hexane) afforded the product **4b** as a yellow oil (32.1 mg, 0.083 mmol, 83%, >20:1 E/Z ratio).

^{1}H NMR (400 MHz, $CDCl_3$): δ 7.55 (dd, *J* = 7.9, 1.7 Hz, 1H, Ar *H*), 7.50 (dd, *J* = 7.9, 1.3 Hz, 1H, Ar *H*), 7.41 – 7.25 (m, 5H, Ar *H*), 7.26 – 7.18 (m, 1H, Ar *H*), 7.06 (app td, *J* = 7.8, 1.7 Hz, 1H, Ar *H*), 6.75 (d, *J* = 15.8 Hz, 1H, CH-CH=C*H*), 6.62 (dd, *J* = 15.9, 7.9 Hz, 1H, CH-C*H*=CH), 4.65 (d, *J* = 8.0 Hz, 1H, C*H*), 3.85 – 3.45 (m, 6H, CH_2), 3.44 – 3.22 (m, 2H, CH_2).

^{13}C{^{1}H} NMR (101 MHz, $CDCl_3$): δ 170.4 (*C*=O), 138.5 (Ar *C*), 136.7 (Ar *C*), 132.8 (Ar *C*H), 132.2 (CH-CH=*C*H), 129.8 (CH-*C*H=CH), 129.3 (Ar *C*), 128.9 (Ar *C*H), 128.0 (Ar *C*H), 127.6 (Ar *C*H), 127.5 (Ar *C*H), 127.4 (Ar *C*H), 123.6 (Ar *C*H), 66.9 (*C*H_2), 66.4 (*C*H_2), 52.8 (*C*H), 46.3 (*C*H_2), 42.5 (*C*H_2).

HRMS: calcd. for $C_{20}H_{20}BrNO_2Na$ $[M+Na]^+$: 408.0570; found (ESI^+): 408.0576.

ν_{max} (neat/cm^{-1}): 700, 750, 964, 1029, 1219, 1270, 1433, 1639, 2855.

(E)-4-(4-Fluorophenyl)-1-morpholino-2-phenylbut-3-en-1-one (4c)

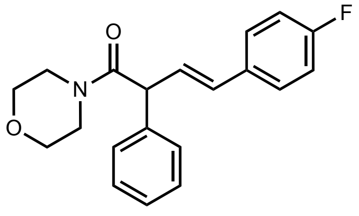

$C_{20}H_{20}FNO_2$
MW: 325.38

Synthesised according to **GP-6** from **2a** (41.5 mg, 0.1 mmol), potassium (*E*)-4-fluorostyryl trifluoroborate (45.6 mg, 0.2 mmol), and PTH (1.4 mg, 0.005 mmol) in MeCN (0.5 mL). Purification by column chromatography (silica gel; 0-50% EtOAc in hexane) afforded the product **4c** as a yellow oil (28.6 mg, 0.088 mmol, 88%, >20:1 E/Z ratio).

^{1}H NMR (400 MHz, $CDCl_3$): δ 7.34 – 7.20 (m, 7H, Ar *H*), 6.95 – 6.88 (m, 2H, Ar *H*), 6.54 (dd, *J* = 15.9, 7.9 Hz, 1H, CH-C*H*=CH), 6.29 (d, *J* = 15.9 Hz, 1H, CH-CH=C*H*), 4.54 (d, *J* = 7.9 Hz, 1H, C*H*), 3.84 – 3.13 (m, 8H, CH_2).

^{13}C{^{1}H} NMR (101 MHz, $CDCl_3$): δ 170.6 (*C*=O), 162.4 (d, *J* = 246.8 Hz, Ar *C*F), 138.6 (Ar *C*), 133.1 (d, *J* = 3.3 Hz, Ar *C*), 130.0 (CH-*C*H=CH), 129.2 (Ar *C*H), 129.1 (d, *J* = 2.4 Hz, CH-CH=*C*H), 128.1 (d, *J* = 8.1 Hz, Ar *C*H), 127.9 (Ar *C*H), 127.5 (Ar *C*H), 115.4 (d, *J* = 21.6 Hz, Ar *C*H), 66.9 (CH_2), 66.4 (CH_2), 52.7 (*C*H), 46.3 (CH_2), 42.5 (CH_2).

^{19}F NMR (376 MHz, $CDCl_3$): δ 114.56 – -114.65 (m).

HRMS: calcd. for $C_{20}H_{20}FNO_2Na$ [M+Na]$^+$: 348.1370; found (ESI$^+$): 348.1374.

ν_{max} (neat/cm^{-1}): 700, 728, 832, 1113, 1223, 1433, 1508, 1599, 1635, 2856.

(*E*)-4-(4-Methoxyphenyl)-1-morpholino-2-phenylbut-3-en-1-one (4d)

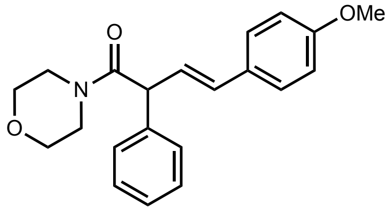

$C_{21}H_{23}NO_3$
MW: 337.42

Synthesised according to **GP-6** from **2a** (41.5 mg, 0.1 mmol), potassium (*E*)-4-methoxystyryl trifluoroborate (48.0 mg, 0.2 mmol), and PTH (1.4 mg, 0.005 mmol) in MeCN (0.5 mL). Purification by column chromatography (silica gel; 0-50% EtOAc in hexane) afforded the product **4d** as a yellow oil (19.0 mg, 0.056 mmol, 56%, >20:1 E/Z ratio).

^{1}H NMR (400 MHz, $CDCl_3$): δ 7.39 – 7.27 (m, 7H, Ar *H*), 6.84 – 6.77 (m, 2H, Ar *H*), 6.51 (dd, *J* = 15.9, 7.9 Hz, 1H, CH-C*H*=CH), 6.33 (d, *J* = 15.9 Hz, 1H, CH-CH=C*H*), 4.58 (d, *J* = 7.9 Hz, 1H, C*H*), 3.78 (s, 3H, CH_3), 3.72 – 3.23 (m, 8H, CH_2).

^{13}C{^{1}H} NMR (101 MHz, $CDCl_3$): δ 170.8 (*C*=O), 159.3 (Ar *C*O), 139.0 (Ar *C*), 130.8 (CH-CH=*C*H), 129.8 (Ar *C*), 129.1 (Ar *C*H), 128.0 (Ar *C*H), 127.8 (Ar *C*H), 127.4 (Ar *C*H), 126.9 (CH-*C*H=CH), 114.0 (Ar *C*H), 66.9 (*C*H_2), 66.5 (*C*H_2), 55.4 (*C*H_3), 52.8 (*C*H), 46.3 (*C*H_2), 42.5 (*C*H_2).

HRMS: calcd. for $C_{21}H_{23}NO_3Na$ [M+Na]$^+$: 360.1570; found (ESI$^+$): 360.1563.

ν_{max} (neat/cm^{-1}): 700, 1025, 1060, 1428, 1447, 1632, 2856.

(*E*)-4-(3-Methylphenyl)-1-morpholino-2-phenylbut-3-en-1-one (4e)

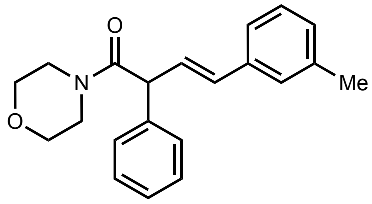

$C_{21}H_{23}NO_2$
MW: 321.42

Synthesised according to **GP-6** from **2a** (41.5 mg, 0.1 mmol), potassium (*E*)-3-methylstyryl trifluoroborate (44.8 mg, 0.2 mmol), and PTH (1.4 mg, 0.005 mmol) in MeCN (0.5 mL). Purification by column chromatography (silica gel; 0-50% EtOAc in hexane) afforded the product **4e** as a yellow oil (22.2 mg, 0.069 mmol, 69%, >20:1 E/Z ratio).

^{1}H NMR (400 MHz, $CDCl_3$): δ 7.33 – 7.05 (m, 8H, Ar *H*), 6.99 – 6.93 (m, 1H, Ar *H*), 6.58 (dd, *J* = 15.9, 8.0 Hz, 1H, CH-C*H*=CH), 6.29 (d, *J* = 15.9 Hz, 1H, CH-CH=C*H*), 4.53 (d, *J* = 8.0 Hz, 1H, C*H*), 3.79 – 3.14 (m, 8H, CH_2), 2.25 (s, 3H, CH_3).

^{13}C{^{1}H} NMR (101 MHz, $CDCl_3$): δ 170.7 (*C*=O), 138.8 (Ar *C*), 138.1 (Ar *C*), 136.8 (Ar *C*), 131.4 (CH-CH=*C*H), 129.2 (Ar *C*H), 128.9 (CH-*C*H=CH), 128.5 (Ar *C*H), 128.4 (Ar *C*H), 128.0 (Ar *C*H), 127.4 (Ar *C*H), 127.2 (Ar *C*H), 123.9 (Ar *C*H), 66.9 (*C*H_2), 66.5 (*C*H_2), 52.8 (*C*H), 46.3 (*C*H_2), 42.5 (*C*H_2), 21.5 (*C*H_3).

HRMS: calcd. for $C_{21}H_{23}NONa$ $[M+Na]^+$: 344.1621; found (ESI$^+$): 344.1618.

ν_{max} (neat/cm^{-1}): 700, 966, 1031, 1115, 1432, 1453, 1639, 2856.

(*E*)-4-(Thiophen-3-yl)-1-morpholino-2-phenylbut-3-en-1-one (4f)

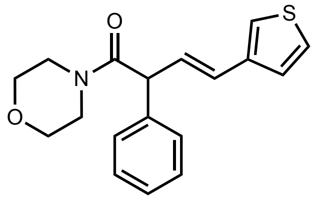

$C_{18}H_{19}NO_2S$
MW: 313.41

Synthesised according to **GP-6** from **2a** (41.5 mg, 0.1 mmol), potassium (*E*)-(thiophen-3-yl)vinyl trifluoroborate (43.2 mg, 0.2 mmol), and PTH (1.4 mg, 0.005 mmol) in MeCN (0.5 mL). Purification by column chromatography (silica gel; 0-50% EtOAc in hexane) afforded the product **4f** as a yellow oil (28.7 mg, 0.092 mmol, 92%, >20:1 E/Z ratio).

^{1}H NMR (400 MHz, $CDCl_3$): δ 7.38 – 7.32 (m, 2H, Ar *H*), 7.32 – 7.26 (m, 3H, Ar *H*), 7.22 (app t, *J* = 2.3 Hz, 2H, Ar *H*), 7.10 (app t, *J* = 1.9 Hz, 1H, Ar *H*), 6.51 (dd, *J* = 15.9, 7.7 Hz, 1H, CH-C*H*=CH), 6.38 (d, *J* = 15.9 Hz, 1H, CH-CH=C*H*), 4.55 (d, *J* = 7.7 Hz, 1H, C*H*), 3.84 – 3.74 (m, 1H, CH_2), 3.73 – 3.64 (m, 1H, CH_2), 3.63 – 3.44 (m, 4H, CH_2), 3.43 – 3.21 (m, 2H, CH_2).

^{13}C{^{1}H} NMR (101 MHz, $CDCl_3$): δ 170.7 (*C*=O), 139.6 (Ar *C*), 138.8 (Ar *C*), 129.2 (Ar *C*H), 129.0 (CH-<u>CH</u>=CH), 128.0 (Ar *C*H), 127.4 (Ar *C*H), 126.0 (Ar *C*H), 125.6 (CH-CH=<u>CH</u>), 125.3 (Ar *C*H), 122.1 (Ar *C*H), 66.9 (*C*H_2), 66.5 (*C*H_2), 52.7 (*C*H), 46.3 (*C*H_2), 42.5 (*C*H_2).

HRMS: calcd. for $C_{18}H_{19}NO_2SNa$ $[M+Na]^+$: 336.1029; found (ESI$^+$): 336.1038.

ν_{max} (neat/cm^{-1}): 580, 700, 729, 761, 964, 1030, 1113, 1217, 1451, 1634, 2854.

(*E*)-1-Morpholino-2-phenylpent-3-en-1-one (4g)

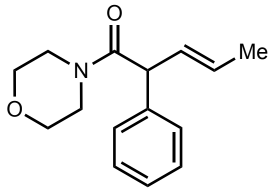

$C_{15}H_{19}NO_2$
MW: 245.32

Synthesised according to **GP-6** from **2a** (41.5 mg, 0.1 mmol), potassium (*E*)-prop-1-en-1-yl trifluoroborate (29.6 mg, 0.2 mmol), and PTH (1.4 mg, 0.005 mmol) in MeCN (0.5 mL). Purification by column chromatography (silica gel; 0-50% EtOAc in hexane) afforded the product **4g** as a yellow oil (9.2 mg, 0.038 mmol, 38%, 7.9:1 E/Z ratio).

^{1}H NMR (400 MHz, $CDCl_3$): δ 7.37 – 7.29 (m, 2H, Ar *H*), 7.25 – 7.23 (m, 2H, Ar *H*), 7.23 – 7.21 (m, 1H, Ar *H*), 5.89 (ddq, J = 15.3, 7.9, 1.6 Hz, 1H, CH-C*H*=CH), 5.54 – 5.40 (m, 1H, CH-CH=C*H*), 4.39 (d, *J* = 7.9 Hz, 1H, C*H*), 3.79 – 3.09 (m, 8H, CH_2), 1.70 (dd, *J* = 6.6, 1.6 Hz, 3H, CH_3).

^{13}C{^{1}H} NMR (101 MHz, $CDCl_3$): δ 171.1 (*C*=O), 139.4 (Ar *C*), 130.4 (CH-*C*H=CH), 129.1 (Ar *C*H), 127.9 (Ar *C*H), 127.23 (CH-CH=*C*H), 127.19 (Ar *C*H), 66.9 (*C*H_2), 66.5 (*C*H_2), 52.7 (*C*H), 46.2 (*C*H_2), 42.4 (*C*H_2), 18.1 (*C*H_3).

HRMS: calcd. for $C_{15}H_{19}NO_2Na$ $[M+Na]^+$: 268.1308; found (ESI$^+$): 268.1300.

ν_{max} (neat/cm^{-1}): 700, 1032, 1114, 1436, 1494, 1643, 1724, 2856.

(*E*)-2,4-Diphenyl-1-(piperidin-1-yl)but-3-en-1-one (4h)

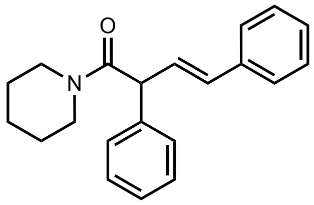

$C_{21}H_{23}NO$
MW: 305.42

Synthesised according to **GP-6** from **2h** (41.4 mg, 0.1 mmol), potassium (*E*)-styryl trifluoroborate (42.0 mg, 0.2 mmol), and PTH (1.4 mg, 0.005 mmol) in MeCN (0.5 mL). Purification by column chromatography (silica gel; 0-50% EtOAc in hexane) afforded the product **3h** as a yellow oil (9.2 mg, 0.038 mmol, 38%, 7.9:1 E/Z ratio).

^{1}H NMR (400 MHz, $CDCl_3$): δ 7.33 – 7.23 (m, 6H, Ar *H*), 7.23 – 7.12 (m, 3H, Ar *H*), 7.15 – 7.06 (m, 1H, Ar *H*), 6.61 (dd, *J* = 15.9, 8.0 Hz, 1H, CH-C*H*=CH), 6.30 (d, *J* = 15.9 Hz, 1H, CH-CH=C*H*), 4.57 (dd, *J* = 8.0, 1.0 Hz, 1H, C*H*), 3.63 (ddd, *J* = 12.7, 6.3, 3.3 Hz, 1H, CH_2), 3.48 – 3.37 (m, 1H, CH_2), 3.31 (app t, *J* = 5.5 Hz, 2H, CH_2), 1.55 – 1.26 (m, 5H, CH_2), 1.06 (app dq, *J* = 11.3, 6.1 Hz, 1H, CH_2).

^{13}C{^{1}H} NMR (101 MHz, $CDCl_3$): δ 170.2 (*C*=O), 139.4 (Ar *C*), 137.2 (Ar *C*), 130.9 (CH-CH=*C*H), 129.9 (CH-*C*H=CH), 129.0 (Ar *C*H), 128.5 (Ar *C*H), 128.1 (Ar *C*H), 127.4 (Ar *C*H), 127.1 (Ar *C*H), 126.6 (Ar *C*H), 52.9 (*C*H), 46.9 (*C*H_2), 43.2 (*C*H_2), 26.2 (*C*H_2), 25.6 (*C*H_2), 24.6 (*C*H_2).

HRMS: calcd. for $C_{21}H_{23}NONa$ $[M+Na]^+$: 328.1672; found (ESI^+): 328.1665.

ν_{max} (neat/cm^{-1}): 695, 743, 964, 1219, 1264, 1438, 1632, 1726, 2855, 2935.

(*E*)-2,4-Diphenyl-1-(4-tosylpiperazin-1-yl)but-3-en-1-one (4i)

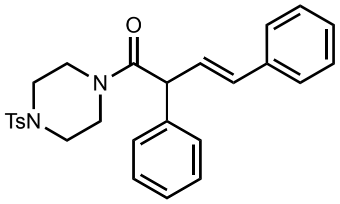

$C_{27}H_{28}N_2O_3S$
MW: 460.59

Synthesised according to **GP-6** from **2i** (57 mg, 0.1 mmol), potassium (*E*)-styryl tetrafluoroborate (42.0 mg, 0.2 mmol), and PTH (1.4 mg, 0.005 mmol) in MeCN (0.5 mL). Purification by column chromatography (silica gel; 0-30% EtOAc in hexanes) afforded the product **4i** as a white solid (31.6 mg, 0.069 mmol, 69%, >20:1 E/Z ratio).

^{1}H NMR (400 MHz, CDCl$_3$): δ 7.56 – 7.50 (m, 2H, Ar *H*), 7.33 – 7.15 (m, 12H, Ar *H*), 6.57 (dd, *J* = 15.9, 7.9 Hz, 1H, CH-C*H*=CH), 6.30 (d, *J* = 15.9 Hz, 1H, CH-CH=C*H*), 4.52 (d, *J* = 7.9 Hz, 1H, C*H*), 3.91 – 3.79 (m, 1H, CH_{2A}), 3.69 – 3.59 (m, 1H, CH_{2B}), 3.59 – 3.43 (m, 2H, CH_2), 3.08 – 2.98 (m, 1H, CH_{2A}), 2.91 – 2.77 (m, 2H, CH_2), 2.46 (s, 3H, CH_3), 2.44 – 2.34 (m, 1H, CH_{2B}).

^{13}C{^{1}H} NMR (101 MHz, CDCl$_3$): δ 170.5 (*C*=O), 144.1 (Ar *C*), 138.5 (Ar *C*), 136.8 (Ar *C*), 132.2 (Ar *C*), 131.4 (CH-CH=*C*H), 129.9 (Ar *C*H), 129.2 (Ar *C*H), 128.8 (CH-*C*H=CH), 128.6 (Ar *C*H), 127.94 (Ar *C*H), 127.85 (Ar *C*H), 127.7 (Ar *C*H), 127.5 (Ar *C*H), 126.6 (Ar *C*H), 52.9 (*C*H), 45.82 (*C*H$_2$), 45.77 (*C*H$_2$), 45.2 (*C*H$_2$), 41.5 (*C*H$_2$), 21.7 (*C*H$_3$).

HRMS: calcd. for $C_{27}H_{28}N_2O_3SNa$ [M+Na]$^+$: 483.1713; found (ESI$^+$): 483.1736.

ν_{max} (neat/cm^{-1}): 692, 722, 923, 963, 1166, 1256, 1274, 1344, 1446, 1596, 1631.

m.p.: 144-145 °C

(*E*)-*N*,*N*-Dimethyl-2,4-diphenylbut-3-enamide (4j)

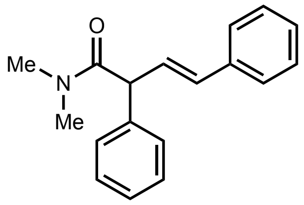

$C_{18}H_{19}NO$
MW: 265.36

Synthesised according to **GP-6** from **2d** (37.3 mg, 0.1 mmol), potassium (*E*)-styryl trifluoroborate (42.0 mg, 0.2 mmol), and PTH (1.4 mg, 0.005 mmol) in MeCN (0.5 mL). Purification by column chromatography (silica gel; 0-50% EtOAc in hexane) afforded the product **4j** as a yellow oil (16.5 mg, 0.62 mmol, 62%, >20:1 E/Z ratio).

^{1}H NMR (400 MHz, $CDCl_3$): δ 7.41 – 7.35 (m, 6H, Ar *H*), 7.32 – 7.27 (m, 3H, Ar *H*), 7.24 – 7.19 (m, 1H, Ar *H*), 6.70 (dd, *J* = 15.9, 8.1 Hz, 1H, CH-<u>*CH*</u>=CH), 6.41 (d, *J* = 15.9 Hz, 1H, CH-CH=<u>*CH*</u>), 4.67 (dd, *J* = 8.1, 0.9 Hz, 1H, C*H*), 3.03 (3H, CH_3), 3.02 (3H, CH_3).

^{13}C{^{1}H} NMR (101 MHz, $CDCl_3$): δ 172.1 (*C*=O), 139.0 (Ar *C*), 137.1 (Ar *C*), 131.0 (CH-CH=<u>CH</u>), 129.7 (CH-<u>CH</u>=CH), 129.0 (Ar *C*H), 128.5 (Ar *C*H), 128.1 (Ar *C*H), 127.5 (Ar *C*H), 127.3 (Ar *C*H), 126.6 (Ar *C*H), 52.9 (*C*H), 37.4 (CH_3), 36.1 (CH_3).

HRMS: calcd. for $C_{18}H_{19}NONa$ $[M+Na]^+$: 288.1359; found (ESI$^+$): 288.1362.

ν_{max} (neat/cm^{-1}): 696, 744, 965, 1179, 1395, 1493, 1639, 2926, 3026.

(*E*)-*N*-Methoxy-*N*-methyl-2,4-diphenylbut-3-enamide (4k)

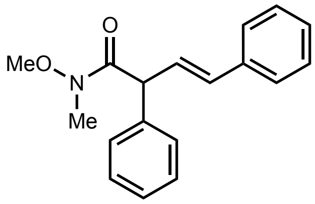

$C_{18}H_{19}NO_2$
MW: 281.35

Synthesised according to **GP-6** from **2f** (38.9 mg, 0.1 mmol), potassium (*E*)-styryl tetrafluoroborate (42.0 mg, 0.2 mmol), and PTH (1.4 mg, 0.005 mmol) in MeCN (0.5 mL). Purification by column chromatography (silica gel; 0-50% EtOAc in hexanes) afforded the product **4k** as a white solid (15.8 mg, 0.056 mmol, 56%, >20:1 E/Z ratio).

^{1}H NMR (400 MHz, $CDCl_3$): δ 7.43 – 7.17 (m, 10H, Ar *H*), 6.67 (dd, *J* = 15.9, 8.4 Hz, 1H, CH-C*H*=CH), 6.46 (d, *J* = 15.9 Hz, 1H, CH-CH=C*H*), 4.95 (d, *J* = 8.4 Hz, 1H, C*H*), 3.53 (s, 3H, CH_3), 3.22 (s, 3H, CH_3).

^{13}C{^{1}H} NMR (101 MHz, $CDCl_3$): δ 173.4 (*C*=O), 139.3 (Ar *C*), 137.0 (Ar *C*), 131.8 (CH-CH=*C*H), 128.9 (CH-*C*H=CH), 128.6 (Ar *C*H), 128.3 (Ar *C*H), 127.6 (Ar *C*H), 127.2 (Ar *C*H), 126.6 (Ar *C*H), 61.6 (CH_3), 51.4 (*C*H), 32.4 (CH_3).

HRMS: calcd. for $C_{18}H_{19}NO_2Na$ $[M+Na]^+$: 328.304.1308; found (ESI$^+$): 304.1315.

ν_{max} (neat/cm^{-1}): 696, 744, 992, 1175, 1382, 1659, 2937, 3026.

(*E*)-*N*-Methyl-2,4-diphenyl-*N*-(3-phenyl-3-(4-(trifluoromethyl)phenoxy)propyl)but-3-enamide (4l)

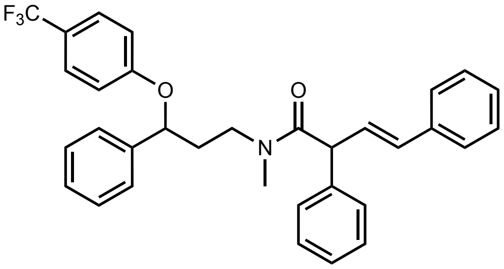

$C_{33}H_{30}F_3NO_2$
MW: 529.60

Synthesised according to **GP-6** from **2j** (64 mg, 0.1 mmol), potassium (*E*)-styrenyl tetrafluoroborate (42 mg, 0.2 mmol), and PTH (1.4 mg, 0.005 mmol) in MeCN (0.5 mL). Purification by column chromatography (silica gel; 0-20% EtOAc in hexanes) afforded the product **4l** as a white foam (35.8 mg, 0.068 mmol, 68%, >20:1 E/Z ratio, 1:1 d.r.). The diastereomers were then separated by preparative TLC (silica gel; 10% EtOAc in hexane).

Diastereomer 1 (*1:1 mixture of rotamers*):

^{1}H NMR (400 MHz, $CDCl_3$): δ 7.52 (d, *J* = 8.6 Hz, 1H, Ar *H*), 7.44 – 7.13 (m, 16H, Ar *H*), 6.97 (d, *J* = 8.6 Hz, 1H, Ar *H*), 6.87 (d, *J* = 8.5 Hz, 1H, Ar *H*), 6.66 (dd, *J* = 15.9, 8.1 Hz, 0.5H, CH-C*H*=CH), 6.57 (dd, *J* = 15.9, 8.4 Hz, 0.5H, CH-C*H*=CH), 6.37 (d, *J* = 15.9 Hz, 0.5H, CH-CH=C*H*), 5.81 (d, *J* = 15.9 Hz, 0.5H, CH-CH=C*H*), 5.22 – 5.14 (m, 1H, OC*H*), 4.62 (d, *J* = 8.1 Hz, 0.5H, C*H*), 4.50 (d, *J* = 8.5 Hz, 0.5H, C*H*), 3.86 – 3.74 (m, 0.5H, CH_2), 3.73 – 3.64 (m, 0.5H, CH_2), 3.64 – 3.54 (m, 0.5H, CH_2), 3.31 – 3.20 (m, 0.5H, CH_2), 3.01 (s, 1.5H NCH_3), 3.00 (s, 1.5H, NCH_3), 2.28 – 2.01 (m, 2H, CH_2).

^{13}C{^{1}H} NMR (101 MHz, $CDCl_3$): δ 172.3 (*C*=O), 172.1 (*C*=O), 140.1 (Ar *C*), 137.0 (Ar *C*), 136.9 (Ar *C*), 131.1 (CH-CH=*C*H), 130.7 (CH-CH=*C*H), 129.6 (CH-CH=*C*H), 129.5 (CH-CH=*C*H), 129.24 (Ar *C*H), 129.16 (Ar *C*H), 129.1 (Ar *C*H), 129.0 (Ar *C*H), 128.6 (Ar *C*H), 128.5 (Ar *C*H), 128.4 (Ar *C*H), 128.14 (Ar *C*H), 128.08 (Ar *C*H), 127.9 (Ar *C*H), 127.6 (Ar *C*H), 127.5 (Ar *C*H), 127.32 (Ar *C*H), 127.27 (Ar *C*H), 127.2 (Ar *C*H), 127.0 (Ar *C*H), 126.94 (Ar *C*H), 126.91 (Ar *C*H), 126.6 (Ar *C*H), 126.5 (Ar *C*H), 125.8 (Ar *C*H), 125.7 (Ar *C*H), 116.0 (Ar *C*H), 115.9 (Ar *C*H), 78.4 (*C*HOPh), 76.6 (*C*HOPh), 53.1 (*C*H), 52.8 (*C*H), 45.90 (*C*H_2), 45.87 (*C*H_2), 37.4 (*C*H_2), 36.4 (*C*H_2), 36.1 (*C*H_3), 33.6 (*C*H_3). *The quaternary carbon corresponding to the CF_3 was not observed, though its presence was confirmed by ^{19}F NMR spectroscopy.*

^{19}F NMR (376 MHz, $CDCl_3$): δ -61.57 (s, CF_3).

HRMS: calcd. for $C_{33}H_{30}F_3NO_2Na$ $[M+Na]^+$: 552.2121; found (ESI^+): 552.2107.

ν_{max} (neat/cm^{-1}): 722, 1067, 1111, 1161, 1249, 1326, 1516, 1614, 1640, 2926.

(*E*)-*N*-(3-(9,10-Ethanoanthracen-9(10*H*)-yl)propyl)-*N*-methyl-2,4-diphenylbut-3-enamide (4m)

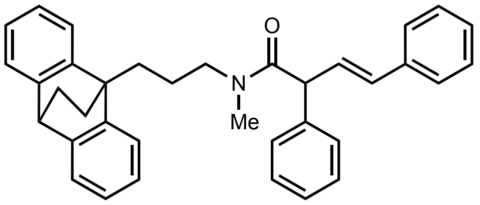

$C_{36}H_{35}NO$
MW: 497.68

Synthesised according to **GP-6** from **2k** (61 mg, 0.1 mmol), potassium (*E*)-styrenyl tetrafluoroborate (42 mg, 0.2 mmol), and PTH (1.4 mg, 0.005 mmol) in MeCN (0.5 mL). Purification by column chromatography (silica gel; 0-10% EtOAc in hexanes) afforded the product **4m** as a white foam (29.6 mg, 0.060 mmol, 60%, >20:1 E/Z ratio).

A 3:1 mixture of rotamers was observed at 298 K in $CDCl_3$

^{1}H NMR (400 MHz, $CDCl_3$): δ 7.49 – 7.00 (m, 18H, Ar *H*), 6.80 – 6.61 (m, 1H, CH-C*H*=CH), 6.49 – 6.14 (m, 1H, CH-CH=C*H*), 5.18 – 4.62 (m, 1H, C*H*), 4.30 – 4.22 (m, 1H, C*H*), 3.93 – 3.25 (m, 2H, C*H$_2$*), 3.17 – 2.88 (m, 3H, C*H$_3$*), 2.51 – 1.71 (m, 6H, C*H$_2$*), 1.55 – 1.28 (m, 2H, C*H$_2$*).

^{13}C{^{1}H} NMR (101 MHz, $CDCl_3$): δ 172.1 (*C*=O), 171.9 (*C*=O), 145.41 (Ar *C*), 145.38 (Ar *C*), 145.1 (Ar *C*), 145.02 (Ar *C*), 144.98 (Ar *C*), 139.4 (Ar *C*), 139.0 (Ar *C*), 137.1 (Ar *C*), 137.0 (Ar *C*), 131.4 (CH-*C*H=CH), 131.1 (CH-*C*H=CH), 130.0 (CH-CH=*C*H), 129.9 (CH-CH=*C*H), 129.8 (CH-CH=*C*H), 129.2 (Ar *C*H), 129.1 (Ar *C*H), 128.7 (Ar *C*H), 128.57 (Ar *C*H), 128.56 (Ar *C*H), 128.55 (Ar *C*H), 128.2 (Ar *C*H), 128.11 (Ar *C*H), 128.07 (Ar *C*H), 128.0 (Ar *C*H), 127.6 (Ar *C*H), 127.5 (Ar *C*H), 127.4 (Ar *C*H), 127.3 (Ar *C*H), 127.2 (Ar *C*H), 126.7 (Ar *C*H), 126.6 (Ar *C*H), 125.6 (Ar *C*H), 125.54 (Ar *C*H), 125.48 (Ar *C*H), 125.43 (Ar *C*H), 125.36 (Ar *C*H), 125.35 (Ar *C*H), 123.7 (Ar *C*H), 123.6 (Ar *C*H), 123.5 (Ar *C*H), 121.4 (Ar *C*H), 121.02 (Ar *C*H), 120.99 (Ar *C*H), 53.3 (*C*H), 53.0 (*C*H), 50.8 (N*C*H$_2$), 50.7 (N*C*H$_2$), 49.2 (N*C*H$_2$), 48.0 (N*C*H$_2$), 47.7 (N*C*H$_2$), 44.9 (*C*H), 44.7 (*C*H), 44.59 (*C*H), 44.55 (*C*H), 35.7 (*C*H$_2$), 35.5 (*C*H$_2$), 34.2 (*C*H$_2$), 34.0 (*C*H$_2$), 29.90 – 29.59 (m, *C*H$_2$), 28.2 (*C*H$_2$), 27.8 (*C*H$_2$), 27.7 (*C*H$_2$), 24.3 (*C*H$_2$), 24.2 (*C*H$_2$), 22.9 (*C*H$_2$).

HRMS: calcd. for $C_{36}H_{35}NONa$ $[M+Na]^+$: 520.2611; found (ESI$^+$): 520.2635.

ν_{max} (neat/cm^{-1}): 744, 962, 1164, 1398, 1452, 1489, 1635, 2937.

m.p.: 72-74 °C

(*E*)-2-(4-Methoxyphenyl)-1-morpholino-4-phenylbut-3-en-1-one (4n)

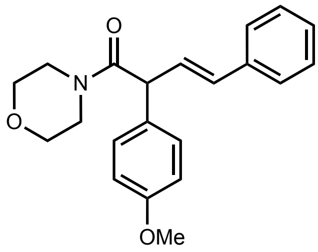

$C_{21}H_{23}NO_3$
MW: 337.42

Synthesised according to **GP-6** from **2m** (44.6 mg, 0.1 mmol), potassium (*E*)-styryl trifluoroborate (42.0 mg, 0.2 mmol), and PTH (1.4 mg, 0.005 mmol) in MeCN (0.5 mL). Purification by column chromatography (silica gel; 0-50% EtOAc in hexane) afforded the product **4n** as a yellow oil (16.6 mg, 0.49 mmol, 49%, >20:1 E/Z ratio).

^{1}H NMR (400 MHz, $CDCl_3$): δ 7.32 – 7.26 (m, 2H, Ar *H*), 7.21 – 7.10 (m, 5H, Ar *H*), 6.85 – 6.80 (m, 2H, Ar *H*), 6.57 (dd, *J* = 15.9, 7.8 Hz, 1H, 1H, CH-C*H*=CH), 6.29 (d, *J* = 15.9 Hz, 1H, CH-CH=C*H*), 4.49 (dd, *J* = 7.8, 0.9 Hz, 1H, C*H*), 3.74 (s, 3H, CH_3), 3.72 – 3.18 (m, 8H, CH_2).

^{13}C{^{1}H} NMR (101 MHz, $CDCl_3$): δ 171.0 (*C*=O), 158.9 (Ar *C*O), 137.0 (Ar *C*), 131.1 (CH-CH=*C*H), 130.8 (Ar *C*), 129.4 (CH-*C*H=CH), 129.1 (Ar *C*H), 128.6 (Ar *C*H), 127.6 (Ar *C*H), 126.6 (Ar *C*H), 114.6 (Ar *C*H), 66.9 (CH_2), 66.5 (CH_2), 55.4 (CH_3), 51.9 (*C*H), 46.3 (CH_2), 42.5 (CH_2).

HRMS: calcd. for $C_{21}H_{23}NO_3Na$ $[M+Na]^+$: 360.1570; found (ESI$^+$): 360.1579.

ν_{max} (neat/cm^{-1}): 833, 966, 1031, 1114, 1178, 1249, 1434, 1637, 1723, 2854.

(*E*)-2-(4-Methylphenyl)-1-morpholino-4-phenylbut-3-en-1-one (4o)

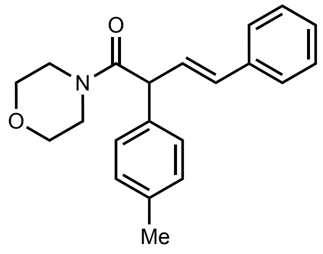

$C_{21}H_{23}NO_2$
MW: 321.42

Synthesised according to **GP-6** from **2n** (43 mg, 0.1 mmol), potassium (*E*)-styryl tetrafluoroborate (42 mg, 0.2 mmol), and PTH (1.4 mg, 0.005 mmol) in MeCN (0.5 mL). Purification by column chromatography (silica gel; 10-40% EtOAc in hexanes) afforded the product **4o** as a colourless oil (28.5 mg, 0.089 mmol, 89%, >20:1 E/Z ratio).

^{1}H NMR (400 MHz, CDCl$_3$): δ 7.40 – 7.13 (m, 9H, Ar *H*), 6.65 (dd, *J* = 15.9, 7.9 Hz, 1H, CH-C*H*=CH), 6.37 (d, *J* = 15.9 Hz, 1H, CH-CH=C*H*), 4.56 (d, *J* = 7.9 Hz, 1H, C*H*), 3.86 – 3.16 (m, 8H, 4 × C*H*$_2$), 2.33 (s, 3H, C*H*$_3$).

^{13}C{^{1}H} NMR (101 MHz, CDCl$_3$): δ 170.9 (*C*=O), 137.1 (Ar *C*), 137.0 (Ar *C*), 135.7 (Ar *C*), 131.1 (CH-CH=*C*H), 129.9 (Ar *C*H), 129.4 (CH-*C*H=CH), 128.5 (Ar *C*H), 127.8 (Ar *C*H), 127.6 (Ar *C*H), 126.6 (Ar *C*H), 66.9 (*C*H$_2$), 66.5 (*C*H$_2$), 52.4 (*C*H), 46.3 (*C*H$_2$), 42.5 (*C*H$_2$), 21.2 (*C*H$_3$).

ν_{max} (neat/cm^{-1}): 1030, 1158, 1258, 1322, 1490, 1592, 1637, 1732.

(*E*)-4-(1-Morpholino-1-oxo-4-phenylbut-3-en-2-yl)phenyl acetate (4p)

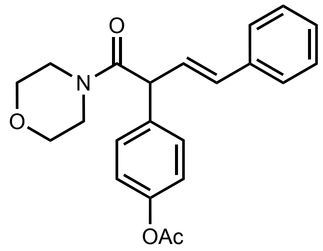

$C_{23}H_{24}NO_4$
MW 365.43

Synthesised according to **GP-6** from **2o** (47 mg, 0.1 mmol), potassium (*E*)-styryl tetrafluoroborate (42.0 mg, 0.2 mmol), and PTH (1.4 mg, 0.005 mmol) in MeCN (0.5 mL) using 390 nm lamps at 25% intensity. Purification by column chromatography (silica gel; 10-40% EtOAc in hexanes) afforded the product **4p** as a colourless foam (18.8 mg, 0.051 mmol, 51%, 17:1 E/Z ratio).

^{1}H NMR (400 MHz, $CDCl_3$): δ 7.39 – 7.27 (m, 6H, Ar *H*), 7.24 – 7.16 (m, 1H, Ar *H*), 7.11 – 7.05 (m, 2H, Ar *H*), 6.62 (dd, *J* = 15.9, 7.9 Hz, 1H, CH-C*H*=CH), 6.39 (d, *J* = 15.9 Hz, 1H, CH-CH=C*H*), 4.61 (d, *J* = 7.9 Hz, 1H, C*H*), 3.80 – 3.46 (m, 6H, 3 × CH_2), 3.46 – 3.30 (m, 2H, CH_2), 2.29 (s, 3H, CH_3).

^{13}C NMR (101 MHz, $CDCl_3$): δ 170.4 (*C*=O), 169.5 (*C*=O), 149.9 (Ar *C*), 136.7 (Ar *C*), 136.2 (Ar *C*), 131.5 (CH-CH=*C*H), 129.1 (Ar *C*H), 128.8 (Ar *C*H), 128.6 (CH-*C*H=CH), 127.8 (Ar *C*H), 126.6 (Ar *C*H), 122.2 (Ar *C*H), 66.9 (CH_2), 66.5 (CH_2), 51.9 (CH_2), 46.3 (*C*H), 42.5 (CH_2), 21.3 (CH_3).

HRMS: calcd. for $C_{22}H_{23}NO_4Na$ $[M+Na]^+$: 388.1519; found (ESI^+): 388.1507.

(*E*)-2-(4-(1-Morpholino-1-oxo-4-phenylbut-3-en-2-yl)phenyl)isoindoline-1,3-dione (4q)

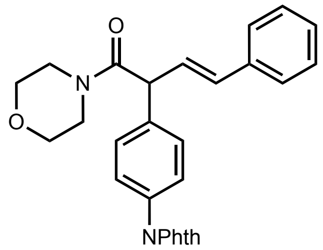

$C_{28}H_{24}N_2O_4$

MW: 452.51

Synthesised according to **GP-6** from **2p** (56 mg, 0.1 mmol), potassium (*E*)-styryl trifluoroborate (42 mg, 0.2 mmol), and PTH (1.4 mg, 0.005 mmol) in MeCN (0.5 mL) using 390 nm lamps. Purification by column chromatography (silica gel; 0-50% EtOAc in hexane) afforded the product **4p** as a yellow oil (31.2 mg, 0.069 mmol, 69%, >20:1 E/Z ratio).

^{1}H NMR (400 MHz, $CDCl_3$): δ 7.95 (dd, *J* = 5.5, 3.1 Hz, 2H, Ar *H*), 7.80 (dd, *J* = 5.5, 3.1 Hz, 2H, Ar *H*), 7.51 – 7.43 (m, 4H, Ar *H*), 7.38 (d, *J* = 7.5 Hz, 2H, Ar *H*), 7.29 (app t, *J* = 7.5 Hz, 2H, Ar *H*), 7.22 (app t, *J* = 7.3 Hz, 1H, Ar *H*), 6.66 (dd, *J* = 15.9, 8.0 Hz, 1H, CH-C*H*=CH), 6.44 (d, *J* = 15.9 Hz, 1H, CH-CH=C*H*), 4.68 (d, *J* = 8.0 Hz, 1H, C*H*), 3.80 – 3.36 (m, 8H, 4 × CH_2).

^{13}C NMR (101 MHz, $CDCl_3$): δ 170.3 (*C*=O), 167.3 (*C*=O), 138.5 (Ar *C*), 136.7 (Ar *C*), 134.6 (Ar *C*H), 131.8 (Ar *C*), 131.7 (CH-*C*H=CH), 131.0 (Ar *C*), 128.8 (Ar *C*H), 128.7 (Ar *C*H), 128.6 (CH-*C*H=CH), 127.8 (Ar *C*H), 127.0 (Ar *C*H), 126.6 (Ar *C*H), 123.9 (Ar *C*H), 67.0 (CH_2), 66.6 (CH_2), 52.3 (*C*H), 46.4 (CH_2), 42.5 (CH_2).

HRMS: calcd. for $C_{18}H_{23}N_2O_4$ [M-H]$^-$: 451.1677; found (APCI): 451.1663.

(*E*)-2-(4-Fluorophenyl)-1-morpholino-4-phenylbut-3-en-1-one (4r)

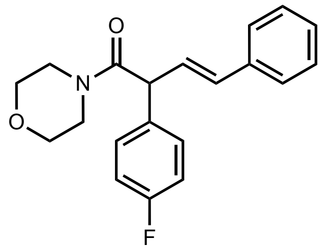

$C_{20}H_{20}FNO_2$
MW: 325.38

Synthesised according to **GP-6** from **2q** (43.4 mg, 0.1 mmol), potassium (*E*)-styryl trifluoroborate (42.0 mg, 0.2 mmol), and PTH (1.4 mg, 0.005 mmol) in MeCN (0.5 mL). Purification by column chromatography (silica gel; 0-50% EtOAc in hexane) afforded the product **4r** as a yellow oil (30.8 mg, 0.095 mmol, 95%, >20:1 E/Z ratio).

^{1}H NMR (400 MHz, $CDCl_3$): δ 7.37 – 7.14 (m, 7H, Ar *H*), 7.06 – 6.98 (m, 2H, Ar *H*), 6.58 (dd, *J* = 15.9, 7.8 Hz, 1H, CH-C*H*=CH), 6.35 (d, *J* = 15.9 Hz, 1H, CH-CH=C*H*), 4.58 (d, *J* = 7.8 Hz, 1H, C*H*), 3.77 – 3.27 (m, 8H, CH_2).

^{13}C{^{1}H} NMR (101 MHz, $CDCl_3$): δ 170.5 (*C*=O), 162.1 (d, *J* = 246.1 Hz, Ar *C*F), 136.7 (Ar *C*), 134.5 (d, *J* = 3.4 Hz, Ar *C*), 131.6 (CH-CH=*C*H), 129.7 (d, *J* = 7.9 Hz, Ar *C*H), 128.8 (Ar *C*H), 128.6 (CH-*C*H=CH), 127.8 (Ar *C*H), 126.6 (Ar *C*H), 116.0 (d, *J* = 21.5 Hz, Ar *C*H), 66.9 (*C*H_2), 66.5 (*C*H_2), 51.7 (*C*H), 46.3 (*C*H_2), 42.5 (*C*H_2).

^{19}F NMR (376 MHz, $CDCl_3$): -115.01 – -115.11 (m, Ar *F*)

HRMS: calcd. for $C_{20}H_{20}FNO_2Na$ [M+Na]$^+$: 348.1370; found (ESI$^+$): 348.1375.

ν_{max} (neat/cm^{-1}): 561, 743, 836, 1031, 1115, 1222, 1433, 1507, 1638, 1720, 2856.

(*E*)-2-((4-Trifluoromethyl)phenyl)-1-morpholino-4-phenylbut-3-en-1-one (4s)

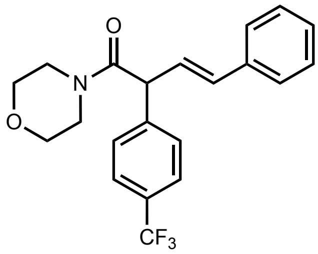

$C_{21}H_{20}F_3NO_2$
MW: 375.39

Synthesised according to **GP-6** from **2l** (43.4 mg, 0.1 mmol), potassium (*E*)-styryl trifluoroborate (42.0 mg, 0.2 mmol), and PTH (1.4 mg, 0.005 mmol) in MeCN (0.5 mL). Purification by column chromatography (silica gel; 0-50% EtOAc in hexane) afforded the product **4s** as a yellow oil (27.4 mg, 0.73 mmol, 73%, >20:1 E/Z ratio).

^{1}H NMR (400 MHz, $CDCl_3$): δ 7.62 (d, *J* = 8.1 Hz, 2H, Ar *H*), 7.46 (d, *J* = 8.1 Hz, 2H, Ar *H*), 7.41 – 7.34 (m, 2H, Ar *H*), 7.33 – 7.27 (m, 2H, Ar *H*), 7.25 – 7.19 (m, 1H, Ar *H*), 6.61 (dd, *J* = 15.9, 7.8 Hz, 1H, CH-C*H*=CH), 6.44 (d, *J* = 16.0 Hz, 1H, CH-CH=C*H*), 4.69 (d, *J* = 7.8 Hz, 1H, C*H*), 3.76 – 3.30 (m, 8H, C*H*$_2$).

^{13}C{^{1}H} NMR (101 MHz, $CDCl_3$): δ 169.9 (*C*=O), 142.9 (Ar *C*), 136.5 (Ar *C*), 132.3 (CH-CH=*C*H), 129.8 (q, *J* = 32.6 Hz, Ar *C*), 128.7 (Ar *C*H), 128.6 (Ar *C*H), 128.0 (Ar *C*H), 127.9 (CH-*C*H=CH), 126.6 (Ar *C*H), 126.1 (q, *J* = 3.7 Hz, Ar *C*H), 124.2 (q, *J* = 272.1 Hz, *C*F$_3$), 66.9 (*C*H$_2$), 66.5 (*C*H$_2$), 52.2 (*C*H), 46.4 (*C*H$_2$), 42.6 (*C*H$_2$).

^{19}F NMR (376 MHz, $CDCl_3$): -62.53 (s, C*F*$_3$).

HRMS: calcd. for $C_{21}H_{20}F_3NO_2Na$ $[M+Na]^+$: 398.1338; found (ESI$^+$): 398.1336.

***ν*$_{max}$ (neat/cm^{-1}):** 700, 845, 920, 1115, 1233, 1430, 1632, 1680, 2857.

(*E*)-2-Benzyl-1-morpholino-4-phenylbut-3-en-1-one (4t)

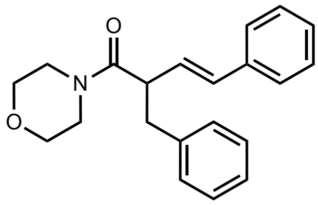

$C_{21}H_{23}NO_2$
MW: 321.42

Synthesised according to **GP-6** from **2u** (43.0 mg, 0.1 mmol), potassium (*E*)-styryl trifluoroborate (42.0 mg, 0.2 mmol), and PTH (1.4 mg, 0.005 mmol) in MeCN (0.5 mL). Purification by column chromatography (silica gel; 0-50% EtOAc in hexane) afforded the product **4t** as a yellow oil (24.9 mg, 0.77 mmol, 77%, >20:1 E/Z ratio).

^{1}H NMR (400 MHz, CDCl$_3$): δ 7.38 – 7.17 (m, 10H, Ar *H*), 6.38 (d, *J* = 15.9 Hz, 1H, CH-CH=C*H*), 6.38 – 6.28 (m, 1H, CH-C*H*=CH), 3.71 – 3.33 (m, 7H, C*H*$_2$), 3.31 – 3.19 (m, 2H, C*H*$_2$), 3.16 – 3.07 (m, 1H, C*H*$_2$), 2.90 (dd, *J* = 13.3, 6.2 Hz, 1H, C*H*).

^{13}C{^{1}H} NMR (101 MHz, CDCl$_3$): δ 171.6 (*C*=O), 139.4 (Ar *C*), 136.8 (Ar *C*), 131.9 (CH-CH=*C*H), 129.4 (Ar *C*H), 128.7 (Ar *C*H), 128.6 (CH-*C*H=CH), 128.6 (Ar *C*H), 127.8 (Ar *C*H), 126.6 (Ar *C*H), 126.4 (Ar *C*H), 66.9 (*C*H$_2$), 66.5 (*C*H$_2$), 48.0 (*C*H$_2$), 46.2 (*C*H$_2$), 42.4 (*C*H$_2$), 40.1 (*C*H).

HRMS: calcd. for $C_{21}H_{23}NO_2Na$ [M+Na]$^+$: 344.1621; found (ESI$^+$): 344.1628.

ν_{max} (neat/cm^{-1}): 571, 584, 696, 743, 968, 1113, 1431, 1494, 1632, 1722, 2854.

(*E*)-1-Morpholino-2-styrylpent-4-en-1-one (4u)

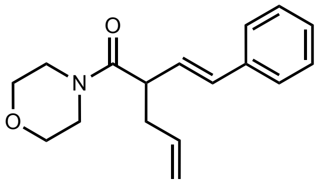

$C_{17}H_{21}NO_2$
MW: 271.36

Synthesised according to **GP-6** from **2v** (38.0 mg, 0.1 mmol), potassium (*E*)-styryl trifluoroborate (42.0 mg, 0.2 mmol), and PTH (1.4 mg, 0.005 mmol) in MeCN (0.5 mL). Purification by column chromatography (silica gel; 0-50% EtOAc in hexane) afforded the product **4u** as a yellow oil (9.0 mg, 0.033 mmol, 33%, >20:1 E/Z ratio).

^{1}H NMR (400 MHz, CDCl$_3$): δ 7.37 – 7.28 (m, 4H, Ar *H*), 7.25 – 7.20 (m, 1H, Ar *H*), 6.42 (d, *J* = 16.0 Hz, 1H, CH-CH=C*H*), 6.22 (dd, *J* = 16.0, 8.5 Hz, 1H, CH-C*H*=CH), 5.81 (ddt, *J* = 17.1, 10.2, 7.0 Hz, 1H, CH_2-*C*H=CH_2), 5.17 – 5.00 (m, 2H, CH_2-CH=CH_2), 3.78 – 3.51 (m, 8H, CH_2), 3.43 (dt, *J* = 8.8, 7.1 Hz, 1H, C*H*), 2.71 – 2.60 (m, 1H, CH_2), 2.47 – 2.32 (m, 1H, CH_2).

^{13}C{^{1}H} NMR (101 MHz, CDCl$_3$): δ 171.5 (*C*=O), 136.8 (Ar *C*), 135.8 (CH_2-*C*H=CH_2), 132.2 (CH-CH=*C*H), 128.8 (Ar *C*H), 128.2 (CH-*C*H=CH), 127.9 (Ar *C*H), 126.4 (Ar *C*H), 117.0 (CH_2-CH=*C*H_2), 67.1 (*C*H_2), 66.9 (*C*H_2), 46.3 (*C*H_2), 46.0 (*C*H), 42.5 (*C*H_2), 37.3 (*C*H_2).

HRMS: calcd. for $C_{17}H_{21}NO_2Na$ $[M+Na]^+$: 294.1465; found (ESI$^+$): 294.1469.

ν_{max} (neat/cm^{-1}): 694, 747, 1115, 1268, 1433, 1636, 1726, 2853, 2918.

(*E*)-1-Morpholino-2-styrylhept-6-en-1-one (4v)

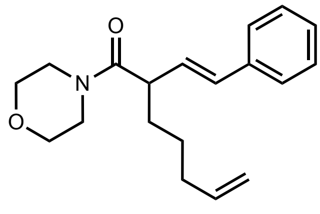

$C_{19}H_{25}NO_2$
MW: 299.41

Synthesised according to **GP-6** from **2x** (40.7 mg, 0.1 mmol), potassium (*E*)-styryl tetrafluoroborate (42.0 mg, 0.2 mmol), and PTH (1.4 mg, 0.005 mmol) in MeCN (0.5 mL). Purification by column chromatography (silica gel; 0-30% EtOAc in hexanes) afforded the product **4v** as a colourless oil (19.8 mg, 0.066 mmol, 66%, >20:1 E/Z ratio).

^{1}H NMR (400 MHz, $CDCl_3$): δ 7.30 – 7.11 (m, 5H, Ar *H*), 6.32 (d, *J* = 16.0 Hz, 1H, CH-CH=C*H*), 6.14 (dd, *J* = 16.0, 8.7 Hz, 1H, CH-C*H*=CH), 5.71 (ddt, *J* = 17.0, 10.2, 6.7 Hz, 1H, CH_2-C*H*=CH_2), 4.92 (dq, *J* = 17.0, 1.7 Hz, 1H, CH=CH_{trans}), 4.86 (ddt, *J* = 10.2, 2.2, 1.2 Hz, 1H, CH=CH_{cis}), 3.70 – 3.41 (m, 7H, 3 × CH_2, CH_{2A}), 3.26 (app dt, *J* = 8.7, 7.1 Hz, 1H, CH_{2B}), 2.07 – 1.93 (m, 2H, CH_2), 1.80 (app ddt, *J* = 13.6, 9.0, 6.7 Hz, 1H), 1.63 – 1.50 (m, 1H), 1.41 – 1.28 (m, 2H).

$^{13}C\{^1H\}$ NMR (101 MHz, $CDCl_3$) δ 172.1 (*C*=O), 138.6 (*C*H=CH_2), 136.8 (Ar *C*), 131.9 (*C*H=CH), 128.9 (CH=*C*H), 128.7 (Ar *C*H), 127.8 (Ar *C*H), 126.4 (Ar *C*H), 114.9 (CH=*C*H_2), 67.1 (*C*H_2), 66.9 (*C*H_2), 46.3 (*C*H_2), 46.0 (*C*H), 42.4 (*C*H_2), 33.8 (*C*H_2), 32.5 (*C*H_2), 26.8 (*C*H_2).

HRMS: calcd. for $C_{19}H_{25}NO_2Na$ $[M+Na]^+$: 322.1777; found (ESI+): 322.1788.

ν_{max} (neat/cm^{-1}): 1116, 1228, 1270, 1433, 1456, 1637, 2855.

(*E*)-6-Chloro-1-morpholino-2-styrylhexan-1-one (4w)

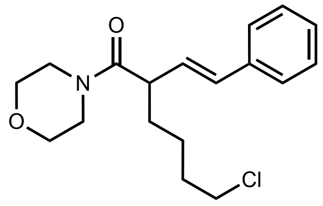

$C_{18}H_{24}ClNO_2$
MW: 359.47

Synthesised according to **GP-6** from **2y** (43.0 mg, 0.1 mmol), potassium (*E*)-styryl tetrafluoroborate (42.0 mg, 0.2 mmol), and PTH (1.4 mg, 0.005 mmol) in MeCN (0.5 mL). Purification by column chromatography (silica gel; 0-50% EtOAc in hexanes) afforded the product **4w** as a yellow oil (14.6 mg, 0.045 mmol, 45%, >20:1 E/Z ratio).

^{1}H NMR (400 MHz, $CDCl_3$): δ 7.39 – 7.28 (m, 4H, Ar *H*), 7.25 – 7.17 (m, 1H, Ar *H*), 6.42 (d, *J* = 16.0 Hz, 1H, CH-CH=C*H*), 6.20 (dd, *J* = 16.0, 8.7 Hz, 1H, CH-C*H*=CH), 3.80 – 3.49 (m, 10H, CH_2), 3.35 (dd, *J* = 8.8, 7.0 Hz, 1H, C*H*), 1.96 – 1.84 (m, 1H, CH_{2A}), 1.84 – 1.74 (m, 2H, CH_2), 1.71 – 1.57 (m, 1H, CH_{2B}), 1.55 – 1.38 (m, 2H, CH_2).

^{13}C NMR (101 MHz, $CDCl_3$): δ 171.8 (*C*=O), 136.7 (Ar *C*), 132.1 (CH-CH=*C*H), 128.9 (Ar *C*H), 128.4 (CH-*C*H=CH), 127.9 (Ar *C*H), 126.4 (Ar *C*H), 67.1 (*C*H_2), 66.9 (*C*H_2), 46.2 (*C*H_2), 46.0 (*C*H), 45.0 (*C*H_2), 42.4 (*C*H_2), 32.6 (*C*H_2), 32.2 (*C*H_2), 24.9 (*C*H_2).

HRMS: calcd. for $C_{18}H_{24}N_2O_4Na$ $[M+Na]^+$: 373.1159; found (ESI$^+$): 373.1150.

Methyl (*E*)-7-(morpholine-4-carbonyl)-9-phenylnon-8-enoate (4x)

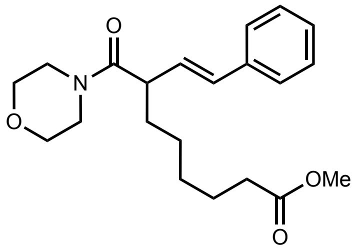

$C_{21}H_{29}NO_4$
MW: 359.47

Synthesised according to **GP-6** from **2z** (43.0 mg, 0.1 mmol), potassium (*E*)-styryl tetrafluoroborate (42.0 mg, 0.2 mmol), and PTH (1.4 mg, 0.005 mmol) in MeCN (0.5 mL) using 390 nm lamps at 25% intensity. Purification by column chromatography (silica gel; 0-50% EtOAc in hexanes) afforded the product **4x** as a yellow oil (18.2 mg, 0.051 mmol, 51%, 8:1 E/Z ratio).

^{1}H NMR (400 MHz, CDCl$_3$): δ 7.42 – 7.14 (m, 5H), 6.39 (d, *J* = 16.0 Hz, 1H, CH-CH=C*H*), 6.20 (dd, *J* = 16.0, 8.7 Hz, 1H, CH-C*H*=CH), 3.82 – 3.51 (m, 11H, 4 × CH_2, CH$_3$), 3.33 (dt, *J* = 8.8, 7.1 Hz, 1H, C*H*), 2.29 (t, *J* = 7.5 Hz, 2H, CH_2CO$_2$Me), 1.94 – 1.79 (m, 1H, CH_{2A}), 1.69 – 1.54 (m, 3H, CH_2, CH_{2B}), 1.42 – 1.25 (m, 4H, 2 × CH_2).

^{13}C NMR (101 MHz, CDCl$_3$): δ 174.3 (**Major** C=O), 172.1 (**Major** C=O), 147.2 (**Minor** Ar *C*), 137.1 (**Minor** Ar *C*H), 136.8 (**Major** Ar *C*), 131.84 (**Major** CH-CH=*C*H), 131.76 (**Minor** CH-*C*H=CH), 130.4 (**Minor** CH-CH=*C*H), 128.85 (Ar *C*H), 128.77 (Ar *C*H), 128.72 (**Major** Ar *C*H), 128.63 (Ar *C*H), 128.35 (**Major** CH-*C*H=CH), 128.25 (Ar *C*H), 127.80 (Ar *C*H), 127.76 (**Major** Ar *C*H), 127.38 (**Minor** Ar *C*H), 126.35 (**Major** Ar *C*H), 67.10 (**Major** *C*H$_2$), 66.86 (**Major** *C*H$_2$), 51.63 (**Major** *C*H$_3$), 46.20 **(Major** *C*H$_2$) 45.96 (**Major** *C*H), 42.40 (**Major** *C*H$_2$), 40.80 (**Minor** CH$_2$), 34.08 (**Major** *C*H$_2$), 32.78 (**Minor** CH$_2$), 32.71 (**Major** *C*H$_2$), 29.25 (**Minor** CH$_2$), 29.11 (**Major** *C*H$_2$), 27.30 (**Minor** CH$_2$), 27.14 (**Major** *C*H$_2$), 24.88 (**Major** *C*H$_2$). *^{13}C NMR peaks that could not be unambiguously assigned to the major or minor isomer have been left unassigned.*

HRMS: calcd. for $C_{21}H_{29}NO_4Na$ [M+Na]$^+$: 382.1989; found (ESI$^+$): 382.1978.

5.3 General Procedure 7 (GP-7): Photochemical α-(Hetero)arylation

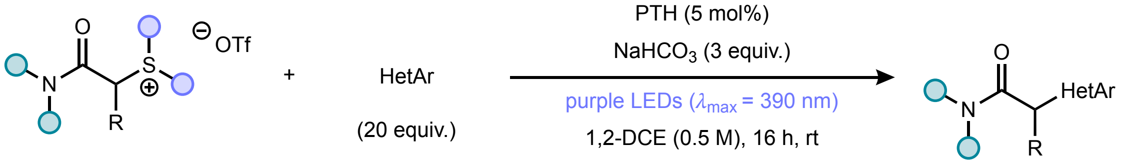

An oven-dried microwave vial was charged with 10-phenylphenothiazine (**PTH**, 1.4 mg, 0.005 mmol), (hetero)arene (2.0 mmol, if solid) and $NaHCO_3$ (25.2 mg, 0.3 mmol) and then sealed and evacuated and back-filled with dinitrogen 3 times. A solution of sulfonium salt (0.1 mmol) in anhydrous DCE (0.2 mL, 0.5 M) was then added, followed by the (hetero)arene (2.0 mmol, if liquid). The sample was stirred under constant irradiation (390 nm Kessil lamp, 5 cm from light source) at rt overnight (for reactions involving sulfonium salt **2u** or **2z** a 48 h reaction time was employed). The crude material was purified directly by column chromatography using eluents given below to afford the pure product.

2-(1-Methyl-1H-pyrrol-2-yl)-1-morpholino-2-phenylethan-1-one (5a)

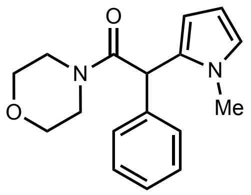

$C_{17}H_{20}N_2O_2$
MW: 284.36

Synthesised according to **GP-4** from **2a** (0.18 mg, 0.1 mmol), 1-methyl-1*H*-pyrrole (0.18 mL, 2.0 mmol), and PTH (1.4 mg, 0.005 mmol) in 1,2-DCE (0.2 mL). Purification by column chromatography (silica gel; 0-30% EtOAc in hexanes) afforded the product **5a** as a yellow oil (15.6 mg, 0.055 mmol, 55%, 5.7:1 r.r.).

^{1}H NMR (400 MHz, CDCl$_3$): δ 7.35 – 7.22 (m, 3H, ***Major*** Ar *H*), 7.35 – 7.15 (m, 5H, ***Minor*** Ar *H*), 7.19 – 7.15 (m, 2H, ***Major*** Ar *H*), 6.58 (dd, *J* = 2.8, 1.8 Hz, 1H, ***Major*** Ar *H*),), 6.53 (t, *J* = 2.4 Hz, 1H, ***Minor*** Ar *H*), 6.07 (dd, *J* = 3.7, 2.8 Hz, 1H, ***Major*** Ar *H*), 5.99 (dd, *J* = 2.8, 1.8 Hz, 1H, ***Minor*** Ar *H*), 5.91 (dd, *J* = 3.7, 1.8 Hz, 1H, ***Major*** Ar *H*), 5.09 (s, 1H, ***Major*** C*H*), 5.05 (s, 1H, ***Minor*** C*H*), 3.77 – 3.60 (m, 4H, 2 x ***Major*** CH_2), 3.77 – 3.60 (m, 4H, 2 x ***Minor*** CH_2), 3.58 (s, 3H, ***Minor*** CH_3), 3.52 – 3.38 (m, 4H, 2 x ***Major*** CH_2), 3.52 – 3.38 (m, 4H, 2 x ***Minor*** CH_2), 3.37 (s, 3H, ***Major*** CH_3).

^{13}C{^{1}H} NMR (101 MHz, CDCl$_3$) δ 171.5 (***Minor*** *C*=O), 169.7 (***Major*** *C*=O), 140.6 (***Minor*** Ar *C*), 137.8 (***Major*** Ar *C*), 129.4 (***Major*** Ar *C*), 129.1 (***Major*** Ar *C*H), 128.7 (***Major*** Ar *C*H), 128.6 (***Minor*** Ar *C*H), 128.5 (***Minor*** Ar *C*H), 127.4 (***Major*** Ar *C*H), 126.9 (***Minor*** Ar *C*H), 122.9 (***Major*** Ar *C*H), 122.0 (***Minor*** Ar *C*H), 121.9 (***Minor*** Ar *C*), 120.7 (***Minor*** Ar *C*H), 109.5 (***Major*** Ar *C*H), 108.8 (***Minor*** Ar *C*H), 107.0 (***Major*** Ar *C*H), 67.0 (***Minor*** *C*H_2), 66.9 (***Major*** *C*H_2), 66.6 (***Minor*** *C*H_2), 66.5 (***Major*** *C*H_2), 48.0 (***Major*** *C*H), 47.8 (***Minor*** *C*H), 46.6 (***Major*** *C*H_2), 42.8 (***Major*** *C*H_2), 42.6 (***Minor*** *C*H_2), 41.0 (***Minor*** *C*H_2), 36.4 (***Minor*** *C*H_3) 34.2 (***Major*** *C*H_3);

HRMS: calcd. for $C_{17}H_{21}N_2O_2$ [M+H]$^+$: 285.1598; found (ESI$^+$): 285.1602.

ν_{max} (neat/cm^{-1}): 708, 1115, 1458, 1492, 1648, 2090, 2927, 2960.

*The regiochemistry of **5a** was confirmed by HMBC analysis:*

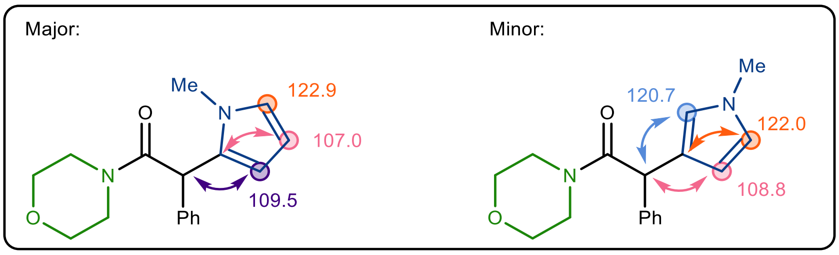

2-(1,2-Dimethyl-1H-indol-3-yl)-1-morpholino-2-phenylethan-1-one (5b)

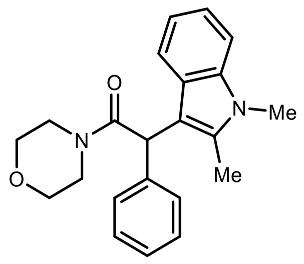

$C_{22}H_{24}N_2O_2$
MW: 348.45

Synthesised according to **GP-7** from **2a** (41.5 mg, 0.1 mmol), PTH (1.4 mg, 0.005 mmol), and 1,2-dimethyl-1*H*-indole (290 mg, 2.0 mmol) in 1,2-DCE (0.2 mL). Purification by column chromatography (silica gel; 0-50% EtOAc in hexanes) afforded the product **5b** as an off yellow oil (19.8 mg, 0.057 mmol, 57%).

^{1}H NMR (400 MHz, $CDCl_3$): δ 7.62 – 7.55 (m, 1H, Ar C*H*), 7.36 – 7.16 (m, 7H, Ar C*H*), 7.14 – 7.05 (m, 1H, Ar C*H*), 5.34 (s, 1H, C*H*), 3.89 – 3.30 (m, 10H, CH_2 + CH_3), 3.18 – 3.06 (m, 1H, CH_2), 2.38 (s, 3H, CH_3).

^{13}C{^{1}H} NMR (101 MHz, $CDCl_3$): δ 171.3 (*C*=O), 140.2 (Ar *C*), 136.7 (Ar *C*), 134.0 (Ar *C*), 129.0 (Ar *C*H), 128.3 (Ar *C*H), 127.0 (Ar *C*), 126.7 (Ar *C*H), 121.1 (Ar *C*H), 119.6 (Ar *C*H), 118.5 (Ar *C*H), 108.8 (Ar *C*H), 108.3 (Ar *C*), 67.1 (*C*H_2), 66.4 (*C*H_2), 46.6 (*C*H_2), 46.4 (*C*H), 42.6 (*C*H_2), 29.7 (*C*H_3), 10.8 (*C*H_3).

HRMS: calcd. for $C_{16}H_{22}NO_2S$ $[M+H]^+$: 349.1911; found (ESI$^+$): 349.1915.

ν_{max} (neat/cm^{-1}): 652, 1021, 1259, 1422, 1469, 1651, 2870, 2941.

2-(1-Methyl-1H-pyrrol-2-yl)-1-morpholino-2-(4-(trifluoromethyl)phenyl)ethan-1-one (5c)

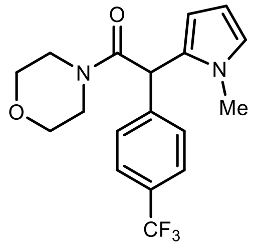

$C_{18}H_{19}F_3N_2O_2$
MW: 352.36

Synthesised according to **GP-7** from **2l** (48 mg, 0.1 mmol), 1-methyl-1*H*-pyrrole (0.18 mL, 2.0 mmol), and PTH (1.4 mg, 0.005 mmol) in 1,2-DCE (0.2 mL). Purification by column chromatography (silica gel; 0-40% EtOAc in hexanes) afforded the product **5c** as a yellow oil (15.6 mg, 0.044 mmol, 44%, 9:1 r.r.).

^{1}H NMR (400 MHz, CDCl$_3$): δ 7.60 – 7.48 (m, 2H, **Major** Ar *H* + **Minor** Ar *H*), 7.34 – 7.26 (m, 1H, **Major** Ar *H* + **Minor** Ar *H*), 6.62 – 6.46 (m, 1H, **Major** Pyrrole *H* + **Minor** Pyrrole *H*), 6.12 – 6.08 (m, 1H, **Major** Pyrrole *H*), 6.04 – 5.98 (m, 1H, **Major** Pyrrole *H* + **Minor** Pyrrole *H*), 5.14 (s, 1H, **Major** C*H*), 5.11 (s, 1H, **Minor** Pyrrole *H*), 3.85 – 3.32 (m, 11H, **Major** CH_2 + **Minor** CH_2 + **Major** CH_3 + **Minor** CH_3).

^{13}C{^{1}H} NMR (126 MHz, CDCl$_3$): δ 168.9 (**Major** *C*=O), 142.1 (**Major** Ar *C*), 129.6 (**Major** Ar *C*), 129.7 (d, *J* = 32.2 Hz, **Major** Ar *C*), 128.1 (**Major** Ar *C*H), 125.6 (q, *J* = 3.8 Hz, **Major** CF$_3$-Ar C- Ar *C*H), 123.4 (q, *J* = 270.6 Hz, **Major** *C*F$_3$),123.3 (**Major** Ar *C*H), 109.6 (**Major** Ar *C*H), 107.3 (**Major** Ar *C*H), 66.9 (**Major** *C*H$_2$), 66.5 (**Major** *C*H$_2$), 47.7 (**Major** *C*H), 46.6 (**Major** *C*H$_2$), 42.8 (**Major** *C*H$_2$), 34.2 (**Major** *C*H$_3$).

^{19}F NMR (376 MHz, CDCl$_3$): δ -62.52 (s, C*F$_3$*).

HRMS: calcd. for $C_{18}H_{20}F_3N_2O_2$ [M+H]$^+$: 353.1471; found (ESI$^+$): 353.1479.

ν_{max} (neat/cm^{-1}): 713, 1119, 1477, 1484, 1660, 2091, 2944, 2972.

2-(1,2-Dimethyl-1H-indol-3-yl)-1-morpholino-2-(4-(trifluoromethyl)phenyl)ethan-1-one (5d)

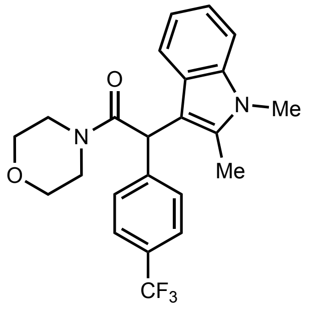

$C_{23}H_{23}F_3N_2O_2$
MW: 416.44

Synthesised according to **GP-7** from **2l** (48 mg, 0.1 mmol), PTH (1.4 mg, 0.005 mmol), and 1,2-dimethyl-1*H*-indole (290 mg, 2.0 mmol) in 1,2-DCE (0.2 mL). Purification by column chromatography (silica gel; 0-50% EtOAc in hexanes) afforded the product **5d** as an off yellow oil (26.2 mg, 0.063 mmol, 63%).

^{1}H NMR (400 MHz, $CDCl_3$): δ 7.57 – 7.47 (m, 3H, Ar C*H*), 7.33 – 7.28 (m, 3H, Ar C*H*), 7.22 – 7.18 (m, 1H, Ar C*H*), 7.12 – 7.05 (m, 1H, Ar C*H*), 5.31 (s, 1H, C*H*), 3.89 – 3.81 (m, 1H, CH_{2A}), 3.72 – 3.67 (m, 4H, CH_{2B}, CH_3), 3.62 – 3.49 (m, 2H, CH_2), 3.46 – 3.24 (m, 3H, CH_2, CH_{2A}), 3.04 – 2.96 (m, 1H, CH_{2B}), 2.35 (s, 3H, CH_3).

^{13}C{^{1}H} NMR (126 MHz, $CDCl_3$): δ 170.6 (*C*=O), 144.4 (Ar *C*), 136.8 (Ar *C*), 134.0 (Ar *C*), 129.5 (Ar *C*H), 128.9 (q, J = 32.5 Hz, CF_3-Ar *C*), 126.7 (Ar *C*), 125.2 (q, J = 3.8 Hz, CF_3-Ar C-Ar *C*H), 124.4 (q, J = 271.4 Hz, *C*F_3), 121.5 (Ar *C*H), 120.0 (Ar *C*H), 118.3 (Ar *C*H), 109.1 (Ar *C*H), 107.3 (Ar *C*), 67.1 (*C*H_2), 66.3 (*C*H_2), 46.7 (*C*H_2), 46.5 (*C*H), 42.7 (*C*H_2), 29.8 (*C*H_3), 10.7 (*C*H_3).

^{19}F NMR (376 MHz, $CDCl_3$): δ -62.42 (s, CF_3).

HRMS: calcd. for $C_{23}H_{24}F_3N_2O_2$ $[M+H]^+$: 417.1784; found (ESI$^+$): 417.1788.

ν_{max} (neat/cm^{-1}): 647, 1018, 1255, 1431, 1471, 1644, 2854, 2923.

2-(1-Methyl-1H-pyrrol-2-yl)-1-morpholino-3-phenylpropan-1-one (5e)

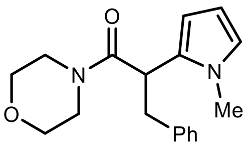

$C_{18}H_{22}N_2O_2$
MW: 298.39

Synthesised according to **GP-7** from **2u** (42.9 mg, 0.1 mmol), PTH (1.4 mg, 0.005 mmol), and N-methylpyrrole (0.18 mL, 2.0 mmol) in 1,2-DCE (0.2 mL). Purification by column chromatography (silica gel; 0-50% EtOAc in hexane) afforded the product **5e** as an off yellow oil (9.0 mg, 0.030 mmol, 30%, 5:1 r.r.).

^{1}H NMR (400 MHz, CDCl$_3$): δ 7.25 – 7.15 (m, 3H, **Major** Ar *H* + **Minor** Ar *H*), 7.11 – 7.03 (m, 2H, **Major** Ar *H* + **Minor** Ar *H*), 6.47 (t, *J* = 2.3 Hz, 1H, **Major** Ar *H* + **Minor** Ar *H*), 6.10 – 6.02 (m, 2H, **Major** Ar *H* + **Minor** Ar *H*), 3.94 (dd, *J* = 8.1, 6.6 Hz, 1H, **Major** C*H* + **Minor** C*H*), 3.69 – 3.35 (m, 6H, **Major** CH_2 + **Minor** CH_2 + **Major** CH_2CH + **Minor** CH_2CH), 3.23 (s, 3H, **Major** CH_3 + **Minor** CH_3), 3.20 – 3.02 (m, 4H, **Major** CH_2 + **Minor** CH_2 + **Major** CH_2CH + **Minor** CH_2CH).

^{13}C{^{1}H} NMR (101 MHz, CDCl$_3$): δ 170.6 (**Major** *C*=O), 139.8 (**Major** Ar *C*), 130.3 (**Major** Ar *C*), 129.4 (**Major** Ar *C*H), 128.5 (**Major** Ar *C*H), , 126.6 (**Major** Ar *C*H), 122.2 (**Major** Ar *C*H), 107.8 (**Major** Ar *C*H), 107.5 (**Major** Ar *C*H), 66.8 (**Major** *C*H_2), 66.2 (**Major** *C*H_2), 46.1 (**Major** *C*H_2), 43.1 (**Major** *C*H_2), 42.7 (**Major** *C*H_2), 40.0 (**Major** *C*H_2CH), 33.7 (**Major** *C*H_3).

HRMS: calcd. for $C_{18}H_{23}N_2O_2$ $[M+H]^+$: 299.1754; found (ESI$^+$): 299.1759.

ν_{max} (neat/cm^{-1}): 712, 1124, 1471, 1503, 1651, 2081, 2939, 2989.

1-Methyl-3-(1-morpholino-1-oxo-3-phenylpropan-2-yl)-1H-indole-2-carbaldehyde (5f)

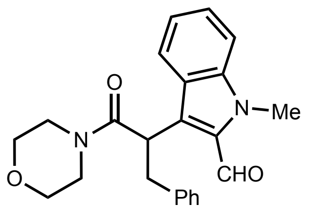

$C_{23}H_{24}N_2O_3$
MW: 376.46

Synthesised according to **GP-7** from **2u** (42.9 mg, 0.1 mmol), PTH (1.4 mg, 0.005 mmol), and 1-methyl-1*H*-indole-2-carbaldehyde (318 mg, 2.0 mmol) in 1,2-DCE (0.2 mL). Purification by column chromatography (silica gel; 0-40% EtOAc in hexane) afforded the product **5f** as an off yellow oil (21.0 mg, 0.056 mmol, 56%).

^{1}H NMR (500 MHz, $CDCl_3$): δ 9.79 (s, 1H, C*H*O), 8.02 – 7.96 (m, 1H, Ar *H*), 7.47 – 7.34 (m, 2H, Ar *H*), 7.20 – 7.14 (m, 4H, Ar *H*), 7.02 – 6.93 (m, 2H, Ar *H*), 4.74 (dd, *J* = 8.0, 6.5 Hz, 1H, CH_2CH), 4.00 (s, 3H, CH_3), 3.72 – 3.52 (m, 4H, CH_2), 3.52 – 3.08 (m, 5H, CH_2 + CH_2CH), 3.02 – 2.91 (m, 1H, CH_2).

^{13}C{^{1}H} NMR (126 MHz, $CDCl_3$): δ 181.8 (*C*HO), 170.8 (*C*=O), 139.7 (Ar *C*), 139.6 (Ar *C*), 130.9 (Ar *C*), 129.4 (Ar *C*H), 128.4 (Ar *C*H), 127.4 (Ar *C*H), 126.6 (Ar *C*H), 125.0 (Ar *C*), 125.0 (Ar *C*), 122.7 (Ar *C*H), 121.3 (Ar *C*H), 110.5 (Ar *C*H), 66.9 (CH_2), 66.3 (CH_2), 46.3 (CH_2), 42.8 (*C*H), 42.3 (CH_2-CH), 40.3 (CH_2), 31.5 (CH_3).

HRMS: calcd. for $C_{23}H_{25}N_2O_3$ $[M+H]^+$: 377.1860; found (ESI$^+$): 377.1863.

ν_{max} (neat/cm^{-1}): 647, 1021, 1213, 1443, 1468, 1612, 1657, 2854, 2925.

2-(5-Methylfuran-2-yl)-1-morpholino-3-phenylpropan-1-one (5g)

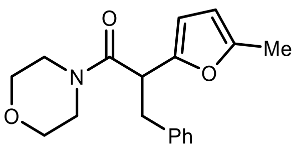

$C_{18}H_{21}NO_3$
MW: 299.37

Synthesised according to **GP-7** from **2u** (42.9 mg, 0.1 mmol), PTH (1.4 mg, 0.005 mmol), and 2-methylfuran (164 mg, 2.0 mmol) in 1,2-DCE (0.2 mL). Purification by column chromatography (silica gel; 0-50% EtOAc in hexane) afforded the product **5g** as an off yellow oil (16.7 mg, 0.056 mmol, 56%, >20:1 r.r.).

^{1}H NMR (400 MHz, $CDCl_3$): δ 7.30 – 7.25 (m, 2H, Ar *H*), 7.24 – 7.17 (m, 3H, Ar *H*), 6.06 (d, *J* = 3.1 Hz, 1H, Ar *H*), 5.90 (dd, *J* = 3.0, 1.2 Hz, 1H, Ar *H*), 4.13 (dd, *J* = 9.0, 5.8 Hz, 1H, C*H*), 3.67 – 3.34 (m, 7H, CH_{2A} + CH_2CH), 3.34 – 3.26 (m, 1H, CH_{2A}), 3.25 – 3.18 (m, 1H, CH_{2B}), 3.15 (dd, *J* = 13.4, 5.9 Hz, 1H, CH_2CH), 2.26 (d, *J* = 1.0 Hz, 3H, CH_3).

^{13}C{^{1}H} NMR (101 MHz, $CDCl_3$): δ 169.4 (*C*=O), 151.3 (Furan *C*), 151.0 (Furan *C*), 139.4 (Ar *C*), 129.2 (Ar *C*H), 128.5 (Ar *C*H), 126.6 (Ar *C*H), 107.5 (Furan *C*H), 106.5 (Furan *C*H), 66.9 (CH_2), 66.5 (CH_2), 46.4 (CH_2), 43.8 (*C*H), 42.7 (CH_2), 38.0 (CH_2), 13.7 (CH_3).

HRMS: calcd. for $C_{18}H_{22}NO_3$ $[M+H]^+$: 300.1594; found (ESI$^+$): 300.1597.

ν_{max} (neat/cm^{-1}): 646, 1022, 1268, 1433, 1494, 1627, 1716, 2857, 2962.

2-(4-Methylfuran-2-yl)-1-morpholino-3-phenylpropan-1-one (5h)

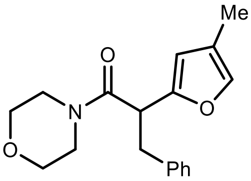

$C_{18}H_{21}NO_3$
MW: 299.37

Synthesised according to **GP-7** from **2u** (42.9 mg, 0.1 mmol), PTH (1.4 mg, 0.005 mmol), and 3-methylfuran (164 mg, 2.0 mmol) in 1,2-DCE (0.2 mL). Purification by column chromatography (silica gel; 0-40% EtOAc in Hexane) afforded the product **5h** as an off yellow oil (15.8 mg, 0.053 mmol, 53%, 6:1 r.r.).

^{1}H NMR (500 MHz, $CDCl_3$): δ 7.24 – 7.14 (m, 4H, **Major** Ar *H* + **Minor** Ar *H*), 7.09 – 6.99 (m, 2H, **Major** Ar *H* + **Minor** Ar *H* + **Major** Furan C*H* + **Minor** Furan C*H*), 6.11 (d, *J* = 1.8 Hz, 1H, **Major** Furan C*H*), 6.07 (s, 1H, **Minor** Furan C*H*), 4.12 (dd, *J* = 9.1, 5.9 Hz, 1H, **Minor** C*H*), 3.97 (dd, *J* = 8.6, 6.5 Hz, 1H, **Major** C*H*), 3.83 – 3.75 (m, 1H, **Major** CH_{2A} + **Minor** CH_{2A}), 3.66 – 3.43 (m, 3H, **Major** CH_2, CH_{2B} + **Minor** CH_2, CH_{2B}), 3.42 – 3.32 (m, 2H, **Major** PhCH_2 + **Minor** PhCH_2 + **Major** CH_{2A} + **Minor** CH_{2A}), 3.30 – 3.22 (m, 2H, **Major** CH_2 + **Minor** CH_2), 3.16 – 3.07 (m, 2H, **Major** CH_2 + **Major** CH_2 + **Minor** CH_2), 3.00 (s, 1H, **Minor** CH_2), 2.94 (s, 1H, **Minor** CH_2), 1.67 (s, 3H, **Major** CH_3), 1.58 (s, 3H, **Minor** CH_3).

$^{13}C\{^1H\}$ NMR (126 MHz, $CDCl_3$): δ 169.1 (**Major** *C*=O), 146.6 (**Major** Furan *C*), 141.1 (**Major** Furan *C*), 139.6 (**Major** Ar *C*), 129.3 (**Major** Ar *C*H), 128.3 (**Major** Ar *C*H), 126.4 (**Major** Ar *C*H), 116.3 (**Major** Furan *C*H), 113.3 (**Major** Furan *C*H), 67.1 (**Major** CH_2), 66.5 (**Major** CH_2), 46.1 (**Major** CH_2), 43.7 (**Major** CH_2-*C*H), 42.8 (**Major** CH_2), 37.6 (**Major** Ar-CH_2), 9.5 (**Major** Furan-CH_3).

HRMS: calcd. for $C_{18}H_{22}NO_3$ $[M+H]^+$: 300.1594; found (ESI^+): 300.1598.

v_{max} (neat/cm^{-1}): 646, 1028, 1269, 1441, 1494, 1630, 1759, 2854, 2960.

2-(5-Acetylfuran-2-yl)-1-morpholino-3-phenylpropan-1-one (5i)

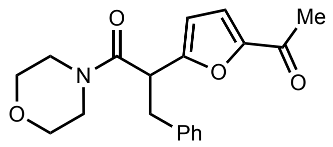

$C_{19}H_{21}NO_4$

MW: 327.38

Synthesised according to **GP-7** from **2u** (42.9 mg, 0.1 mmol), PTH (1.4 mg, 0.005 mmol), and 1-(furan-2-yl)ethan-1-one (220 mg, 2.0 mmol) in 1,2-DCE (0.2 mL). Purification by column chromatography (silica gel; 0-45% EtOAc in hexane) afforded the product **5i** as an off yellow oil (18.6 mg, 0.057 mmol, 57%, >20:1 r.r.).

^{1}H NMR (500 MHz, $CDCl_3$): δ 7.38 – 7.24 (m, 5H, Ar *H*), 7.19 (d, *J* = 3.6 Hz, 1H, Furan *H*), 6.54 (d, *J* = 3.6 Hz, 1H, Furan *H*), 4.37 (dd, *J* = 9.7, 5.6 Hz, 1H, C*H*), 3.75 – 3.40 (m, 7H, CH_2+ CH_2CH), 3.31 – 3.22 (m, 2H, CH_2 + Ar-CH_2), 3.17 – 3.08 (m, 1H, CH_2CH), 2.48 (s, 3H, CH_3).

^{13}C{^{1}H} NMR (126 MHz, $CDCl_3$): δ 186.2 (*C*=O), 168.4 (*C*=O), 158.4 (Furan *C*), 151.6 (Furan *C*), 138.2 (Ar *C*), 129.2 (Ar *C*H), 128.8 (Ar *C*H), 127.1 (Ar *C*H), 119.4 (Furan *C*H), 109.8 (Furan *C*H), 66.7 (*C*H_2), 66.4 (*C*H_2), 46.5 (*C*H_2), 43.7 (CH_2-*C*H), 42.7 (*C*H_2), 38.6 (*C*H_2CH), 26.0 (*C*H_3).

HRMS: calcd. for $C_{19}H_{22}NO_4$ $[M+H]^+$: 328.1543; found (ESI^+): 328.1538.

ν_{max} (neat/cm^{-1}): 629, 1026, 1232, 1454, 1509, 1644, 1672, 2856, 2923.

2-(Benzofuran-2-yl)-1-morpholino-3-phenylpropan-1-one (5j)

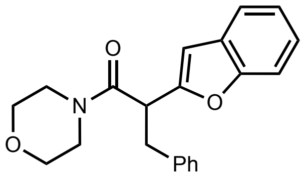

$C_{21}H_{21}NO_3$
MW: 335.40

Synthesised according to **GP-7** from **2u** (42.9 mg, 0.1 mmol), PTH (1.4 mg, 0.005 mmol), and benzofuran (236 mg, 2.0 mmol) in 1,2-DCE (0.2 mL). Purification by column chromatography (silica gel; 0-45% EtOAc in hexane) afforded the product **5j** as an off yellow oil (14.4 mg, 0.043 mmol, 43%, 8:1 r.r.).

^{1}H NMR (500 MHz, $CDCl_3$): δ 7.59 – 7.48 (m, 1H, **Major** Ar *H* + **Minor** Ar *H*), 7.46 – 7.40 (m, 1H, **Major** Ar *H* + **Minor** Ar *H*), 7.31 – 7.17 (m, 9H, **Major** Ar *H* + **Minor** Ar *H*), 6.60 (s, 1H, **Major** Ar *H*), 6.30 (s, 1H, **Minor** Ar *H*), 4.36 – 4.25 (m, 1H, **Major** C*H* + **Minor** C*H*), 3.63 – 3.15 (m, 10H, **Major** CH_2 + **Major** CH_2CH + **Minor** CH_2 + **Minor** CH_2CH).

^{13}C{^{1}H} NMR (126 MHz, $CDCl_3$): δ 168.8 (**Major** *C*=O), 155.9 (**Major** Ar *C*), 154.6 (**Major** Ar *C*), 139.0 (**Major** Ar *C*), 129.2 (**Major** Ar *C*H), 129.1 (**Minor** Ar *C*H), 128.7 (**Major** Ar *C*H), 128.5 (**Minor** Ar *C*H), 127.7 (**Major** Ar *C*), 127.5 (**Minor** Ar *C*), 126.8 (**Major** Ar *C*H), 124.1 (**Major** Ar *C*H), 123.5 (**Minor** Ar *C*H), 123.0 (**Major** Ar *C*H), 122.6 (**Minor** Ar *C*H), 121.0 (**Major** Ar *C*H), 120.7 (**Minor** Ar *C*H), 111.2 (**Major** Ar *C*H), 110.8 (**Minor** Ar *C*H), 104.1 (**Minor** Ar *C*H), 103.9 (**Major** Ar *C*H), 66.8 (**Major** CH_2), 66.5 (**Major** CH_2), 47.5 (**Minor** CH_2), 46.5 (**Major** CH_2), 44.2 (**Major** *C*H), 42.8 (**Major** CH_2), 42.6 (**Minor** CH_2), 38.0 (**Major** CH_2CH).

HRMS: calcd. for $C_{21}H_{22}NO_3$ $[M+H]^+$: 336.1594; found (ESI^+): 336.1599.

v_{max} (neat/cm^{-1}): 647, 1024, 1236, 1453, 1583, 1644, 1783, 2855, 2922.

1-Morpholino-3-phenyl-2-(thiophen-2-yl)propan-1-one (5k)

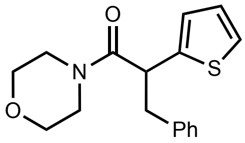

$C_{17}H_{19}NO_2S$
MW: 301.40

Synthesised according to **GP-7** from **2u** (42.9 mg, 0.1 mmol), PTH (1.4 mg, 0.005 mmol), and thiophene (168 mg, 2.0 mmol) in 1,2-DCE (0.2 mL). Purification by column chromatography (silica gel; 0-45% EtOAc in hexane) afforded the product **5k** as an off yellow oil (12.0 mg, 0.040 mmol, 40%, 9:1 r.r.).

^{1}H NMR (500 MHz, CDCl$_3$): δ 7.29 – 7.26 (m, 1H, Ar *H*), 7.24 – 7.14 (m, 5H, Ar *H* + Thiophene *H*), 6.92 (dd, *J* = 5.1, 3.5 Hz, 1H, Thiophene *H*), 6.86 (dd, *J* = 3.6, 1.2 Hz, 1H, Thiophene *H*), 4.32 (dd, *J* = 8.7, 6.1 Hz, 1H, C*H*), 3.60 – 3.42 (m, 5H, CH_2 + CH_2CH), 3.39-3.23 (m, 3H, CH_2), 3.17-3.05 (m, 2H, CH_2).

^{13}C{^{1}H} NMR (126 MHz, CDCl$_3$): δ 170.7 (*C*=O), 142.1 (Thiophene *C*), 139.3 (Ar *C*), 129.3 (Thiophene *C*H), 128.5 (Ar *C*H), 126.7 (Ar *C*H), 126.7 (Thiophene *C*H), 125.3 (Thiophene *C*H), 124.9 (Ar *C*H), 66.8 (*C*H$_2$), 66.4 (*C*H$_2$), 46.5 (*C*H$_2$), 45.4 (*C*H), 42.7 (*C*H$_2$), 42.4 (*C*H$_2$CH).

HRMS: calcd. for $C_{17}H_{20}NO_2S$ [M+H]$^+$: 302.1209; found (ESI$^+$): 302.1201.

v_{max} (neat/cm^{-1}): 639, 1019, 1233, 1452, 1528, 1638, 1785, 2853, 2921.

2-(5-Methylthiophen-2-yl)-1-morpholino-3-phenylpropan-1-one (5l)

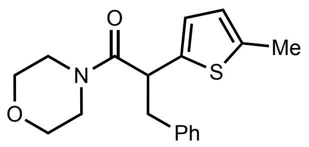

$C_{18}H_{21}NO_2S$
MW: 315.43

Synthesised according to **GP-7** from **2u** (42.9 mg, 0.1 mmol), PTH (1.4 mg, 0.005 mmol), and 2-methylthiophene (196 mg, 2.0 mmol) in 1,2-DCE (0.2 mL). Purification by column chromatography (silica gel; 0-45% EtOAc in hexane) afforded the product **5l** as an off yellow oil (13.2 mg, 0.042 mmol, 42%, 19:1 r.r.).

^{1}H NMR (500 MHz, $CDCl_3$): δ 7.29 – 7.26 (m, 1H, Ar *H*), 7.25 – 7.14 (m, 4H, Ar *H*), 6.61 (d, *J* = 3.4 Hz, 1H, Thiophene *H*), 6.55 (dt, *J* = 3.4, 1.2 Hz, 1H, Thiophene *H*), 4.22 (dd, *J* = 8.8, 5.9 Hz, 1H, C*H*), 3.60 – 3.26 (m, 8H, CH_2 + CH_2CH), 3.18 – 3.02 (m, 2H, CH_2), 2.43 (s, 3H, CH_3).

^{13}C{^{1}H} NMR (126 MHz, $CDCl_3$): δ 170.8 (*C*=O), 139.6 (Ar *C*), 139.4 (Thiophene *C*), 139.4 (Thiophene *C*), 129.3 (Ar *C*H), 128.5 (Ar *C*H), 126.7 (Ar *C*H), 125.0 (Thiophene *C*H), 124.7 (Thiophene *C*H), 66.8 (*C*H_2), 66.4 (*C*H_2), 46.5 (*C*H_2), 45.6 (*C*H), 42.7 (*C*H_2), 42.2 (*C*H_2CH), 15.5 (*C*H_3).

HRMS: calcd. for $C_{18}H_{22}NO_2S$ $[M+H]^+$: 316.1366; found (ESI$^+$): 316.1371.

v_{max} (neat/cm^{-1}): 660, 1019, 1232, 1453, 1643, 1711, 1785, 2855, 2960.

1-Morpholino-3-phenyl-2-(5-phenylthiophen-2-yl)propan-1-one (5m)

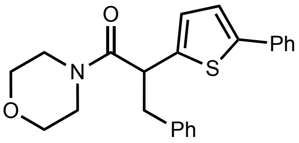

$C_{23}H_{23}NO_2S$

MW: 377.50

Synthesised according to **GP-7** from **2u** (42.9 mg, 0.1 mmol), PTH (1.4 mg, 0.005 mmol), and 2-phenylthiophene (320 mg, 2.0 mmol) in 1,2-DCE (0.2 mL). Purification by column chromatography (silica gel; 0-45% EtOAc in hexane) afforded the product **5m** as an off yellow oil (16.5 mg, 0.044 mmol, 44%, >20:1 r.r.).

^{1}H NMR (500 MHz, $CDCl_3$): δ 7.60 – 7.54 (m, 2H, Ar *H*), 7.39 – 7.33 (m, 2H, Ar *H*), 7.31 – 7.26 (m, 2H, Ar *H*), 7.26 – 7.21 (m, 2H, Ar *H*), 7.20 – 7.16 (m, 2H, Ar *H*), 7.13 (d, *J* = 3.6 Hz, 1H, Thiophene *H*), 6.81 (d, *J* = 3.6 Hz, 1H, Thiophene *H*), 4.30 (dd, *J* = 8.7, 6.1 Hz, 1H, C*H*), 3.61 – 3.27 (m, 8H, CH_2+ CH_2CH), 3.18 – 3.07 (m, 2H, CH_2).

^{13}C{^{1}H} NMR (126 MHz, $CDCl_3$): δ 170.4 (*C*=O), 143.7 (Ar *C*), 141.4 (Thiophene *C*), 139.0 (Thiophene *C*), 134.3 (Ar *C*), 129.2 (Ar *C*H), 128.9 (Ar *C*H), 128.5 (Ar *C*H), 127.4 (Ar *C*H), 126.7 (Ar *C*H), 126.2 (Thiophene *C*H), 125.6 (Ar *C*H), 122.4 (Thiophene *C*H), 66.7 (*C*H_2), 66.3 (*C*H_2), 46.4 (*C*H_2), 45.6 (*C*H), 42.6 (*C*H_2), 42.2 (*C*H_2CH).

HRMS: calcd. for $C_{23}H_{24}NO_2S$ [M+H]$^+$: 378.1522; found (ESI$^+$): 378.1525.

v_{max} (neat/cm^{-1}): 643, 1029, 1232, 1454, 1641, 2855, 2962.

2-(4-Acetylthiophen-2-yl)-1-morpholino-3-phenylpropan-1-one (5n)

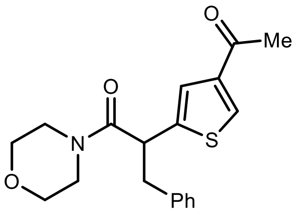

$C_{18}H_{21}NO_3S$
MW: 343.44

Synthesised according to **GP-7** from **2u** (42.9 mg, 0.1 mmol), PTH (1.4 mg, 0.005 mmol), and 1-(thiophen-3-yl)ethan-1-one (252 mg, 2.0 mmol) in 1,2-DCE (0.2 mL). Purification by column chromatography (silica gel; 0-50% EtOAc in hexane) afforded the product **5n** as an off yellow oil (14.0 mg, 0.041 mmol, 41%, 5.2:1 r.r.).

^{1}H NMR (500 MHz, $CDCl_3$): δ 7.38 – 7.27 (m, 4H, **Major** Ar *H* + **Minor** Ar *H* + **Major** Thiophene C*H* + **Minor** Thiophene C*H*), 7.26 – 7.23 (m, 1H, **Major** Ar *H* + **Minor** Ar *H* + **Major** Thiophene C*H*), 7.06 (d, *J* = 5.4 Hz, 1H, **Minor** Thiophene C*H*), 5.80 (dd, *J* = 9.7, 5.5 Hz, 1H, **Major** CH_2CH), 4.35 (dd, *J* = 8.3, 5.9 Hz, 1H, **Minor** CH_2CH), 3.88 – 3.75 (m, 1H, **Minor** CH_2), 3.69 – 3.60 (m, 1H, **Major** CH_2 + **Minor** CH_2), 3.60 – 3.22 (m, 8H, **Major** CH_2 + **Major** CH_2 + **Minor** CH_2), 3.10 (dd, *J* = 12.8, 5.4 Hz, 1H, **Major** CH_2 + **Minor** CH_2), 3.04 (s, 1H, **Minor** CH_2), 2.98 (s, 1H, **Minor** CH_2), 2.95 – 2.88 (m, 1H, **Major** CH_2), 2.58 (s, 3H, **Minor** CH_3), 2.47 (s, 3H, **Major** CH_3).

$^{13}C\{^1H\}$ NMR (126 MHz, $CDCl_3$): δ 195.2 (**Major** *C*=O), 171.0 (**Major** *C*=O), 151.4 (**Major** Ar *C*), 151.3 (**Minor** Ar *C*), 138.6 (**Major** Ar *C*), 135.6 (**Major** Ar *C*), 129.5 (**Major** Thiophene *C*H), 129.0 (**Minor** Ar *C*H), 128.7 (**Minor** Ar *C*H), 128.5 (**Major** Thiophene *C*H), 128.2 (**Major** Ar *C*H), 128.0 (**Minor** Ar *C*H), 127.4 (**Minor** Thiophene *C*H), 126.9 (**Major** Ar *C*H), 124.8 (**Major** Ar *C*H), 123.0 (**Minor** CH_2), 66.9 (**Minor** CH_2), 66.7 (**Major** CH_2), 66.5 (**Major** CH_2), 66.4 (**Minor** CH_2), 49.3 (**Minor** Ar-CH_2-*C*H), 46.5 (**Major** CH_2), 46.1 (**Minor** CH_2), 43.6 (**Major** CH_2), 42.7 (**Major** Ar-CH_2-*C*H), 42.6 (**Minor** CH_2-CH), 30.3 (**Major** CH_3), 29.8 (**Minor** CH_3).

HRMS: calcd. for $C_{19}H_{22}NO_3S$ $[M+H]^+$: 344.1315; found (ESI^+): 344.1319.

ν_{max} (neat/cm^{-1}): 633, 1028, 1225, 1439, 1477, 1636, 1676, 1721, 2854, 2925.

Methyl 3-(1-morpholino-1-oxo-3-phenylpropan-2-yl)benzo[b]thiophene-2-carboxylate (5o)

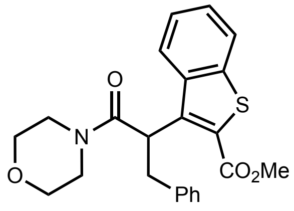

$C_{23}H_{23}NO_4S$
MW: 409.50

Synthesised according to **GP-7** from **2u** (43 mg, 0.1 mmol), PTH (1.4 mg, 0.005 mmol), and methyl benzo[b]thiophene-2-carboxylate (384 mg, 2.0 mmol) in 1,2-DCE (0.2 mL). Purification by column chromatography (silica gel; 0-50% EtOAc in hexane) afforded the product **5o** as an off yellow oil (27.4 mg, 0.067 mmol, 67%).

^{1}H NMR (400 MHz, $CDCl_3$): δ 8.41 (dt, *J* = 8.3, 1.0 Hz, 1H, Ar *H*), 7.85 (dt, *J* = 8.2, 1.0 Hz, 1H, Ar *H*), 7.52 (ddd, *J* = 8.2, 7.1, 1.2 Hz, 1H, Ar *H*), 7.46 (ddd, *J* = 8.2, 7.0, 1.2 Hz, 1H, Ar *H*), 7.14 – 7.06 (m, 3H, Ar *H*), 6.81 – 6.72 (m, 2H, Ar *H*), 5.65 (dd, *J* = 9.9, 5.6 Hz, 1H, C*H*), 3.68 – 3.50 (m, 5H, 2 × CH_2, PhCH_{2A}), 3.43 – 3.30 (m, 3H, CH_2, CH_{2A}), 3.11 (dd, *J* = 13.5, 9.9 Hz, 1H, PhCH_{2B}), 2.92 – 2.84 (m, 1H, CH_{2B}) 2.17 (s, 3H, CO_2CH_3).

^{13}C{^{1}H} NMR (126 MHz, $CDCl_3$): δ 194.4 (*C*=O), 170.6 (*C*=O), 139.6 (Ar *C*), 139.1 (Ar *C*), 138.2 (Ar *C*), 138.2 (Ar *C*), 134.9 (Ar *C*), 129.4 (Ar *C*H), 127.9 (Ar *C*H), 127.6 (Ar *C*H), 126.1 (Ar *C*H), 125.7 (Ar *C*H), 125.5 (Ar *C*H), 122.9 (Ar *C*H), 66.9 (CH_2), 66.5 (CH_2), 45.7 (CH_2), 44.0 (*C*H), 43.0 (CH_2), 38.6 (PhCH_2), 30.8 (CO_2CH_3).

HRMS: calcd. for $C_{23}H_{24}NO_4S$ $[M+H]^+$: 410.1421; found (ESI$^+$): 410.1429.

v_{max} (neat/cm^{-1}): 649, 1025, 1265, 1431, 1495, 1643, 1669, 2853, 2961.

2-(1-Morpholino-1-oxo-3-phenylpropan-2-yl)benzo[b]thiophene-3-carbonitrile (5p)

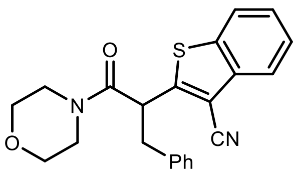

$C_{22}H_{20}N_2O_2S$
MW: 376.47

Synthesised according to **GP-7** from **2u** (42.9 mg, 0.1 mmol), PTH (1.4 mg, 0.005 mmol), and benzo[b]thiophene-3-carbonitrile (318 mg, 2.0 mmol) in 1,2-DCE (0.2 mL). Purification by column chromatography (silica gel; 0-50% EtOAc in Hexane) afforded the product **5p** as an off yellow oil (21.0 mg, 0.056 mmol, 56%).

^{1}H NMR (500 MHz, $CDCl_3$): δ 7.89 – 7.83 (m, 2H, Ar *H*), 7.52 – 7.40 (m, 2H, Ar *H*), 7.35 – 7.25 (m, 5H, Ar *H*), 4.92 (dd, *J* = 10.0, 5.2 Hz, 1H, CH_2CH), 3.74 – 3.65 (m, 1H, CH_{2A}), 3.62 – 3.54 (m, 1H, CH_{2B}), 3.51 – 3.36 (m, 5H, CH_2, CH_{2A} + $PhCH_2$), 3.30 – 3.15 (m, 2H, CH_{2A}), 2.94 – 2.85 (m, 1H, CH_{2B}).

$^{13}C\{^1H\}$ NMR (126 MHz, $CDCl_3$): δ 169.0 (*C*N), 156.6 (*C*=O), 138.7 (Ar *C*), 137.4 (Ar *C*), 136.7 (Ar *C*), 129.3 (Ar *C*H), 129.0 (Ar *C*H), 127.5 (Ar *C*H), 126.1 (Ar *C*H), 126.0 (Ar *C*H), 122.8 (Ar *C*H), 122.2 (Ar *C*H), 114.4 (Ar *C*), 104.7 (Ar *C*), 66.6 (CH_2), 66.3 (CH_2), 46.7 (CH_2), 45.5 (*C*H), 43.0 (CH_2-CH), 42.9 (CH_2).

HRMS: calcd. for $C_{22}H_{21}N_2O_2S$ $[M+H]^+$: 377.1318; found (ESI$^+$): 377.1310.

v_{max} (neat/cm^{-1}): 648, 1023, 1262, 1435, 1494, 1640, 1787, 2855, 2962.

2-(1-Methyl-1H-pyrrolo[2,3-b]pyridin-2-yl)-1-morpholino-3-phenylpropan-1-one (5q)

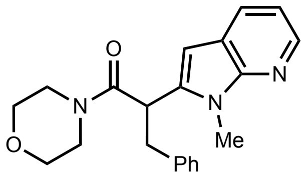

$C_{21}H_{23}N_3O_2$
MW: 349.43

Synthesised according to **GP-7** from **2u** (42.9 mg, 0.1 mmol), PTH (1.4 mg, 0.005 mmol), and 1-methyl-7-aza-1*H*-indole (264 mg, 2.0 mmol) in 1,2-DCE (0.2 mL). Purification by column chromatography (silica gel; 0-45% EtOAc in hexane) afforded the product **5q** as an off yellow oil (19.2 mg, 0.055 mmol, 55%, 3.0:1 r.r.).

^{1}H NMR (500 MHz, $CDCl_3$): δ 9.51 – 9.46 (m, 1H, **Minor** Ar *H*), 8.49 – 8.44 (m, 1H, **Major** Ar *H* + **Minor** Ar *H*), 8.31 – 8.23 (m, 1H, **Major** Ar *H* + **Minor** Ar *H*), 7.82 – 7.81 (m, 1H, **Major** Ar *H* + **Minor** Ar *H*), 7.31 – 7.19 (m, 5H, **Major** Ar *H* + **Minor** Ar *H*), 7.15 – 7.01 (m, 5H, **Major** Ar *H* + **Minor** Ar *H*), 6.45 (s, 1H, **Major** Ar *H*), 4.21 (dd, *J* = 8.2, 6.3 Hz, 1H, **Major** C*H*), 3.72 – 3.51 (m, 8H, **Major** CH_2+ **Major** CH_2+ **Major** CH_2 + **Minor** CH_2), 3.40 (s, 3H, **Minor** CH_3), 3.35 (s, 3H, **Major** CH_3), 3.23 – 3.13 (m, 3H, **Major** CH_2+ **Major** CH_2 + **Major** CH_2 + **Minor** CH_2).

$^{13}C\{^1H\}$ NMR (126 MHz, $CDCl_3$): δ 173.7 (**Major** *C*=O), 156.0 (**Major** Ar *C*), 142.6 (**Major** Ar *C*H), 139.0 (**Major** Ar *C*), 132.9 (**Major** Ar *C*H), 129.3 (**Major** Ar *C*H), 128.8 (**Major** Ar *C*H), 128.3 (**Major** Ar *C*), 127.0 (**Major** Ar *C*), 119.7 (**Major** Ar *C*H), 116.2 (**Major** Ar *C*H), 98.8 (**Major** Ar *C*H), 66.8 (**Major** CH_2), 66.2 (**Major** CH_2), 43.2 (**Major** *C*H), 42.8 (**Major** CH_2CH), 39.7 (**Major** CH_2), 28.1 (**Major** CH_2), 25.3 (**Major** CH_3).

HRMS: calcd. for $C_{21}H_{24}N_3O_2$ $[M+H]^+$: 350.1863; found (ESI$^+$): 350.1869.

ν_{max} (neat/cm^{-1}): 629, 1027, 1228, 1462, 1510, 1644, 1735, 2844, 2929.

6-Chloro-2-(1-methyl-1*H*-pyrrol-2-yl)-1-morpholinohexan-1-one (5r)

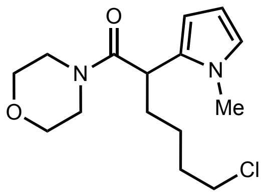

$C_{15}H_{23}ClN_2O_2$
MW: 298.81

Synthesised according to **GP-7** from **2y** (42.9 mg, 0.1 mmol), PTH (1.4 mg, 0.005 mmol), and N-methylpyrrole (0.18 mL, 2.0 mmol) in 1,2-DCE (0.2 mL). Purification by column chromatography (silica gel; 0-50% EtOAc in hexane) afforded the product **5r** as a colourless oil (13.0 mg, 0.044 mmol, 44%, >20:1 r.r.).

^{1}H NMR (400 MHz, $CDCl_3$): δ 6.53 (dd, *J* = 2.8, 1.8 Hz, 1H, Ar *H*), 6.03 (dd, *J* = 3.6, 2.8 Hz, 1H, Ar *H*), 5.91 (dd, *J* = 3.6, 1.8 Hz, 1H, Ar *H*), 3.81 – 3.18 (m, 8H, 4 × CH_2), 3.72 (dd, *J* = 8.2, 6.1 Hz, 1H, C*H*), 3.58 (s, 3H, CH_3), 3.53 (t, *J* = 6.7 Hz, 2H, CH_2Cl), 2.15 – 2.03 (m, 1H, CH_{2A}), 1.87 – 1.69 (m, 3H, CH_2, CH_{2B}), 1.56 – 1.35 (m, 2H, CH_2).

^{13}C NMR (101 MHz, $CDCl_3$): δ 170.6 (*C*=O), 130.2 (Ar *C*), 122.7 (Ar *C*H), 107.9 (Ar *C*H), 107.3 (Ar *C*H), 67.0 (*C*H$_2$), 66.5 (*C*H$_2$), 46.2 (*C*H$_2$), 45.0 (*C*H$_2$), 42.8 (*C*H$_2$), 41.9 (*C*H), 34.3 (*C*H$_3$), 32.6 (*C*H$_2$), 32.0 (*C*H$_2$), 25.4 (*C*H$_2$).

HRMS: calcd. for $C_{15}H_{23}N_2O_2ClNa$ $[M+Na]^+$: 321.1340; found (ESI$^+$): 321.1332.

Sulfonium Salt Formation

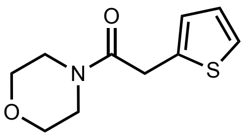

<5%
decomposition

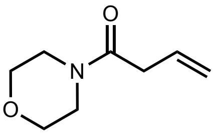

<5%
decomposition

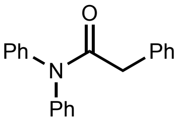

<5%

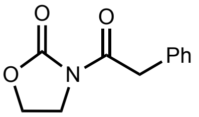

<5%

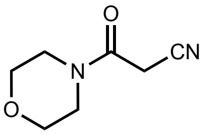

<5%

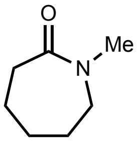

<5%

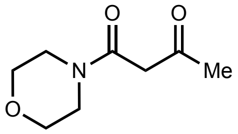

<5%
decomposition

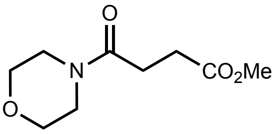

<5%
decomposition

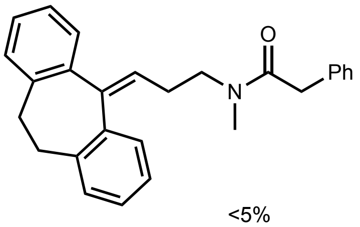

<5%
decomposition

Photochemical Alkenylation

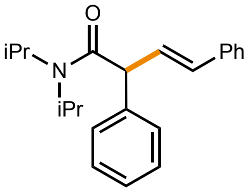

<5%

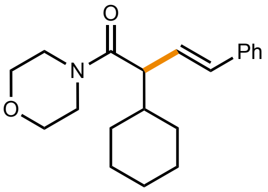

<5%

Photochemical Alkylation

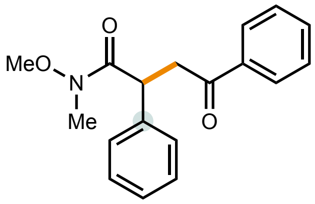

<5%

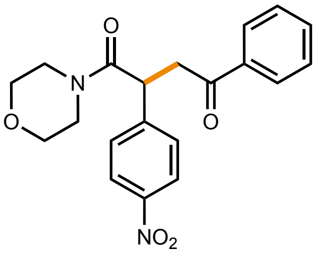

18%

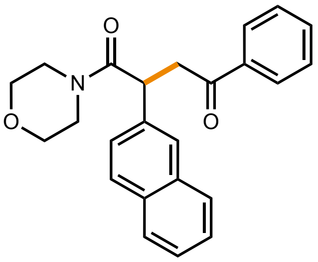

<5%

Photochemical Arylation

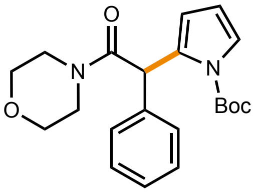

<5%

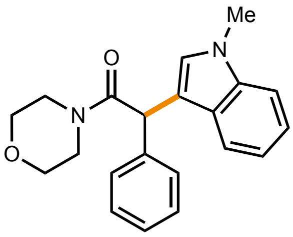

<5%

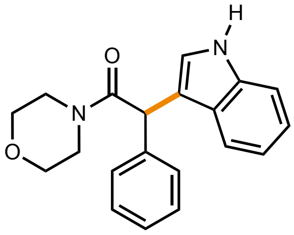

<5%

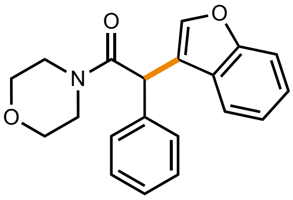

<5%

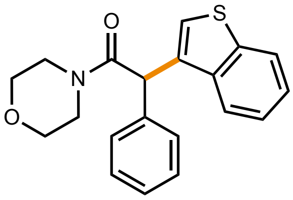

<5%

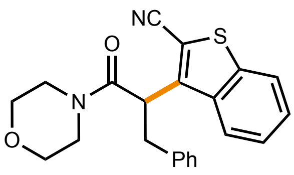

<5%

7 Mechanistic Investigations

7.1 Cyclic Voltammetry

General Experimental Details

CVs were recorded on an EmStat (PalmSens) potentiostat using a 3-electrode cell configuration. A glassy carbon working electrode was employed alongside a platinum wire counter electrode and an Ag/AgCl reference electrode. Prior to measurements, all solutions were degassed by sparging with dinitrogen. Tetrabutylammonium hexafluorophosphate solutions (0.1 M) in MeCN were used as supporting electrolyte, with 0.005 M – 0.1 M solutions of each compound prepared in the electrolyte solutions.

Measurements were conducted from 0 V to -2.0 – -3.0 V and employed a scan-rate of 0.05 V s^{-1} and step of 0.01 V. Corrections were performed to give the final potential values against saturated calomel electrodes (SCE), in order to better compare these values with reported data. The CV traces obtained are irreversible (Supplementary Figures S1-S4), hence reduction potentials are reported as the half peak potential ($E_{1/2}^{red}$).

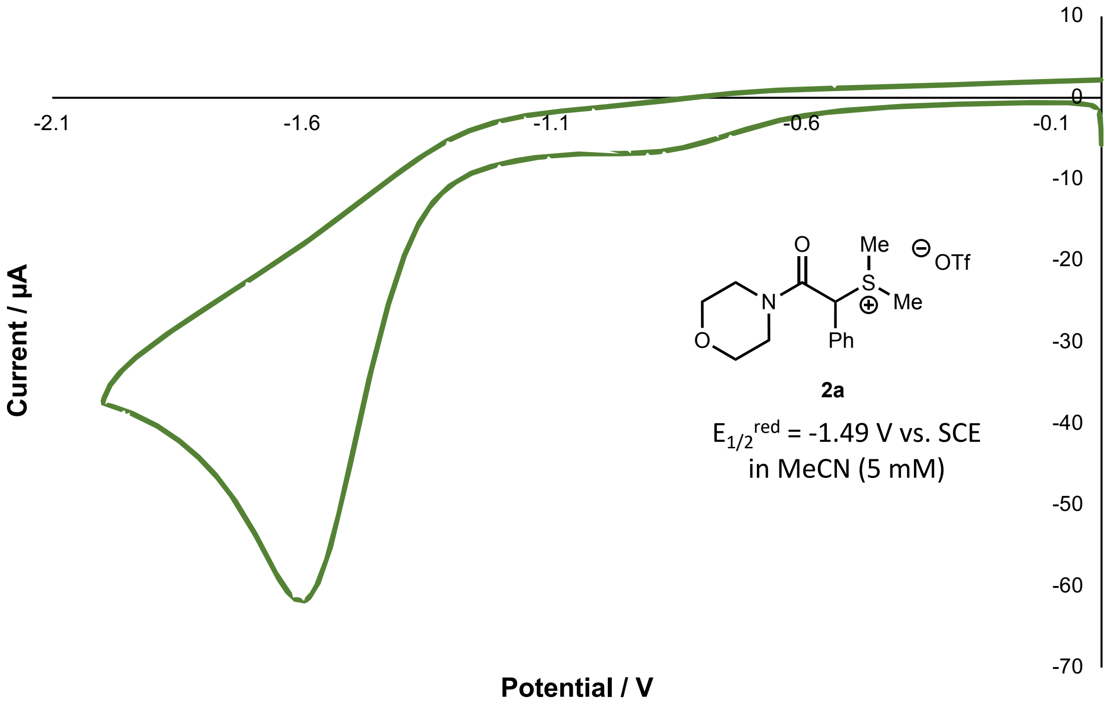

Supplementary Figure S1: *Cyclic voltammogram of α-amido sulfonium salt **2a***

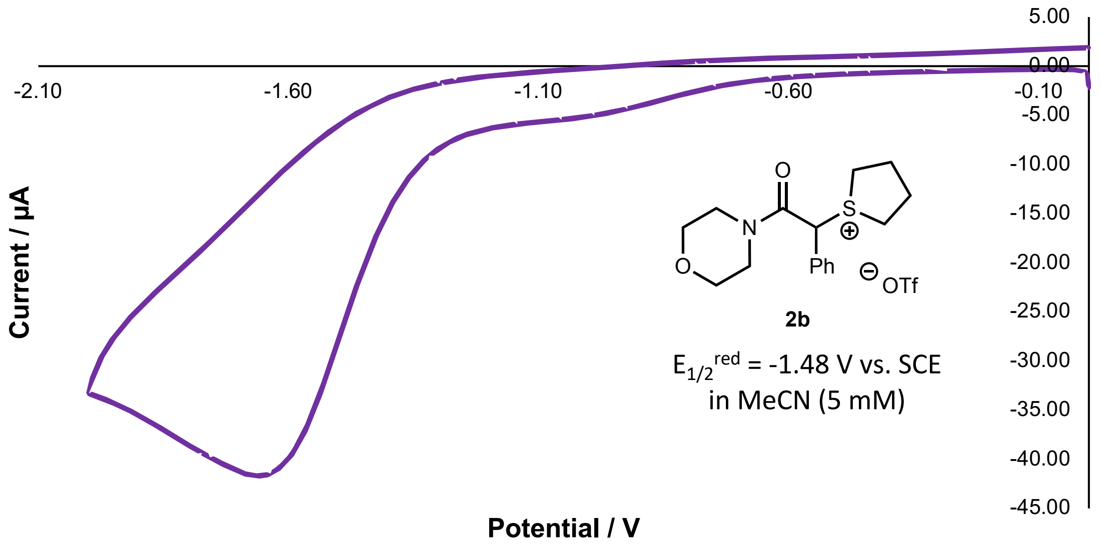

Supplementary Figure S2: *Cyclic voltammogram of α-amido sulfonium salt **2b***

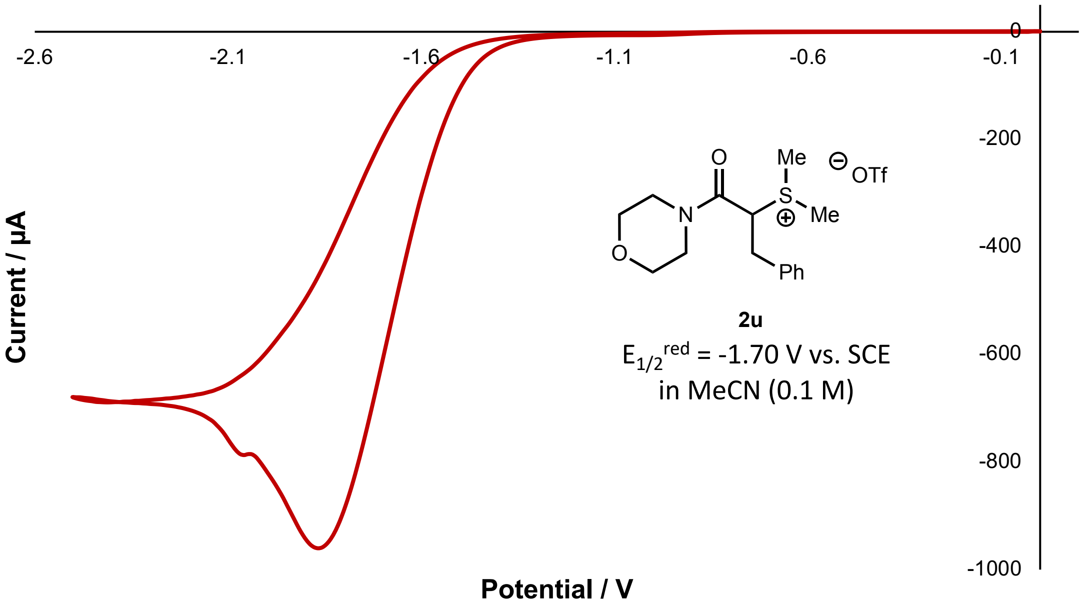

Supplementary Figure S3: *Cyclic voltammogram of α-amido sulfonium salt* ***2s***

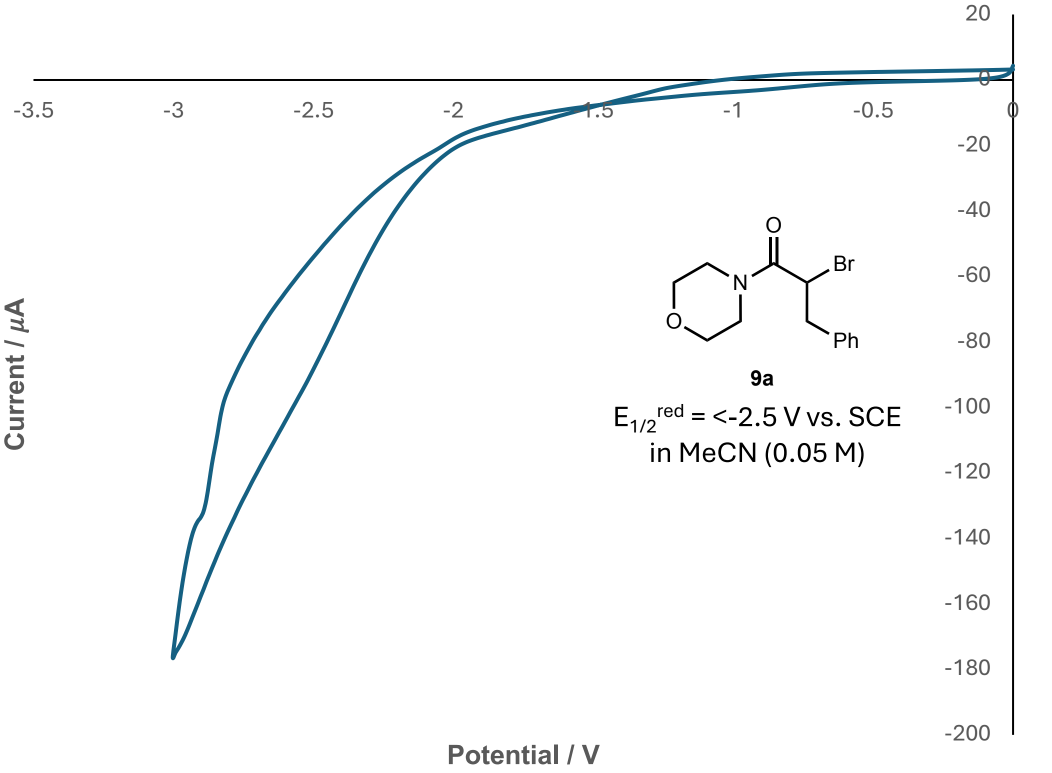

Supplementary Figure S4: *Cyclic voltammogram of α-bromo amide* ***9a***

2-Bromo-1-morpholino-3-phenylpropan-1-one (9a)

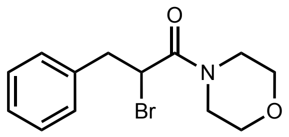

$C_{13}H_{16}BrNO_2$

MW: 298.18

According to the literature procedure,[20] a flame-dried flask was charged with 1-morpholino-3-phenylpropan-1-one (219 mg, 1.0 mmol), 2-iodopyridine (0.23 mL, 2.2 mmol) and anhydrous CH_2Cl_2 (10 mL, 0.1 M) and cooled to 0 °C (ice/water bath). Tf_2O (0.18 mL, 1.1 mmol) was added drop-wise and the reaction stirred for 15 min, after which 2,6-lutidine *N*-oxide (0.11 mL, 1.0 mmol) was added drop-wise. After stirring for an additional 5 min, tetra(n-butyl)ammonium bromide (TBAB, 967 mg, 3.0 mmol) was added in one portion and the reaction mixture warmed to rt and stirred for 3 h. The reaction was quenched with saturated aqueous NH_4Cl (10 mL), the organic layer separated, and the aqueous layer extracted with CH_2Cl_2 (3 × 10 mL). The combined organics were dried over $MgSO_4$, filtered, and concentrated *in vacuo.* Purification by column chromatography (silica gel; 20-30% EtOAc in hexane) afforded the product **9a** as a yellow oil (254 mg, 0.852 mmol, 85%).

A 1.5:1 mixture of rotamers was observed at 298 K in $CDCl_3$.

^{1}H NMR (400 MHz, $CDCl_3$): δ 7.35 – 7.21 (m, 5H), 4.60 – 4.54 (m, 1H), 3.79 – 3.14 (m, 10H).

^{13}C NMR (101 MHz, $CDCl_3$): δ 167.1 (*C*=O), 166.9 (*C*=O), 137.6 (Ar *C*), 136.9 (Ar *C*H), 129.7 (Ar *C*H), 129.6 (Ar *C*H), 127.33 (Ar *C*H), 127.31 (Ar *C*H), 66.7 (CH_2), 66.5 (CH_2), 66.3 (CH_2), 53.9 (*C*H), 46.7 (CH_2), 46.5 (CH_2), 43.1 (CH_2), 42.9 (CH_2), 41.2 (CH_2), 40.9 (CH_2).

HRMS: calcd. for $C_{13}H_{16}BrNO_2Na$ $[M+Na]^+$: 320.0257; found (ESI^+): 320.0249.

7.2 Quantum Yield Measurements

Quantum Yield measurements were carried out as previously reported.[21,22]

Preparation of Solutions

Ferrioxalate solution (A)

In a dark room, potassium ferrioxalate trihydrate (1.84 g, 3.75 mmol) was added to a 25 mL volumetric flask. H_2SO_4 (0.05 M in H_2O) was then added until the graduation mark was reached, and the solution allowed to equilibrate for 30 min. The solution was wrapped in aluminium foil and stored in the dark.

Phenanthroline solution (B)

Phenanthroline (50 mg, 0.28 mmol) was added to a 25 mL volumetric flask and H_2O was added until the solution reached the graduation mark. The solution allowed to equilibrate for 30 min.

Buffer Solution (C)

NaOAc (1.24 g, 12.5 mmol) was added to a 25 mL volumetric flask. Water (HPLC grade, 20 mL) was added followed by H_2SO_4 (95% w/w, 250 μL), water was then added until the graduation mark was reached, and the solution allowed to equilibrate for 30 min.

Measurements

Photon Flux Measurement (390 nm)

In a dark room, a microwave vial was charged with **solution A** (1.0 mL) and irradiated for 5 s. After irradiation, 0.1 mL of the solution was immediately transferred to a 5 mL volumetric flask containing **solution B** (0.5 mL), **solution C** (2 mL) and water (HPLC grade, 1.5 mL). This was repeated 2 more times, irradiating for 10 s and 15 s, respectively. A control sample was also made, where **solution A** (0.1 mL) was added directly to a 5 mL volumetric flask containing **solution B** (0.5 mL) and **solution C** (2 mL) and water (HPLC grade, 1.5 mL). The UV/Vis spectra of the samples were then taken (blank sample = solution C (2 mL) in H_2O (2 mL)) and the absorption measured at 510 nm.

The conversion was calculated using equation 1:

$$mol\ Fe^{2+} = \frac{V_1\, V_3 \Delta A}{V_2\ l\ \varepsilon} \qquad (1)$$

V_1 = irradiated volume (0.001 L).

V_2 = aliquot of irradiated solution added to quencher (0.0001 L).

V_3 = volume after complexation (0.0041 L).

ΔA = difference in absorbance between the irradiated and non-irradiated solutions.

l = path length (1 cm).

ε = molar absorptivity at 510 nm (11100 L mol^{-1} cm^{-1}).

Photon Flux was calculated using equation 2:

$$photon\ flux = \frac{mol\ Fe^{2+}}{\Phi\ t\ f} \qquad (2)$$

Φ = quantum yield for the ferrioxalate actinometer (1.14 at 436 nm).[21]

t = time.

f = fraction of light absorbed by ferrioxalate at 390 nm (>0.999, calculation shown below).

The moles of Fe^{2+} were plotted as a function of time (fig. x), allowing the slope of the graph to be used to represent $\frac{mol\ Fe^{2+}}{t}$ which was determined to be 2.085x10^{-7} mol s^{-1}. The photon flux was then calculated to be 1.83x10^{-7} einstein s^{-1}.

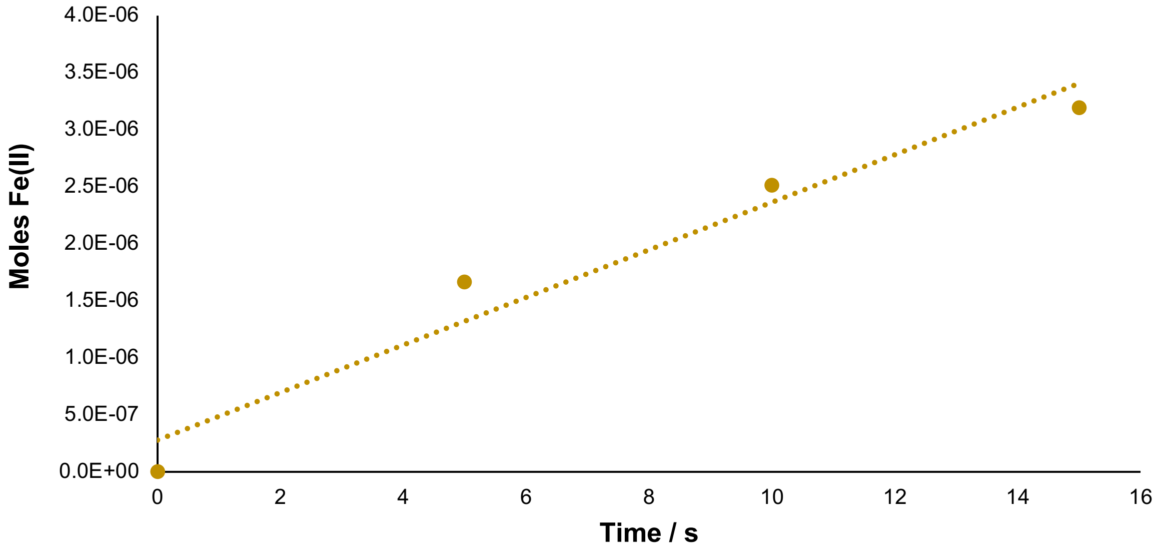

Supplementary Figure S5: *Moles of actinometer converted to Fe(II) as a function of time. Used to calculate photon flux of 390 nm light source*

Fraction of Light Absorbed (f) by Ferrioxalate Measurement

In a dark room, a quartz cuvette was charged with solution A directly. The UV/Vis spectrum of the sample was taken and the absorbance at 390 nm measured. The fraction of light absorbed was calculated using eq. 3:

$$fraction\ of\ light\ absorbed\ (f) = 1 - 10^{-A} \quad (3)$$

A = absorbance of the actinometer at 390 nm (>4).

Quantum Yield Measurement – Photochemical Alkylation

In a darkened room, the photochemical alkylation was performed; an oven dried microwave vial containing a stirring bar was charged with α-amido sulfonium salt **2a** (41.5 mg, 0.10 mmol) and PTH (1.4 mg, 0.01 mmol), then sealed and purged with N_2. ((1-(4-fluorophenyl)vinyl)oxy)trimethylsilane (105.2 mg, 0.5 mmol) was added via syringe, followed by 1,2-DCE (0.25 mL). The sample was stirred for set times under irradiation with a 34 W Kessil PR160L purple LED (λ_{max} = 390 nm) set to 25% intensity. After irradiation the reaction was diluted in CH_2Cl_2 and concentrated *in vacuo*. The moles of product **3d** formed was quantified by ^1H NMR spectroscopy, using $MeNO_2$ as an internal standard; this was used to calculate the quantum yield using a modified eq 2.

$$\Phi = \frac{mol\ product}{flux\ t\ f} \quad (2)$$

t = reaction time.

f = fraction of light absorbed by reaction mixture at 390 nm (>0.999 based on an absorbance of >4).

Reactions performed for two different time periods (4 h and 8 h) were carried out, each averaged over two runs. The moles of product formed were plotted as a function of incident photons, allowing the slope of the graph to be used to represent $\frac{mol\ product}{flux\ t}$.

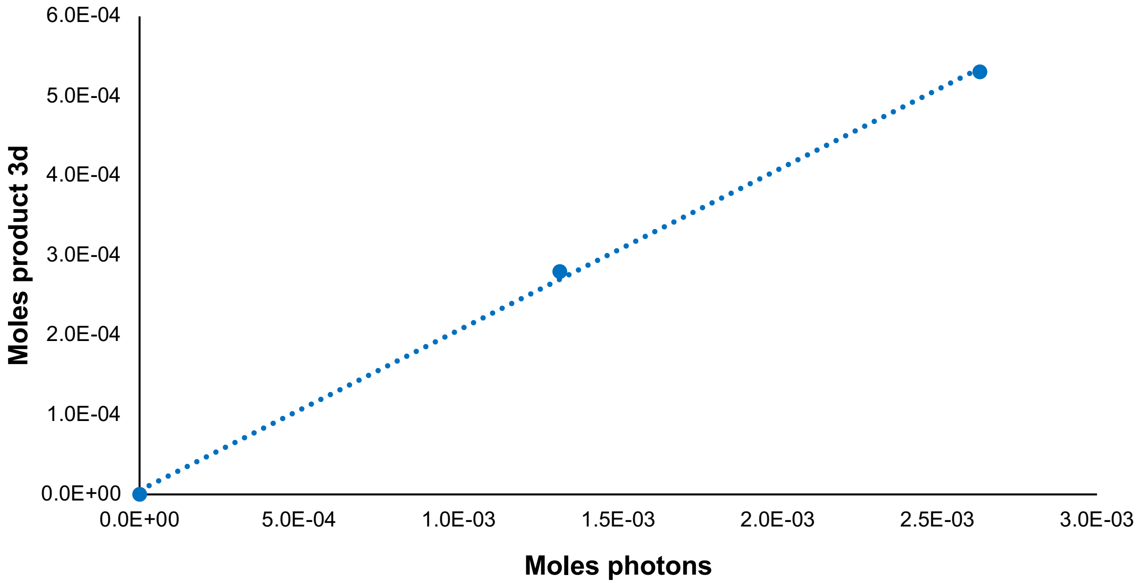

Supplementary Figure S6: *Moles of product* ***3d*** *formed in the photochemical alkylation reaction as a function of the moles of incident photons. Used to calculate the quantum yield of the reaction.*

The slope was calculated to be 0.2012 and hence Φ was calculated to be 0.20.

Quantum Yield Measurement – Photochemical Arylation

In a dark room, the photochemical arylation reaction was performed; an oven dried microwave vial containing a stirring bar was charged with α-amido sulfonium salt **2a** (41.5 mg, 0.10 mmol), PTH (1.4 mg, 0.01 mmol) and $NaHCO_3$ (25.2 mg, 0.30 mmol), then sealed and purged with dinitrogen. *N*-methyl pyrrole **8a** (0.18 mL, 2.00 mmol) was added via syringe, followed by 1,2-DCE (0.25 mL). The sample was stirred under irradiation with a 34 W Kessil PR160L purple LED (λ_{max} = 390 nm) set to 25% intensity for set time intervals. After irradiation the reaction was diluted in CH_2Cl_2 and concentrated *in vacuo*. The moles of product **5a** formed was quantified by ^1H NMR spectroscopy, using $MeNO_2$ as an internal standard. This value was used to calculate the quantum yield using a modified equation 2.

$$\Phi = \frac{mol\ product}{flux\ t\ f} \quad (2)$$

t = reaction time.

f = fraction of light absorbed by reaction mixture at 390 nm (>0.999 based on an absorbance of >4).

Reactions performed for two different time periods (4 h and 8 h) were carried out, each averaged over two runs. The moles of product formed were plotted as a function of incident photons, allowing the slope of the graph to be used to represent $\frac{mol\ product}{flux\ t}$.

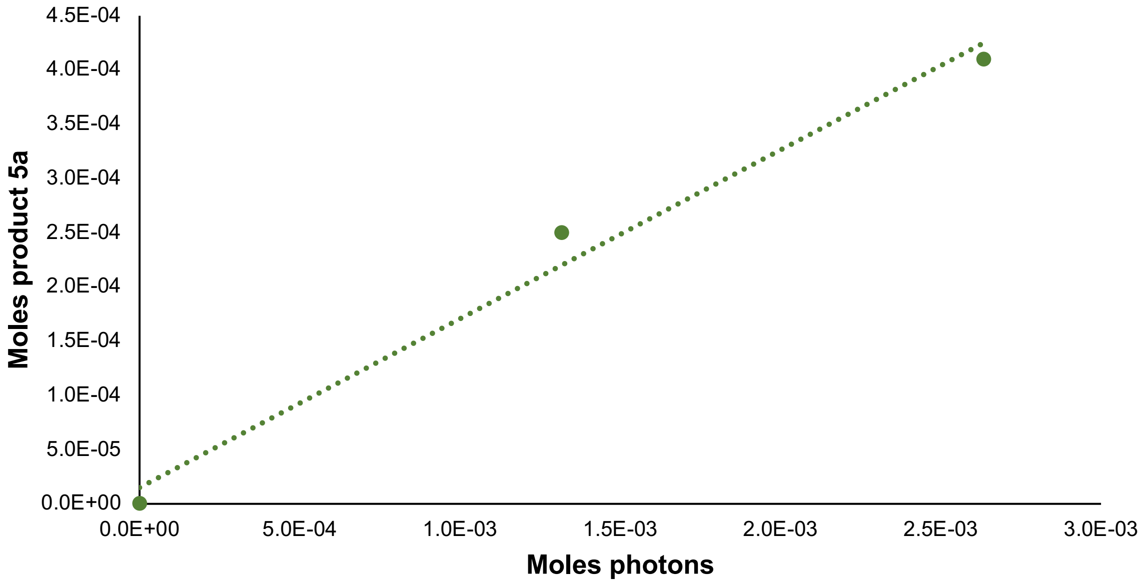

Supplementary Figure S7: *Moles of product* ***5a*** *formed in the photochemical heteroarylation reaction as a function of the moles of incident photons. Used to calculate the quantum yield of the reaction.*

Supplementary Figure S8:

The slope was calculated to be 0.1557 and hence Φ was calculated to be 0.156.

Photon Flux measurement (456 nm)

In a darkened room, a microwave vial was charged with 1.0 mL of **solution A** and irradiated for 5 s. After irradiation, 0.1 mL of the solution was immediately transferred to a 5 mL volumetric flask containing 0.5 mL **solution B** and 2 mL **solution C** and 1.5 mL H_2O (HPLC grade). This was repeated 2 more times, irradiating for 10 s and 15 s. A control sample was also made, where 0.1 mL **solution A** was added directly to a 5 mL volumetric flask containing 0.5 mL **solution B** and 2 mL **solution C** and 1.5 mL H_2O (HPLC grade). The UV/Via spectra of the samples were then taken (blank sample = 2 mL **solution C** in 2 mL H_2O) and the absorbance measured at 510 nm.

Conversion was calculated using eq. 1:

$$mol\,Fe^{2+} = \frac{V_1\,V_3\Delta A}{V_2\;l\;\varepsilon} \quad (1)$$

V_1 = irradiated volume (0.001 L).

V_2 = aliquot of irradiated solution added to quencher (0.0001 L).

V_3 = volume after complexation (0.0041 L).

ΔA = difference in absorbance between the irradiated and non-irradiated solutions.

l = path length (1 cm).

ε = molar absorptivity at 510 nm (11100 L mol^{-1} cm^{-1}).

Photon Flux was calculated using eq. 2:

$$photon\,flux = \frac{mol\,Fe^{2+}}{\Phi\;t\;f} \quad (2)$$

Φ = quantum yield for the ferrioxalate actinometer (1.14 at 436 nm – Hatchard Parker).[56]

t = time.

f = fraction of light absorbed by ferrioxalate at 456 nm (0.9925, calculation shown below).

The moles of Fe^{2+} were plotted as a function of time allowing the slope of the graph to be used to represent $\frac{mol\ Fe^{2+}}{t}$.

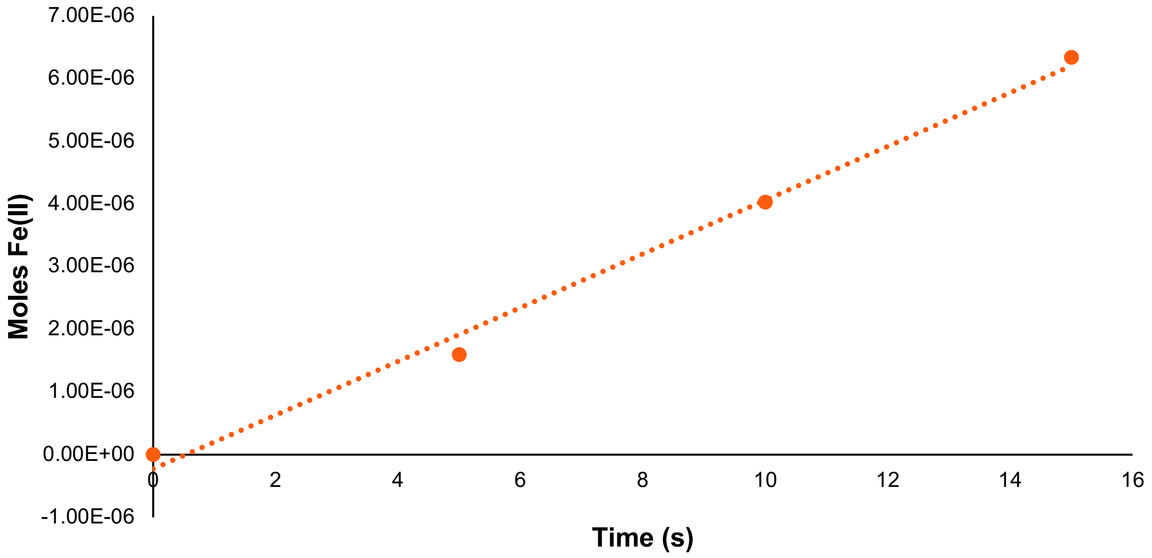

Supplementary Figure S9: *Moles of actinometer converted to Fe(II) as a function of time. Used to calculate photon flux of 456 nm light source.*

Supplementary Figure S10:

The slope was determined to be 4.287×10^{-7} mol s^{-1}. The photon flux was then calculated to be 3.89×10^{-7} einstein s^{-1}.

Fraction of Light Absorbed (f) by Ferrioxalate Measurement

In a dark room, a quartz cuvette was charged with **solution A** directly. The UV/Vis spectrum of the sample was taken and the absorbance at 456 nm was measured. The fraction of light absorbed was calculated using eq. 3:

$$fraction\ of\ light\ absorbed\ (f) = 1 - \ 10^{-A}\ \ (3)$$

A = absorbance of the actinometer at 456 nm (1.125).

Quantum Yield Measurement – Photochemical Alkenylation

In a dark room, the photochemical alkenylation reaction was performed; an oven dried microwave vial containing a stirring bar was charged with α-amido sulfonium salt **2a** (41.5 mg, 0.10 mmol), PTH (1.4 mg, 0.01 mmol) and potassium trifluoroborate salt **7a** (42.0 mg, 0.2 mmol), then sealed and purged with N_2. MeCN (0.5 mL) was added via syringe. The sample was stirred under irradiation with a broad wavelength Kessil A160WE Tuna blue LED (λ_{max} = 456 nm) set to 50% intensity for set time intervals. After irradiation, the reaction was diluted in MeCN and concentrated *in vacuo*. The moles of product **4a** formed was quantified by ^{1}H NMR spectroscopy using $MeNO_2$ as an internal standard. This value was used to calculate the quantum yield using equation 4.

$$\Phi = \frac{mol\,product}{flux\;t\;f} \qquad (4)$$

t = reaction time.

f = fraction of light absorbed by reaction mixture at 456 nm (0.14 based on an absorbance of 0.06 at 456 nm).

Reactions performed for two different time periods (4 h and 8 h) were carried out, each averaged over two runs. The moles of product formed were plotted as a function of incident photons, allowing the slope of the graph to be used to represent $\frac{mol\,product}{flux\;t}$.

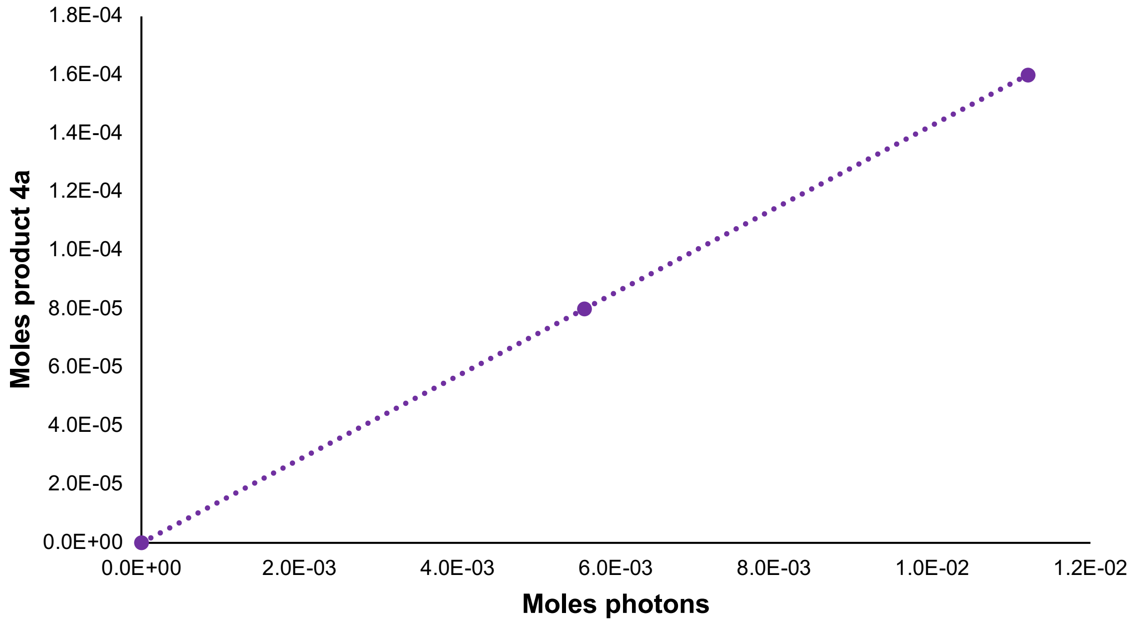

Supplementary Figure S11: *Moles of product **4a** formed in the photochemical alkenylation reaction, as a function of the moles of incident photons. Used to calculate the quantum yield of the reaction.*

The slope was calculated to be 0.0143 and hence Φ was calculated to be 0.10.

7.3 Stern-Volmer Fluorescence Quenching

General Experimental Details

Prior to measurements, the concentrations of the solutions were verified by UV-Vis electronic absorption spectroscopy, recorded on a Mettler Toledo UV5Bio spectrophotometer, and were kept below 1.5 absorption units (A.U.) to minimise inner filter effects. Emission spectra were recorded on an Edinburgh Instrument FP920 phosphorimeter equipped with a 450 W xenon lamp (with single 325 mm focal length excitation and emission monochromators in Czerny Turner configuration) and a red sensitive photomultiplier in a Peltier (air-cooled) housing, (Hamamatsu R928P). All spectra are an average of five runs and the same excitation and emission windows were used throughout. All spectra were corrected for the excitation source and the detector response, using the correction files provided by the software.

The Stern-Volmer relationship (Eq. 4) can be used to calculate the Stern-Volmer constant K_{SV} when the Stern-Volmer plot exhibits a linear relationship.

$$\frac{I_0}{I} = 1 + K_{SV}\,[Q] \qquad (5)$$

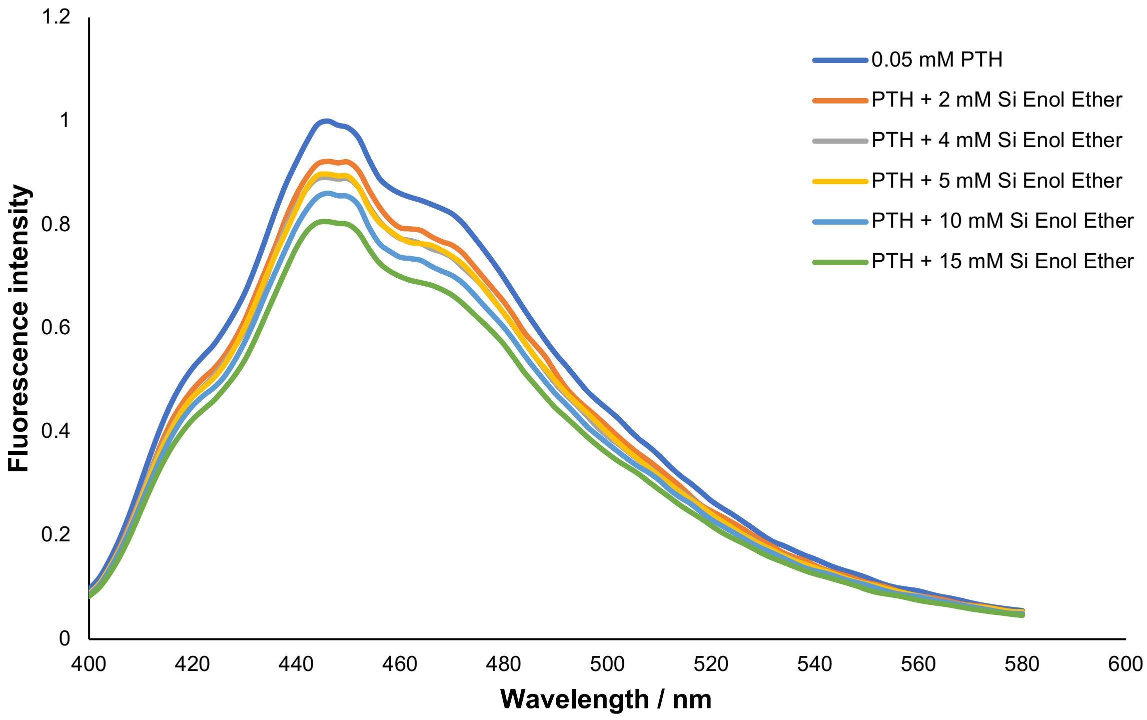

Supplementary Figure S12: *Emission spectra of PTH in MeCN (0.05 mM) with varying concentrations of silyl enol ether, normalised to the spectrum of the sole PTH .*

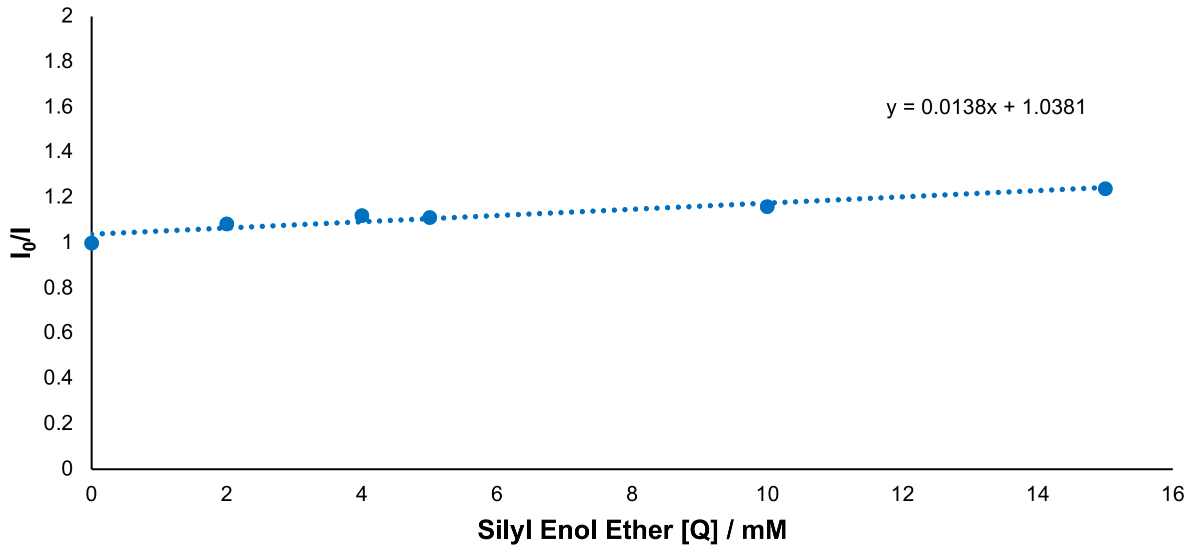

Supplementary Figure S13: *Stern-Volmer plot for PTH in MeCN (0.05 mM) with varying concentrations of silyl enol ether.*

The Stern-Volmer plot of PTH in the presence of silyl enol ether displays a linear relationship with an almost flat slope, suggesting weak quenching of PTH's excited state. The quenching constant was calculated as K_{SV} = 13.8 M^{-1}.

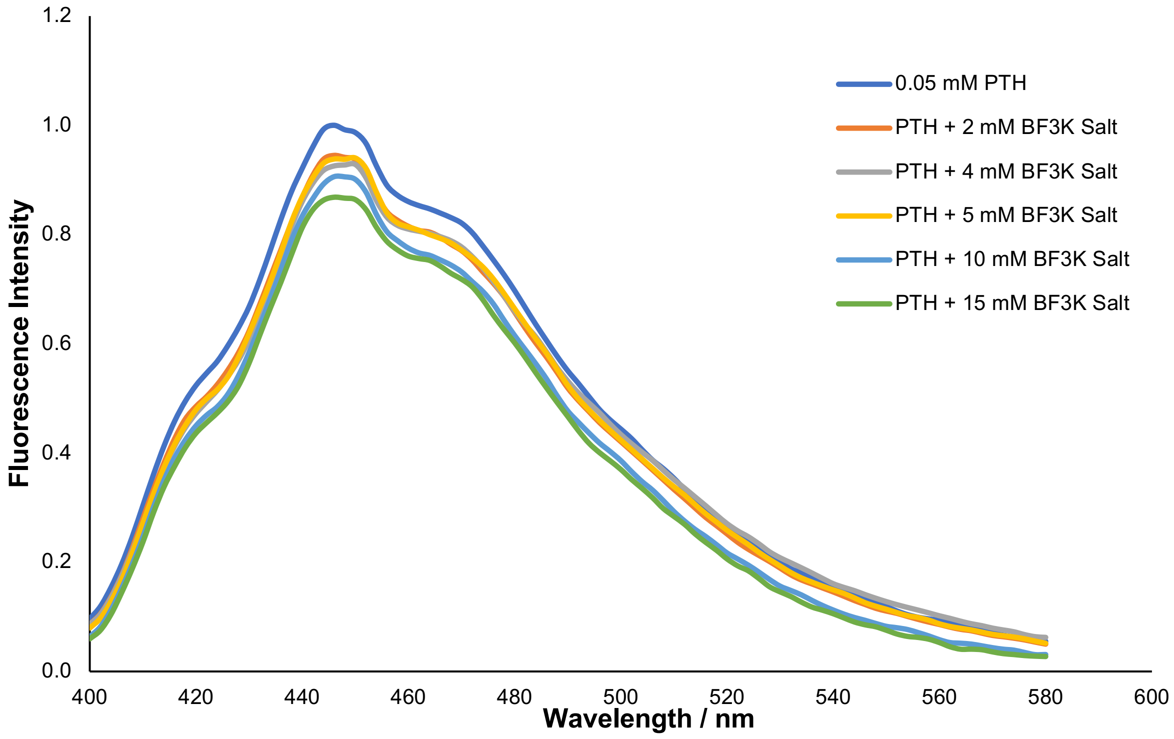

Supplementary Figure S14: *Stern-Volmer plot for PTH in MeCN (0.05 mM) with varying concentrations of BF_3K salt **7a**.*

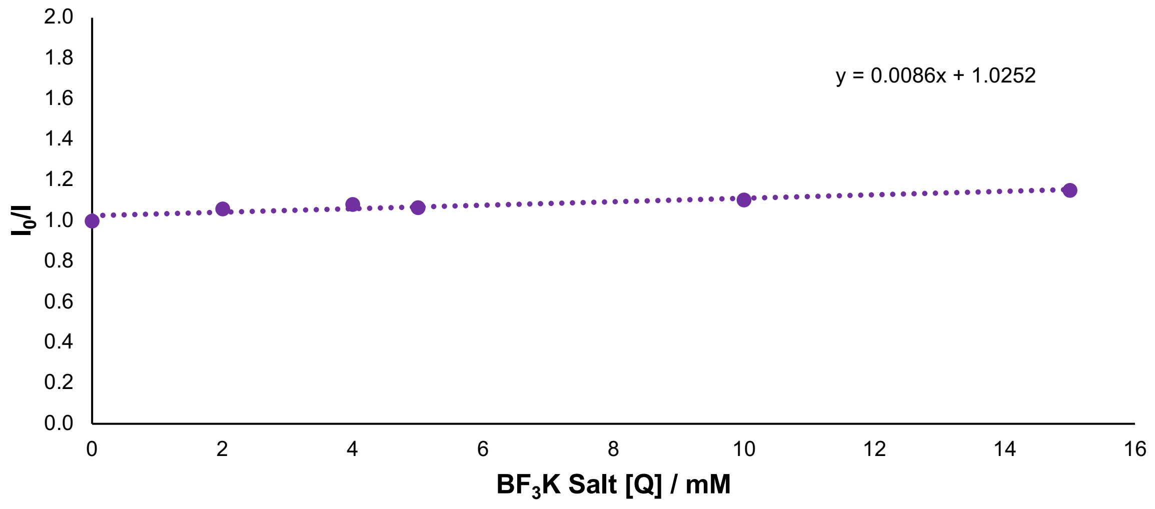

Supplementary Figure S15: *Stern-Volmer plot for PTH in MeCN (0.05 mM) with varying concentrations of BF_3K salt.*

The Stern-Volmer plot of PTH in the presence of BF_3K salt **xx** shows a linear relationship with almost flat slope, suggesting weak quenching of PTH's excited state. The quenching constant was calculated as K_{SV} = 8.6 M^{-1}.

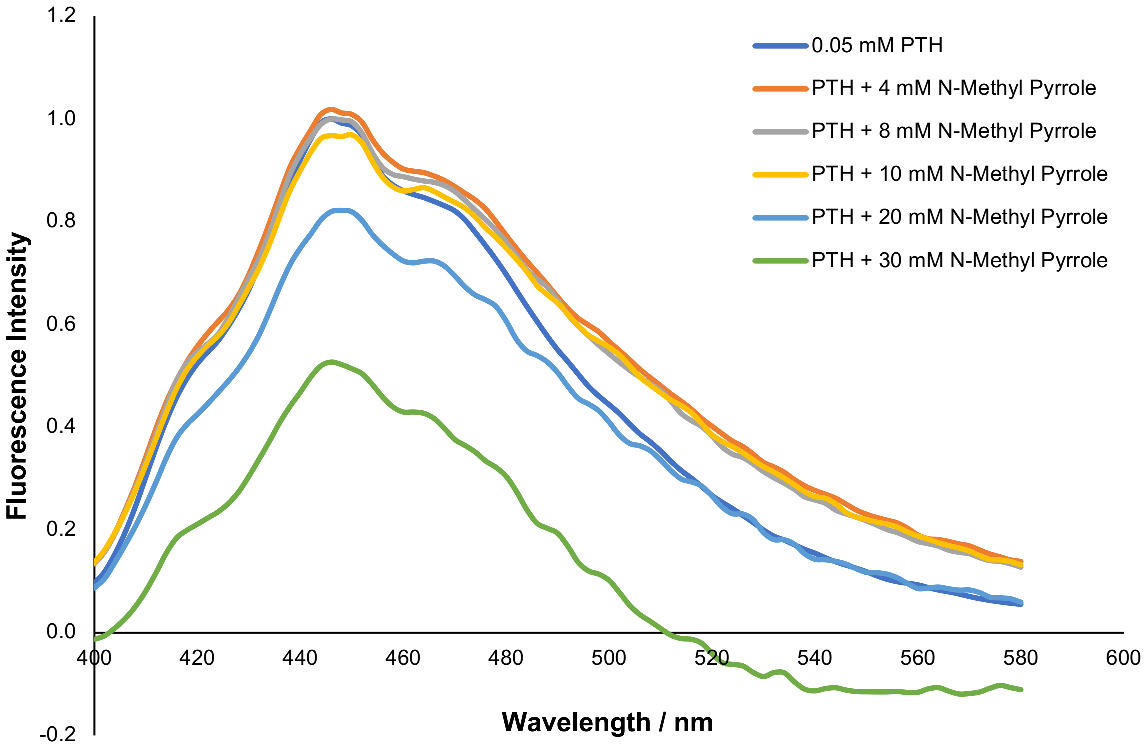

Supplementary Figure S16: *Stern-Volmer plot for PTH in MeCN (0.05 mM) with varying concentrations of N-methyl pyrrole.*

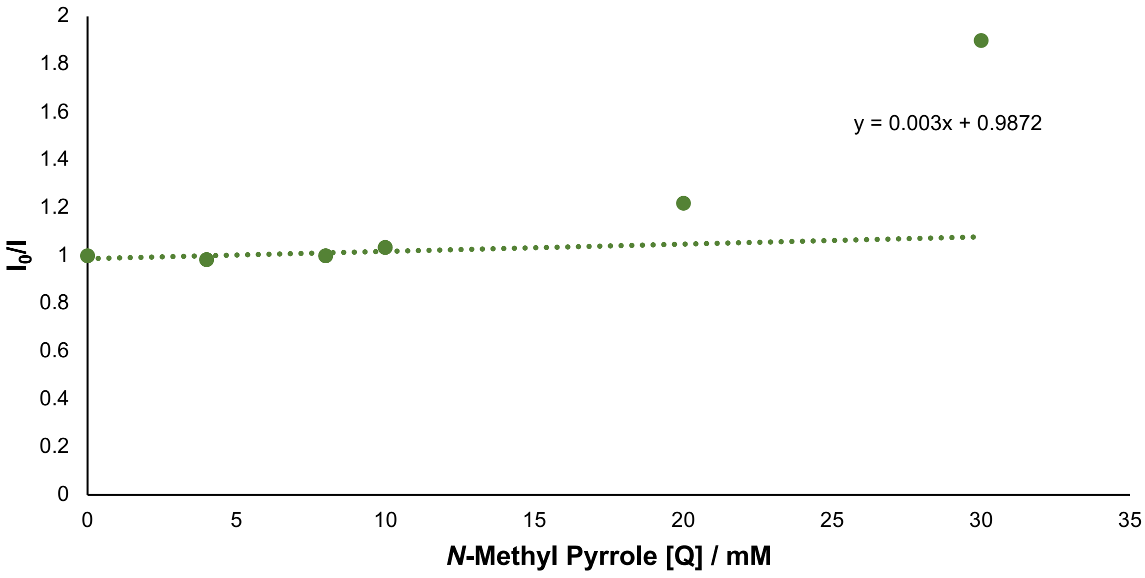

Supplementary Figure S17: *Stern-Volmer plot for PTH in MeCN (0.05 mM) with varying concentrations of N-methyl pyrrole* ***8a***.

The Stern-Volmer plot of PTH in the presence of *N*-methyl pyrrole **8a**, shows a non-linear relationship. At lower concentrations (up to 10 mM) a linear relationship can be approximated, suggesting weak quenching of PTH's excited state. The quenching constant is calculated as $K_{SV} = 3.0\ M^{-1}$.

Non-Linear effects in the Stern-Volmer plot can be caused by a number of factors, namely the existence of multiple emitting species in the solution, or the presence of multiple quenching mechanisms (for example both static and dynamic quenching).[23]

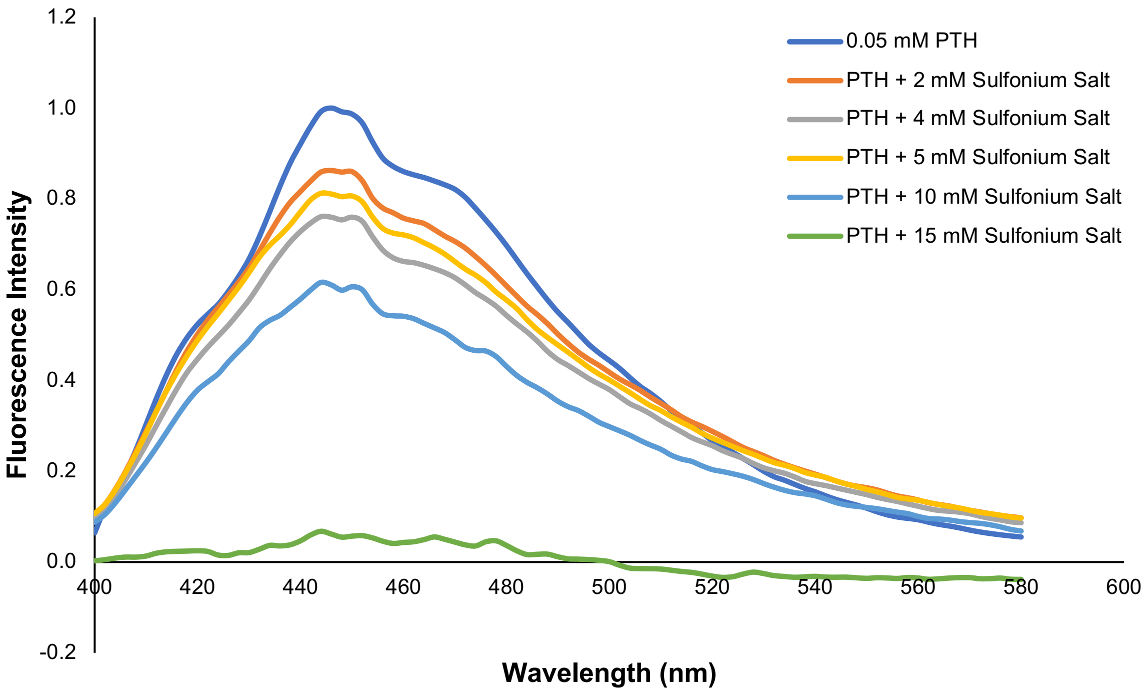

Supplementary Figure S18: *Stern-Volmer plot for PTH in MeCN (0.05 mM) with varying concentrations of α-amido sulfonium salt **2a**.*

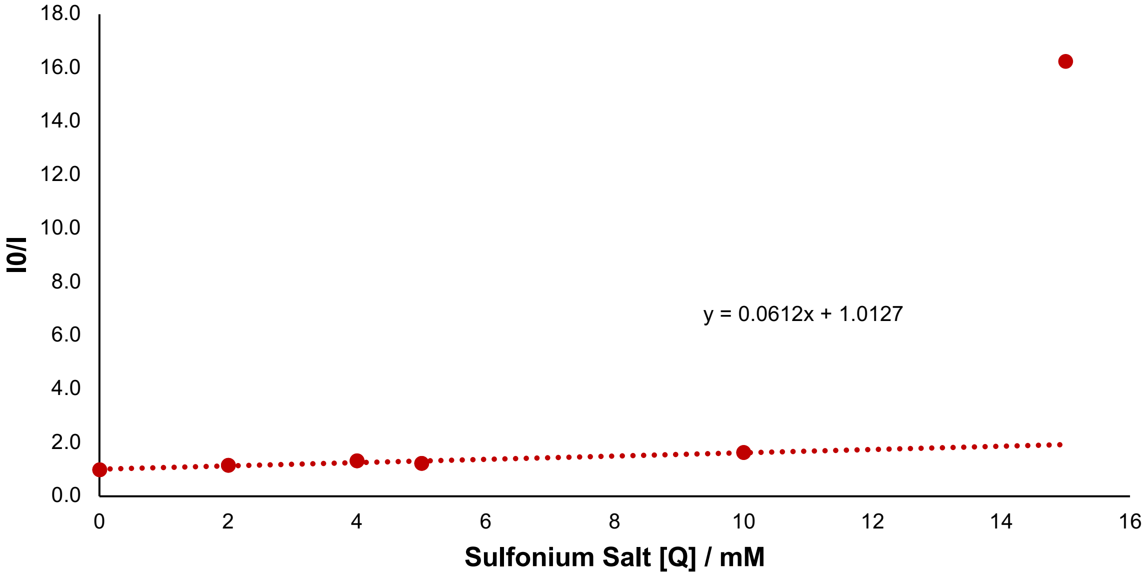

Supplementary Figure S19: *Stern-Volmer plot for PTH in MeCN (0.05 mM) with varying concentrations of α-amido sulfonium salt **2a**.*

The Stern-Volmer plot of PTH displays a non-linear relationship (*vide supra)*. At lower concentrations (up to 10 mM) a linear relationship can be approximated, suggesting quenching of PTH's excited state. The quenching constant is calculated as K_{SV} = 61.2 M^{-1}.

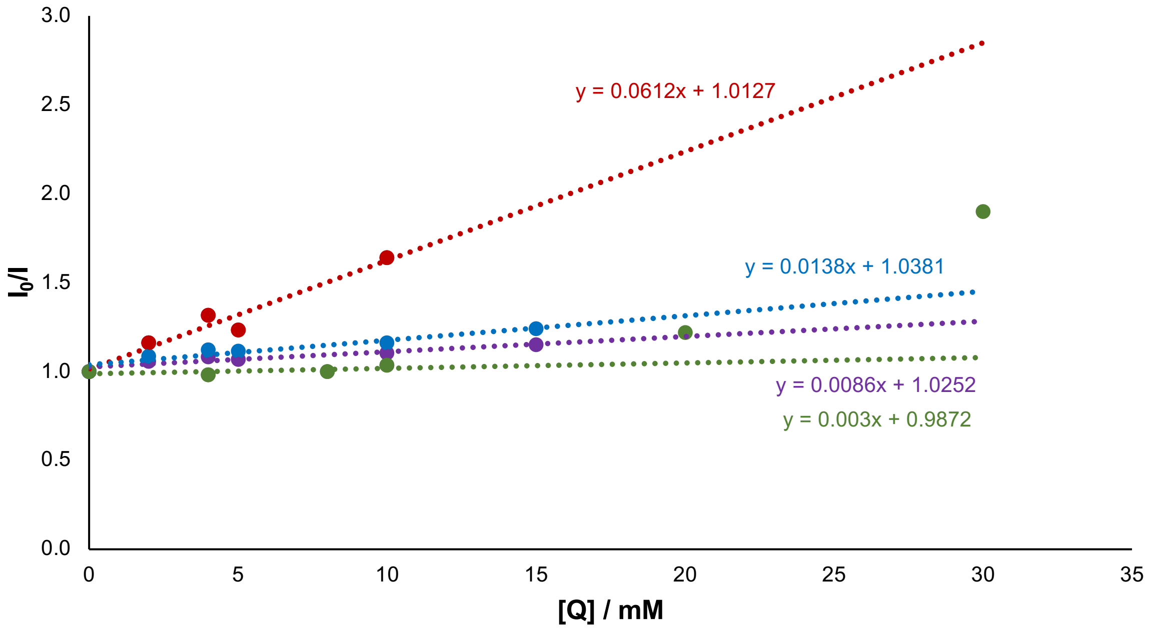

Supplementary Figure S20: *Combined Stern-Volmer plot for PTH in MeCN (0.05 mM) with each reaction substrate.*

The combined Stern-Volmer plots for each substrate (displaying the linear and approximated linear relationships) suggests that although weak quenching with other substrates is possible, α-amido sulfonium salt is the strongest quencher of the excited state of PTH.

8 X-Ray Structures

Data Collection

X-ray data were collected and reduced using CrysAlisPro v41. Absorption correction was performed using empirical methods (SCALE3 ABSPACK) based upon symmetryequivalent reflections combined with measurements at different azimuthal angles. The crystal structures were solved using ShelXT and refined against all F^2 values using the SHELXL implemented through Olex2. All the non-hydrogen atoms were refined anisotropically. Hydrogen atoms were placed in calculated positions refined using idealized geometries (riding model) and assigned fixed isotropic displacement parameters. These data sets can be obtained free of charge via www.ccdc.cam.ac.uk/conts/retrieving.html (or from the Cambridge Crystallographic Data Centre, 12 Union Road, Cambridge CB21EZ, UK; fax: (+44)1223 336033; or deposit@ccdc.cam.ac.uk).

Crystal Structure Determinations and Refinements

X-ray data were collected and reduced using CrysAlisPro v41. Absorption correction was performed using empirical methods (SCALE3 ABSPACK) based upon symmetryequivalent reflections combined with measurements at different azimuthal angles. The crystal structures were solved using ShelXT and refined against all F2 values using the SHELXL implemented through Olex2. All the non-hydrogen atoms were refined anisotropically. Hydrogen atoms were placed in calculated positions refined using idealized geometries (riding model) and assigned fixed isotropic displacement parameters. These data sets can be obtained free of charge via www.ccdc.cam.ac.uk/conts/retrieving.html (or from the Cambridge Crystallographic Data Centre, 12 Union Road, Cambridge CB21EZ, UK; fax: (+44)1223 336033; or deposit@ccdc.cam.ac.uk).

X-ray structure of **2a** – CCDC: 2362276

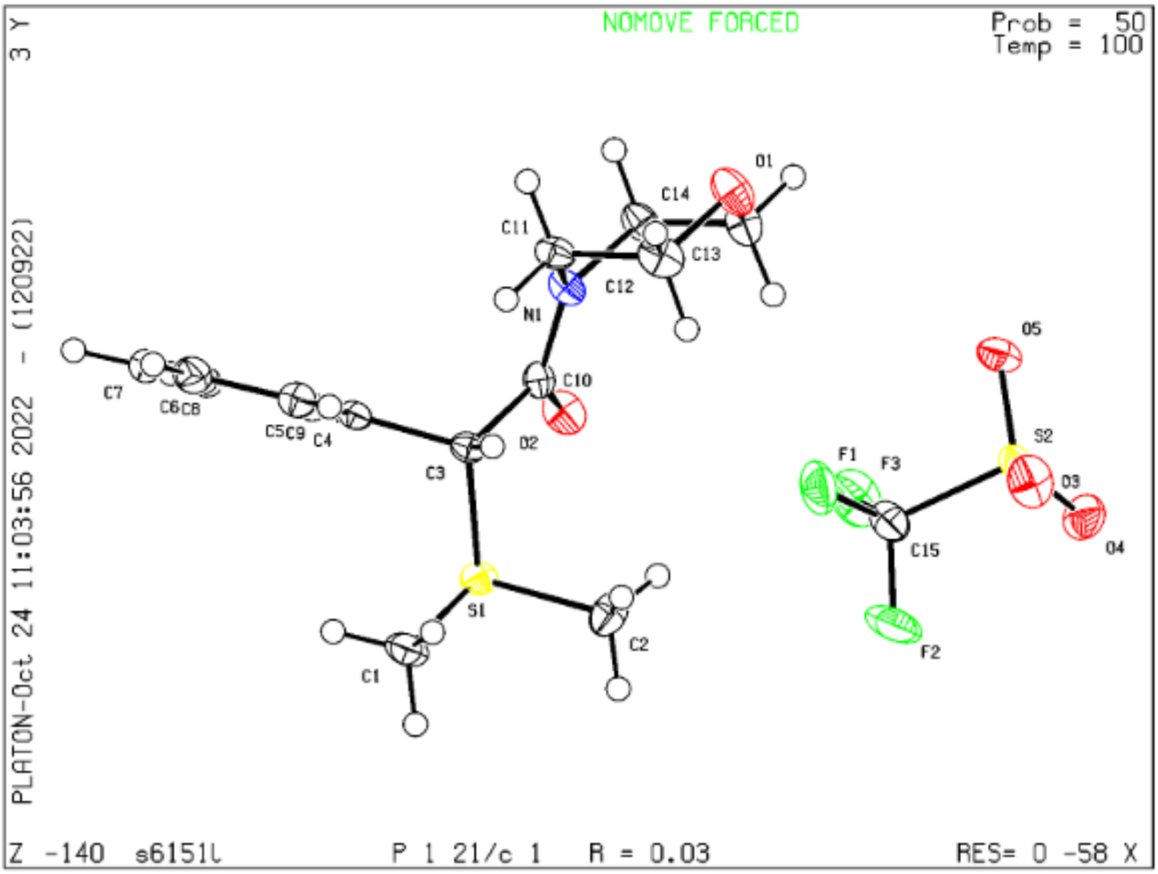

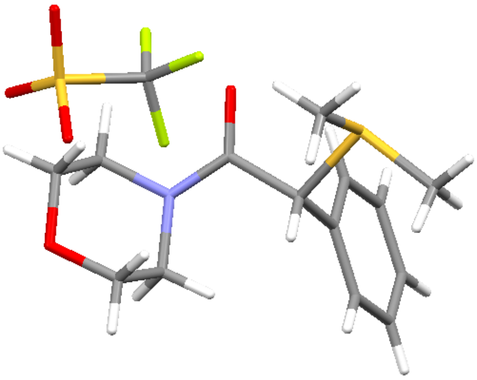

**Identification code**	*(2-Morpholino-2-oxo-1-phenylethyl) Dimethylsulfonium trifluoromethanesulfonate (**2a**)*
**Empirical Formula**	$C_{15}H_{20}F_3NO_5S_2$
**Formula weight**	415.44
**Temperature / K**	100
**Crystal system**	Monoclinic
**Space group**	P 1 2_1/c 1
**a / Å**	10.1937 (3)
**b / Å**	10.7384 (3)
**c / Å**	17.2077 (5)
**α / °**	90
**β / °**	102.113(3)
**γ / °**	90
**Volume / Å³**	1841.68(9)
**Z**	4
**P_{calc}g / cm³**	1.498
**μ / mm⁻¹**	3.147
**F(000)**	864.0
**Radiation**	CuKα (λ = 1.54184)
**2Θ range for data collection/°**	8.872 to 151.610
**Index ranges**	-12 ≤ h ≤ 12, -11 ≤ k ≤ 13, -19 ≤ l ≤ 21
**Reflections collected**	11270
**Independent reflections**	3729
**Data/restraints/parameters**	3729/0/237
**Goodness-of-fit on F^2**	1.083
**Final R indexes [I>=2σ (I)]**	R_1= 0.0306 wR_2= 0.0823
**Final R indexes [all data]**	R_1= 0.0332 wR_2= 0.0811

Supplementary Table S17: *Crystal data and structure refinement for **2a***

X-ray structure of **2c** – CCDC: 2362286

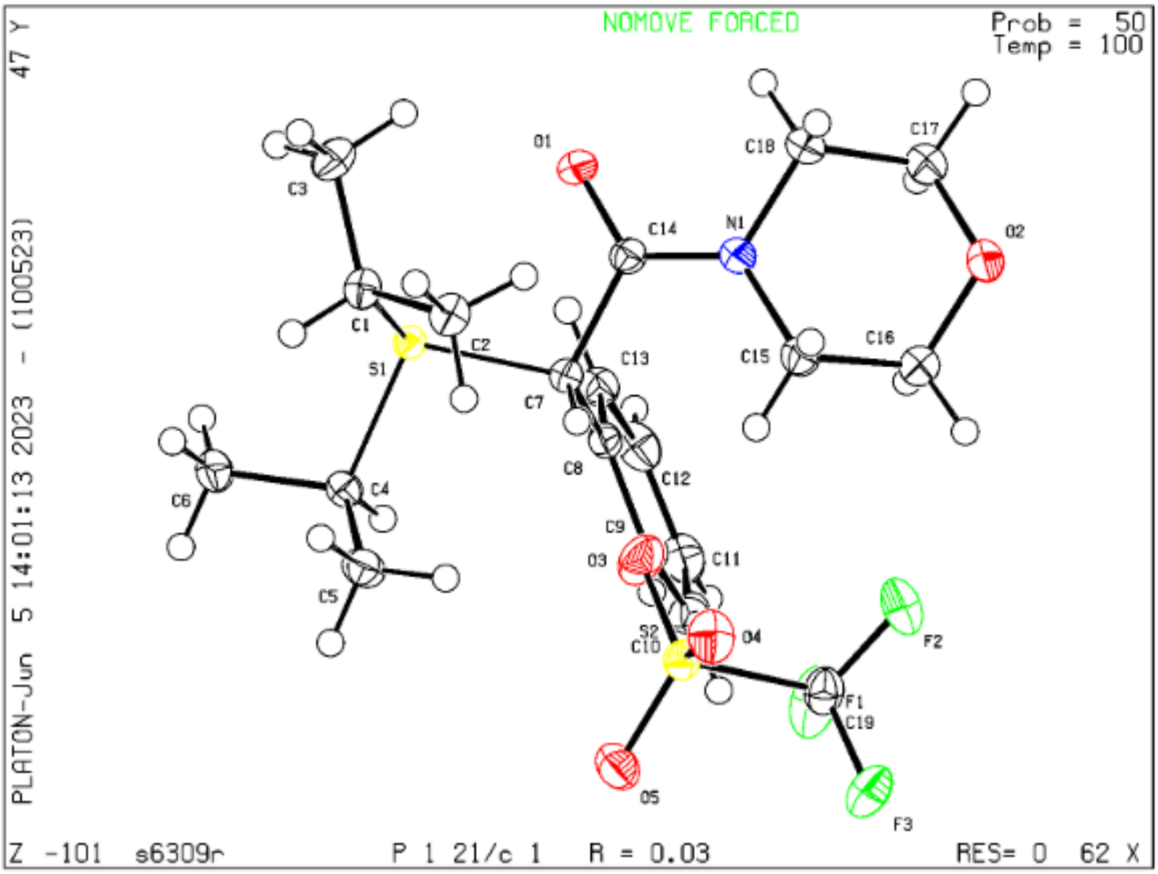

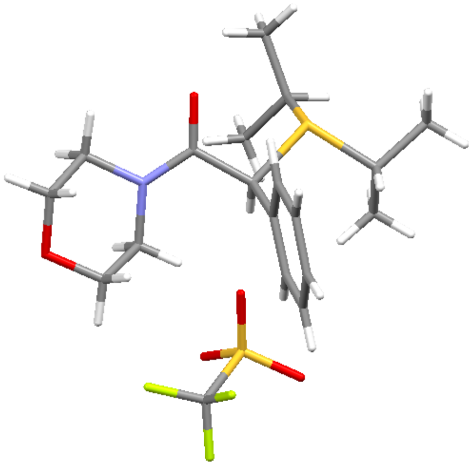

**Identification code**	*(2-Morpholino-2-oxo-1-phenylethyl)diisopropyl sulfonium trifluoromethanesulfonate (**2c**)*
**Empirical Formula**	$C_{19}H_{28}NF_3O_5S_2$
**Formula weight**	470.98
**Temperature/K**	100
**Crystal system**	Monoclinic
**Space group**	P 1 2_1/c 1
**a/Å**	9.03620 (12)
**b/Å**	10.27584 (15)
**c/Å**	23.8937 (3)
**α/°**	90
**β/°**	91.8736(11)
**γ/°**	90
**Volume/Å³**	2217.46 (5)
**Z**	4
**P_{calc}g/cm³**	1.411
**μ/mm⁻¹**	2.664
**F(000)**	991.0
**Radiation**	CuKα (λ = 1.54184)
**2Θ range for data collection/°**	7.404 to 152.028
**Index ranges**	-11 ≤ h ≤ 10, -12 ≤ k ≤ 12, -30 ≤ l ≤ 30
**Reflections collected**	33189
**Independent reflections**	4575
**Data/restraints/parameters**	4575/0/385
**Goodness-of-fit on F²**	1.038
**Final R indexes [I>=2σ (I)]**	R_1= 0.0278 wR_2= 0.0703
**Final R indexes [all data]**	R_1= 0.0286 wR_2= 0.0698

Supplementary Table S18*: Crystal data and structure refinement for 52c.*

X-ray structure of **2l** – CCDC: 2362287

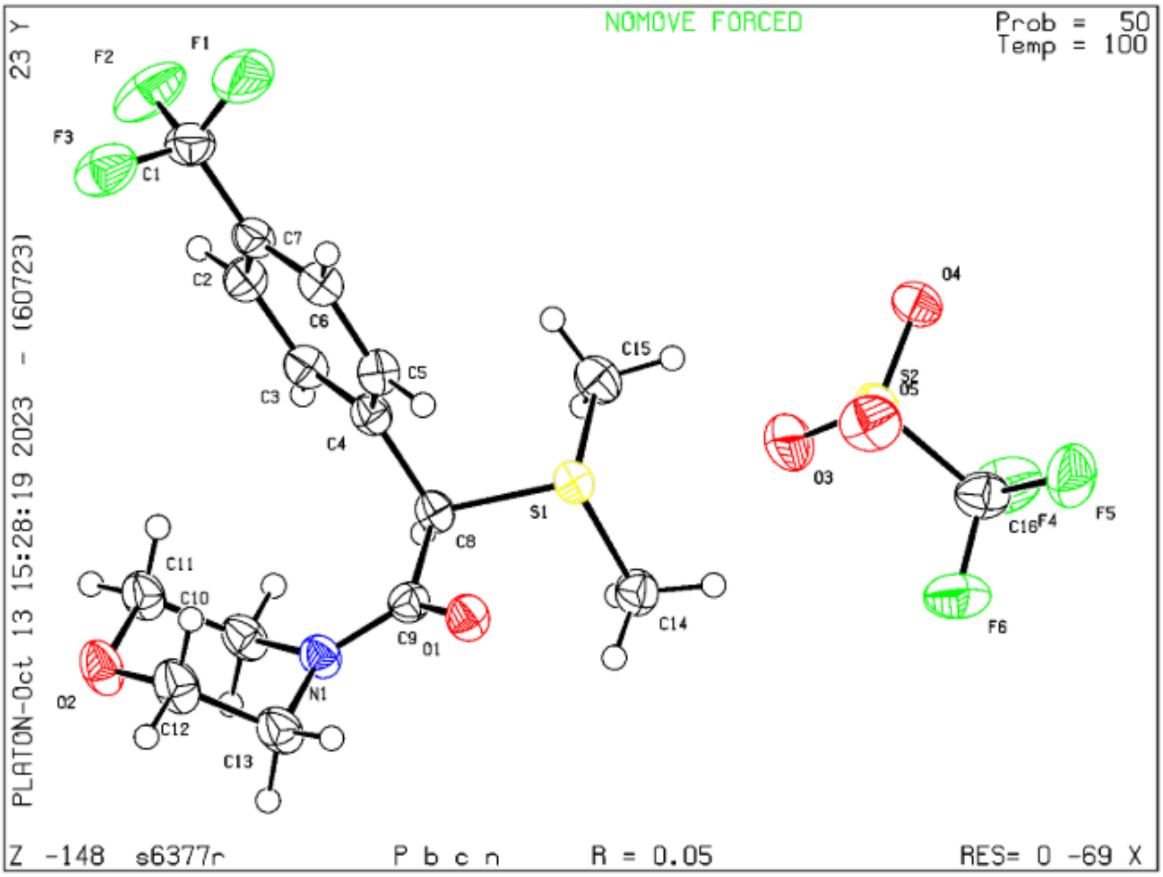

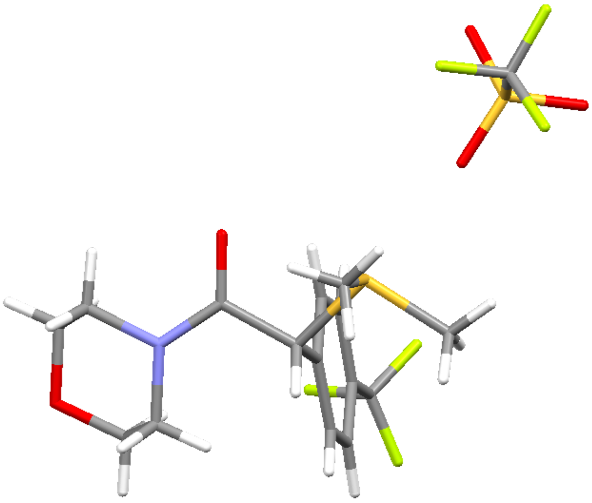

**Identification code**	*Dimethyl(2-morpholino-2-oxo-1-(4-(trifluoromethyl)phenyl)ethyl)sulfonium trifluoromethanesulfonate (**2l**)*
**Empirical Formula**	$C_{16}H_{19}F_6NO_5S_2$
**Formula weight**	483.44
**Temperature/K**	100
**Crystal system**	Orthorhombic
**Space group**	P b c n
**a/Å**	22.7868 (9)
**b/Å**	10.8305 (3)
**c/Å**	16.1252 (5)
**α/°**	90
**β/°**	90
**γ/°**	90
**Volume/Å^3**	3979.6 (2)
**Z**	8
**P_{calc}g/cm^3**	1.614
**μ/mm^{-1}**	3.231
**F(000)**	1984.0
**Radiation**	CuKα (λ = 1.54184)
**2Θ range for data collection/°**	7.724 to 153.1540
**Index ranges**	$-28 \le h \le 27$, $-13 \le k \le 12$, $-18 \le l \le 20$
**Reflections collected**	9561
**Independent reflections**	4134
**Data/restraints/parameters**	4134/0/273
**Goodness-of-fit on F^2**	1.079
**Final R indexes [I>=2σ (I)]**	R_1= 0.0522 wR_2= 0.1481
**Final R indexes [all data]**	R_1= 0.0586 wR_2= 0.1433

*Supplementary Table S19: Crystal data and structure refinement for **2l**.*

X-ray structure of **2s** – CCDC: 2362275

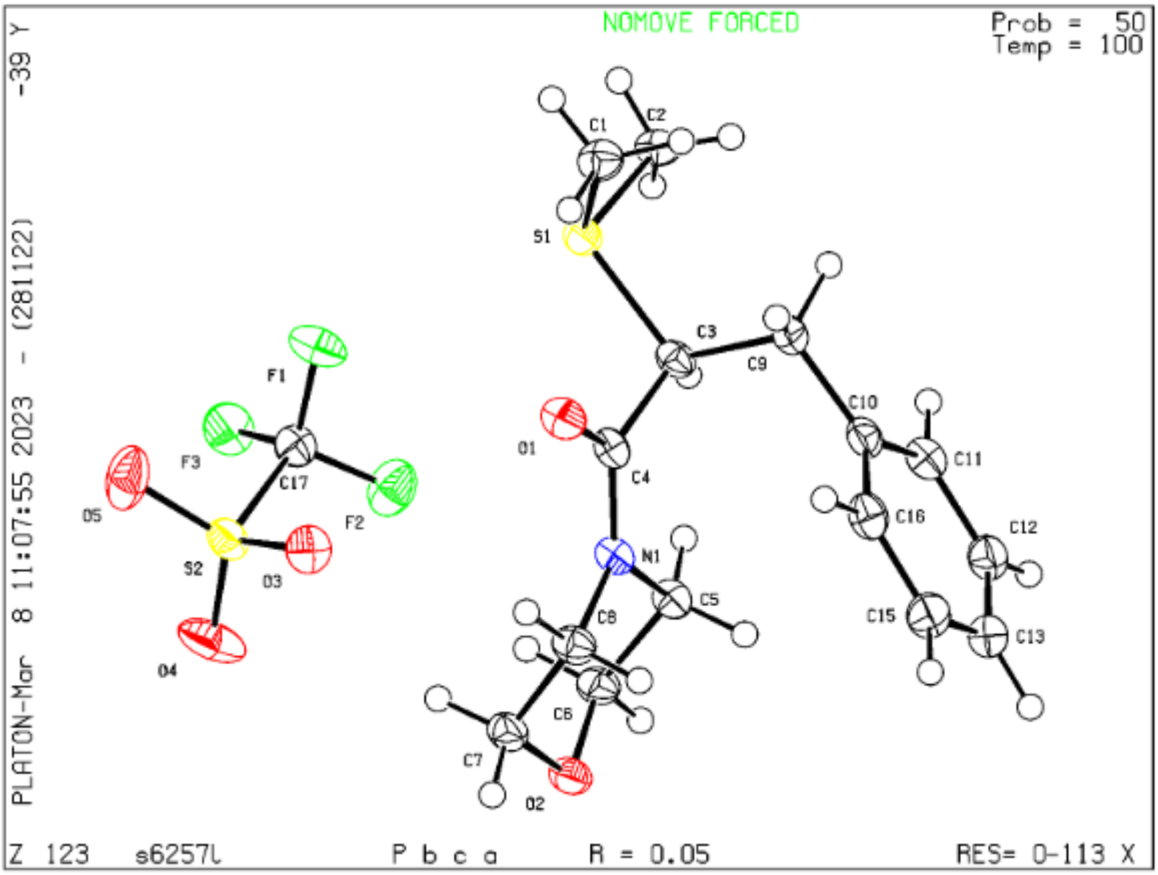

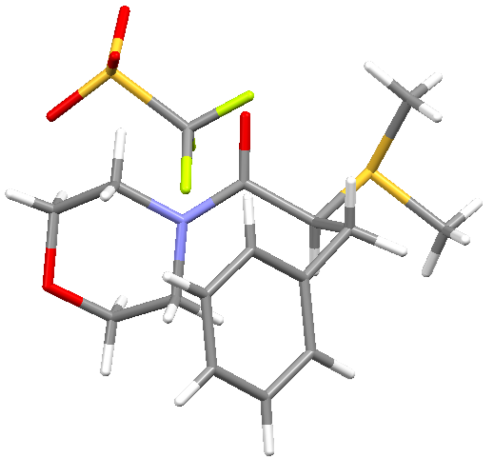

**Identification code**	*(1-morpholino-1-oxo-3-phenylpropan-2-yl) dimethylsulfonium trifluoromethanesulfonate (**2u**)*
**Empirical Formula**	$C_{16}H_{22}F_3NO_5S_2$
**Formula weight**	429.46
**Temperature/K**	100
**Crystal system**	Orthorhombic
**Space group**	P b c a
**a/Å**	8.4167 (3)
**b/Å**	12.4608 (4)
**c/Å**	35.7313 (12)
**α/°**	90
**β/°**	90
**γ/°**	90
**Volume/Å^3**	3747.5 (2)
**Z**	8
**P_{calc}g/cm^3**	1.522
**μ/mm^{-1}**	3.112
**F(000)**	1792.0
**Radiation**	CuKα (λ = 1.54184)
**2Θ range for data collection/°**	4.946 to 152.824
**Index ranges**	$-9 \leq h \leq 7$, $-15 \leq k \leq 15$, $-44 \leq l \leq 35$
**Reflections collected**	4665
**Independent reflections**	3804
**Data/restraints/parameters**	3804/0/332
**Goodness-of-fit on F^2**	1.072
**Final R indexes [I>=2σ (I)]**	R_1= 0.0479 wR_2= 0.1255
**Final R indexes [all data]**	R_1= 0.0583 wR_2= 0.1196

Supplementary Table S20: *Crystal data and structure refinement for **2s**.*

9 References

[1] B. D. A. Shennan, S. Sánchez-Alonso, G. Rossini, D. J. Dixon, *J. Am. Chem. Soc.* **2023**, *145*, 21745–21751.

[2] C. Heyde, I. Zug, H. Hartmann, *Eur. J. Org. Chem.* **2000**, *2000*, 3273–3278.

[3] W. Zhang, J. M. Ready, *Angew. Chem. Int. Ed.* **2014**, *53*, 8980–8984.

[4] N. Oku, M. Murakami, T. Miura, *Org. Lett.* **2022**, *24*, 1616–1619.

[5] D. Djukanovic, M. A. Ganiek, K. Nishi, K. Karaghiosoff, K. Mashima, P. Knochel, *Angew. Chem. Int. Ed.* **2022**, *61*, e202205440.

[6] M.-E. Trân-Huu-Dâu, R. Wartchow, E. Winterfeldt, Y.-S. Wong, *Chem. – Eur. J.* **2001**, *7*, 2349–2369.

[7] Z. Wu, J. D. Laffoon, T. T. Nguyen, J. D. McAlpin, K. L. Hull, *Angew. Chem.* **2017**, *129*, 1391–1395.

[8] J. Rodrigalvarez, H. Wang, R. Martin, *J. Am. Chem. Soc.* **2023**, *145*, 3869–3874.

[9] M. A. Bigi, M. C. White, *J. Am. Chem. Soc.* **2013**, *135*, 7831–7834.

[10] H. Yu, B. Gao, B. Hu, H. Huang, *Org. Lett.* **2017**, *19*, 3520–3523.

[11] M. C. D'Amaral, N. Jamkhou, M. J. Adler, *Green Chem.* **2021**, *23*, 288–295.

[12] N. Duchemin, R. Buccafusca, M. Daumas, V. Ferey, S. Arseniyadis, *Org. Lett.* **2019**, *21*, 8205–8210.

[13] D. R. Dreyer, H.-P. Jia, A. D. Todd, J. Geng, C. W. Bielawski, *Org. Biomol. Chem.* **2011**, *9*, 7292–7295.

[14] M. Gao, Y. Tian, X. Li, Y. Gong, M. Fang, J. Yang, Z. Li, *Angew. Chem. Int. Ed.* **2023**, *62*, e202214908.

[15] A. Dewanji, L. van Dalsen, J. A. Rossi-Ashton, E. Gasson, G. E. M. Crisenza, D. J. Procter, *Nat. Chem.* **2023**, *15*, 43–52.

[16] B. Brooks, N. Hiller, J. A. May, *Tetrahedron Lett.* **2021**, *83*, 153412.

[17] K. Brak, J. A. Ellman, *J. Am. Chem. Soc.* **2009**, *131*, 3850–3851.

[18] S. Liao, A. Porta, X. Cheng, X. Ma, G. Zanoni, L. Zhang, *Angew. Chem.* **2018**, *130*, 8382–8386.

[19] M. Leypold, K. A. D'Angelo, M. Movassaghi, *Org. Lett.* **2020**, *22*, 8802–8807.

[20] C. R. Gonçalves, M. Lemmerer, C. J. Teskey, P. Adler, D. Kaiser, B. Maryasin, L. González, N. Maulide, *J. Am. Chem. Soc.* **2019**, *141*, 18437–18443.

[21] C. G. Hatchard, C. A. Parker, *Proc. R. Soc. Lond. Ser. Math. Phys. Sci.* **1956**, *235*, 518–536.

[22] M. A. Cismesia, T. P. Yoon, *Chem. Sci.* **2015**, *6*, 5426–5434.

[23] J. R. Lakowicz, Ed. , *Principles of Fluorescence Spectroscopy*, Springer US, Boston, MA, **2006**.

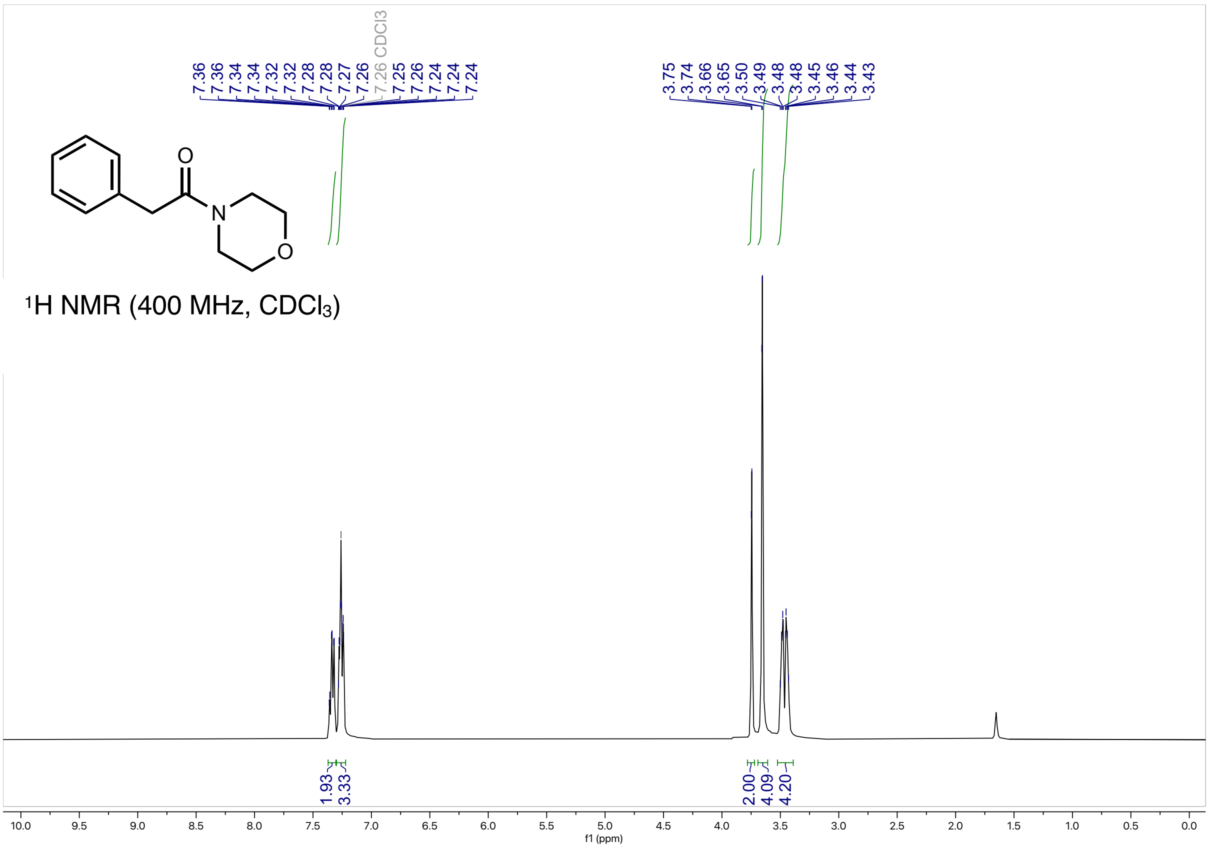
7.36
7.36
7.34
7.34
7.32
7.32
7.28
7.28
7.27
7.26
7.26 CDCl3
7.25
7.26
7.24
7.24
7.24
3.75
3.74
3.66
3.65
3.50
3.49
3.48
3.48
3.48
3.45
3.46
3.44
3.43
O
N
O
1H NMR (400 MHz, CDCl3)
1.93
3.33
2.00
4.09
4.20
10.0
9.5
9.0
8.5
8.0
7.5
7.0
6.5
6.0
5.5
5.0
4.5
4.0
3.5
3.0
2.5
2.0
1.5
1.0
0.5
0.0
f1 (ppm)

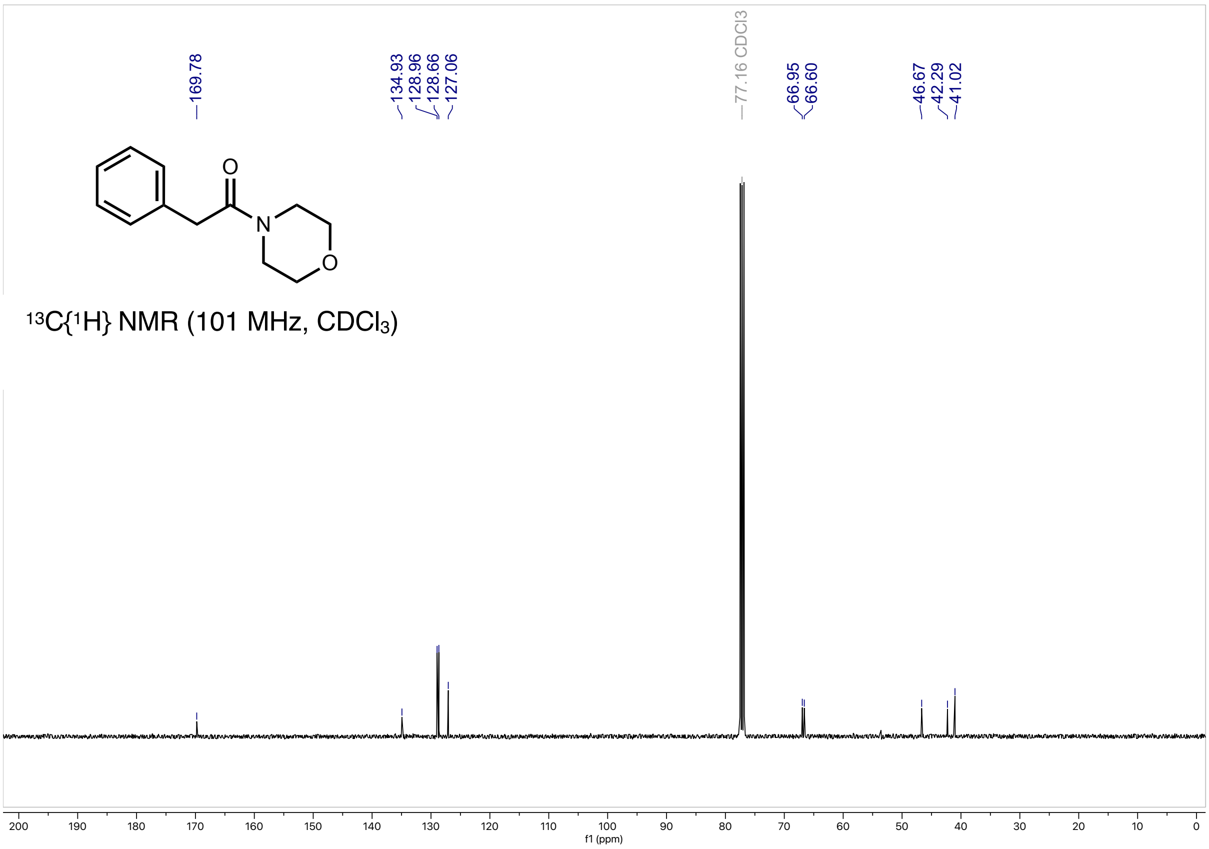
169.78
134.93
128.96
128.66
127.06
77.16 CDCl3
66.95
66.60
46.67
42.29
41.02
O
N
O
13C{1H} NMR (101 MHz, CDCl3)
200
190
180
170
160
150
140
130
120
110
100
90
80
70
60
50
40
30
20
10
0
f1 (ppm)

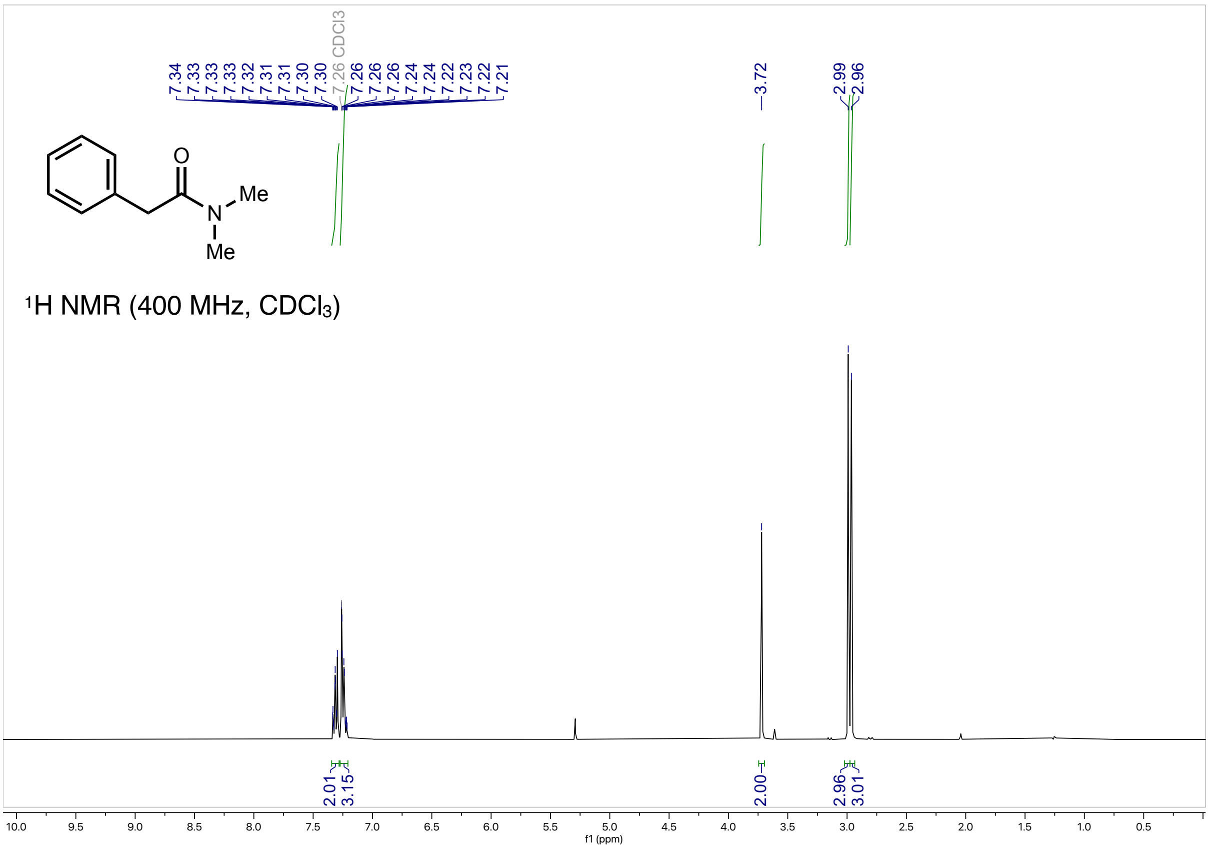

O
N
Me
Me
1H NMR (400 MHz, CDCl3)
7.34
7.33
7.33
7.33
7.32
7.31
7.31
7.30
7.30
7.26 CDCl3
7.26
7.26
7.26
7.24
7.24
7.22
7.22
7.23
7.22
7.21
3.72
2.99
2.96
2.01
3.15
2.00
2.96
3.01
f1 (ppm)

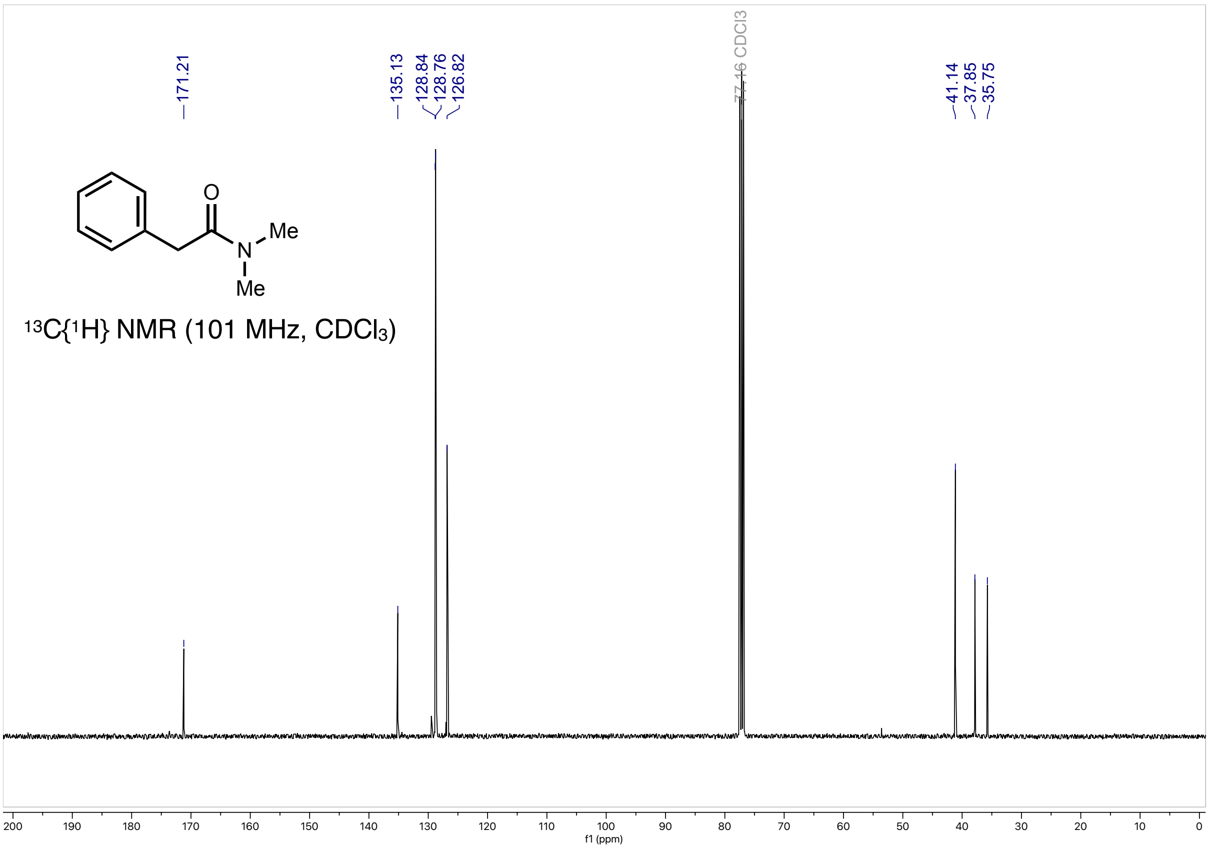

O
N
Me
Me
13C{1H} NMR (101 MHz, CDCl3)
171.21
135.13
128.84
128.76
126.82
77.16 CDCl3
41.14
37.85
35.75
f1 (ppm)

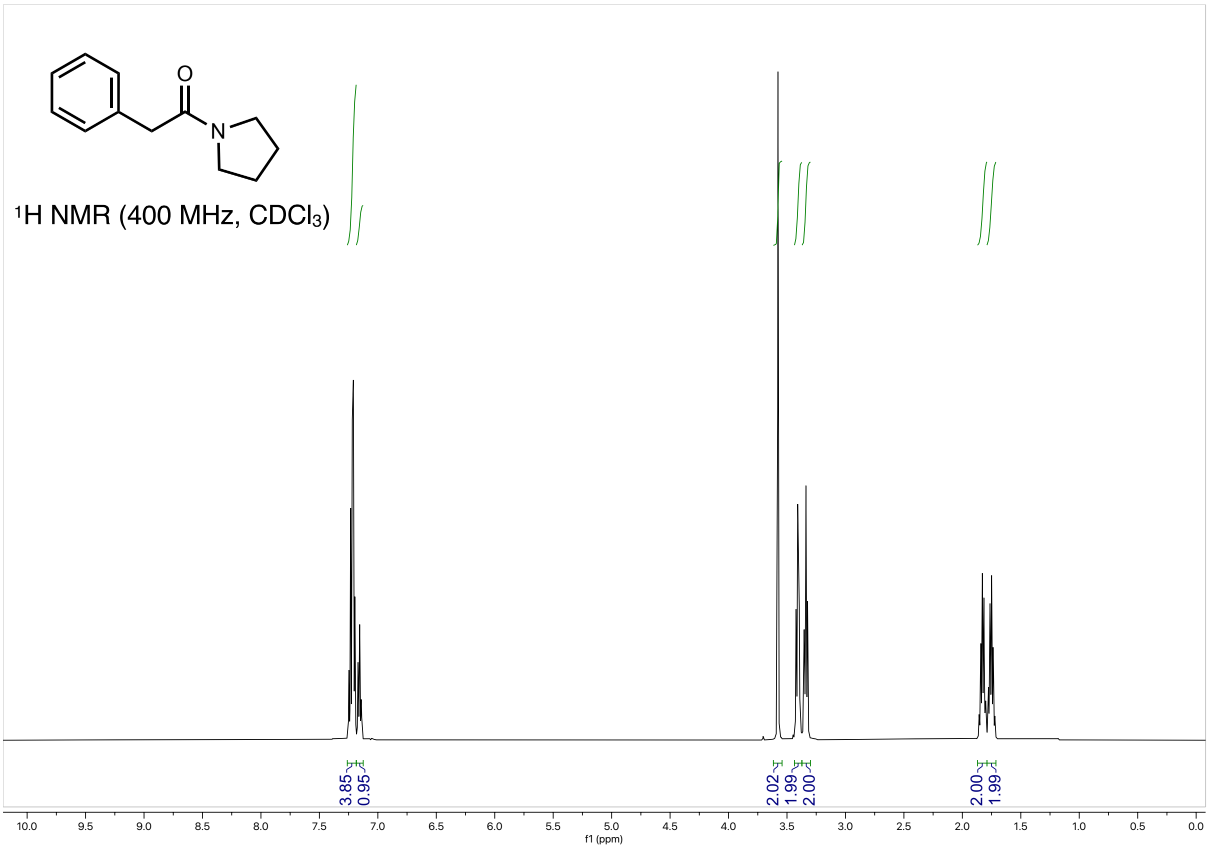
O
N
1H NMR (400 MHz, CDCl3)
3.85
0.95
2.02
1.99
2.00
2.00
1.99
10.0
9.5
9.0
8.5
8.0
7.5
7.0
6.5
6.0
5.5
5.0
4.5
4.0
3.5
3.0
2.5
2.0
1.5
1.0
0.5
0.0
f1 (ppm)

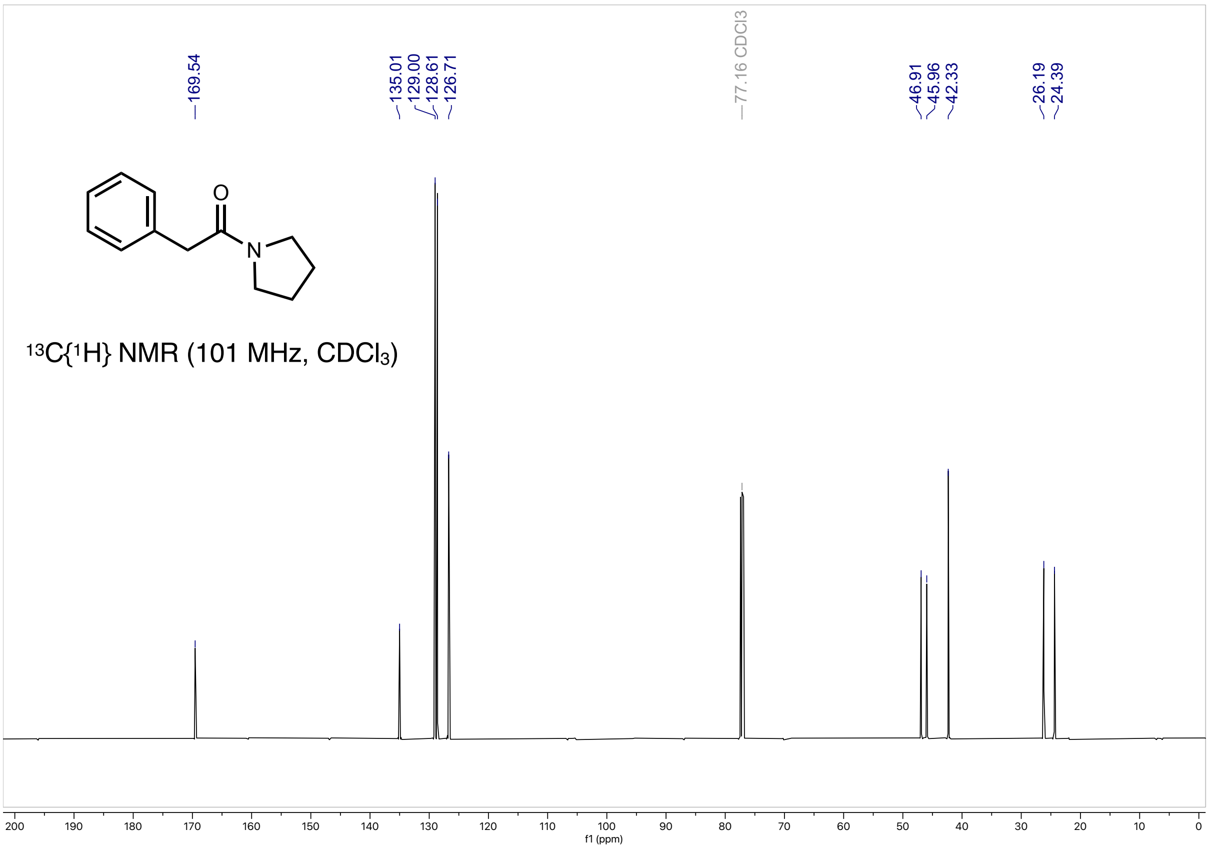
169.54
135.01
129.00
128.61
126.71
77.16 CDCl3
46.91
45.96
42.33
26.19
24.39
O
N
13C{1H} NMR (101 MHz, CDCl3)
200
190
180
170
160
150
140
130
120
110
100
90
80
70
60
50
40
30
20
10
0
f1 (ppm)

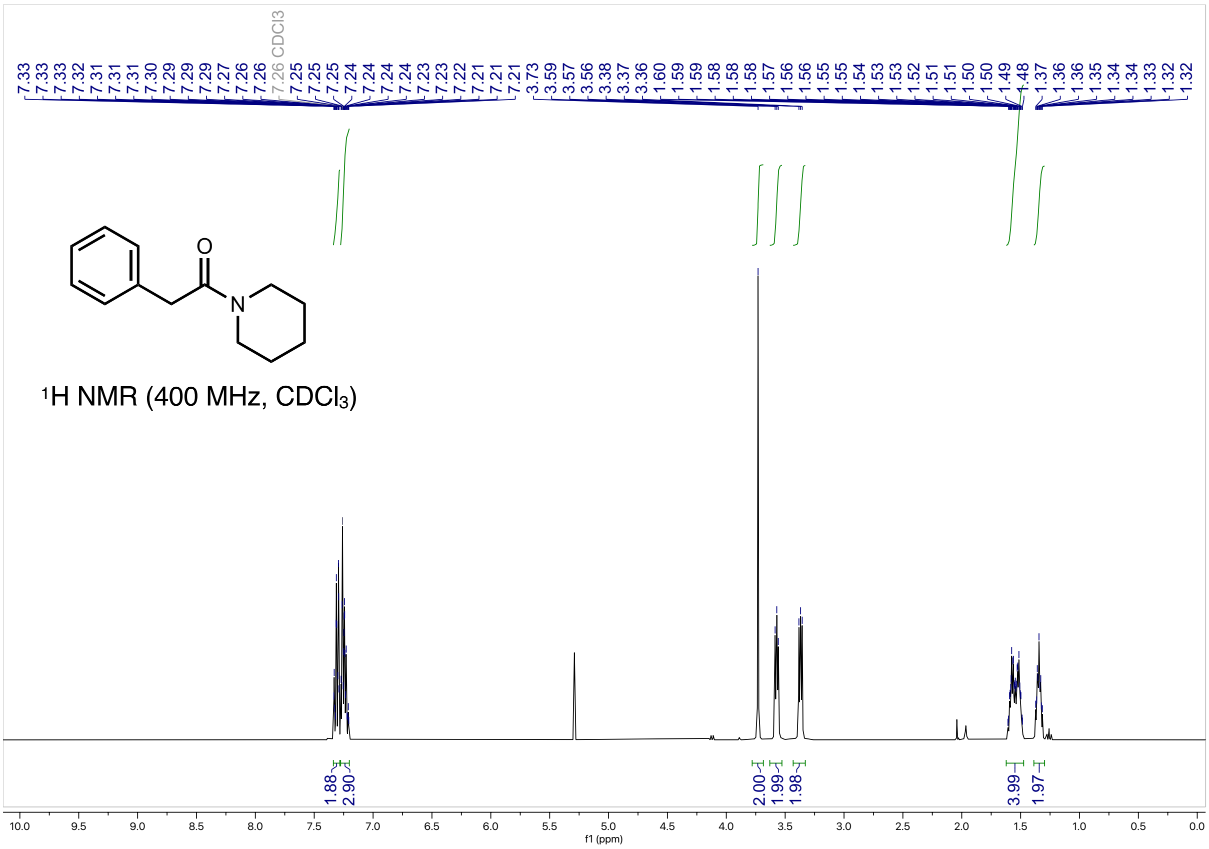

O
N
¹H NMR (400 MHz, CDCl₃)
7.33
7.33
7.32
7.31
7.31
7.30
7.29
7.29
7.29
7.27
7.26
7.26 CDCl3
7.25
7.25
7.25
7.24
7.24
7.24
7.23
7.23
7.22
7.21
7.21
3.73
3.59
3.57
3.56
3.38
3.37
3.36
1.60
1.59
1.59
1.58
1.58
1.57
1.56
1.56
1.55
1.55
1.54
1.53
1.53
1.52
1.51
1.51
1.50
1.50
1.49
1.48
1.37
1.36
1.36
1.35
1.34
1.34
1.33
1.32
1.32
1.88
2.90
2.00
1.99
1.98
3.99
1.97
f1 (ppm)

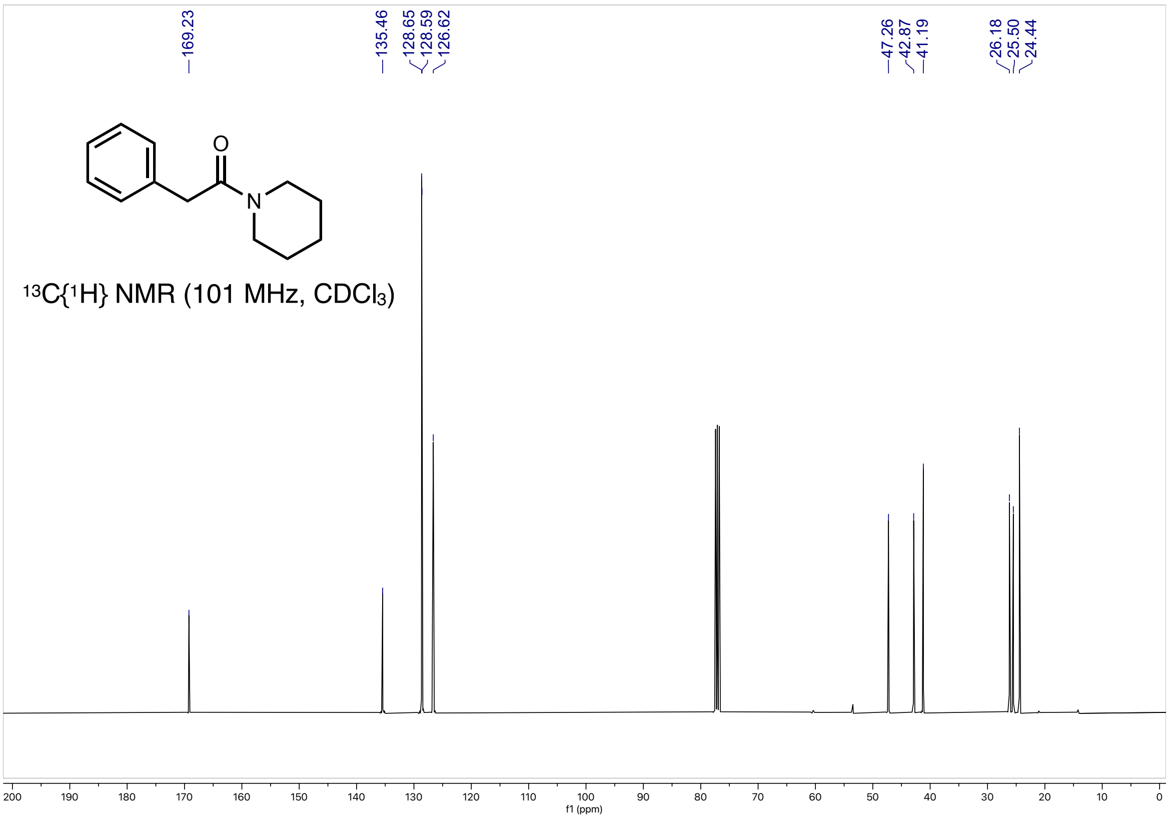

O
N
¹³C{¹H} NMR (101 MHz, CDCl₃)
169.23
135.46
128.65
128.59
126.62
47.26
42.87
41.19
26.18
25.50
24.44
f1 (ppm)

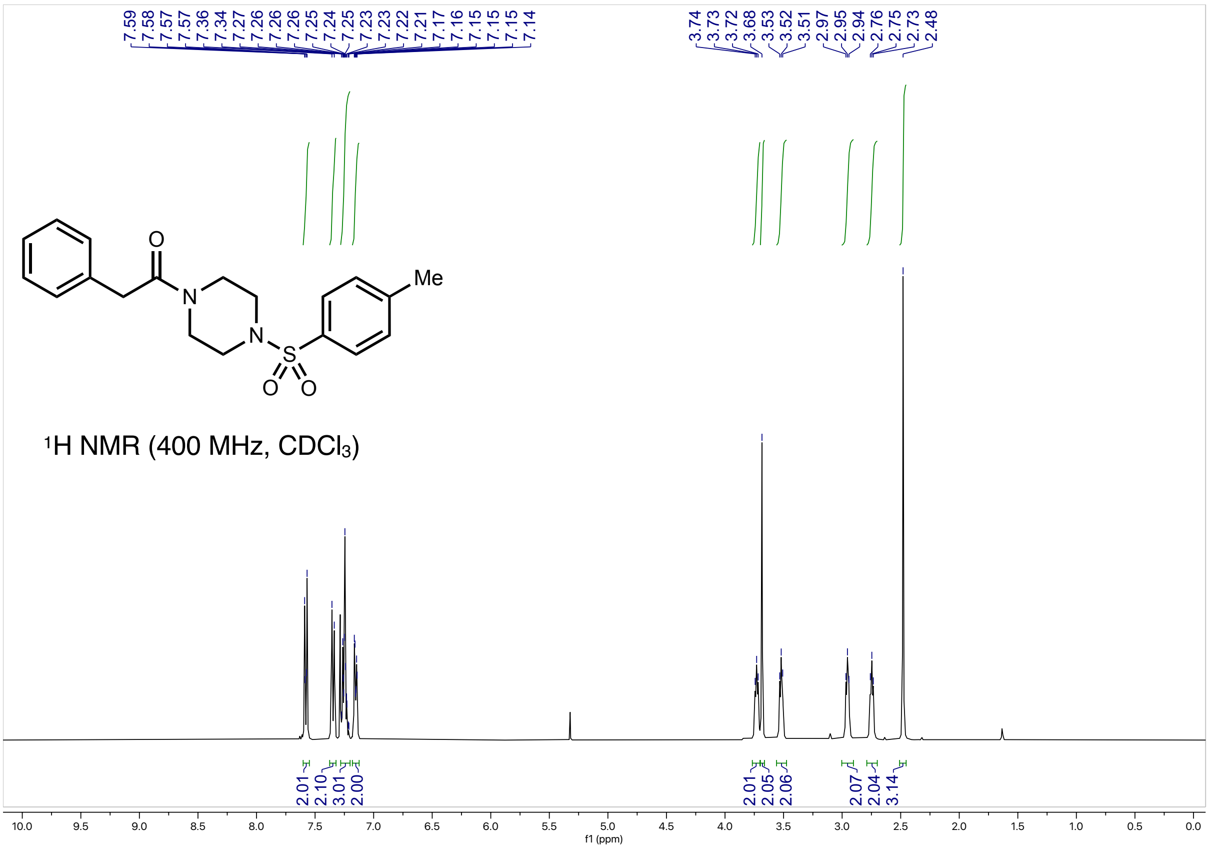

7.59 7.58 7.57 7.57 7.36 7.34 7.27 7.26 7.26 7.25 7.24 7.25 7.23 7.23 7.22 7.21 7.17 7.16 7.15 7.15 7.15 7.14
3.74 3.73 3.72 3.68 3.53 3.52 3.51 2.97 2.95 2.94 2.76 2.75 2.73 2.48
O
N
N
S
O O
Me
¹H NMR (400 MHz, CDCl₃)
2.01 2.10 3.01 2.00
2.01 2.05 2.06
2.07 2.04 3.14
10.0 9.5 9.0 8.5 8.0 7.5 7.0 6.5 6.0 5.5 5.0 4.5 4.0 3.5 3.0 2.5 2.0 1.5 1.0 0.5 0.0
f1 (ppm)

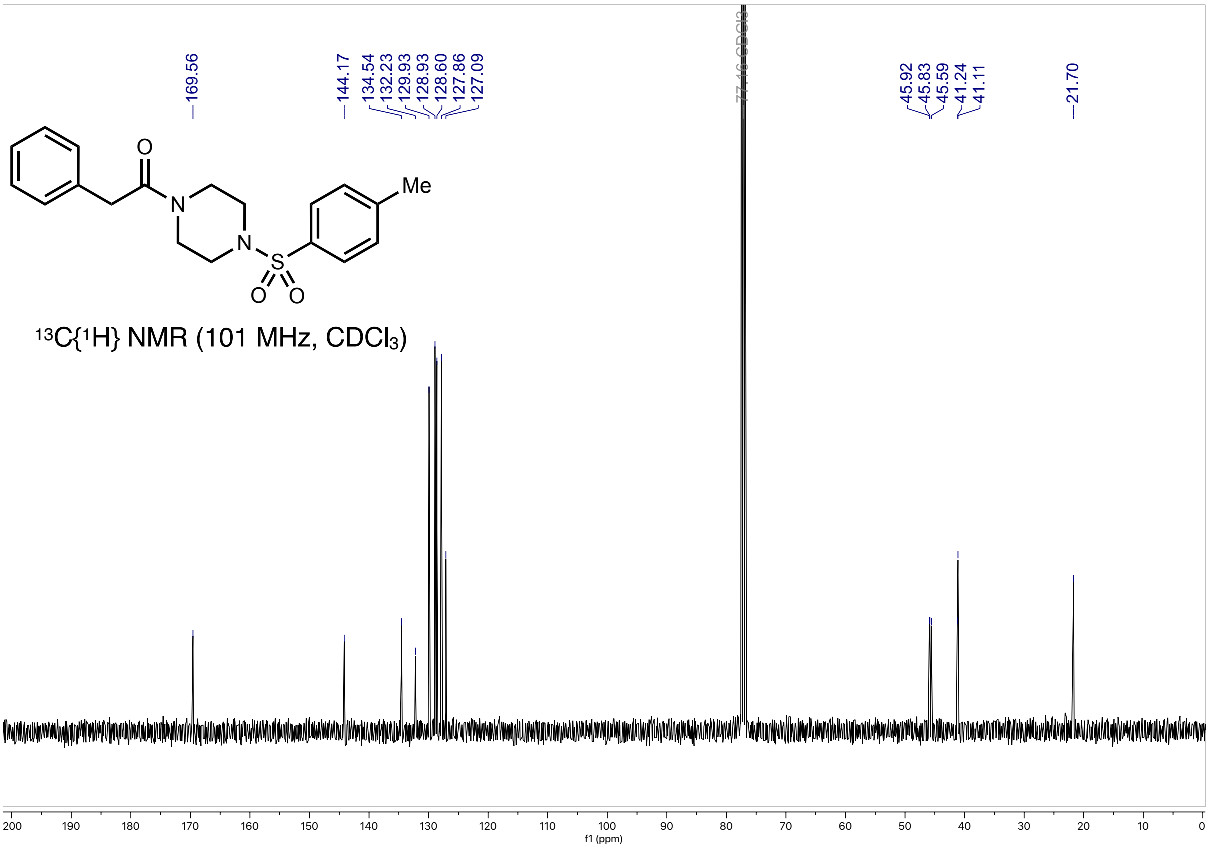

169.56
144.17 134.54 132.23 129.93 128.93 128.60 127.86 127.09
77.16 CDCl3
45.92 45.83 45.59 41.24 41.11
21.70
O
N
N
S
O O
Me
¹³C{¹H} NMR (101 MHz, CDCl₃)
200 190 180 170 160 150 140 130 120 110 100 90 80 70 60 50 40 30 20 10 0
f1 (ppm)

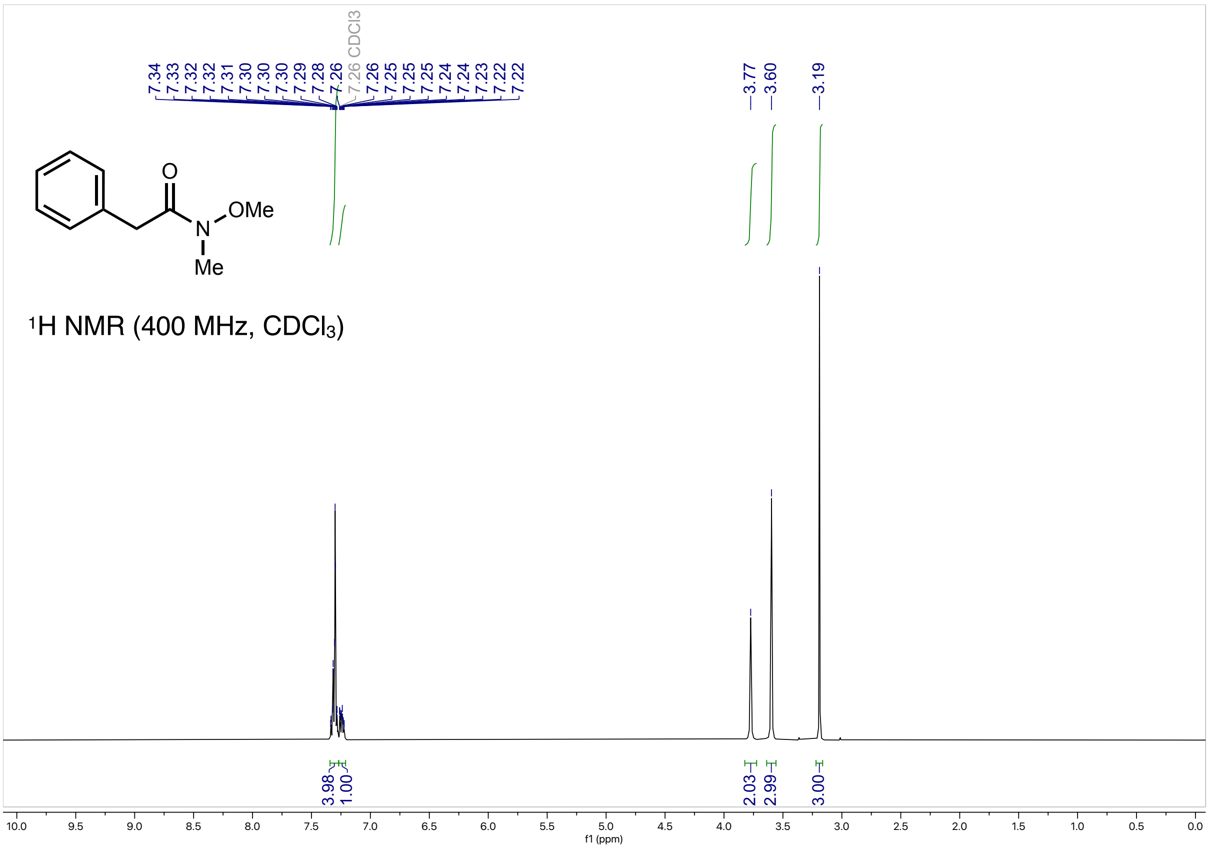

7.34
7.33
7.32
7.32
7.31
7.30
7.30
7.30
7.29
7.28
7.26
7.26 CDCl3
7.26
7.25
7.25
7.25
7.24
7.24
7.23
7.22
7.22
3.77
3.60
3.19
O
OMe
N
Me
¹H NMR (400 MHz, CDCl₃)
3.98
1.00
2.03
2.99
3.00
10.0
9.5
9.0
8.5
8.0
7.5
7.0
6.5
6.0
5.5
5.0
4.5
4.0
3.5
3.0
2.5
2.0
1.5
1.0
0.5
0.0
f1 (ppm)

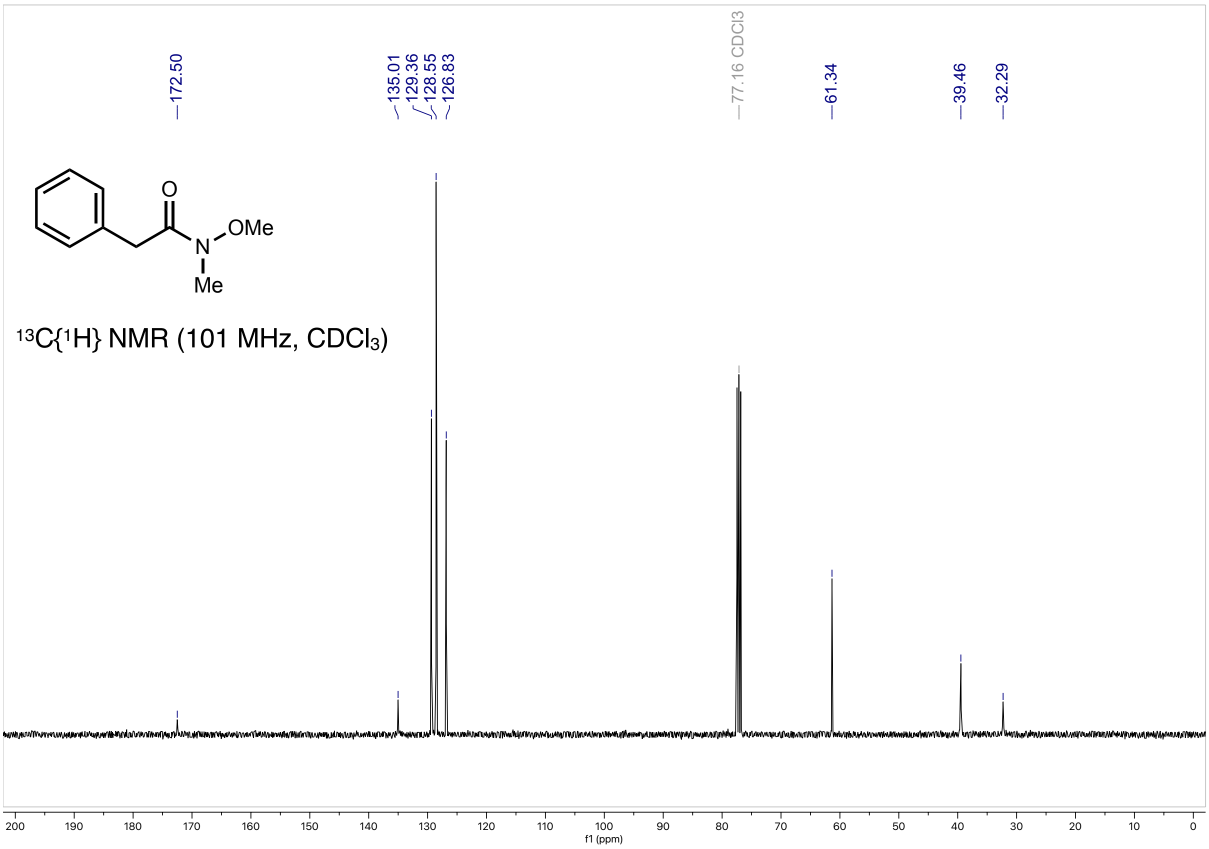

172.50
135.01
129.36
128.55
126.83
77.16 CDCl3
61.34
39.46
32.29
O
OMe
N
Me
¹³C{¹H} NMR (101 MHz, CDCl₃)
200
190
180
170
160
150
140
130
120
110
100
90
80
70
60
50
40
30
20
10
0
f1 (ppm)

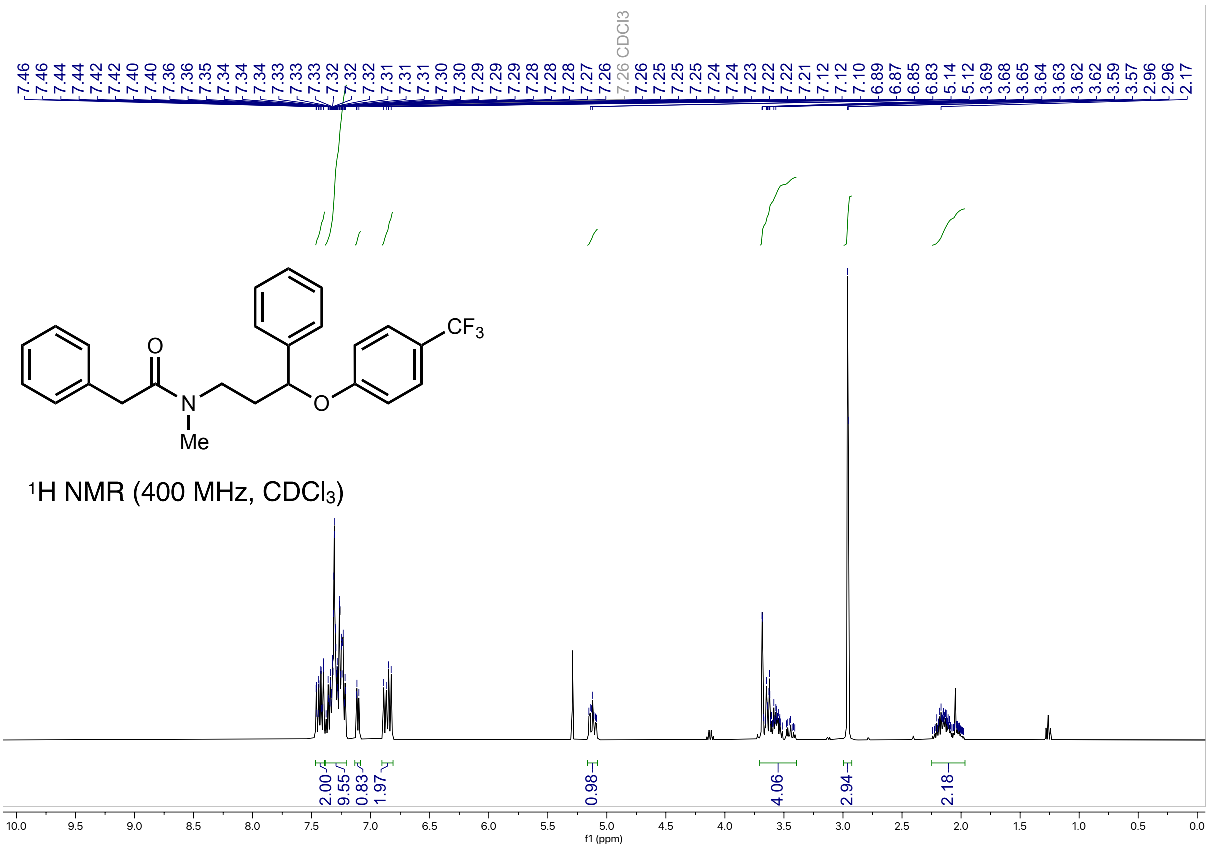
CF3
O
N
Me
O
1H NMR (400 MHz, CDCl3)
7.46 7.46 7.44 7.44 7.42 7.42 7.40 7.40 7.36 7.36 7.35 7.34 7.34 7.34 7.33 7.33 7.33 7.32 7.32 7.32 7.31 7.31 7.31 7.30 7.30 7.29 7.29 7.29 7.28 7.28 7.28 7.27 7.26 CDCl3 7.26 7.25 7.25 7.25 7.24 7.24 7.23 7.22 7.22 7.21 7.12 7.10 6.89 6.87 6.85 6.83 5.14 5.12 3.69 3.68 3.65 3.64 3.63 3.62 3.62 3.59 3.57 2.96 2.96 2.17
2.00 9.55 0.83 1.97 0.98 4.06 2.94 2.18
f1 (ppm)

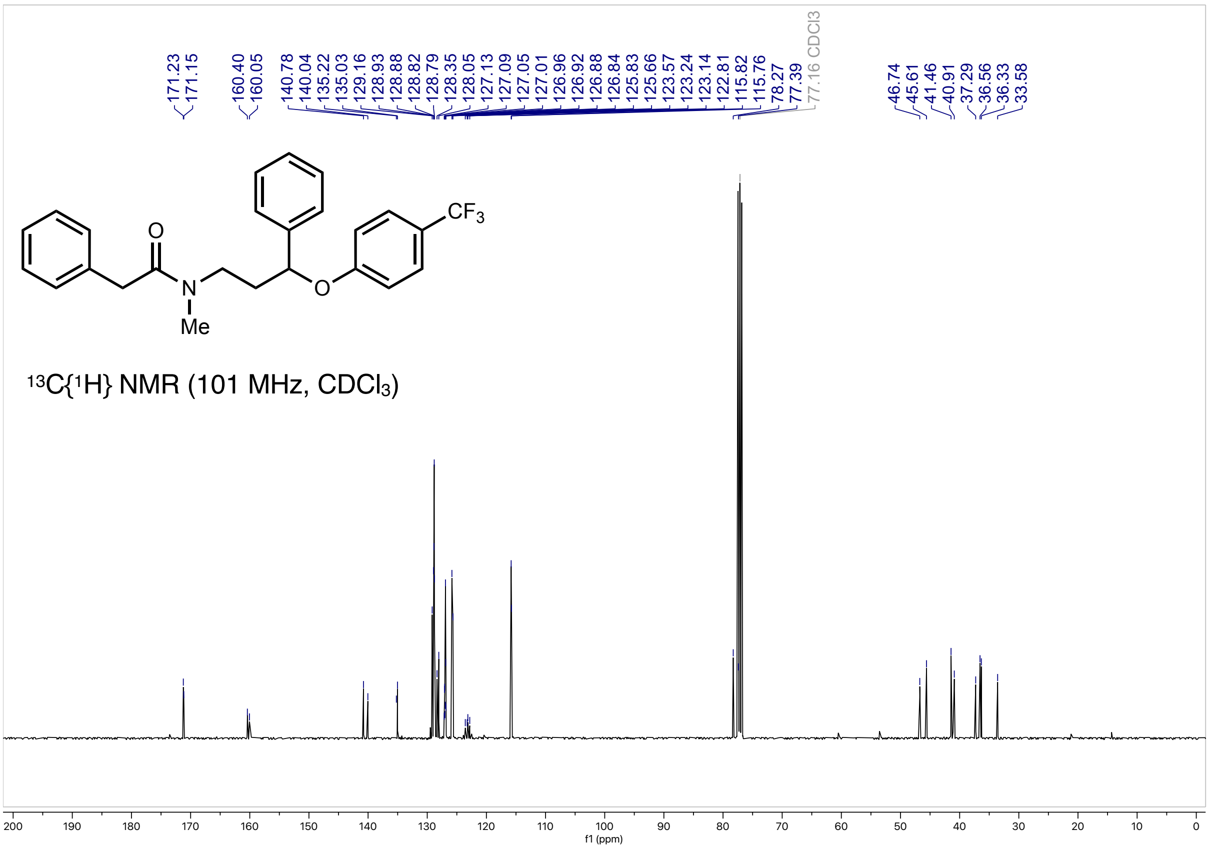
CF3
O
N
Me
O
13C{1H} NMR (101 MHz, CDCl3)
171.23 171.15 160.40 160.05 140.78 140.04 135.22 135.03 129.16 128.93 128.88 128.82 128.79 128.35 128.05 127.13 127.09 127.05 127.01 126.96 126.92 126.88 126.84 125.83 125.66 123.57 123.24 123.14 122.81 115.82 115.76 78.27 77.39 77.16 CDCl3
46.74 45.61 41.46 40.91 37.29 36.56 36.33 33.58
f1 (ppm)

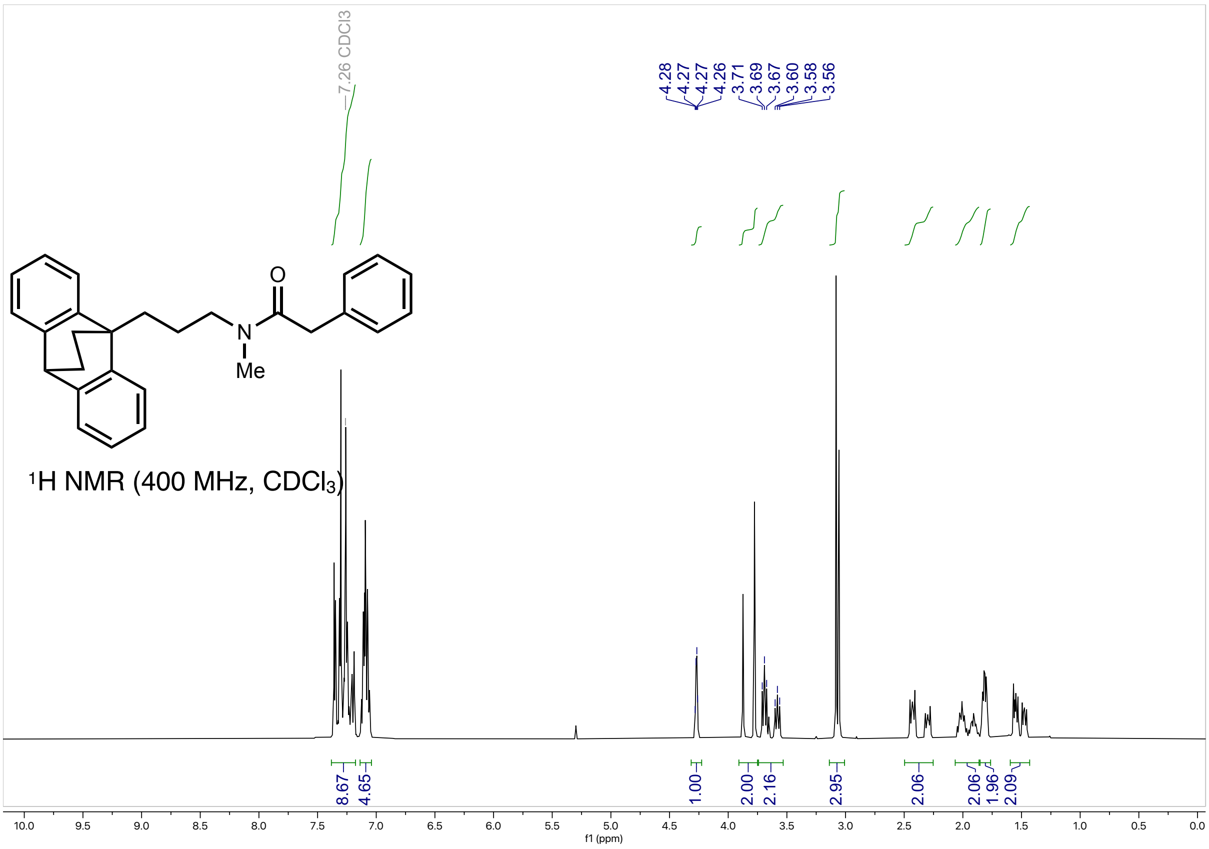

7.26 CDCl3
4.28
4.27
4.27
4.26
3.71
3.69
3.67
3.60
3.58
3.56
O
N
Me
1H NMR (400 MHz, CDCl3)
8.67
4.65
1.00
2.00
2.16
2.95
2.06
2.06
1.96
2.09
f1 (ppm)

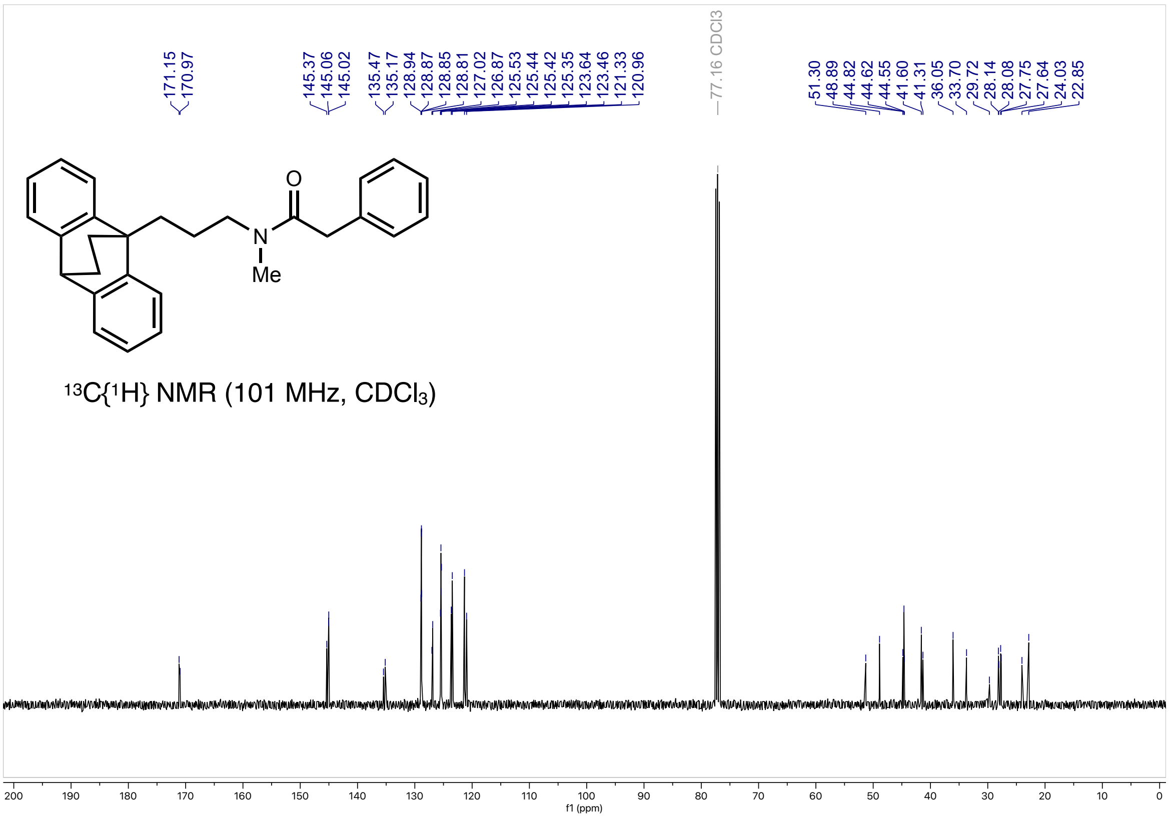

171.15
170.97
145.37
145.06
145.02
135.47
135.17
128.94
128.87
128.85
128.81
127.02
126.87
125.53
125.44
125.42
125.35
123.64
123.46
121.33
120.96
77.16 CDCl3
51.30
48.89
44.82
44.62
44.55
41.60
41.31
36.05
33.70
29.72
28.14
28.08
27.75
27.64
24.03
22.85
O
N
Me
13C{1H} NMR (101 MHz, CDCl3)
f1 (ppm)

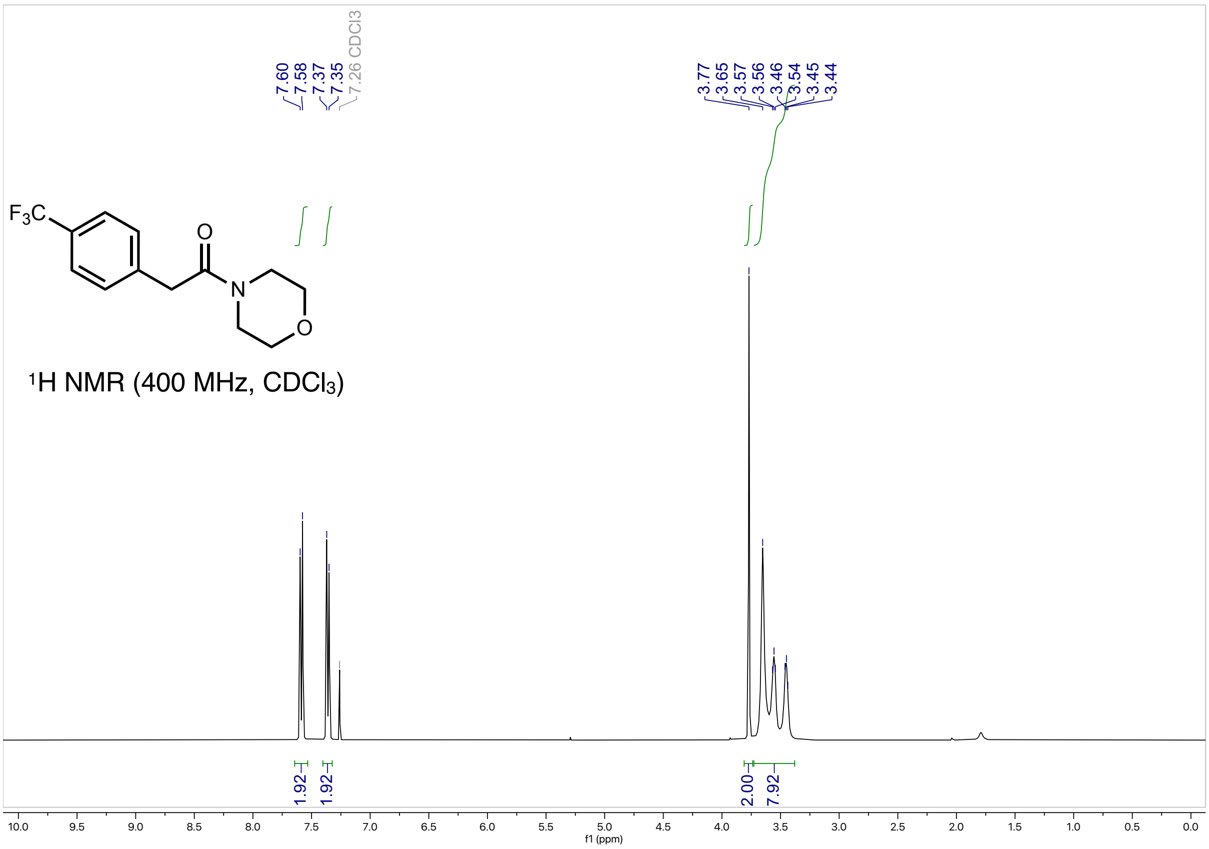
7.60
7.58
7.37
7.35
7.26 CDCl3
3.77
3.65
3.57
3.56
3.46
3.54
3.45
3.44
F_3C
O
N
O
^1H NMR (400 MHz, $CDCl_3$)
1.92
1.92
2.00
7.92
f1 (ppm)

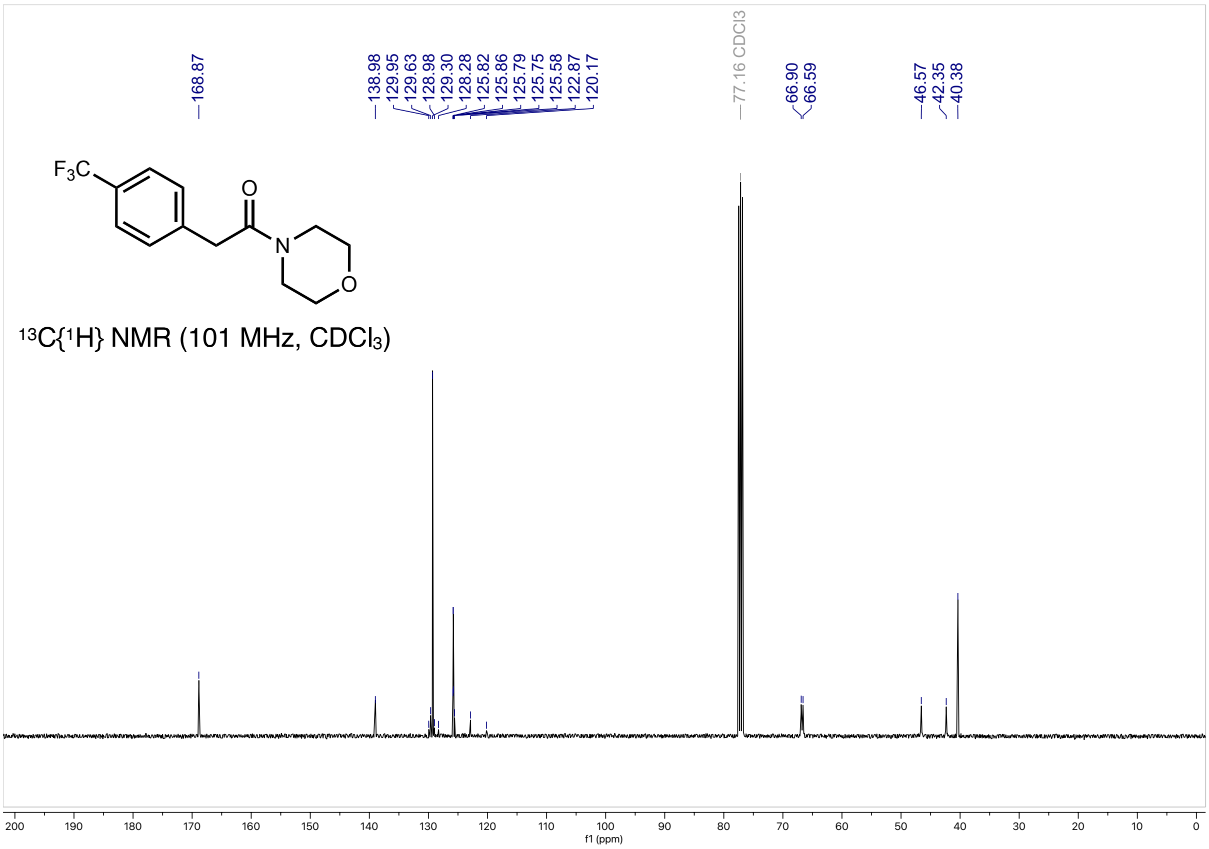
168.87
138.98
129.95
129.63
128.98
129.30
128.28
125.82
125.86
125.79
125.75
125.58
122.87
120.17
77.16 CDCl3
66.90
66.59
46.57
42.35
40.38
F_3C
O
N
O
$^{13}C\{^1H\}$ NMR (101 MHz, $CDCl_3$)
f1 (ppm)

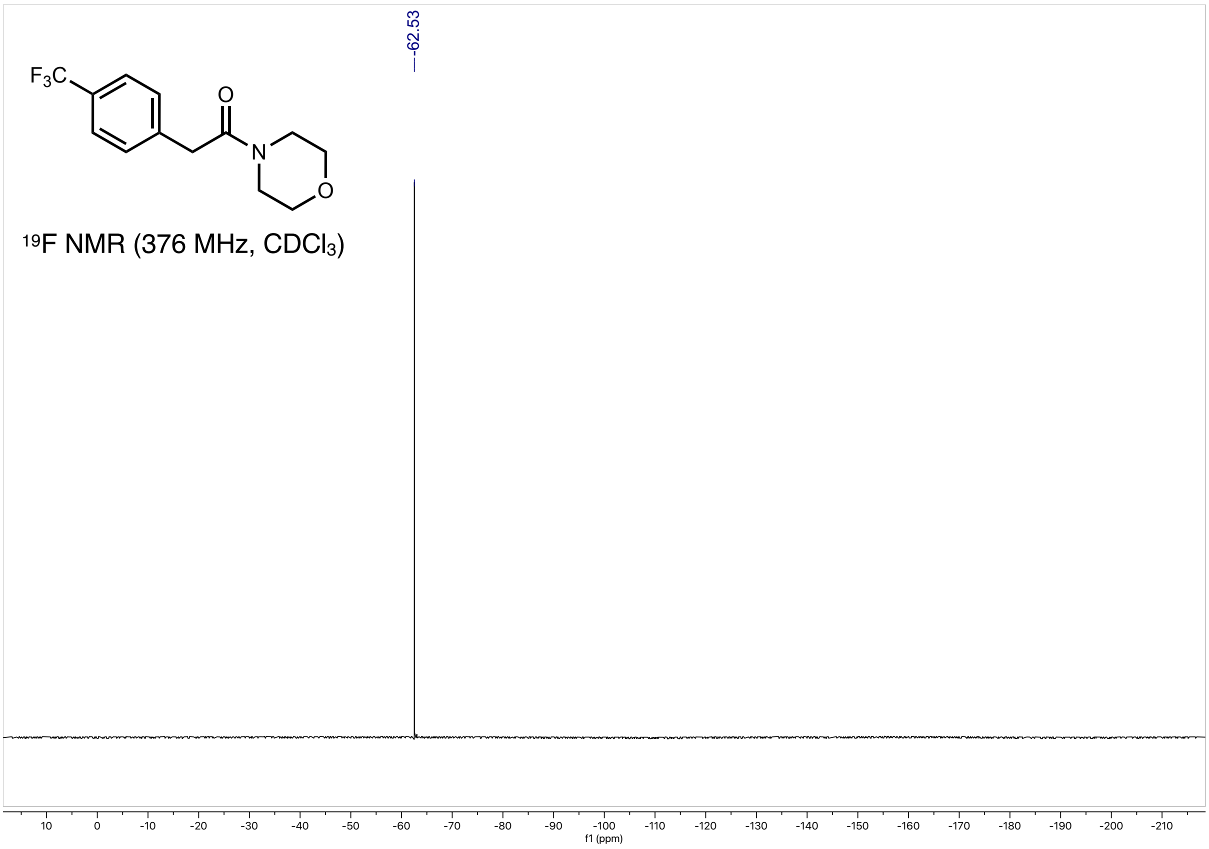

-62.53
F3C
O
N
O
19F NMR (376 MHz, CDCl3)
10
0
-10
-20
-30
-40
-50
-60
-70
-80
-90
-100
-110
-120
-130
-140
-150
-160
-170
-180
-190
-200
-210
f1 (ppm)

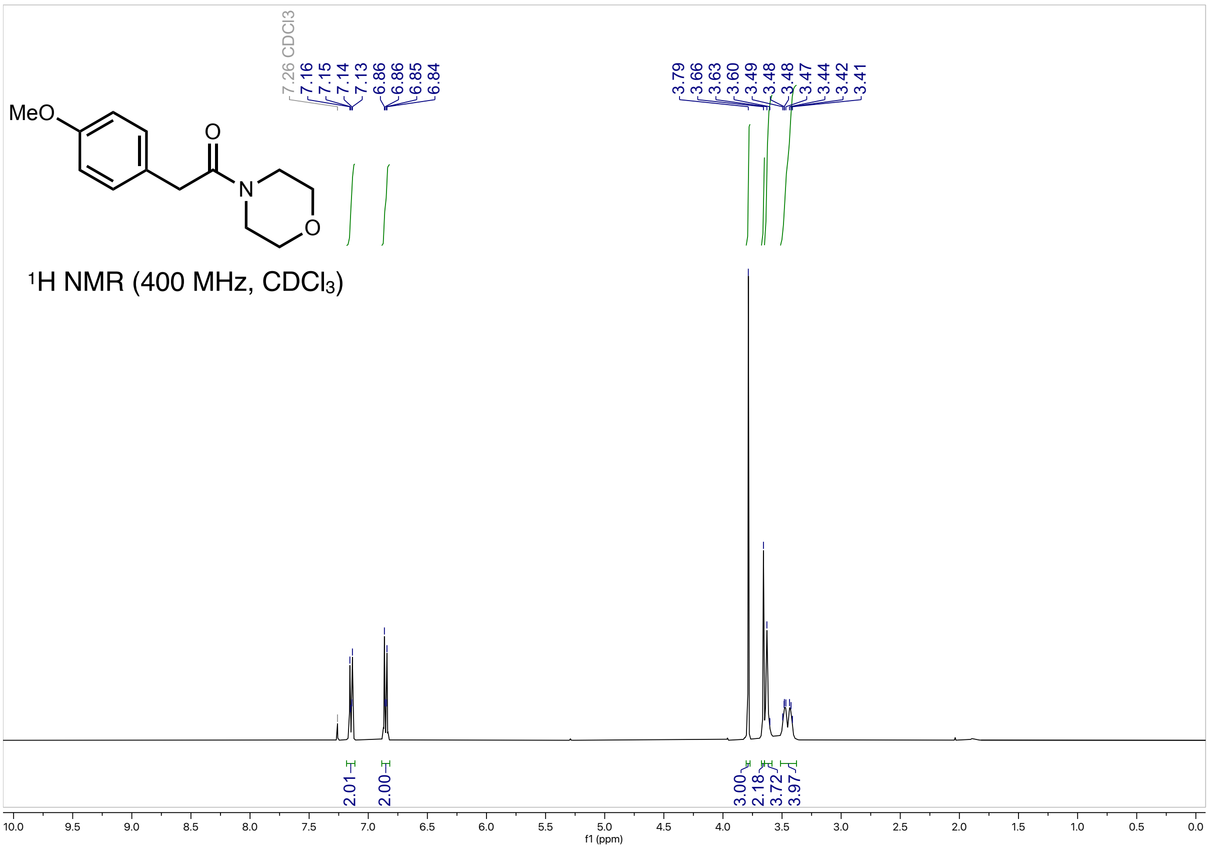
7.26 CDCl3
7.16
7.15
7.14
7.13
6.86
6.86
6.85
6.84
3.79
3.66
3.63
3.60
3.49
3.48
3.48
3.47
3.44
3.42
3.41
MeO
O
N
O
1H NMR (400 MHz, CDCl3)
2.01
2.00
3.00
2.18
3.72
3.97
10.0
9.5
9.0
8.5
8.0
7.5
7.0
6.5
6.0
5.5
5.0
4.5
4.0
3.5
3.0
2.5
2.0
1.5
1.0
0.5
0.0
f1 (ppm)

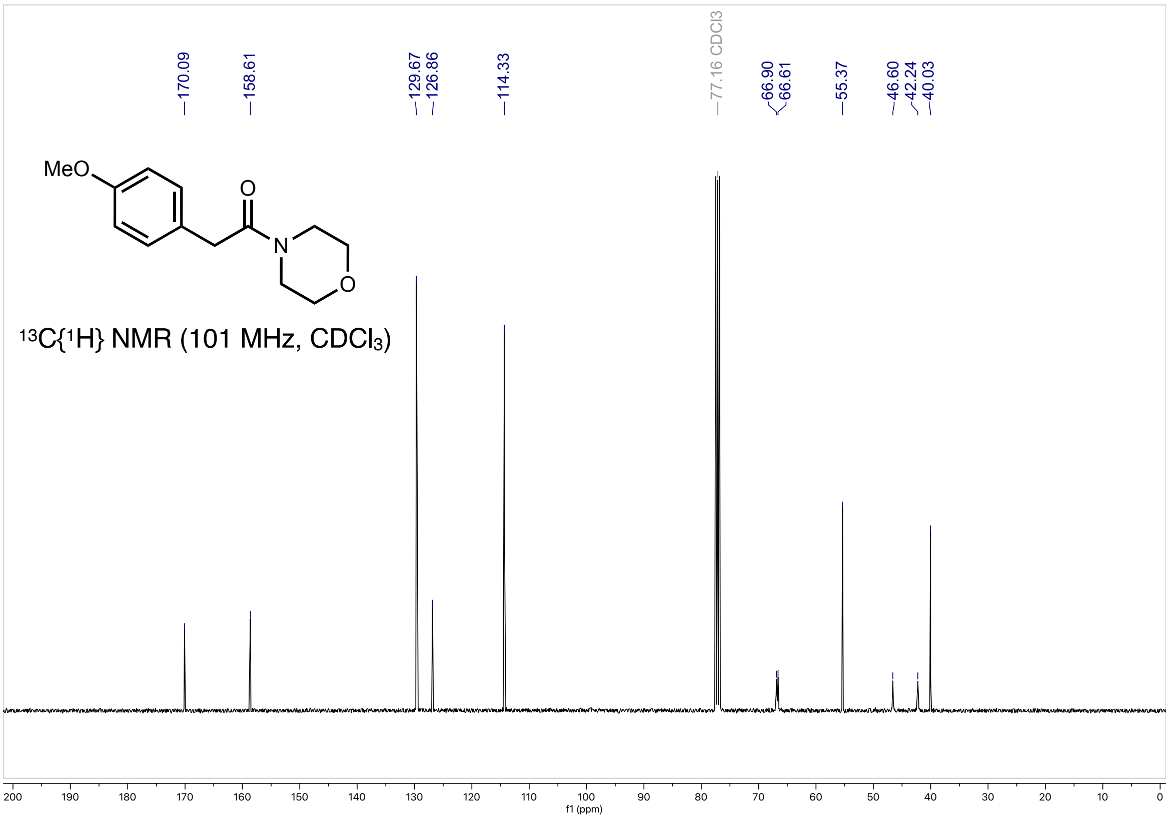
170.09
158.61
129.67
126.86
114.33
77.16 CDCl3
66.90
66.61
55.37
46.60
42.24
40.03
MeO
O
N
O
13C{1H} NMR (101 MHz, CDCl3)
200
190
180
170
160
150
140
130
120
110
100
90
80
70
60
50
40
30
20
10
0
f1 (ppm)

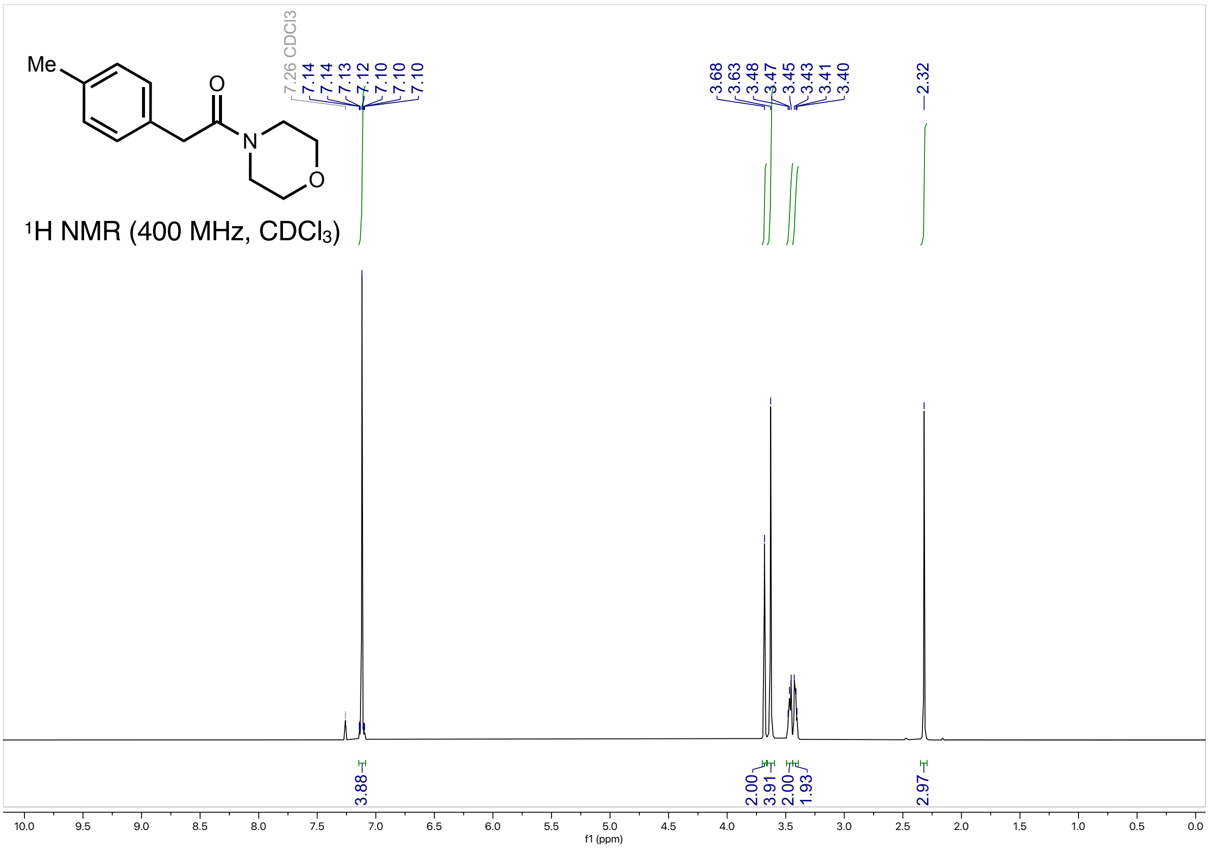
Me
O
N
O
¹H NMR (400 MHz, CDCl₃)
7.26 CDCl3
7.14
7.14
7.13
7.12
7.10
7.10
7.10
3.68
3.63
3.48
3.47
3.45
3.43
3.41
3.40
2.32
3.88
2.00
3.91
2.00
1.93
2.97
f1 (ppm)

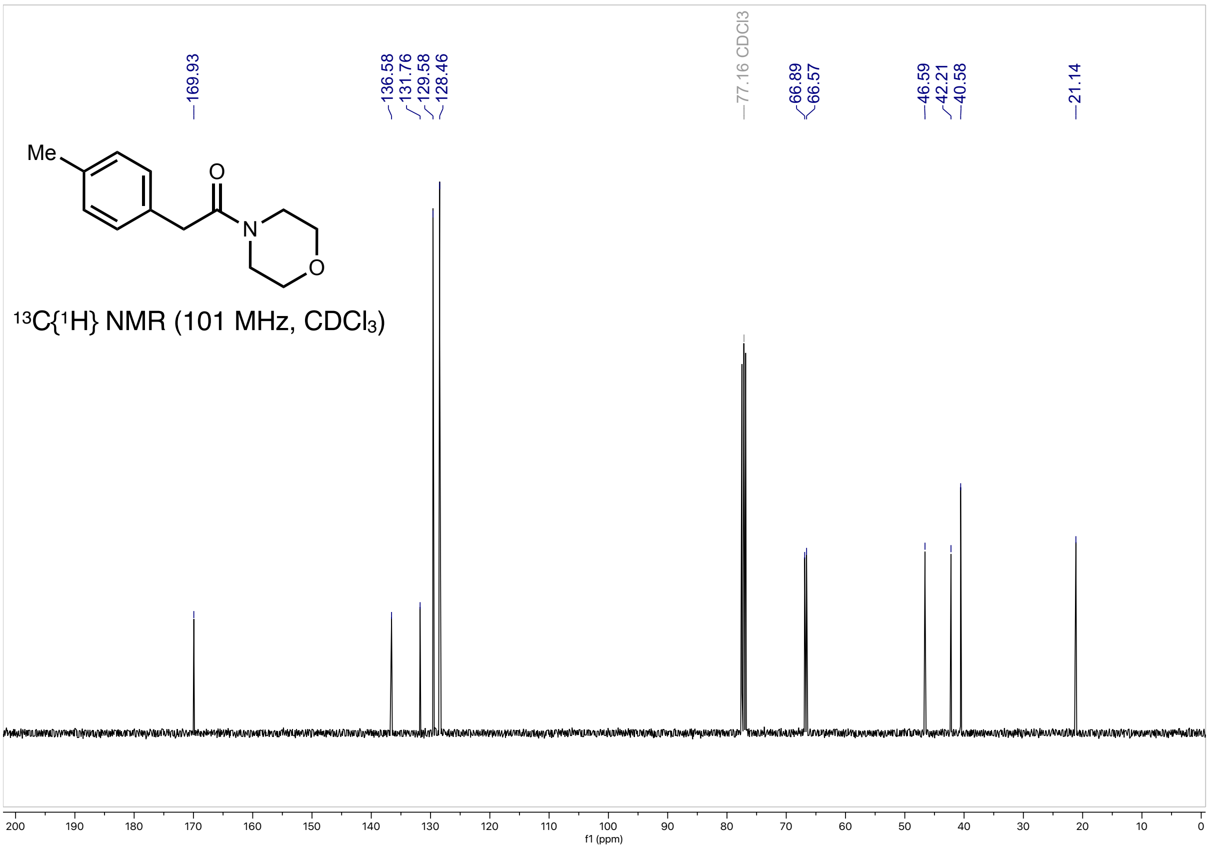
169.93
136.58
131.76
129.58
128.46
77.16 CDCl3
66.89
66.57
46.59
42.21
40.58
21.14
Me
O
N
O
¹³C{¹H} NMR (101 MHz, CDCl₃)
f1 (ppm)

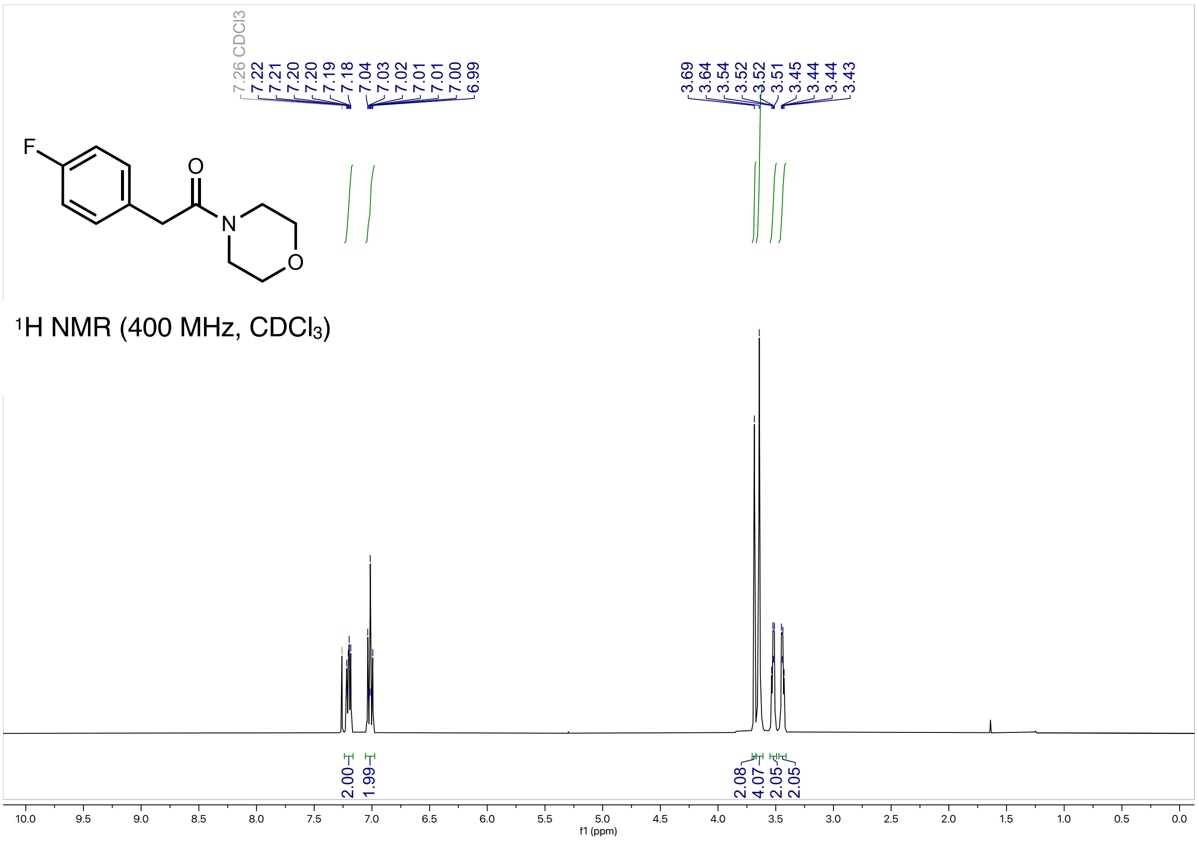
7.26 CDCl3
7.22
7.21
7.20
7.20
7.19
7.18
7.04
7.03
7.02
7.01
7.00
6.99
3.69
3.64
3.54
3.52
3.52
3.51
3.45
3.44
3.44
3.43
F
O
N
O
¹H NMR (400 MHz, CDCl₃)
2.00
1.99
2.08
4.07
2.05
2.05
f1 (ppm)

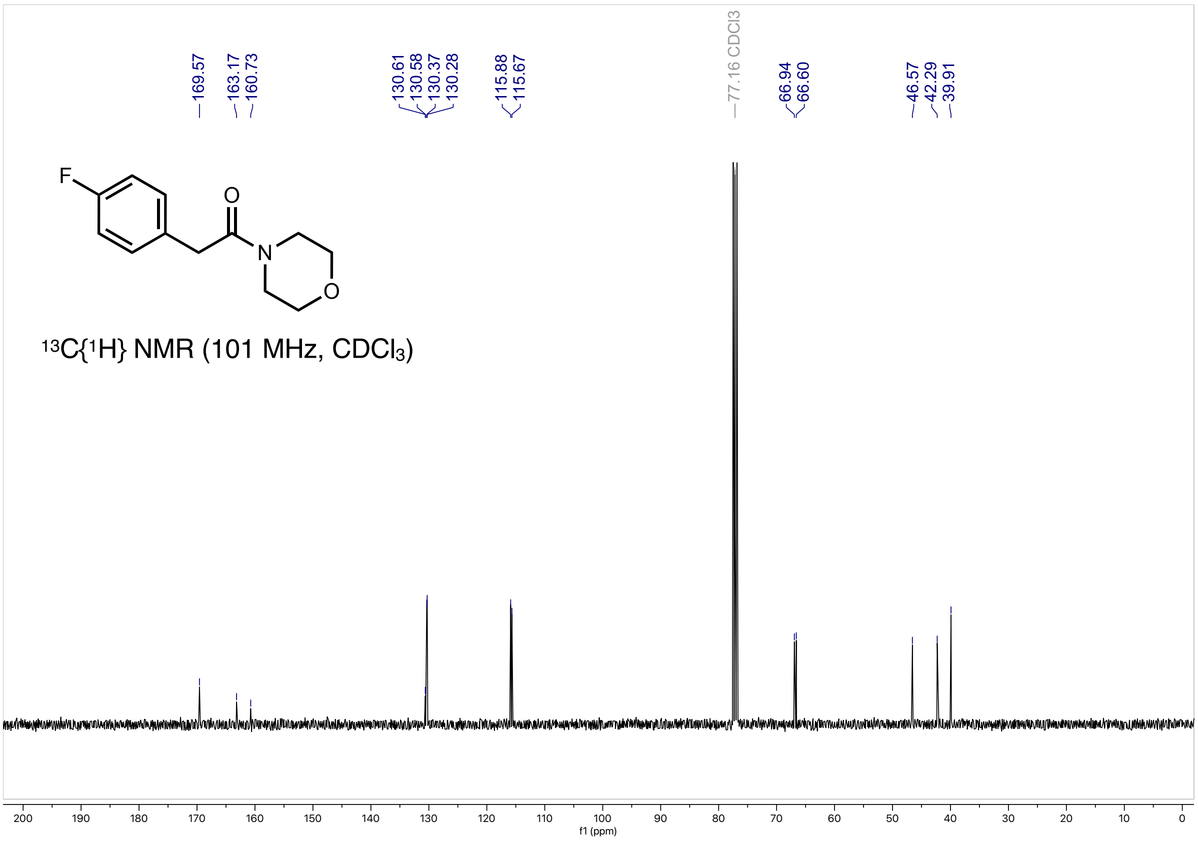
169.57
163.17
160.73
130.61
130.58
130.37
130.28
115.88
115.67
77.16 CDCl3
66.94
66.60
46.57
42.29
39.91
F
O
N
O
¹³C{¹H} NMR (101 MHz, CDCl₃)
f1 (ppm)

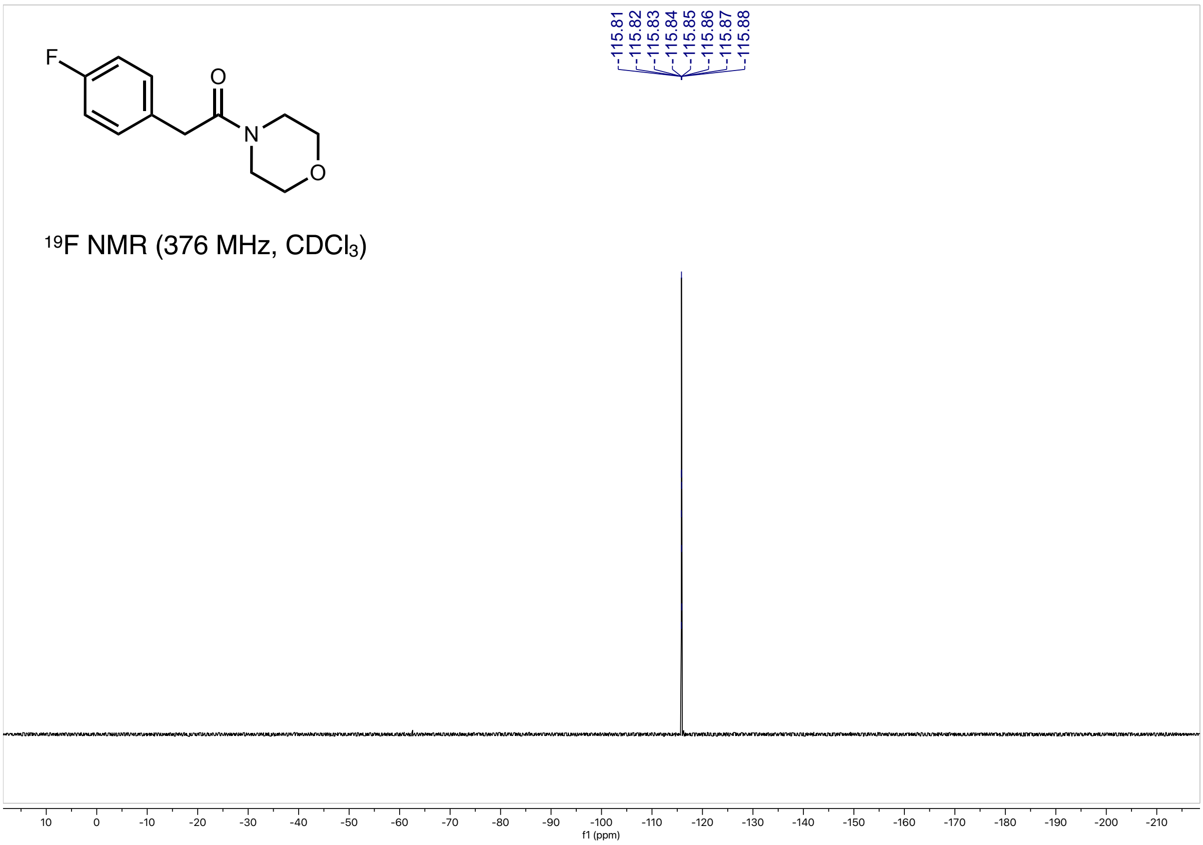

-115.81
-115.82
-115.83
-115.84
-115.85
-115.86
-115.87
-115.88
F
O
N
O
^{19}F NMR (376 MHz, CDCl$_3$)
10
0
-10
-20
-30
-40
-50
-60
-70
-80
-90
-100
-110
-120
-130
-140
-150
-160
-170
-180
-190
-200
-210
f1 (ppm)

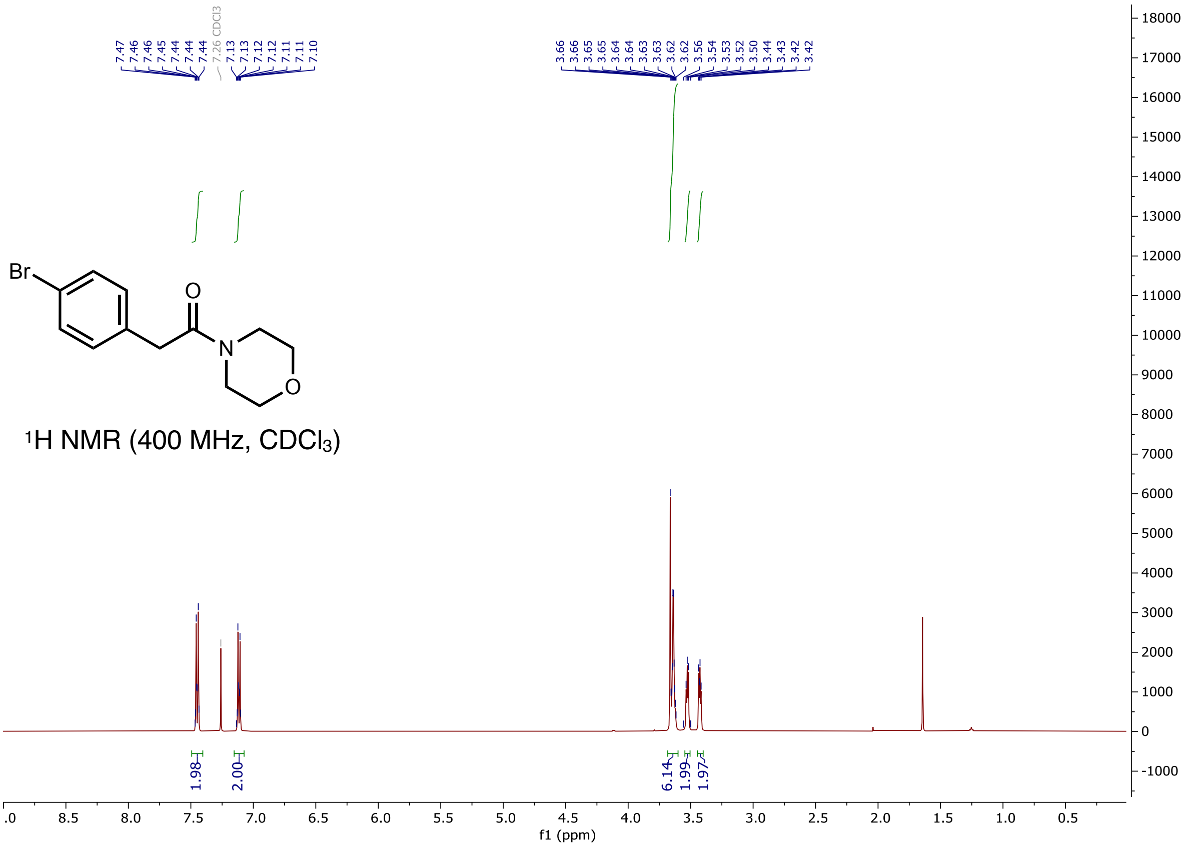
7.47
7.46
7.46
7.45
7.44
7.44
7.44
7.26 CDCl3
7.13
7.13
7.12
7.12
7.11
7.11
7.10
3.66
3.66
3.65
3.65
3.64
3.64
3.63
3.63
3.62
3.62
3.56
3.54
3.53
3.52
3.50
3.44
3.43
3.42
3.42
Br
O
N
O
¹H NMR (400 MHz, CDCl₃)
1.98
2.00
6.14
1.99
1.97
f1 (ppm)

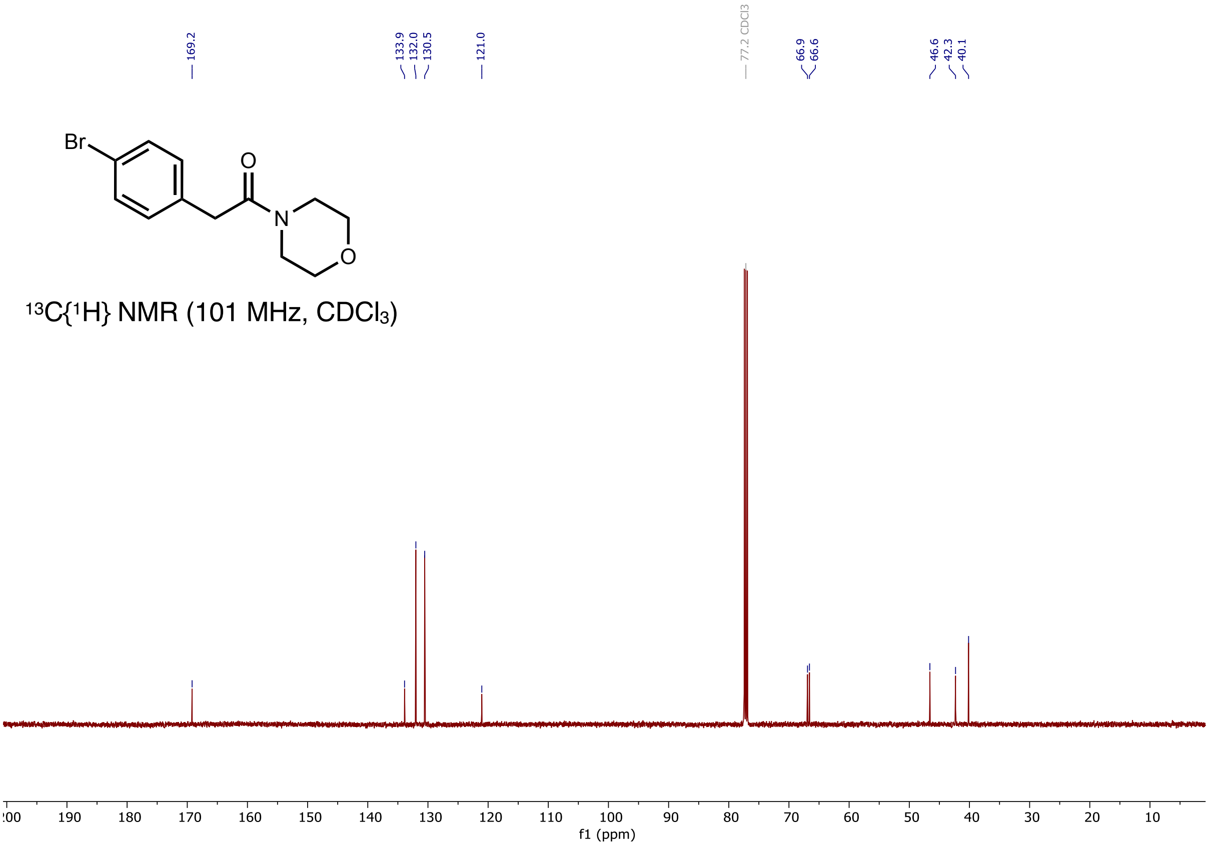
169.2
133.9
132.0
130.5
121.0
77.2 CDCl3
66.9
66.6
46.6
42.3
40.1
Br
O
N
O
¹³C{¹H} NMR (101 MHz, CDCl₃)
f1 (ppm)

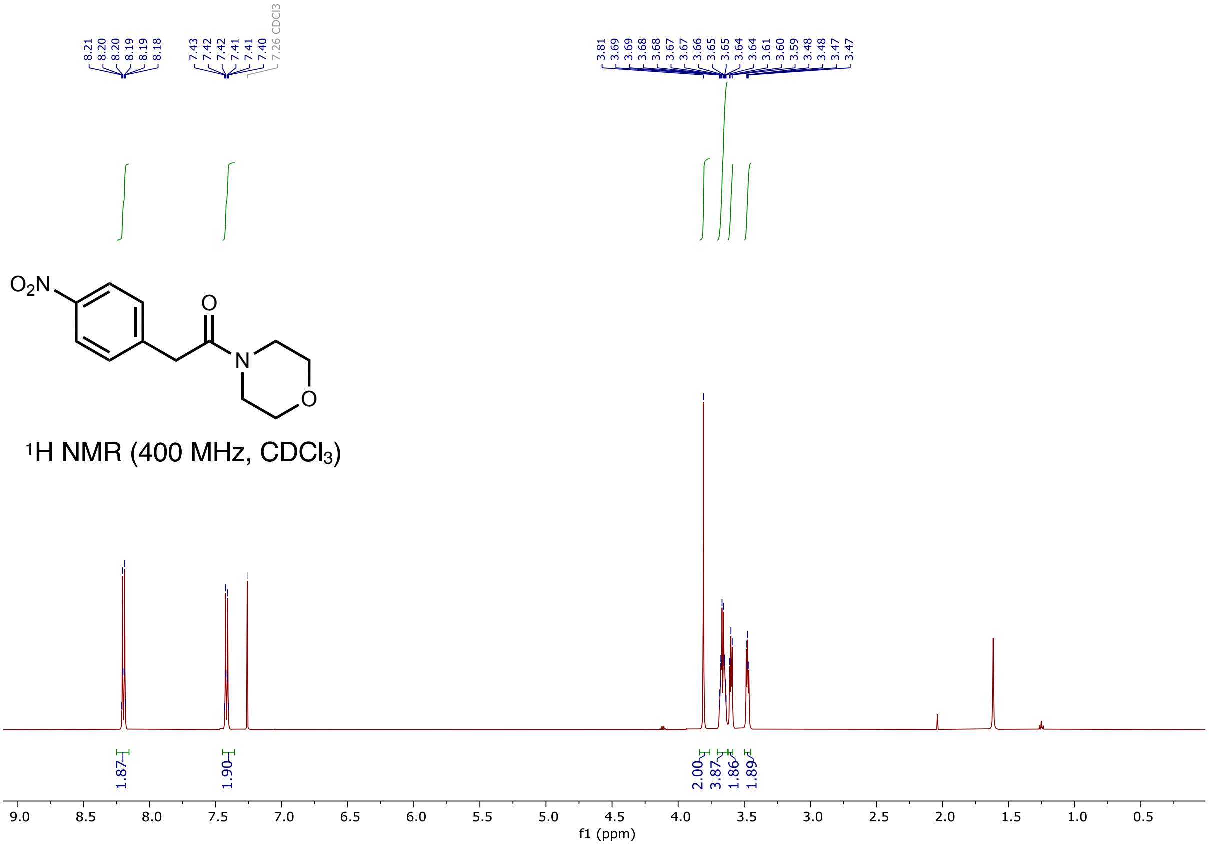

^1H NMR (400 MHz, $CDCl_3$)

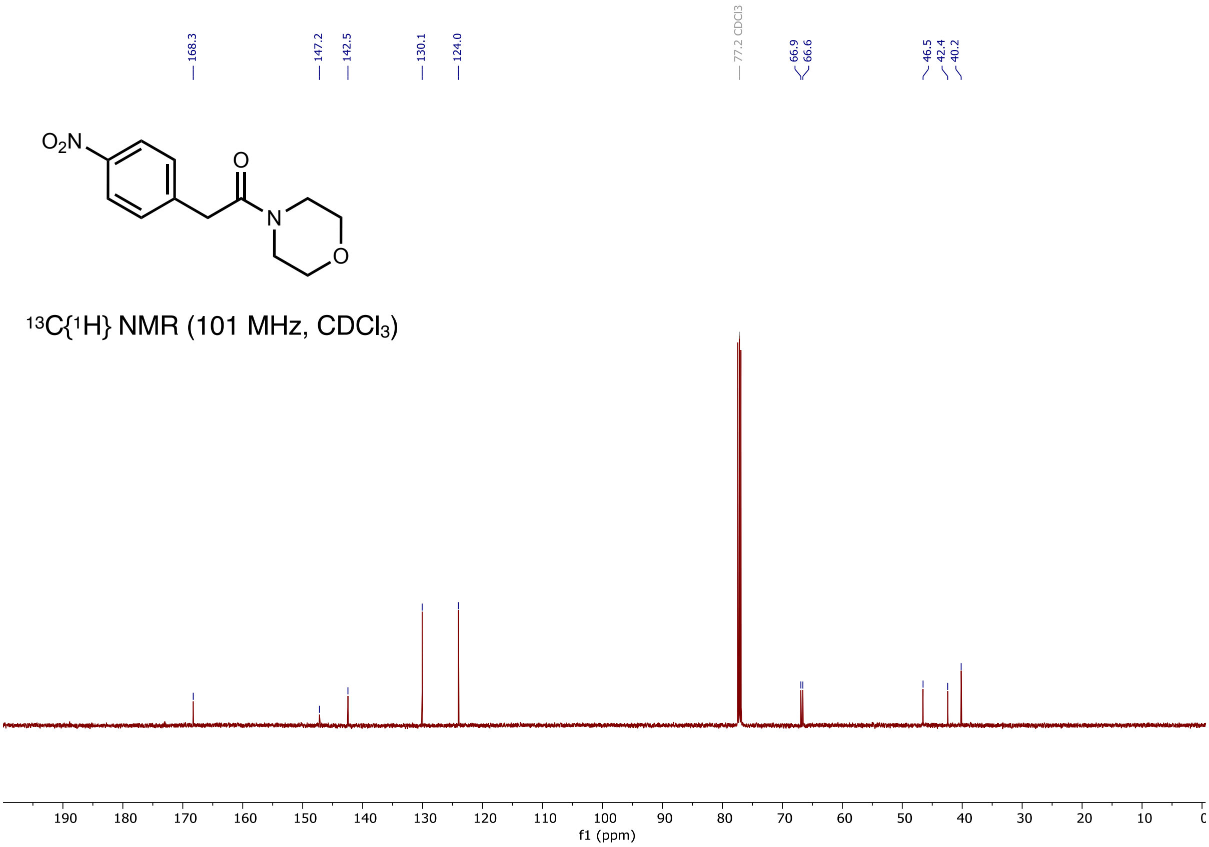

$^{13}C\{^1H\}$ NMR (101 MHz, $CDCl_3$)

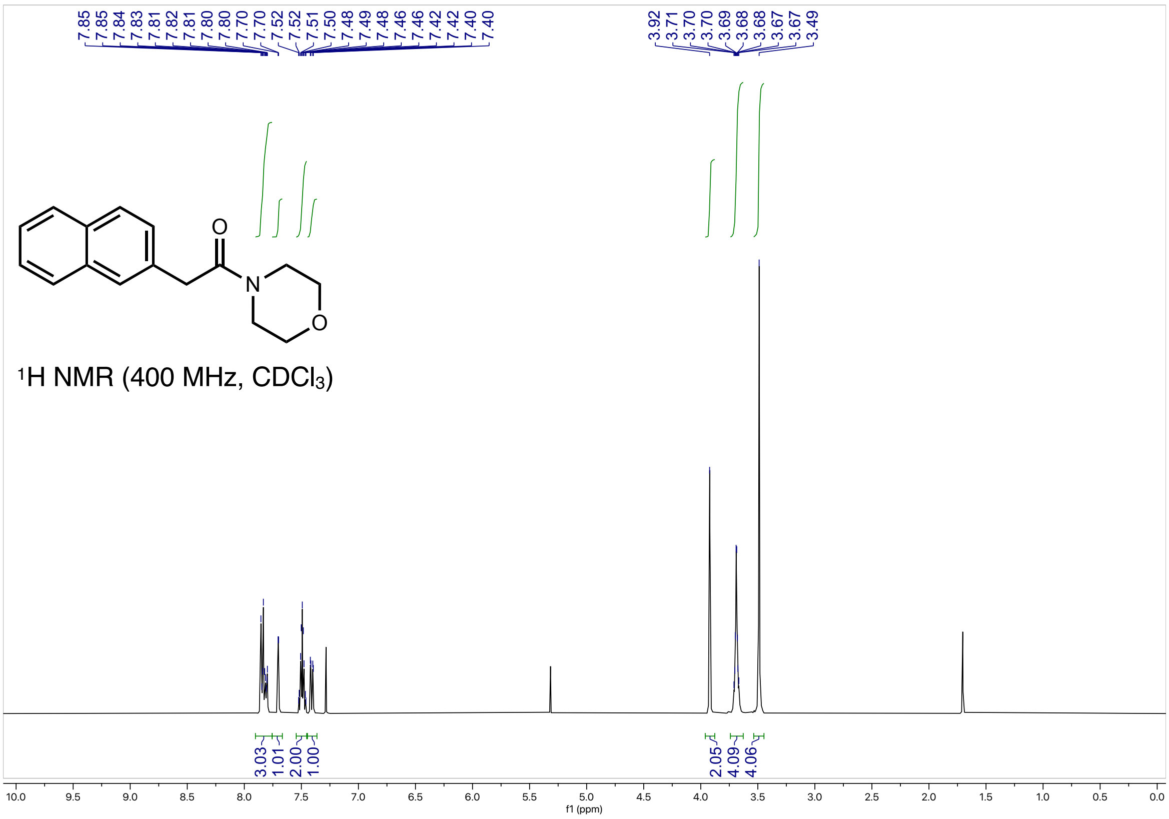
7.85
7.85
7.84
7.83
7.81
7.82
7.81
7.80
7.80
7.70
7.70
7.52
7.52
7.51
7.50
7.48
7.49
7.48
7.48
7.46
7.46
7.42
7.42
7.40
7.40
3.92
3.71
3.70
3.70
3.69
3.68
3.68
3.67
3.49
O
N
O
1H NMR (400 MHz, CDCl3)
3.03
1.01
2.00
1.00
2.05
4.09
4.06
f1 (ppm)

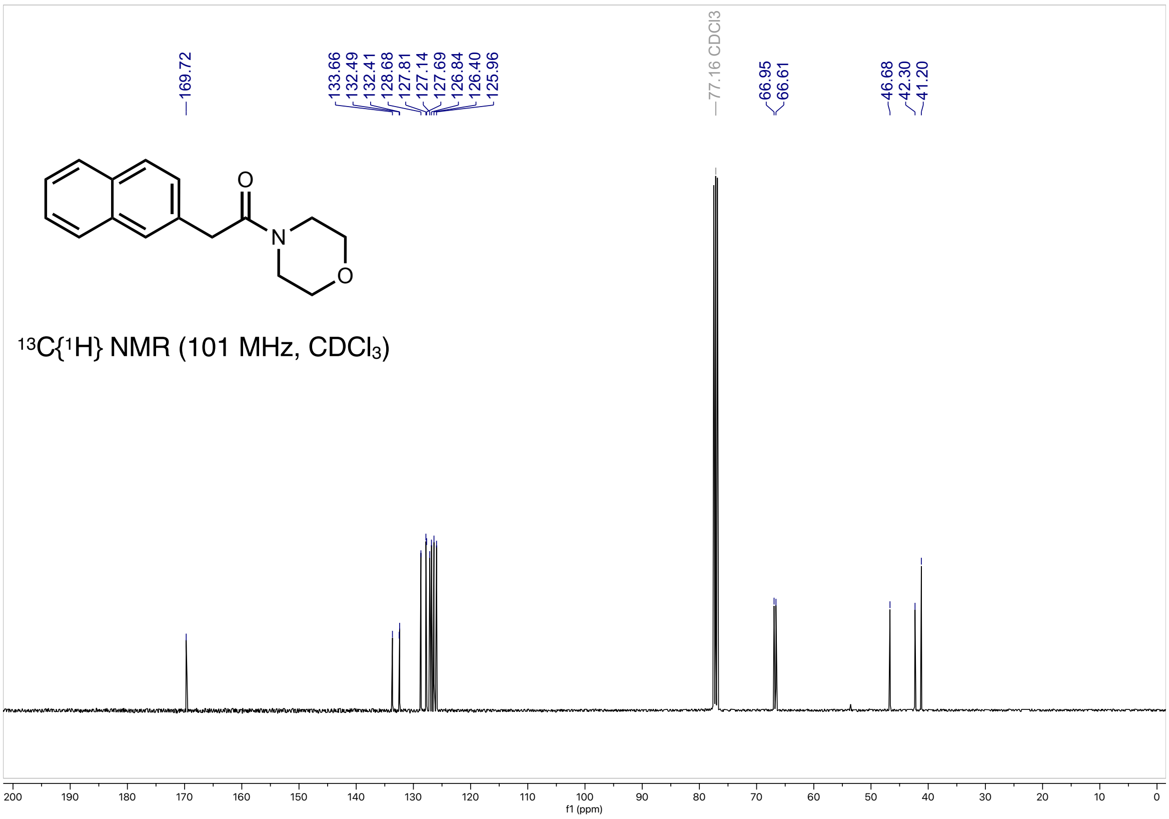
169.72
133.66
132.49
132.41
128.68
127.81
127.14
127.69
126.84
126.40
125.96
77.16 CDCl3
66.95
66.61
46.68
42.30
41.20
O
N
O
13C{1H} NMR (101 MHz, CDCl3)
f1 (ppm)

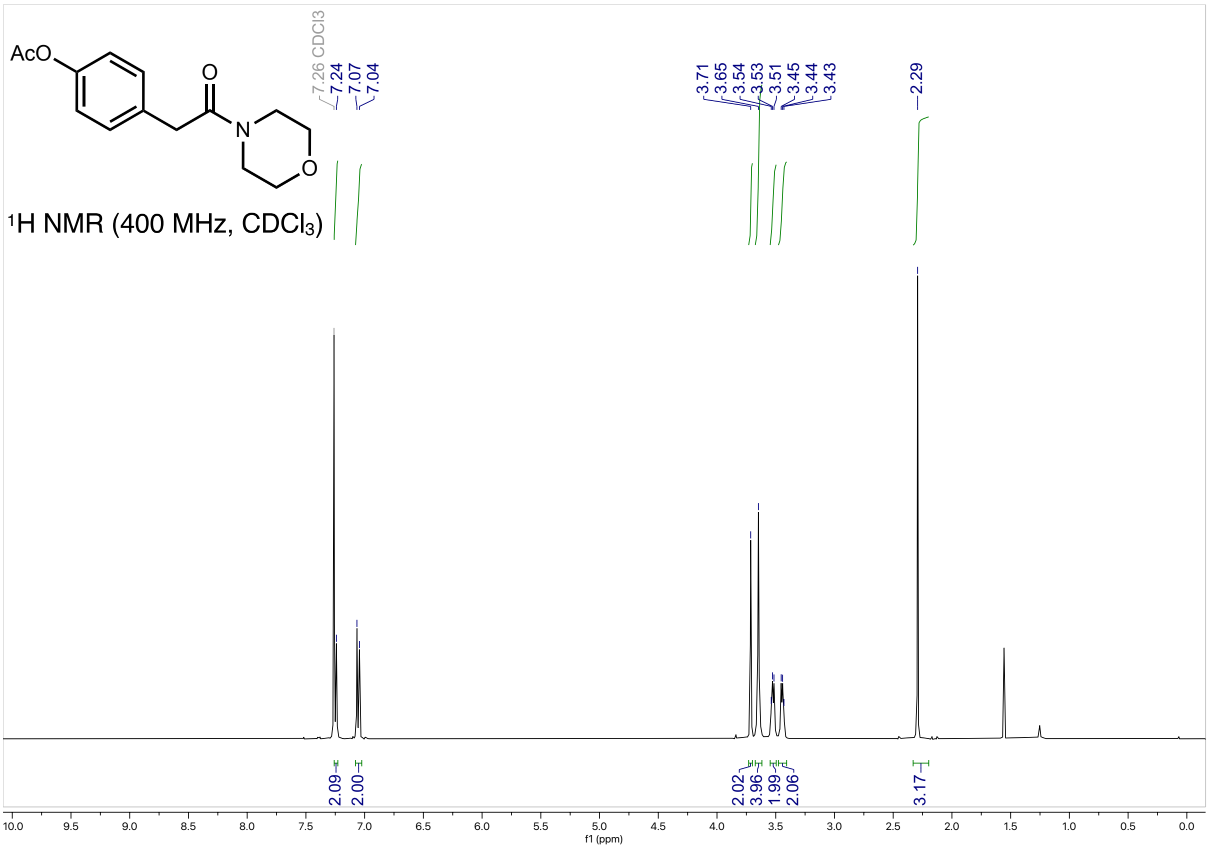
AcO
O
N
O
1H NMR (400 MHz, CDCl3)
7.26 CDCl3
7.24
7.07
7.04
3.71
3.65
3.54
3.53
3.51
3.45
3.44
3.43
2.29
2.09
2.00
2.02
3.96
1.99
2.06
3.17
f1 (ppm)

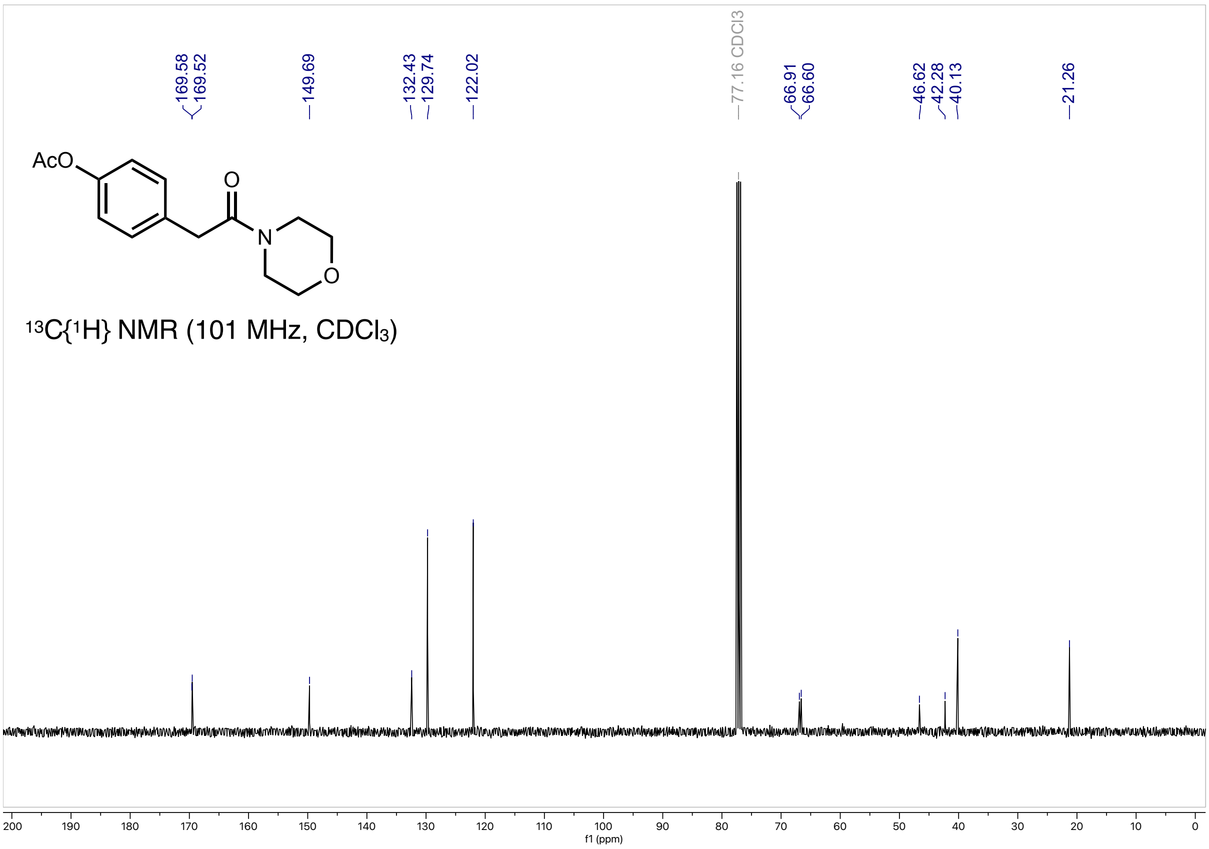
169.58
169.52
149.69
132.43
129.74
122.02
77.16 CDCl3
66.91
66.60
46.62
42.28
40.13
21.26
AcO
O
N
O
13C{1H} NMR (101 MHz, CDCl3)
f1 (ppm)

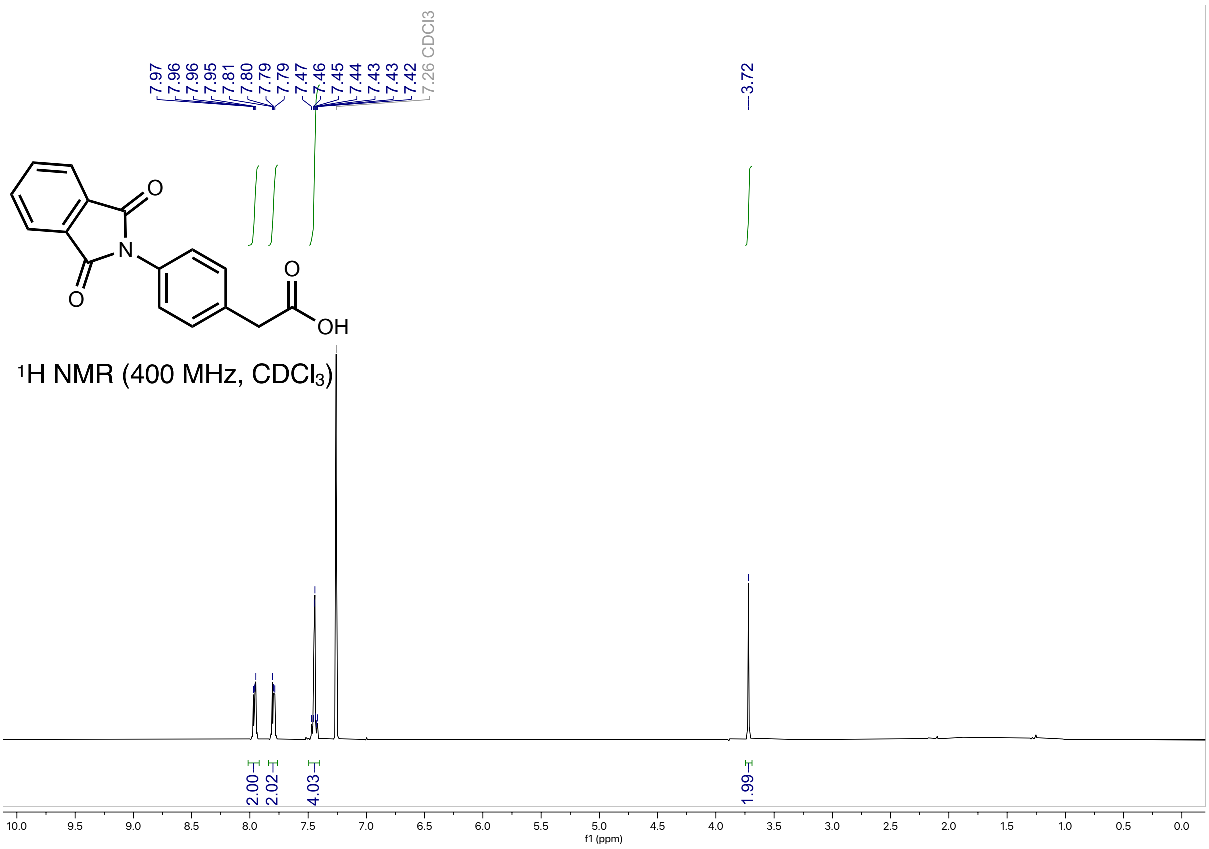
7.97
7.96
7.96
7.95
7.81
7.80
7.79
7.79
7.47
7.46
7.45
7.44
7.43
7.43
7.42
7.26 CDCl3
3.72
O
N
O
O
OH
¹H NMR (400 MHz, CDCl₃)
2.00
2.02
4.03
1.99
10.0
9.5
9.0
8.5
8.0
7.5
7.0
6.5
6.0
5.5
5.0
4.5
4.0
3.5
3.0
2.5
2.0
1.5
1.0
0.5
0.0
f1 (ppm)

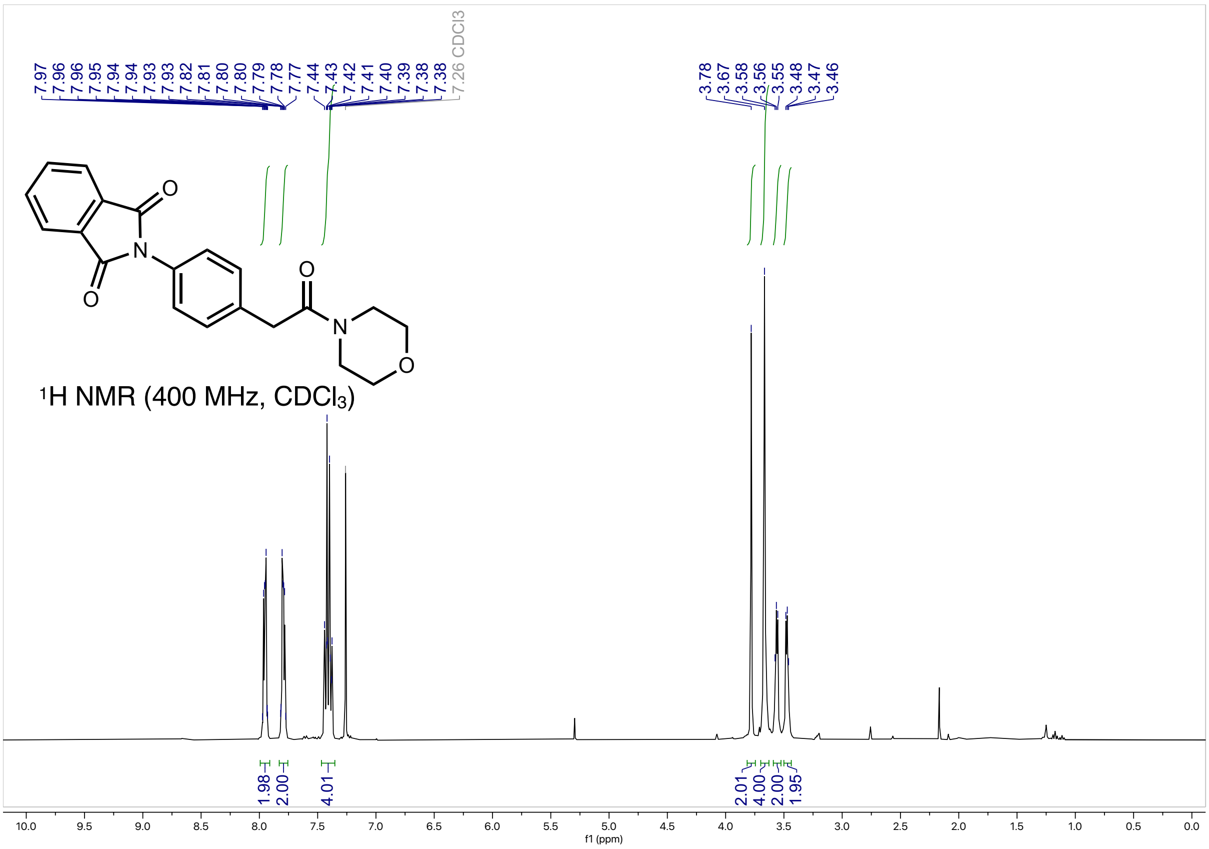
1H NMR (400 MHz, CDCl3)
7.97 7.96 7.95 7.94 7.94 7.93 7.93 7.82 7.81 7.80 7.80 7.79 7.78 7.77 7.44 7.43 7.42 7.41 7.40 7.39 7.38 7.38
7.26 CDCl3
3.78 3.67 3.58 3.56 3.55 3.48 3.47 3.46
1.98 2.00 4.01 2.01 4.00 2.00 1.95
f1 (ppm)

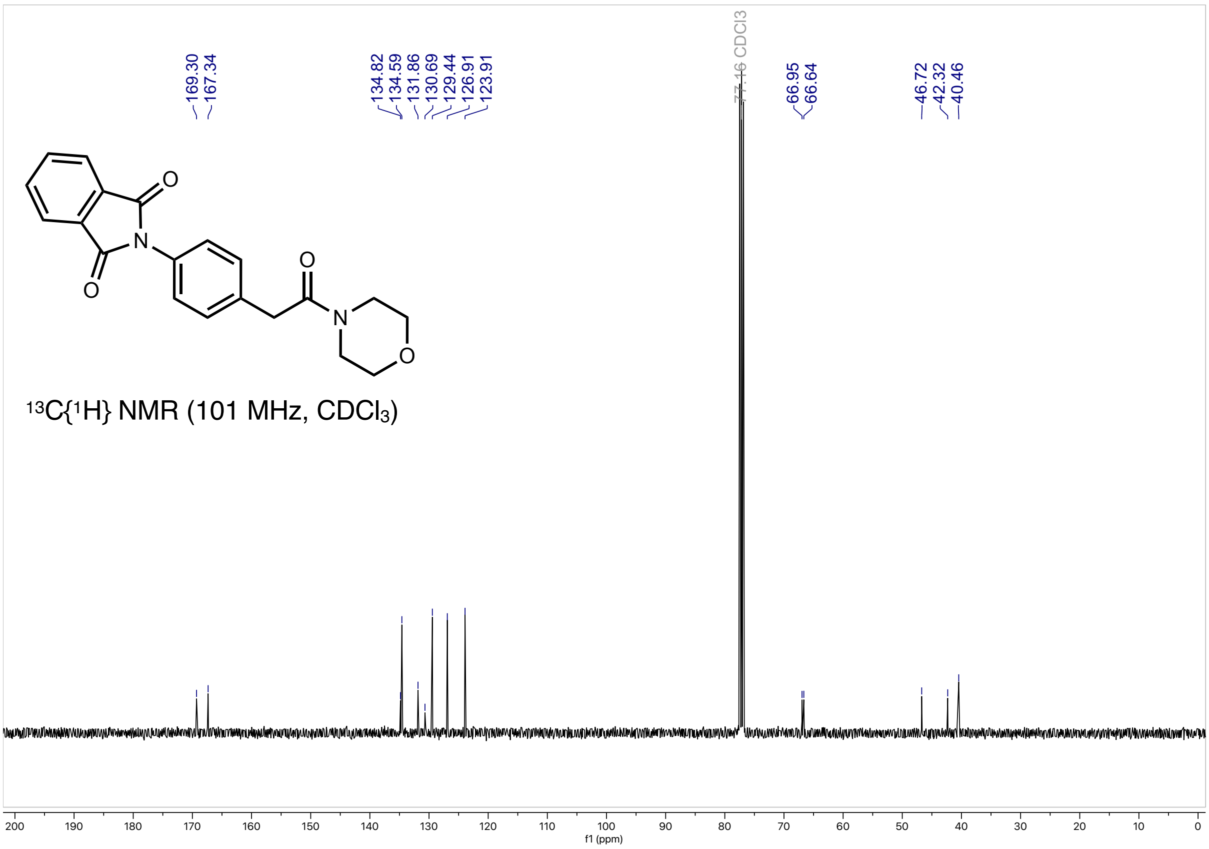
13C{1H} NMR (101 MHz, CDCl3)
169.30 167.34
134.82 134.59 131.86 130.69 129.44 126.91 123.91
77.16 CDCl3
66.95 66.64
46.72 42.32 40.46
f1 (ppm)

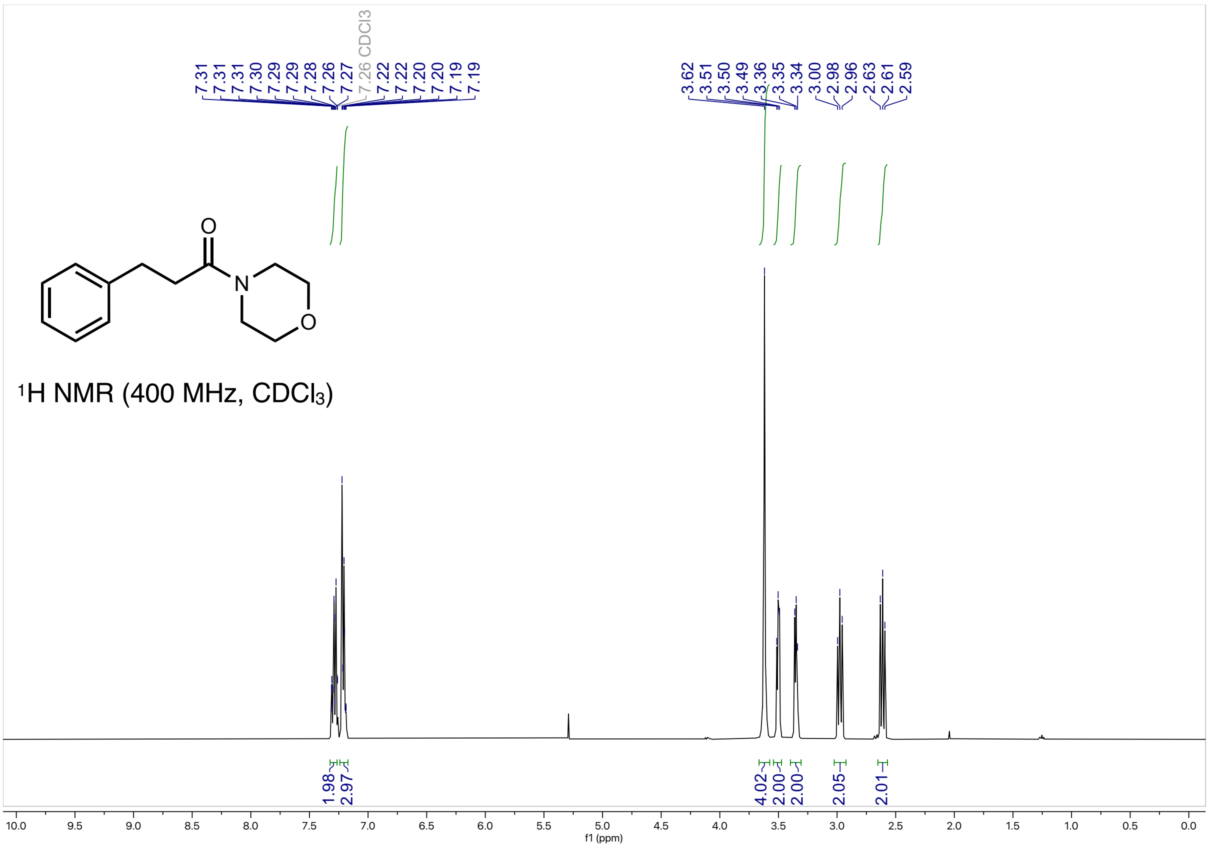
7.31
7.31
7.31
7.30
7.29
7.29
7.28
7.26
7.27
7.26 CDCl3
7.22
7.22
7.22
7.20
7.20
7.19
7.19
3.62
3.51
3.50
3.49
3.36
3.35
3.34
3.00
2.98
2.96
2.63
2.61
2.59
O
N
O
¹H NMR (400 MHz, CDCl₃)
1.98
2.97
4.02
2.00
2.00
2.05
2.01
f1 (ppm)

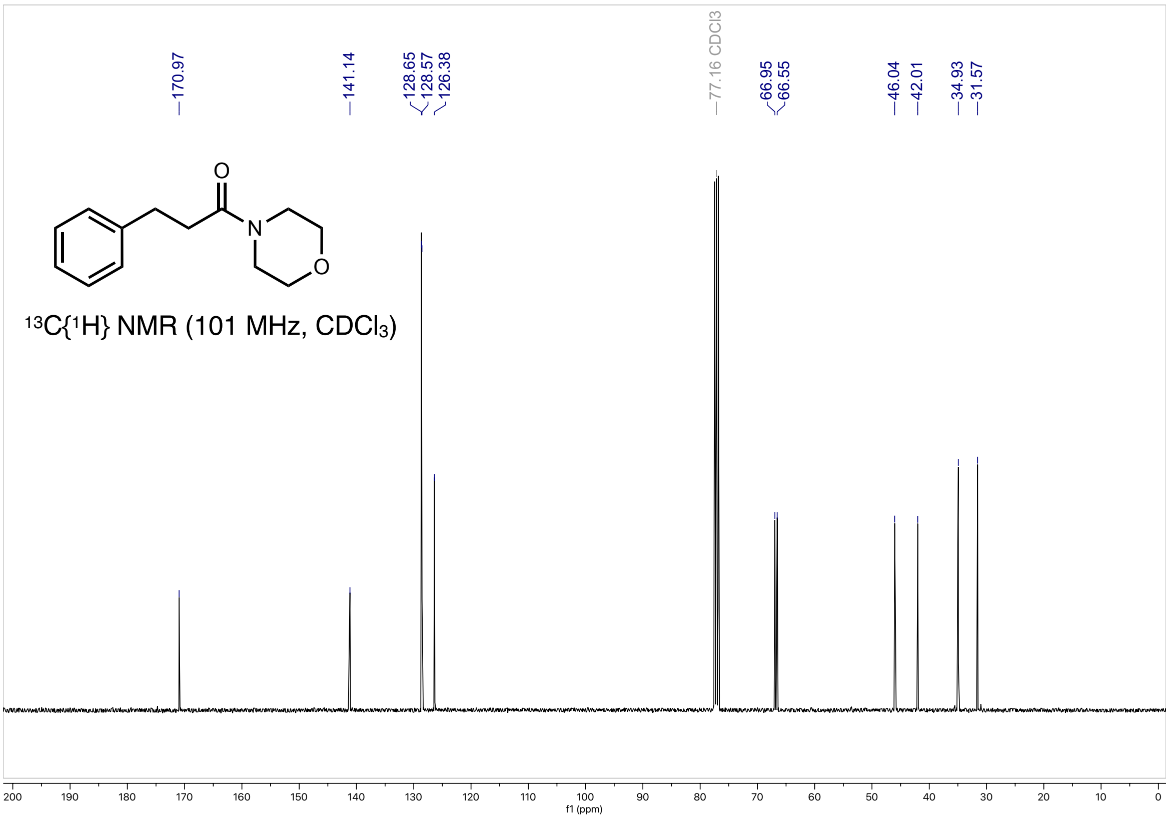
170.97
141.14
128.65
128.57
126.38
77.16 CDCl3
66.95
66.55
46.04
42.01
34.93
31.57
O
N
O
¹³C{¹H} NMR (101 MHz, CDCl₃)
f1 (ppm)

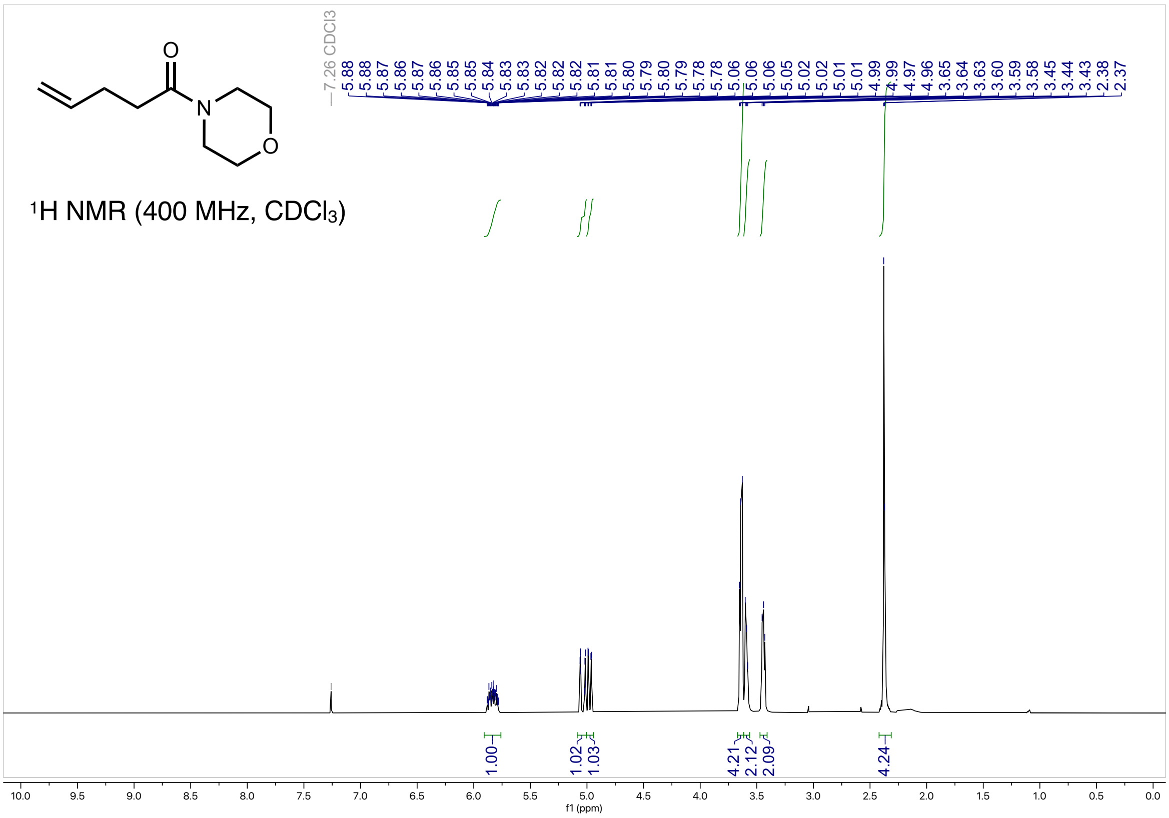

¹H NMR (400 MHz, CDCl₃)
7.26 CDCl3
5.88
5.88
5.87
5.87
5.86
5.86
5.85
5.85
5.84
5.83
5.83
5.82
5.82
5.82
5.81
5.81
5.80
5.79
5.80
5.79
5.78
5.78
5.06
5.06
5.06
5.05
5.02
5.02
5.01
5.01
4.99
4.99
4.97
4.96
3.65
3.64
3.63
3.60
3.59
3.58
3.45
3.44
3.43
2.38
2.37
1.00
1.02
1.03
4.21
2.12
2.09
4.24
f1 (ppm)

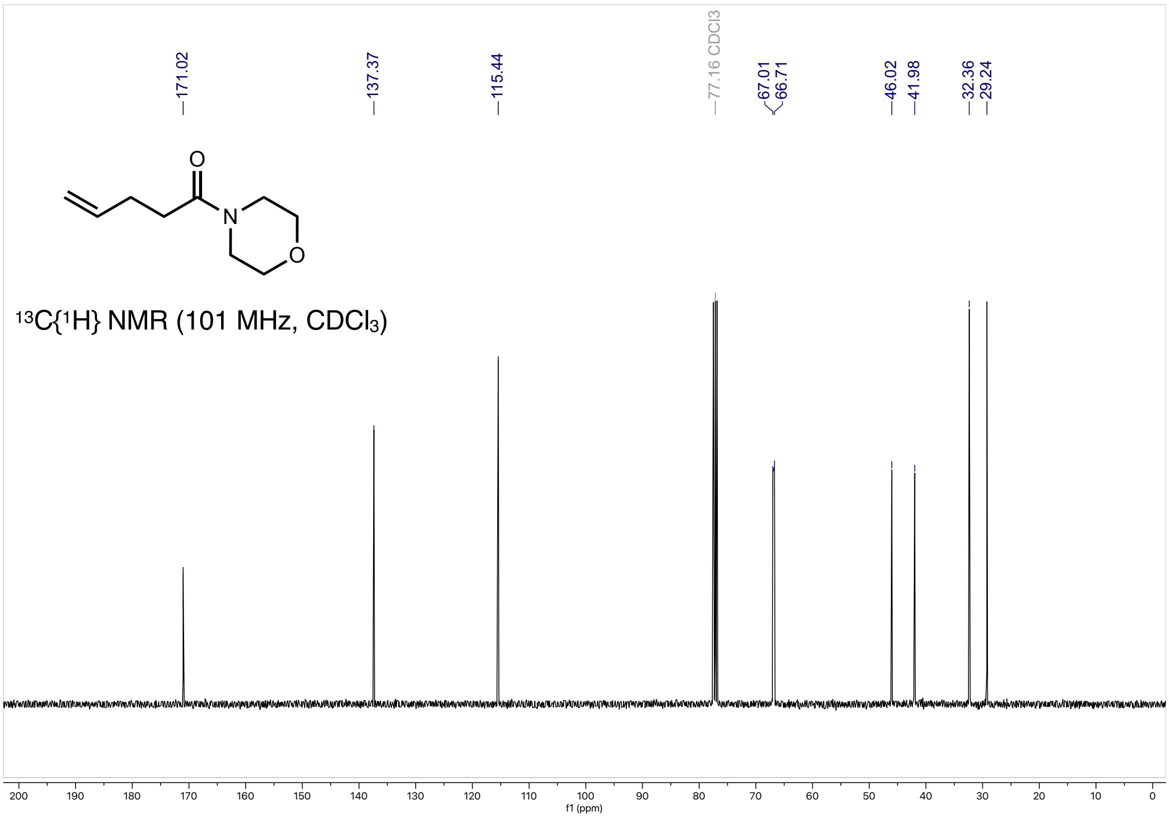

171.02
137.37
115.44
77.16 CDCl3
67.01
66.71
46.02
41.98
32.36
29.24
¹³C{¹H} NMR (101 MHz, CDCl₃)
f1 (ppm)

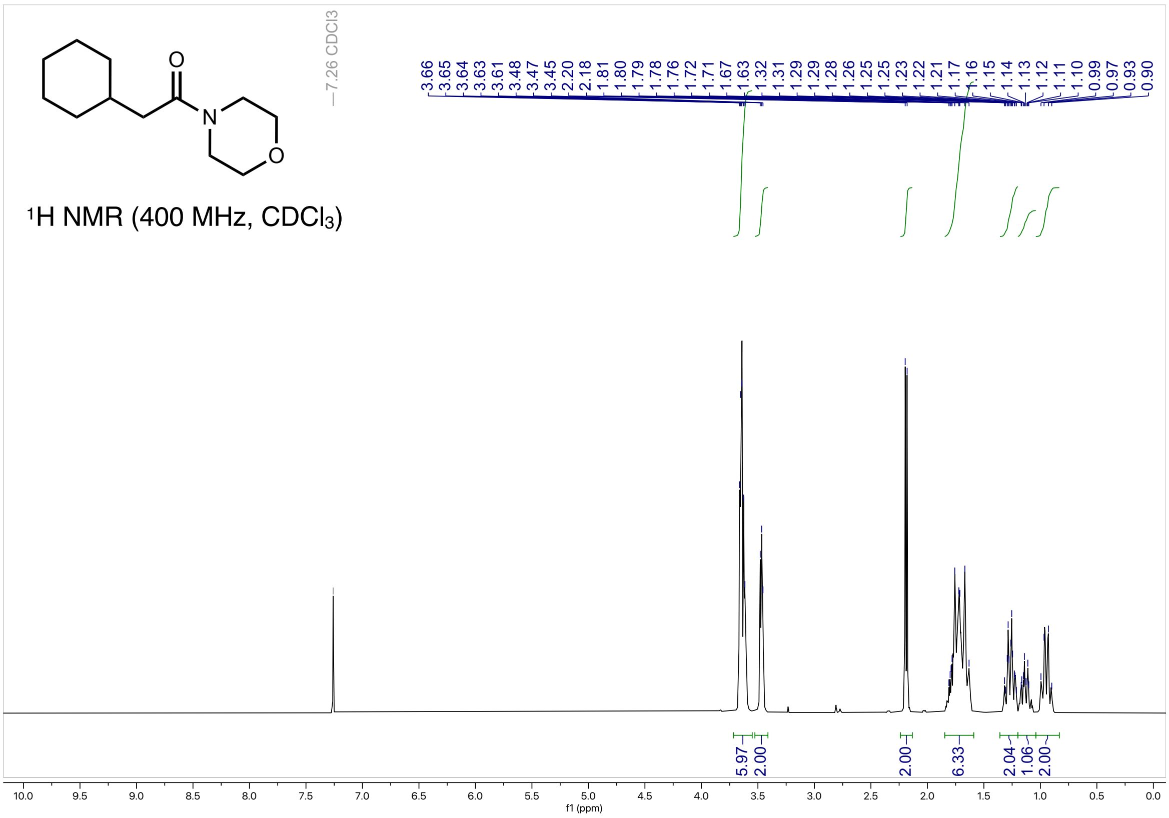

¹H NMR (400 MHz, CDCl₃)

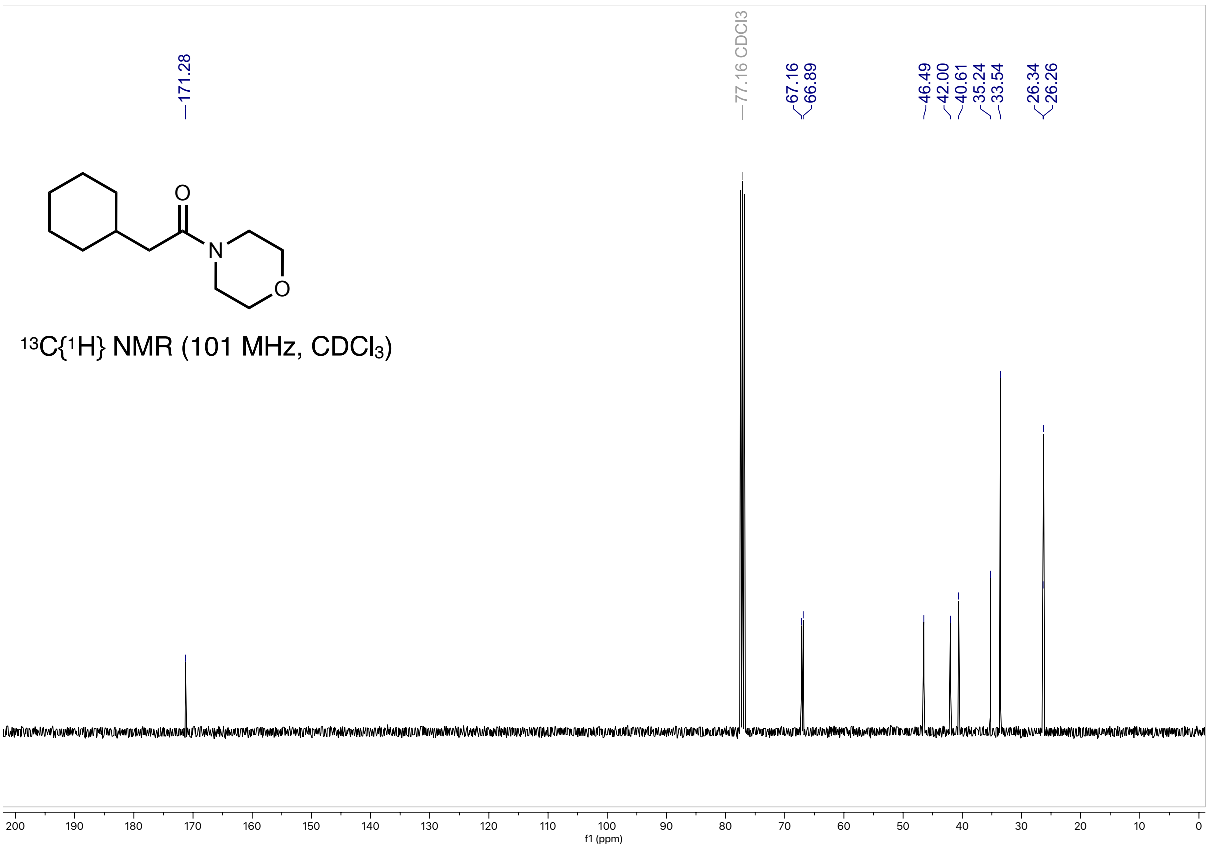

¹³C{¹H} NMR (101 MHz, CDCl₃)

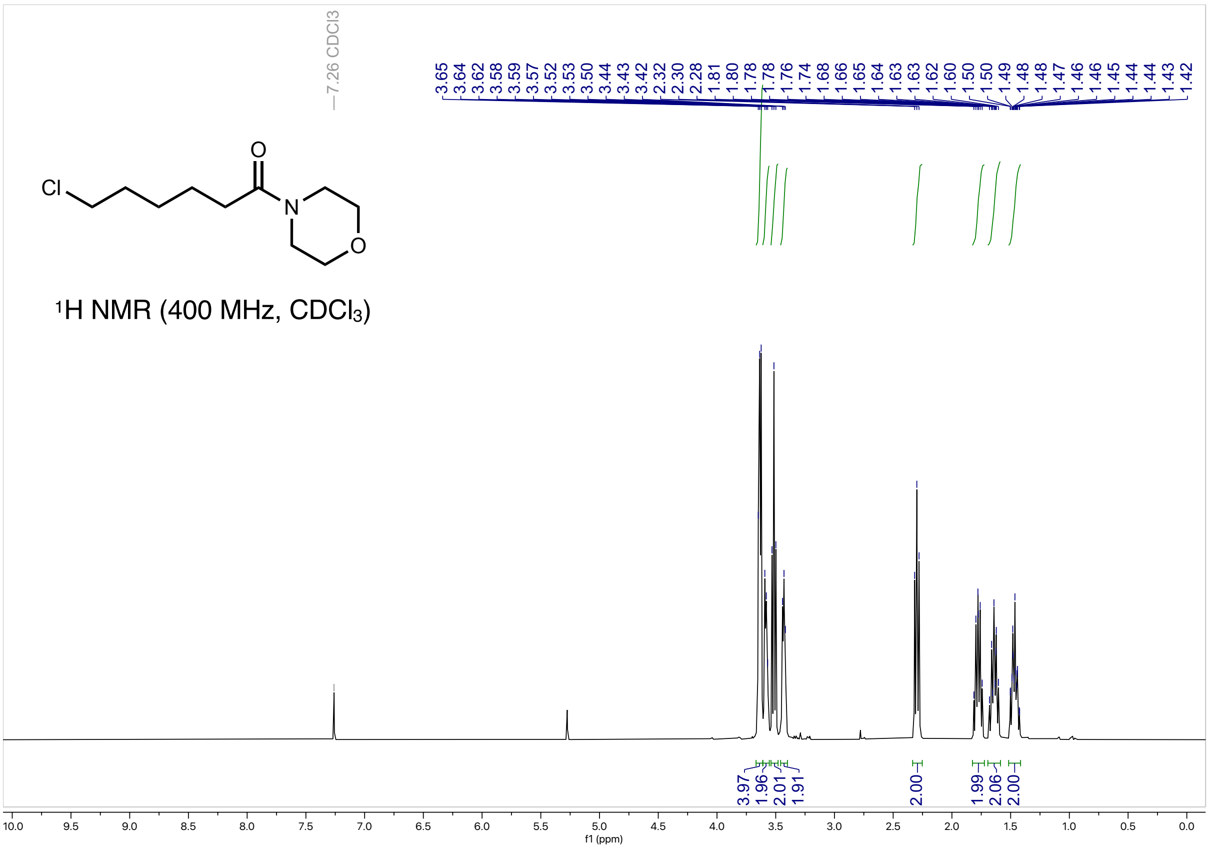
7.26 CDCl3
3.65
3.64
3.62
3.59
3.58
3.57
3.53
3.52
3.50
3.44
3.43
3.42
2.32
2.30
2.28
1.81
1.80
1.78
1.78
1.76
1.74
1.68
1.66
1.65
1.64
1.63
1.63
1.62
1.60
1.50
1.50
1.49
1.48
1.48
1.47
1.46
1.46
1.45
1.44
1.44
1.43
1.42
Cl
O
N
O
1H NMR (400 MHz, CDCl3)
3.97
1.96
2.01
1.91
2.00
1.99
2.06
2.00
f1 (ppm)

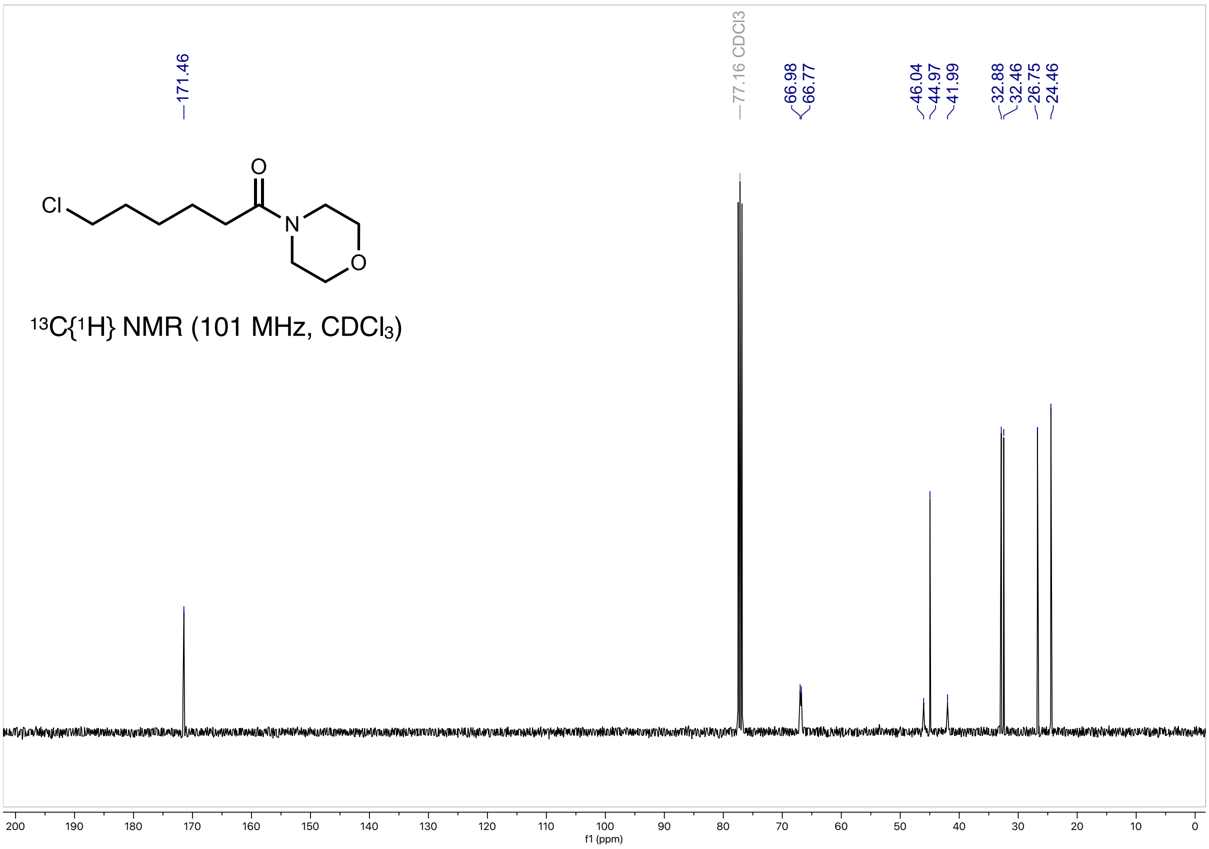
171.46
77.16 CDCl3
66.98
66.77
46.04
44.97
41.99
32.88
32.46
26.75
24.46
Cl
O
N
O
13C{1H} NMR (101 MHz, CDCl3)
f1 (ppm)

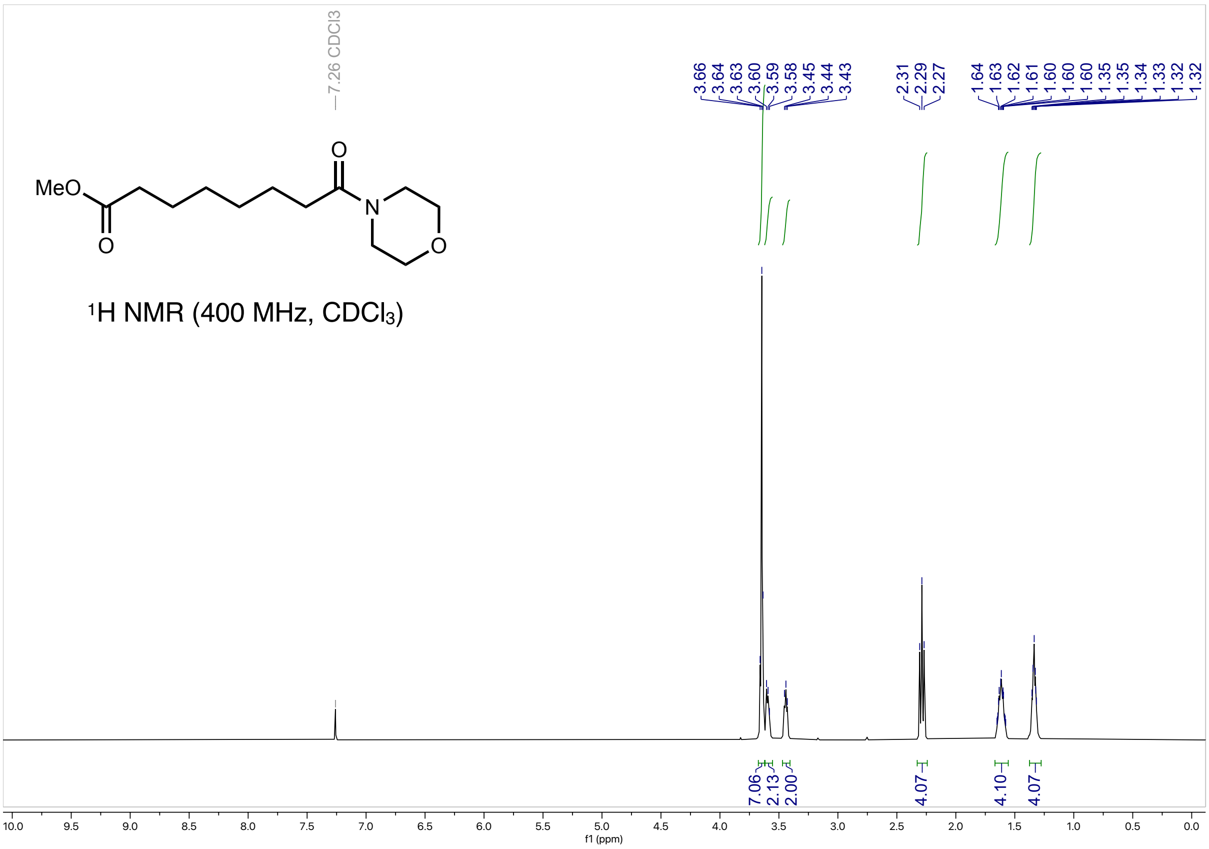

7.26 CDCl3
3.66
3.64
3.63
3.60
3.59
3.58
3.45
3.44
3.43
2.31
2.29
2.27
1.64
1.63
1.62
1.61
1.60
1.60
1.60
1.35
1.35
1.34
1.33
1.32
1.32
MeO
O
N
O
O
^{1}H NMR (400 MHz, CDCl$_3$)
7.06
2.13
2.00
4.07
4.10
4.07
f1 (ppm)

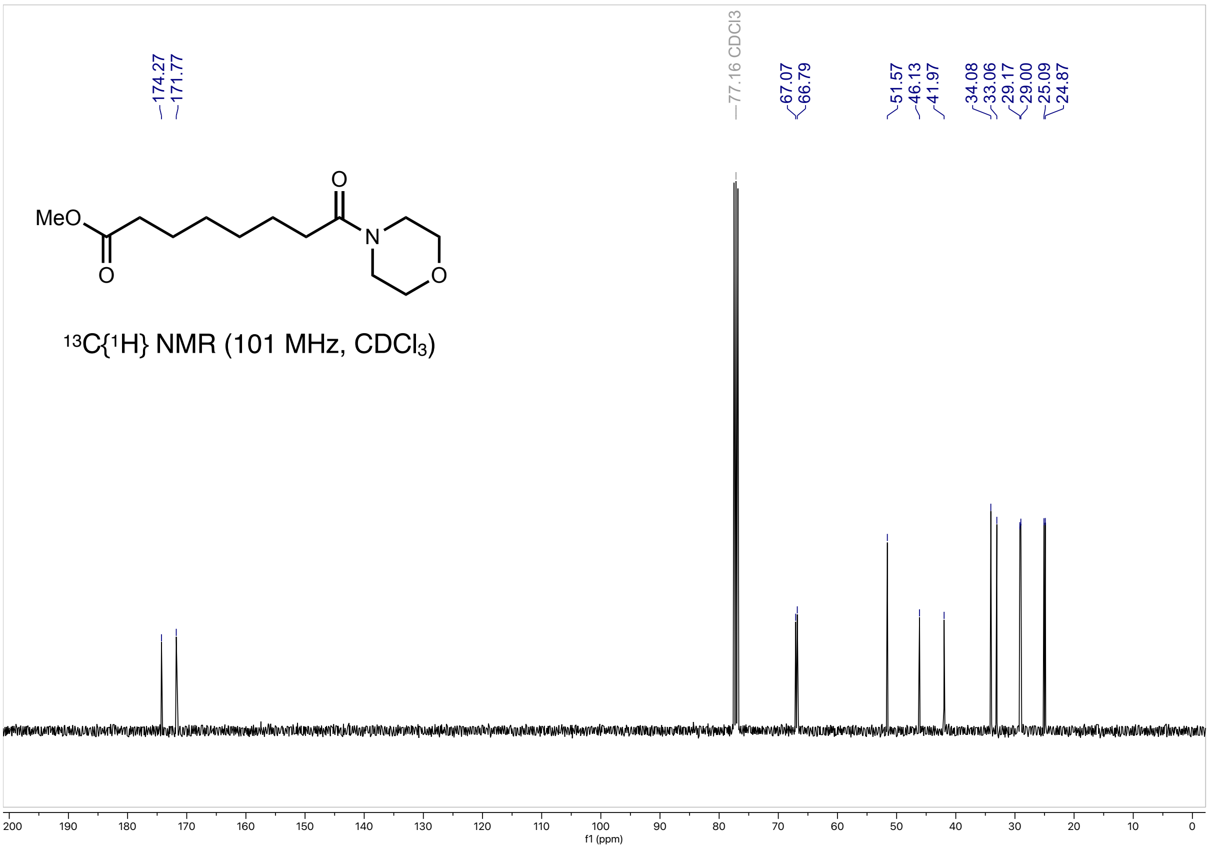

174.27
171.77
77.16 CDCl3
67.07
66.79
51.57
46.13
41.97
34.08
33.06
29.17
29.00
25.09
24.87
MeO
O
N
O
O
^{13}C{^{1}H} NMR (101 MHz, CDCl$_3$)
f1 (ppm)

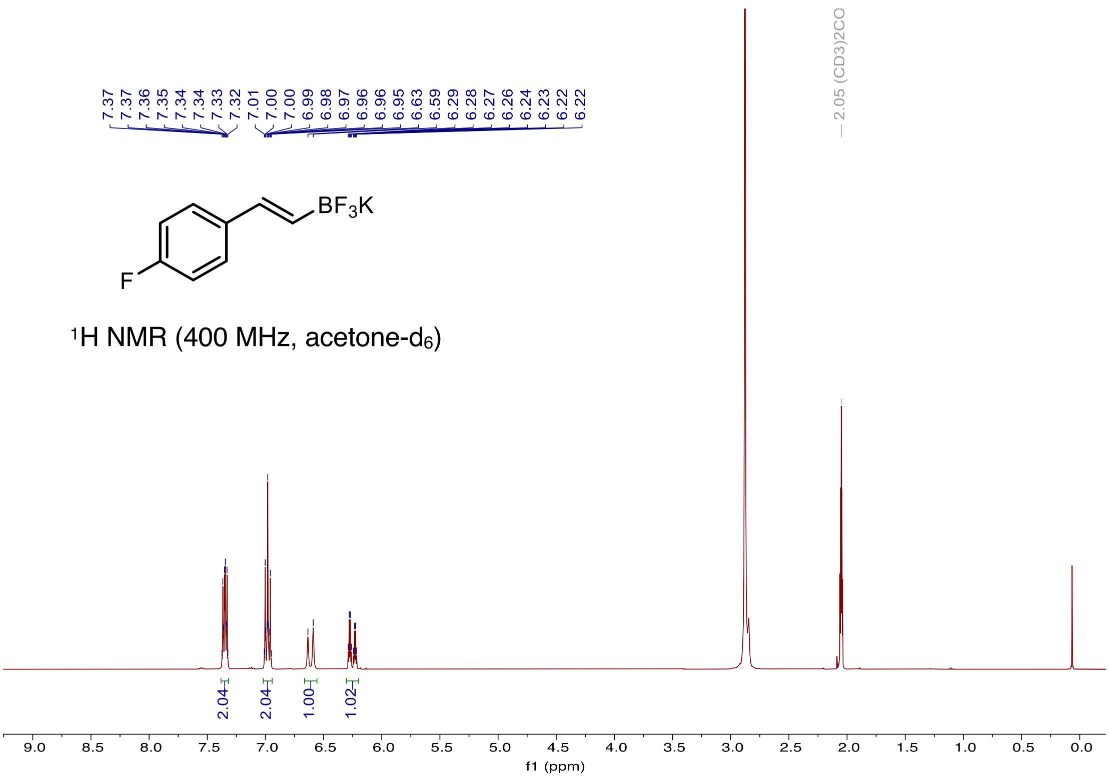
7.37
7.37
7.36
7.35
7.34
7.34
7.33
7.32
7.01
7.00
7.00
6.99
6.98
6.97
6.96
6.96
6.95
6.63
6.59
6.29
6.28
6.27
6.26
6.24
6.23
6.22
6.22
2.05 (CD3)2CO
BF3K
F
1H NMR (400 MHz, acetone-d6)
2.04
2.04
1.00
1.02
f1 (ppm)

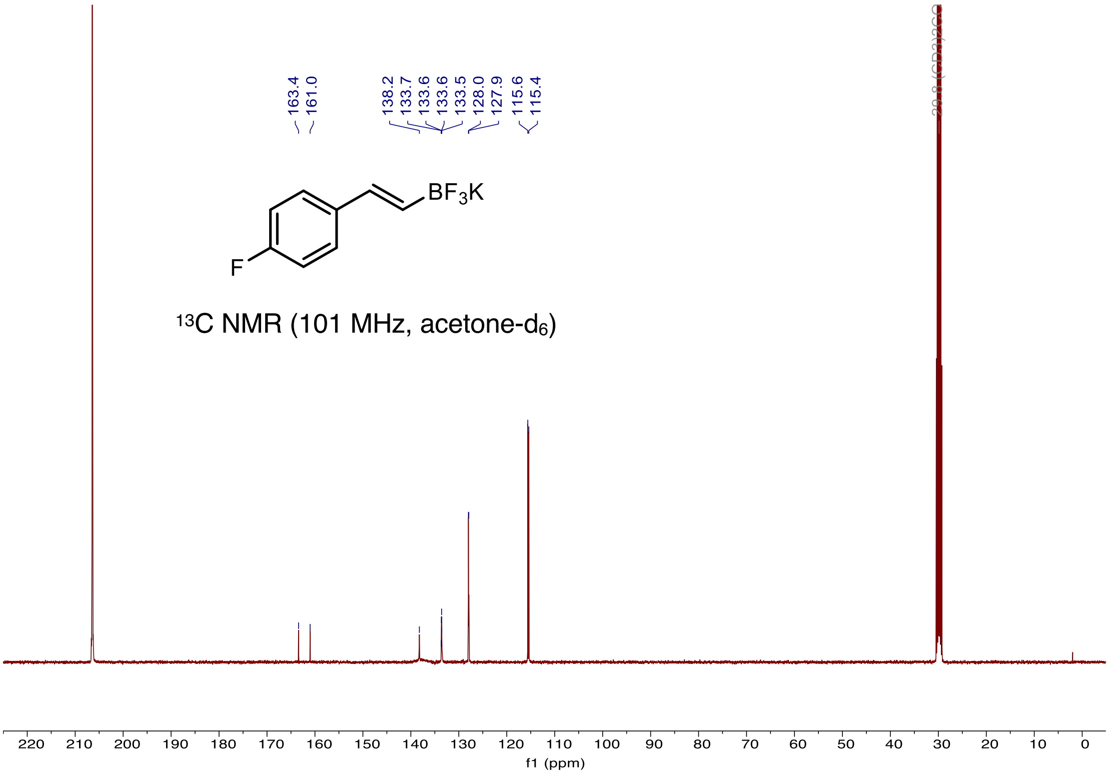
163.4
161.0
138.2
133.7
133.6
133.6
133.5
128.0
127.9
115.6
115.4
BF3K
F
13C NMR (101 MHz, acetone-d6)
f1 (ppm)

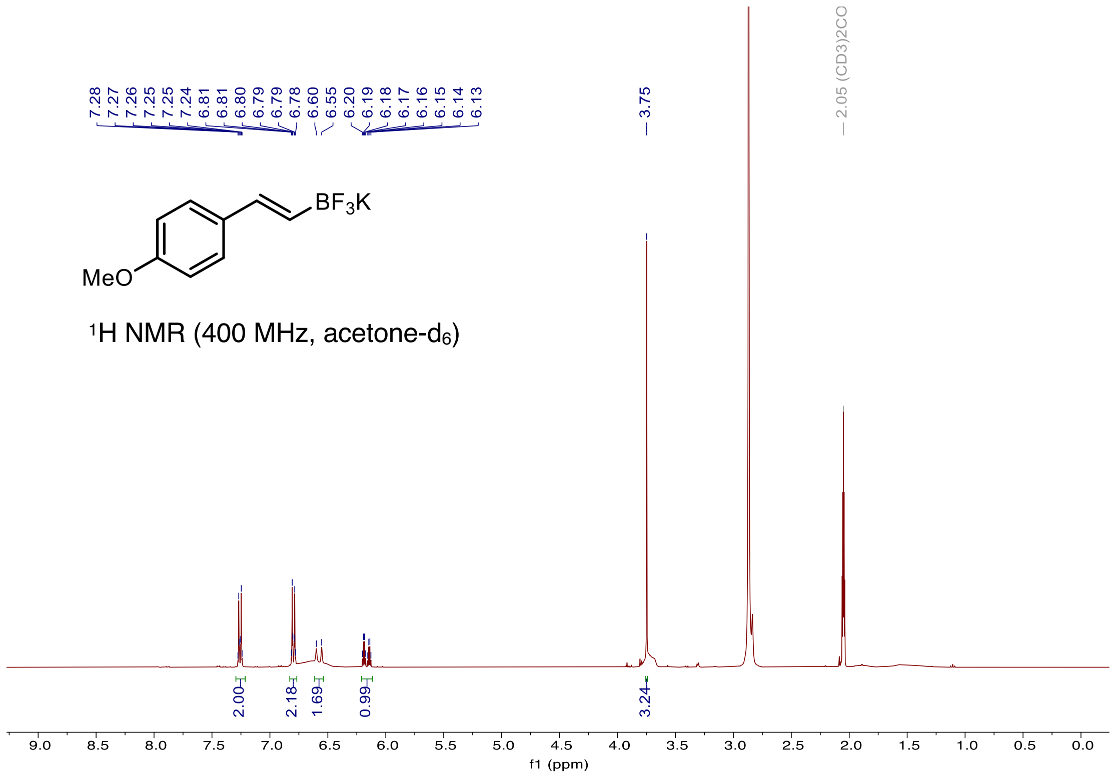

^{1}H NMR (400 MHz, acetone-d_6)

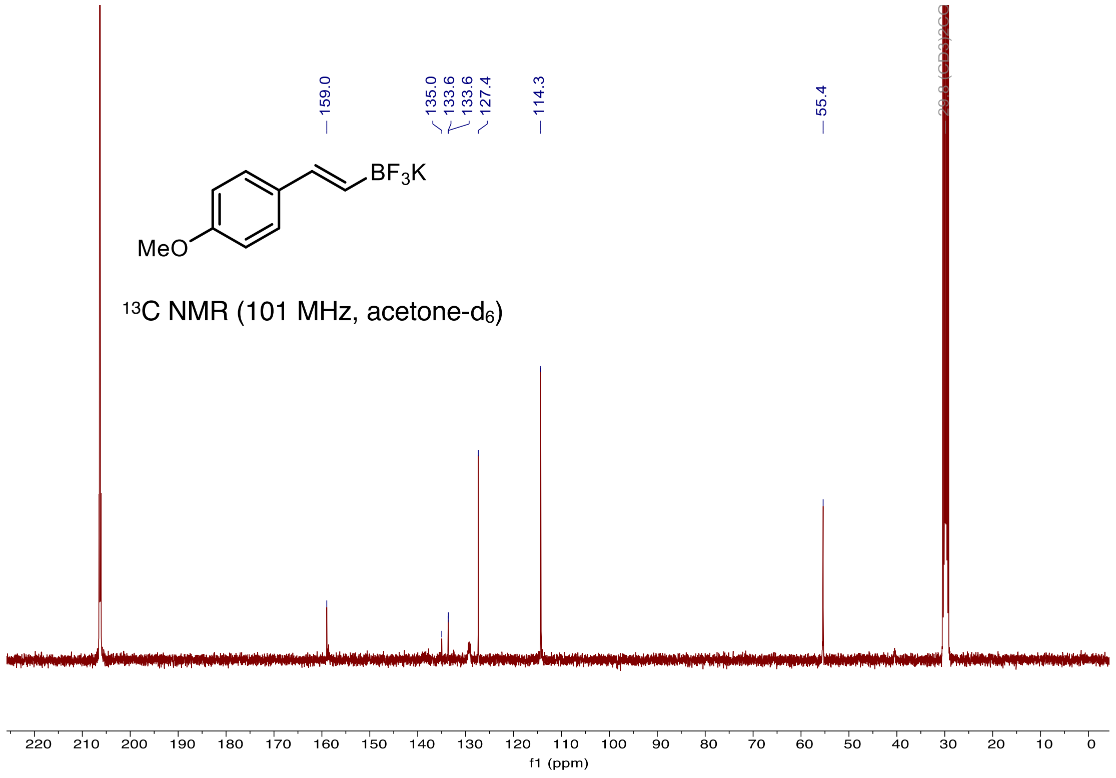

^{13}C NMR (101 MHz, acetone-d_6)

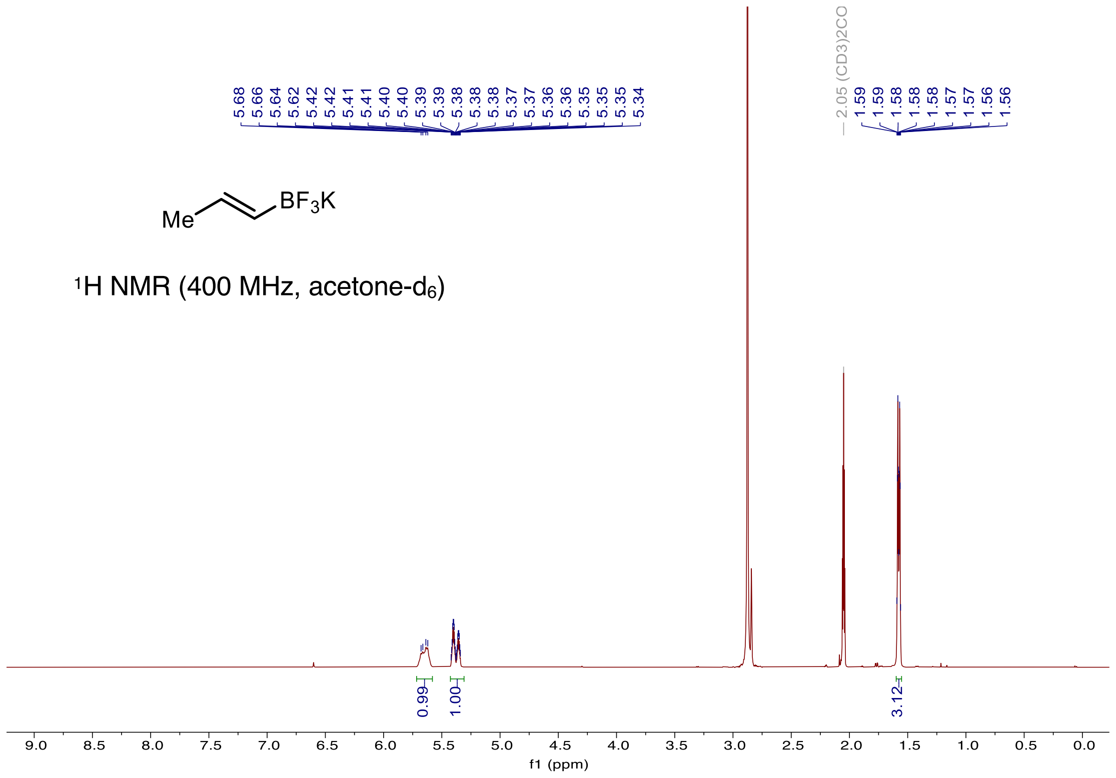
5.68
5.66
5.64
5.62
5.42
5.42
5.41
5.41
5.40
5.40
5.39
5.39
5.38
5.38
5.38
5.37
5.37
5.36
5.36
5.35
5.35
5.35
5.34
2.05 (CD3)2CO
1.59
1.59
1.58
1.58
1.58
1.57
1.57
1.56
1.56
Me
BF3K
1H NMR (400 MHz, acetone-d6)
0.99
1.00
3.12
f1 (ppm)

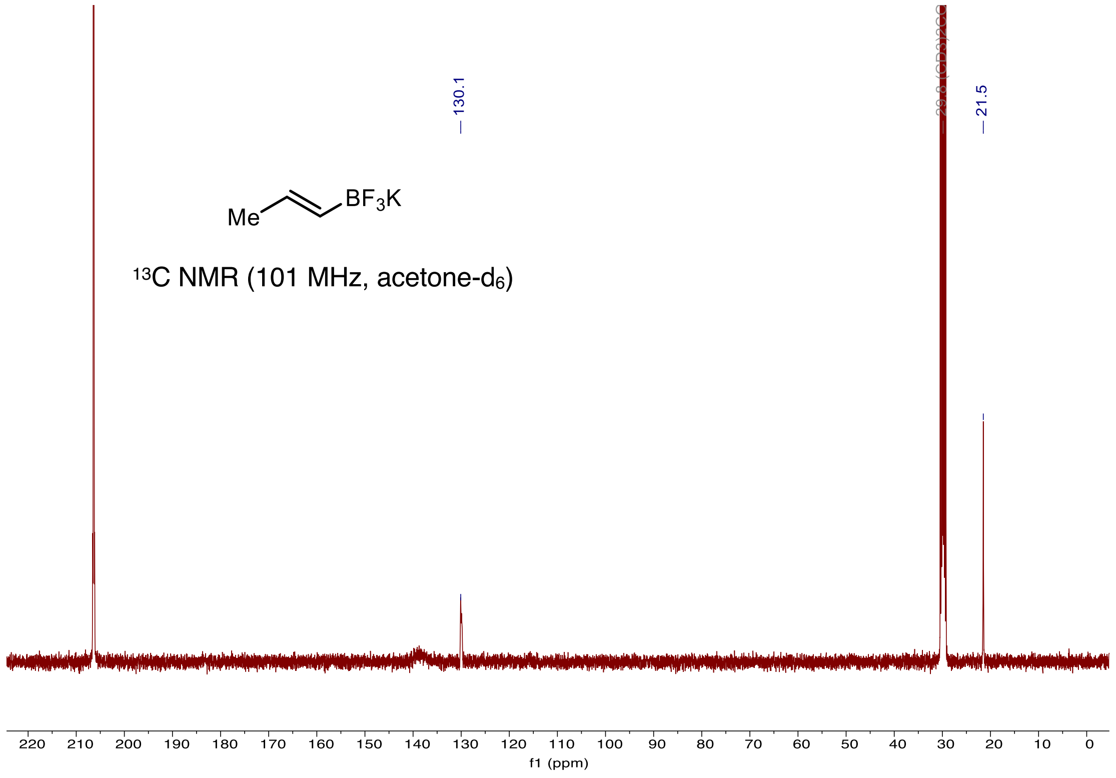
130.1
21.5
Me
BF3K
13C NMR (101 MHz, acetone-d6)
f1 (ppm)

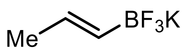

^{19}F NMR (376 MHz, acetone-d$_6$)

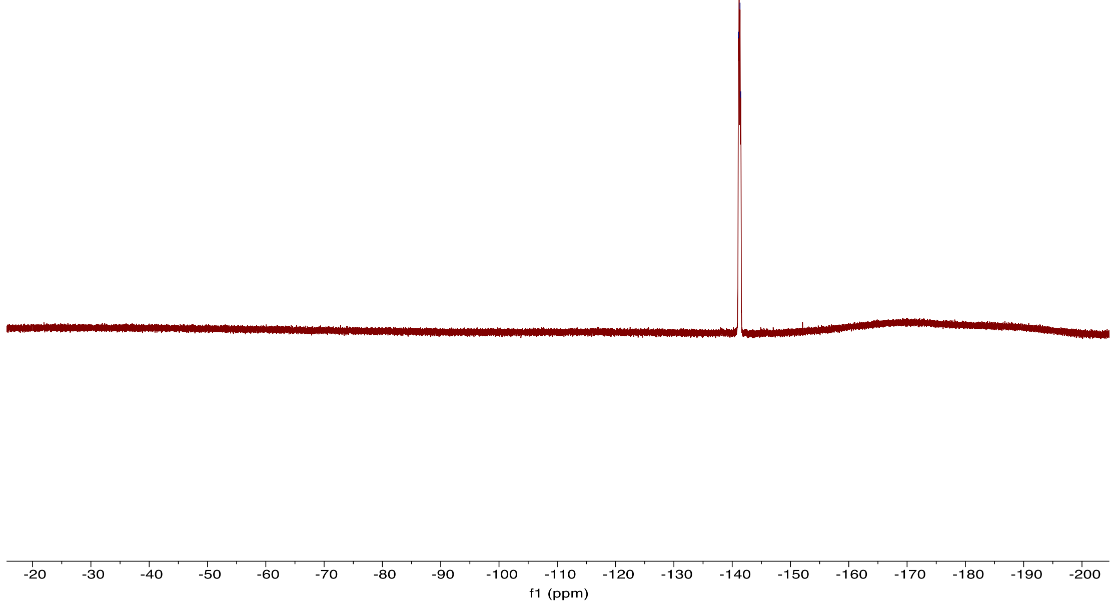

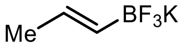

^{11}B NMR (126 MHz, acetone-d$_6$)

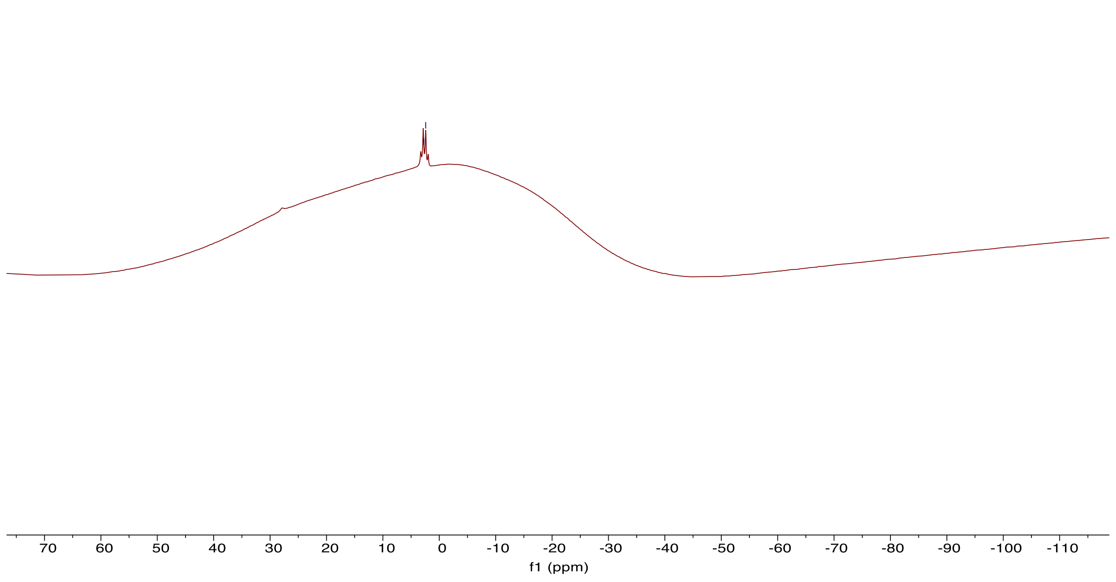

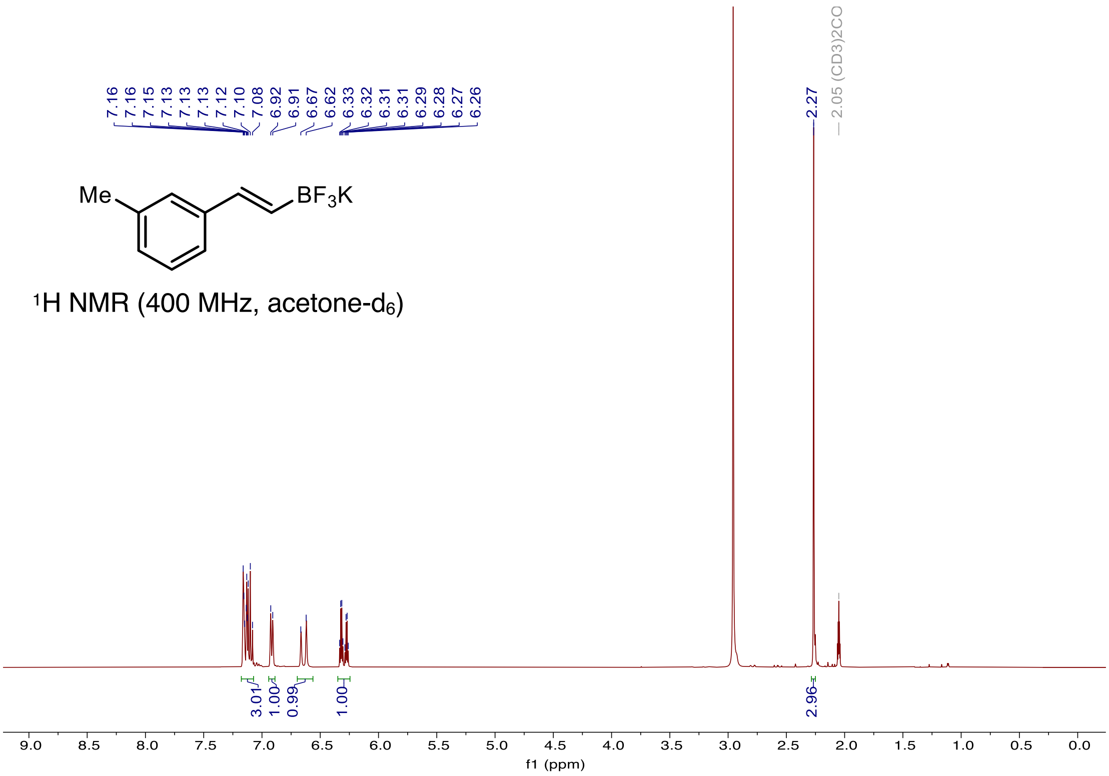
7.16
7.16
7.15
7.13
7.13
7.13
7.12
7.10
7.08
6.92
6.91
6.67
6.62
6.33
6.32
6.31
6.29
6.28
6.27
6.26
2.27
2.05 (CD3)2CO
Me
BF3K
1H NMR (400 MHz, acetone-d6)
3.01
1.00
0.99
1.00
2.96
9.0
8.5
8.0
7.5
7.0
6.5
6.0
5.5
5.0
4.5
4.0
3.5
3.0
2.5
2.0
1.5
1.0
0.5
0.0
f1 (ppm)

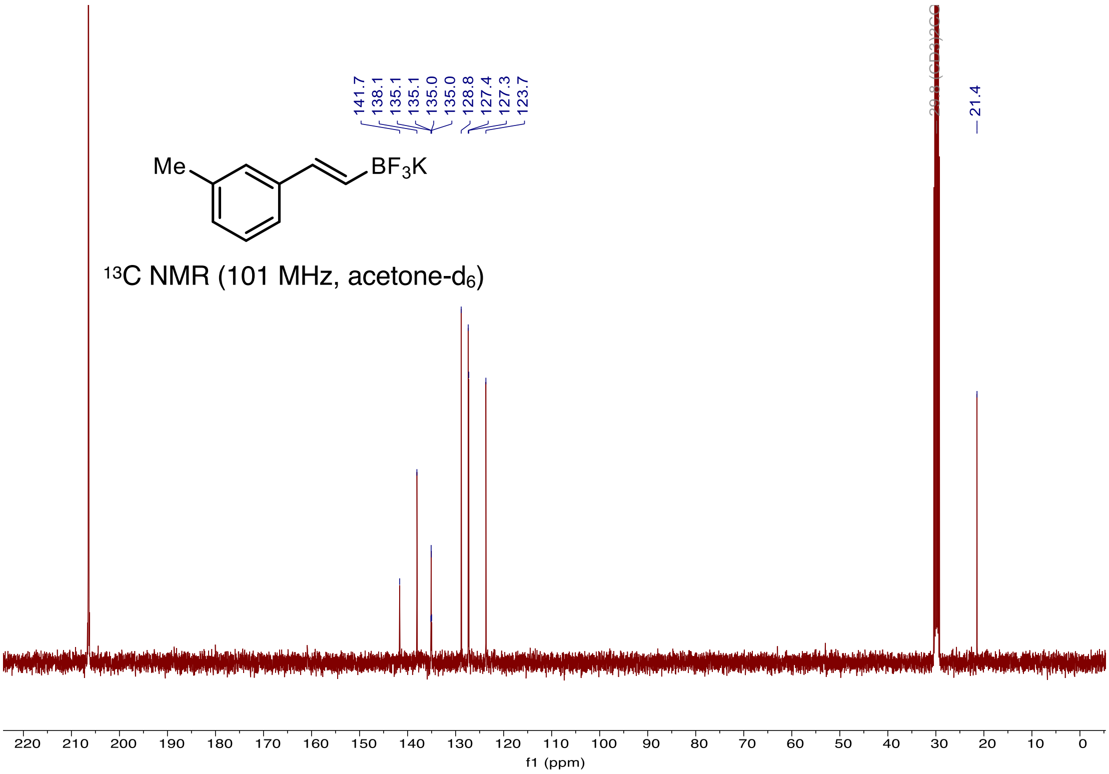
141.7
138.1
135.1
135.1
135.0
135.0
128.8
127.4
127.3
123.7
21.4
Me
BF3K
13C NMR (101 MHz, acetone-d6)
220
210
200
190
180
170
160
150
140
130
120
110
100
90
80
70
60
50
40
30
20
10
0
f1 (ppm)

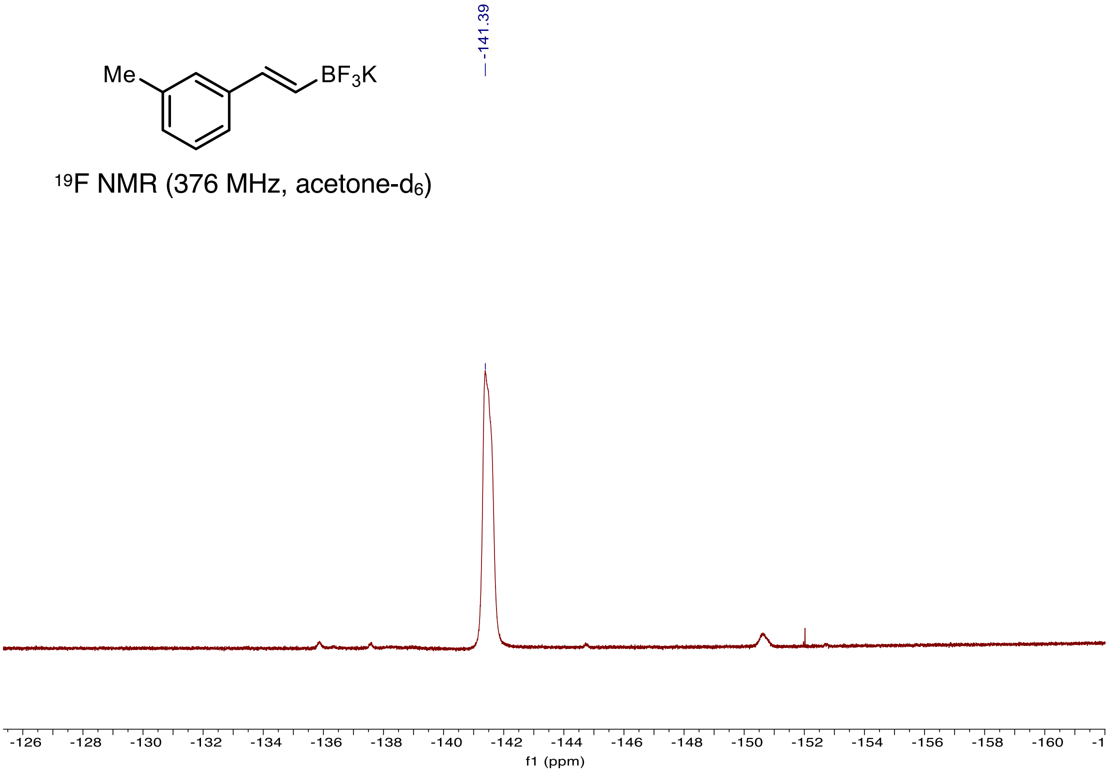

Me
BF3K
19F NMR (376 MHz, acetone-d6)
-141.39
-126 -128 -130 -132 -134 -136 -138 -140 -142 -144 -146 -148 -150 -152 -154 -156 -158 -160
f1 (ppm)

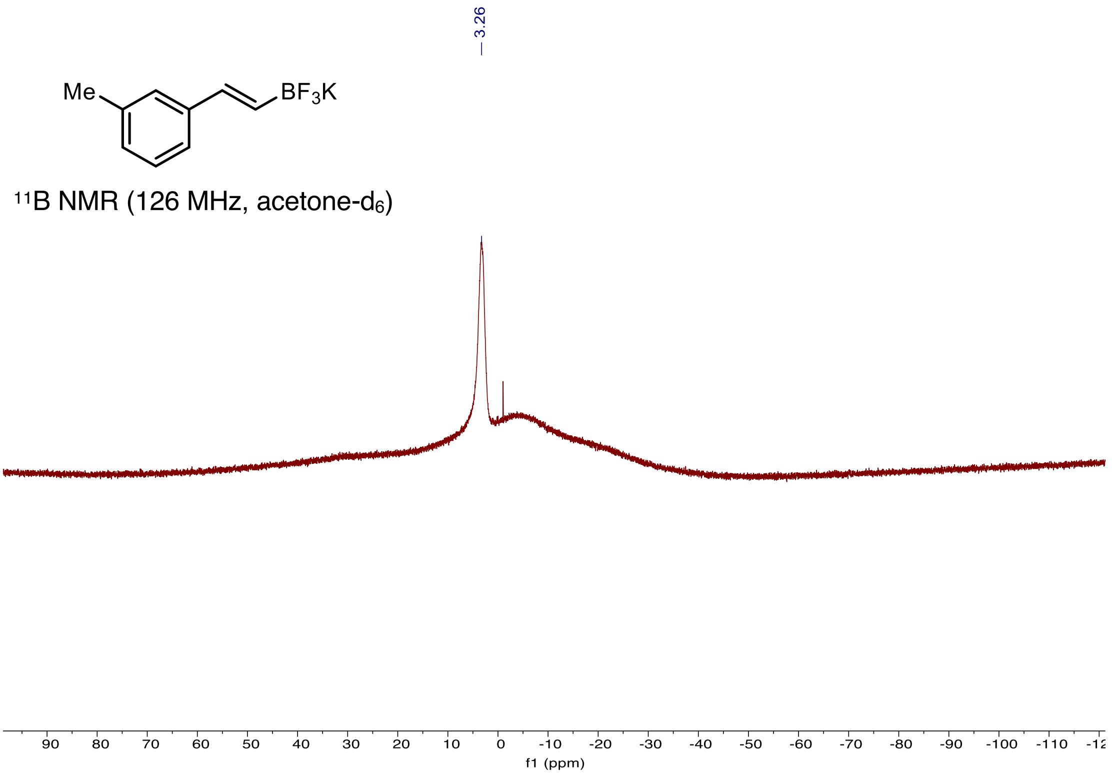

Me
BF3K
11B NMR (126 MHz, acetone-d6)
3.26
90 80 70 60 50 40 30 20 10 0 -10 -20 -30 -40 -50 -60 -70 -80 -90 -100 -110
f1 (ppm)

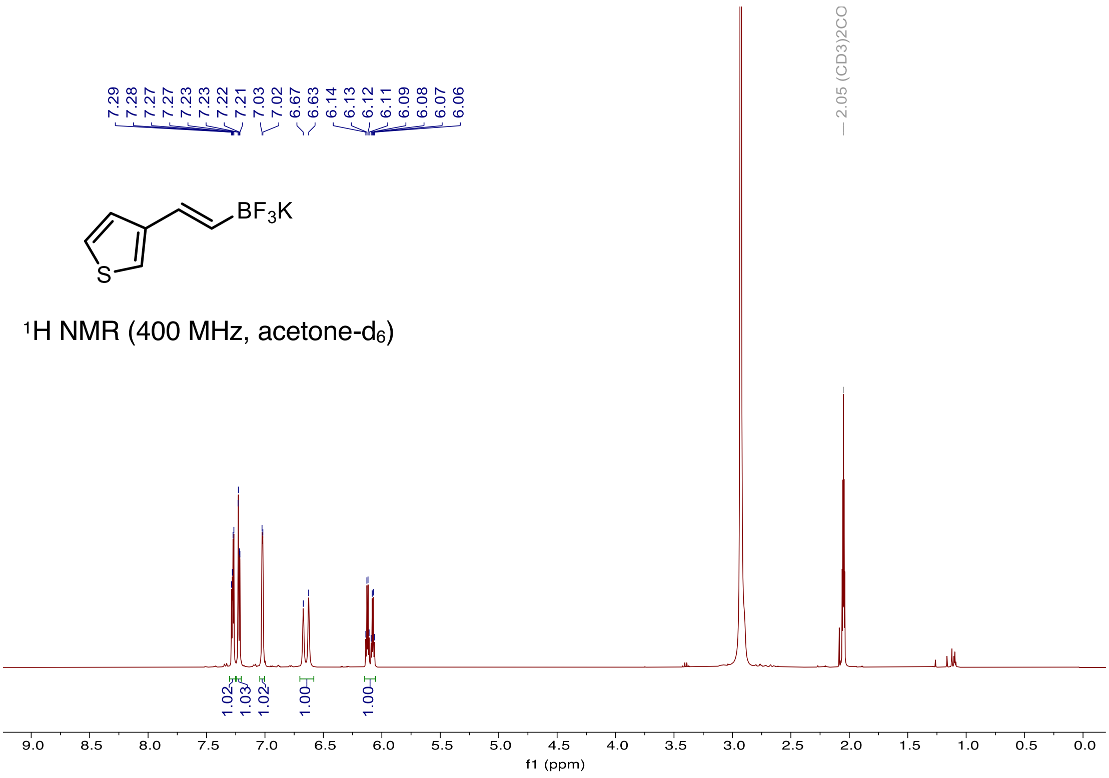
7.29
7.28
7.27
7.27
7.23
7.23
7.22
7.21
7.03
7.02
6.67
6.63
6.14
6.13
6.12
6.11
6.09
6.08
6.07
6.06
2.05 (CD3)2CO
BF3K
S
1H NMR (400 MHz, acetone-d6)
1.02
1.03
1.02
1.00
1.00
9.0
8.5
8.0
7.5
7.0
6.5
6.0
5.5
5.0
4.5
4.0
3.5
3.0
2.5
2.0
1.5
1.0
0.5
0.0
f1 (ppm)

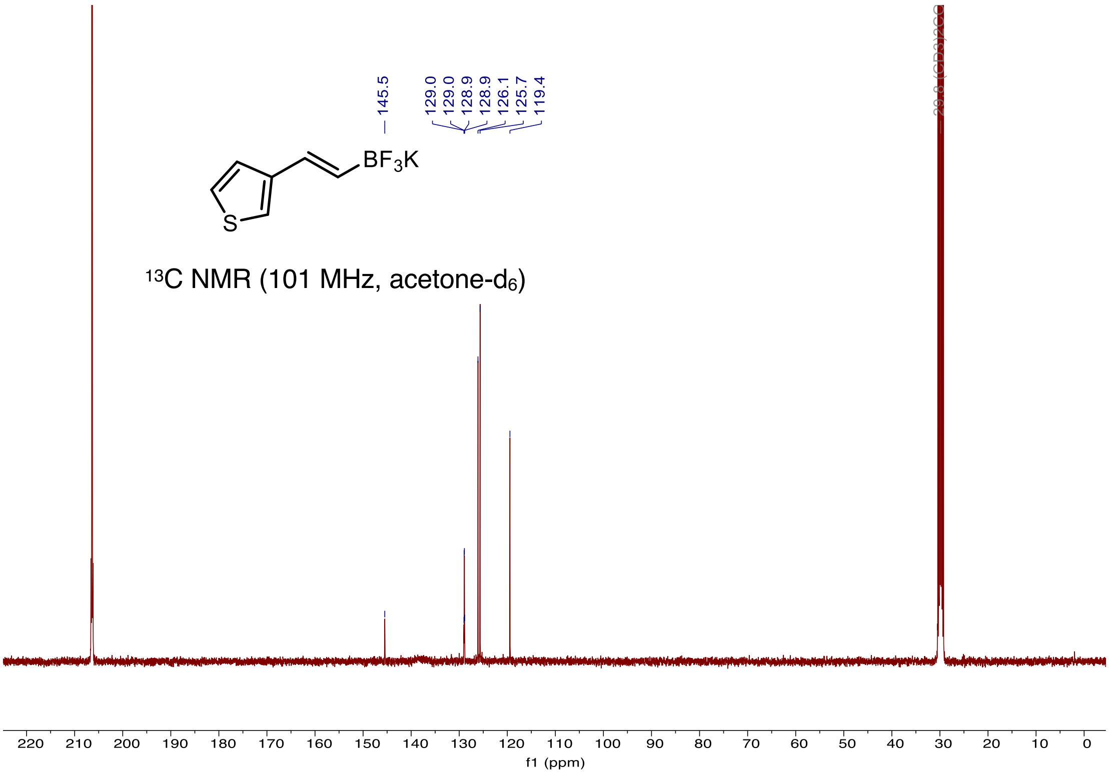
145.5
129.0
129.0
128.9
128.9
126.1
125.7
119.4
BF3K
S
13C NMR (101 MHz, acetone-d6)
220
210
200
190
180
170
160
150
140
130
120
110
100
90
80
70
60
50
40
30
20
10
0
f1 (ppm)

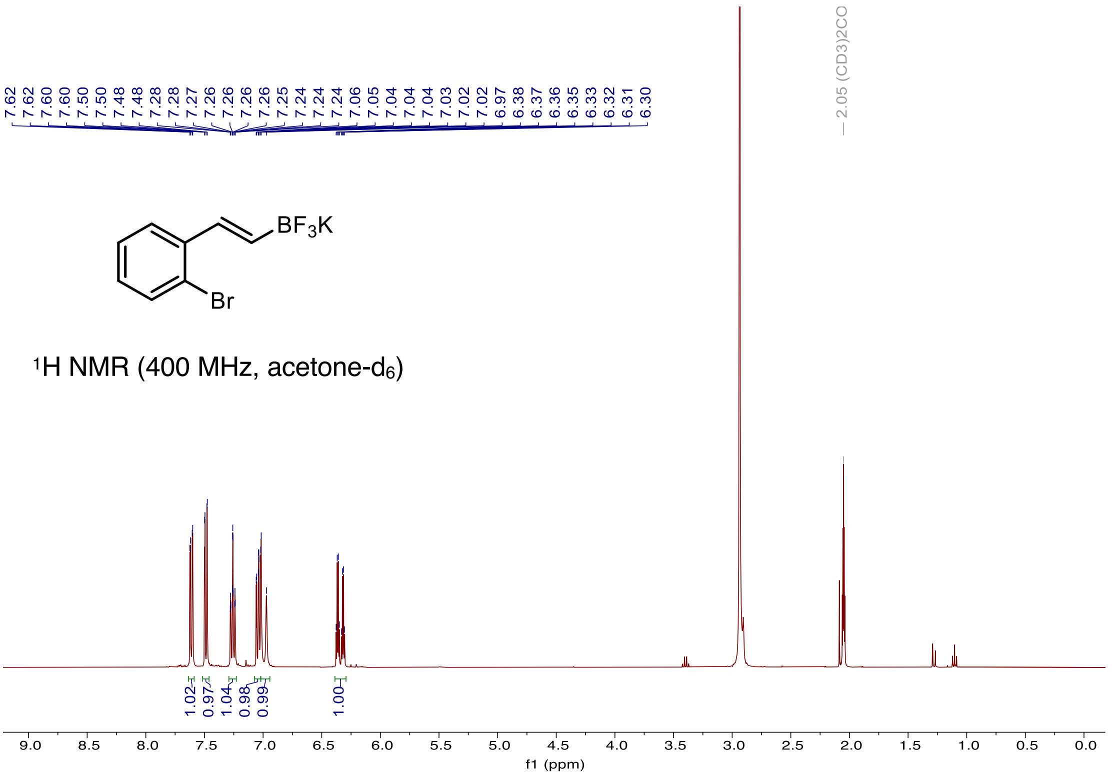
7.62 7.62 7.60 7.60 7.50 7.50 7.48 7.48 7.28 7.28 7.27 7.26 7.26 7.26 7.26 7.25 7.24 7.24 7.24 7.24 7.06 7.05 7.04 7.04 7.04 7.03 7.02 7.02 6.97 6.38 6.37 6.36 6.35 6.33 6.32 6.31 6.30
2.05 (CD3)2CO
BF3K
Br
1H NMR (400 MHz, acetone-d6)
1.02 0.97 1.04 0.98 0.99 1.00
9.0 8.5 8.0 7.5 7.0 6.5 6.0 5.5 5.0 4.5 4.0 3.5 3.0 2.5 2.0 1.5 1.0 0.5 0.0
f1 (ppm)
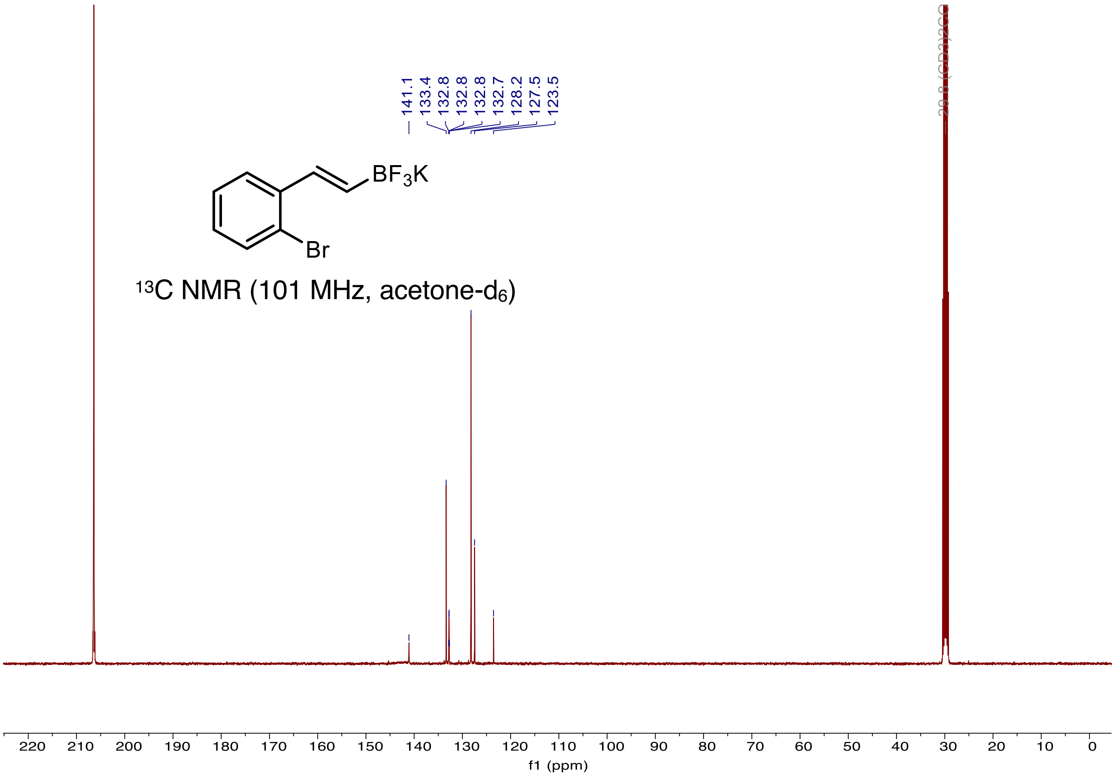
141.1 133.4 132.8 132.8 132.8 132.7 128.2 127.5 123.5
BF3K
Br
13C NMR (101 MHz, acetone-d6)
220 210 200 190 180 170 160 150 140 130 120 110 100 90 80 70 60 50 40 30 20 10 0
f1 (ppm)

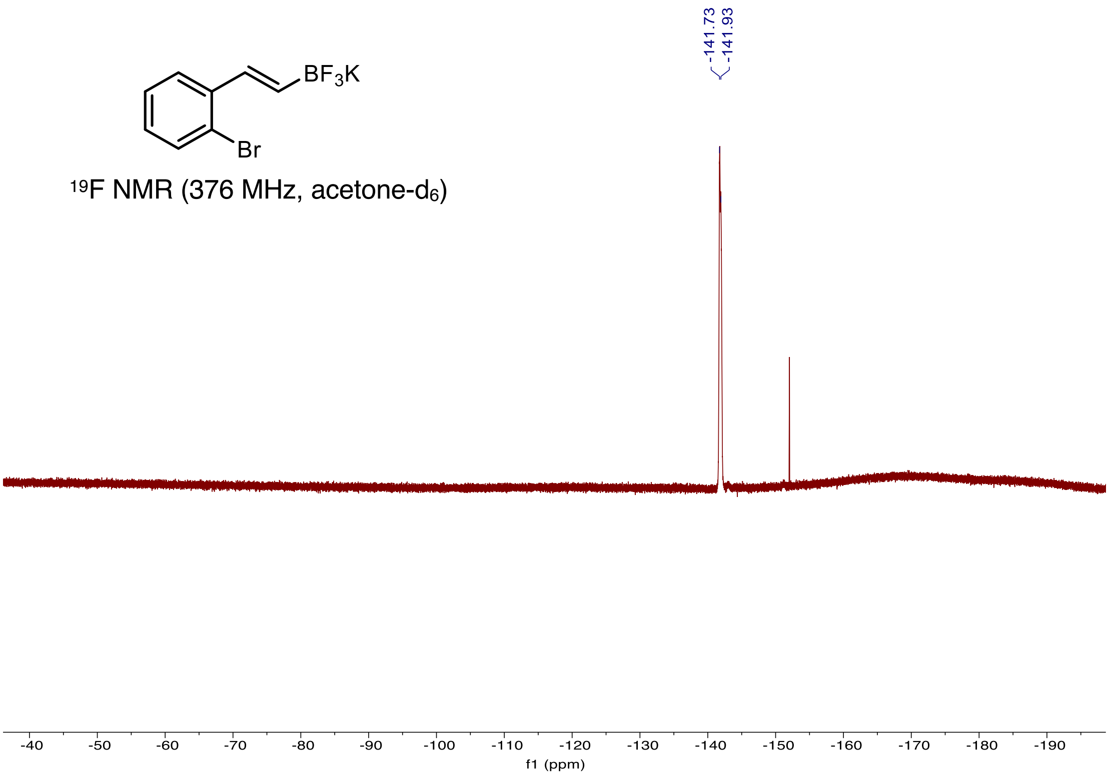

^{19}F NMR (376 MHz, acetone-d$_6$)

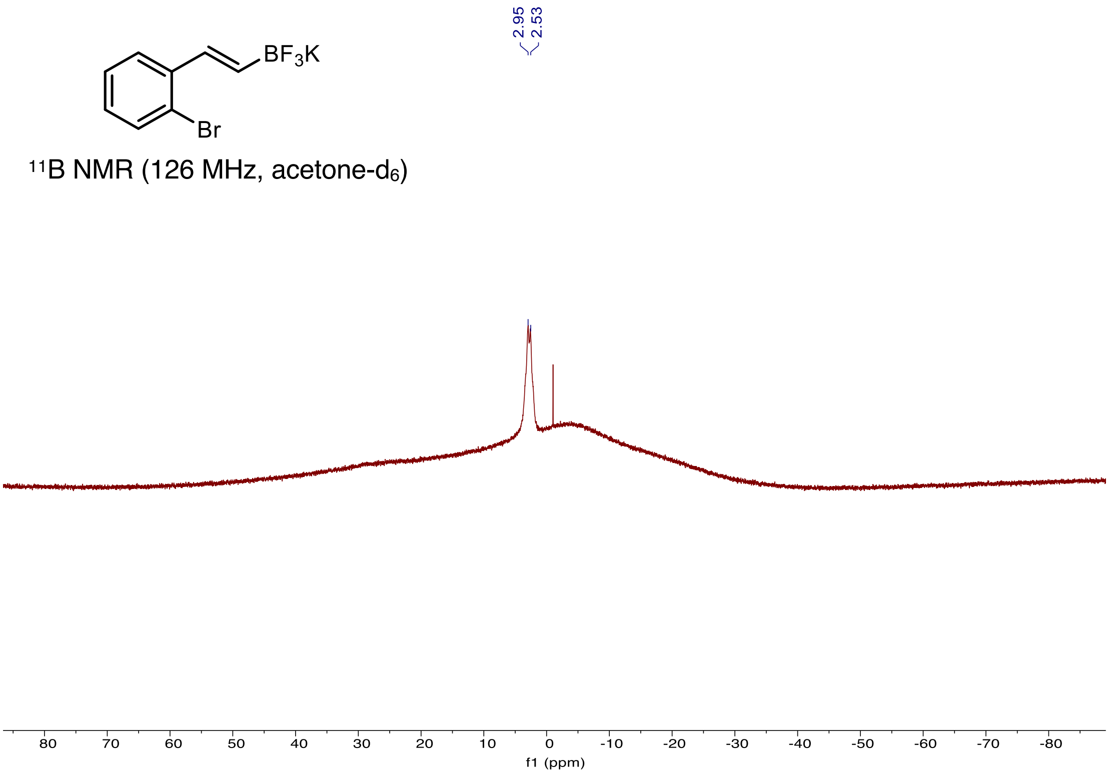

^{11}B NMR (126 MHz, acetone-d$_6$)

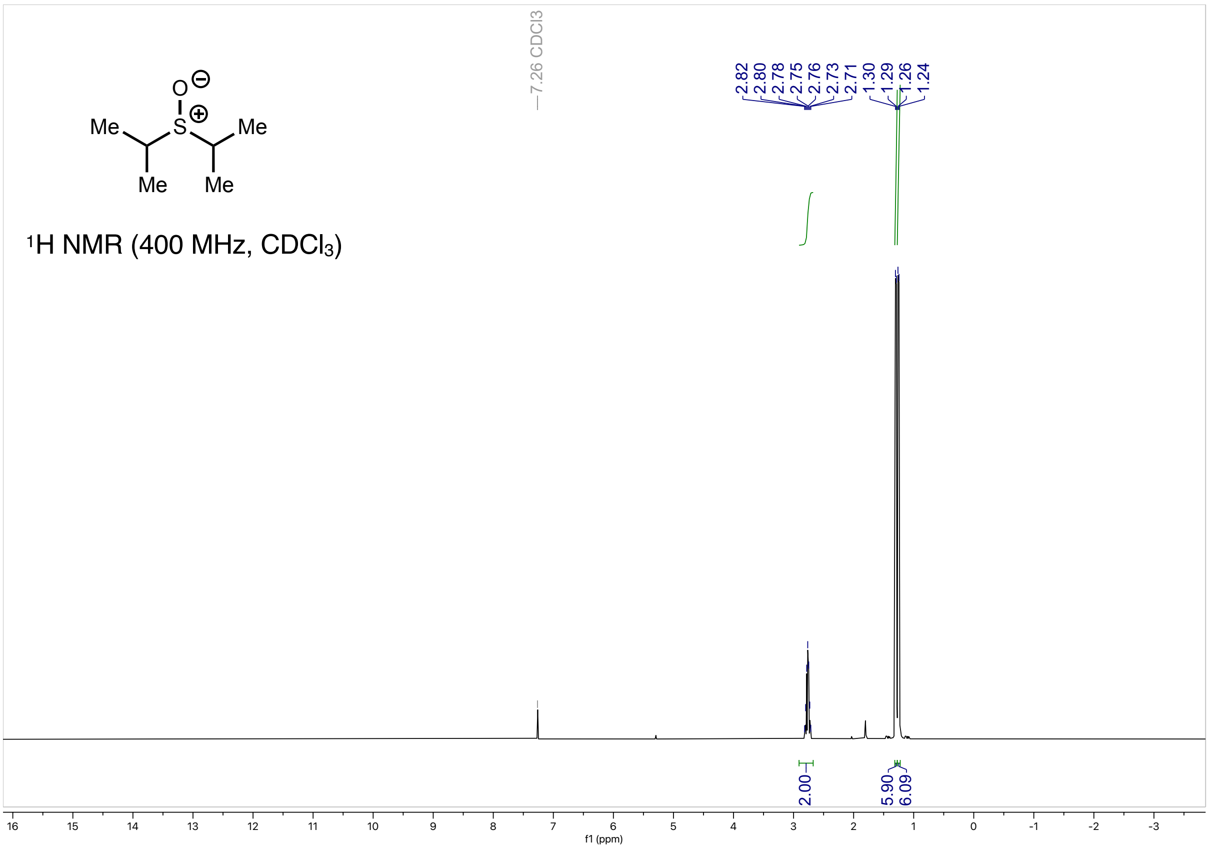

Me
S
O
Me
Me
Me
1H NMR (400 MHz, CDCl3)
7.26 CDCl3
2.82
2.80
2.78
2.76
2.75
2.73
2.71
1.30
1.29
1.26
1.24
2.00
5.90
6.09
f1 (ppm)

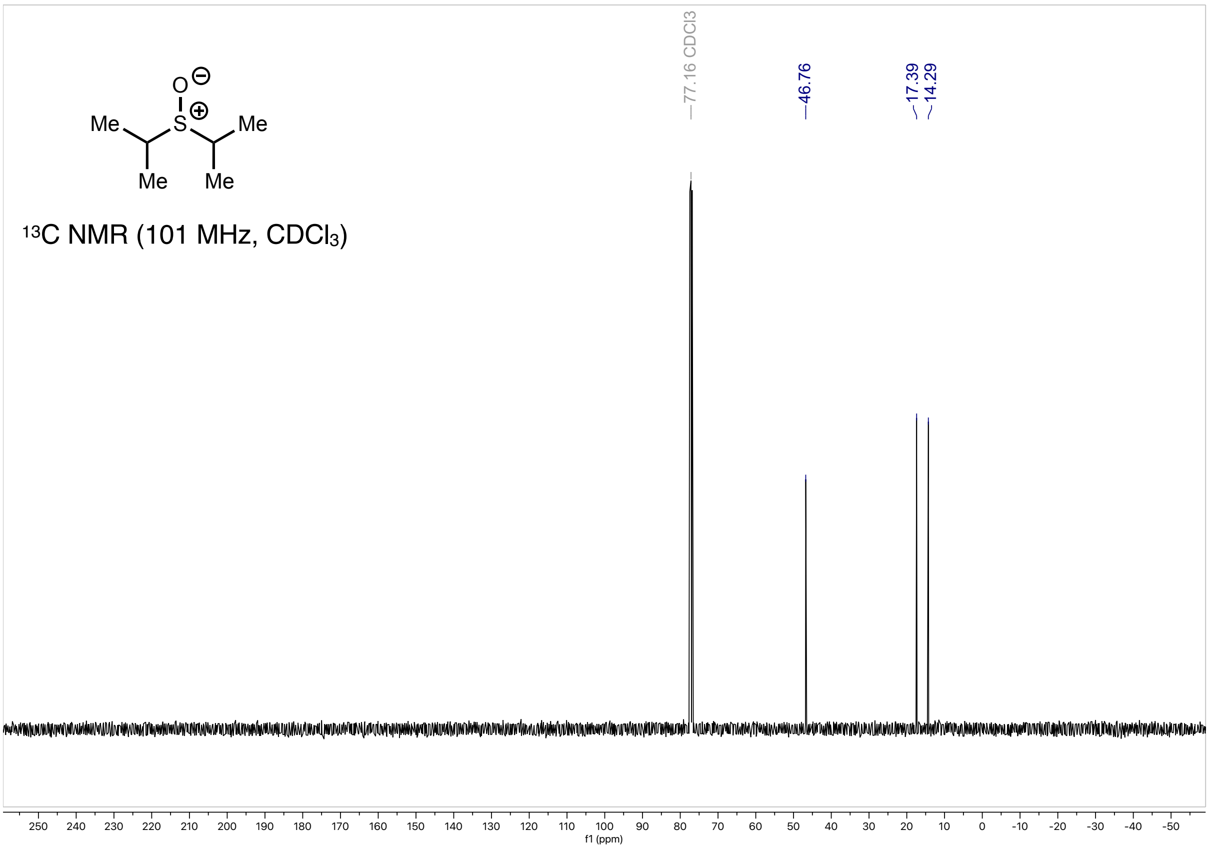

Me
S
O
Me
Me
Me
13C NMR (101 MHz, CDCl3)
77.16 CDCl3
46.76
17.39
14.29
f1 (ppm)

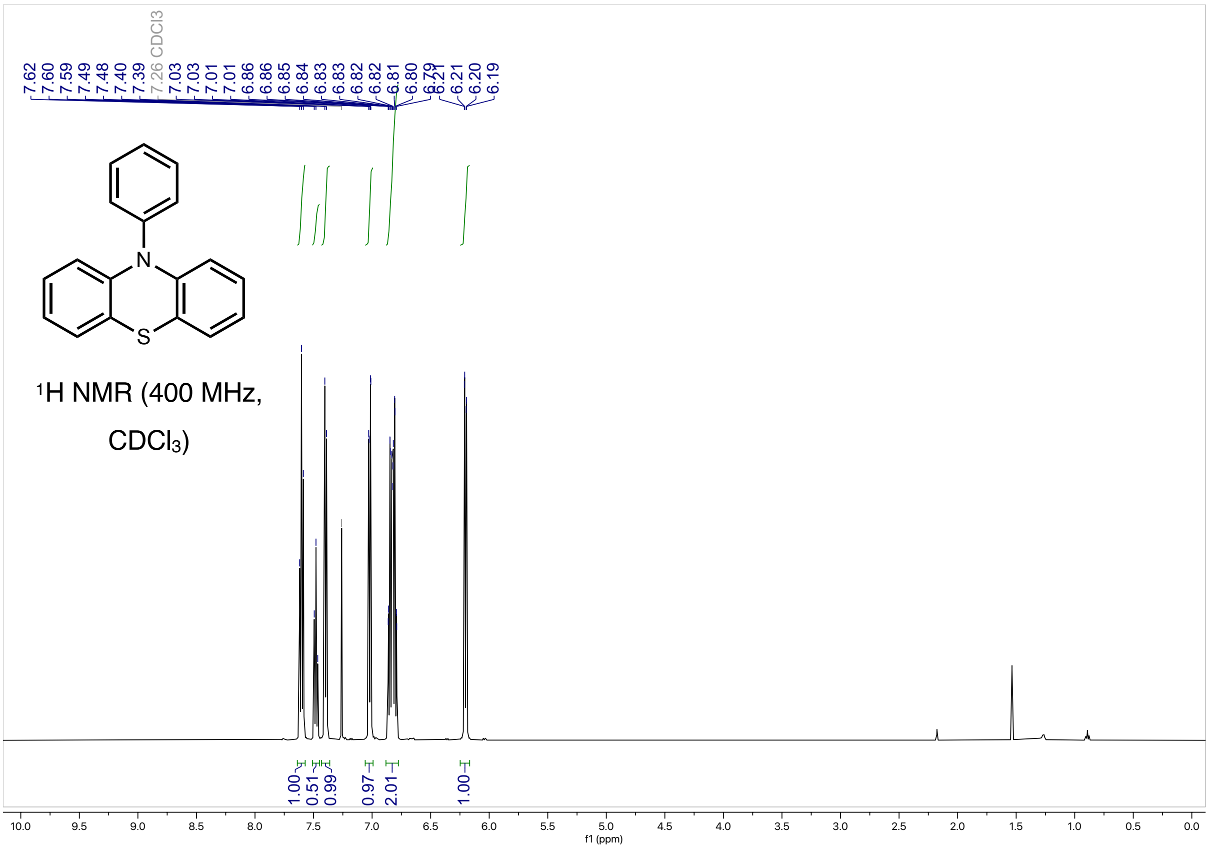

7.62 7.60 7.59 7.49 7.48 7.40 7.39 7.26 CDCl3 7.03 7.03 7.01 7.01 6.86 6.85 6.84 6.83 6.83 6.82 6.82 6.81 6.80 6.79 6.21 6.21 6.20 6.19
N
S
1H NMR (400 MHz, CDCl3)
1.00 0.51 0.99 0.97 2.01 1.00
f1 (ppm)

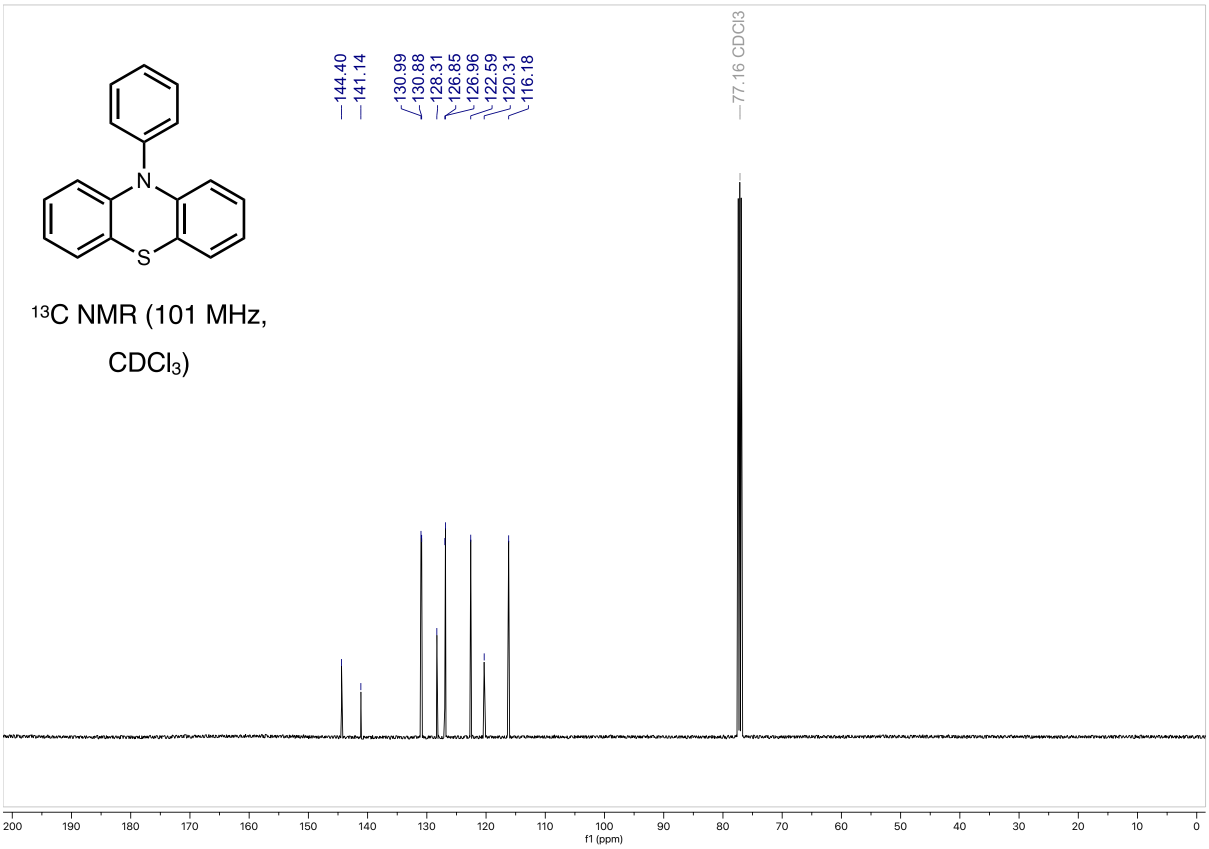

144.40 141.14 130.99 130.88 128.31 126.85 126.96 122.59 120.31 116.18
77.16 CDCl3
N
S
13C NMR (101 MHz, CDCl3)
f1 (ppm)

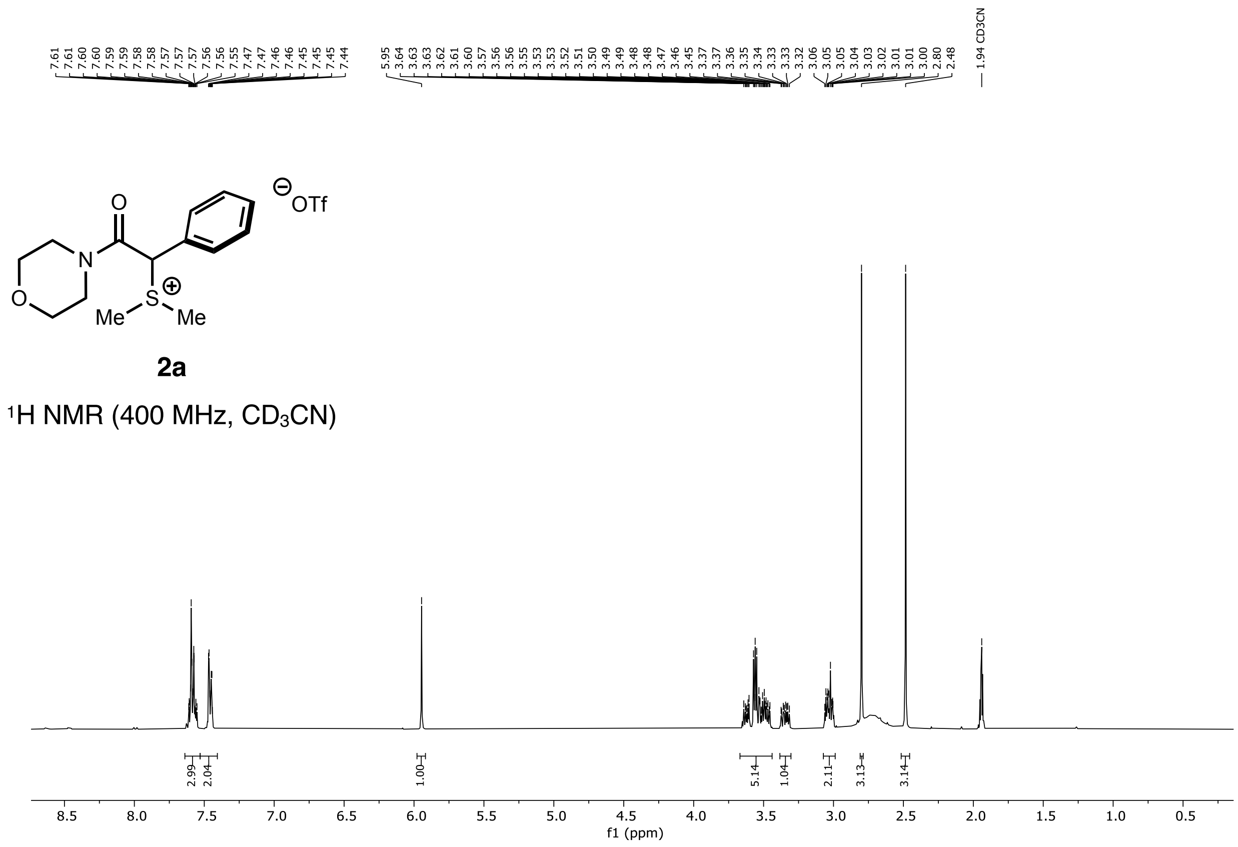

2a

^{1}H NMR (400 MHz, CD_3CN)

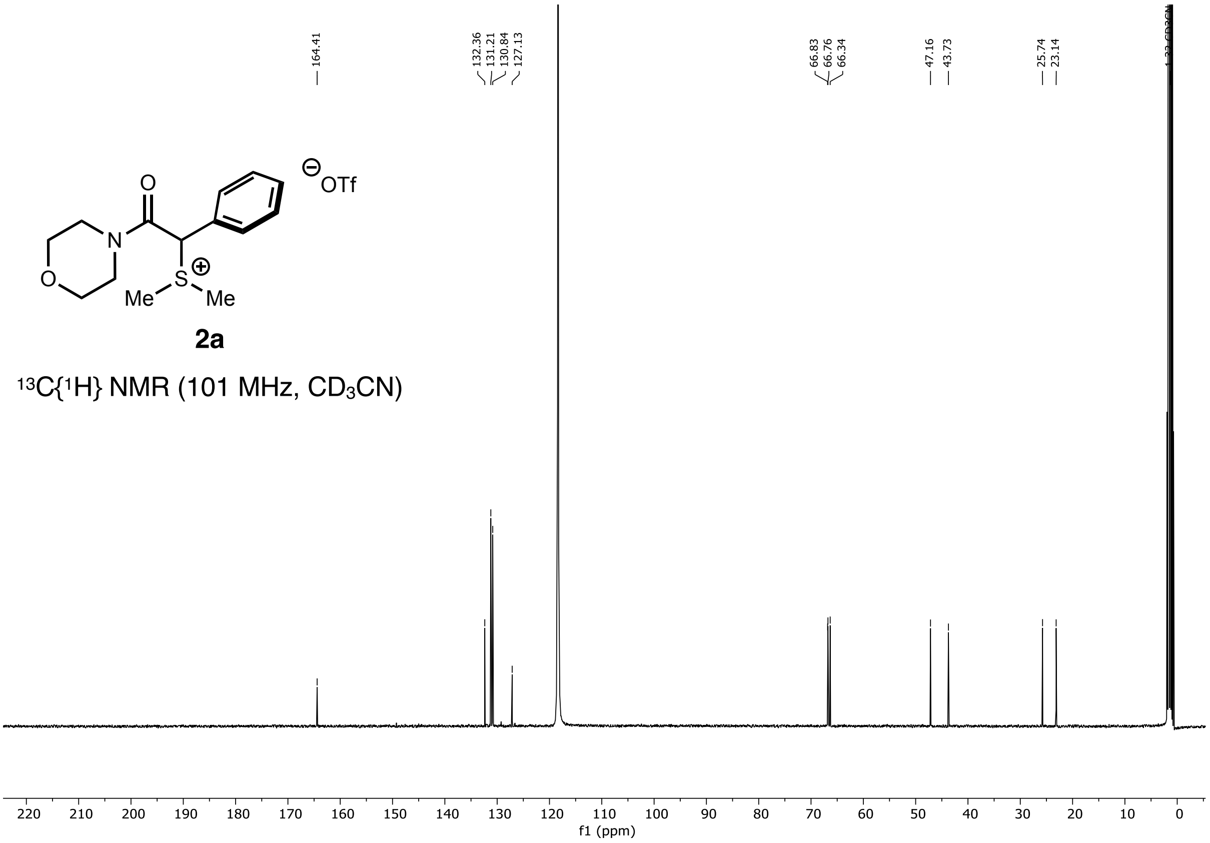

2a

$^{13}C\{^{1}H\}$ NMR (101 MHz, CD_3CN)

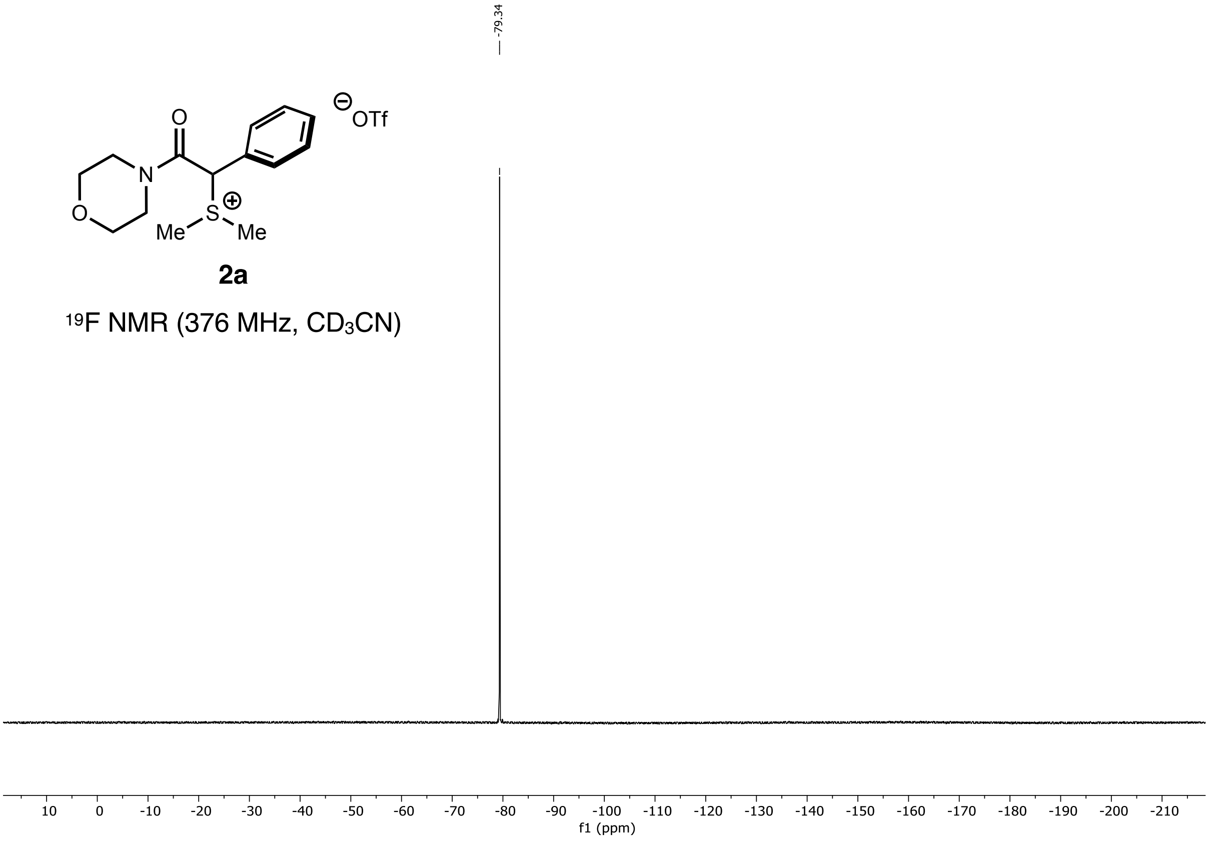

OTf
O
N
O
S
Me
Me
2a
19F NMR (376 MHz, CD3CN)
-79.34
10
0
-10
-20
-30
-40
-50
-60
-70
-80
-90
-100
-110
-120
-130
-140
-150
-160
-170
-180
-190
-200
-210
f1 (ppm)

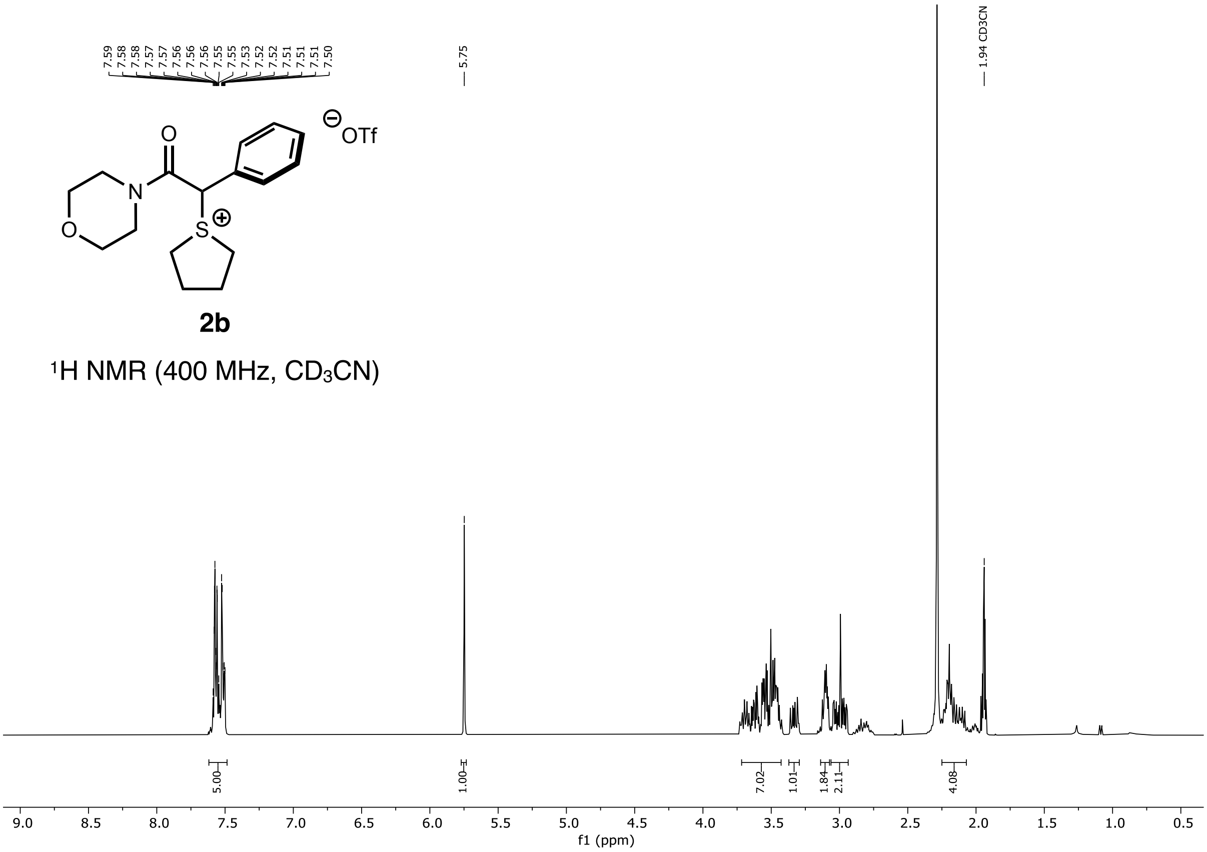

7.59 7.58 7.58 7.57 7.57 7.56 7.56 7.55 7.55 7.53 7.52 7.52 7.51 7.51 7.51 7.50
5.75
1.94 CD3CN
OTf
O
N
O
S
2b
¹H NMR (400 MHz, CD₃CN)
5.00
1.00
7.02
1.01
1.84
2.11
4.08
9.0 8.5 8.0 7.5 7.0 6.5 6.0 5.5 5.0 4.5 4.0 3.5 3.0 2.5 2.0 1.5 1.0 0.5
f1 (ppm)

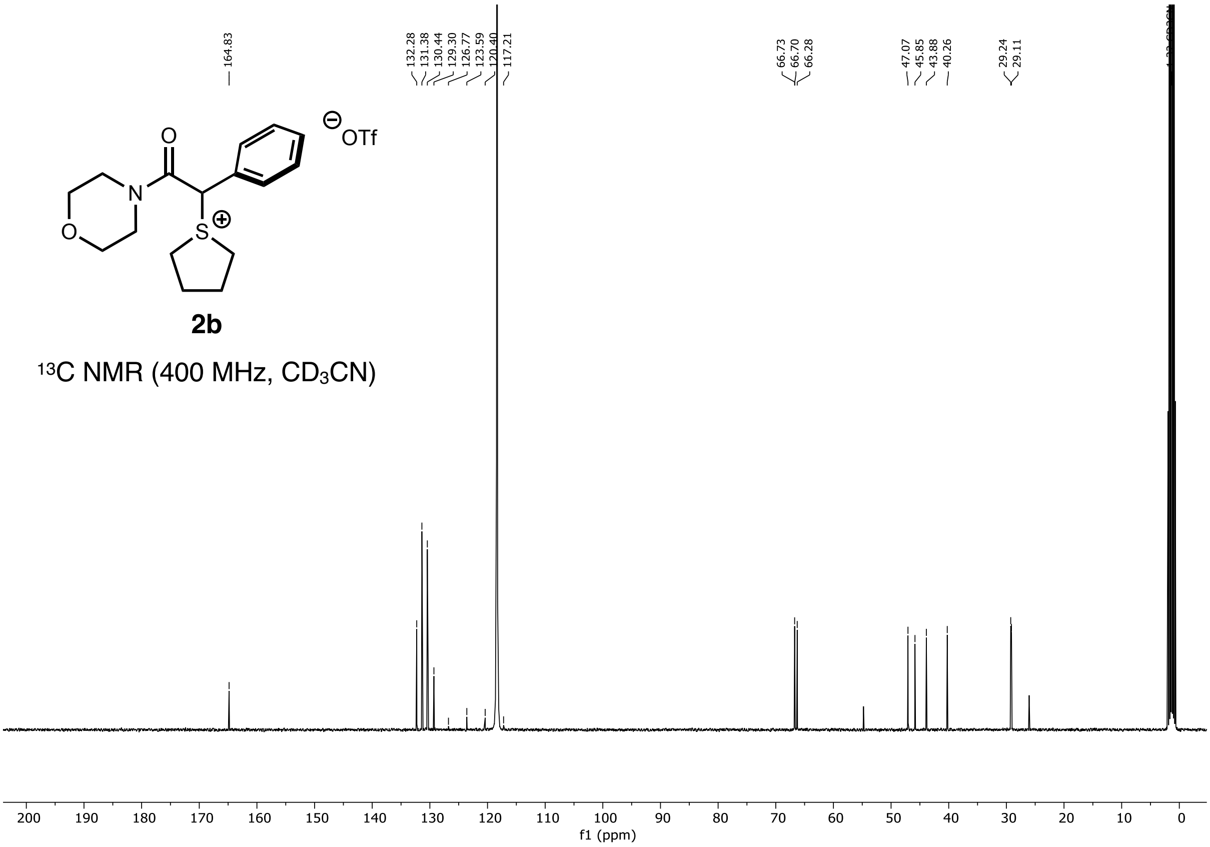

164.83
132.28 131.38 130.44 129.30 126.77 123.59 120.40 117.21
66.73 66.70 66.28
47.07 45.85 43.88 40.26
29.24 29.11
[illegible]
OTf
O
N
O
S
2b
¹³C NMR (400 MHz, CD₃CN)
200 190 180 170 160 150 140 130 120 110 100 90 80 70 60 50 40 30 20 10 0
f1 (ppm)

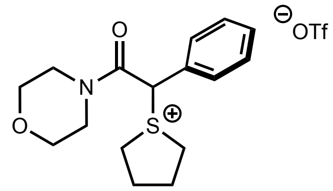
O
N
O
S
OTf

2b

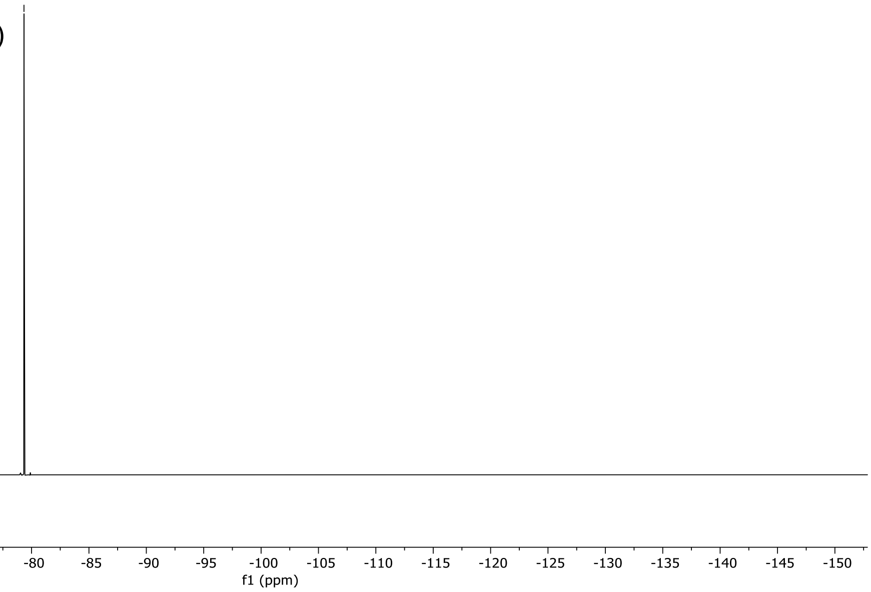
19F NMR (376 MHz, CD3CN)
-79.34
-50
-55
-60
-65
-70
-75
-80
-85
-90
-95
-100
-105
-110
-115
-120
-125
-130
-135
-140
-145
-150
f1 (ppm)

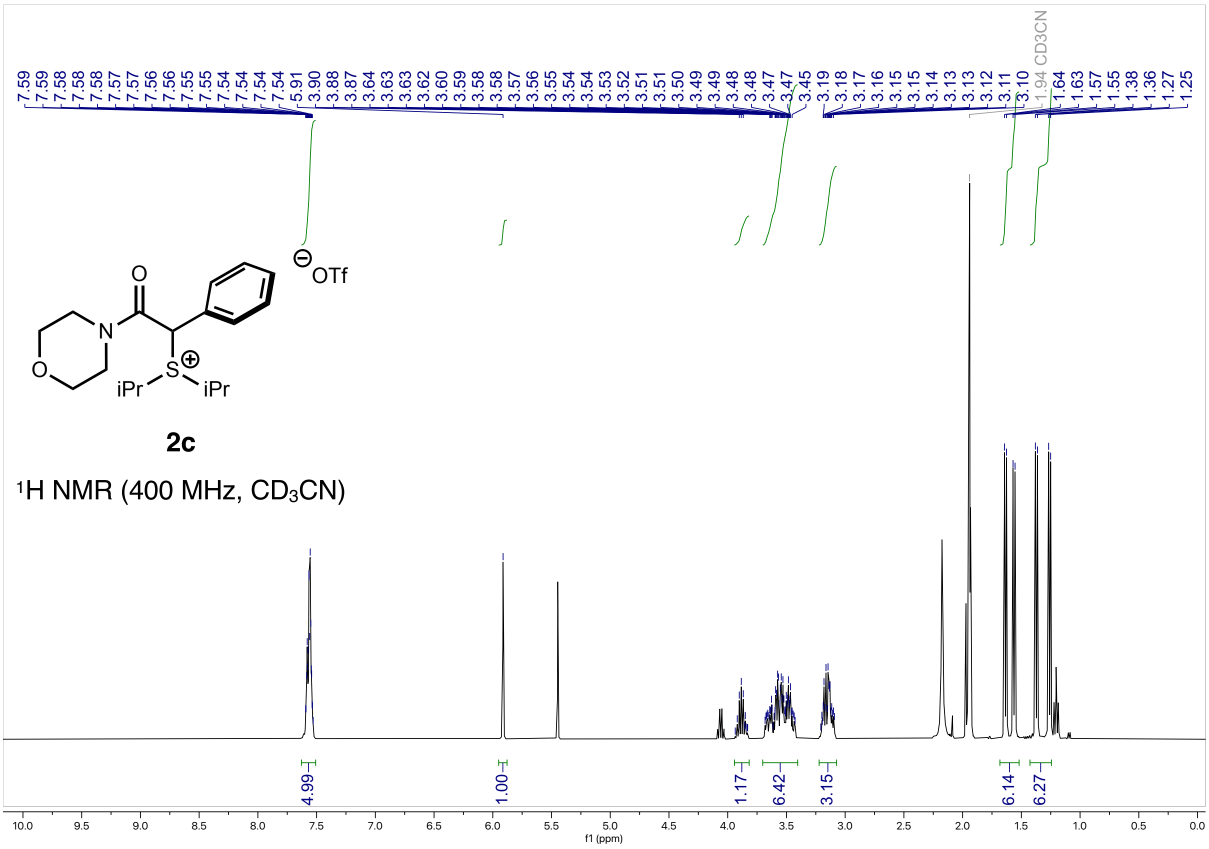
7.59 7.59 7.58 7.58 7.58 7.57 7.57 7.56 7.56 7.55 7.55 7.54 7.54 7.54 5.91 3.90 3.88 3.87 3.64 3.63 3.63 3.62 3.60 3.59 3.58 3.58 3.57 3.56 3.55 3.54 3.54 3.53 3.52 3.51 3.51 3.50 3.49 3.49 3.48 3.48 3.47 3.47 3.45 3.19 3.18 3.17 3.16 3.15 3.15 3.14 3.13 3.13 3.12 3.11 3.10 1.94 CD3CN 1.64 1.63 1.57 1.55 1.38 1.36 1.27 1.25
OTf
O
N
O
S
iPr
iPr
2c
¹H NMR (400 MHz, CD₃CN)
4.99
1.00
1.17
6.42
3.15
6.14
6.27
10.0 9.5 9.0 8.5 8.0 7.5 7.0 6.5 6.0 5.5 5.0 4.5 4.0 3.5 3.0 2.5 2.0 1.5 1.0 0.5 0.0
f1 (ppm)

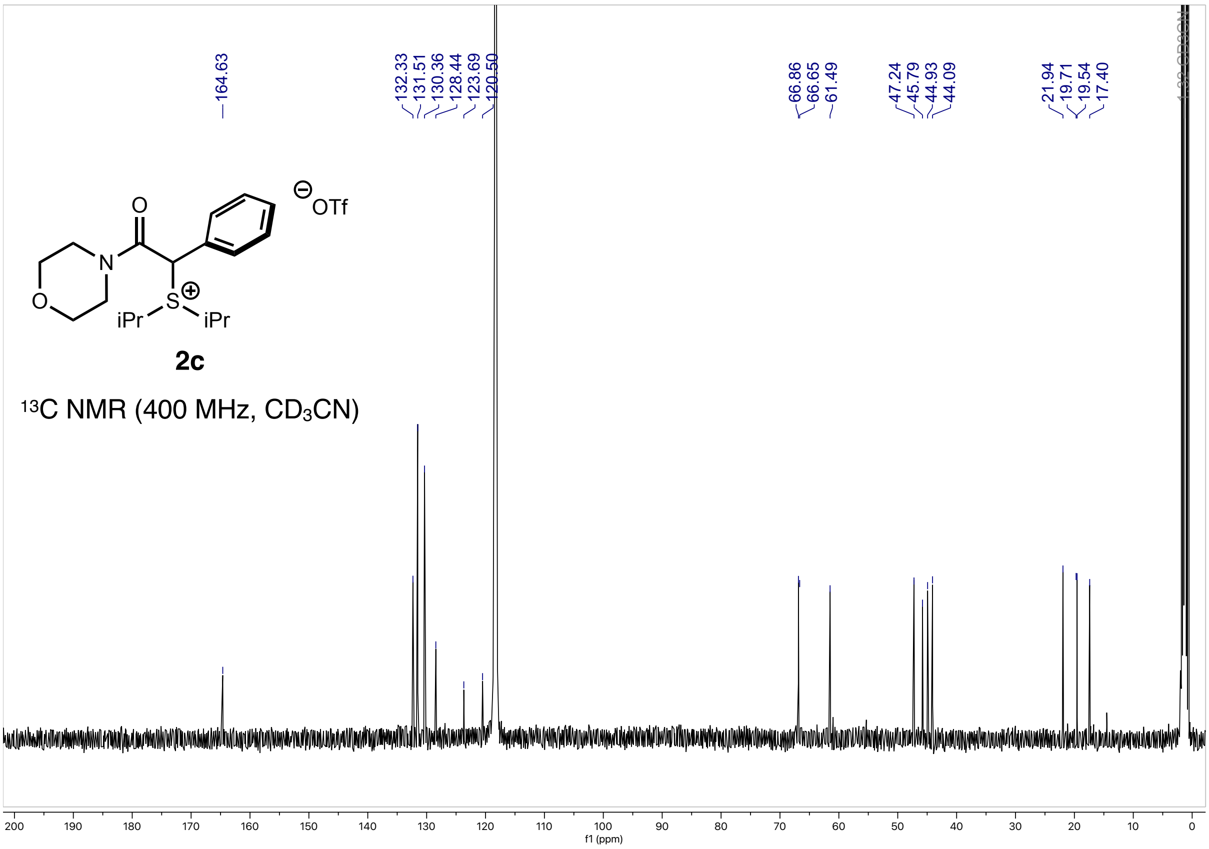
164.63
132.33 131.51 130.36 128.44 123.69 120.50
66.86 66.65 61.49
47.24 45.79 44.93 44.09
21.94 19.71 19.54 17.40
OTf
O
N
O
S
iPr
iPr
2c
¹³C NMR (400 MHz, CD₃CN)
200 190 180 170 160 150 140 130 120 110 100 90 80 70 60 50 40 30 20 10 0
f1 (ppm)

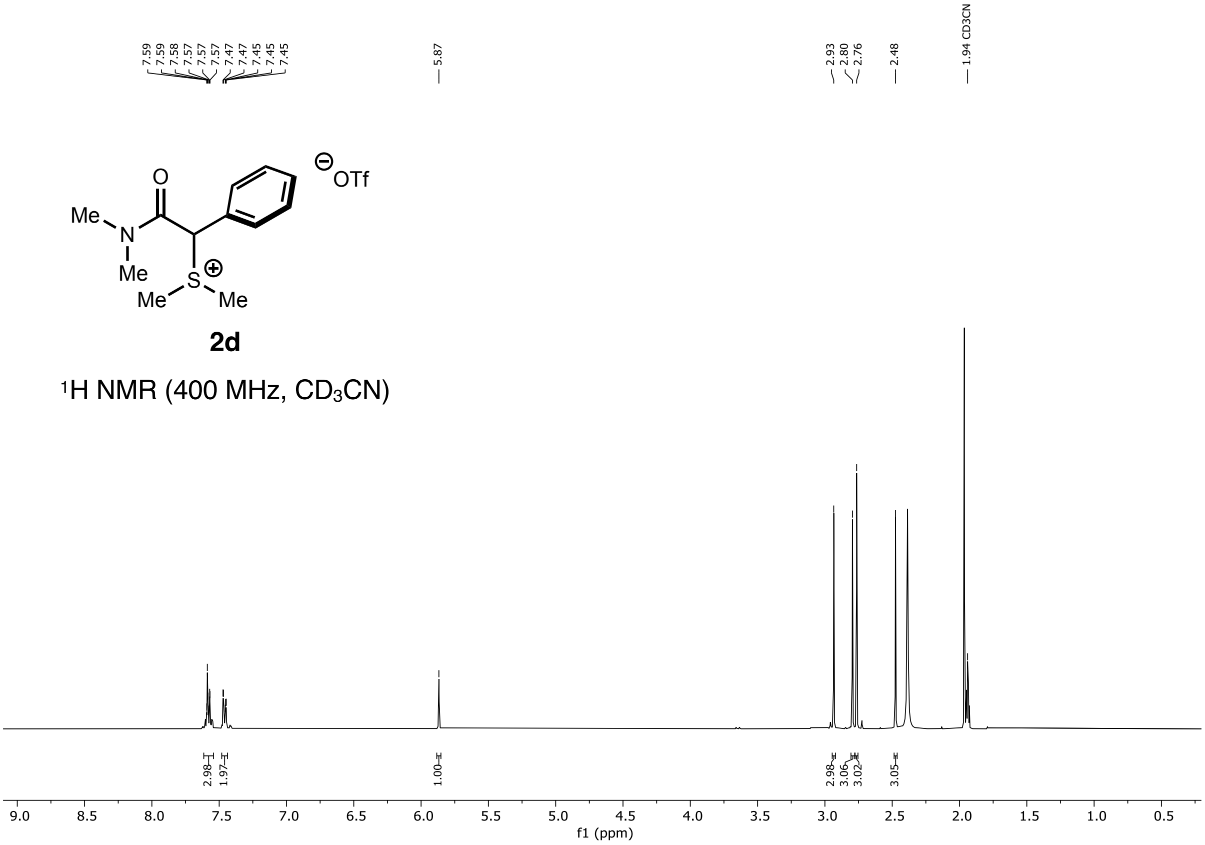

2d

^{1}H NMR (400 MHz, CD_3CN)

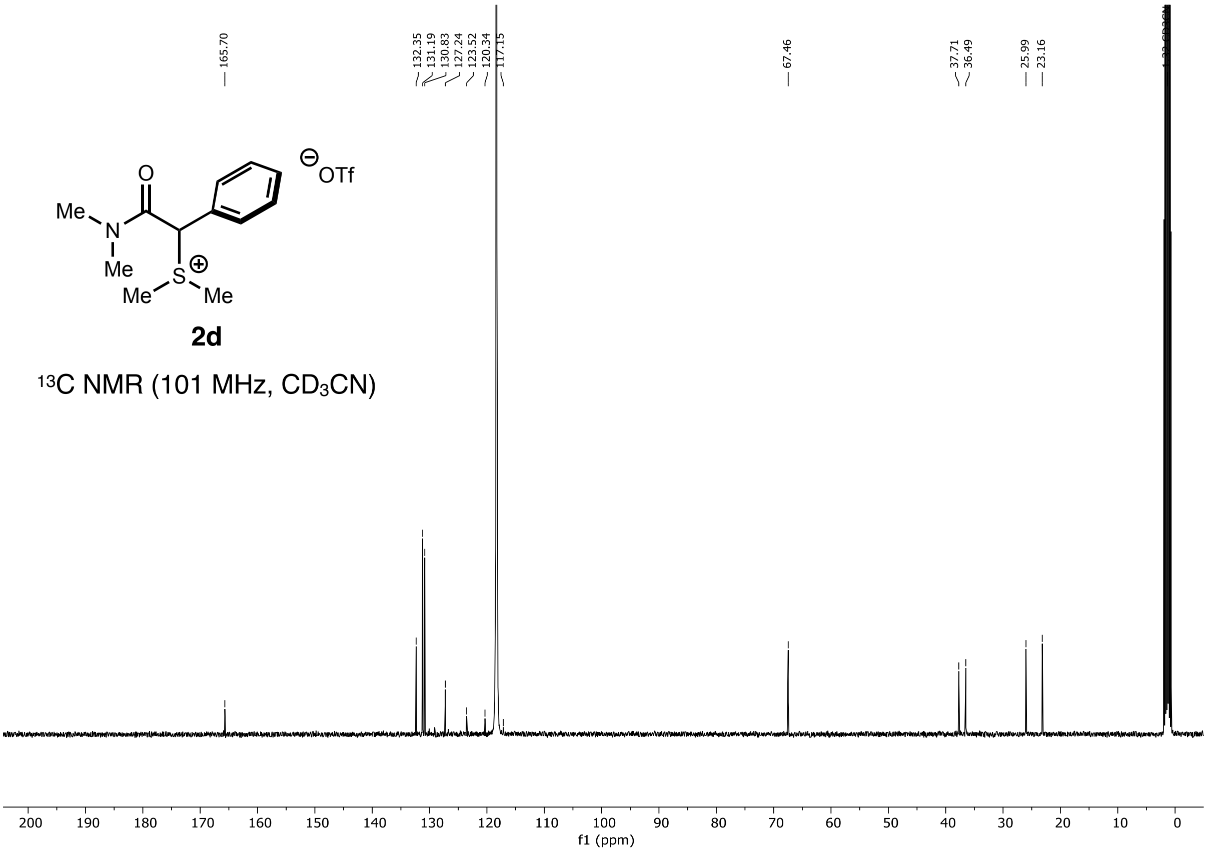

2d

^{13}C NMR (101 MHz, CD_3CN)

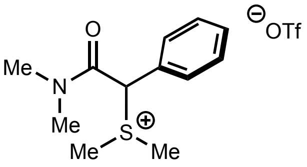

2d

^{19}F NMR (101 MHz, CD_3CN)

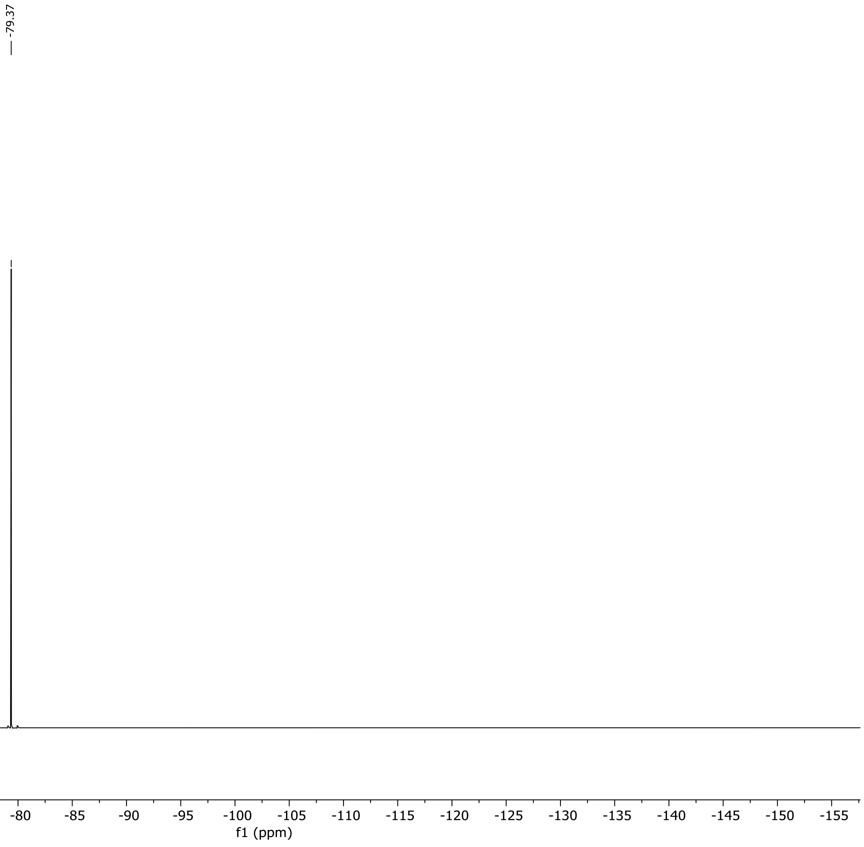

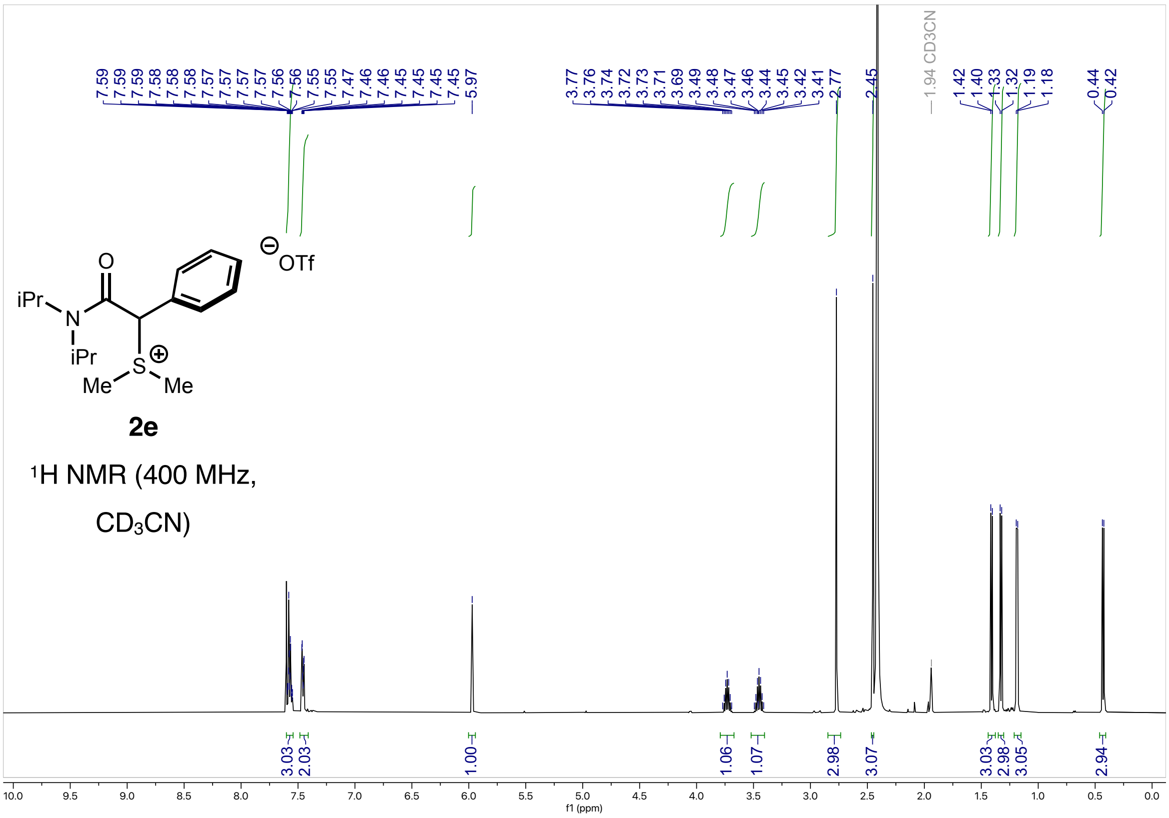

7.59 7.59 7.59 7.58 7.58 7.58 7.57 7.57 7.57 7.57 7.56 7.56 7.55 7.55 7.47 7.46 7.46 7.45 7.45 7.45 7.45 5.97
3.77 3.76 3.74 3.72 3.73 3.71 3.69 3.49 3.48 3.47 3.46 3.44 3.45 3.42 3.41 2.77 2.45
1.94 CD3CN
1.42 1.40 1.33 1.32 1.19 1.18 0.44 0.42
OTf
O
iPr
N
iPr
S
Me
Me
2e
1H NMR (400 MHz, CD3CN)
3.03 2.03 1.00 1.06 1.07 2.98 3.07 3.03 2.98 3.05 2.94
10.0 9.5 9.0 8.5 8.0 7.5 7.0 6.5 6.0 5.5 5.0 4.5 4.0 3.5 3.0 2.5 2.0 1.5 1.0 0.5 0.0
f1 (ppm)

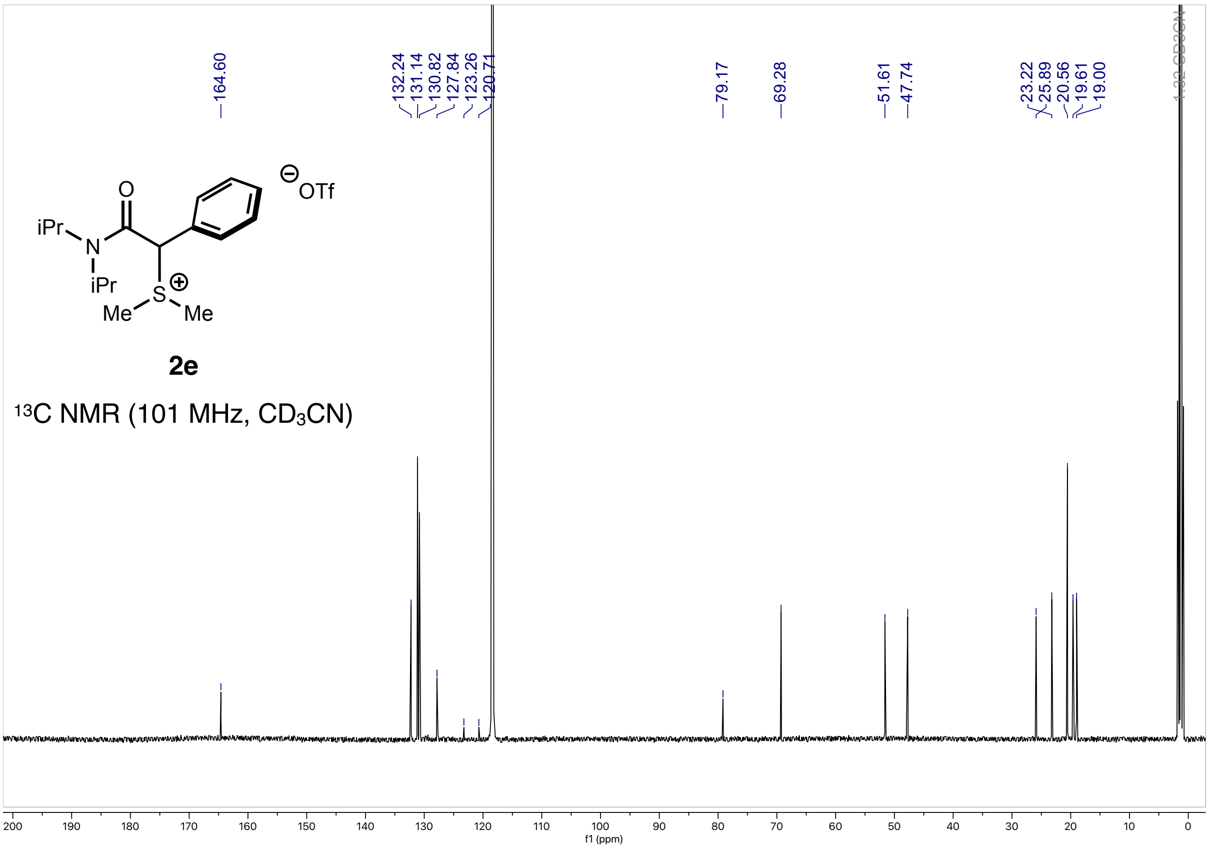

164.60
132.24 131.14 130.82 127.84 123.26 120.71
79.17 69.28 51.61 47.74
23.22 25.89 20.56 19.61 19.00
1.32 CD3CN
OTf
O
iPr
N
iPr
S
Me
Me
2e
13C NMR (101 MHz, CD3CN)
200 190 180 170 160 150 140 130 120 110 100 90 80 70 60 50 40 30 20 10 0
f1 (ppm)

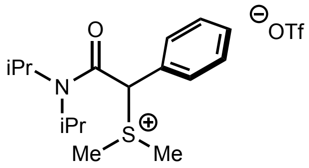

2e

^{19}F NMR (101 MHz, CD_3CN)

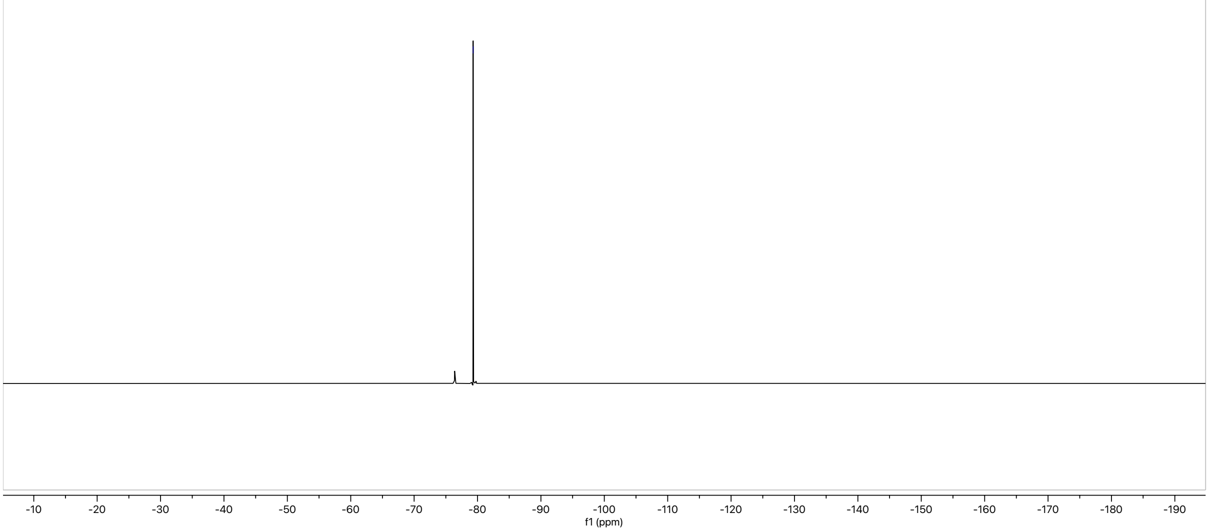

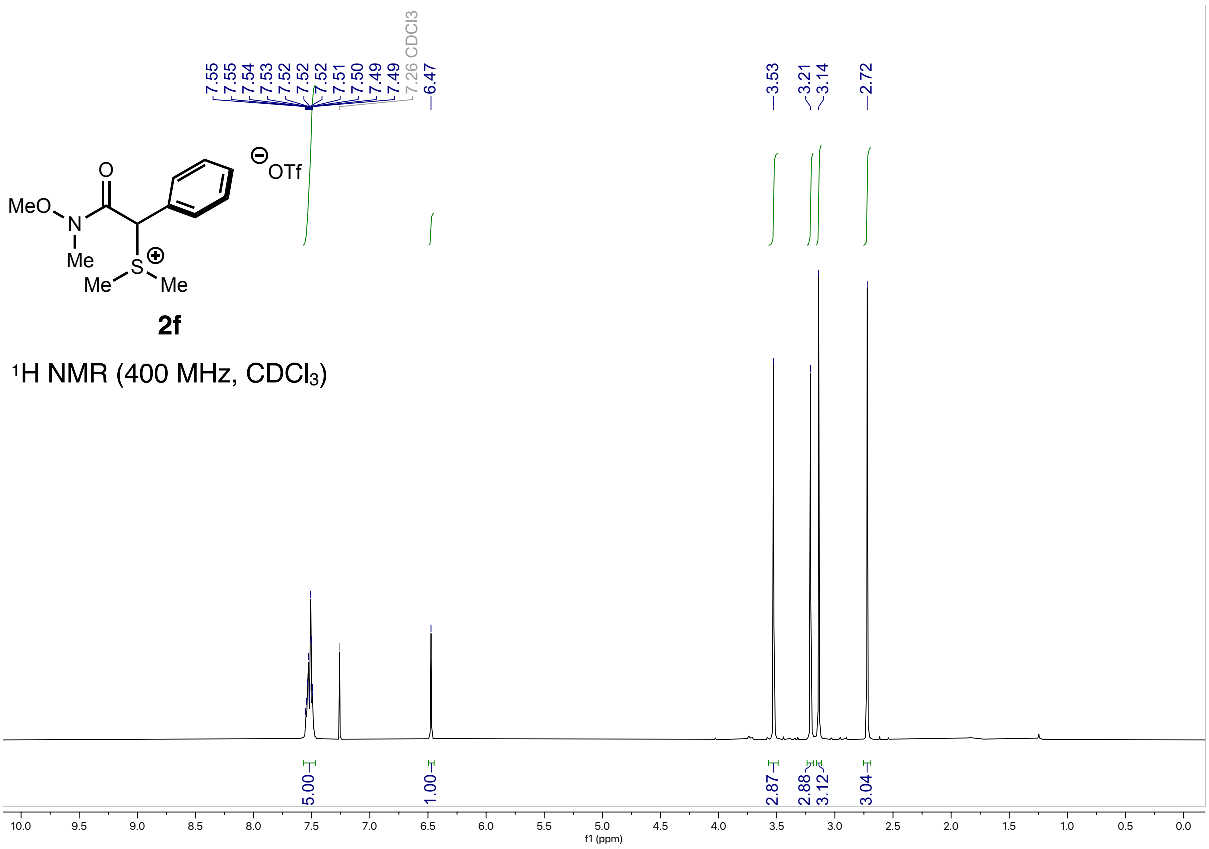
7.55
7.55
7.54
7.53
7.52
7.52
7.52
7.51
7.50
7.49
7.49
7.26 CDCl3
6.47
3.53
3.21
3.14
2.72
MeO
N
Me
O
S
Me
Me
OTf
2f
^1H NMR (400 MHz, $CDCl_3$)
5.00
1.00
2.87
2.88
3.12
3.04
f1 (ppm)

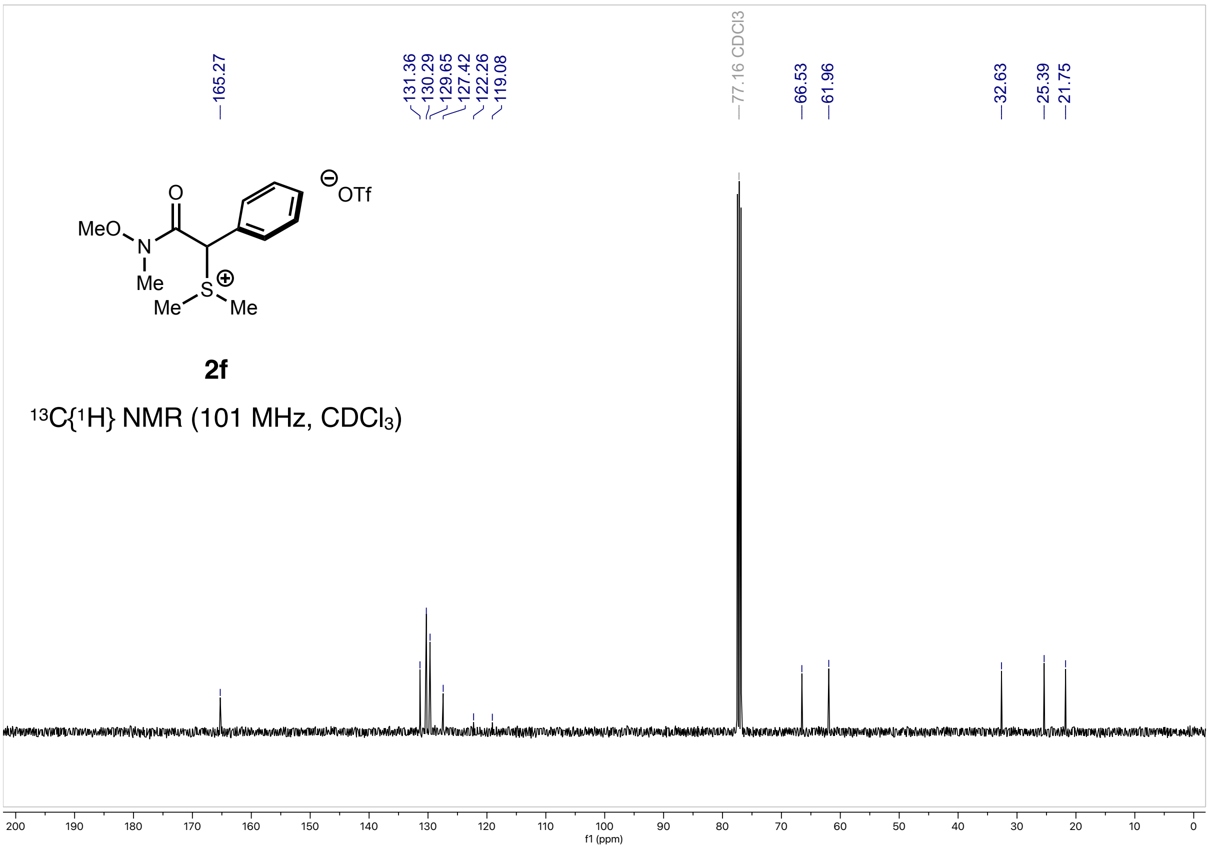
165.27
131.36
130.29
129.65
127.42
122.26
119.08
77.16 CDCl3
66.53
61.96
32.63
25.39
21.75
MeO
N
Me
O
S
Me
Me
OTf
2f
$^{13}C\{^1H\}$ NMR (101 MHz, $CDCl_3$)
f1 (ppm)

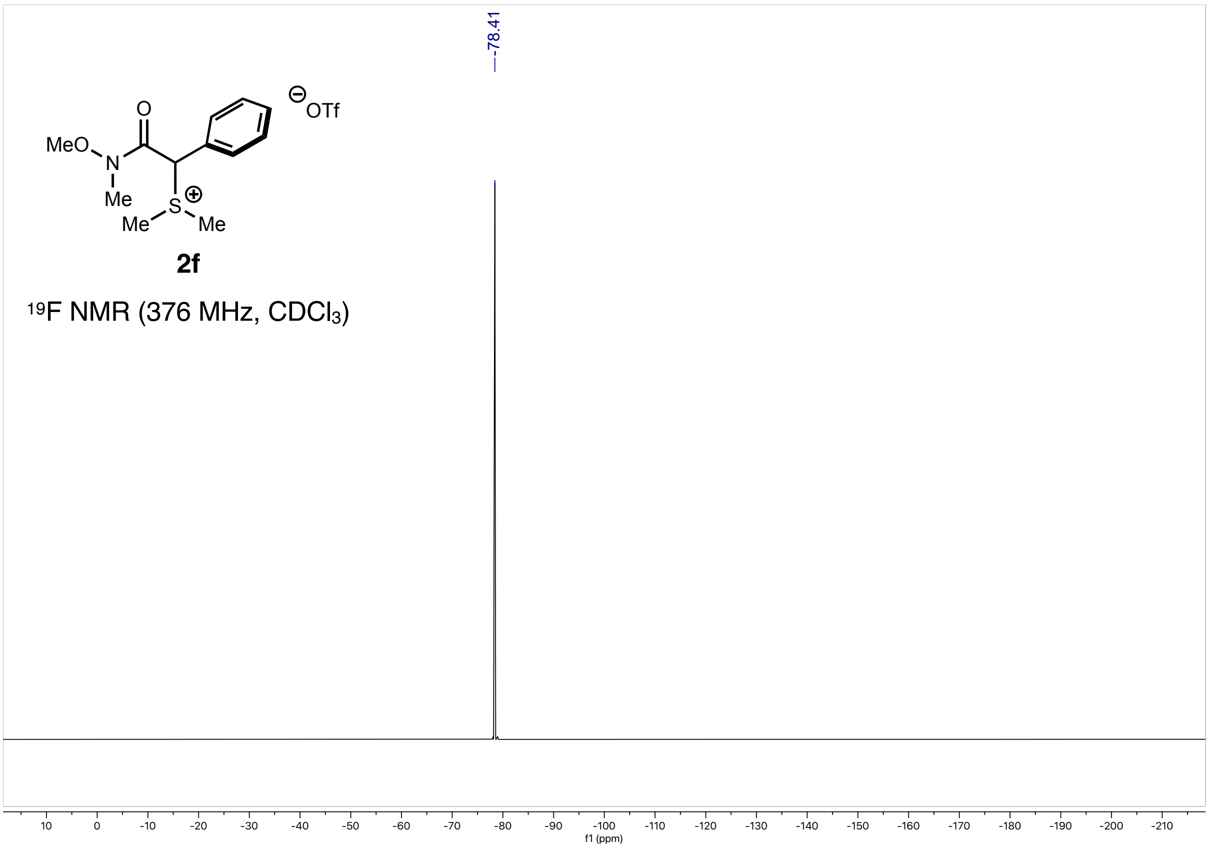

-78.41
MeO
N
Me
O
S
Me
Me
OTf
2f
19F NMR (376 MHz, CDCl3)
10
0
-10
-20
-30
-40
-50
-60
-70
-80
-90
-100
-110
-120
-130
-140
-150
-160
-170
-180
-190
-200
-210
f1 (ppm)

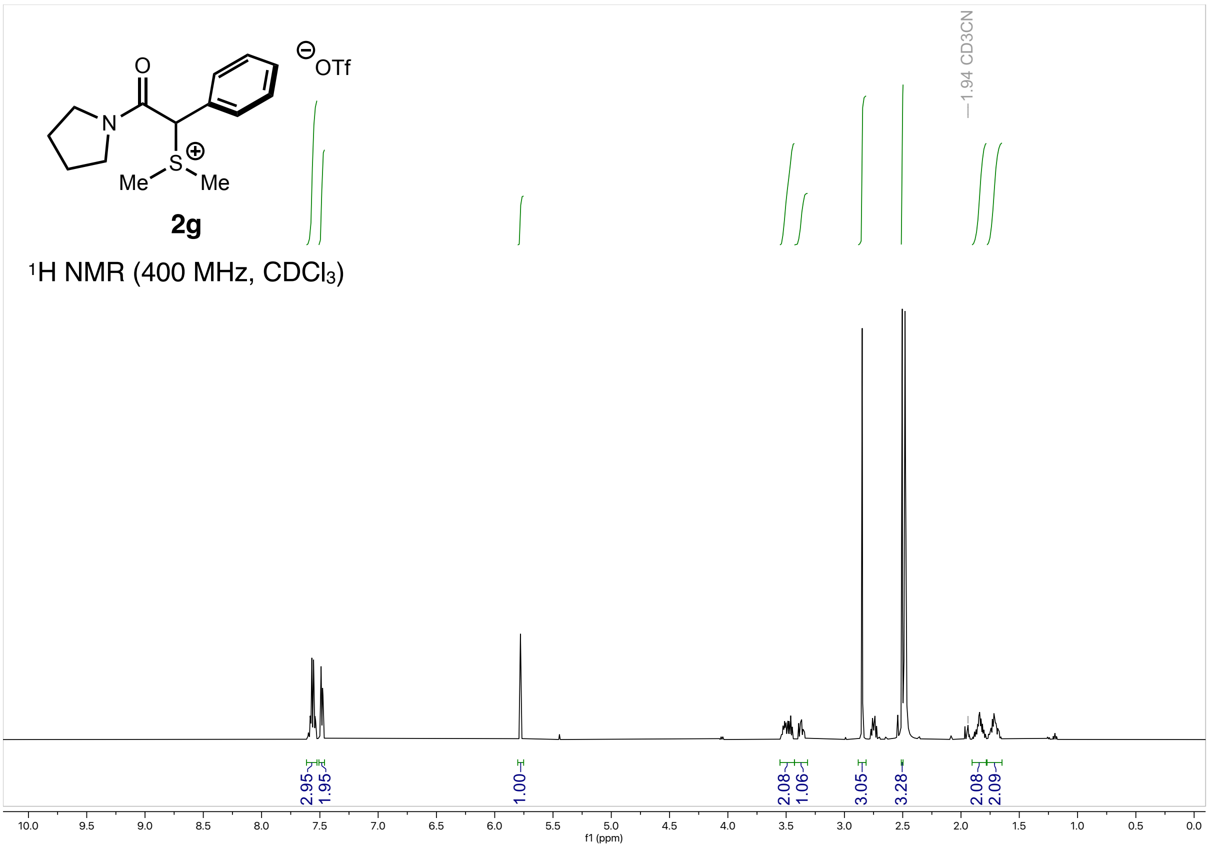

OTf
O
N
S
Me
Me
2g
1H NMR (400 MHz, CDCl3)
1.94 CD3CN
2.95
1.95
1.00
2.08
1.06
3.05
3.28
2.08
2.09
f1 (ppm)

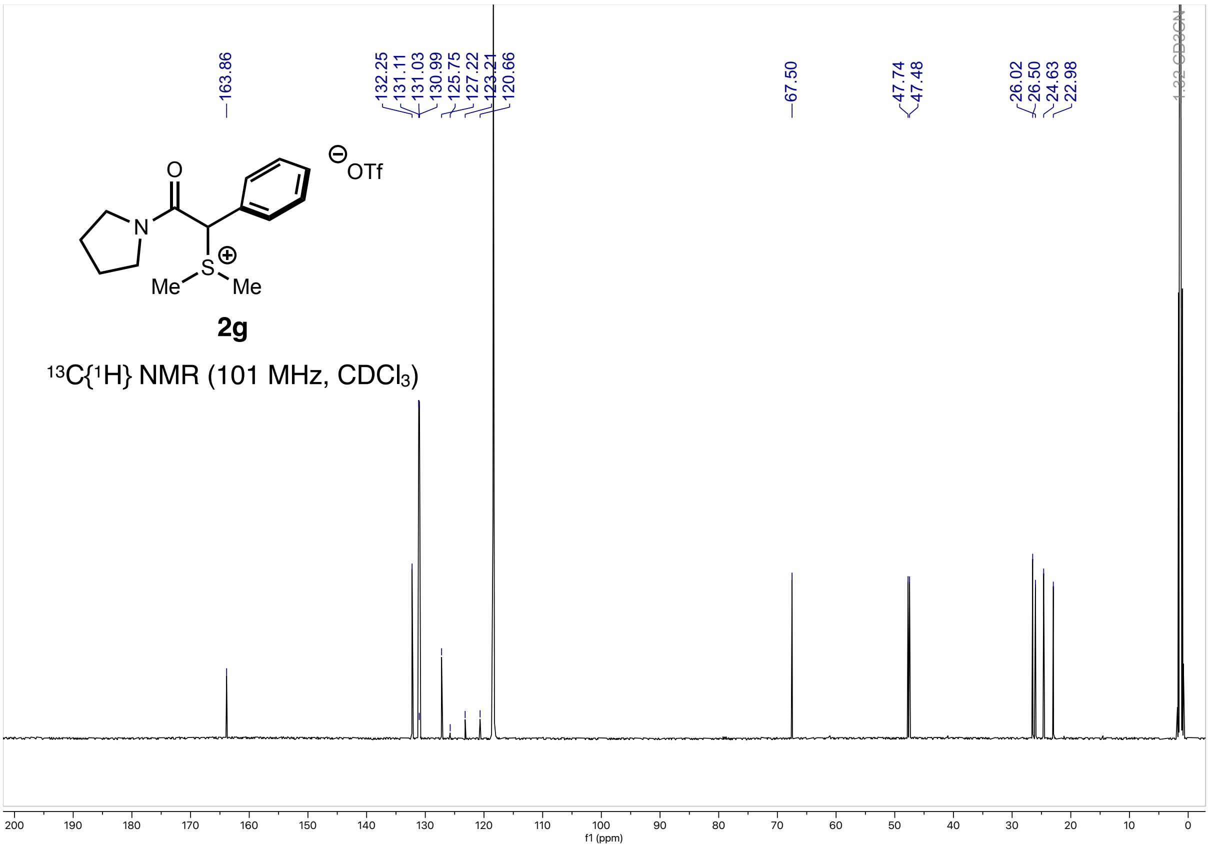

163.86
132.25
131.11
131.03
130.99
125.75
127.22
123.21
120.66
67.50
47.74
47.48
26.02
26.50
24.63
22.98
OTf
O
N
S
Me
Me
2g
13C{1H} NMR (101 MHz, CDCl3)
f1 (ppm)

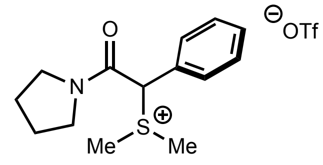

2f

^{19}F NMR (376 MHz, $CDCl_3$)

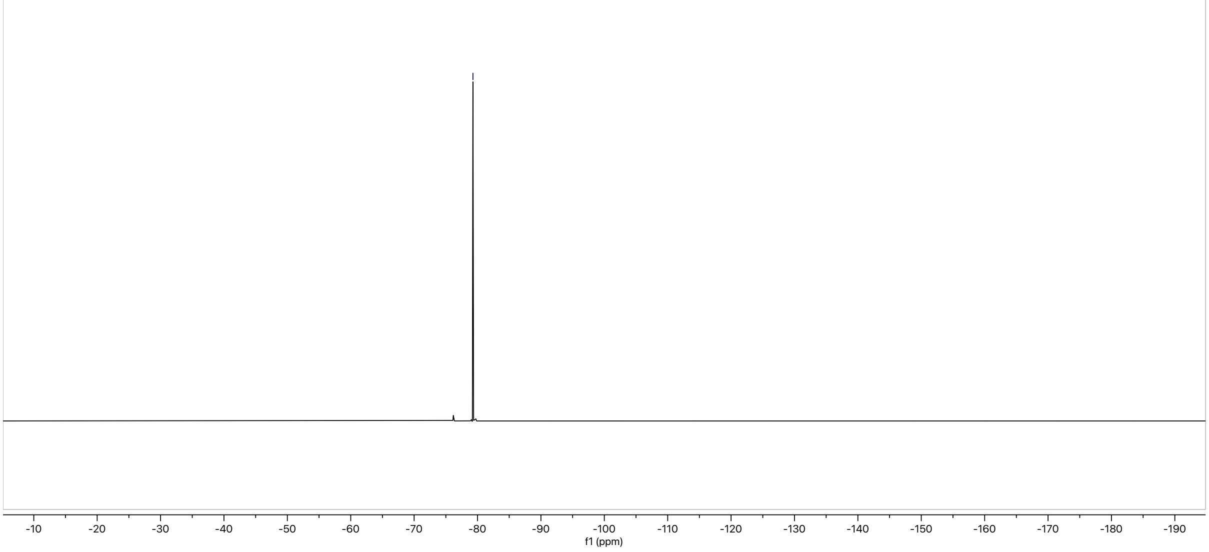

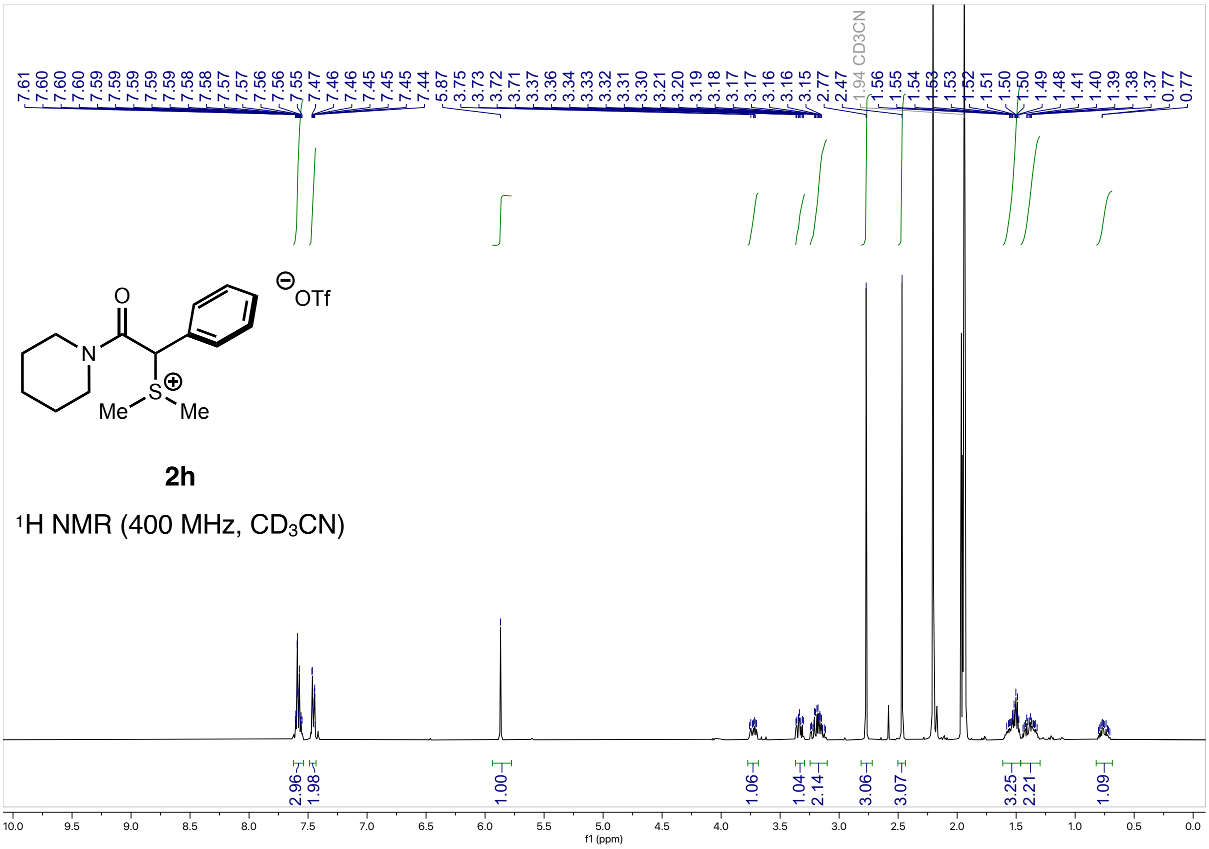
OTf
N
O
S
Me
Me
2h
^{1}H NMR (400 MHz, CD_3CN)
f1 (ppm)

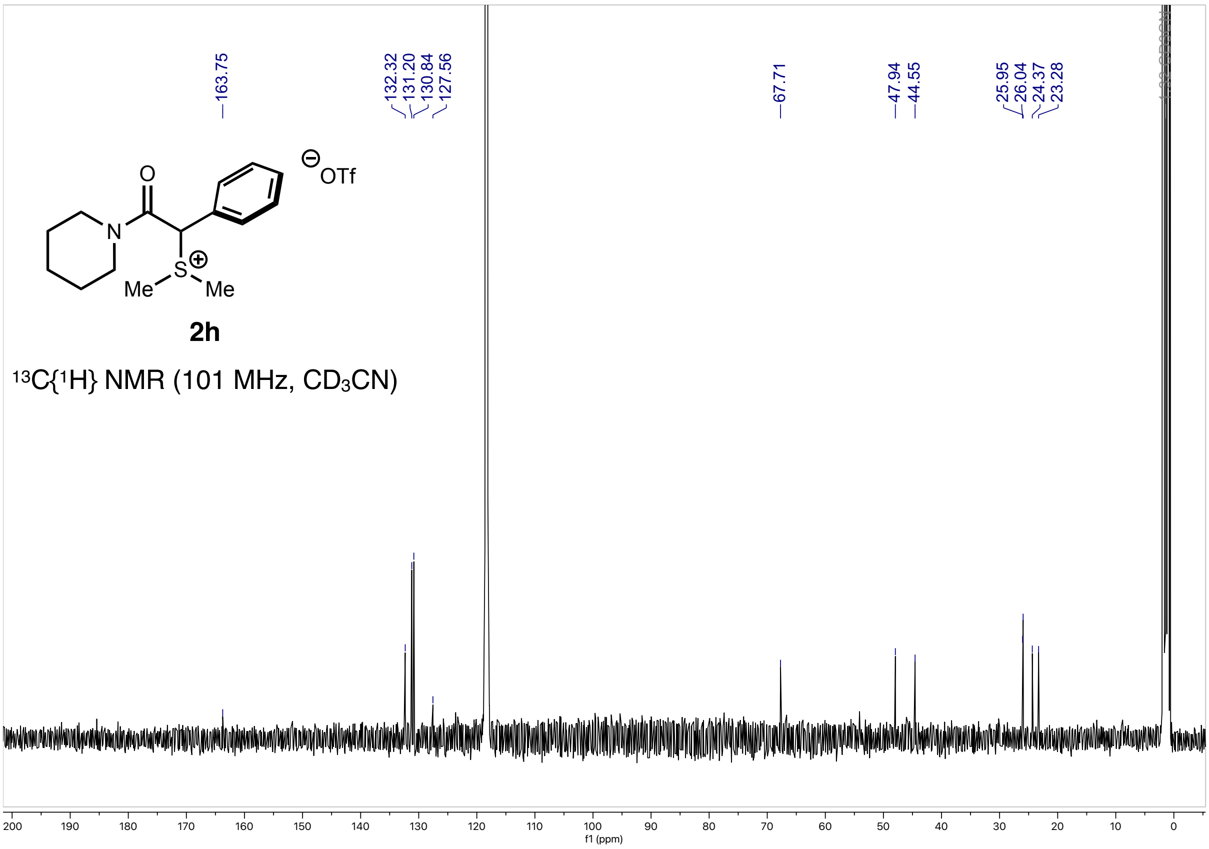
163.75
132.32
131.20
130.84
127.56
67.71
47.94
44.55
25.95
26.04
24.37
23.28
OTf
N
O
S
Me
Me
2h
$^{13}C\{^1H\}$ NMR (101 MHz, CD_3CN)
f1 (ppm)

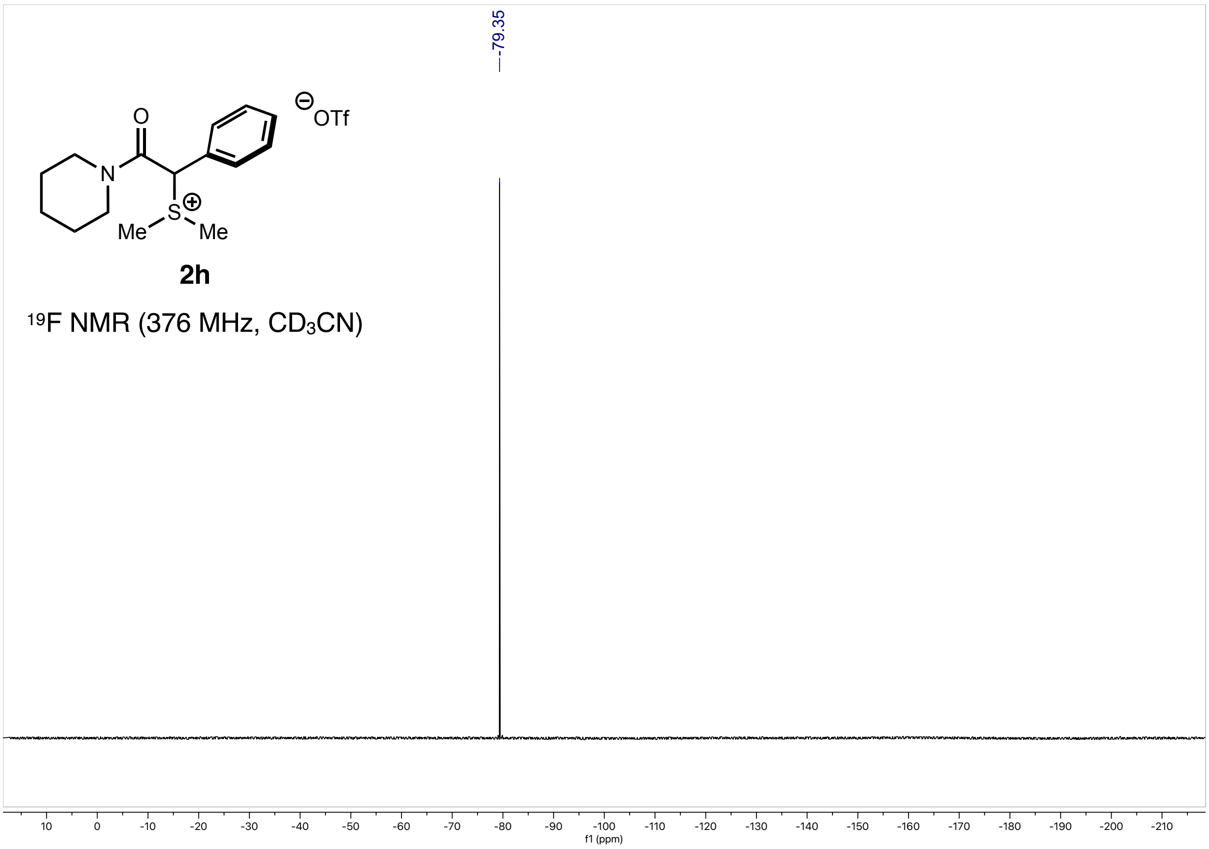

-79.35
O
N
S
Me
Me
OTf
2h
19F NMR (376 MHz, CD3CN)
10
0
-10
-20
-30
-40
-50
-60
-70
-80
-90
-100
-110
-120
-130
-140
-150
-160
-170
-180
-190
-200
-210
f1 (ppm)

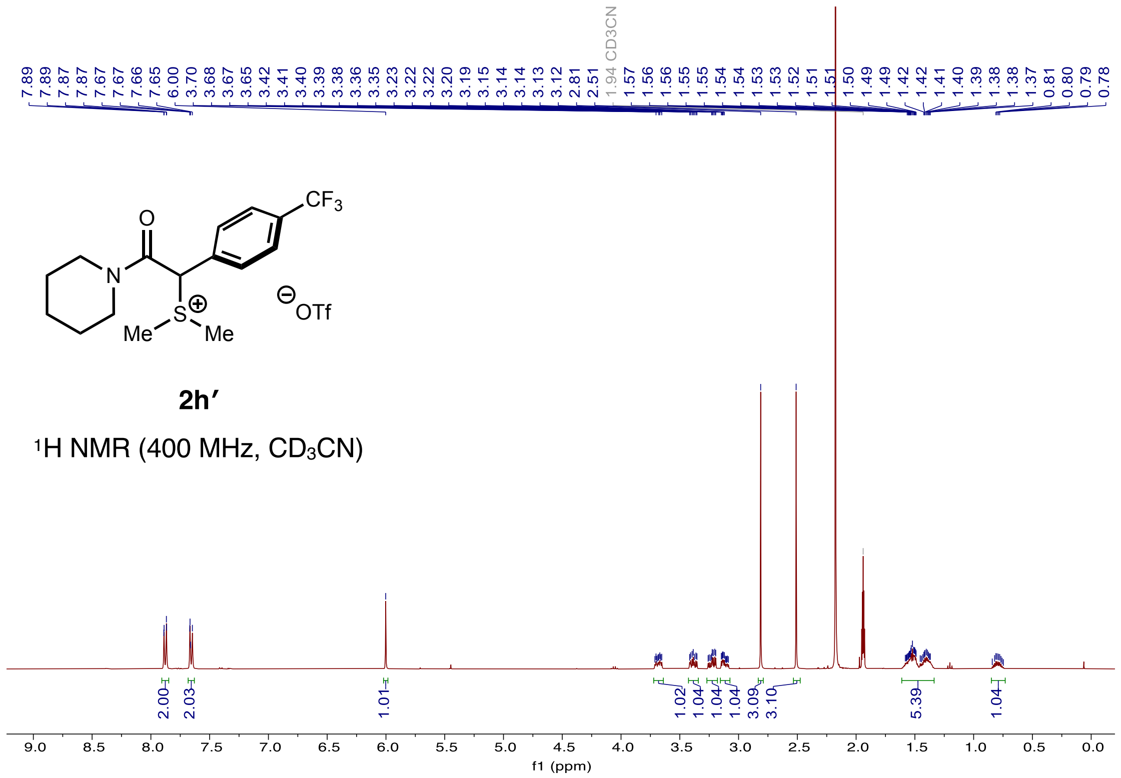

2h′

^{1}H NMR (400 MHz, CD_3CN)

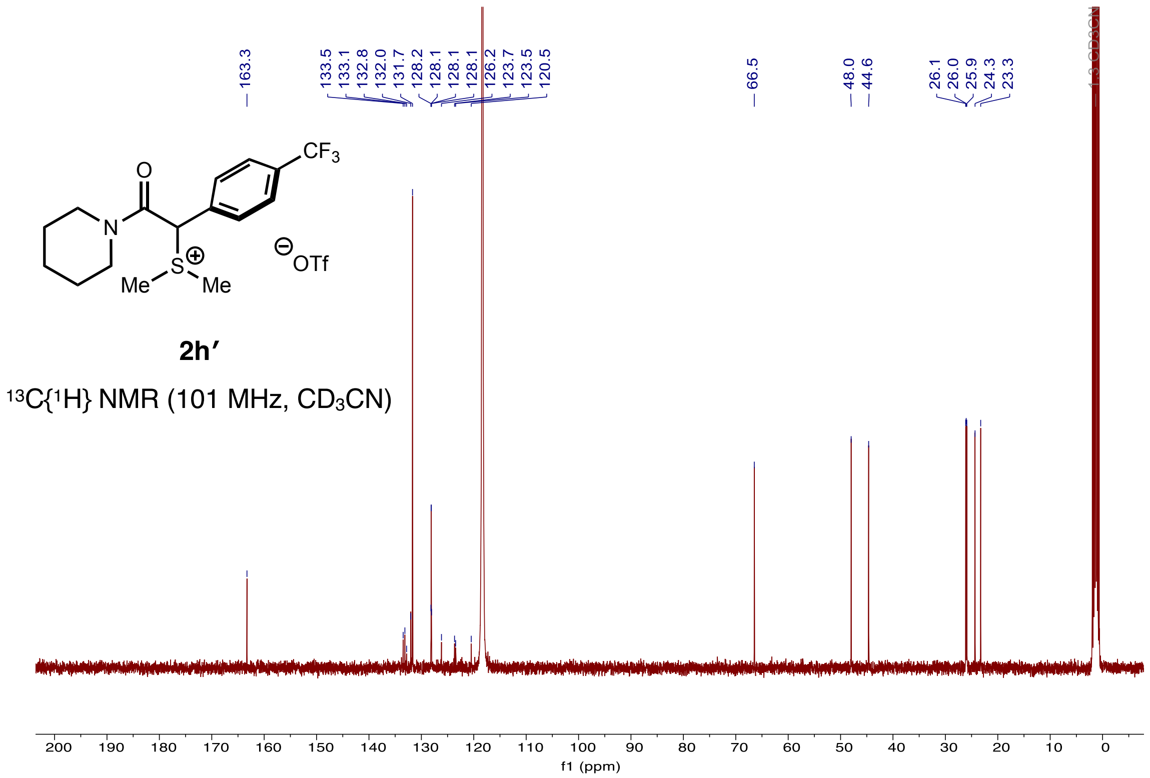

2h′

$^{13}C\{^{1}H\}$ NMR (101 MHz, CD_3CN)

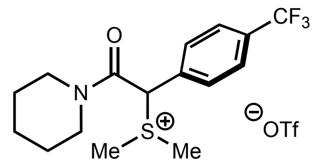

2h′

^{19}F NMR (376 MHz, CD_3CN)

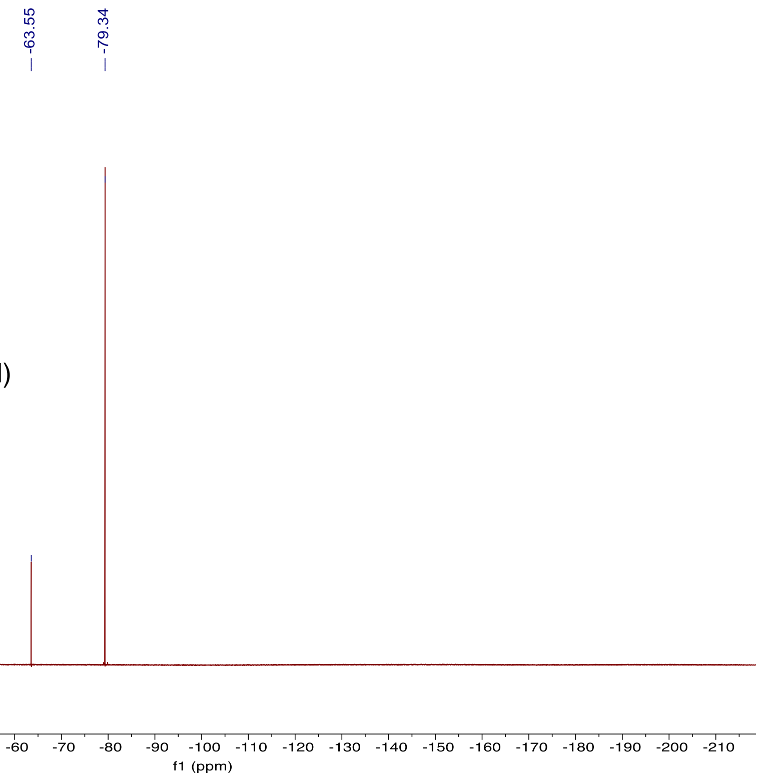

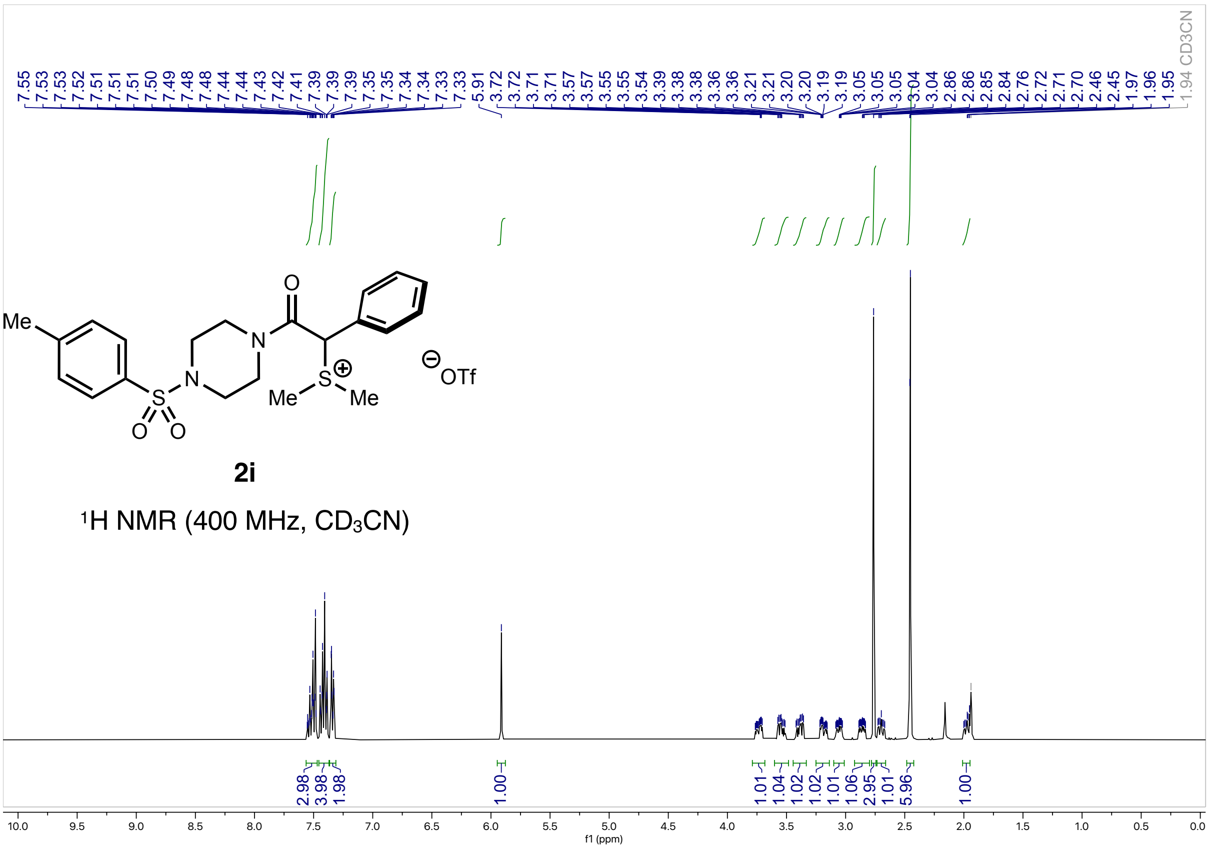

Me
N
N
S
O
O
O
S
Me
Me
OTf
2i
^{1}H NMR (400 MHz, CD$_3$CN)
f1 (ppm)

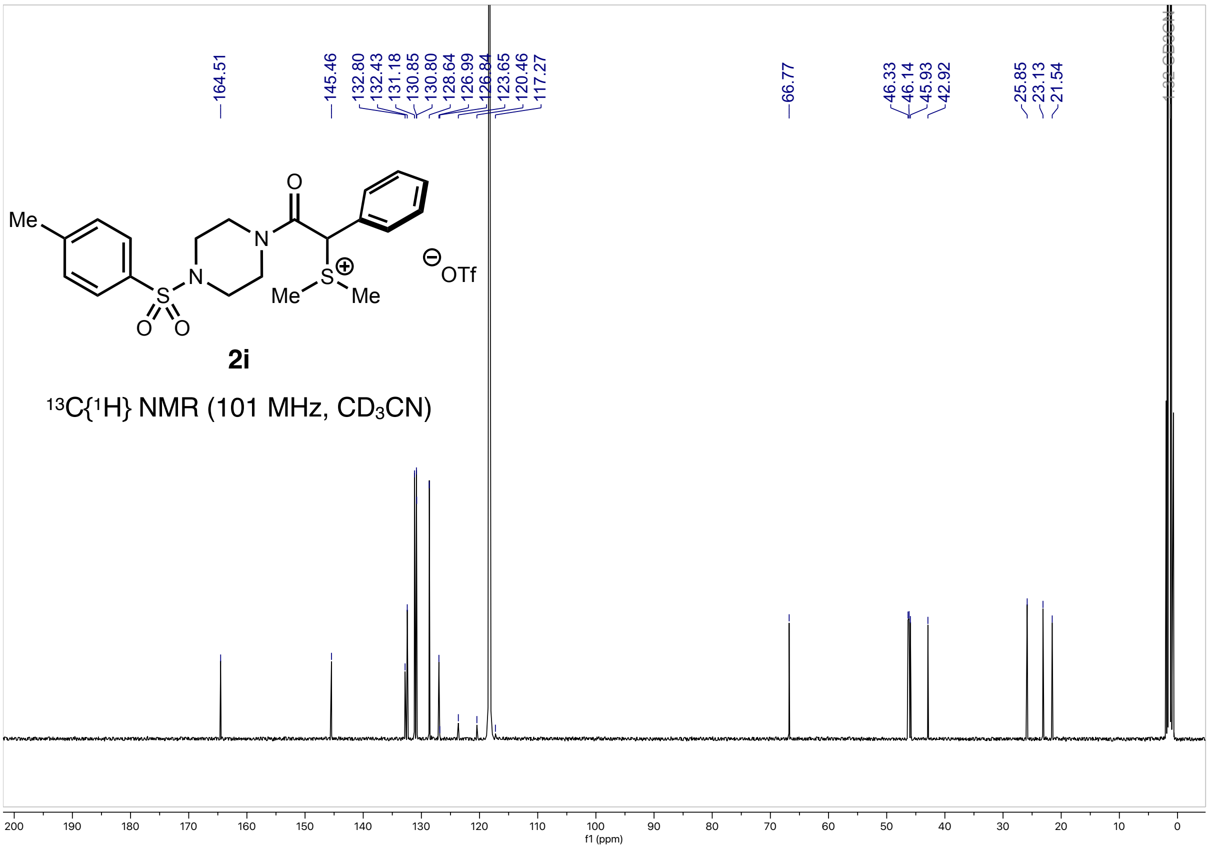

164.51
145.46
132.80
132.43
131.18
130.85
130.80
128.64
126.99
126.84
123.65
120.46
117.27
66.77
46.33
46.14
45.93
42.92
25.85
23.13
21.54
Me
N
N
S
O
O
O
S
Me
Me
OTf
2i
^{13}C{^{1}H} NMR (101 MHz, CD$_3$CN)
f1 (ppm)

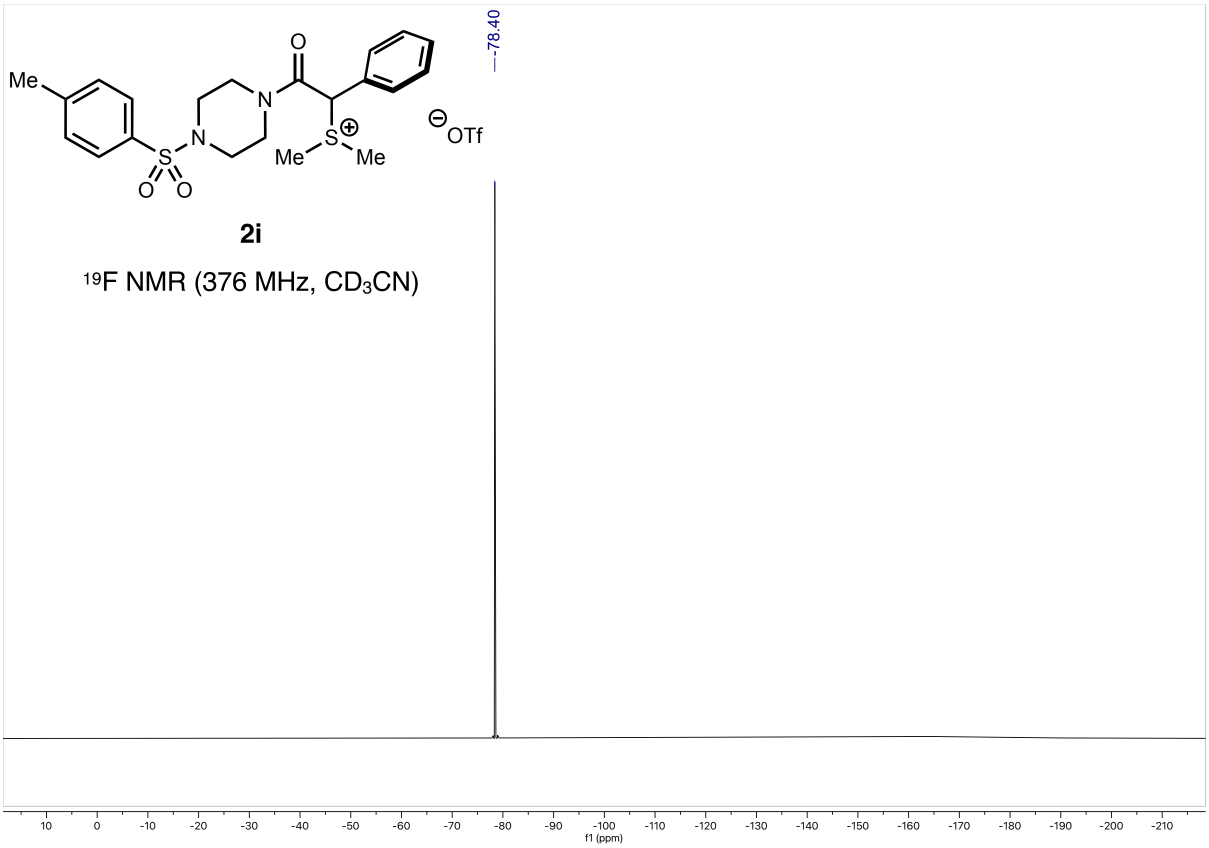

2i

^{19}F NMR (376 MHz, CD_3CN)

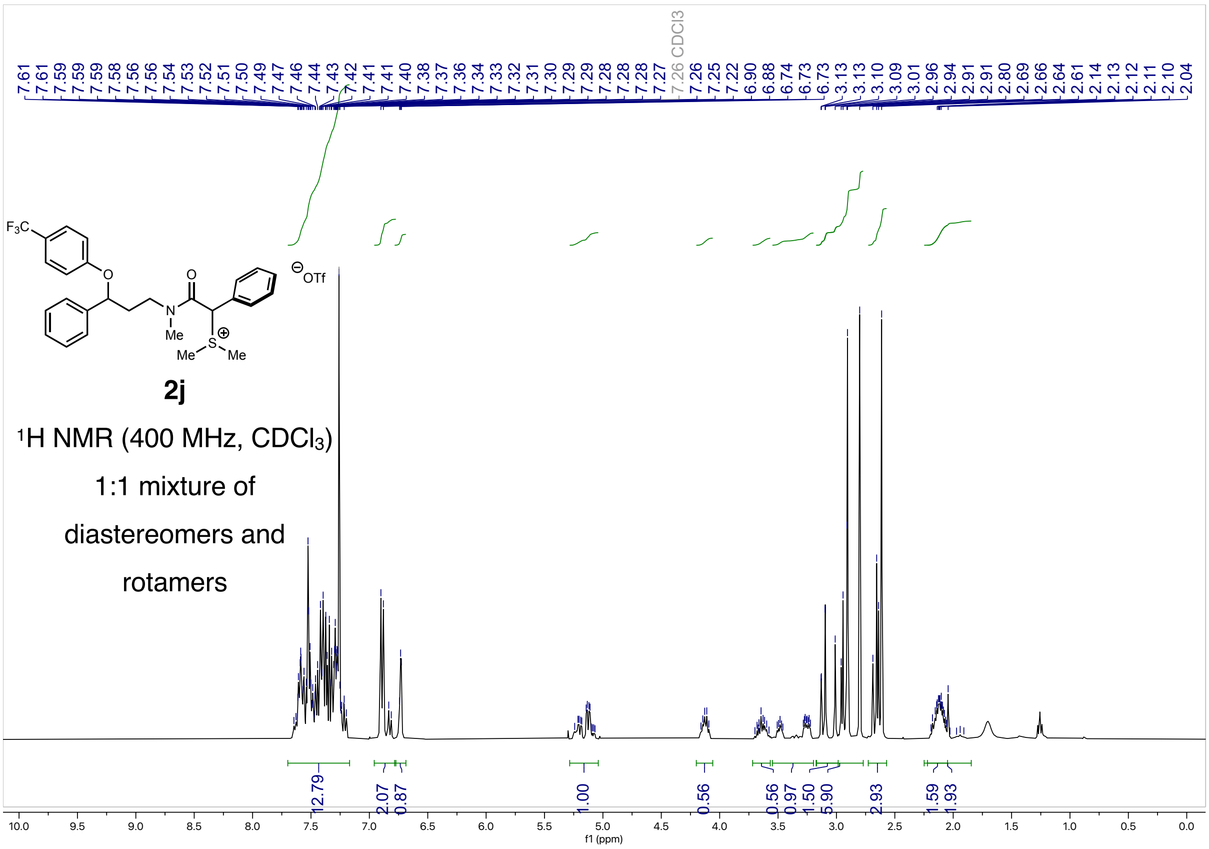

2j

^1H NMR (400 MHz, $CDCl_3$)

1:1 mixture of diastereomers and rotamers

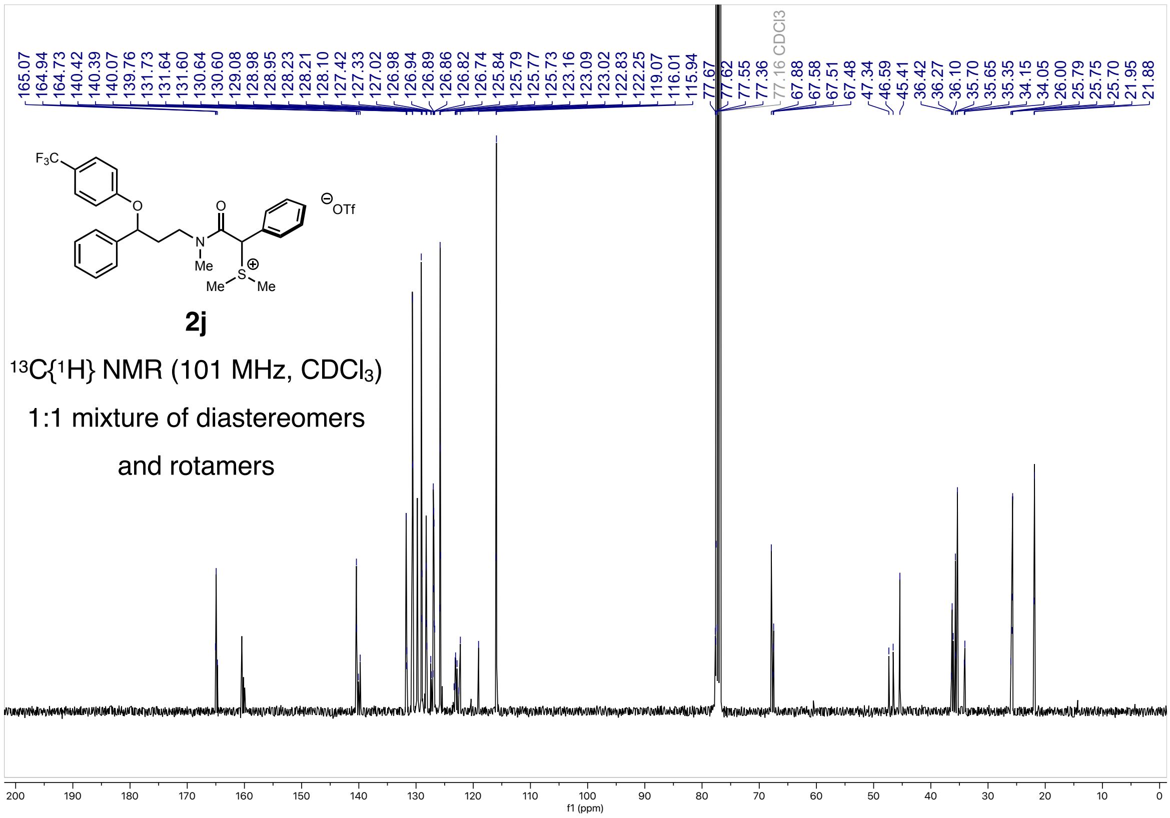

2j

$^{13}C\{^1H\}$ NMR (101 MHz, $CDCl_3$)

1:1 mixture of diastereomers and rotamers

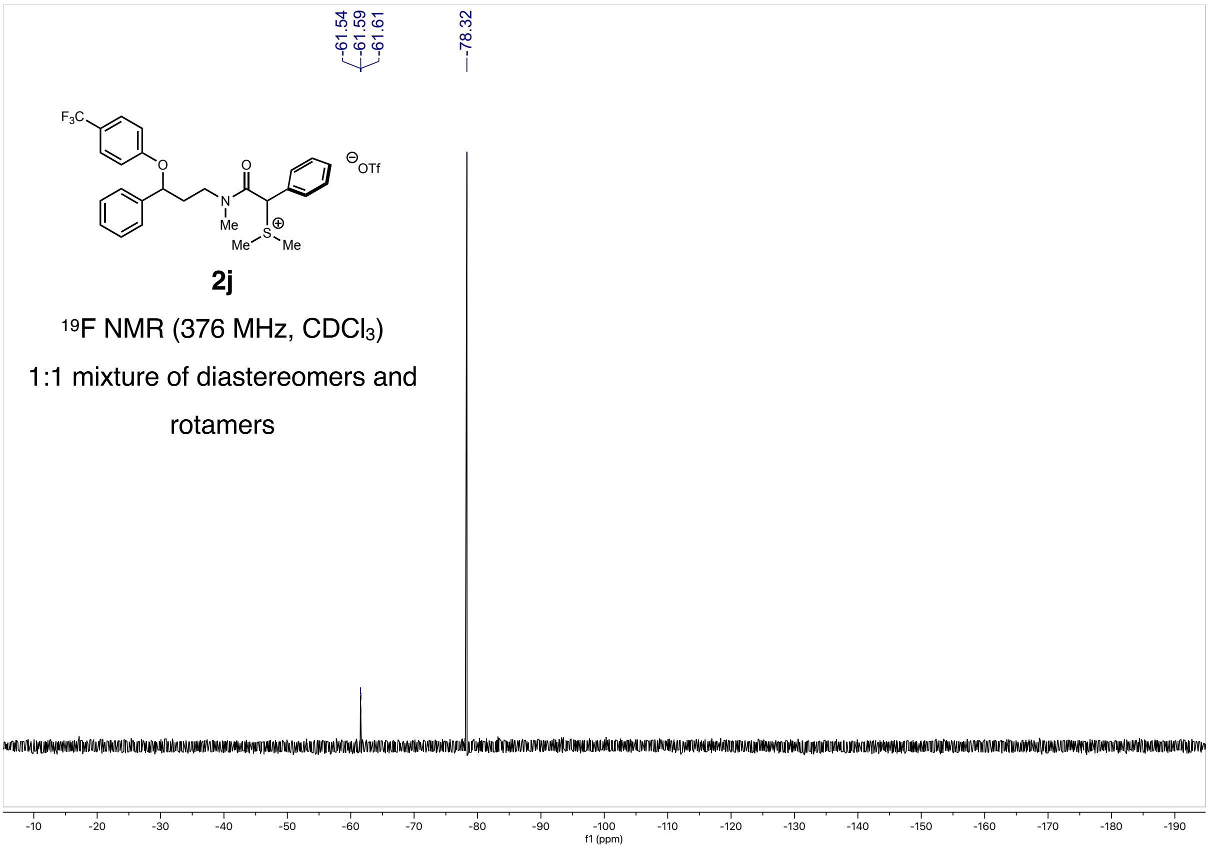
-61.54
-61.59
-61.61
-78.32
F3C
O
O
N
Me
S
Me
Me
OTf
2j
19F NMR (376 MHz, CDCl3)
1:1 mixture of diastereomers and
rotamers
-10
-20
-30
-40
-50
-60
-70
-80
-90
-100
-110
-120
-130
-140
-150
-160
-170
-180
-190
f1 (ppm)

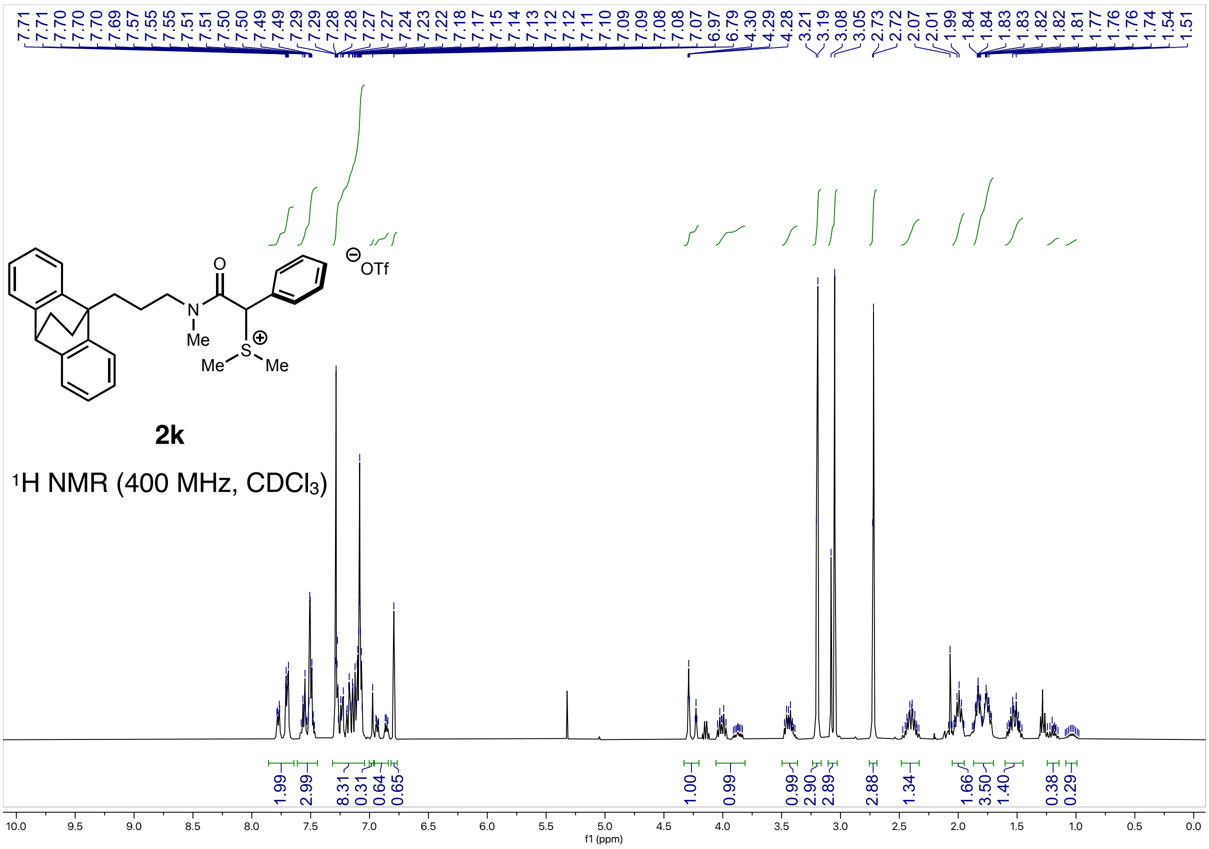

7.71 7.71 7.70 7.70 7.69 7.57 7.55 7.55 7.51 7.51 7.50 7.50 7.49 7.49 7.29 7.29 7.28 7.28 7.27 7.27 7.24 7.23 7.22 7.18 7.17 7.15 7.14 7.13 7.12 7.12 7.11 7.10 7.09 7.09 7.08 7.08 7.07 6.97 6.79 4.30 4.29 4.28 3.21 3.19 3.08 3.05 2.73 2.72 2.07 2.01 1.99 1.84 1.84 1.83 1.83 1.82 1.82 1.81 1.77 1.76 1.76 1.74 1.54 1.51
O
N
Me
S
Me
Me
OTf
2k
^{1}H NMR (400 MHz, $CDCl_3$)
1.99 2.99 8.31 0.31 0.64 0.65 1.00 0.99 0.99 2.90 2.89 2.88 1.34 1.66 3.50 1.40 0.38 0.29
10.0 9.5 9.0 8.5 8.0 7.5 7.0 6.5 6.0 5.5 5.0 4.5 4.0 3.5 3.0 2.5 2.0 1.5 1.0 0.5 0.0
f1 (ppm)

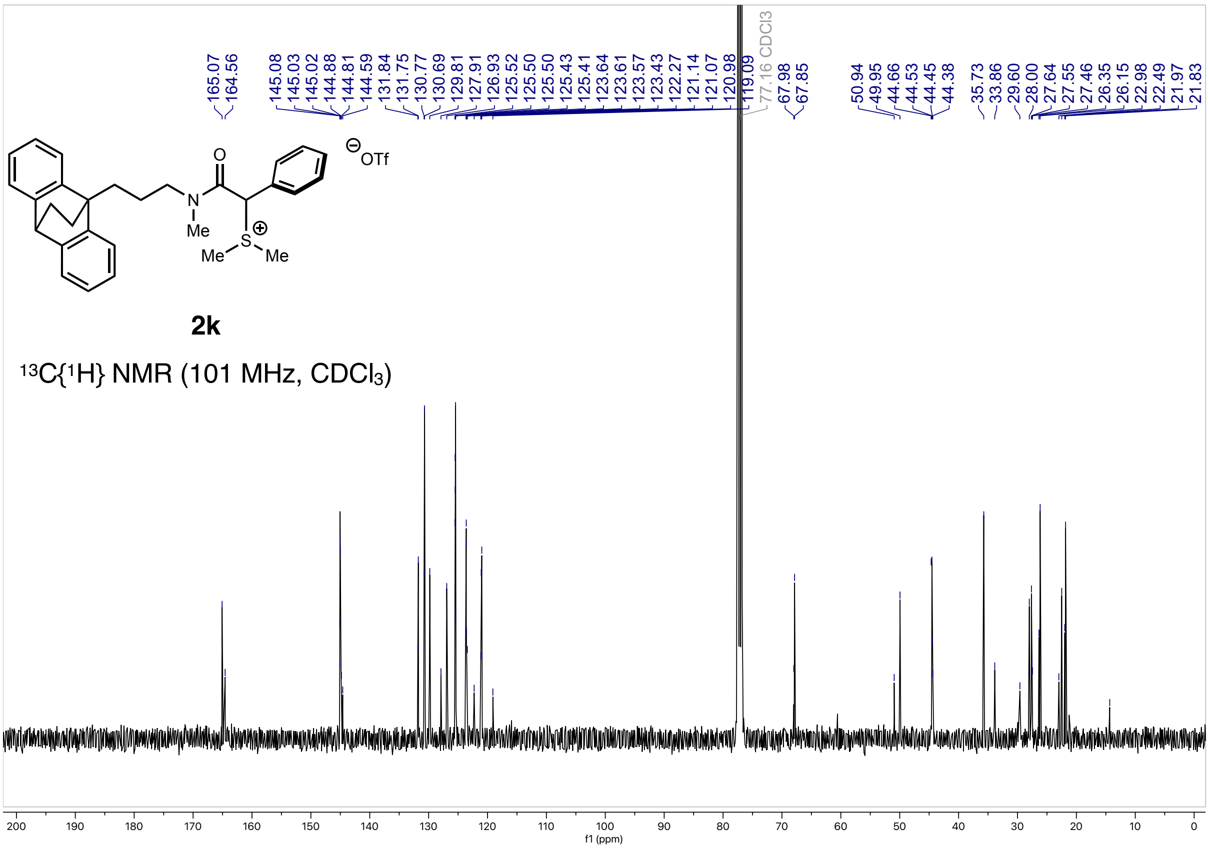

165.07 164.56 145.08 145.03 145.02 144.88 144.81 144.59 131.84 131.75 130.77 130.69 129.81 127.91 126.93 125.52 125.50 125.50 125.43 125.41 123.64 123.61 123.57 123.43 122.27 121.14 121.07 120.98 119.09
77.16 CDCl3
67.98 67.85 50.94 49.95 44.66 44.53 44.45 44.38 35.73 33.86 29.60 28.00 27.64 27.55 27.46 26.35 26.15 22.98 22.49 21.97 21.83
O
N
Me
S
Me
Me
OTf
2k
^{13}C{^{1}H} NMR (101 MHz, $CDCl_3$)
200 190 180 170 160 150 140 130 120 110 100 90 80 70 60 50 40 30 20 10 0
f1 (ppm)

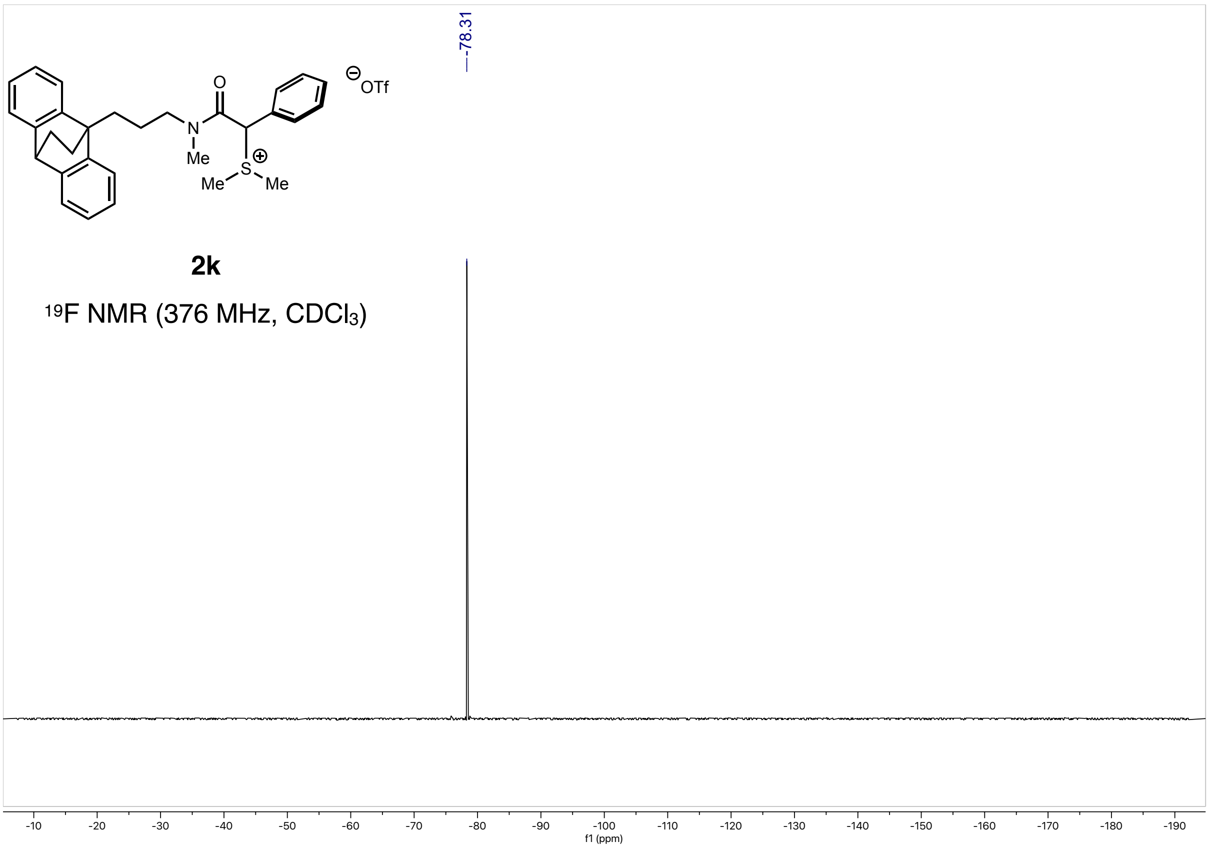
-78.31
⊖OTf
O
N
Me
S⊕
Me
Me
2k
^{19}F NMR (376 MHz, CDCl$_3$)
-10 -20 -30 -40 -50 -60 -70 -80 -90 -100 -110 -120 -130 -140 -150 -160 -170 -180 -190
f1 (ppm)

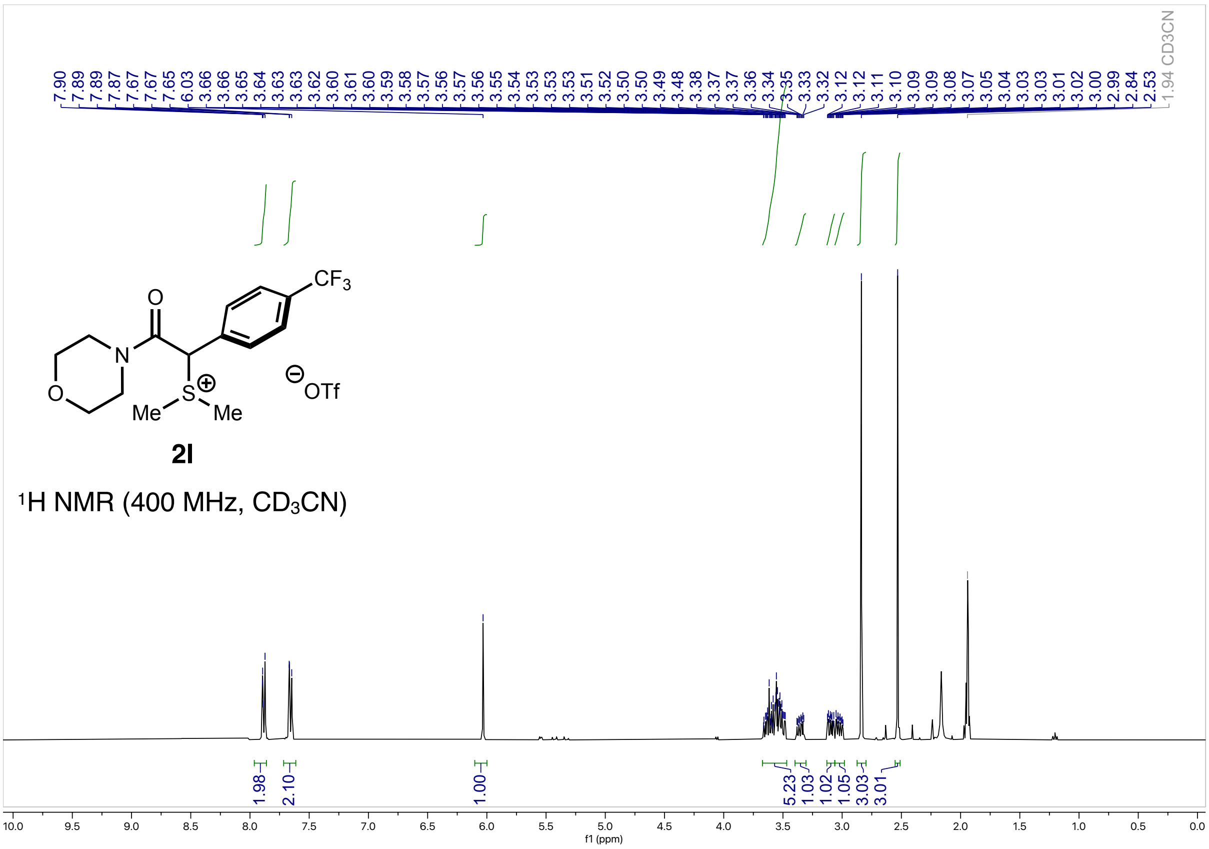

7.90 7.89 7.89 7.87 7.67 7.67 7.65 6.03 3.66 3.66 3.65 3.64 3.63 3.63 3.62 3.60 3.61 3.60 3.59 3.58 3.57 3.56 3.57 3.56 3.55 3.54 3.53 3.53 3.53 3.51 3.52 3.50 3.50 3.49 3.48 3.38 3.37 3.36 3.34 3.35 3.33 3.32 3.12 3.12 3.11 3.10 3.09 3.09 3.08 3.07 3.05 3.04 3.03 3.03 3.01 3.02 3.00 2.99 2.84 2.53
1.94 CD3CN
CF3
O
N
O
S⊕
Me
Me
⊖OTf
2l
1H NMR (400 MHz, CD3CN)
1.98 2.10 1.00 5.23 1.03 1.02 1.05 3.03 3.01
10.0 9.5 9.0 8.5 8.0 7.5 7.0 6.5 6.0 5.5 5.0 4.5 4.0 3.5 3.0 2.5 2.0 1.5 1.0 0.5 0.0
f1 (ppm)

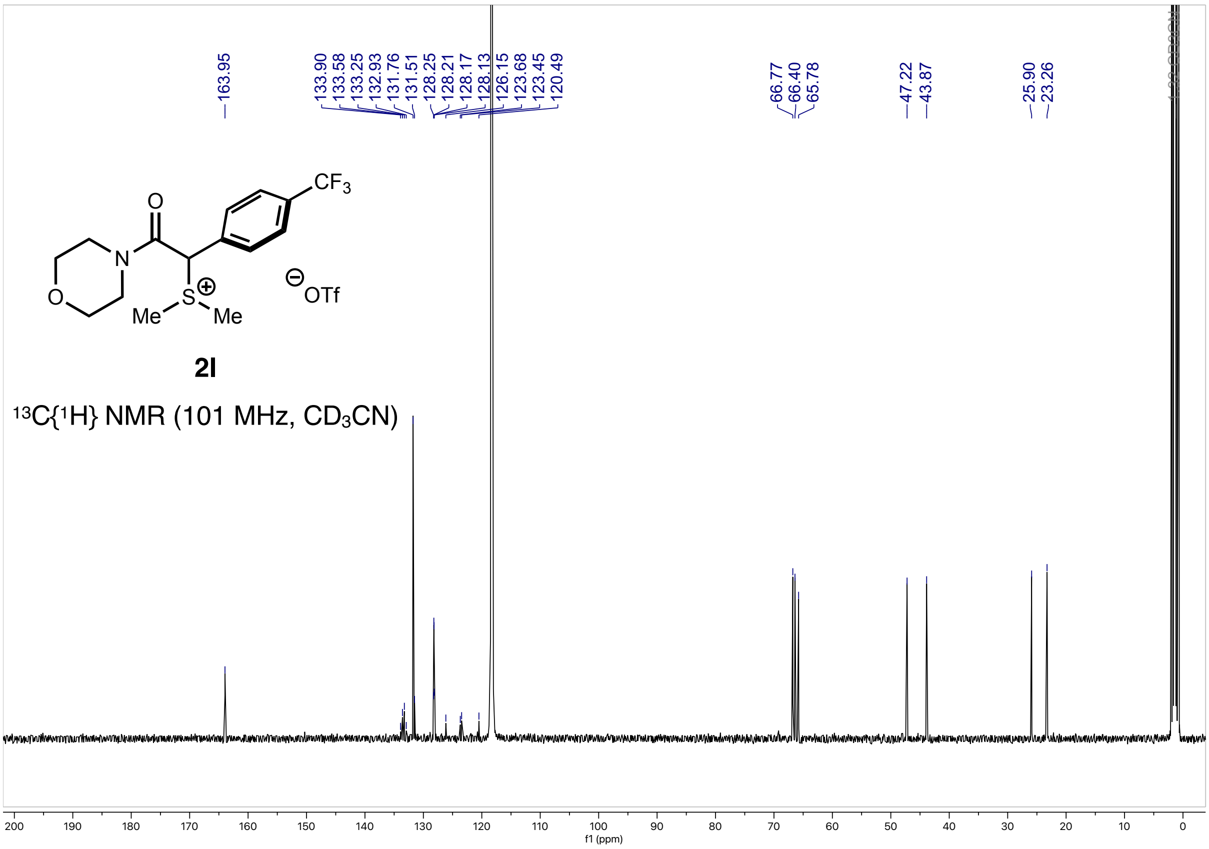

163.95
133.90 133.58 133.25 132.93 131.76 131.51 128.25 128.21 128.17 128.13 126.15 123.68 123.45 120.49
66.77 66.40 65.78
47.22 43.87
25.90 23.26
CF3
O
N
O
S⊕
Me
Me
⊖OTf
2l
13C{1H} NMR (101 MHz, CD3CN)
200 190 180 170 160 150 140 130 120 110 100 90 80 70 60 50 40 30 20 10 0
f1 (ppm)

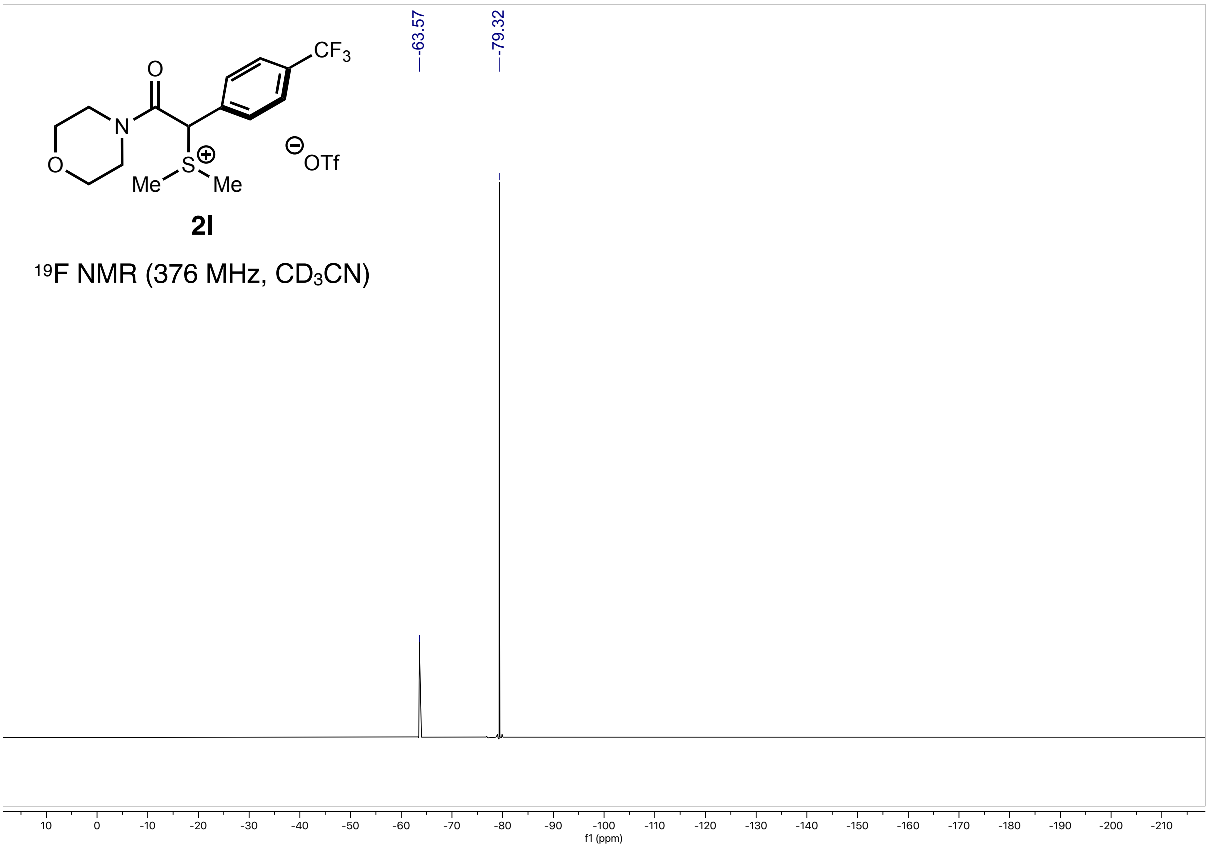

-63.57
-79.32
CF3
O
N
O
S
Me
Me
OTf
2l
19F NMR (376 MHz, CD3CN)
10
0
-10
-20
-30
-40
-50
-60
-70
-80
-90
-100
-110
-120
-130
-140
-150
-160
-170
-180
-190
-200
-210
f1 (ppm)

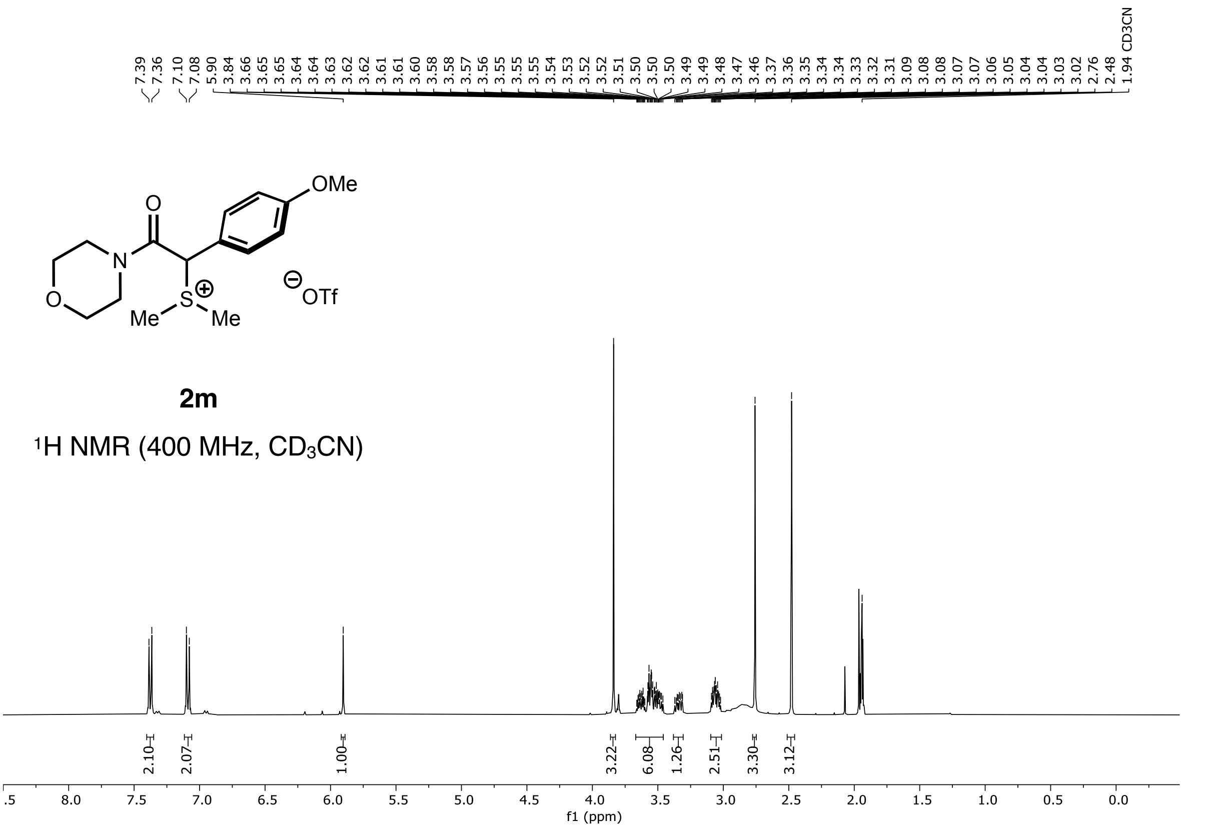

2m

^{1}H NMR (400 MHz, CD_3CN)

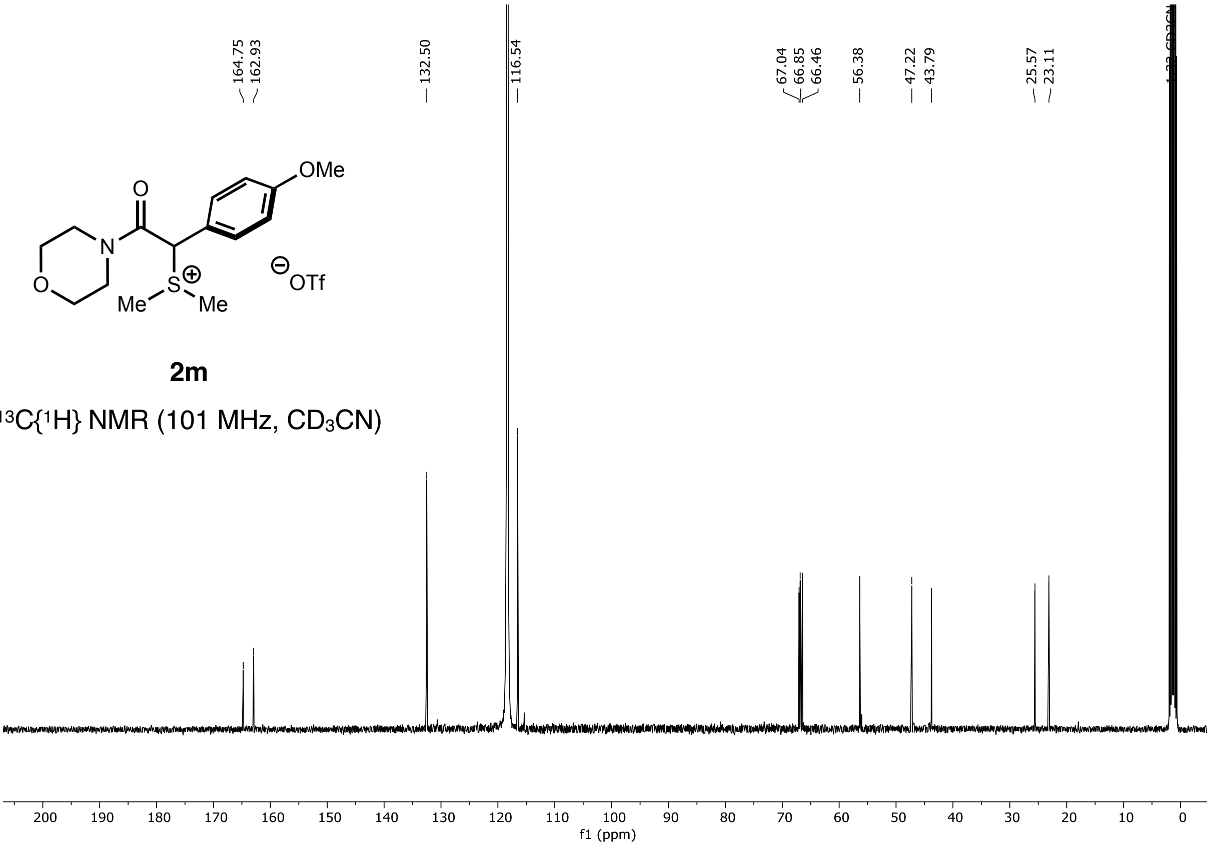

2m

$^{13}C\{^{1}H\}$ NMR (101 MHz, CD_3CN)

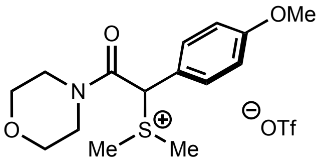

2m

^{19}F NMR (376 MHz, CD_3CN)

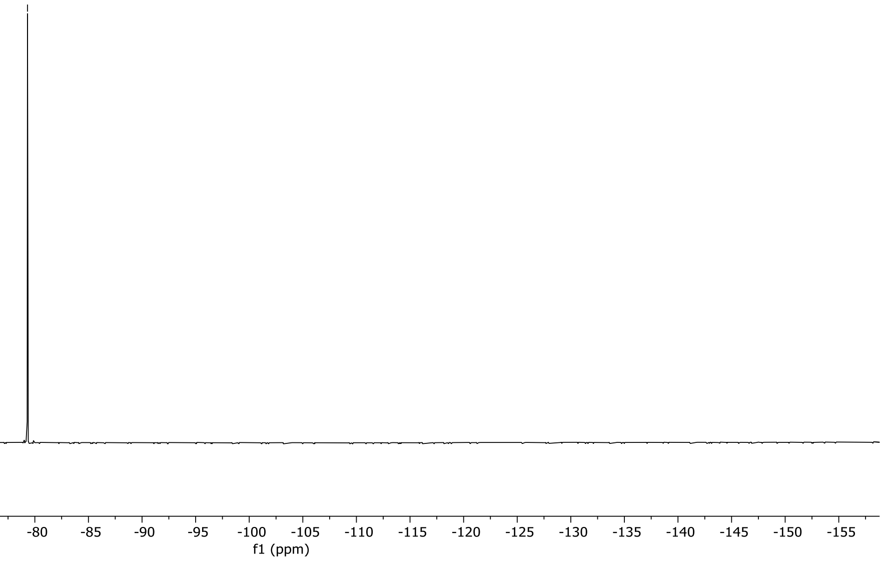

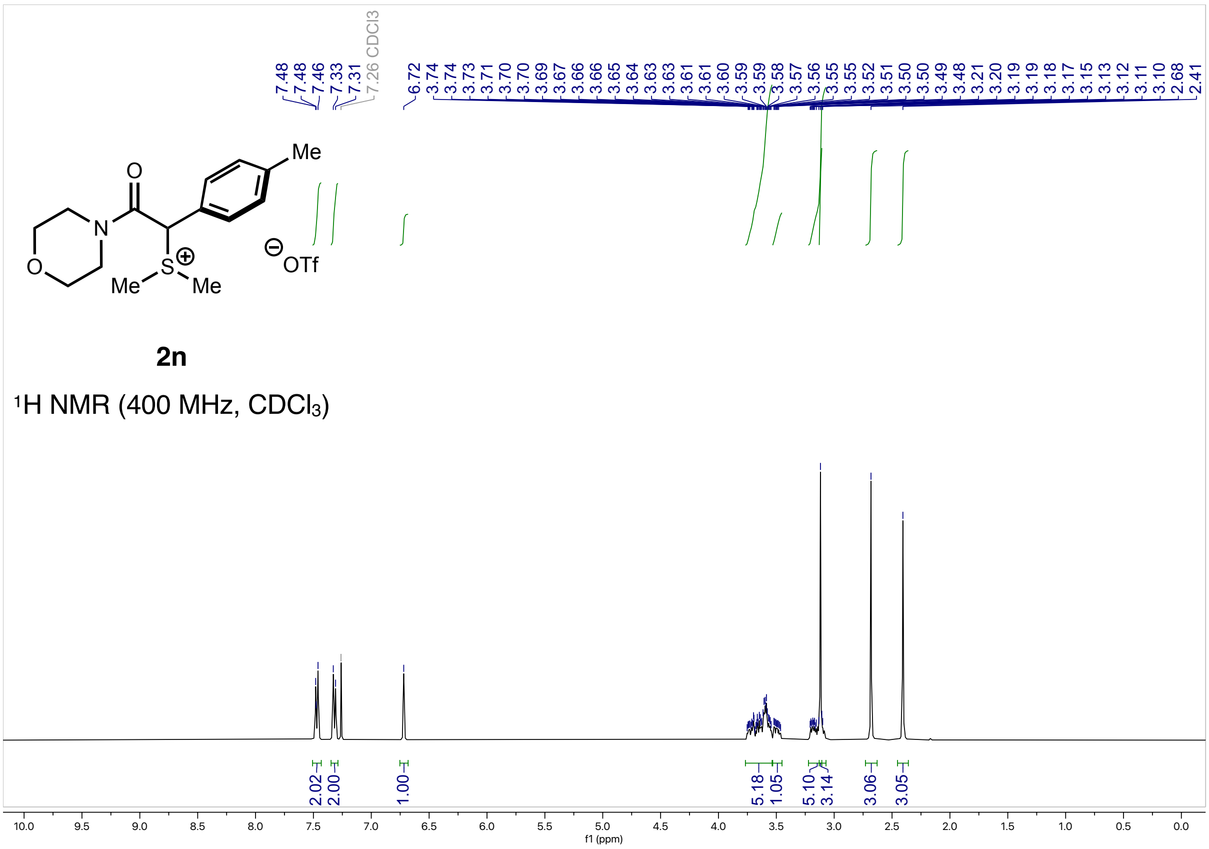

2n

^{1}H NMR (400 MHz, $CDCl_3$)

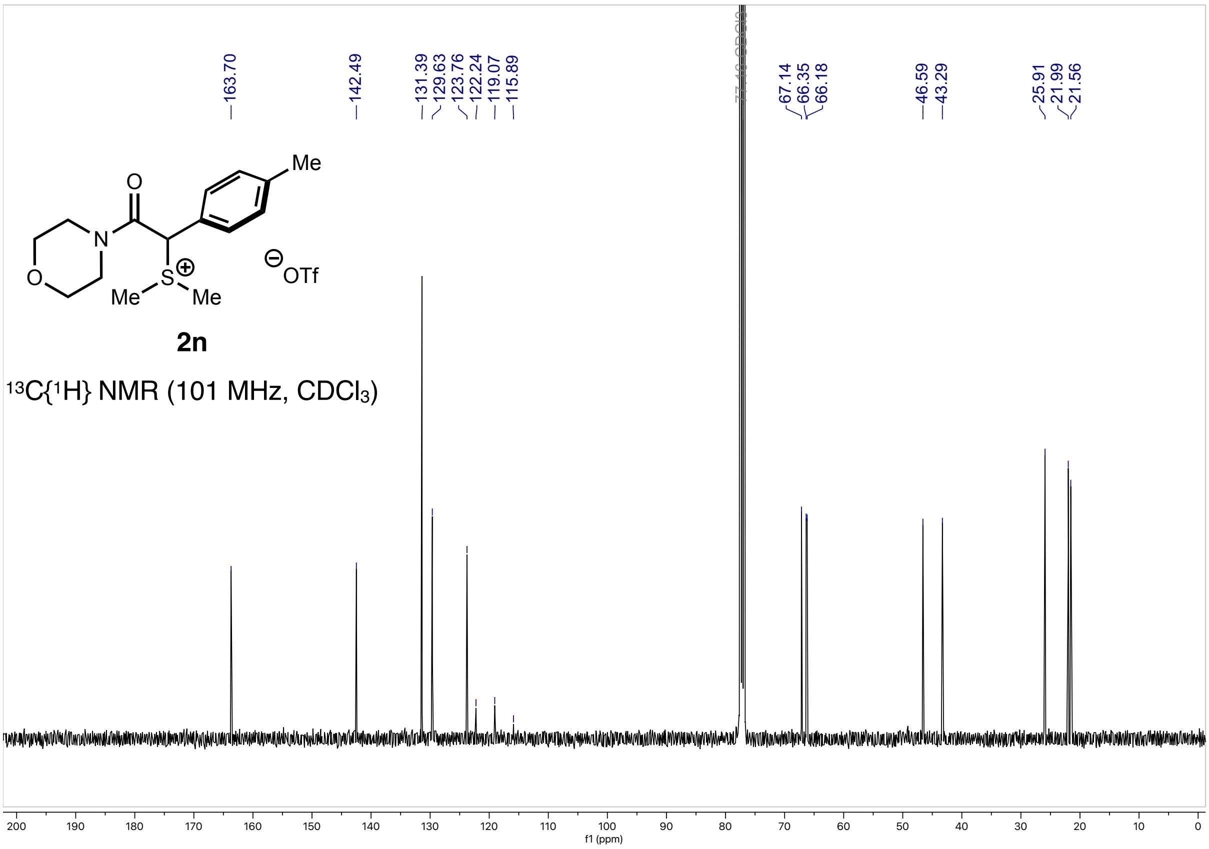

2n

$^{13}C\{^1H\}$ NMR (101 MHz, $CDCl_3$)

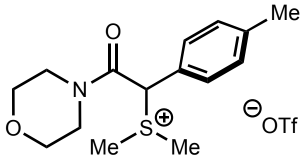

2n

^{19}F NMR (376 MHz, $CDCl_3$)

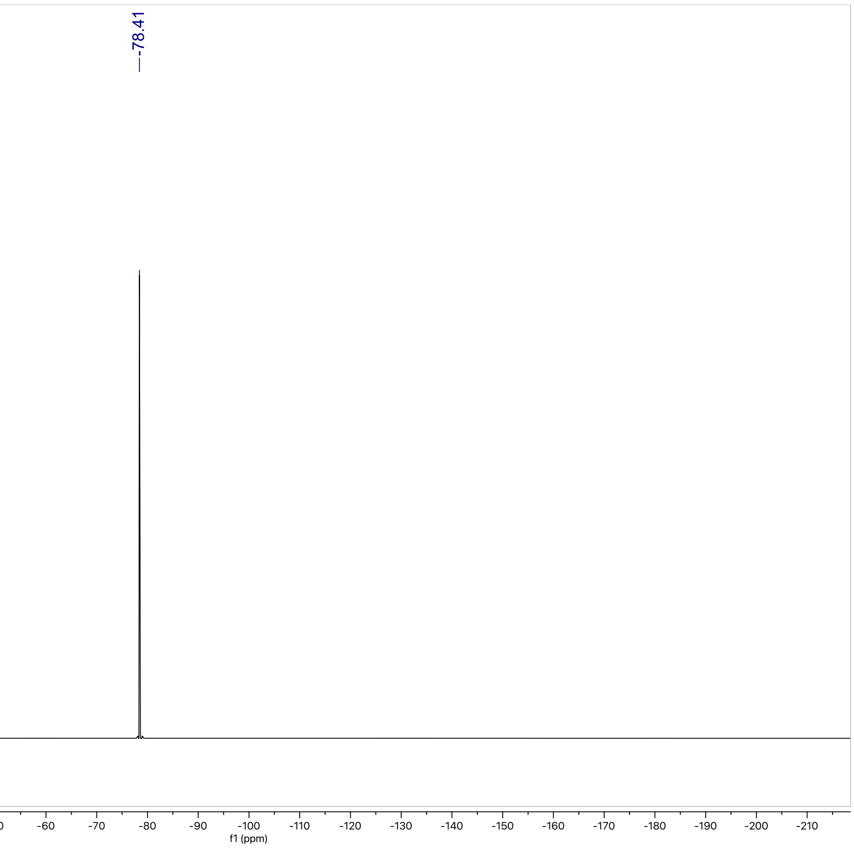

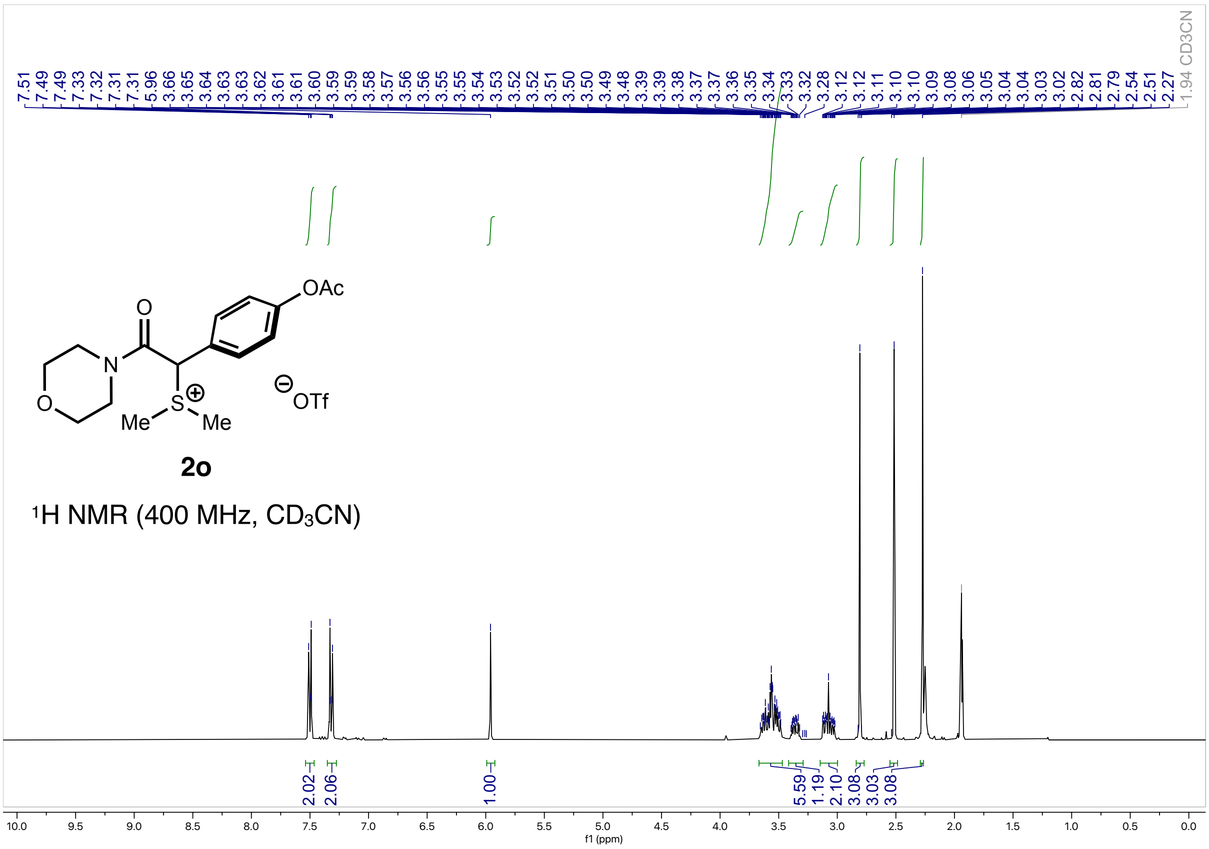

OAc
O
N
O
S
Me
Me
OTf
2o
^{1}H NMR (400 MHz, CD$_3$CN)
1.94 CD3CN
2.02
2.06
1.00
5.59
1.19
2.10
3.08
3.03
3.08
f1 (ppm)

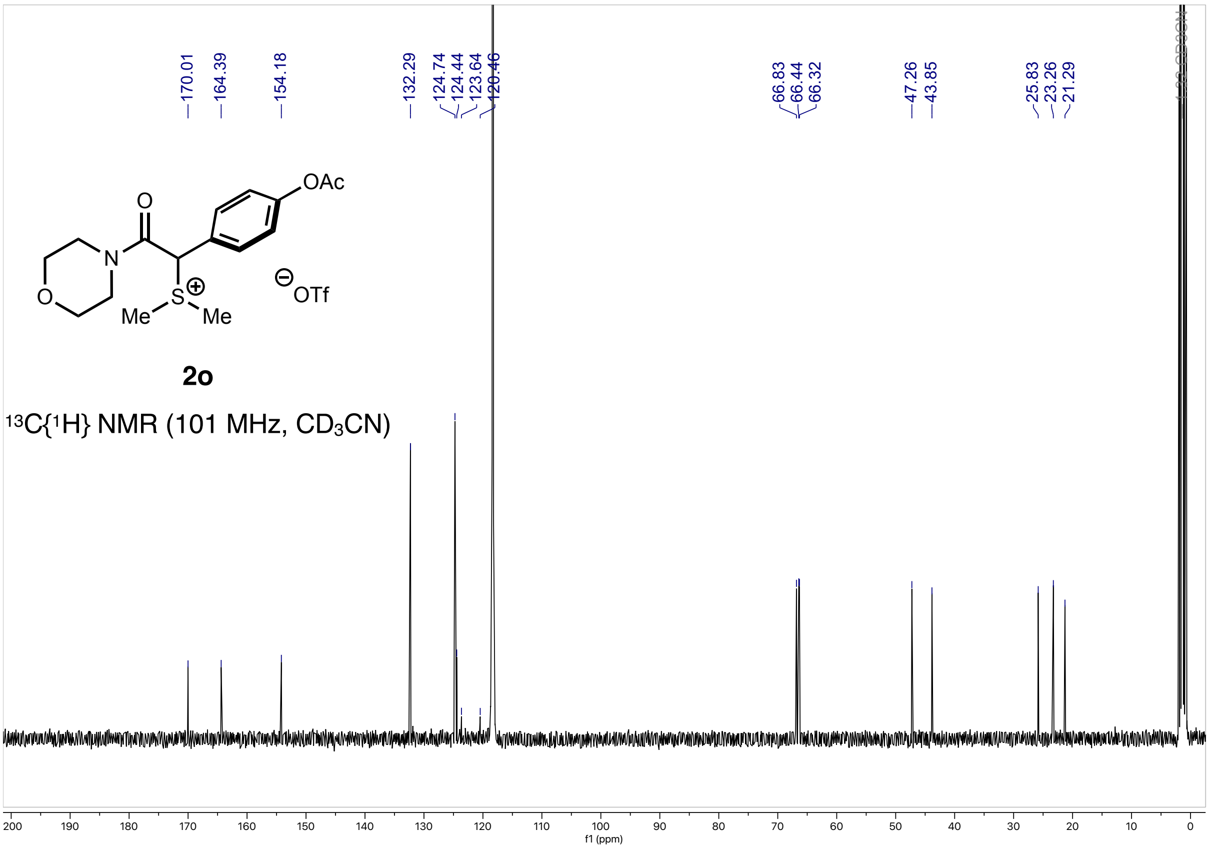

170.01
164.39
154.18
132.29
124.74
124.44
123.64
120.46
66.83
66.44
66.32
47.26
43.85
25.83
23.26
21.29
OAc
O
N
O
S
Me
Me
OTf
2o
^{13}C{^{1}H} NMR (101 MHz, CD$_3$CN)
f1 (ppm)

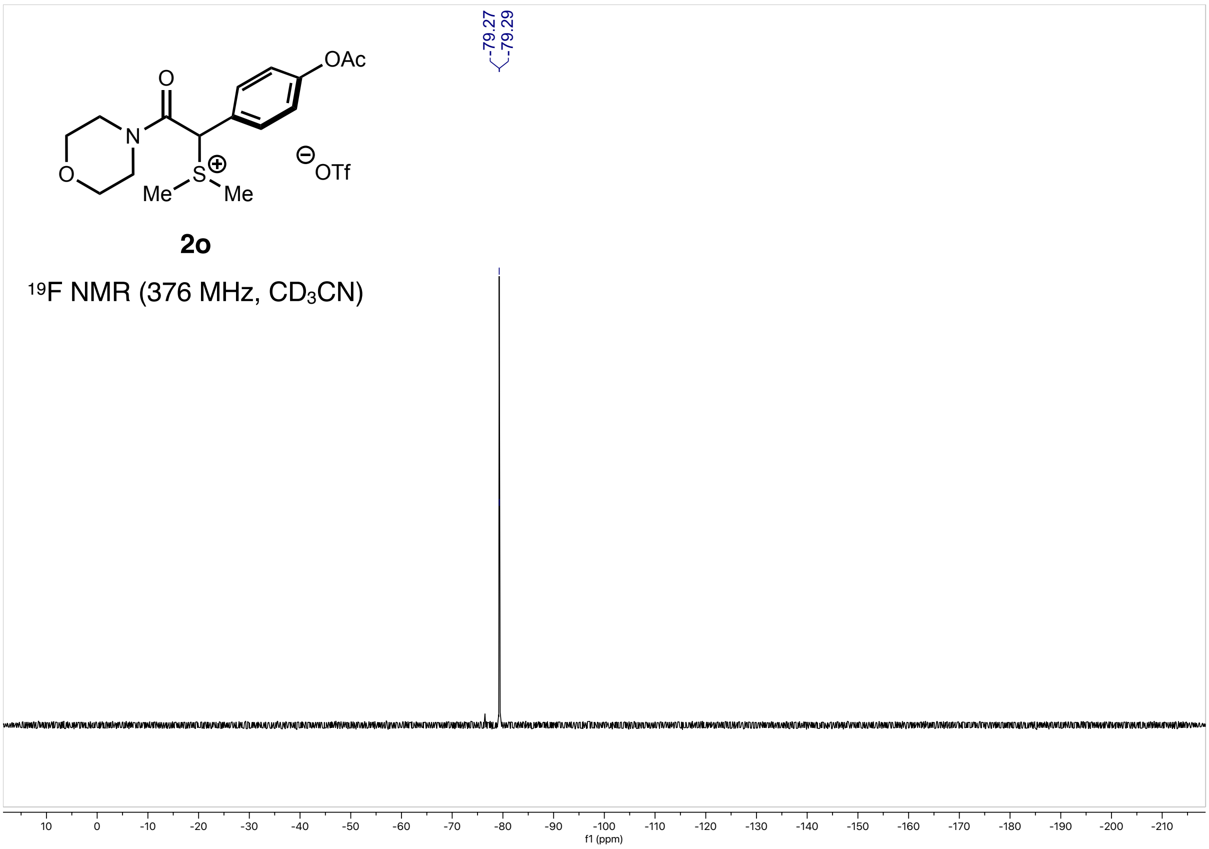

-79.27
-79.29
OAc
O
N
O
S
Me
Me
OTf
2o
^{19}F NMR (376 MHz, CD_3CN)
10
0
-10
-20
-30
-40
-50
-60
-70
-80
-90
-100
-110
-120
-130
-140
-150
-160
-170
-180
-190
-200
-210
f1 (ppm)

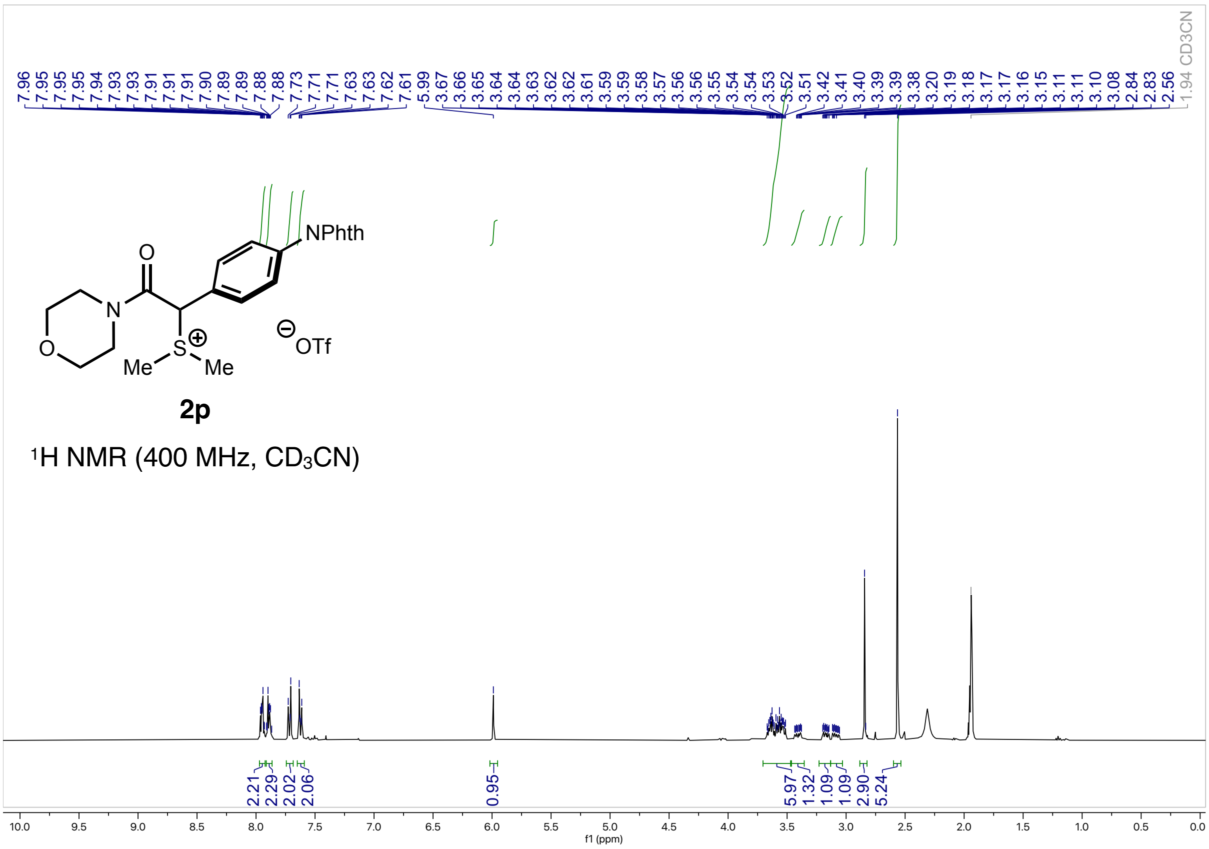

2p

^{1}H NMR (400 MHz, CD_3CN)

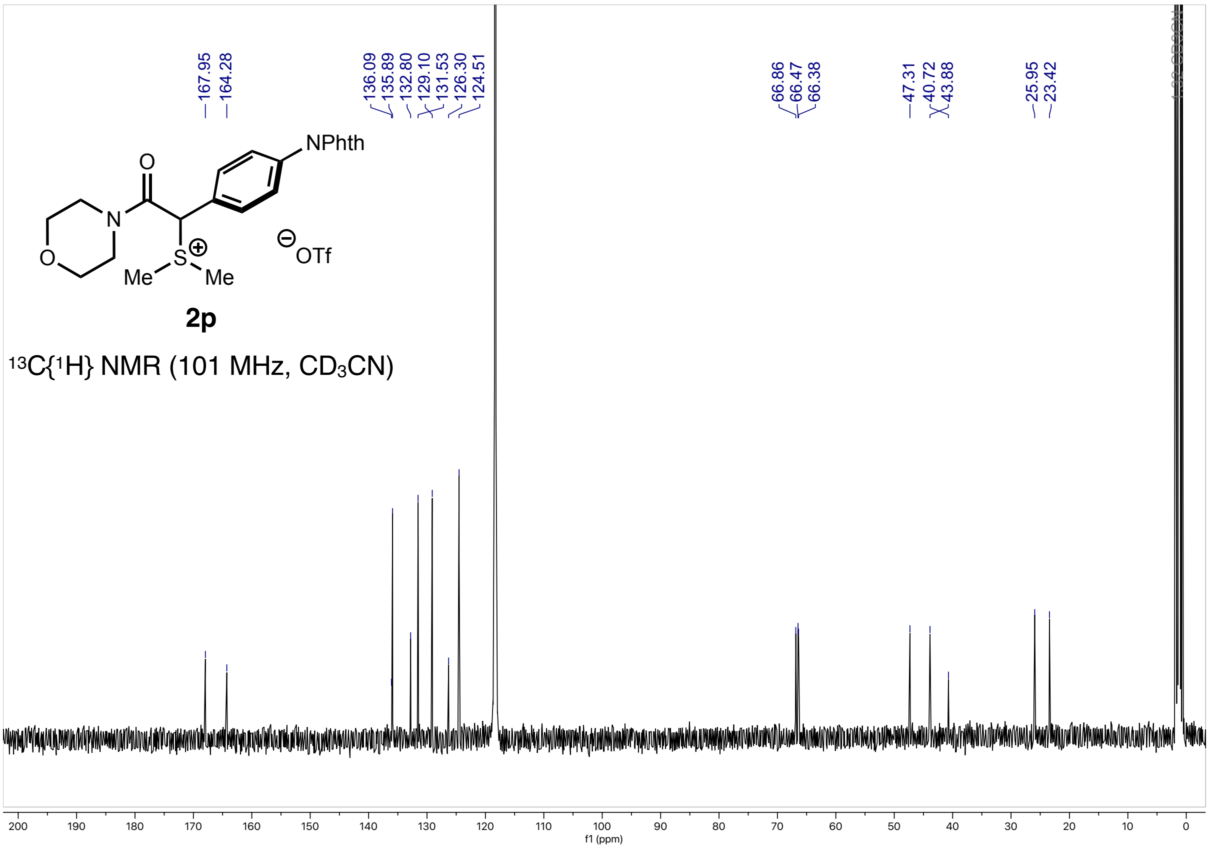

2p

$^{13}C\{^{1}H\}$ NMR (101 MHz, CD_3CN)

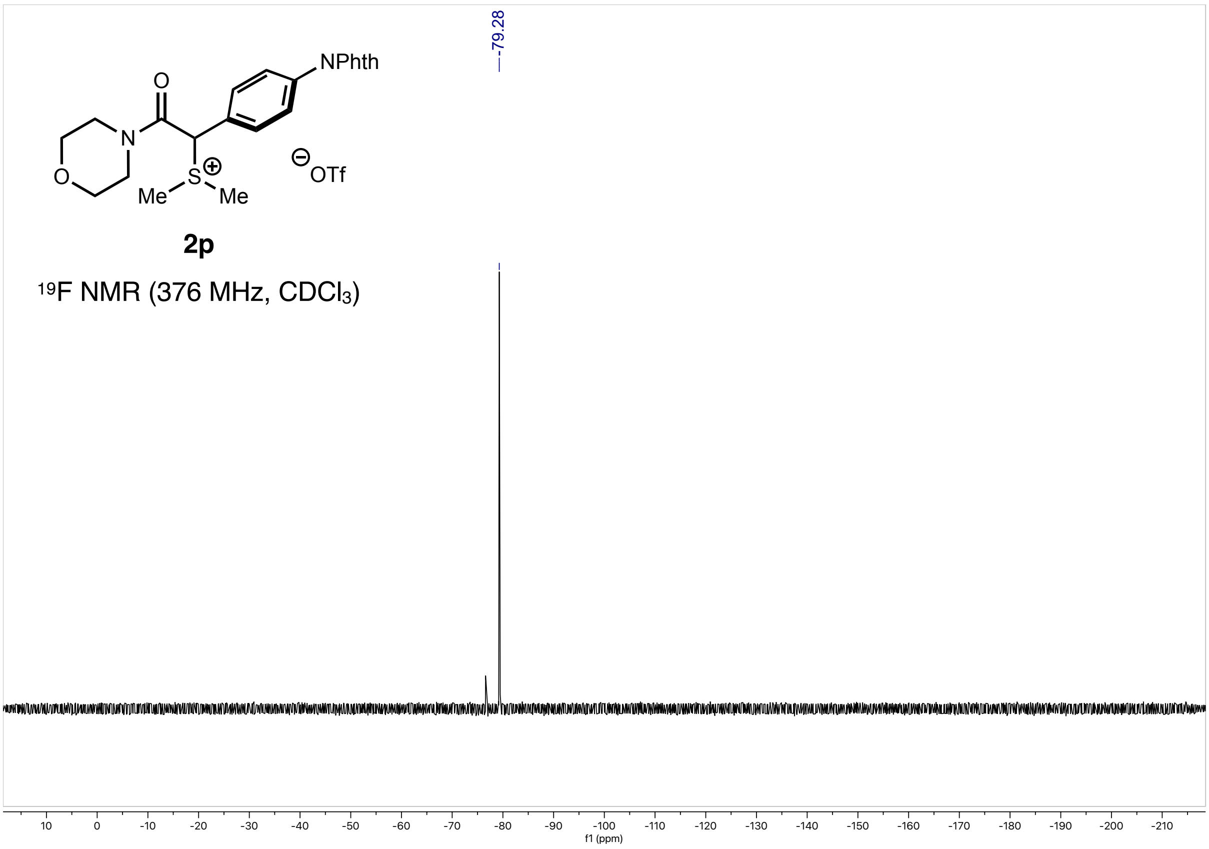

NPhth
O
N
O
S
Me
Me
OTf
2p
¹⁹F NMR (376 MHz, CDCl₃)
-79.28
f1 (ppm)

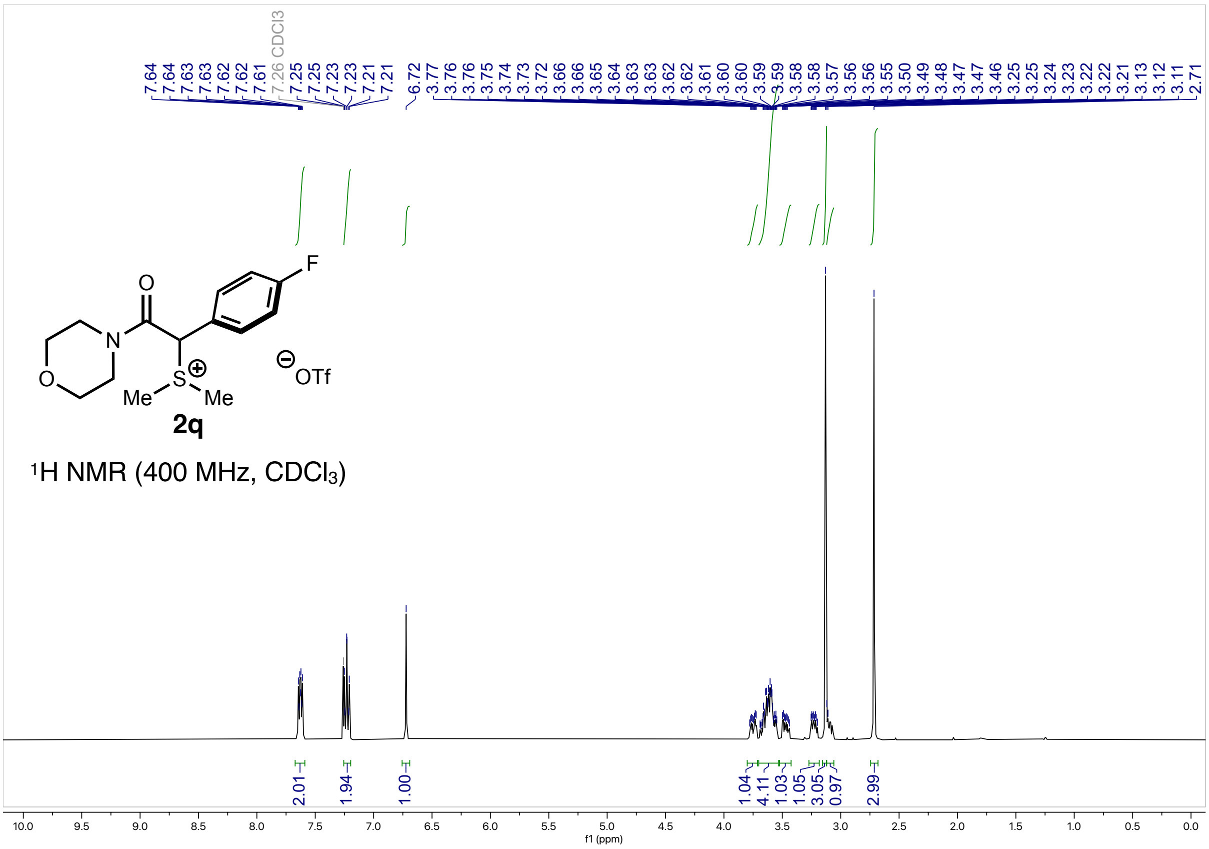
O
F
N
O
S
Me
Me
OTf
2q
¹H NMR (400 MHz, CDCl₃)
7.64
7.64
7.63
7.63
7.62
7.62
7.61
7.26 CDCl3
7.25
7.25
7.23
7.23
7.21
7.21
6.72
3.77
3.76
3.76
3.75
3.74
3.73
3.72
3.66
3.65
3.64
3.63
3.63
3.62
3.62
3.61
3.61
3.60
3.60
3.59
3.59
3.58
3.58
3.57
3.56
3.56
3.55
3.50
3.49
3.48
3.47
3.47
3.46
3.25
3.25
3.24
3.23
3.22
3.22
3.21
3.13
3.12
3.11
2.71
2.01
1.94
1.00
1.04
4.11
1.03
1.05
3.05
0.97
2.99
f1 (ppm)

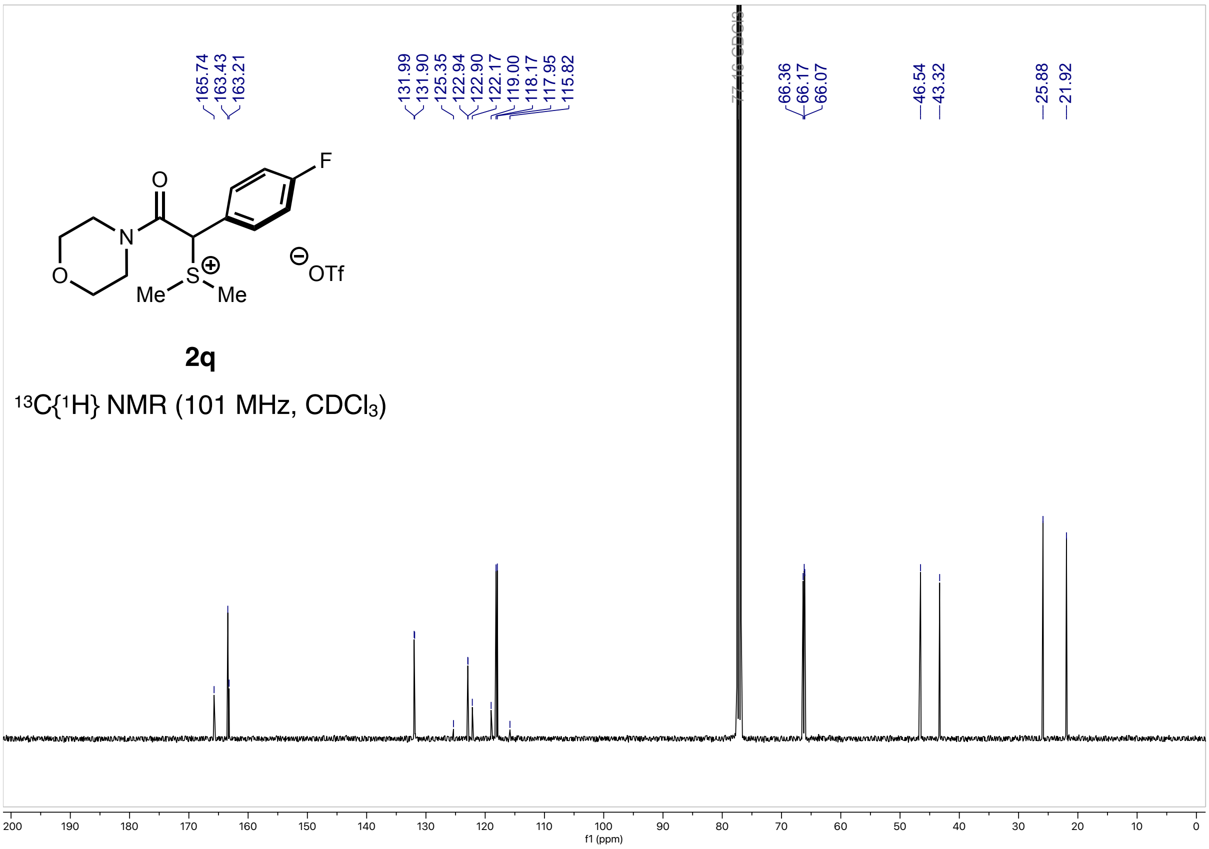
165.74
163.43
163.21
131.99
131.90
125.35
122.94
122.90
122.17
119.00
118.17
117.95
115.82
77.16 CDCl3
66.36
66.17
66.07
46.54
43.32
25.88
21.92
O
F
N
O
S
Me
Me
OTf
2q
¹³C{¹H} NMR (101 MHz, CDCl₃)
f1 (ppm)

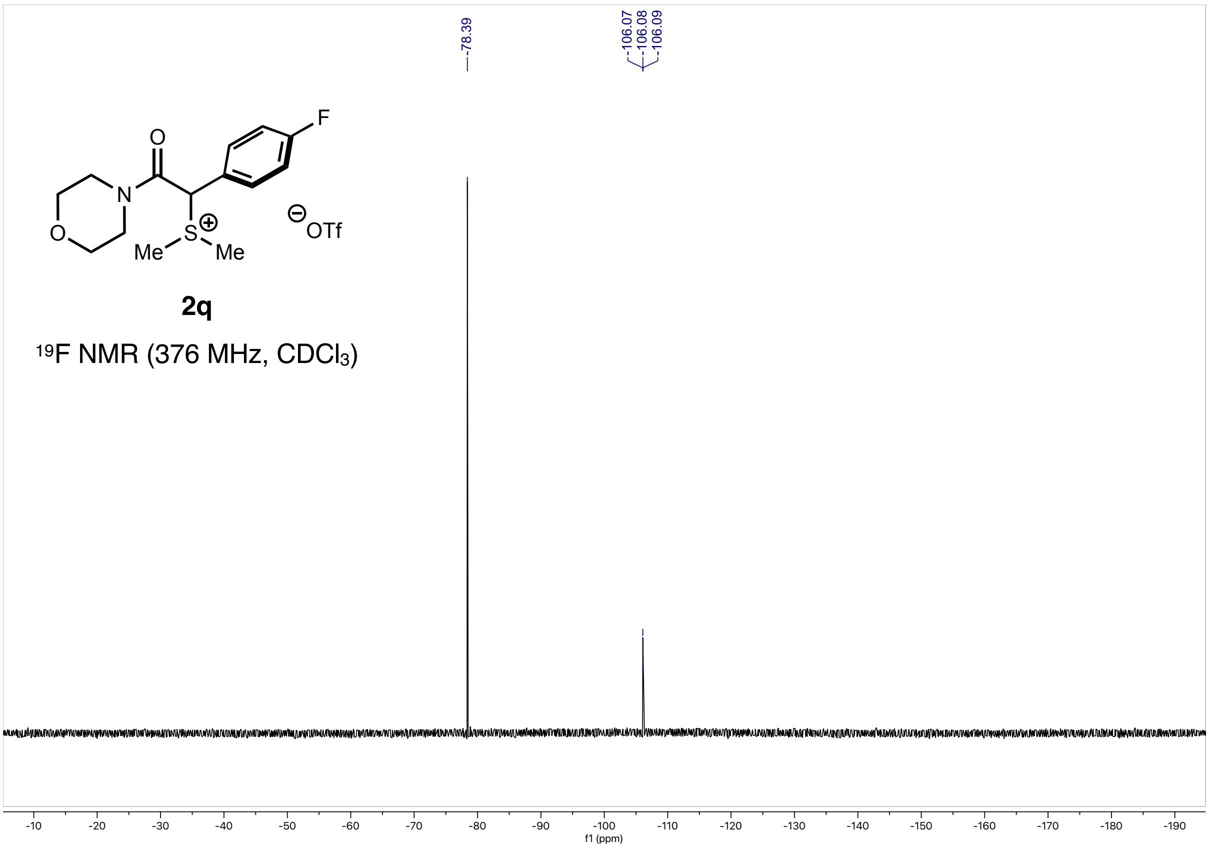
-78.39
-106.07
-106.08
-106.09
O
N
F
O
S
Me
Me
OTf
2q
19F NMR (376 MHz, CDCl3)
f1 (ppm)

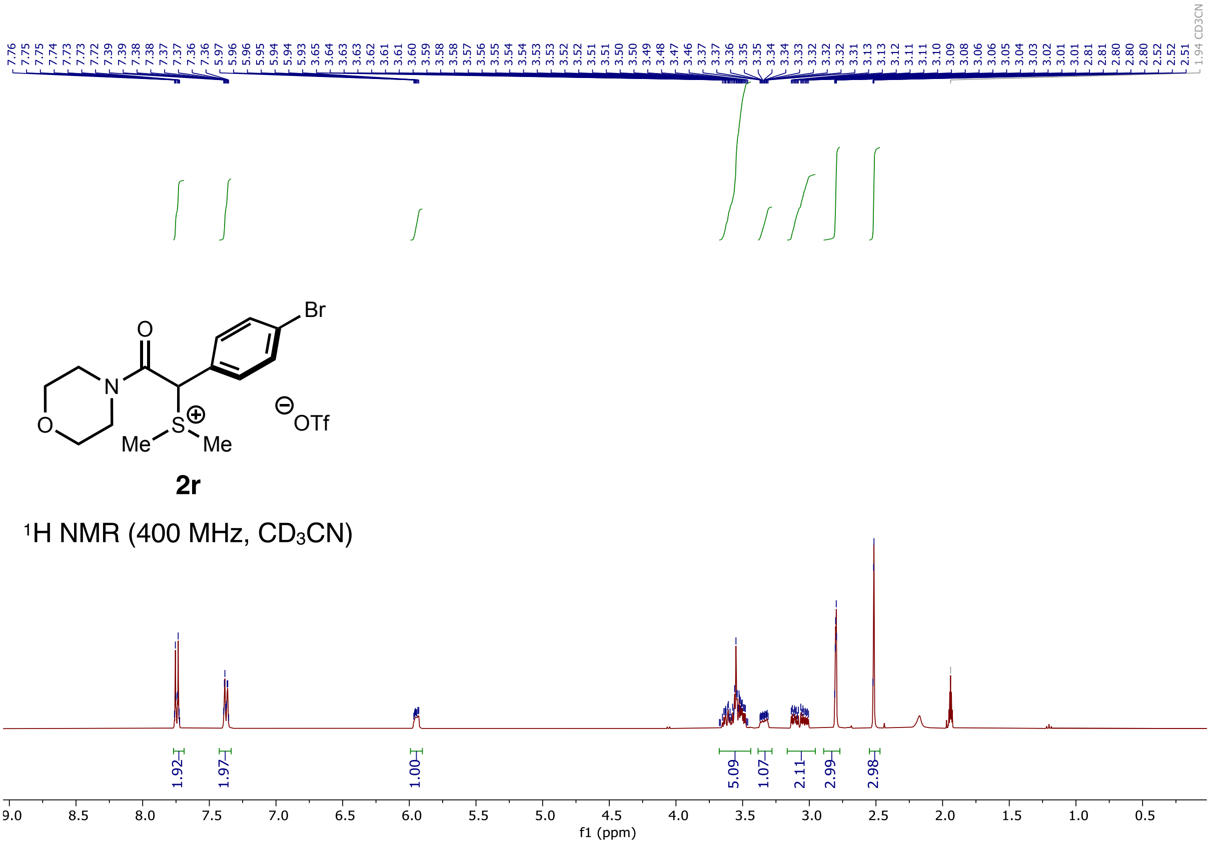

2r

^{1}H NMR (400 MHz, CD_3CN)

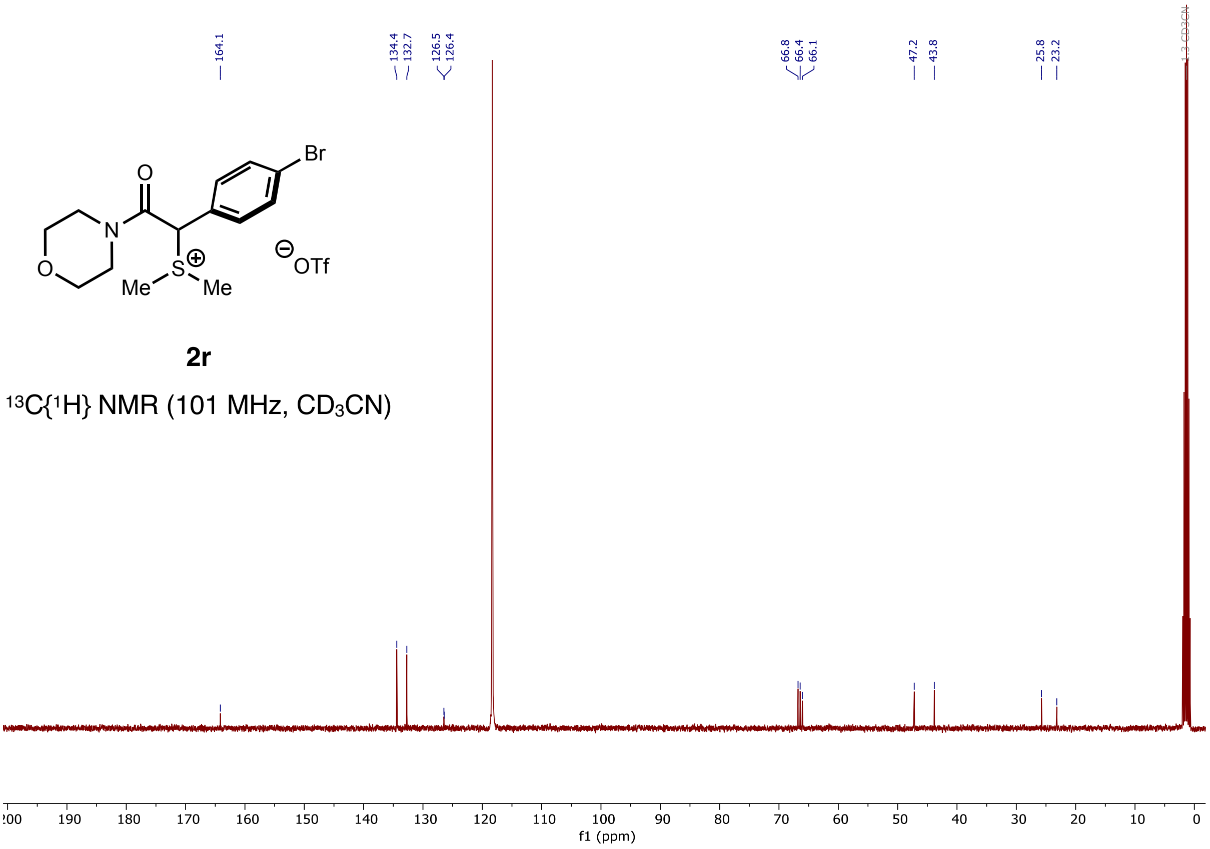

2r

$^{13}C\{^{1}H\}$ NMR (101 MHz, CD_3CN)

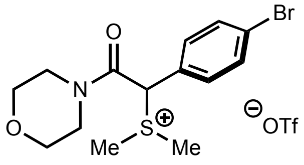

2r

^{19}F NMR (376 MHz, CD_3CN)

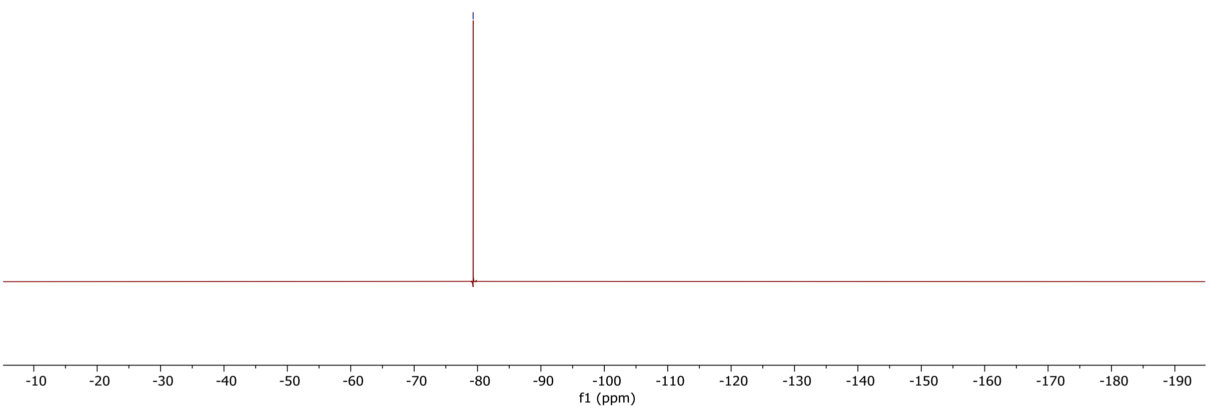

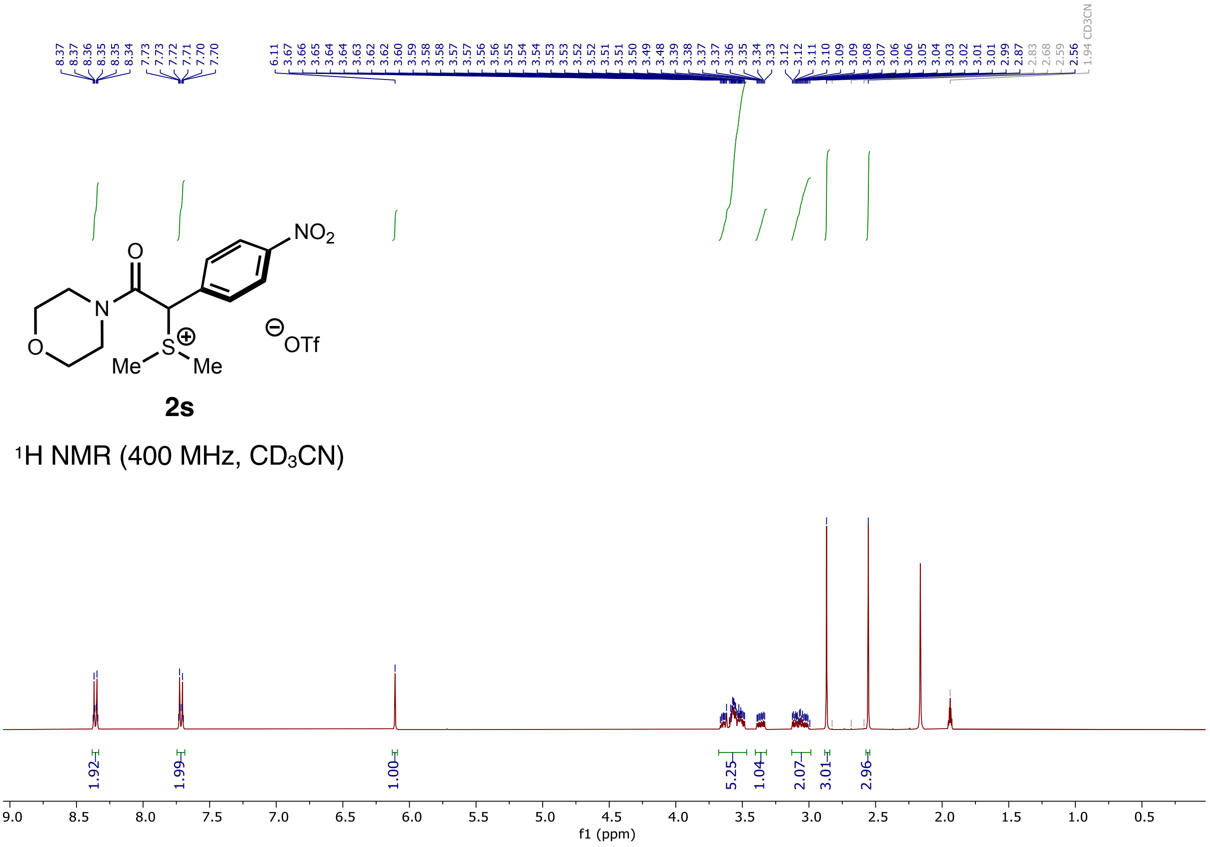

2s

^{1}H NMR (400 MHz, CD_3CN)

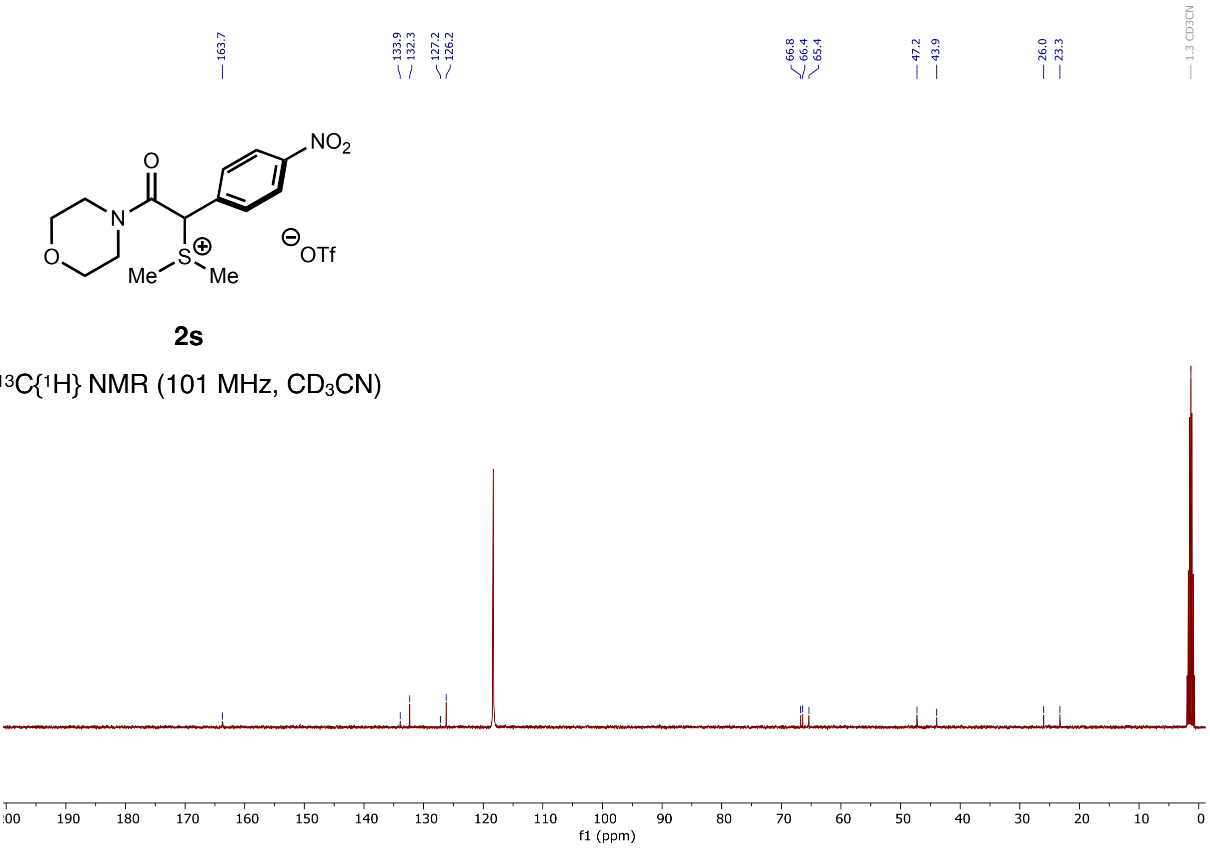

2s

$^{13}C\{^{1}H\}$ NMR (101 MHz, CD_3CN)

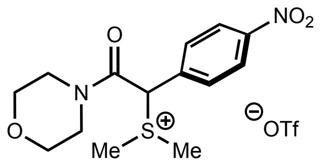

2s

^{19}F NMR (376 MHz, CD_3CN)

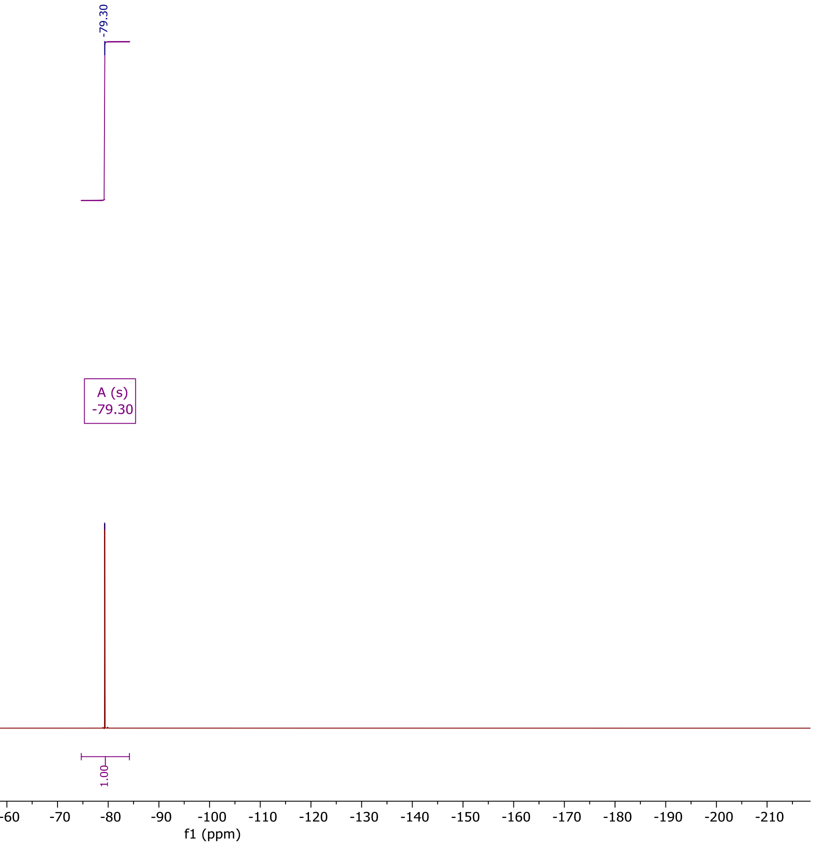

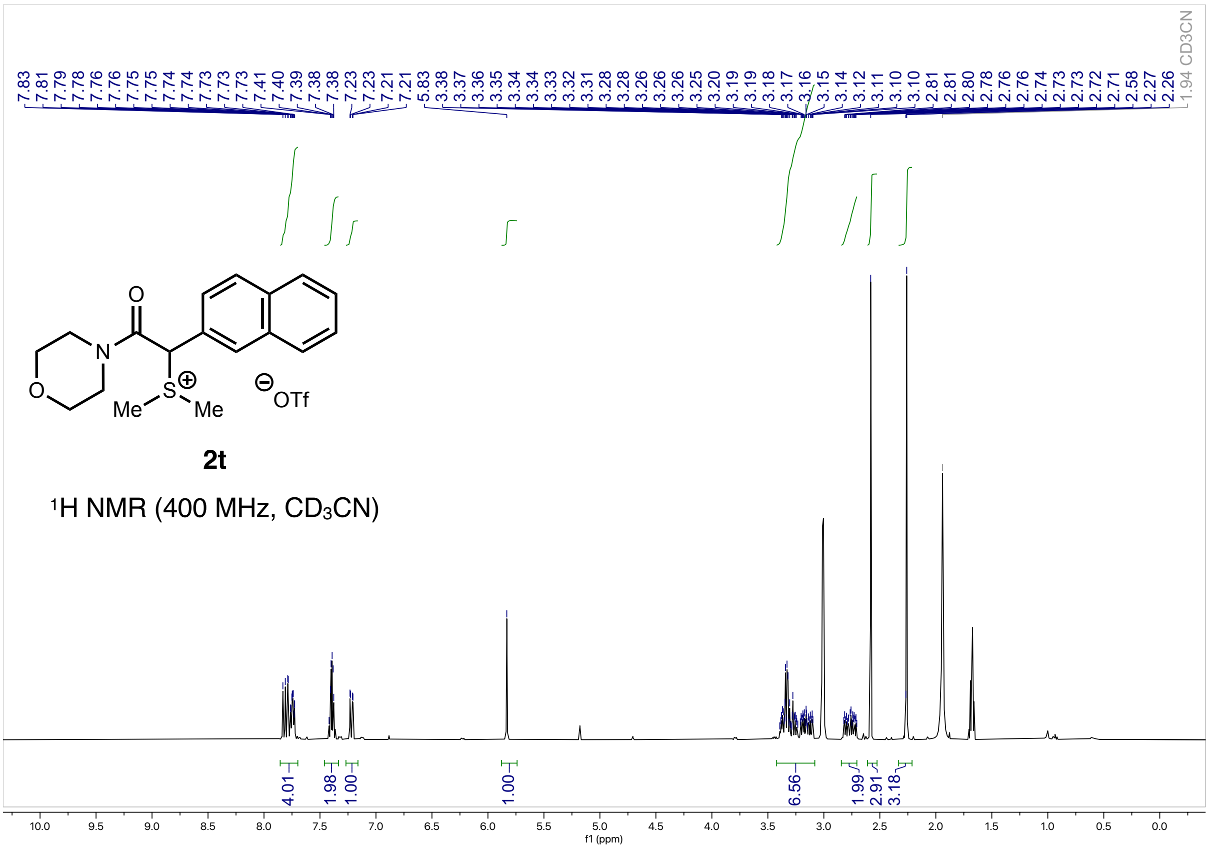

2t

^{1}H NMR (400 MHz, CD_3CN)

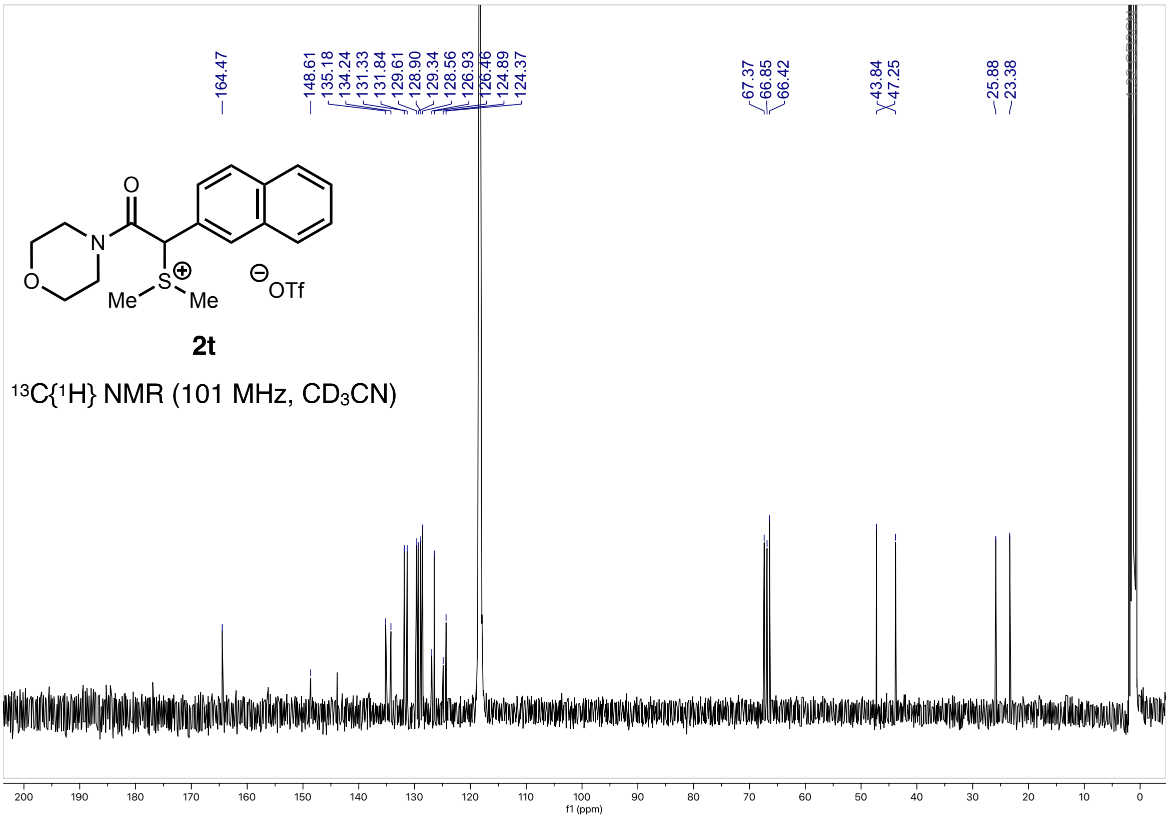

2t

$^{13}C\{^{1}H\}$ NMR (101 MHz, CD_3CN)

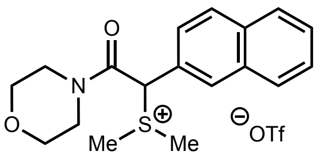

2t

^{19}F NMR (376 MHz, CD_3CN)

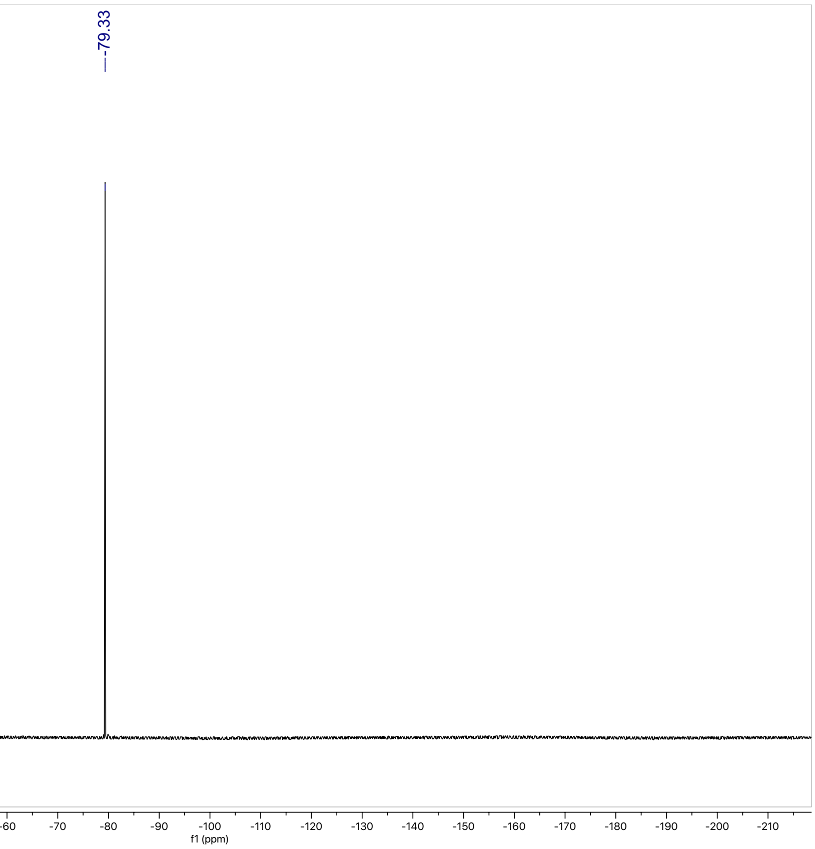

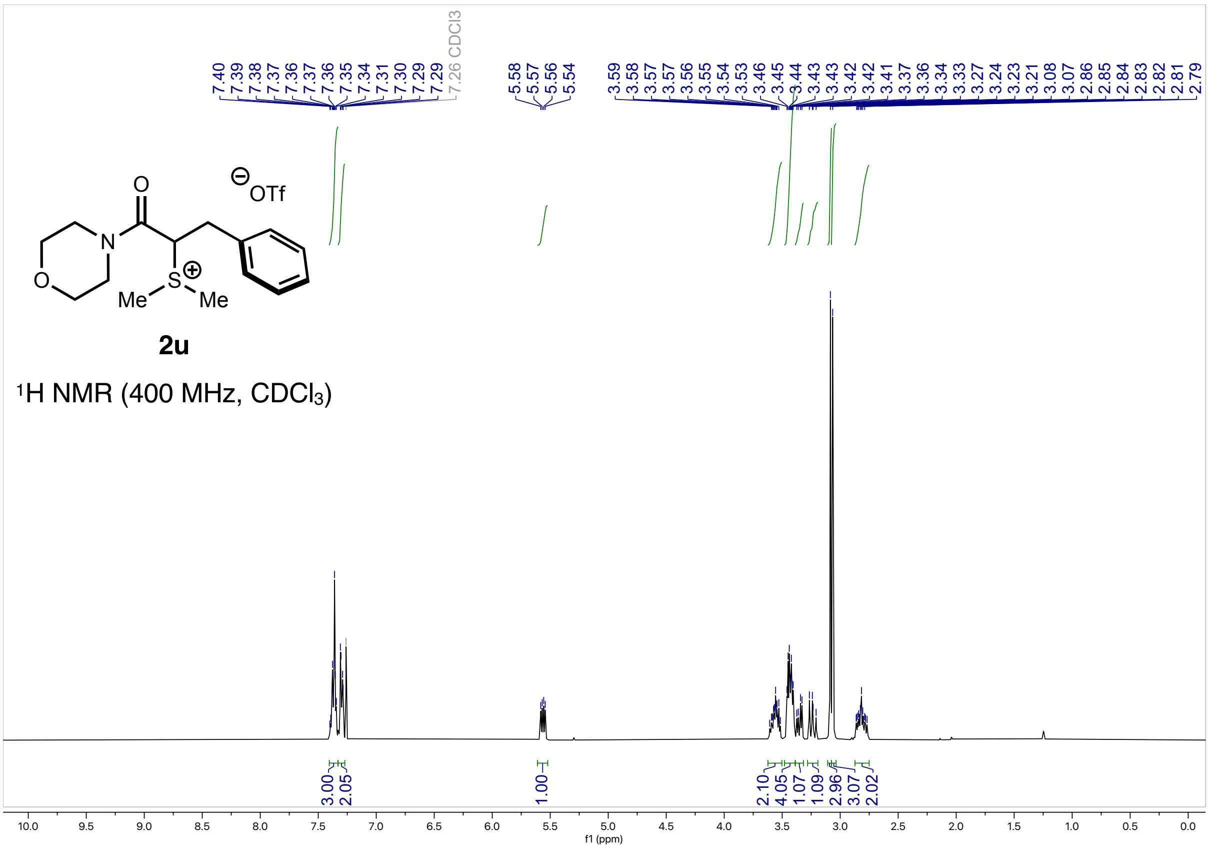
7.40 7.39 7.38 7.37 7.36 7.37 7.36 7.35 7.34 7.31 7.30 7.29 7.29
7.26 CDCl3
5.58 5.57 5.56 5.54
3.59 3.58 3.57 3.57 3.56 3.55 3.54 3.53 3.46 3.45 3.44 3.43 3.43 3.42 3.42 3.41 3.37 3.36 3.34 3.33 3.27 3.24 3.23 3.21 3.08 3.07 2.86 2.85 2.84 2.83 2.82 2.81 2.79
O
N
S
Me
Me
OTf
2u
1H NMR (400 MHz, CDCl3)
3.00
2.05
1.00
2.10
4.05
1.07
1.09
2.96
3.07
2.02
f1 (ppm)

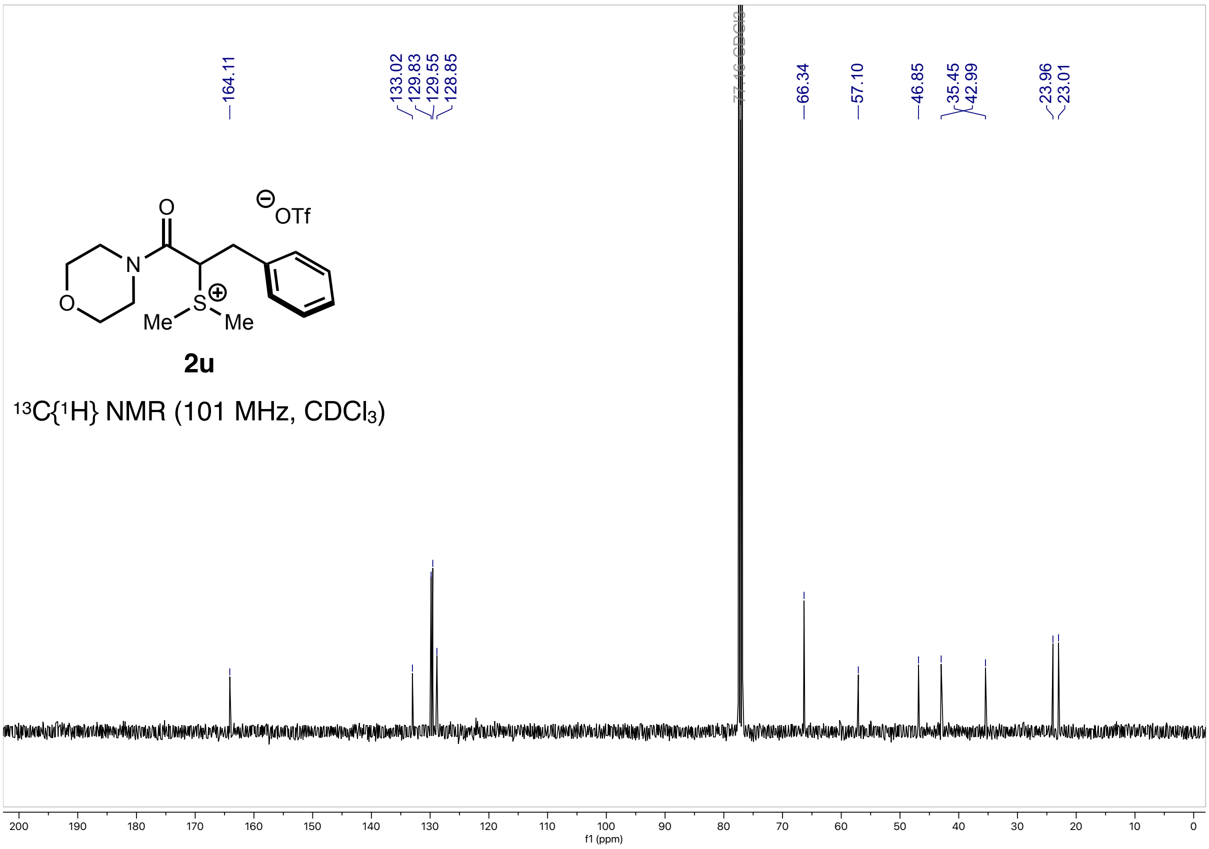
164.11
133.02 129.83 129.55 128.85
77.16 CDCl3
66.34
57.10
46.85
35.45 42.99
23.96 23.01
O
N
S
Me
Me
OTf
2u
13C{1H} NMR (101 MHz, CDCl3)
f1 (ppm)

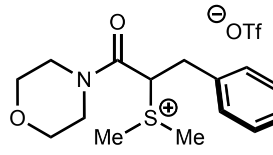

2u

^{19}F NMR (376 MHz, $CDCl_3$)

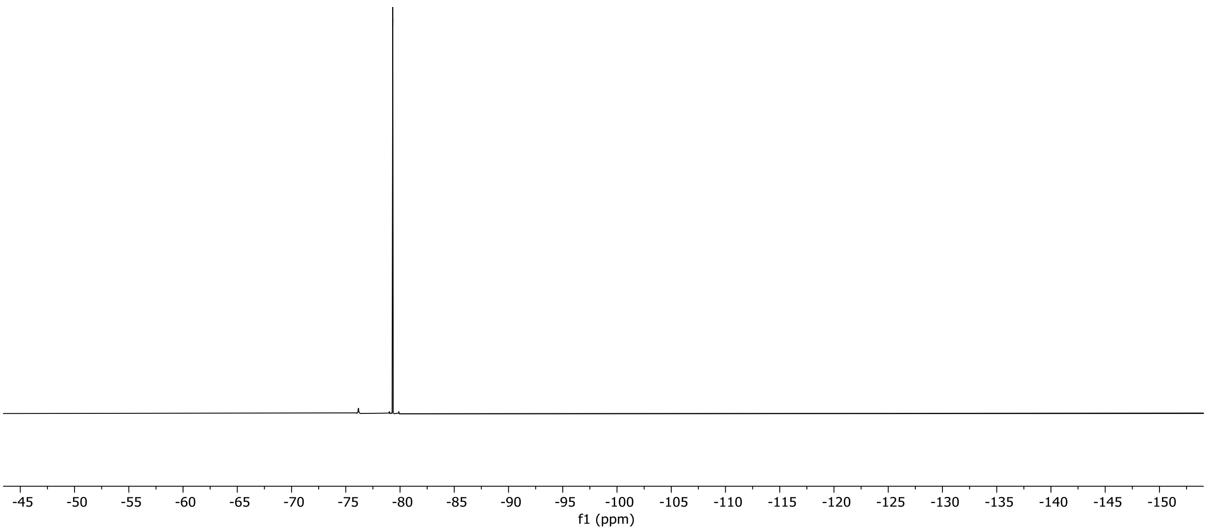

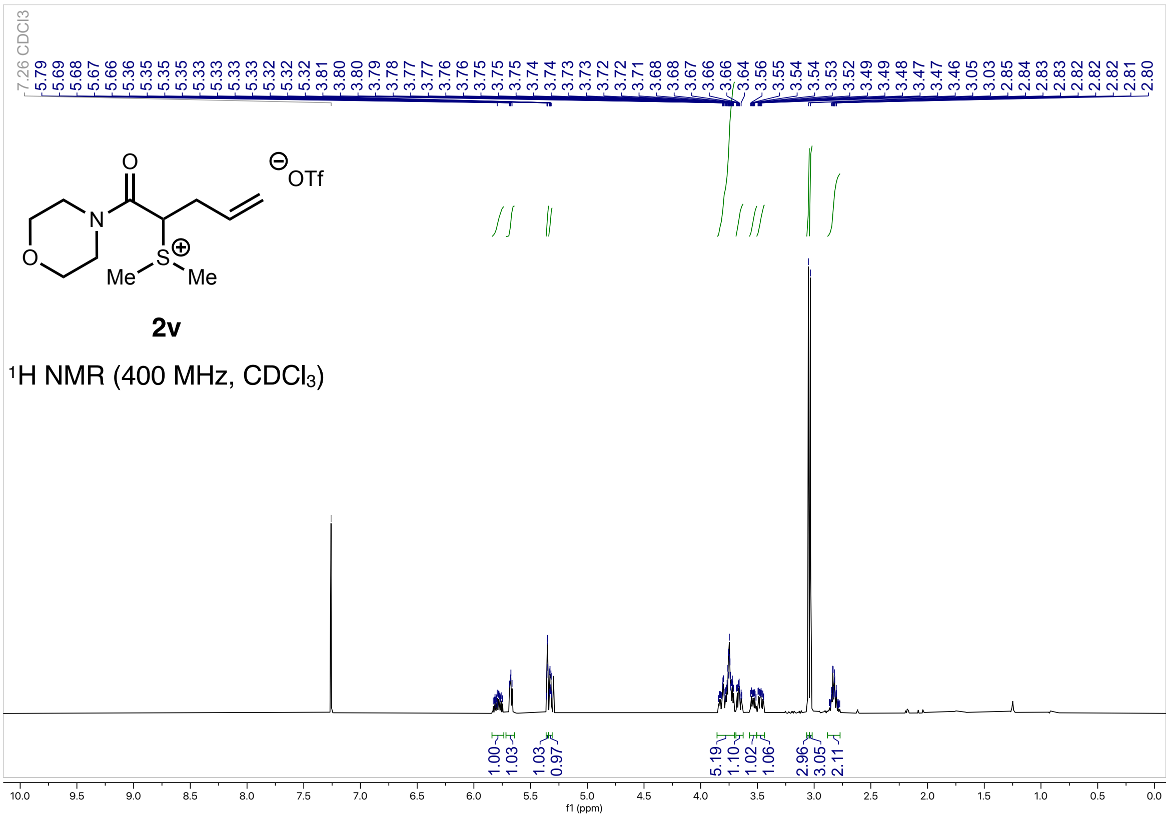
7.26 CDCl3
5.79
5.69
5.68
5.67
5.66
5.36
5.35
5.35
5.35
5.33
5.33
5.33
5.33
5.32
5.32
5.32
3.81
3.80
3.80
3.79
3.78
3.77
3.77
3.76
3.76
3.75
3.75
3.75
3.74
3.74
3.73
3.73
3.72
3.71
3.68
3.68
3.67
3.66
3.66
3.64
3.56
3.55
3.54
3.54
3.53
3.52
3.49
3.49
3.48
3.47
3.47
3.46
3.05
3.03
2.85
2.84
2.83
2.83
2.82
2.82
2.82
2.81
2.80
O
N
O
S
Me
Me
OTf
2v
¹H NMR (400 MHz, CDCl₃)
1.00
1.03
1.03
0.97
5.19
1.10
1.02
1.06
2.96
3.05
2.11
f1 (ppm)

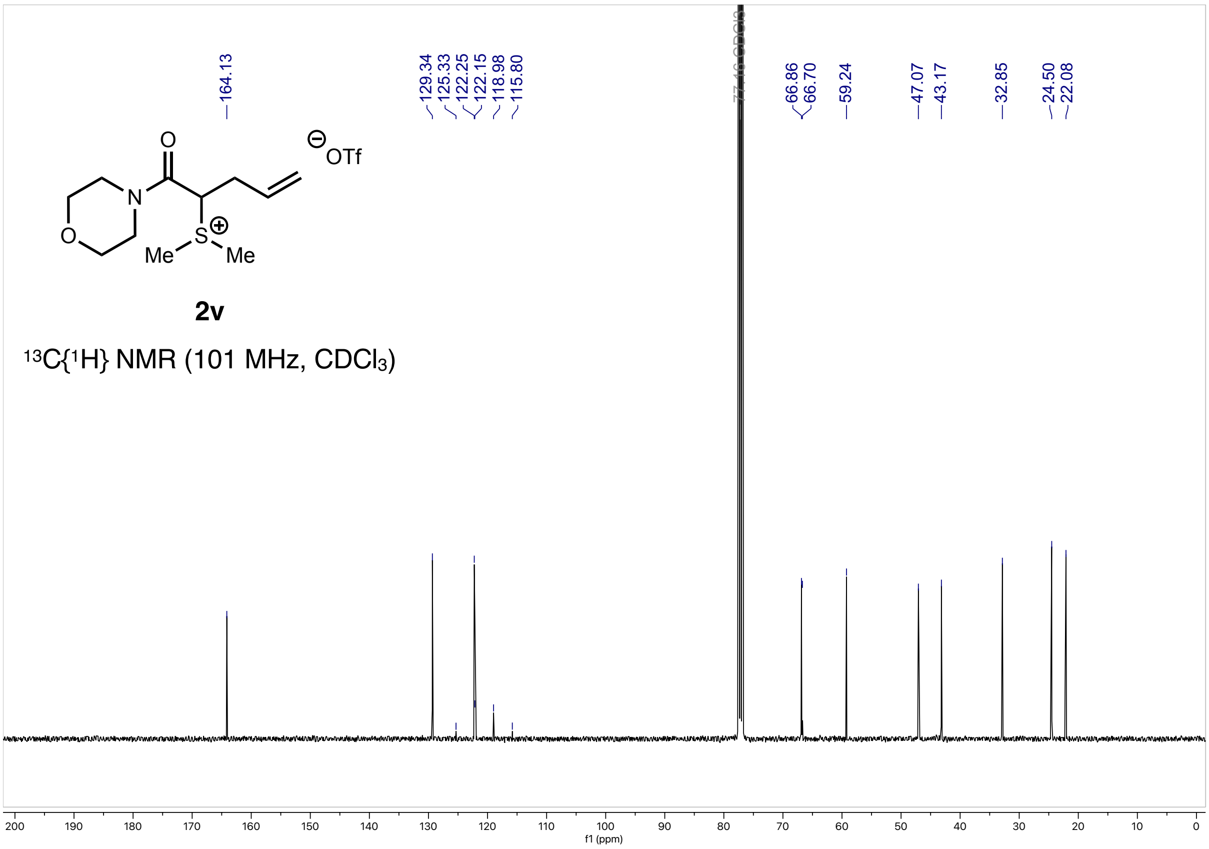
164.13
129.34
125.33
122.25
122.15
118.98
115.80
77.16 CDCl3
66.86
66.70
59.24
47.07
43.17
32.85
24.50
22.08
O
N
O
S
Me
Me
OTf
2v
¹³C{¹H} NMR (101 MHz, CDCl₃)
f1 (ppm)

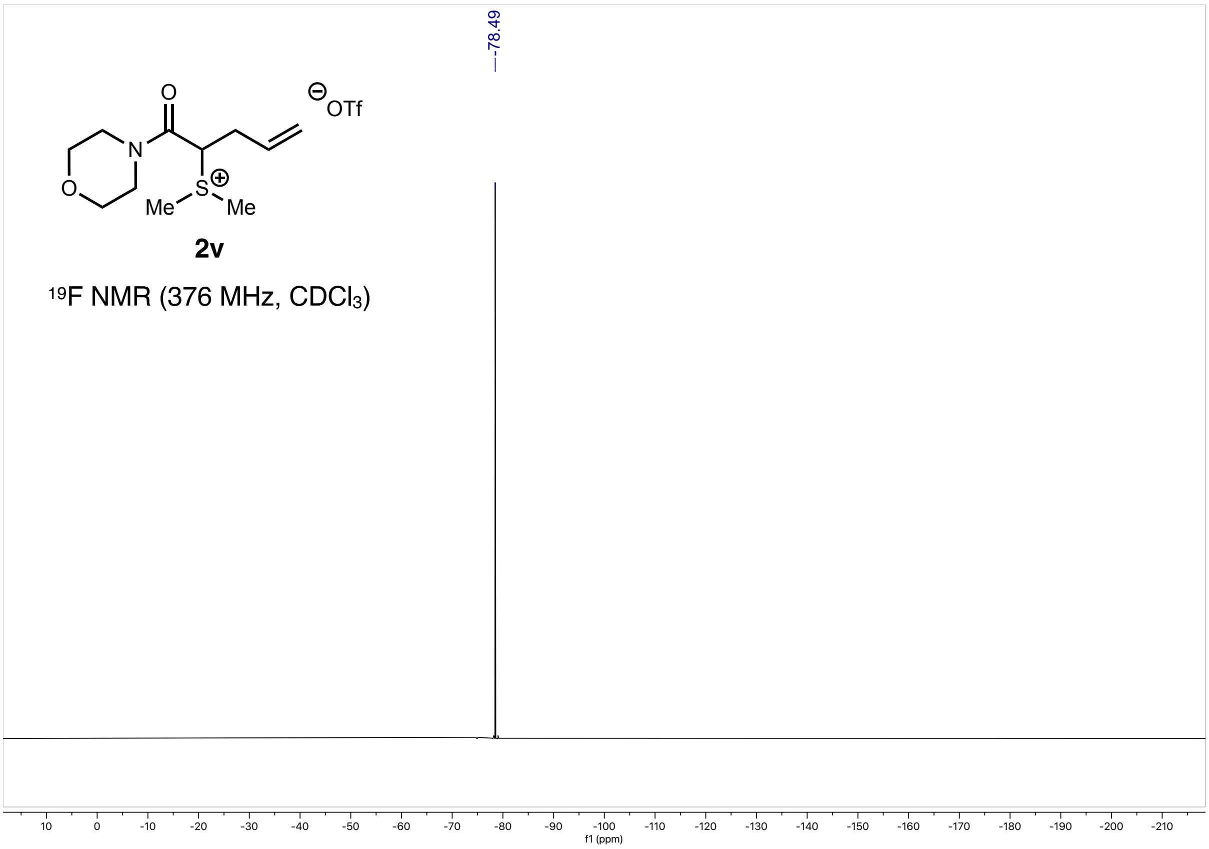

OTf
O
N
O
S
Me
Me
2v
19F NMR (376 MHz, CDCl3)
-78.49
10
0
-10
-20
-30
-40
-50
-60
-70
-80
-90
-100
-110
-120
-130
-140
-150
-160
-170
-180
-190
-200
-210
f1 (ppm)

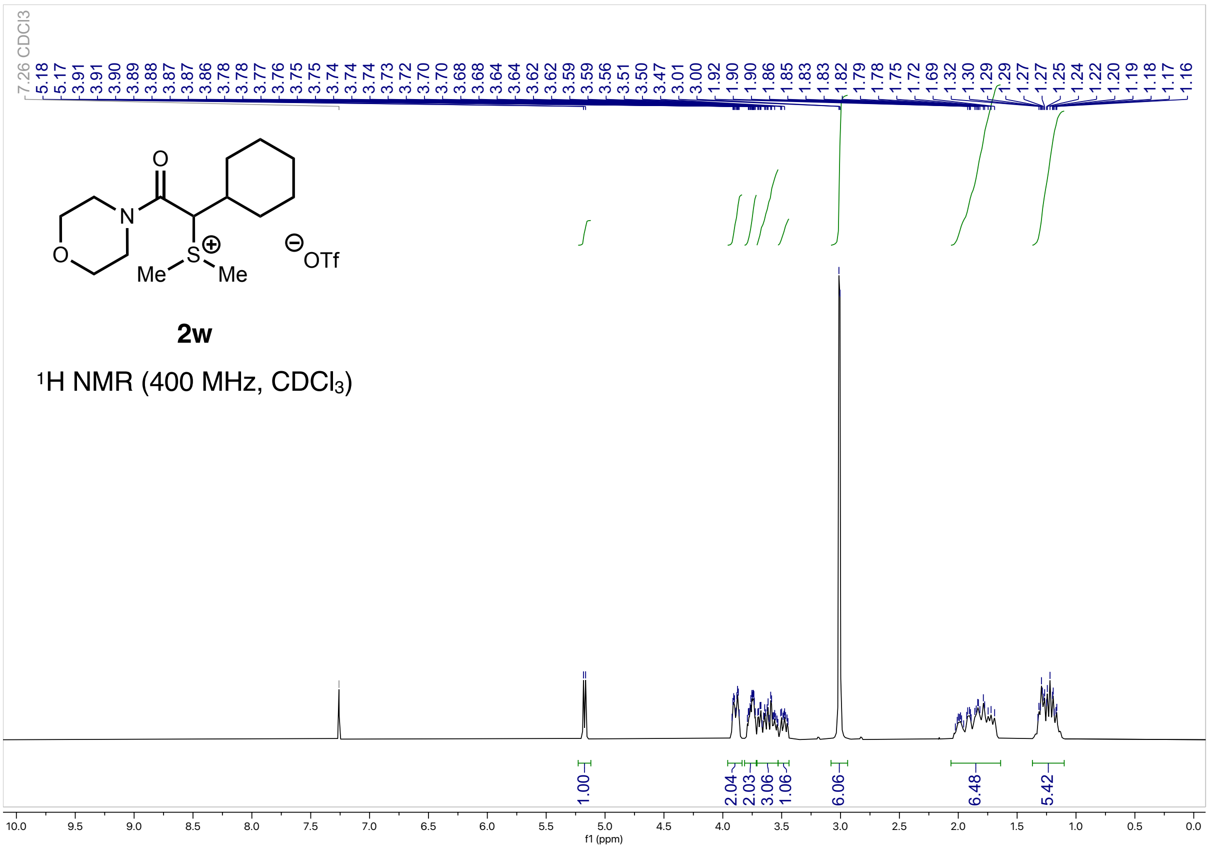

7.26 CDCl3
5.18
5.17
3.91
3.91
3.90
3.89
3.88
3.87
3.87
3.86
3.78
3.78
3.77
3.76
3.75
3.75
3.74
3.74
3.74
3.73
3.72
3.70
3.70
3.68
3.68
3.64
3.64
3.62
3.62
3.59
3.59
3.56
3.51
3.50
3.47
3.01
3.00
1.92
1.90
1.90
1.86
1.85
1.83
1.83
1.82
1.79
1.78
1.75
1.72
1.69
1.32
1.30
1.29
1.29
1.27
1.27
1.25
1.24
1.22
1.20
1.19
1.18
1.17
1.16
O
N
O
S
Me
Me
OTf
2w
^{1}H NMR (400 MHz, $CDCl_3$)
1.00
2.04
2.03
3.06
1.06
6.06
6.48
5.42
f1 (ppm)

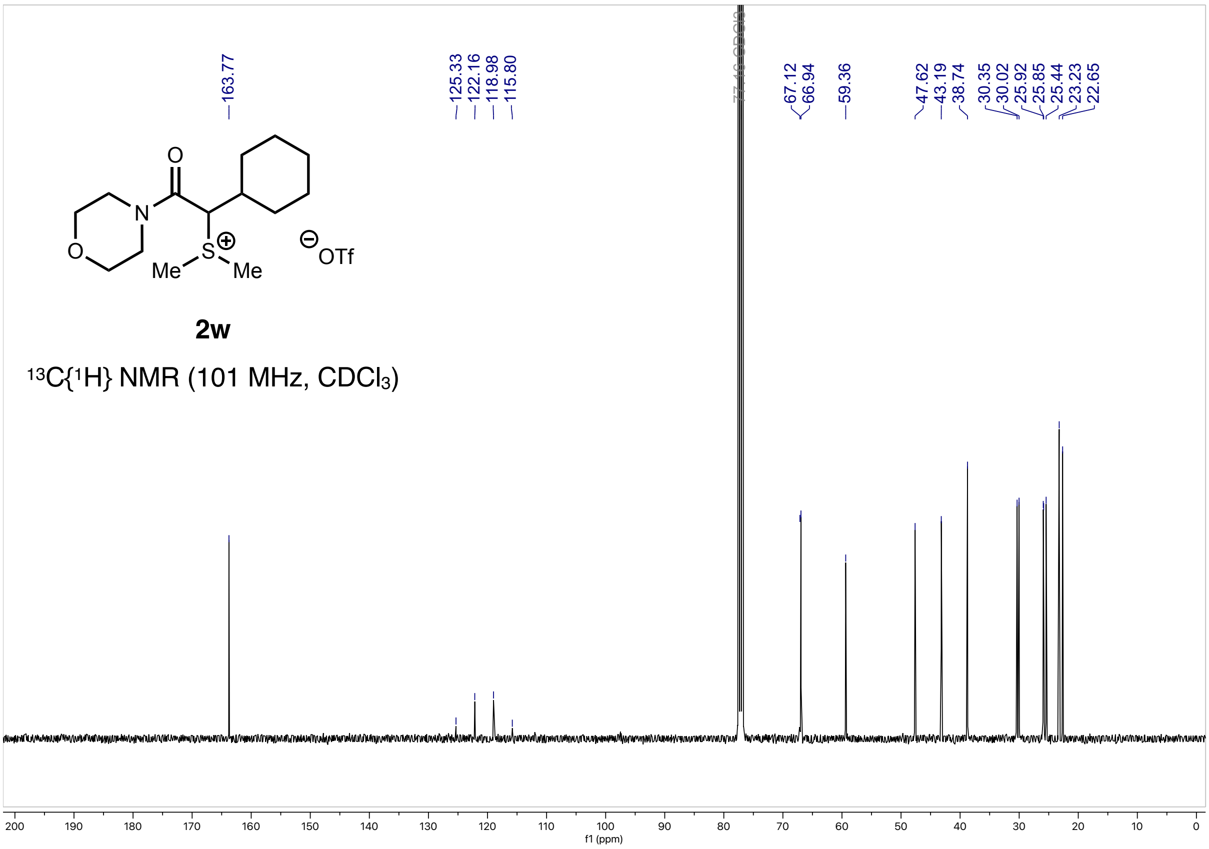

163.77
125.33
122.16
118.98
115.80
77.16 CDCl3
67.12
66.94
59.36
47.62
43.19
38.74
30.35
30.02
25.92
25.85
25.44
23.23
22.65
O
N
O
S
Me
Me
OTf
2w
$^{13}C\{^1H\}$ NMR (101 MHz, $CDCl_3$)
f1 (ppm)

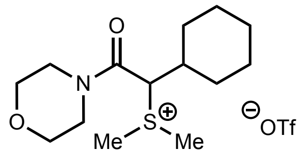

2w

^{19}F NMR (376 MHz, $CDCl_3$)

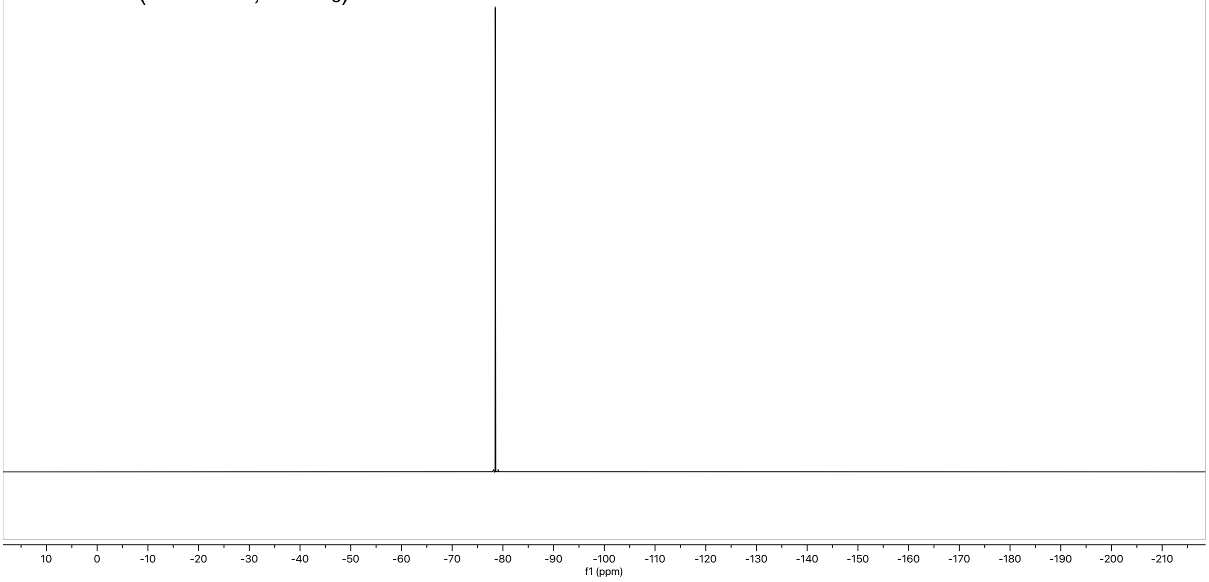

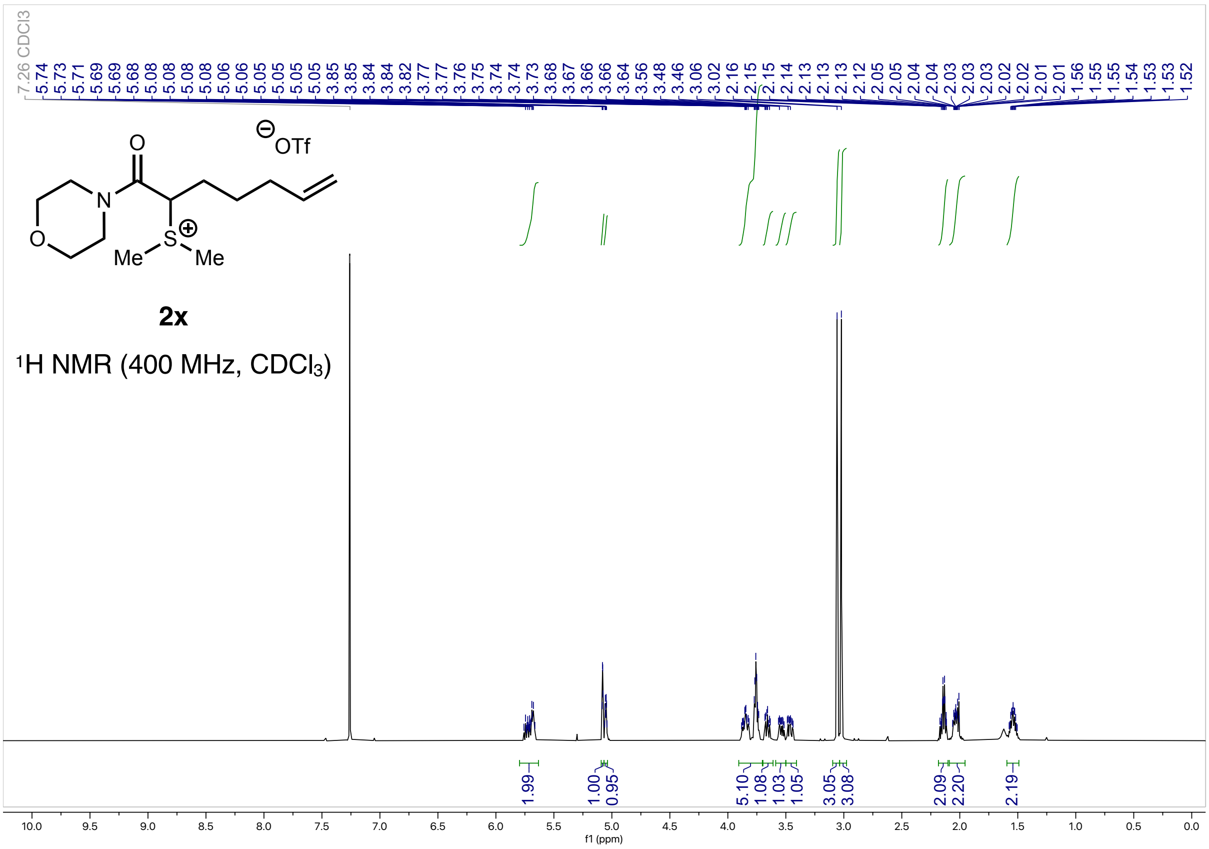

7.26 CDCl3
5.74
5.73
5.71
5.69
5.69
5.68
5.08
5.08
5.08
5.08
5.06
5.06
5.05
5.05
5.05
5.05
3.85
3.85
3.84
3.84
3.82
3.77
3.76
3.76
3.75
3.74
3.74
3.73
3.68
3.67
3.66
3.66
3.64
3.56
3.48
3.46
3.06
3.02
2.16
2.15
2.15
2.14
2.13
2.13
2.13
2.12
2.05
2.05
2.04
2.04
2.03
2.03
2.03
2.02
2.02
2.01
1.56
1.55
1.55
1.54
1.53
1.53
1.52
O
N
O
S
Me
Me
OTf
2x
1H NMR (400 MHz, CDCl3)
1.99
1.00
0.95
5.10
1.08
1.03
1.05
3.05
3.08
2.09
2.20
2.19
f1 (ppm)

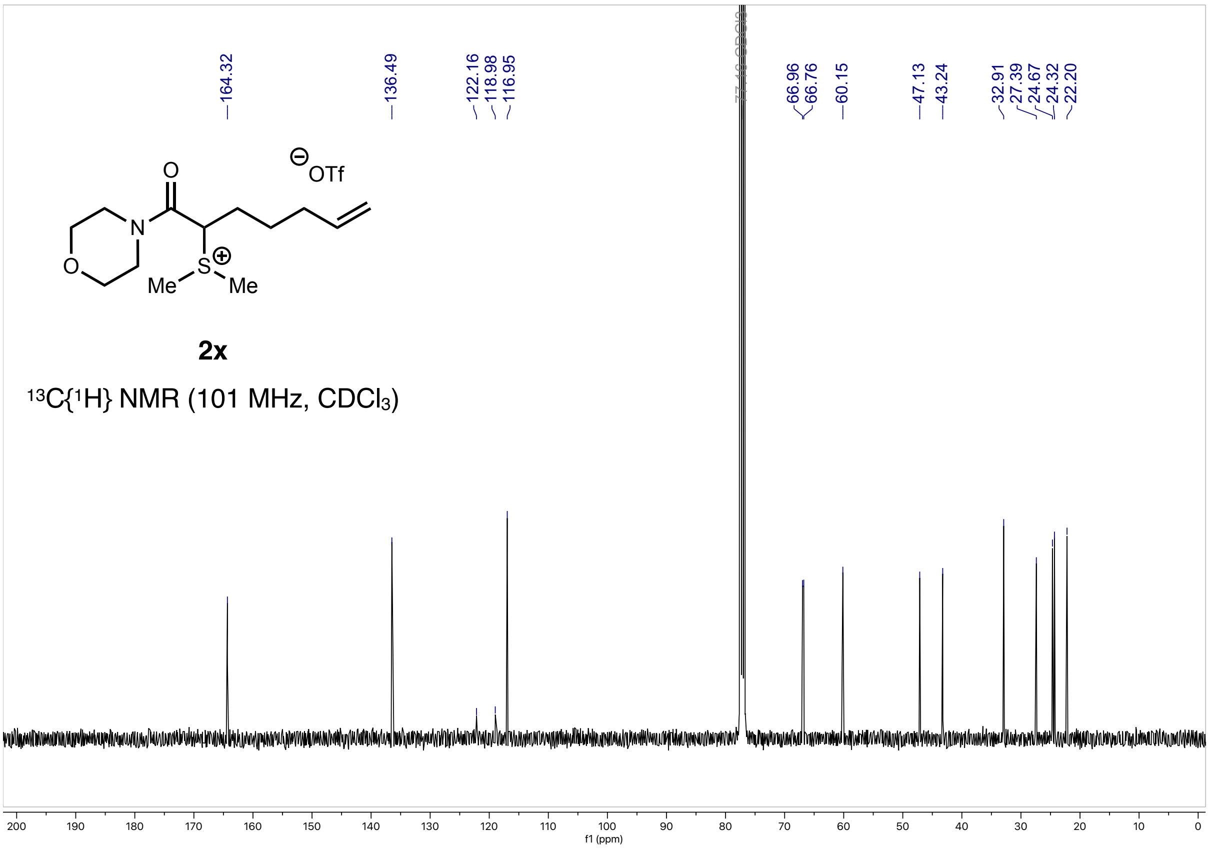

164.32
136.49
122.16
118.98
116.95
66.96
66.76
60.15
47.13
43.24
32.91
27.39
24.67
24.32
22.20
O
N
O
S
Me
Me
OTf
2x
13C{1H} NMR (101 MHz, CDCl3)
f1 (ppm)

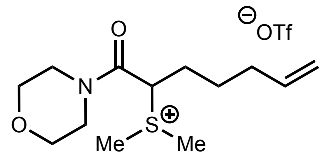

2x

^{19}F NMR (376 MHz, $CDCl_3$)

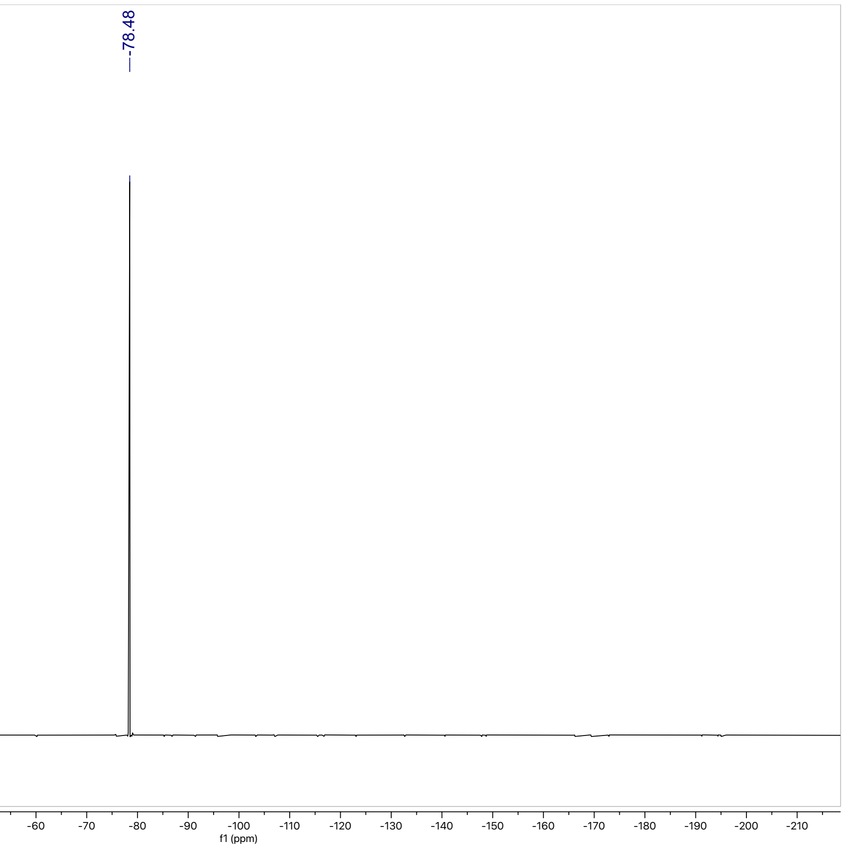

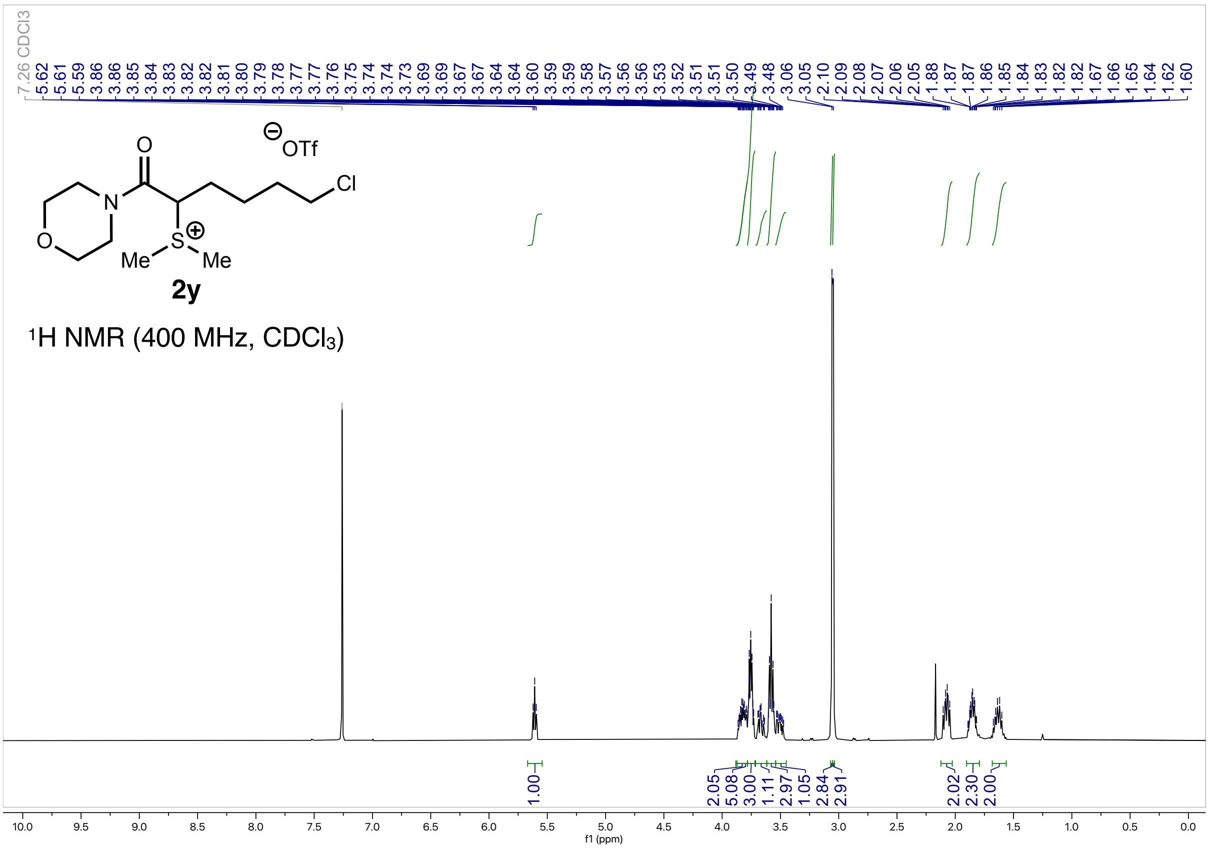
7.26 CDCl3
5.62
5.61
5.59
3.86
3.86
3.85
3.84
3.83
3.82
3.82
3.81
3.80
3.79
3.78
3.77
3.77
3.76
3.75
3.74
3.74
3.73
3.69
3.69
3.67
3.67
3.64
3.64
3.60
3.59
3.59
3.58
3.57
3.56
3.56
3.53
3.52
3.51
3.51
3.50
3.49
3.48
3.06
3.05
2.10
2.09
2.08
2.07
2.06
2.05
1.88
1.87
1.87
1.86
1.85
1.84
1.83
1.82
1.82
1.67
1.66
1.65
1.64
1.62
1.60
O
⊖OTf
N
Cl
O
S⊕
Me
Me
2y
1H NMR (400 MHz, CDCl3)
1.00
2.05
5.08
3.00
1.11
2.97
1.05
2.84
2.91
2.02
2.30
2.00
f1 (ppm)

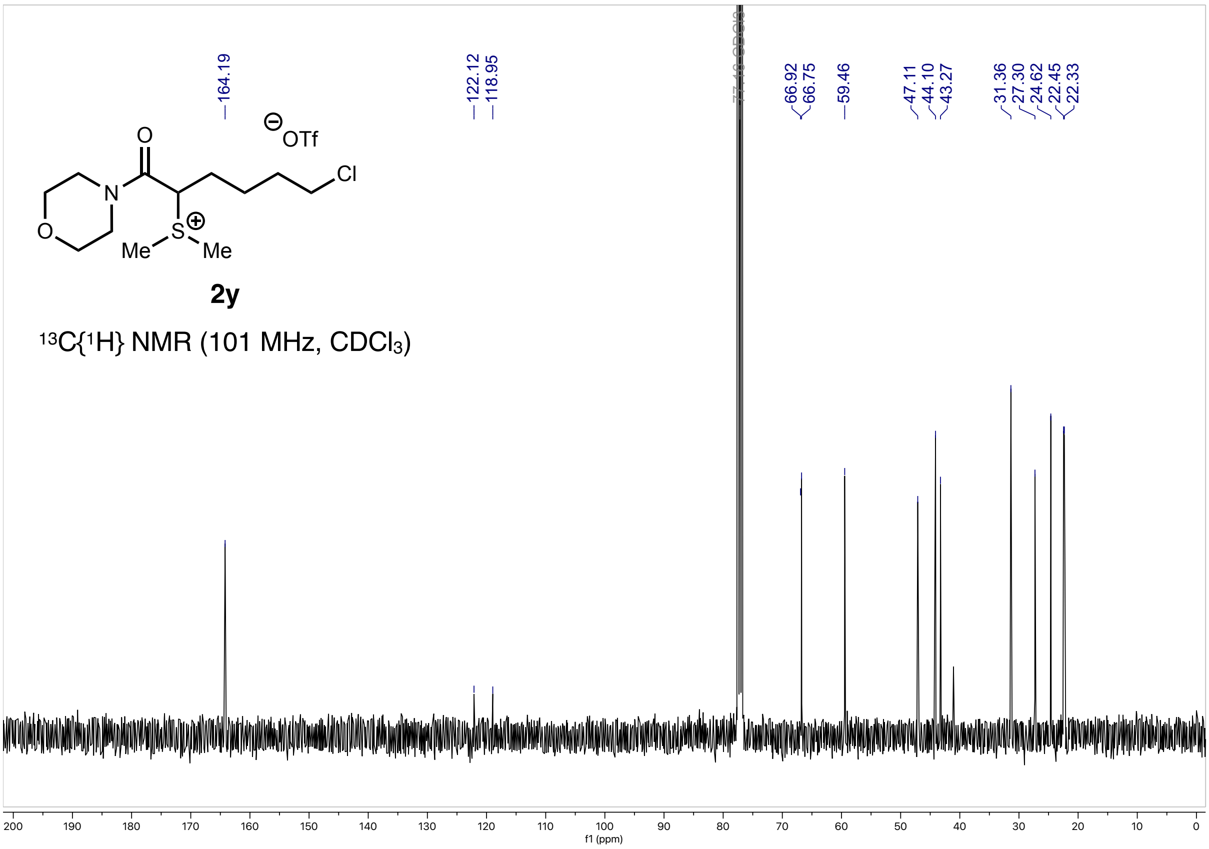
164.19
122.12
118.95
66.92
66.75
59.46
47.11
44.10
43.27
31.36
27.30
24.62
22.45
22.33
O
⊖OTf
N
Cl
O
S⊕
Me
Me
2y
13C{1H} NMR (101 MHz, CDCl3)
f1 (ppm)

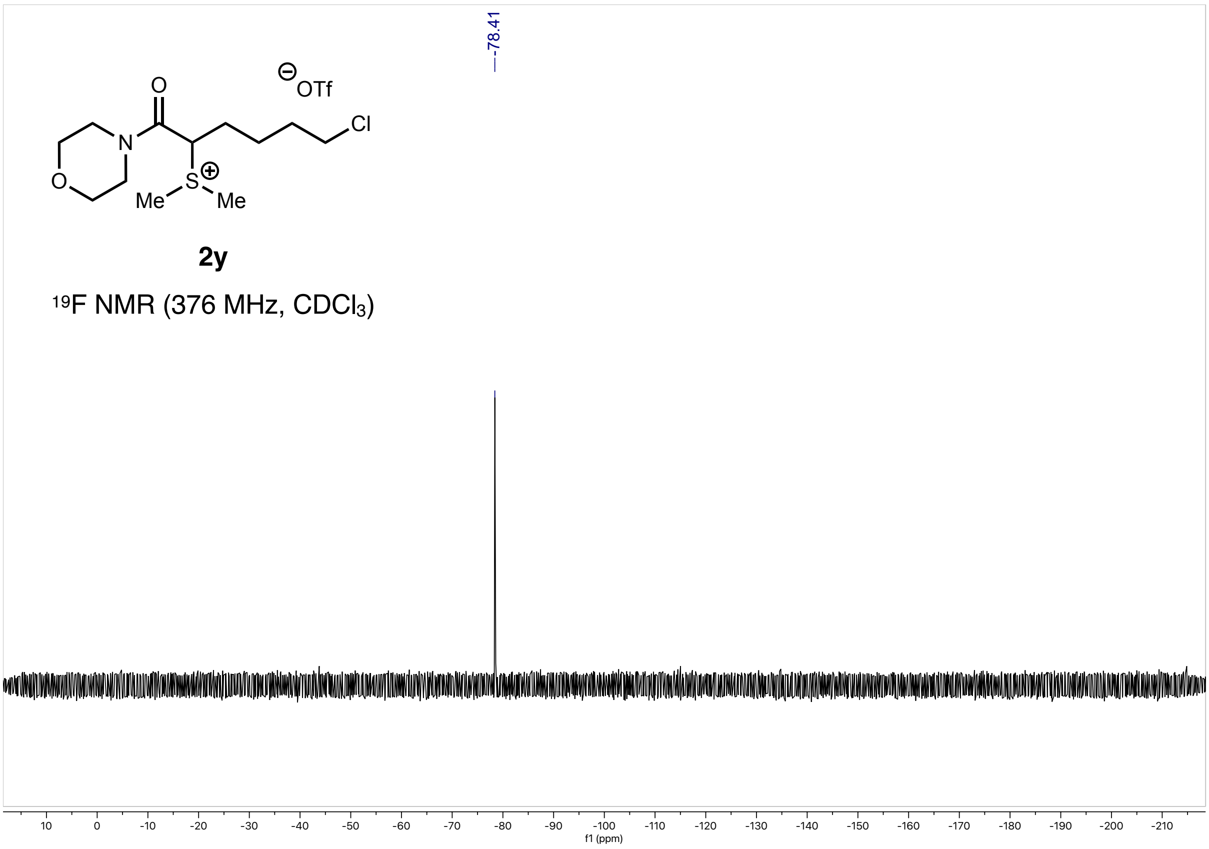

-78.41
O
OTf
N
Cl
O
S
Me
Me
2y
^{19}F NMR (376 MHz, $CDCl_3$)
10
0
-10
-20
-30
-40
-50
-60
-70
-80
-90
-100
-110
-120
-130
-140
-150
-160
-170
-180
-190
-200
-210
f1 (ppm)

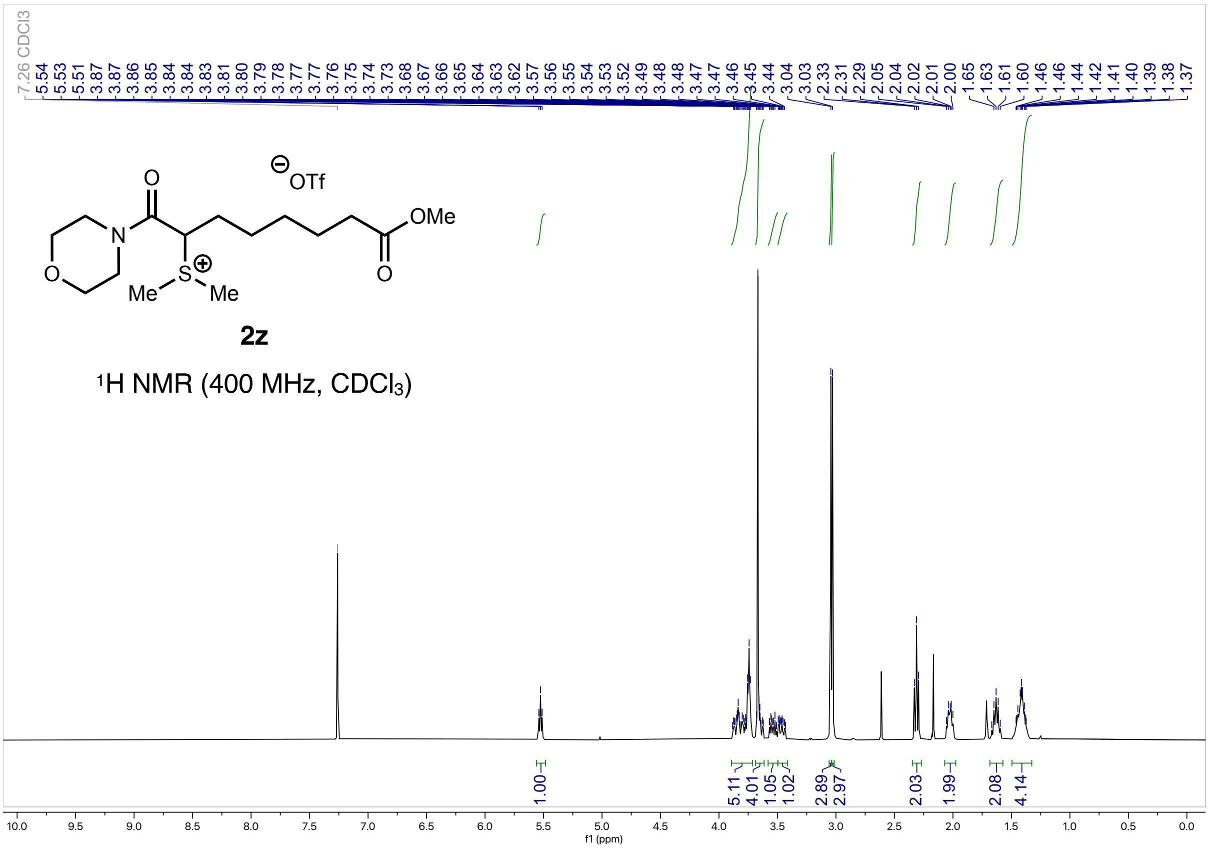

7.26 CDCl3
5.54
5.53
5.51
3.87
3.87
3.86
3.85
3.84
3.84
3.83
3.81
3.80
3.79
3.78
3.77
3.77
3.76
3.75
3.74
3.73
3.68
3.67
3.66
3.65
3.64
3.63
3.62
3.57
3.56
3.55
3.54
3.53
3.52
3.49
3.48
3.48
3.47
3.46
3.45
3.44
3.04
3.03
2.33
2.31
2.29
2.05
2.04
2.02
2.01
2.00
1.65
1.63
1.61
1.60
1.46
1.44
1.42
1.41
1.40
1.39
1.38
1.37
OTf
OMe
O
N
S
Me
Me
2z
1H NMR (400 MHz, CDCl3)
1.00
5.11
4.01
1.05
1.02
2.89
2.97
2.03
1.99
2.08
4.14
f1 (ppm)

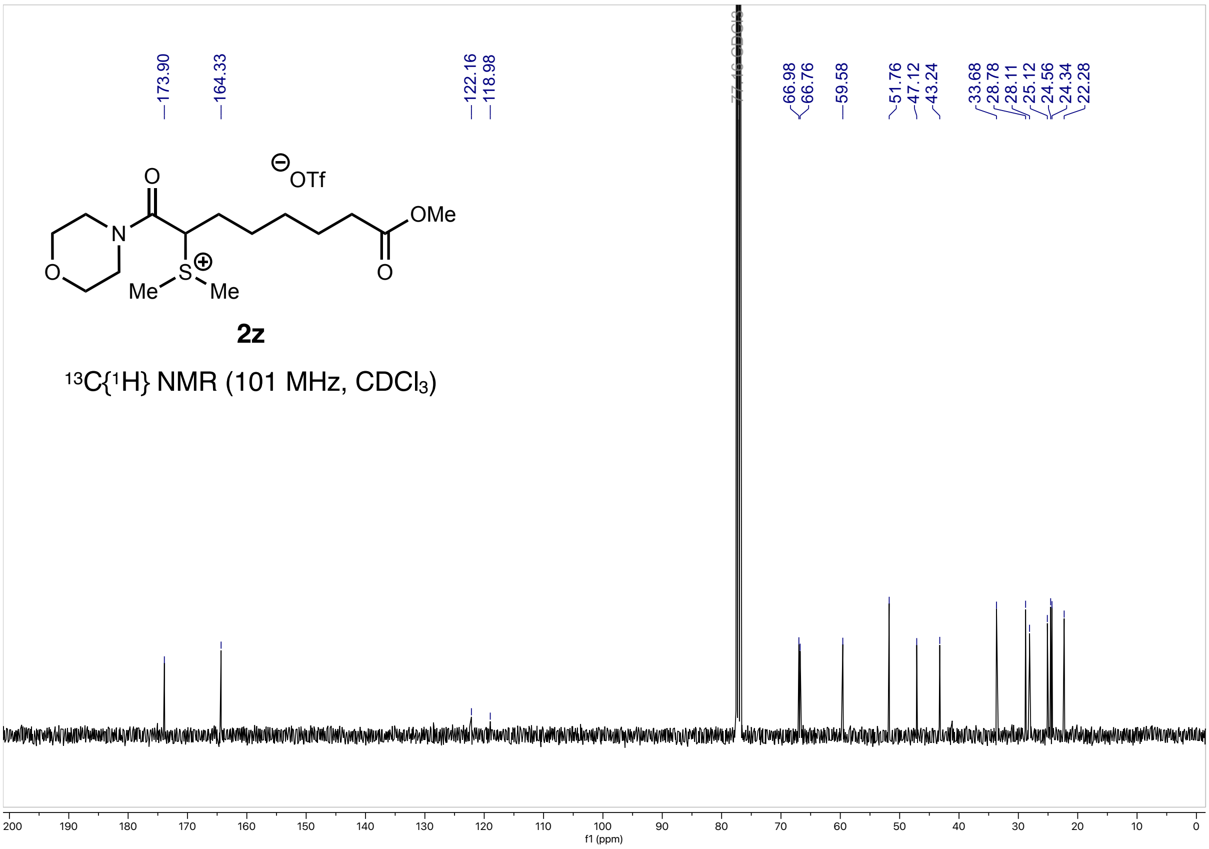

173.90
164.33
122.16
118.98
77.16 CDCl3
66.98
66.76
59.58
51.76
47.12
43.24
33.68
28.78
28.11
25.12
24.56
24.34
22.28
OTf
OMe
O
N
S
Me
Me
2z
13C{1H} NMR (101 MHz, CDCl3)
f1 (ppm)

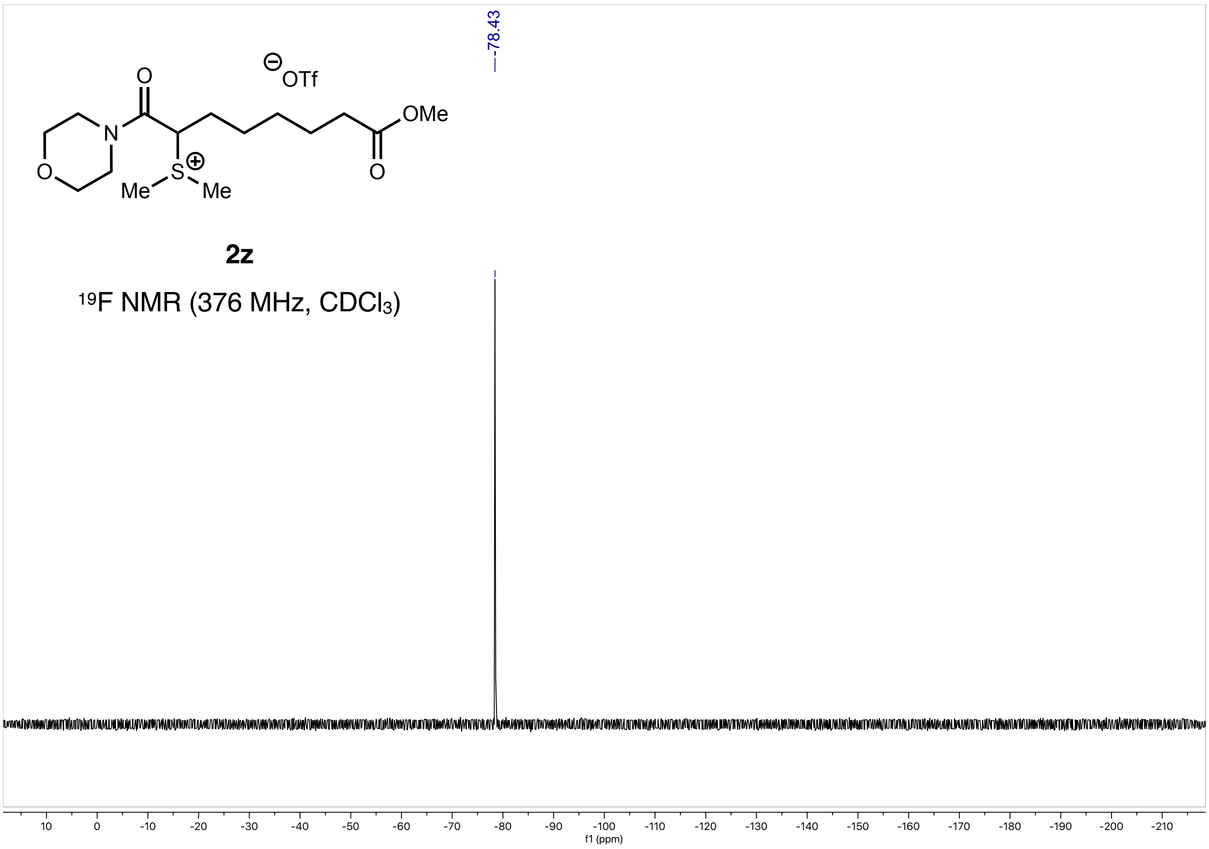
OTf
O
N
O
S
Me
Me
OMe
O
2z
19F NMR (376 MHz, CDCl3)
-78.43
f1 (ppm)

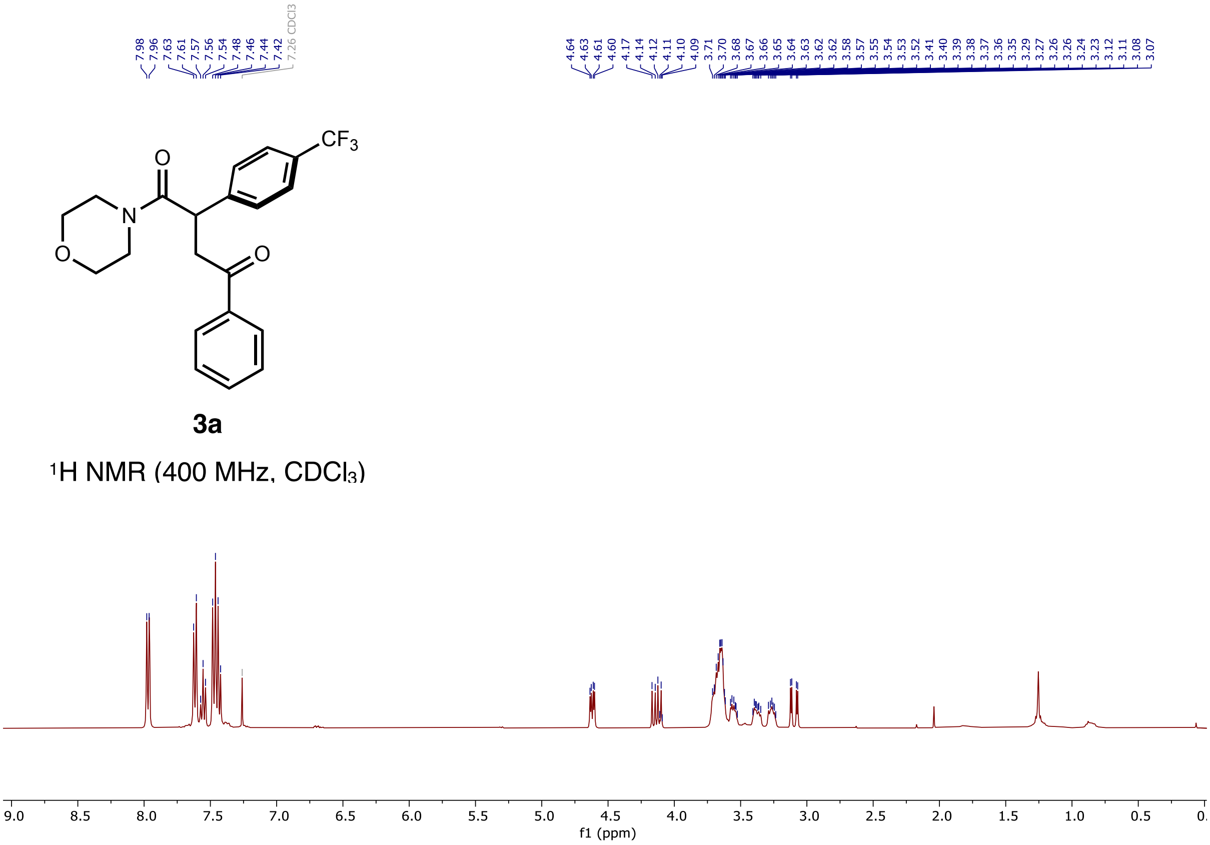

3a

^{1}H NMR (400 MHz, $CDCl_3$)

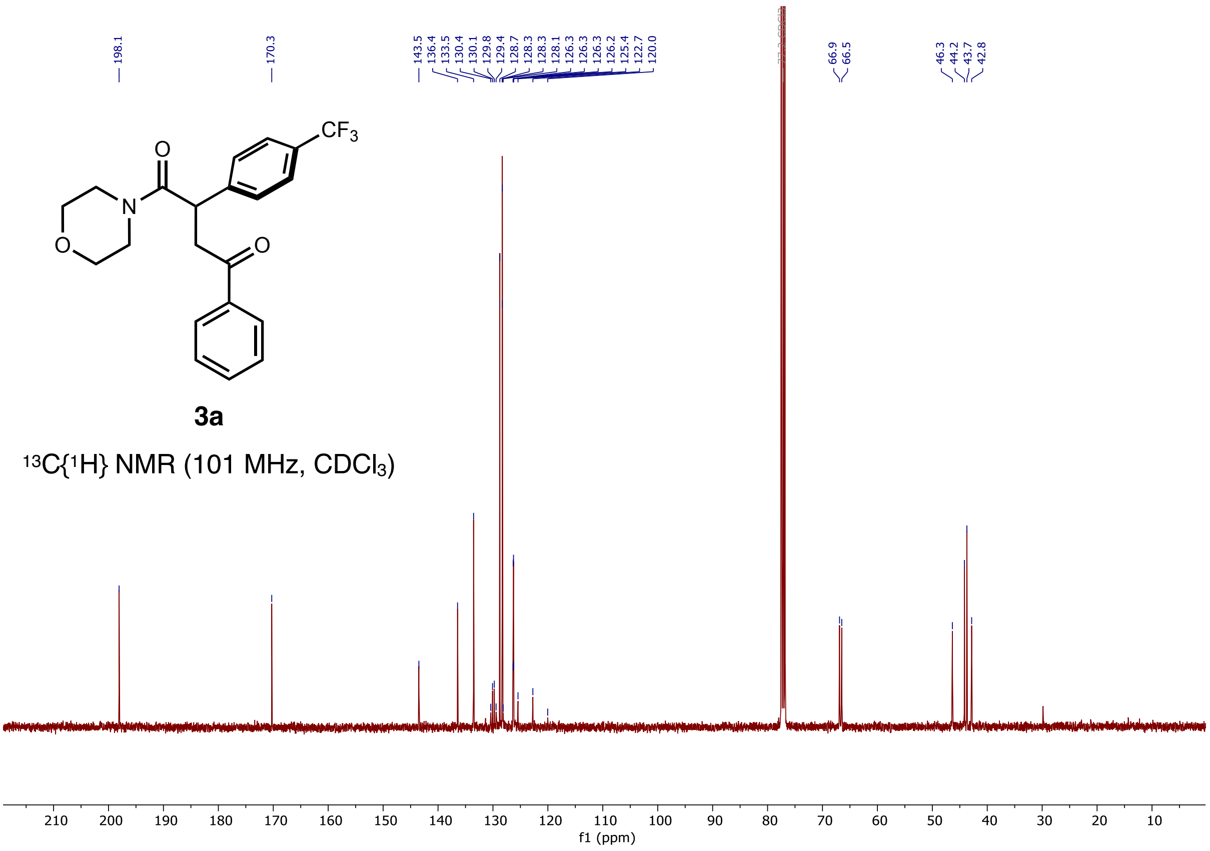

3a

$^{13}C\{^1H\}$ NMR (101 MHz, $CDCl_3$)

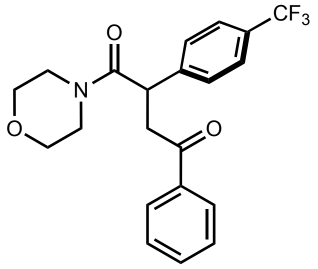

3a

^{19}F NMR (376 MHz, CDCl$_3$)

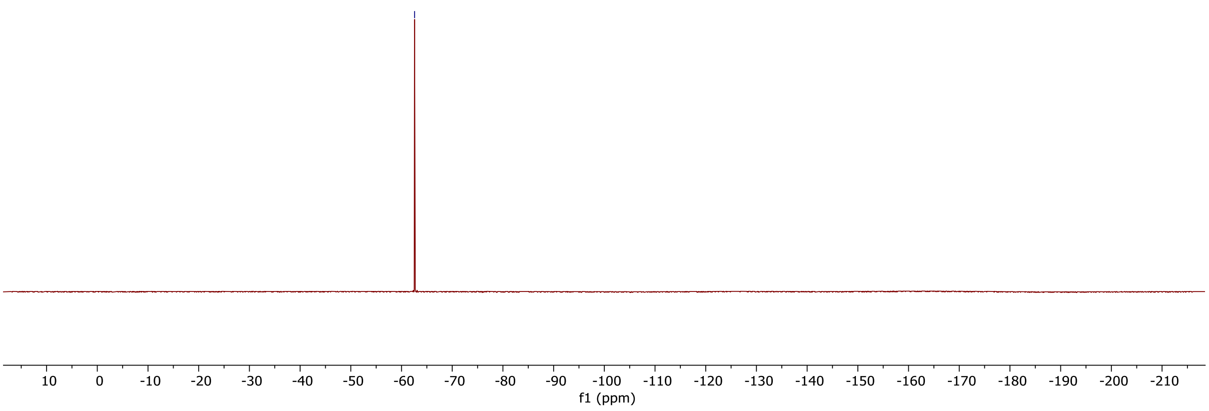

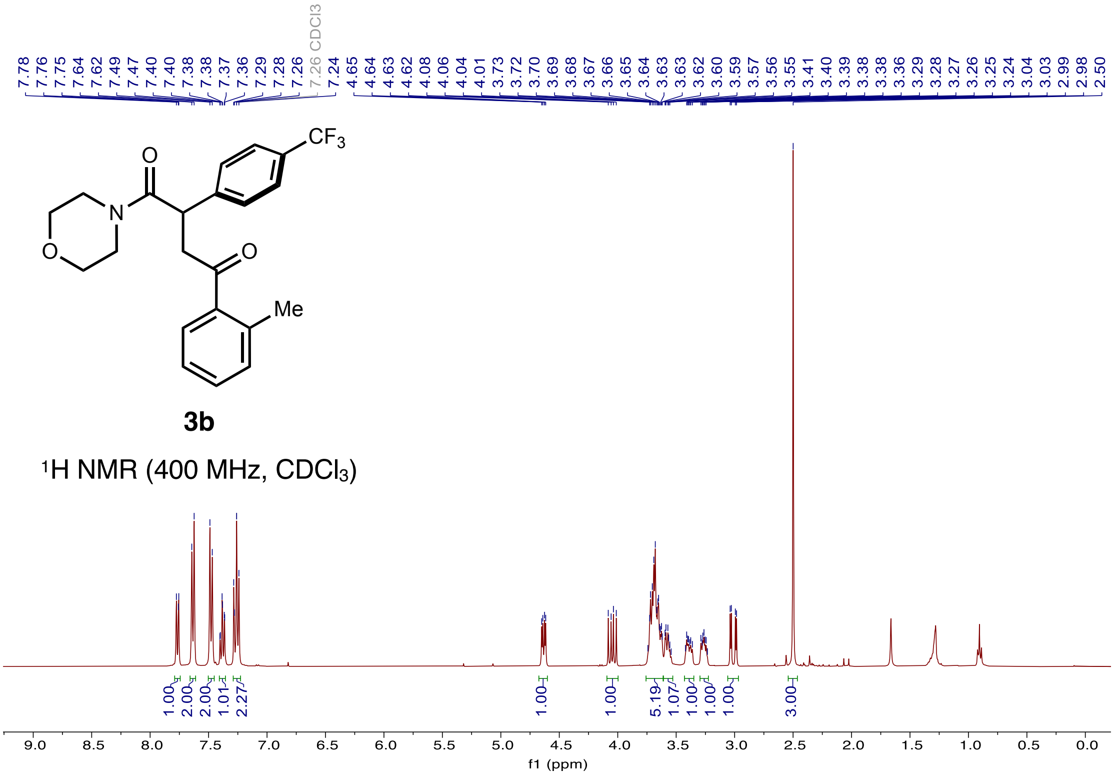

7.78 7.76 7.75 7.64 7.62 7.49 7.47 7.40 7.40 7.38 7.38 7.37 7.36 7.29 7.28 7.26 7.26 CDCl3 7.24 4.65 4.64 4.63 4.62 4.08 4.06 4.04 4.01 3.73 3.72 3.70 3.69 3.68 3.67 3.66 3.65 3.64 3.63 3.63 3.62 3.60 3.59 3.57 3.56 3.55 3.41 3.40 3.39 3.38 3.38 3.36 3.29 3.28 3.27 3.26 3.25 3.24 3.04 3.03 2.99 2.98 2.50
CF3
O
N
O
O
Me
3b
1H NMR (400 MHz, CDCl3)
1.00 2.00 2.00 1.01 2.27 1.00 1.00 5.19 1.07 1.00 1.00 1.00 3.00
9.0 8.5 8.0 7.5 7.0 6.5 6.0 5.5 5.0 4.5 4.0 3.5 3.0 2.5 2.0 1.5 1.0 0.5 0.0
f1 (ppm)

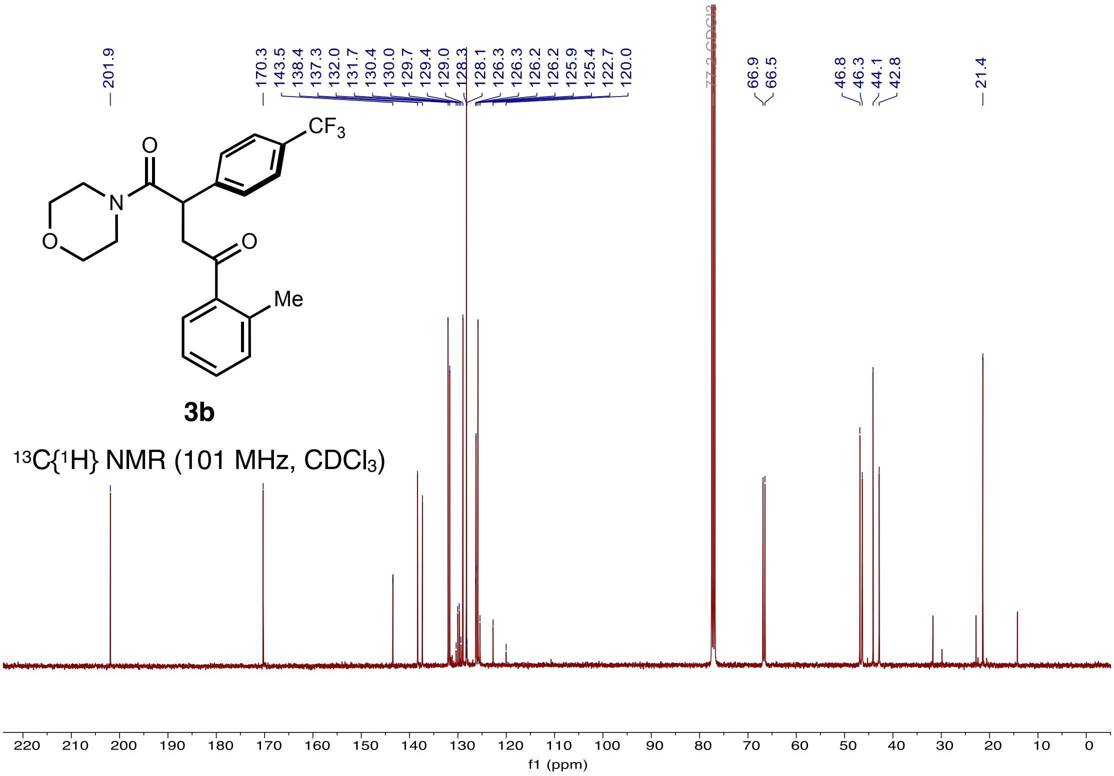

201.9
170.3 143.5 138.4 137.3 132.0 131.7 130.4 130.0 129.7 129.4 129.0 128.3 128.1 126.3 126.3 126.2 126.2 125.9 125.4 122.7 120.0
77.2 CDCl3
66.9 66.5
46.8 46.3 44.1 42.8
21.4
CF3
O
N
O
O
Me
3b
13C{1H} NMR (101 MHz, CDCl3)
220 210 200 190 180 170 160 150 140 130 120 110 100 90 80 70 60 50 40 30 20 10 0
f1 (ppm)

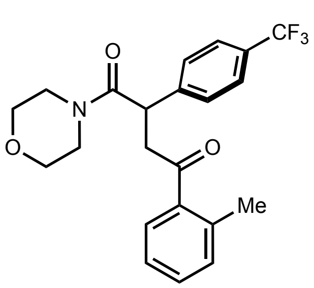

3b

^{19}F NMR (376 MHz, $CDCl_3$)

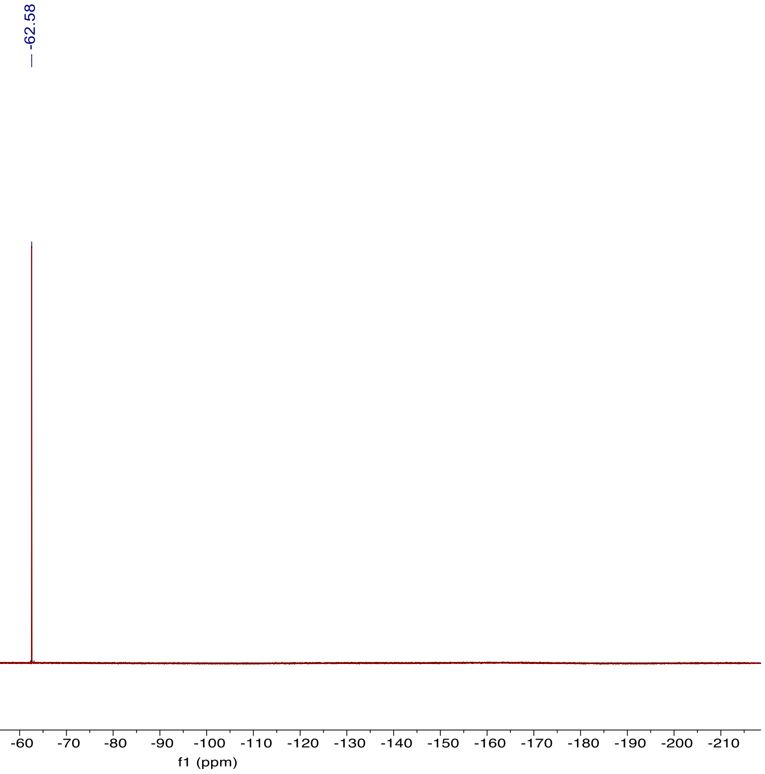

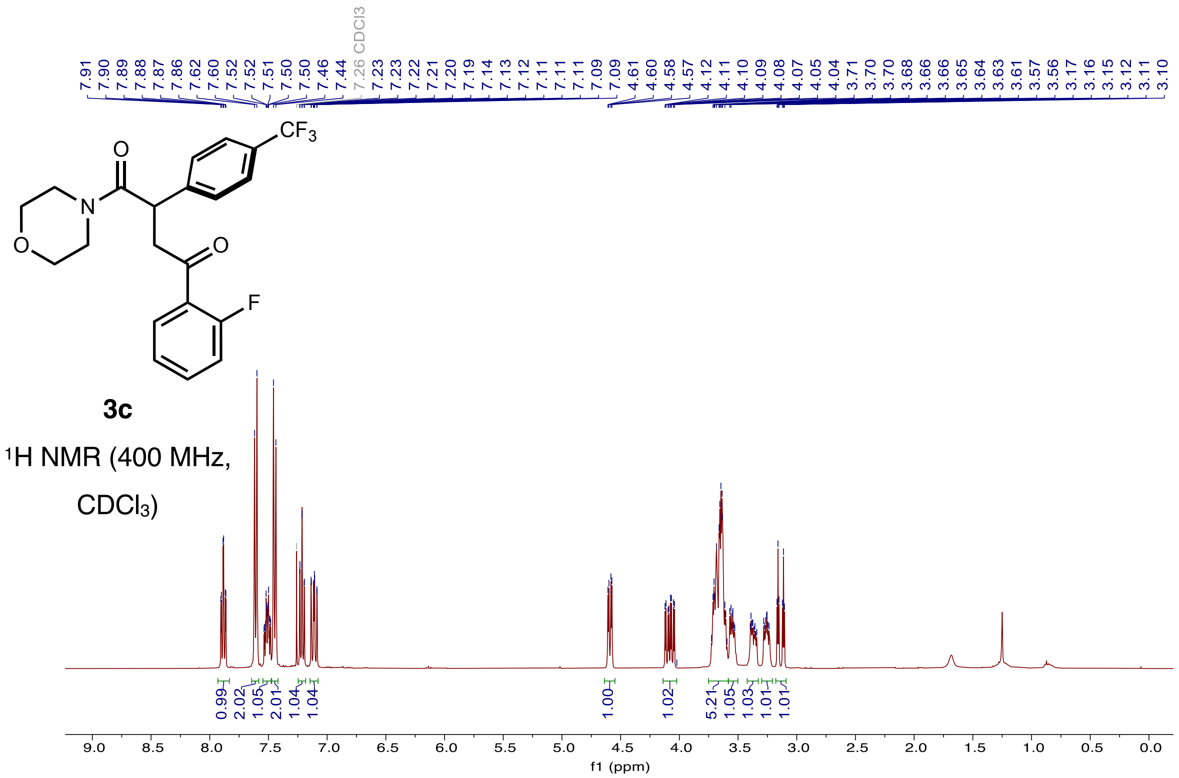

3c

^{1}H NMR (400 MHz, CDCl$_3$)

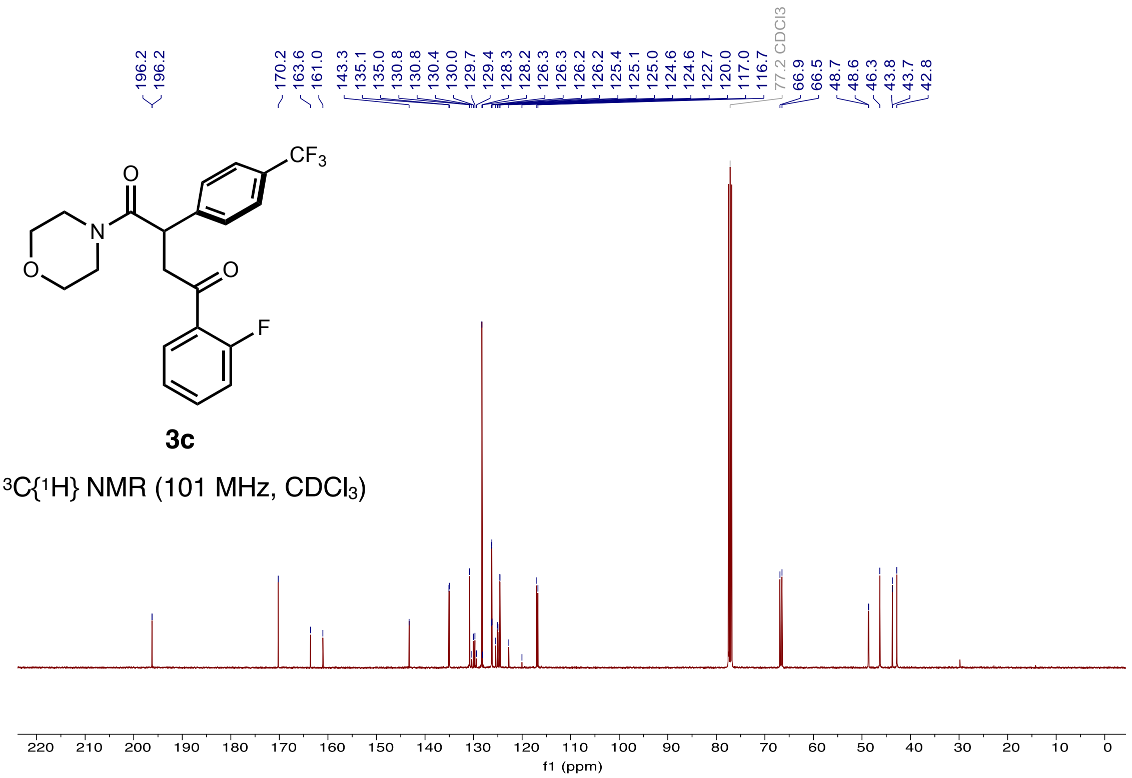

3c

^{13}C{^{1}H} NMR (101 MHz, CDCl$_3$)

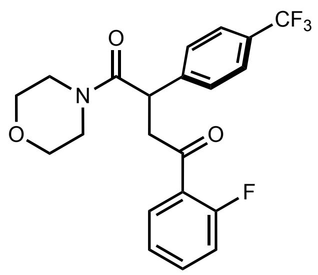

3c

^{19}F NMR (376 MHz, $CDCl_3$)

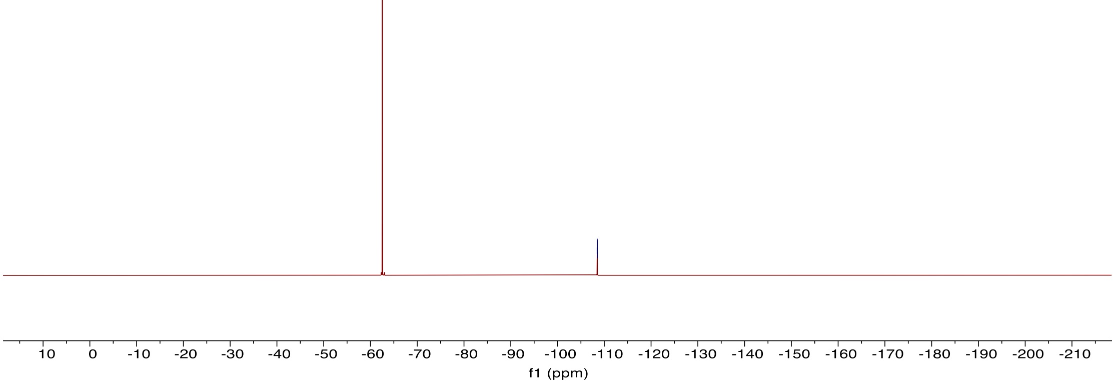

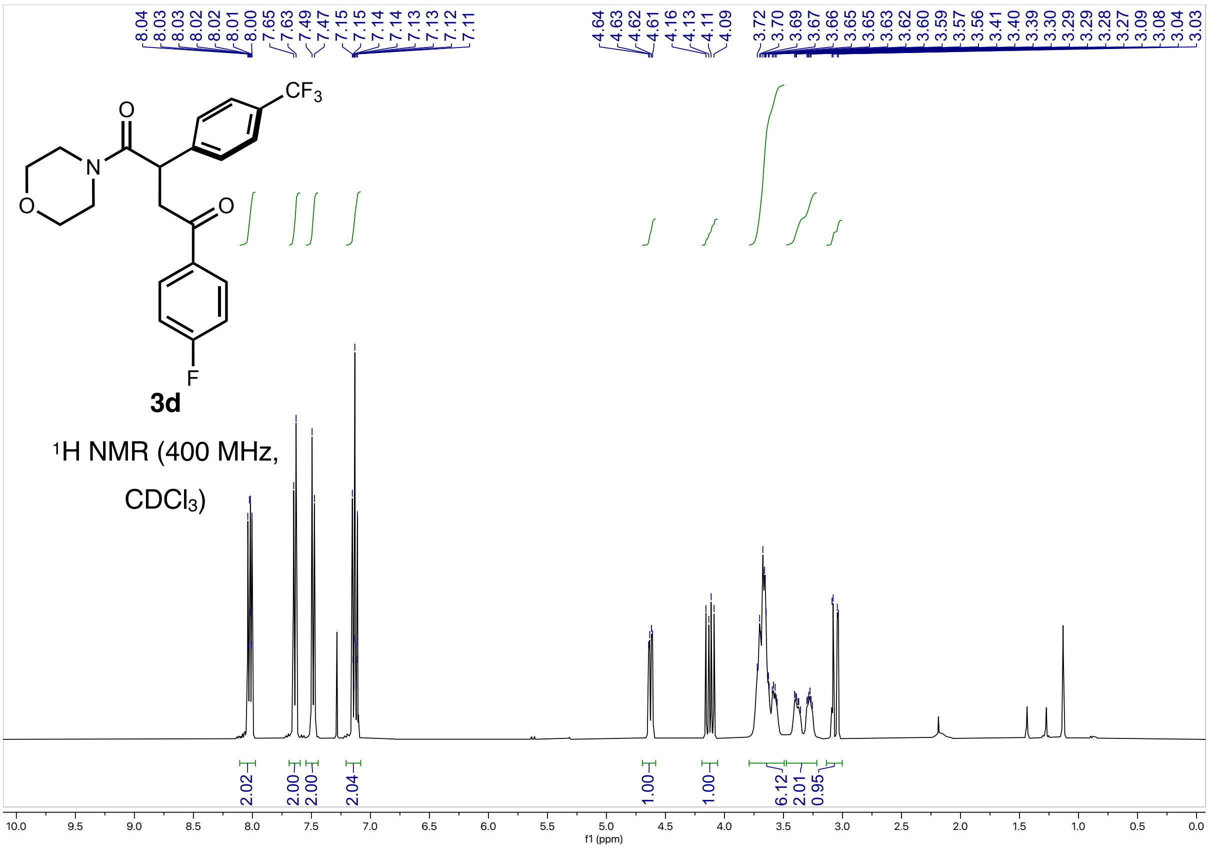

8.04
8.03
8.03
8.02
8.02
8.01
8.00
7.65
7.63
7.49
7.47
7.15
7.15
7.14
7.14
7.13
7.13
7.12
7.11
4.64
4.63
4.62
4.61
4.16
4.13
4.11
4.09
3.72
3.70
3.69
3.67
3.66
3.65
3.65
3.63
3.62
3.60
3.59
3.57
3.56
3.41
3.40
3.39
3.30
3.29
3.29
3.28
3.27
3.09
3.08
3.04
3.03
O
N
O
O
CF3
F
3d
1H NMR (400 MHz, CDCl3)
2.02
2.00
2.00
2.04
1.00
1.00
6.12
2.01
0.95
f1 (ppm)

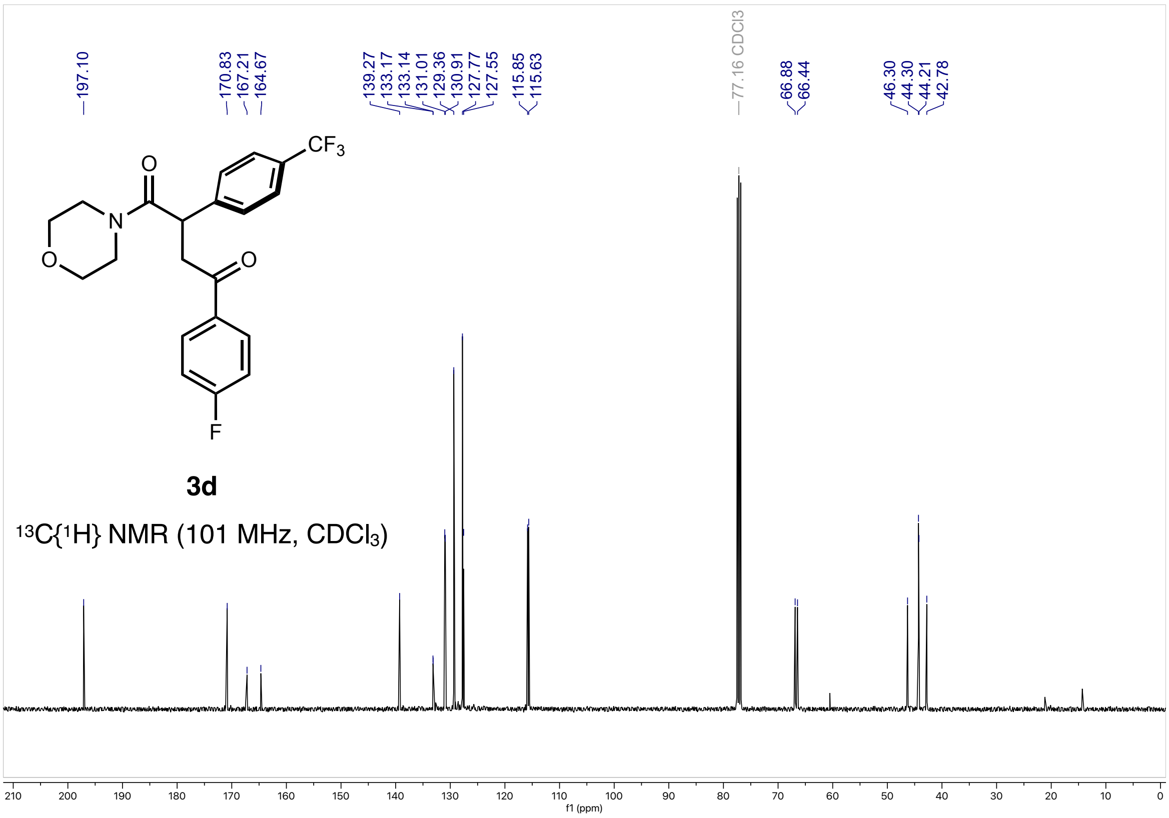

197.10
170.83
167.21
164.67
139.27
133.17
133.14
131.01
129.36
130.91
127.77
127.55
115.85
115.63
77.16 CDCl3
66.88
66.44
46.30
44.30
44.21
42.78
O
N
O
O
CF3
F
3d
13C{1H} NMR (101 MHz, CDCl3)
f1 (ppm)

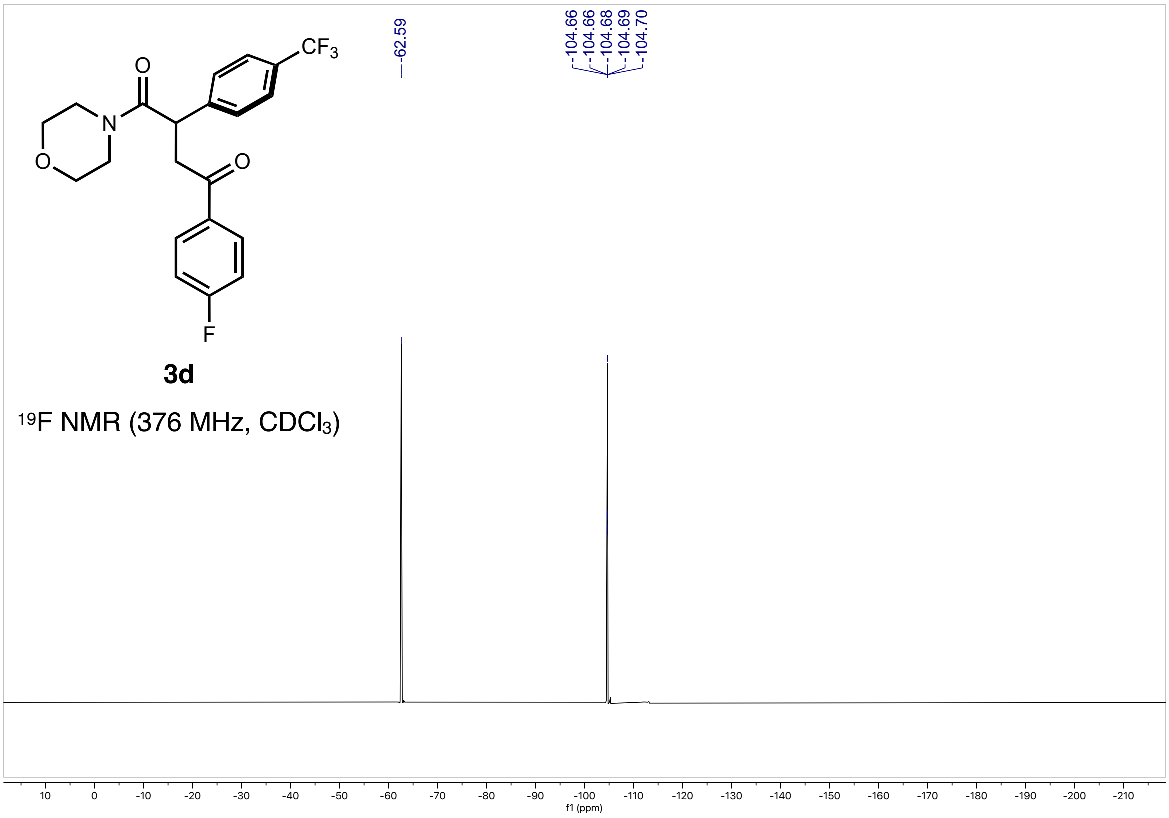
-62.59
-104.66
-104.66
-104.68
-104.69
-104.70
CF3
O
N
O
O
F
3d
19F NMR (376 MHz, CDCl3)
10
0
-10
-20
-30
-40
-50
-60
-70
-80
-90
-100
-110
-120
-130
-140
-150
-160
-170
-180
-190
-200
-210
f1 (ppm)

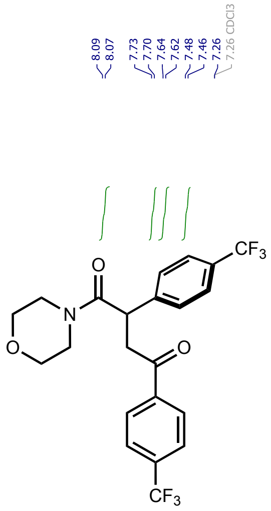

3e

^{1}H NMR (400 MHz, $CDCl_3$)

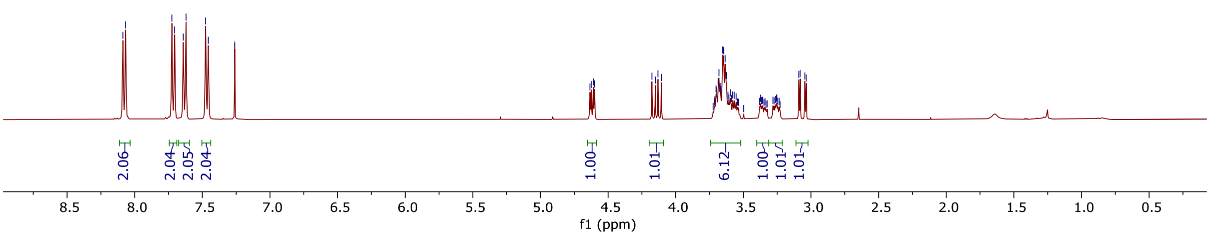

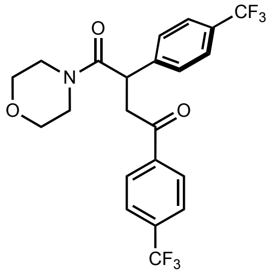

3e

^{13}C NMR (101 MHz, $CDCl_3$)

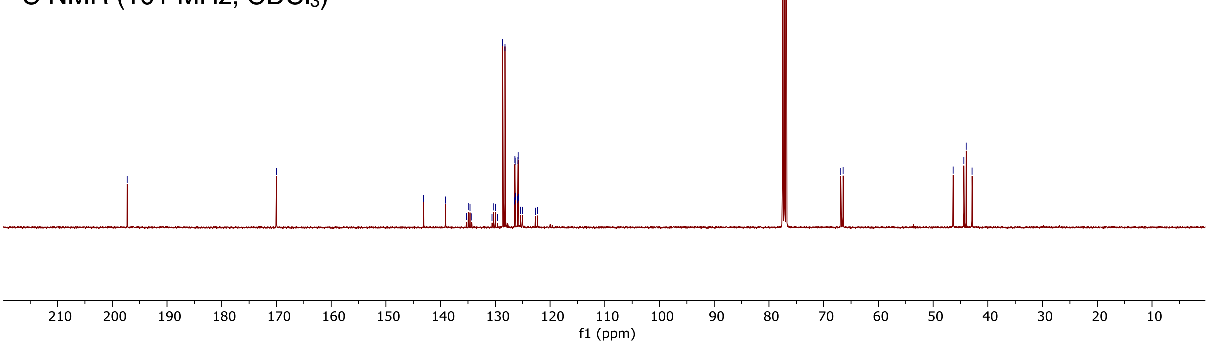

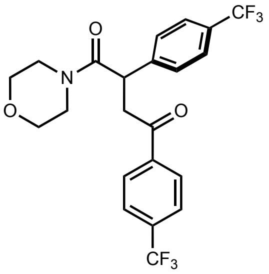

3e

^{19}F NMR (376 MHz, $CDCl_3$)

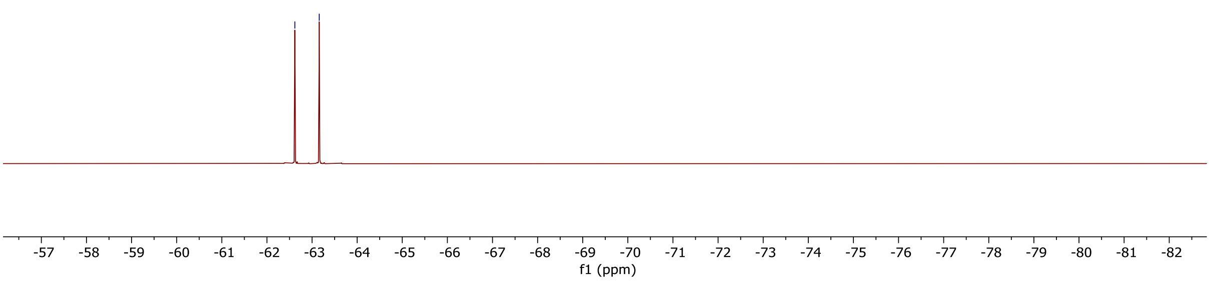

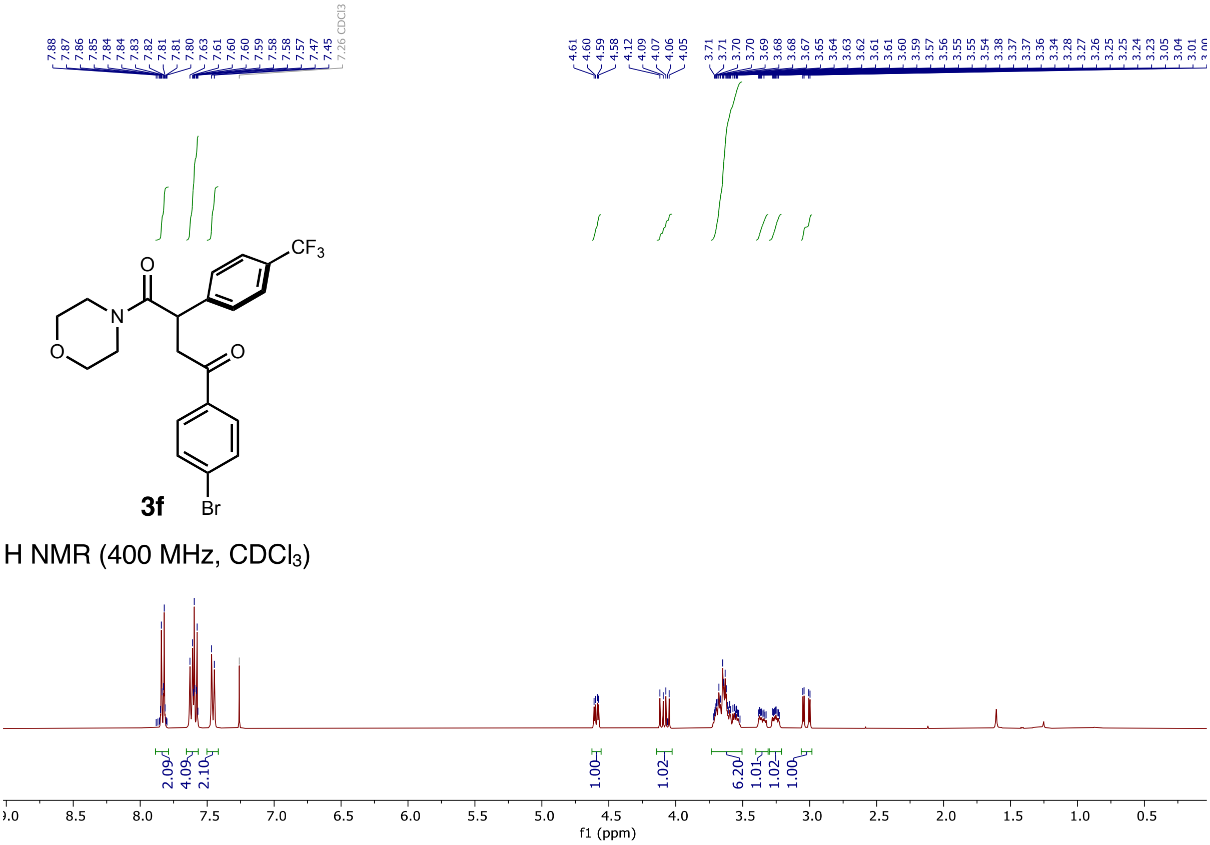

3f

^{1}H NMR (400 MHz, $CDCl_3$)

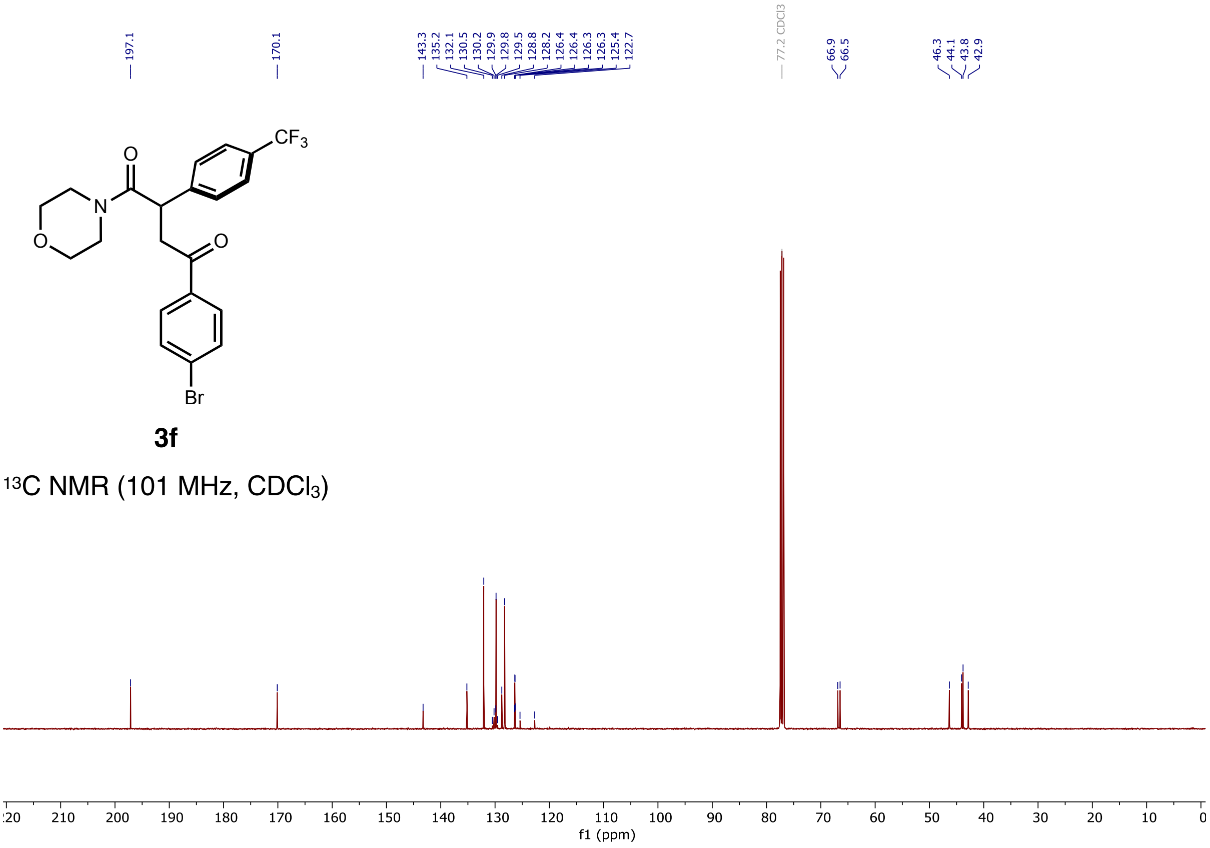

3f

^{13}C NMR (101 MHz, $CDCl_3$)

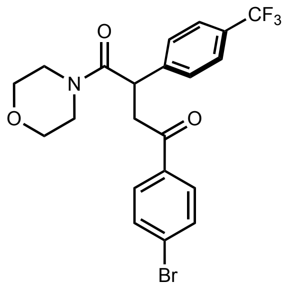

3f

^{19}F NMR (376 MHz, $CDCl_3$)

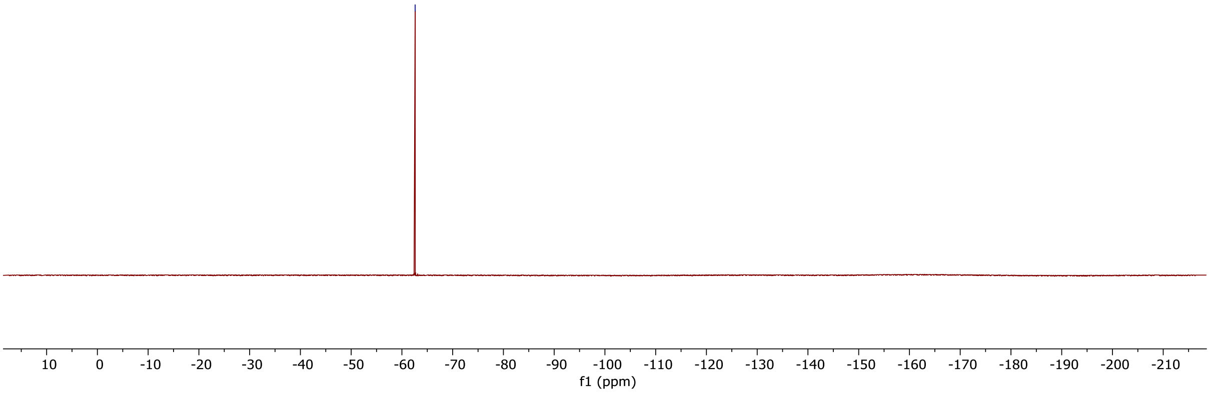

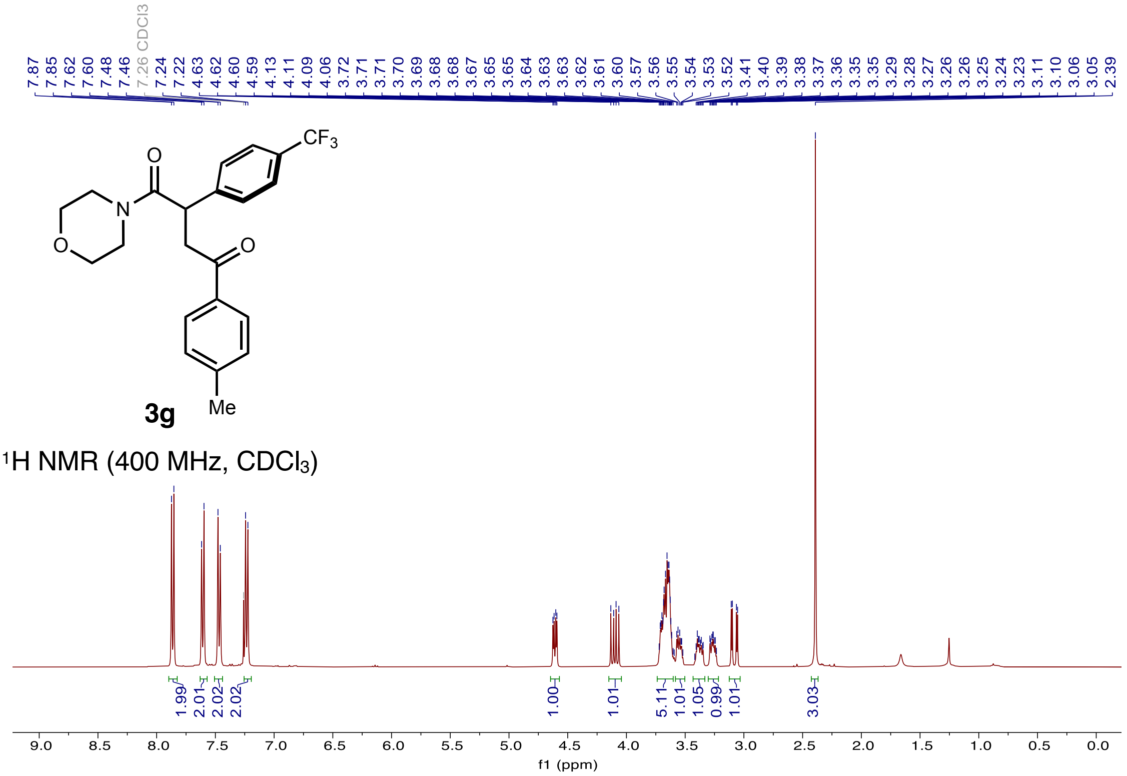
3g
¹H NMR (400 MHz, CDCl₃)
f1 (ppm)

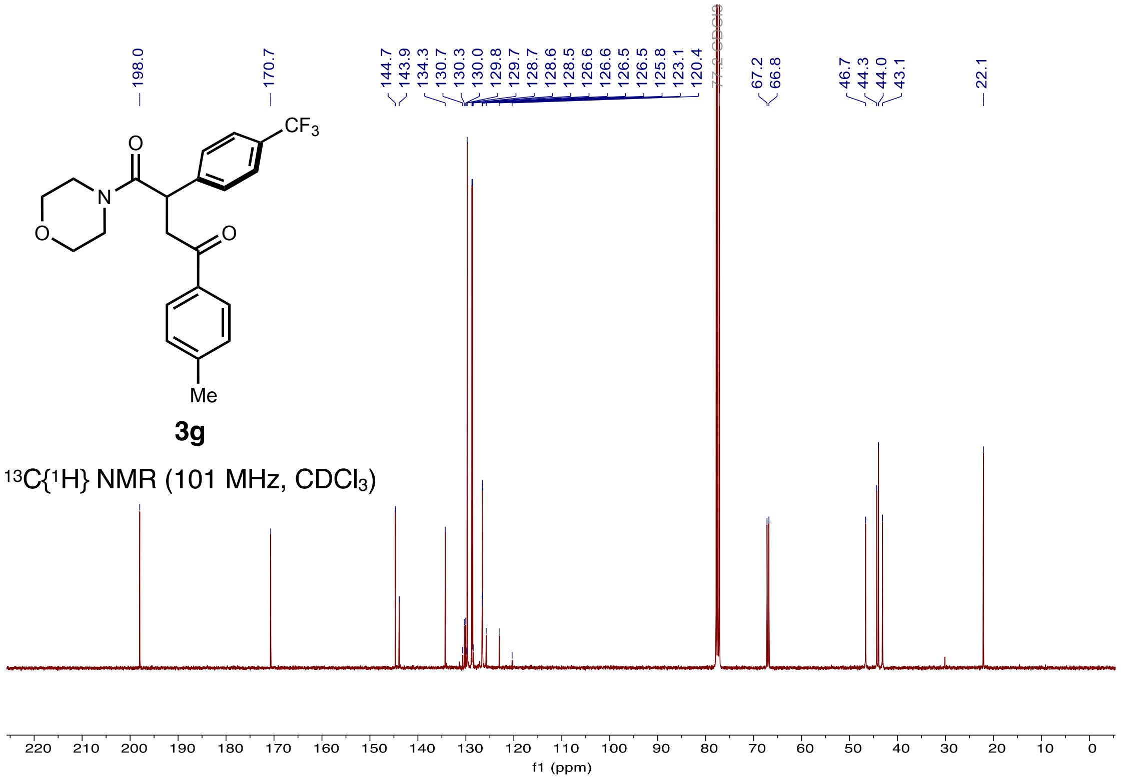
3g
¹³C{¹H} NMR (101 MHz, CDCl₃)
f1 (ppm)

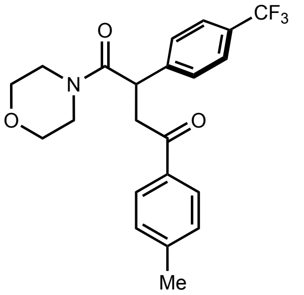

3g

^{19}F NMR (376 MHz, $CDCl_3$)

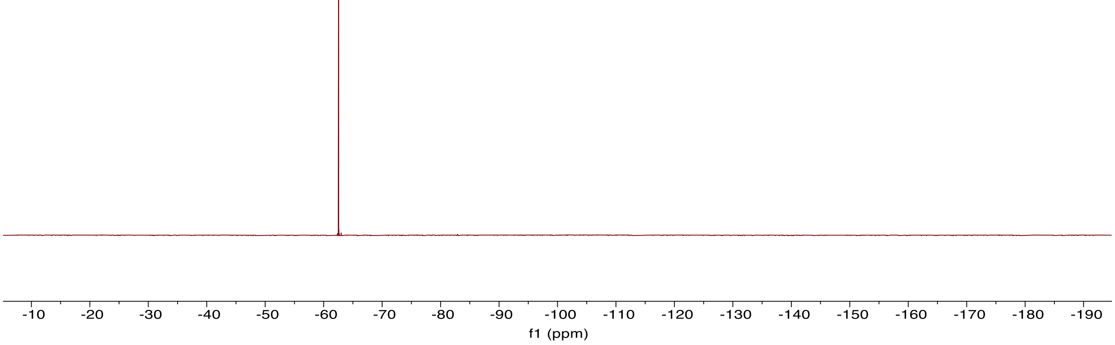

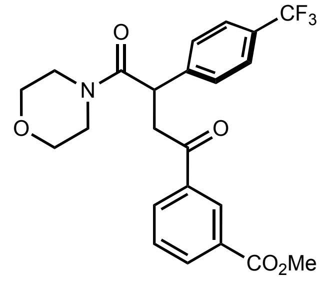

3h

^{1}H NMR (400 MHz, $CDCl_3$)

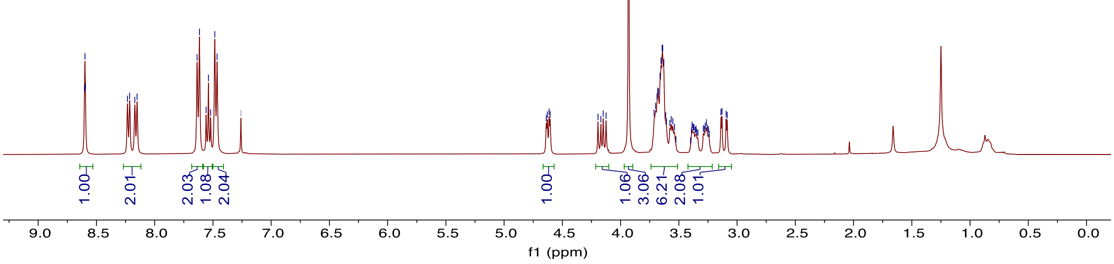

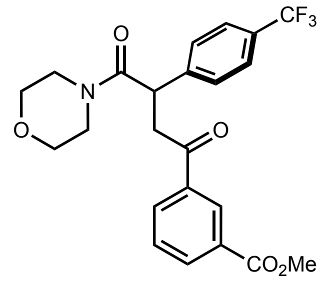

3h

$^{13}C\{^1H\}$ NMR (101 MHz, $CDCl_3$)

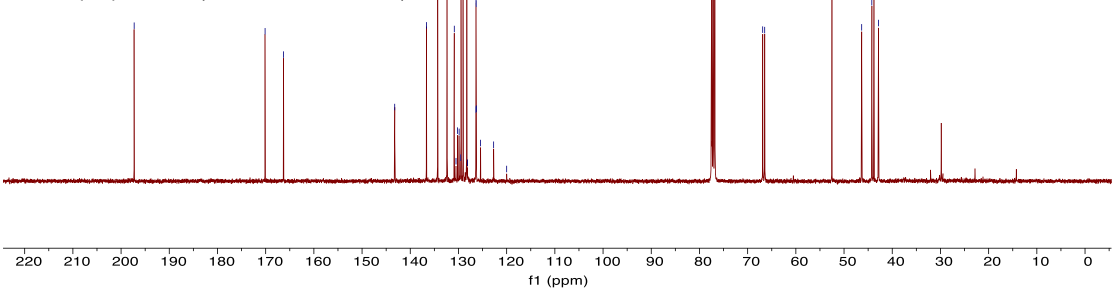

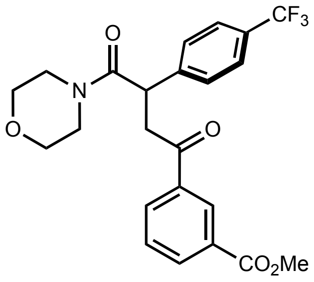

3h

^{19}F NMR (376 MHz, $CDCl_3$)

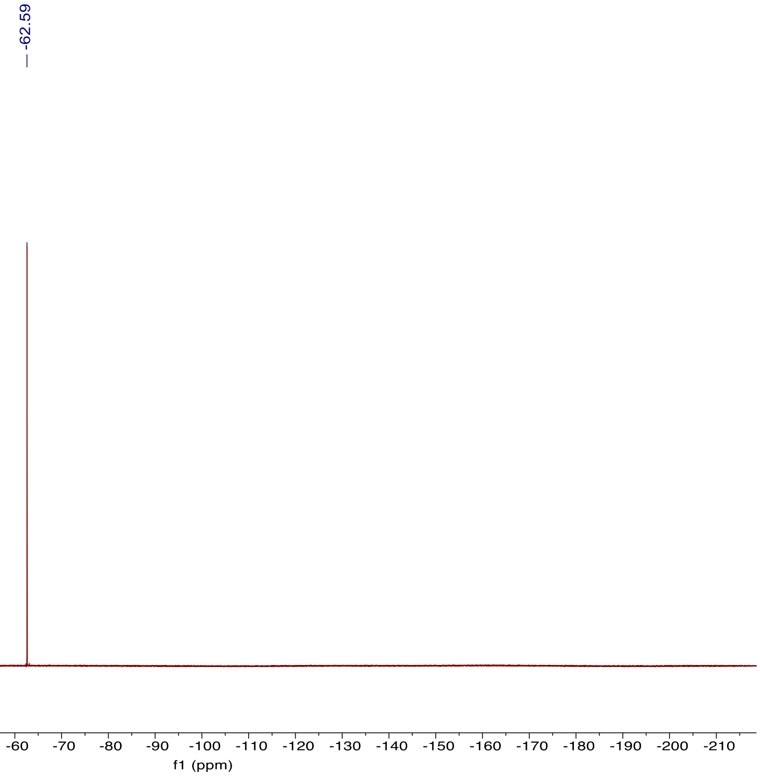

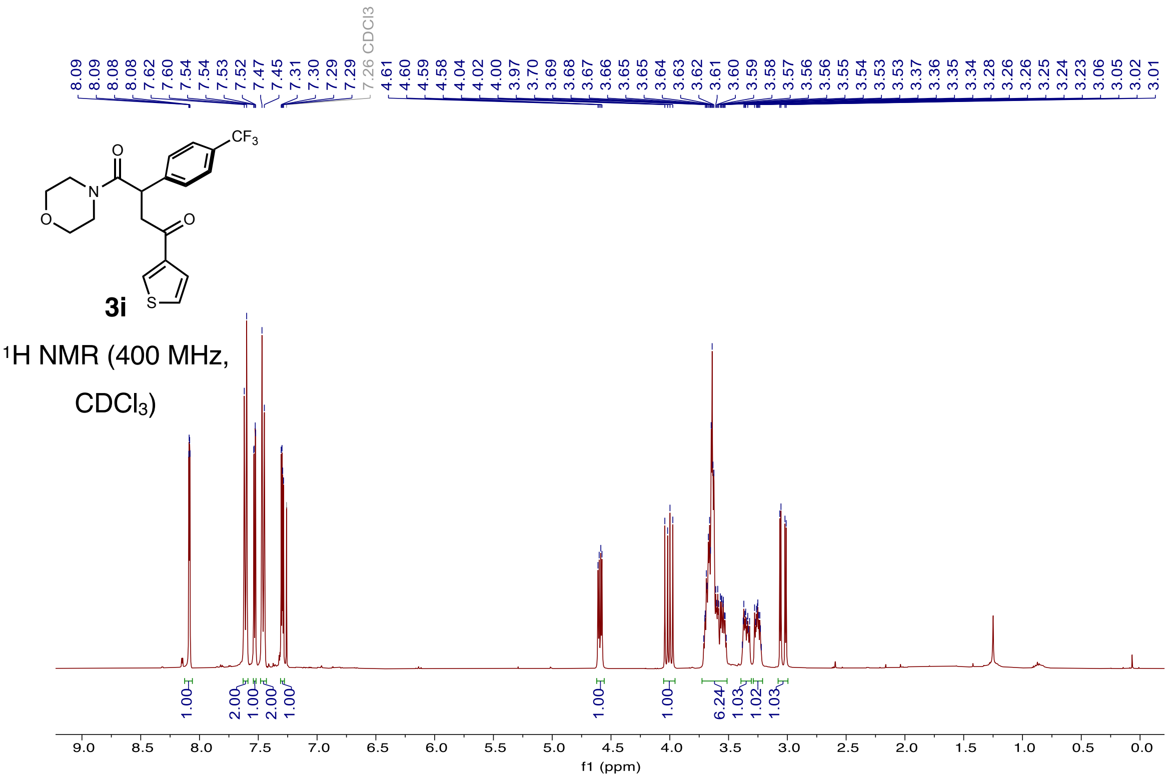
8.09
8.09
8.08
7.62
7.60
7.54
7.54
7.53
7.52
7.47
7.45
7.31
7.30
7.29
7.29
7.26 CDCl3
4.61
4.60
4.59
4.58
4.04
4.02
4.00
3.97
3.70
3.69
3.68
3.67
3.66
3.65
3.65
3.64
3.63
3.62
3.61
3.60
3.59
3.58
3.57
3.56
3.56
3.55
3.54
3.53
3.53
3.37
3.36
3.35
3.34
3.28
3.26
3.26
3.25
3.24
3.23
3.06
3.05
3.02
3.01
CF3
O
N
O
O
S
3i
1H NMR (400 MHz, CDCl3)
1.00
2.00
1.00
2.00
1.00
1.00
1.00
6.24
1.03
1.02
1.03
9.0 8.5 8.0 7.5 7.0 6.5 6.0 5.5 5.0 4.5 4.0 3.5 3.0 2.5 2.0 1.5 1.0 0.5 0.0
f1 (ppm)

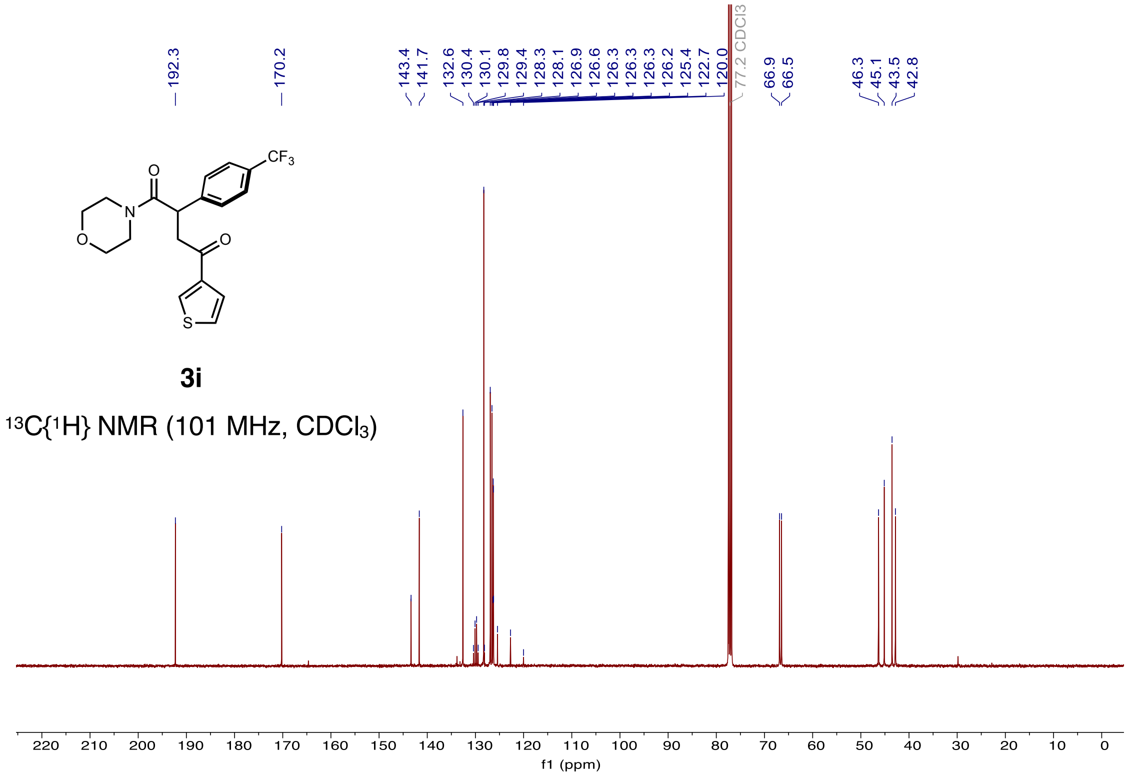
192.3
170.2
143.4
141.7
132.6
130.4
130.1
129.8
129.4
128.3
128.1
126.9
126.6
126.3
126.3
126.2
125.4
122.7
120.0
77.2 CDCl3
66.9
66.5
46.3
45.1
43.5
42.8
CF3
O
N
O
O
S
3i
13C{1H} NMR (101 MHz, CDCl3)
220 210 200 190 180 170 160 150 140 130 120 110 100 90 80 70 60 50 40 30 20 10 0
f1 (ppm)

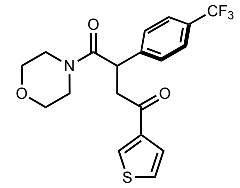

3i

^{19}F NMR (376 MHz, $CDCl_3$)

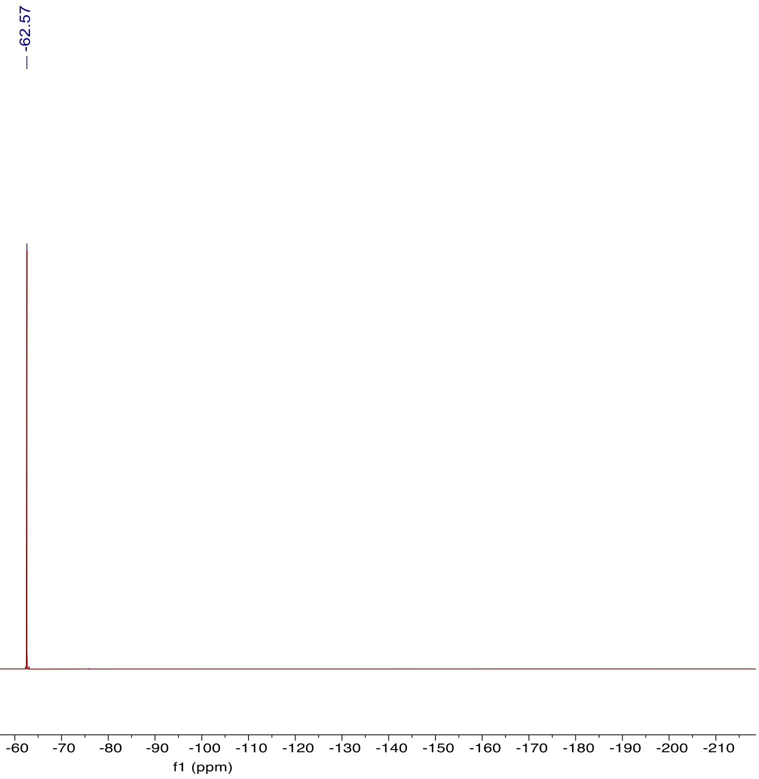

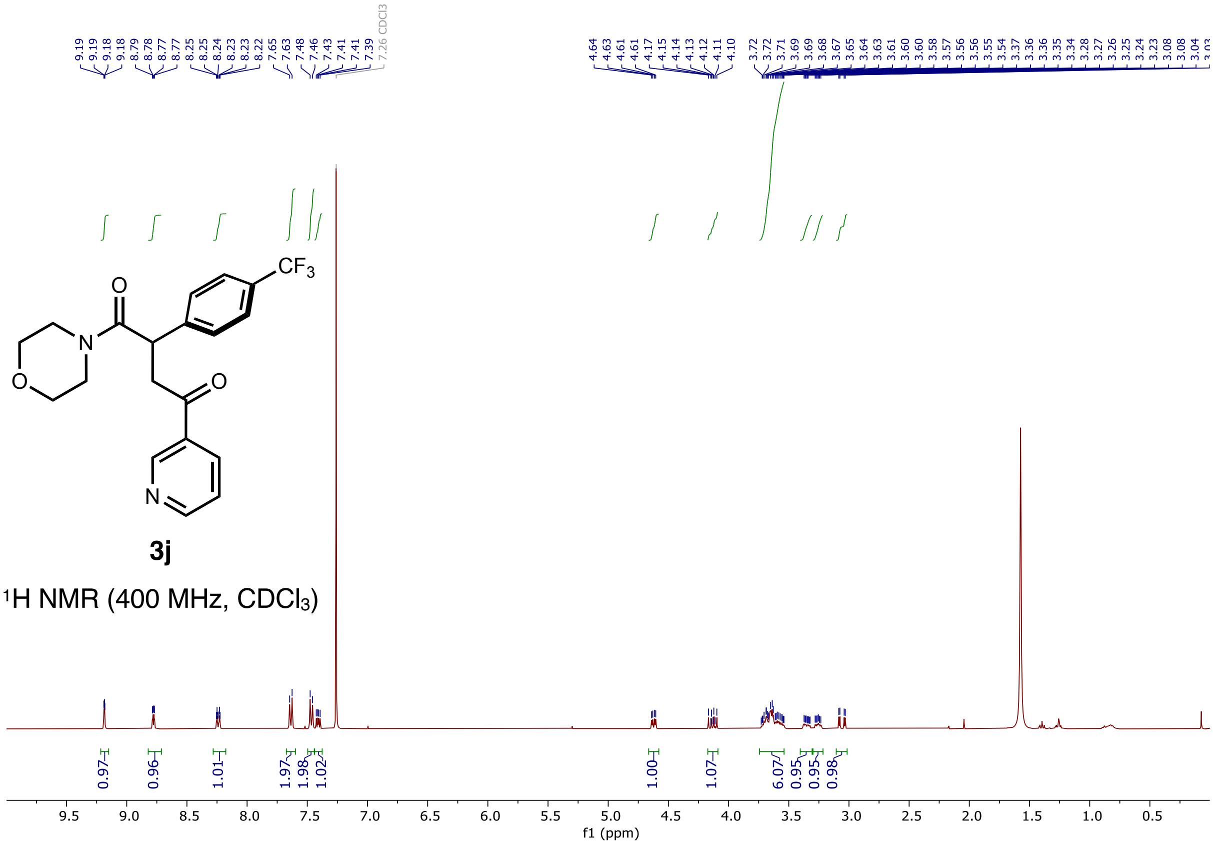
CF3
O
N
O
O
N
3j
1H NMR (400 MHz, CDCl3)
f1 (ppm)

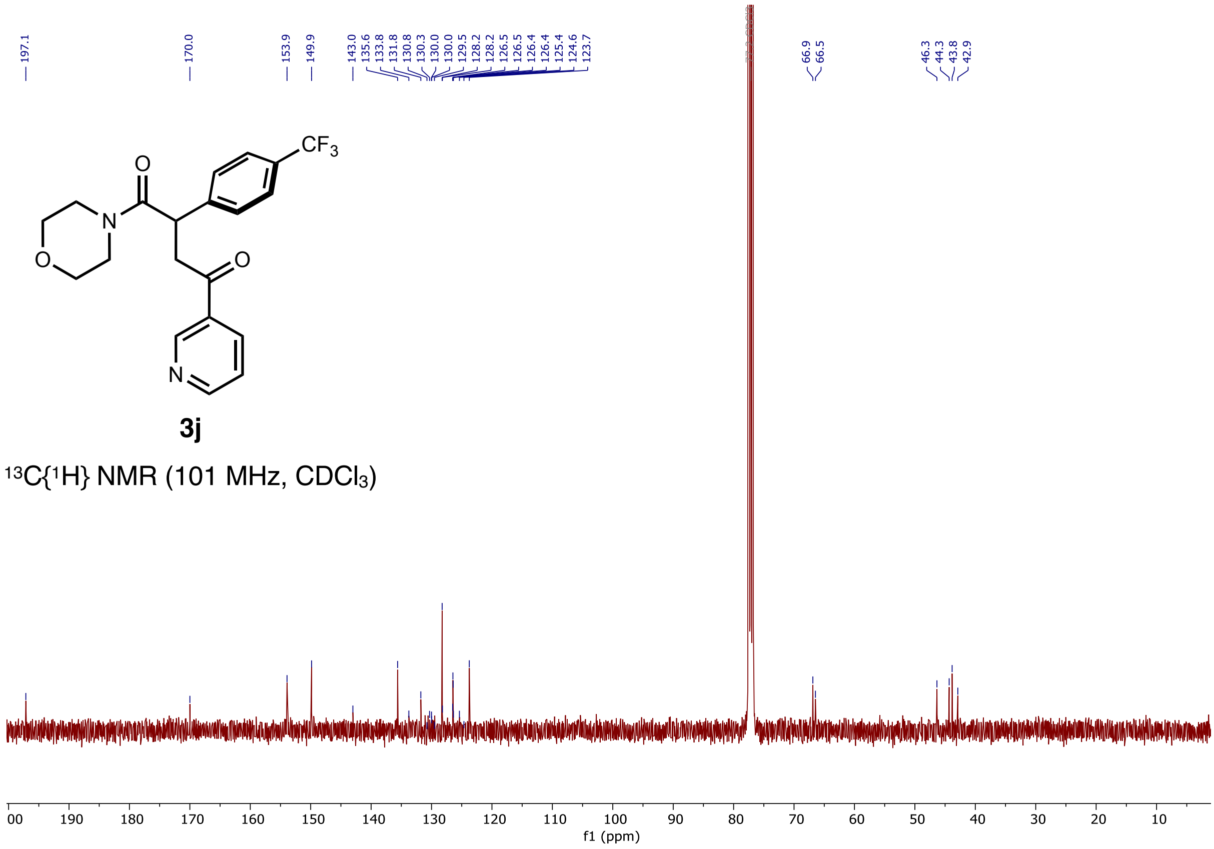
CF3
O
N
O
O
N
3j
13C{1H} NMR (101 MHz, CDCl3)
f1 (ppm)

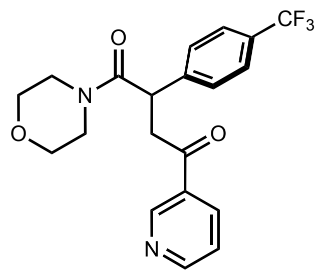

3j

^{19}F NMR (376 MHz, $CDCl_3$)

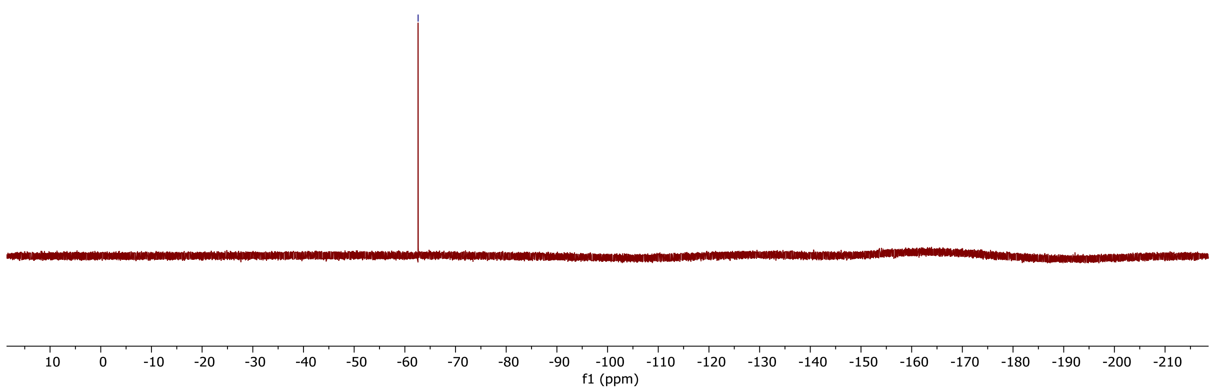

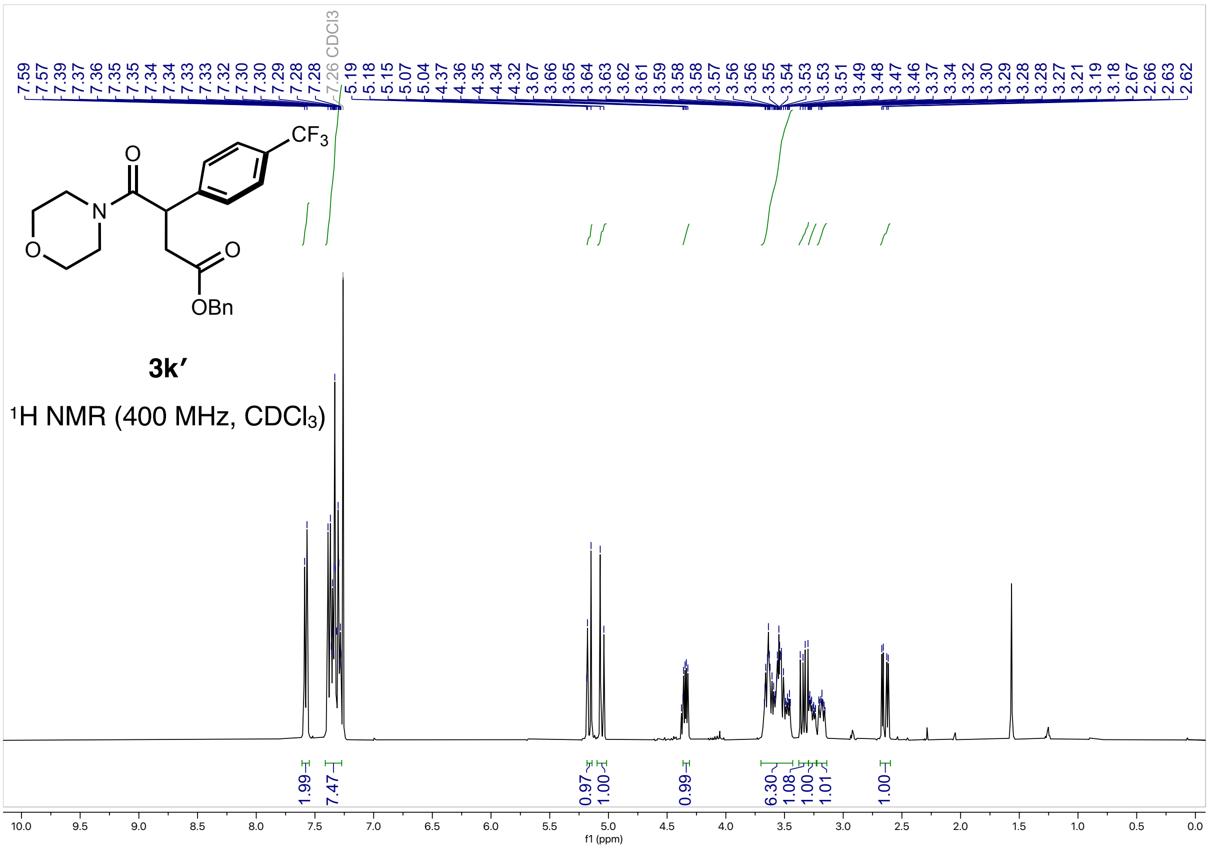

7.59
7.57
7.39
7.37
7.36
7.35
7.35
7.34
7.34
7.33
7.33
7.32
7.30
7.30
7.29
7.28
7.28
7.26 CDCl3
5.19
5.18
5.15
5.07
5.04
4.37
4.36
4.35
4.34
4.32
3.67
3.66
3.65
3.64
3.63
3.62
3.61
3.59
3.58
3.58
3.57
3.56
3.56
3.55
3.54
3.53
3.53
3.51
3.49
3.48
3.47
3.46
3.37
3.34
3.32
3.30
3.29
3.28
3.28
3.27
3.21
3.19
3.18
2.67
2.66
2.63
2.62
CF3
O
N
O
O
OBn
3k′
1H NMR (400 MHz, CDCl3)
1.99
7.47
0.97
1.00
0.99
6.30
1.08
1.00
1.01
1.00
f1 (ppm)

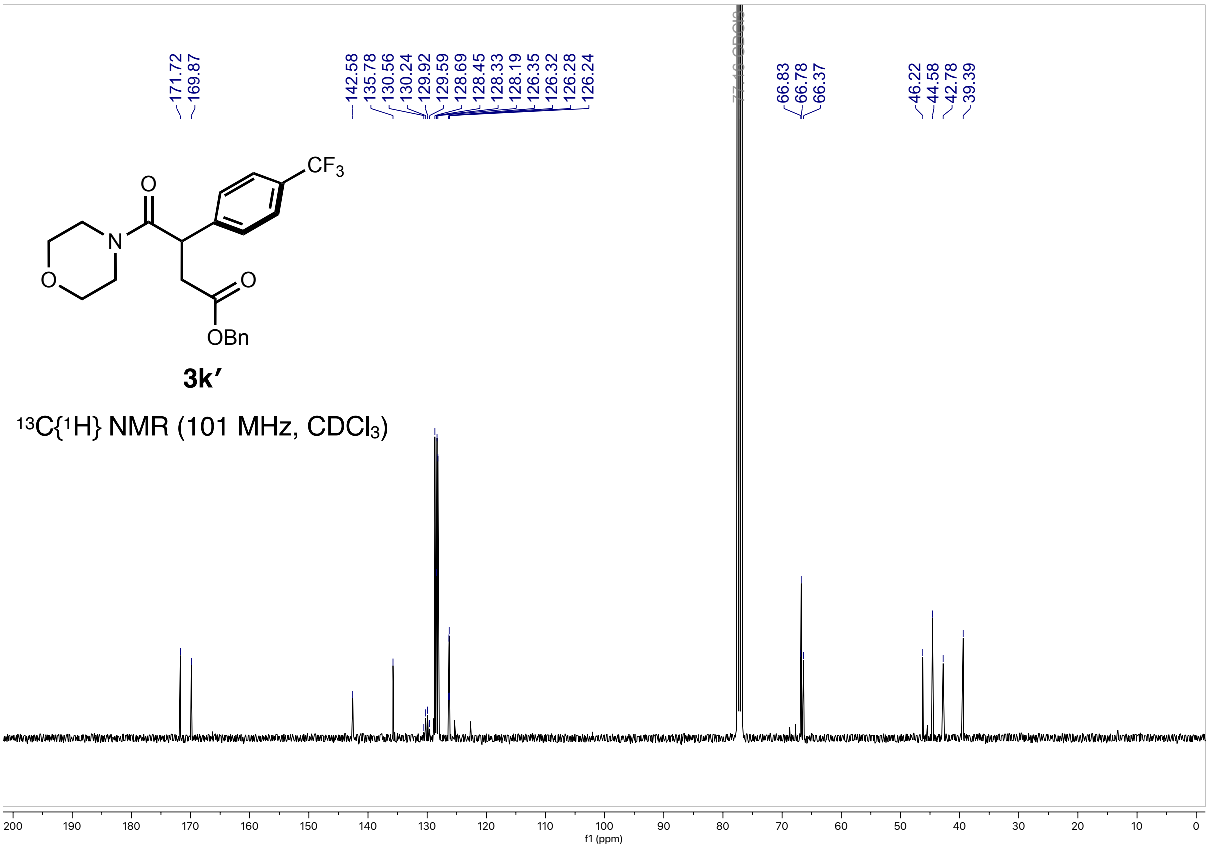

171.72
169.87
142.58
135.78
130.56
130.24
129.92
129.59
128.69
128.45
128.33
128.19
126.35
126.32
126.28
126.24
66.83
66.78
66.37
46.22
44.58
42.78
39.39
CF3
O
N
O
O
OBn
3k′
13C{1H} NMR (101 MHz, CDCl3)
f1 (ppm)

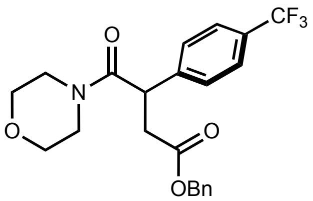

3k′

^{19}F NMR (376 MHz, $CDCl_3$)

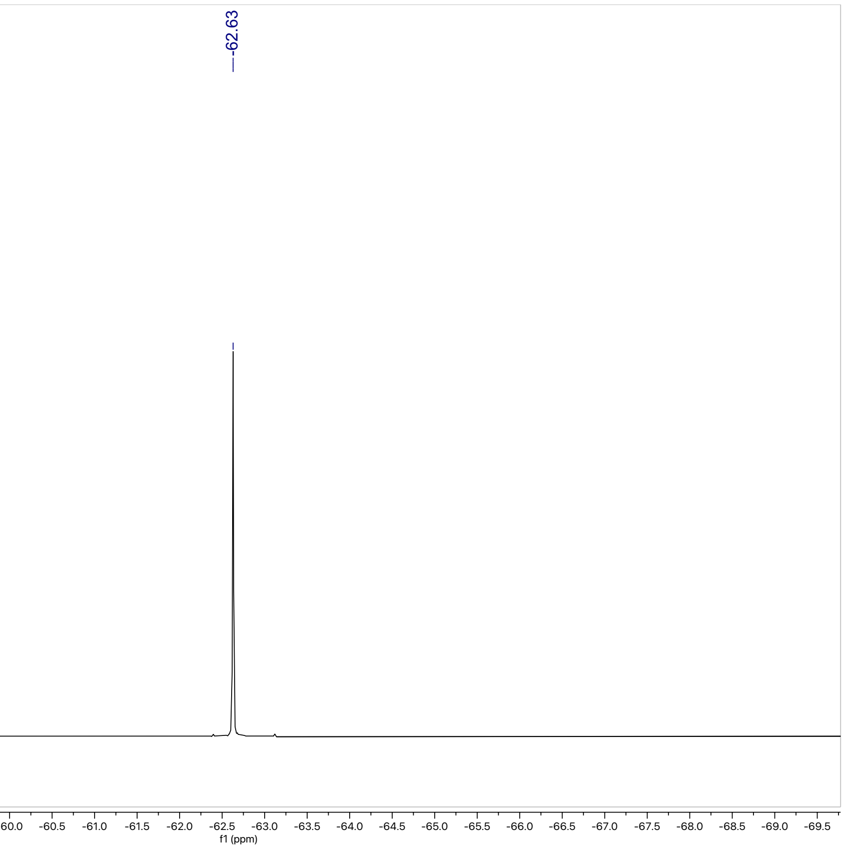

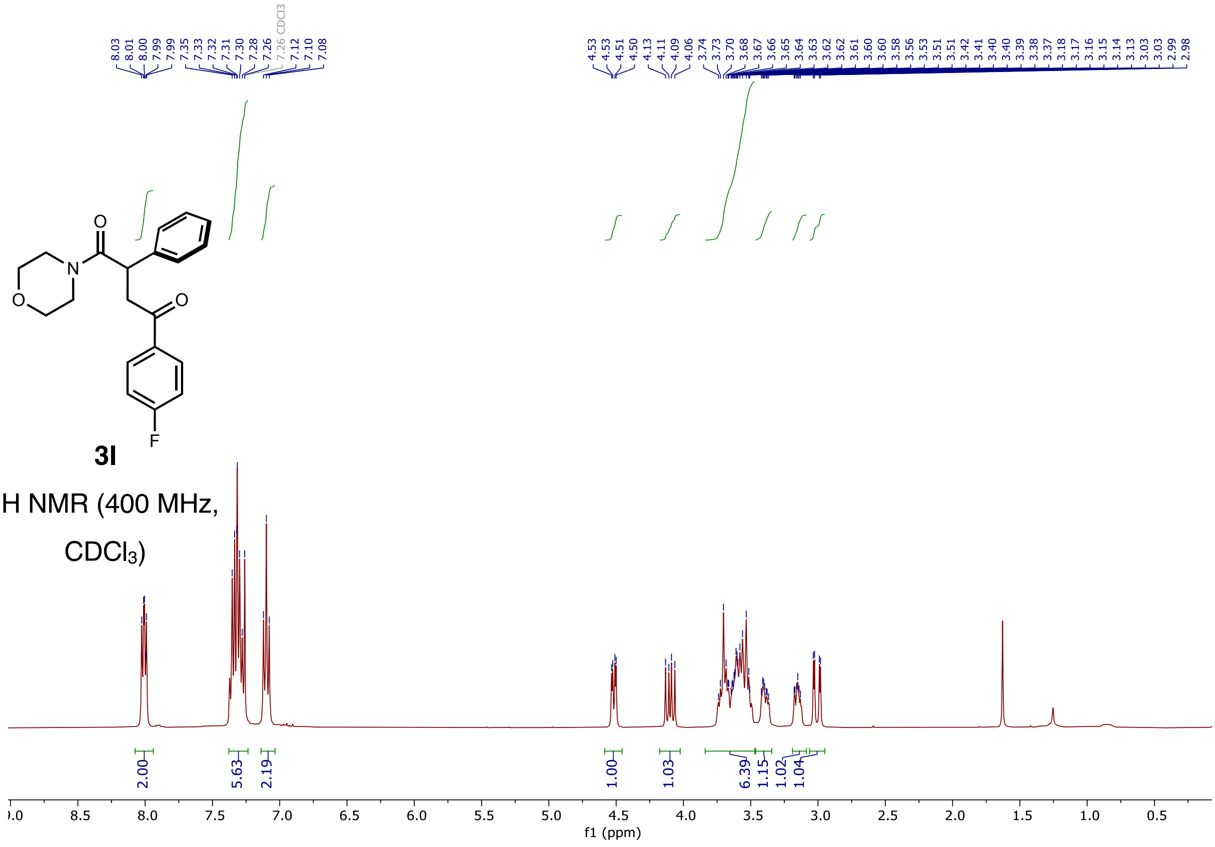

3l

^{1}H NMR (400 MHz, $CDCl_3$)

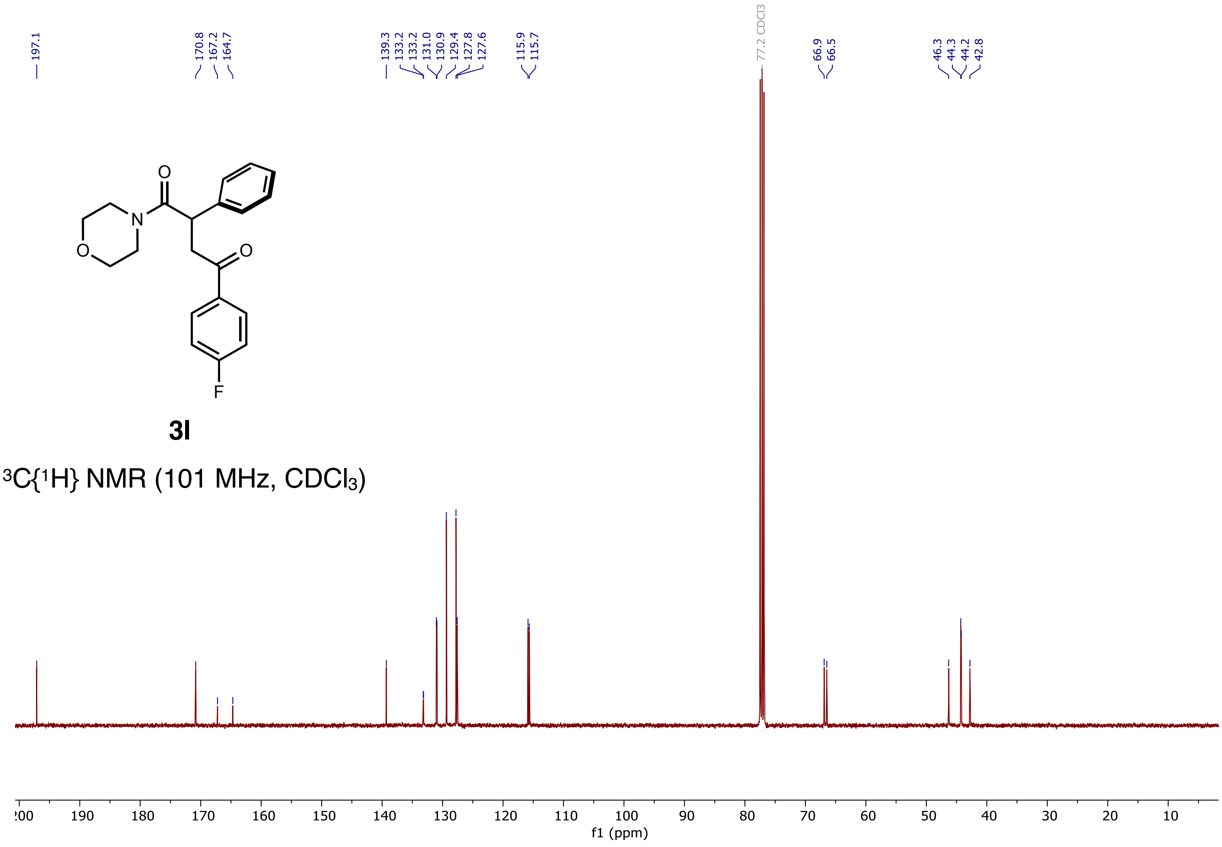

3l

^{13}C{^{1}H} NMR (101 MHz, $CDCl_3$)

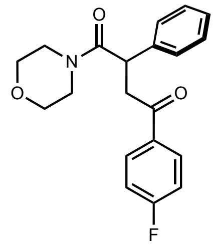

3l

^{19}F NMR (376 MHz, $CDCl_3$)

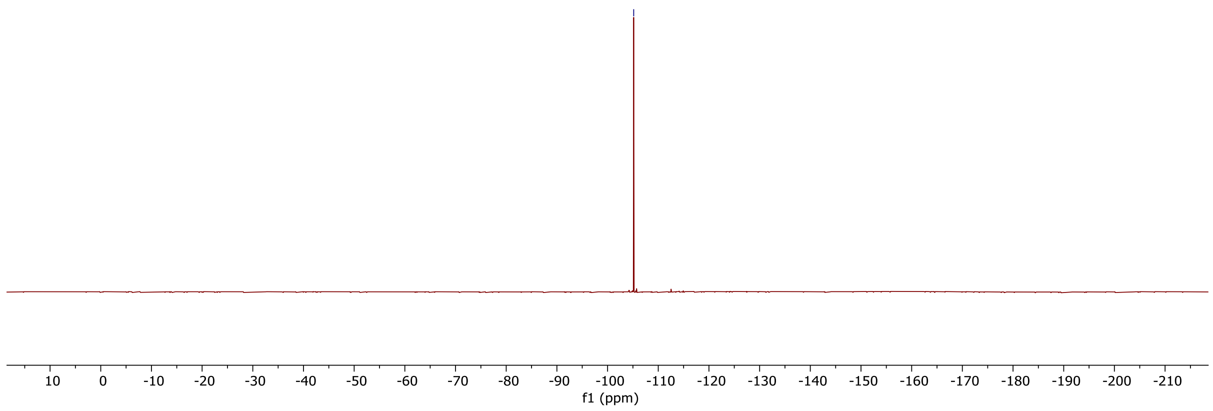

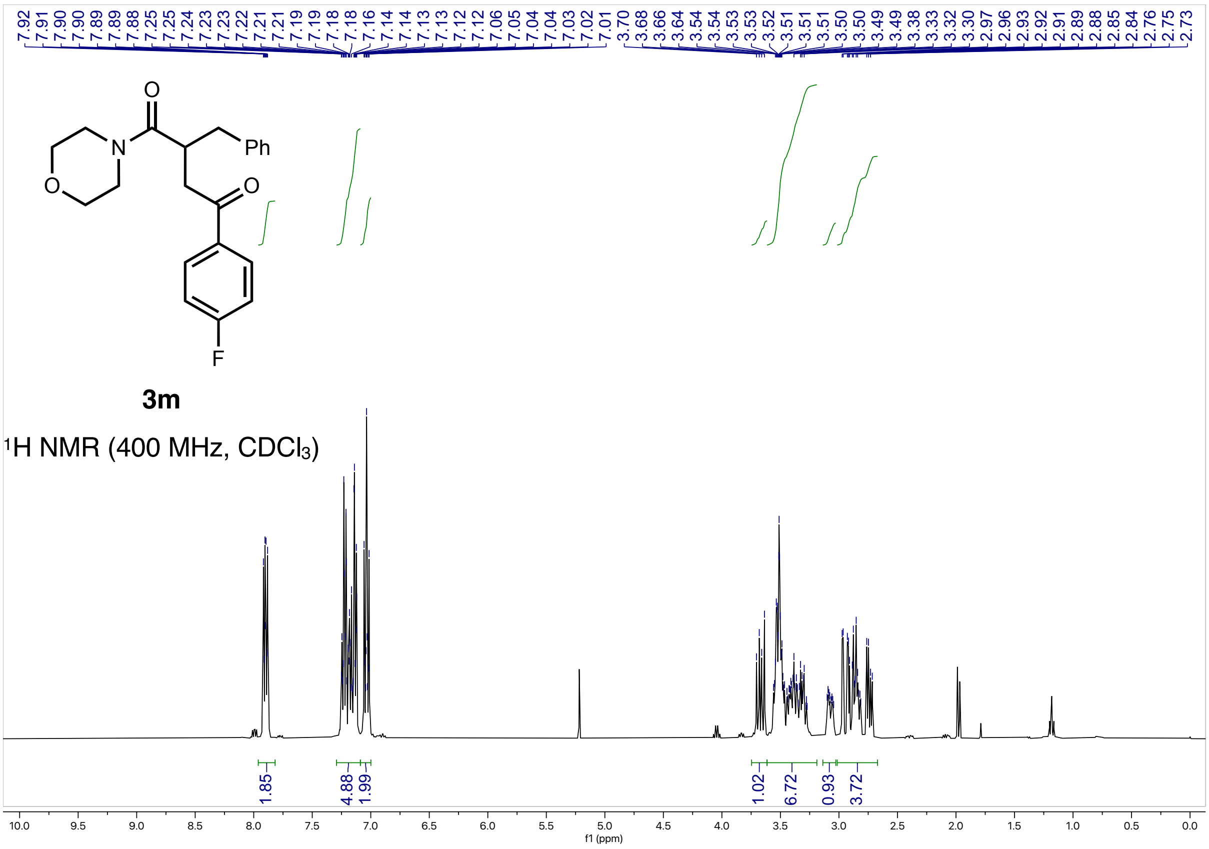

3m

^1H NMR (400 MHz, $CDCl_3$)

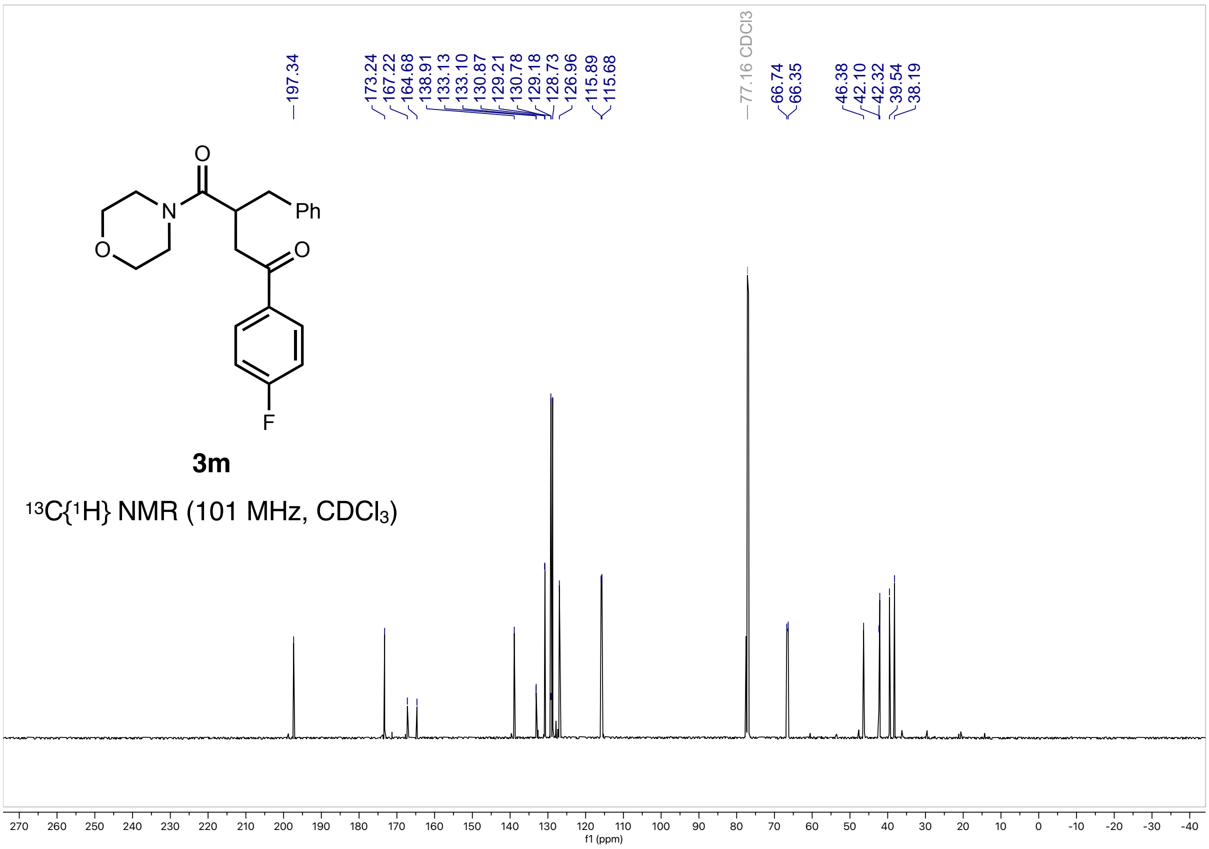

3m

$^{13}C\{^1H\}$ NMR (101 MHz, $CDCl_3$)

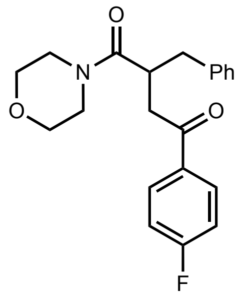

3m

^{19}F NMR (376 MHz, $CDCl_3$)

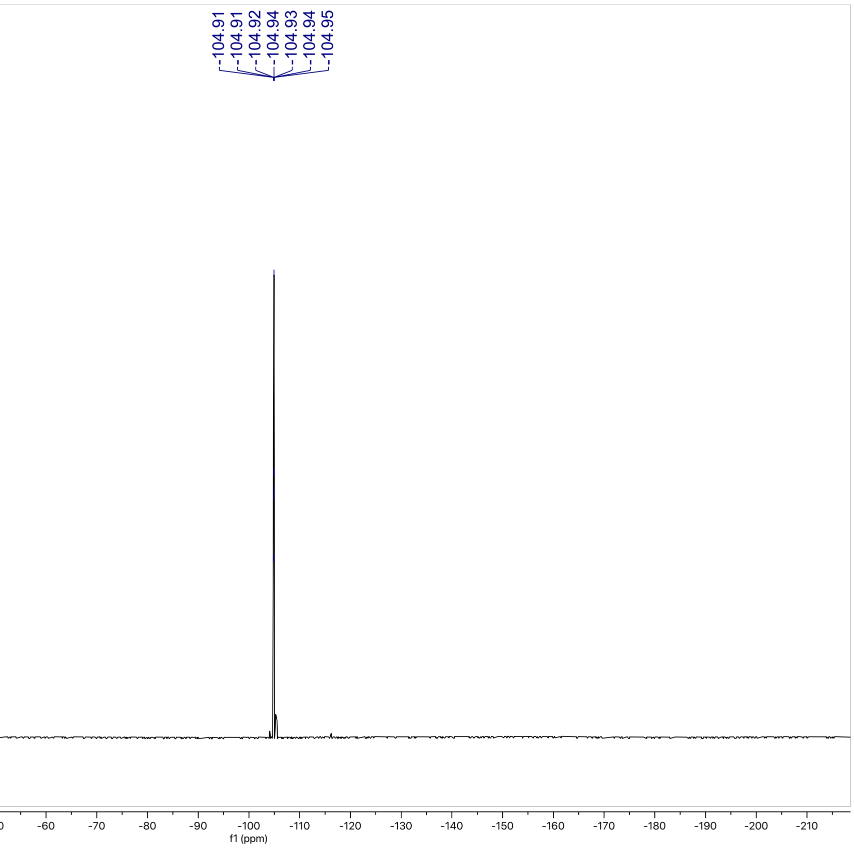

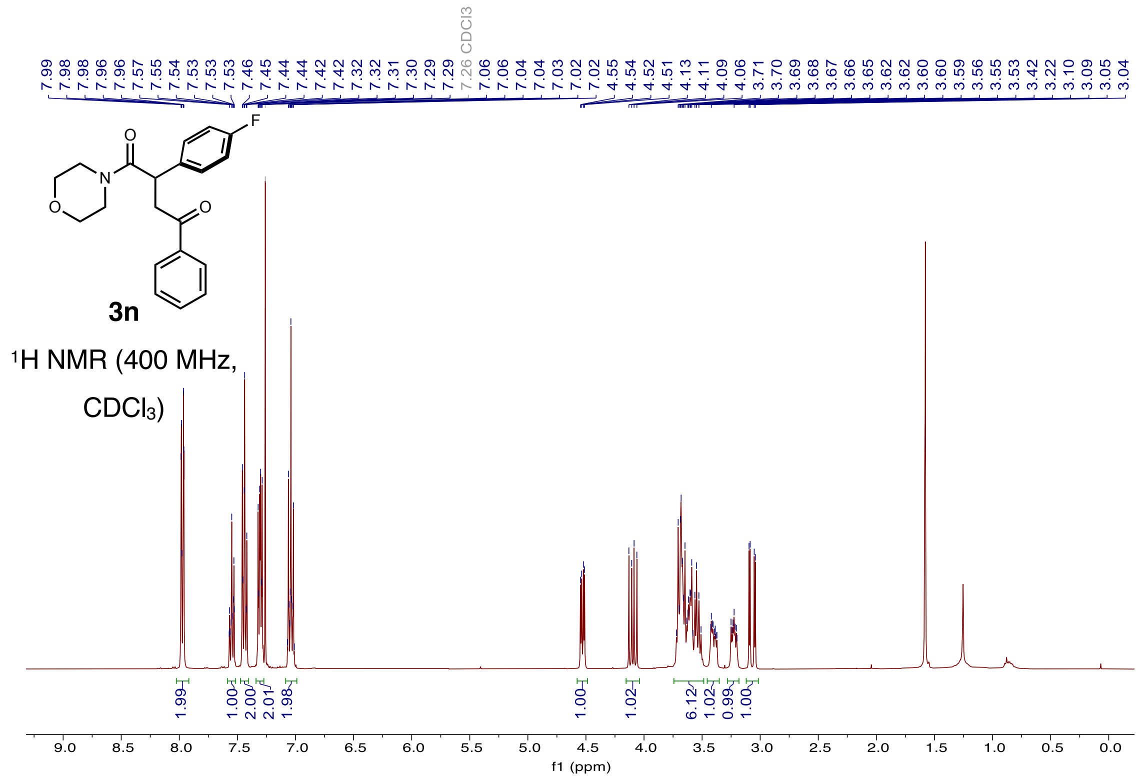
7.99 7.98 7.98 7.96 7.96 7.57 7.55 7.54 7.53 7.53 7.53 7.46 7.45 7.44 7.44 7.42 7.42 7.32 7.32 7.31 7.30 7.29 7.29 7.26 CDCl3 7.06 7.06 7.04 7.04 7.03 7.02 7.02 4.55 4.54 4.52 4.51 4.13 4.11 4.09 4.06 3.71 3.70 3.69 3.68 3.67 3.66 3.65 3.62 3.62 3.60 3.60 3.59 3.56 3.55 3.53 3.42 3.22 3.10 3.09 3.05 3.04
3n
1H NMR (400 MHz, CDCl3)
1.99 1.00 2.00 2.01 1.98 1.00 1.02 6.12 1.02 0.99 1.00
f1 (ppm)
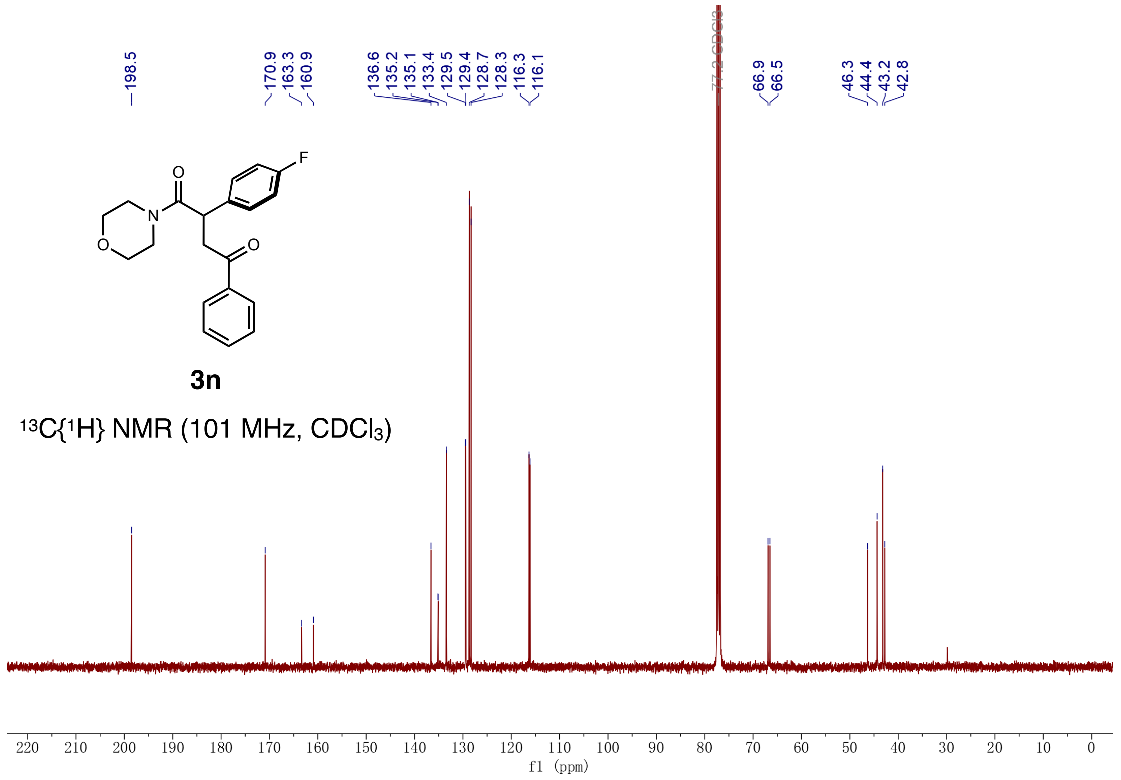
198.5 170.9 163.3 160.9 136.6 135.2 135.1 133.4 129.5 129.4 128.7 128.3 116.3 116.1 77.2 CDCl3 66.9 66.5 46.3 44.4 43.2 42.8
3n
13C{1H} NMR (101 MHz, CDCl3)
f1 (ppm)

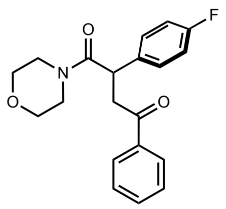

3n

^{19}F NMR (376 MHz, $CDCl_3$)

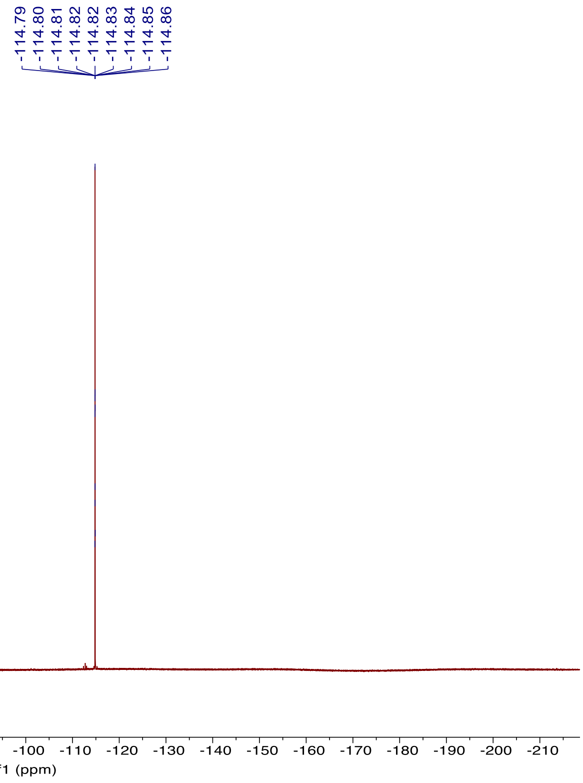

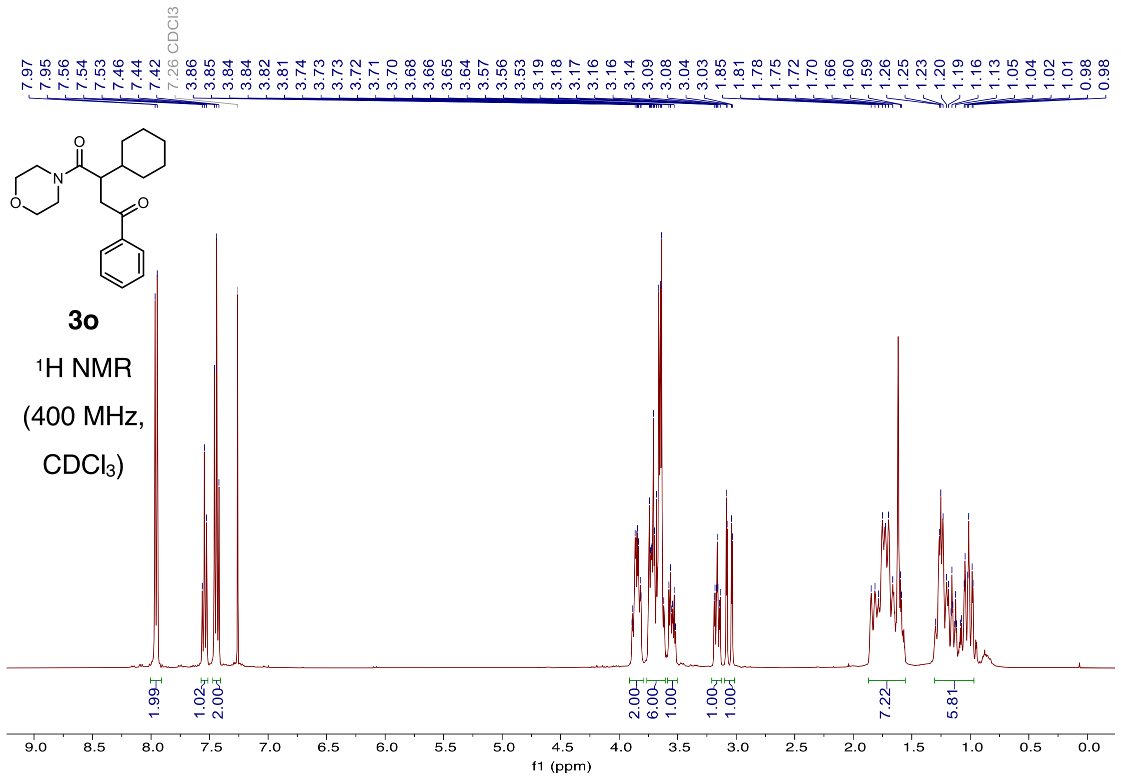
7.97 7.95 7.56 7.54 7.53 7.46 7.44 7.42 7.26 CDCl3 3.86 3.85 3.84 3.84 3.82 3.81 3.74 3.73 3.73 3.72 3.71 3.70 3.68 3.66 3.65 3.64 3.57 3.56 3.53 3.19 3.18 3.17 3.16 3.16 3.14 3.09 3.08 3.04 3.03 1.85 1.81 1.78 1.75 1.72 1.70 1.66 1.60 1.59 1.26 1.25 1.23 1.20 1.19 1.16 1.13 1.05 1.04 1.02 1.01 0.98 0.98
3o
1H NMR
(400 MHz,
CDCl3)
1.99 1.02 2.00 2.00 6.00 1.00 1.00 1.00 7.22 5.81
f1 (ppm)

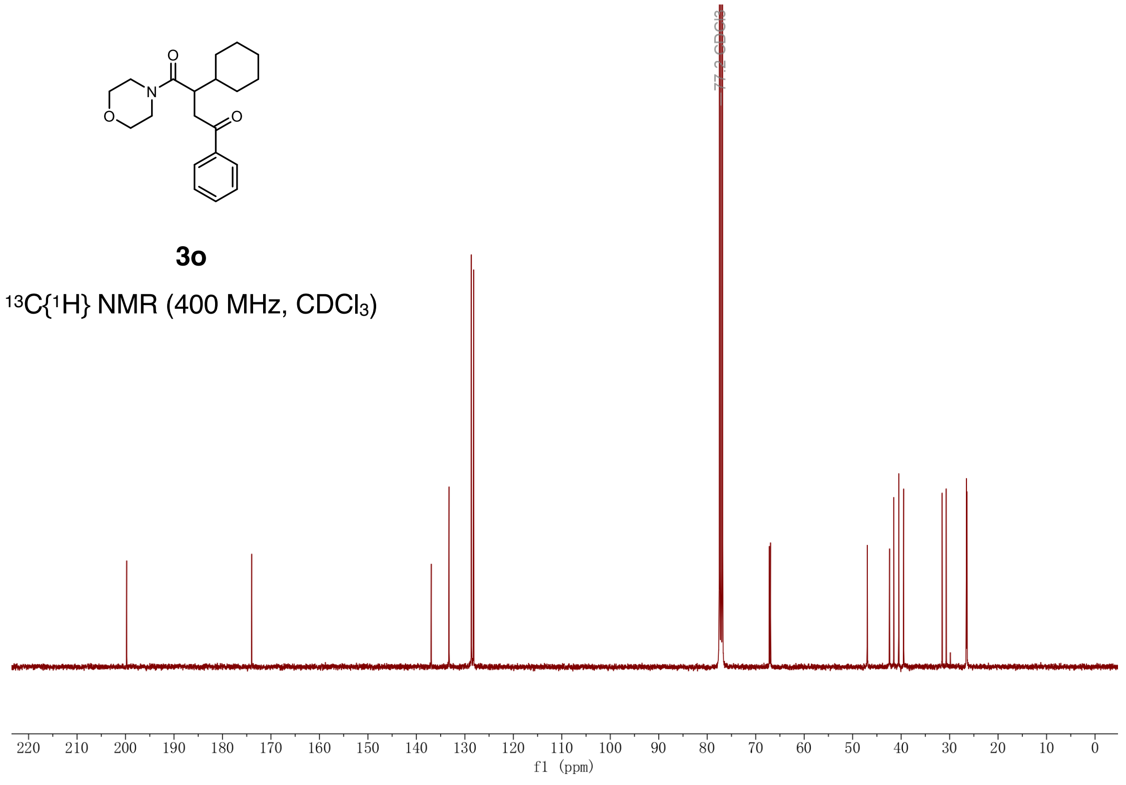
77.2 CDCl3
3o
13C{1H} NMR (400 MHz, CDCl3)
f1 (ppm)

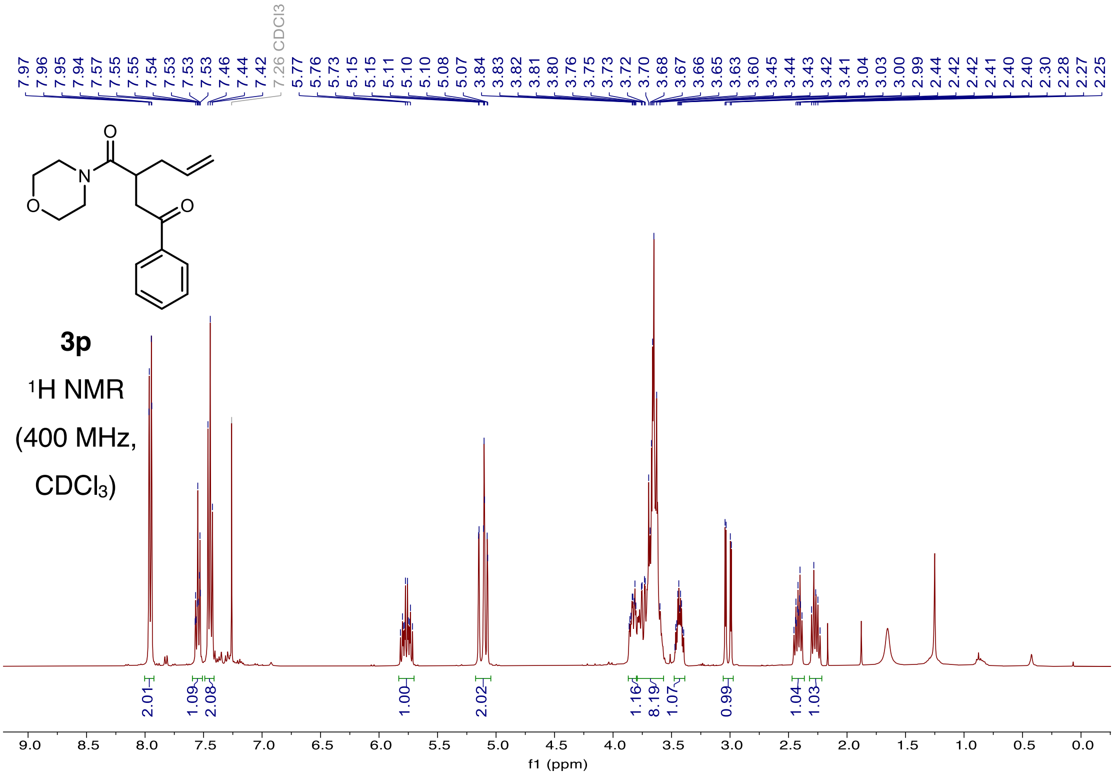
7.97
7.96
7.95
7.94
7.57
7.55
7.55
7.54
7.53
7.53
7.53
7.46
7.44
7.42
7.26 CDCl3
5.77
5.76
5.73
5.15
5.15
5.11
5.10
5.10
5.08
5.07
3.84
3.83
3.82
3.81
3.80
3.76
3.75
3.73
3.72
3.70
3.68
3.67
3.66
3.65
3.63
3.60
3.45
3.44
3.44
3.43
3.42
3.41
3.04
3.03
3.00
2.99
2.44
2.42
2.42
2.41
2.40
2.40
2.30
2.28
2.27
2.25
O
N
O
O
3p
1H NMR
(400 MHz,
CDCl3)
2.01
1.09
2.08
1.00
2.02
1.16
8.19
1.07
0.99
1.04
1.03
f1 (ppm)

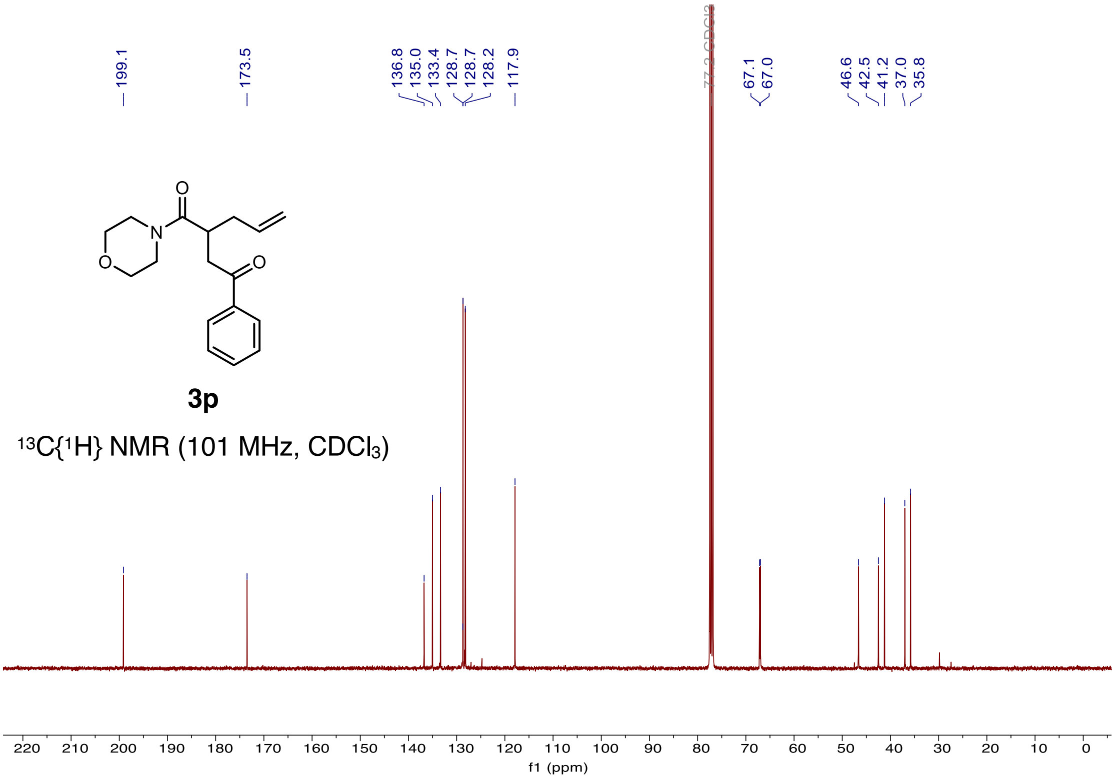
199.1
173.5
136.8
135.0
133.4
128.7
128.7
128.2
117.9
67.1
67.0
46.6
42.5
41.2
37.0
35.8
O
N
O
O
3p
13C{1H} NMR (101 MHz, CDCl3)
f1 (ppm)

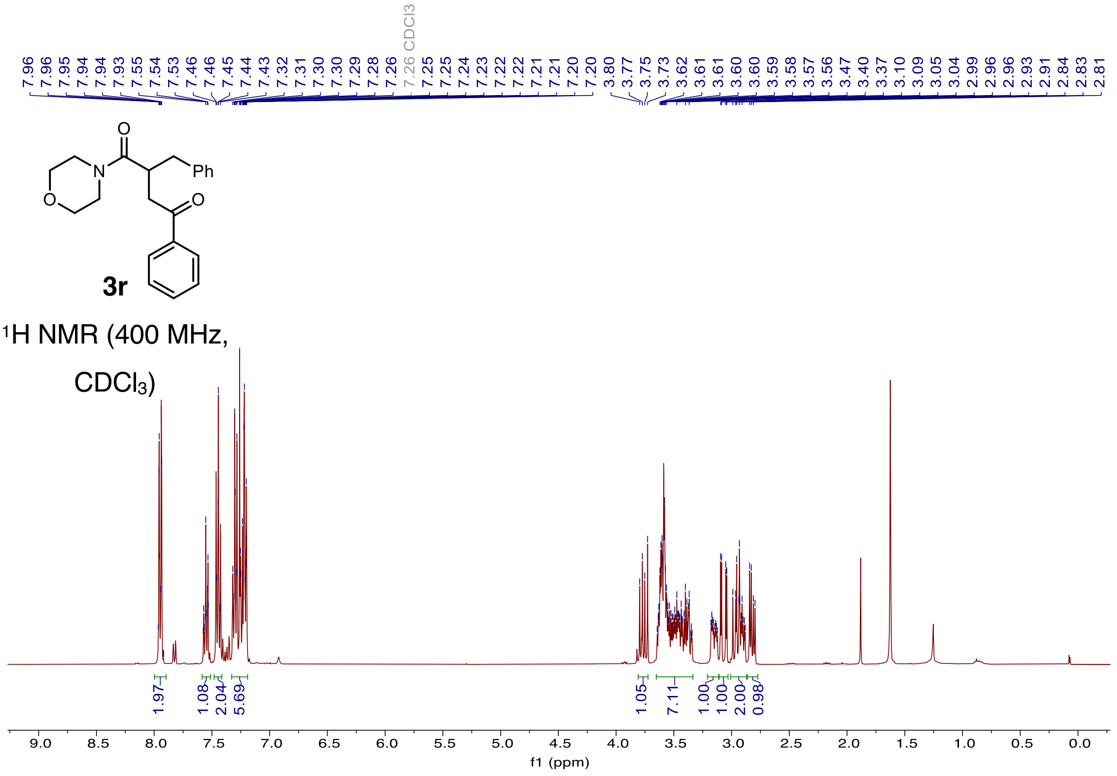
7.96 7.96 7.95 7.94 7.94 7.93 7.55 7.54 7.53 7.46 7.45 7.44 7.43 7.32 7.31 7.30 7.30 7.29 7.28 7.26 7.26 CDCl3 7.25 7.25 7.24 7.23 7.22 7.22 7.21 7.21 7.20 7.20
3.80 3.77 3.75 3.73 3.62 3.61 3.61 3.60 3.60 3.59 3.58 3.57 3.56 3.47 3.40 3.37 3.10 3.09 3.05 3.04 2.99 2.96 2.96 2.93 2.91 2.84 2.83 2.81
Ph
O
N
3r
^{1}H NMR (400 MHz, CDCl$_3$)
1.97 1.08 2.04 5.69 1.05 7.11 1.00 1.00 2.00 0.98
f1 (ppm)

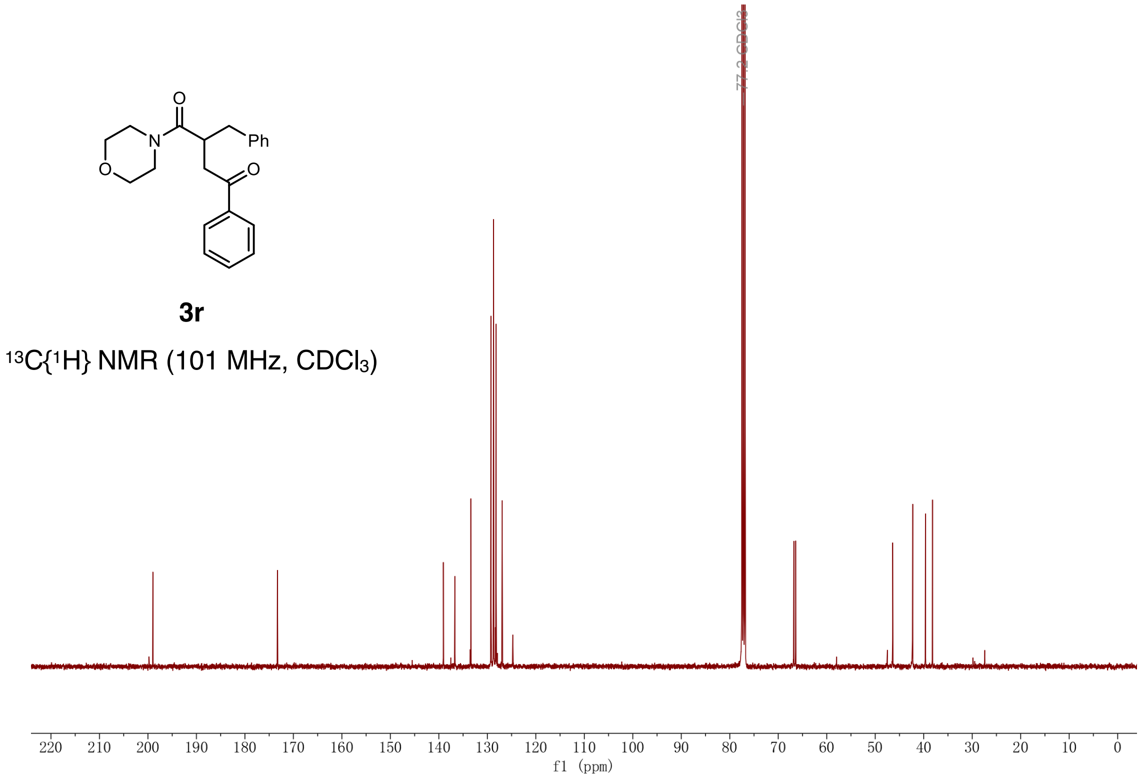
77.2 CDCl3
Ph
O
N
3r
^{13}C{^{1}H} NMR (101 MHz, CDCl$_3$)
f1 (ppm)

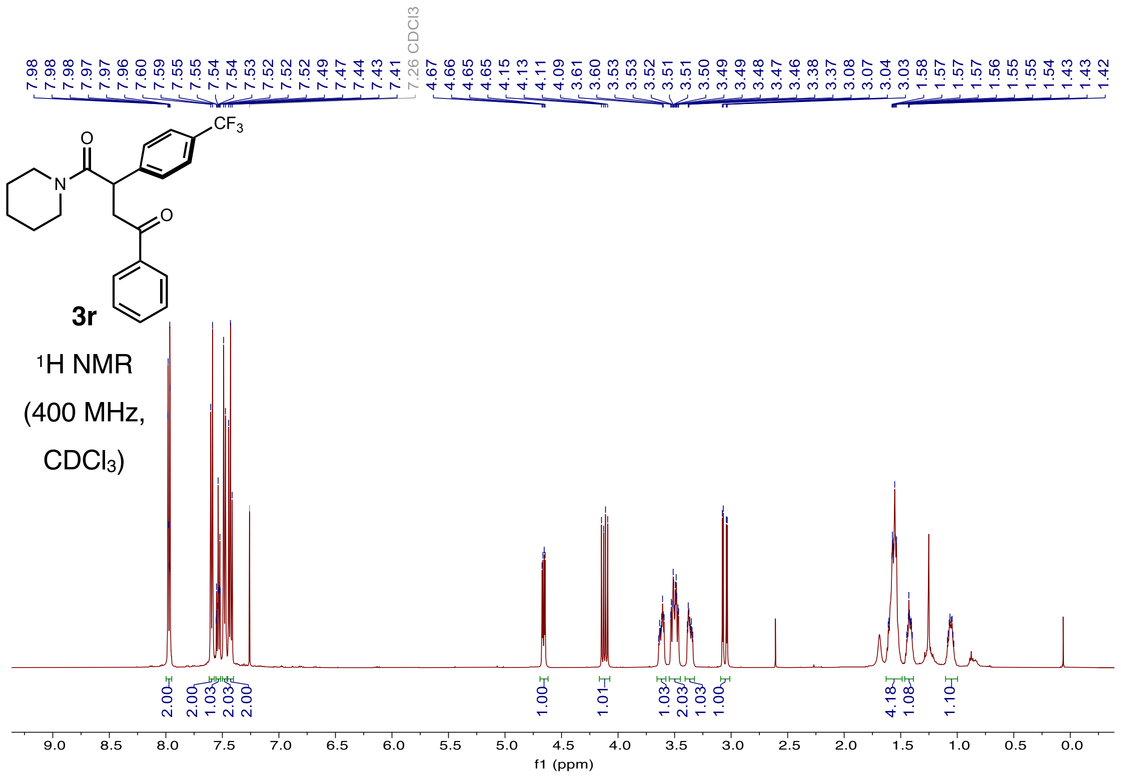
7.98
7.98
7.98
7.97
7.97
7.96
7.60
7.59
7.55
7.55
7.54
7.54
7.53
7.52
7.52
7.52
7.49
7.47
7.44
7.43
7.41
7.26 CDCl3
4.67
4.66
4.65
4.65
4.15
4.13
4.11
4.09
3.61
3.60
3.53
3.53
3.52
3.51
3.51
3.50
3.49
3.49
3.48
3.47
3.46
3.38
3.37
3.08
3.07
3.04
3.03
1.58
1.57
1.57
1.57
1.56
1.55
1.55
1.54
1.43
1.43
1.42
O
N
CF3
O
3r
¹H NMR
(400 MHz,
CDCl3)
2.00
2.00
1.03
2.03
2.00
1.00
1.01
1.03
2.03
1.03
1.00
4.18
1.08
1.10
9.0
8.5
8.0
7.5
7.0
6.5
6.0
5.5
5.0
4.5
4.0
3.5
3.0
2.5
2.0
1.5
1.0
0.5
0.0
f1 (ppm)

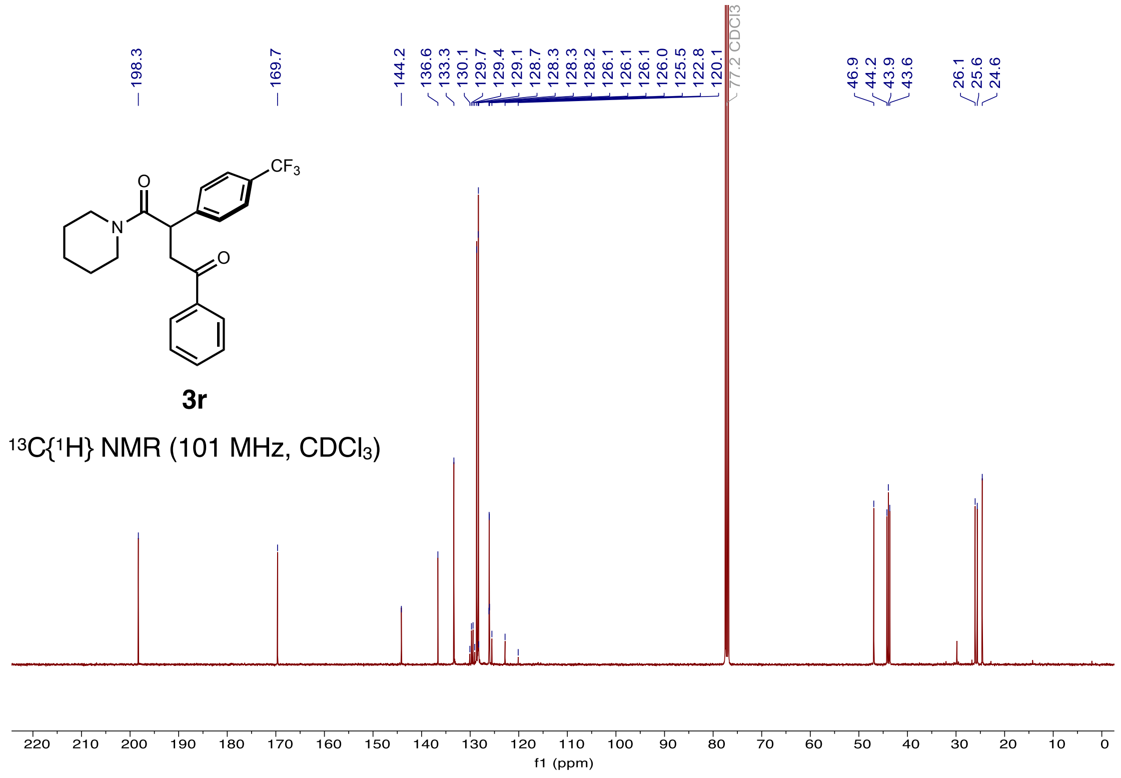
198.3
169.7
144.2
136.6
133.3
130.1
129.7
129.4
129.1
128.7
128.3
128.2
126.1
126.1
126.0
125.5
122.8
120.1
77.2 CDCl3
46.9
44.2
43.9
43.6
26.1
25.6
24.6
O
N
CF3
O
3r
¹³C{¹H} NMR (101 MHz, CDCl3)
220
210
200
190
180
170
160
150
140
130
120
110
100
90
80
70
60
50
40
30
20
10
0
f1 (ppm)

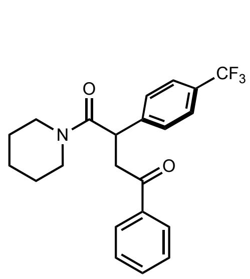

3r

^{19}F NMR (376 MHz, $CDCl_3$)

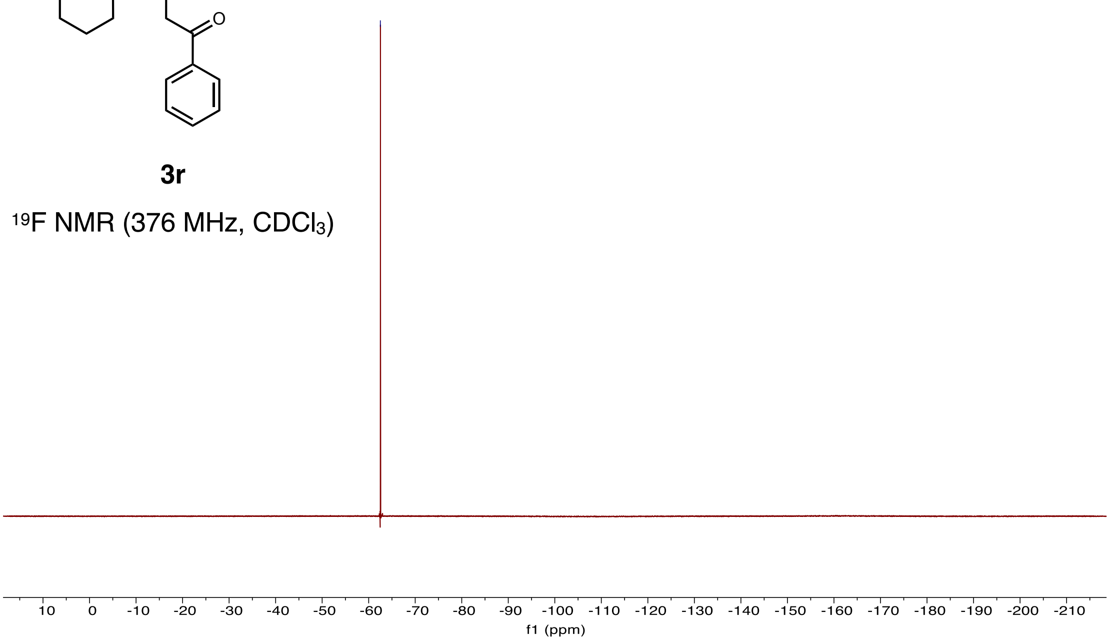

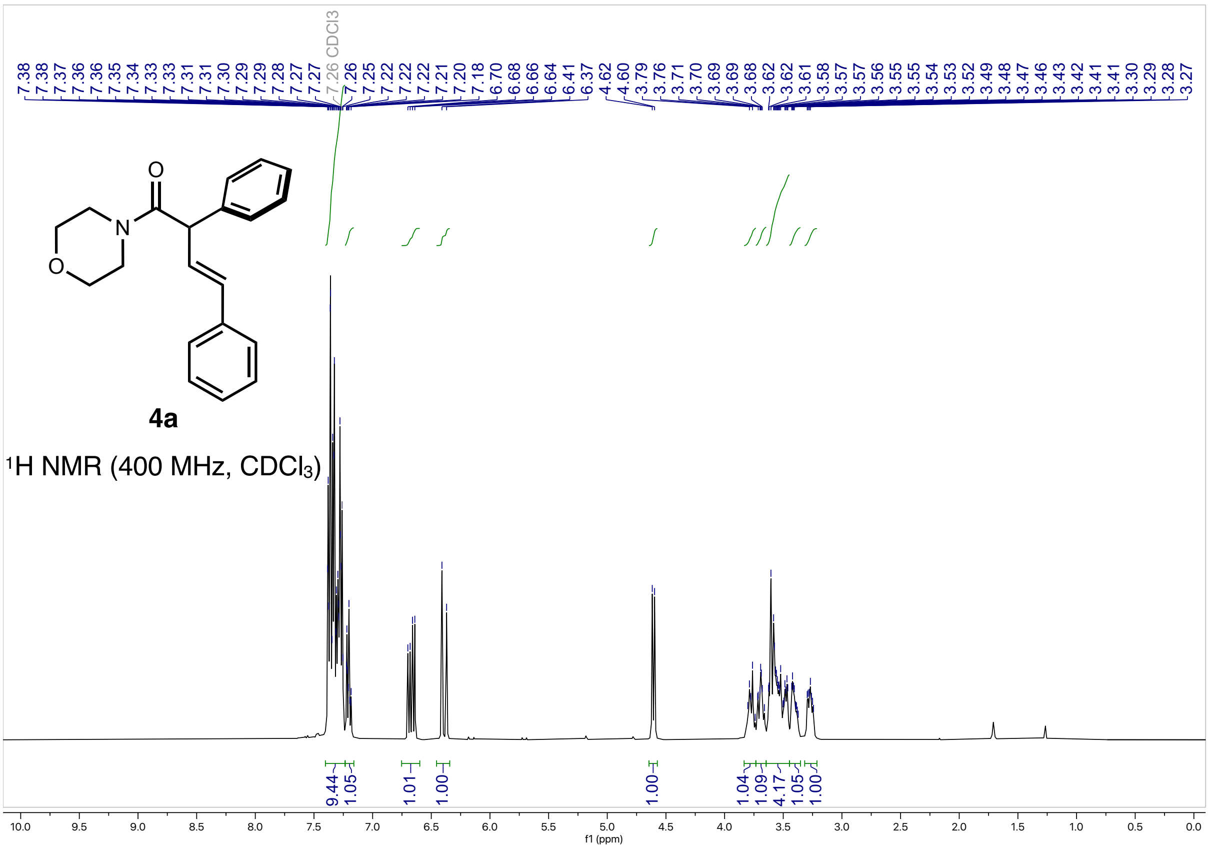

4a
¹H NMR (400 MHz, CDCl₃)
7.26 CDCl3
9.44
1.05
1.01
1.00
1.00
1.04
1.09
4.17
1.05
1.00
f1 (ppm)

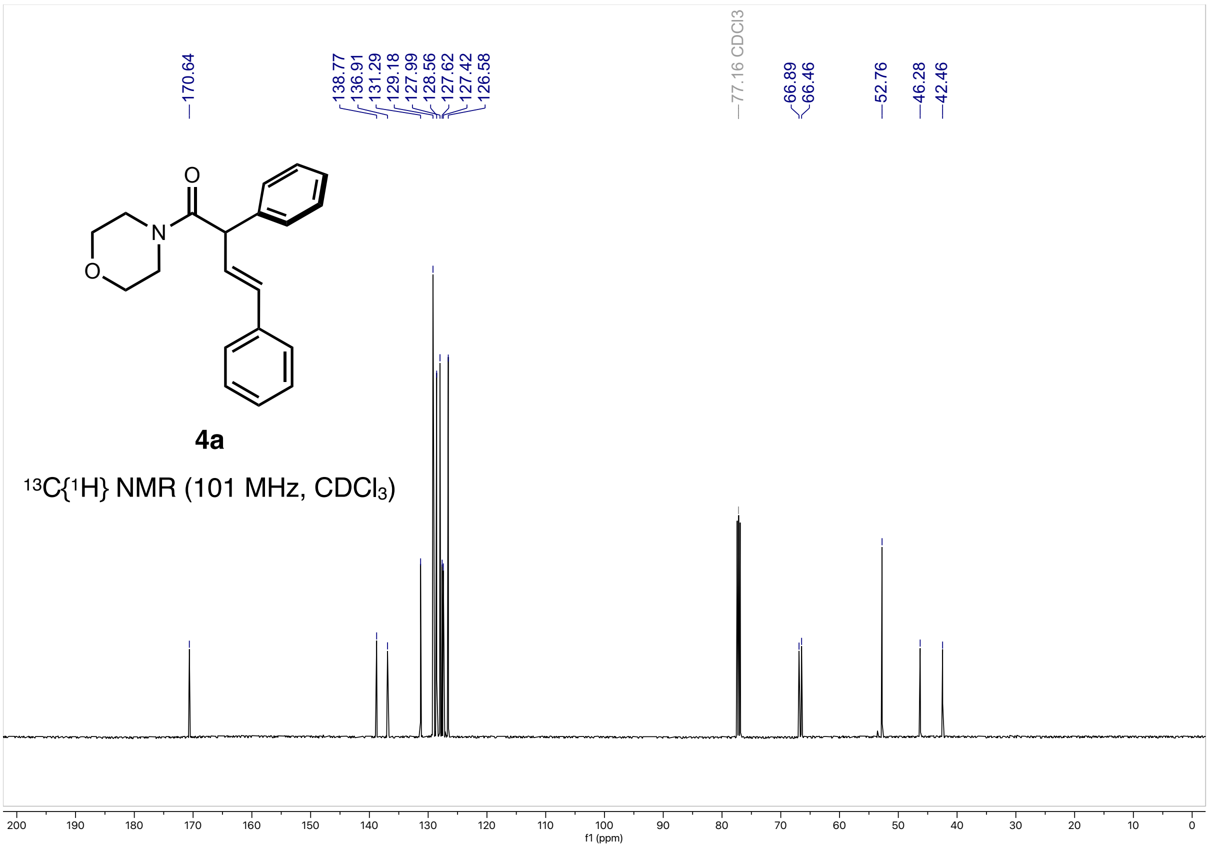

170.64
138.77
136.91
131.29
129.18
127.99
128.56
127.62
127.42
126.58
77.16 CDCl3
66.89
66.46
52.76
46.28
42.46
4a
¹³C{¹H} NMR (101 MHz, CDCl₃)
f1 (ppm)

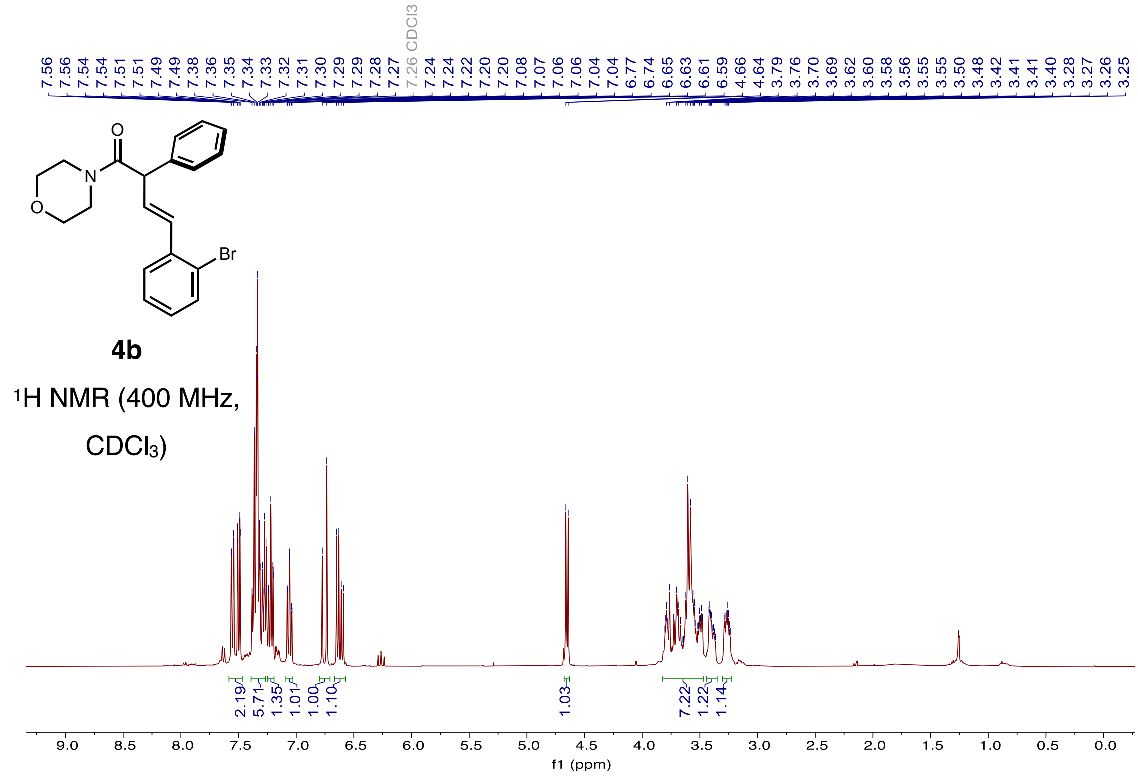
7.56 7.56 7.54 7.54 7.51 7.51 7.49 7.49 7.38 7.36 7.35 7.34 7.33 7.32 7.31 7.30 7.29 7.29 7.28 7.27 7.26 CDCl3 7.24 7.24 7.22 7.20 7.20 7.08 7.07 7.06 7.06 7.04 7.04 6.77 6.74 6.65 6.63 6.61 6.59 4.66 4.64 3.79 3.76 3.70 3.69 3.62 3.60 3.58 3.56 3.55 3.55 3.50 3.48 3.42 3.41 3.40 3.28 3.27 3.26 3.25
O
N
O
Br
4b
1H NMR (400 MHz, CDCl3)
2.19 5.71 1.35 1.01 1.00 1.10 1.03 7.22 1.22 1.14
f1 (ppm)

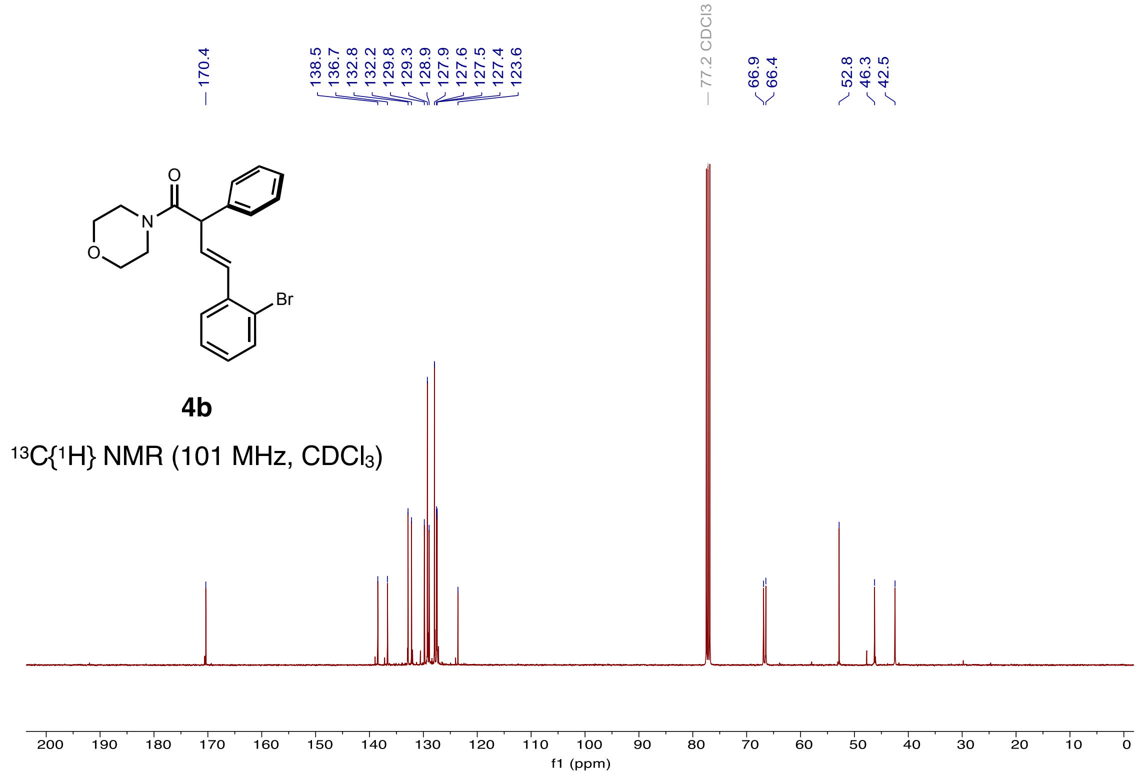
170.4 138.5 136.7 132.8 132.2 129.8 129.3 128.9 127.9 127.6 127.5 127.4 123.6 77.2 CDCl3 66.9 66.4 52.8 46.3 42.5
O
N
O
Br
4b
13C{1H} NMR (101 MHz, CDCl3)
f1 (ppm)

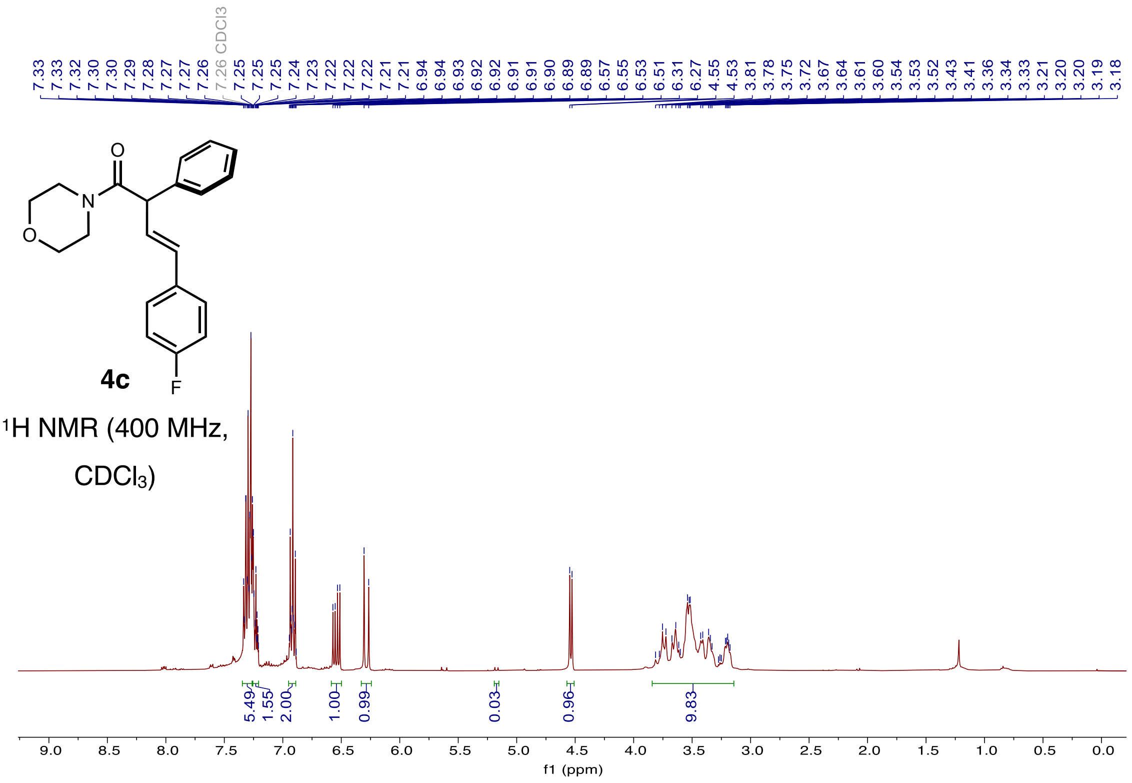
7.33 7.33 7.32 7.30 7.30 7.29 7.28 7.27 7.27 7.26 7.26 CDCl3 7.25 7.25 7.25 7.24 7.23 7.22 7.22 7.22 7.21 7.21 6.94 6.94 6.93 6.92 6.92 6.91 6.91 6.90 6.89 6.89 6.57 6.55 6.53 6.51 6.31 6.27 4.55 4.53 3.81 3.78 3.75 3.72 3.67 3.64 3.61 3.60 3.54 3.53 3.52 3.43 3.41 3.36 3.34 3.33 3.21 3.20 3.19 3.18
4c
^{1}H NMR (400 MHz, CDCl$_3$)
5.49 1.55 2.00 1.00 0.99 0.03 0.96 9.83
f1 (ppm)

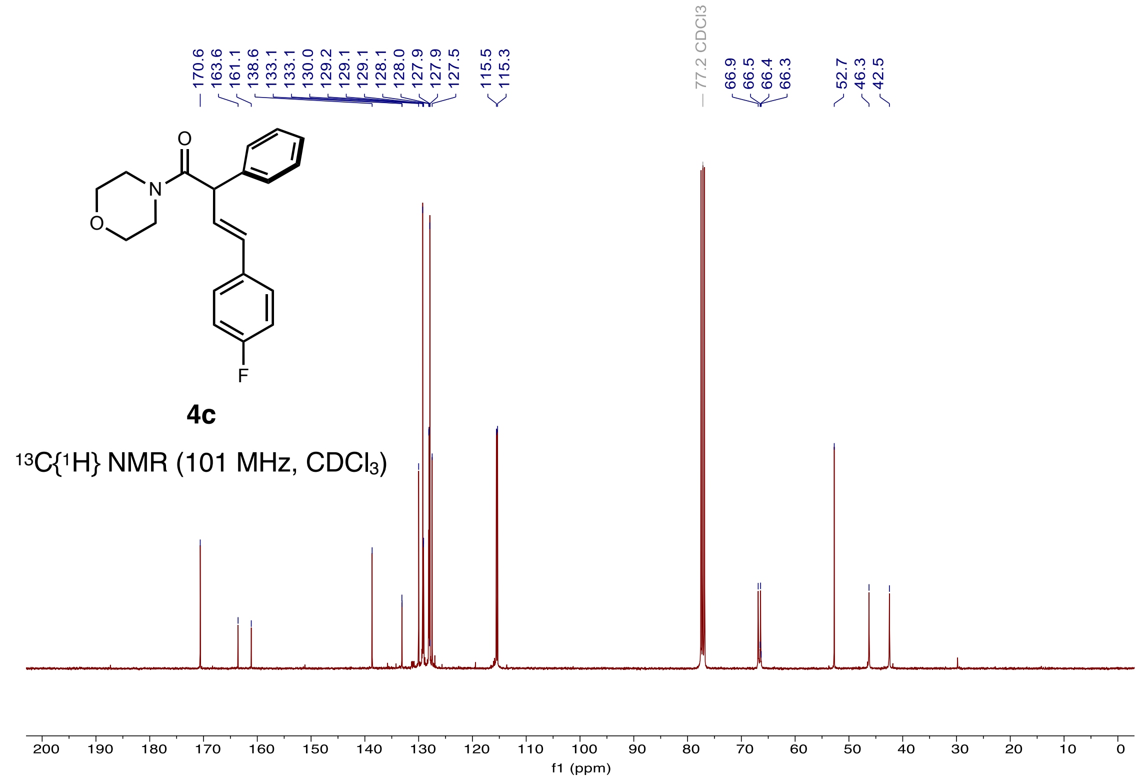
170.6 163.6 161.1 138.6 133.1 130.0 129.2 129.1 129.1 128.1 128.0 127.9 127.9 127.5 115.5 115.3
77.2 CDCl3
66.9 66.5 66.4 66.3
52.7 46.3 42.5
4c
^{13}C{^{1}H} NMR (101 MHz, CDCl$_3$)
f1 (ppm)

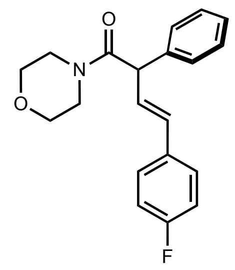

4c

^{19}F NMR (376 MHz, $CDCl_3$)

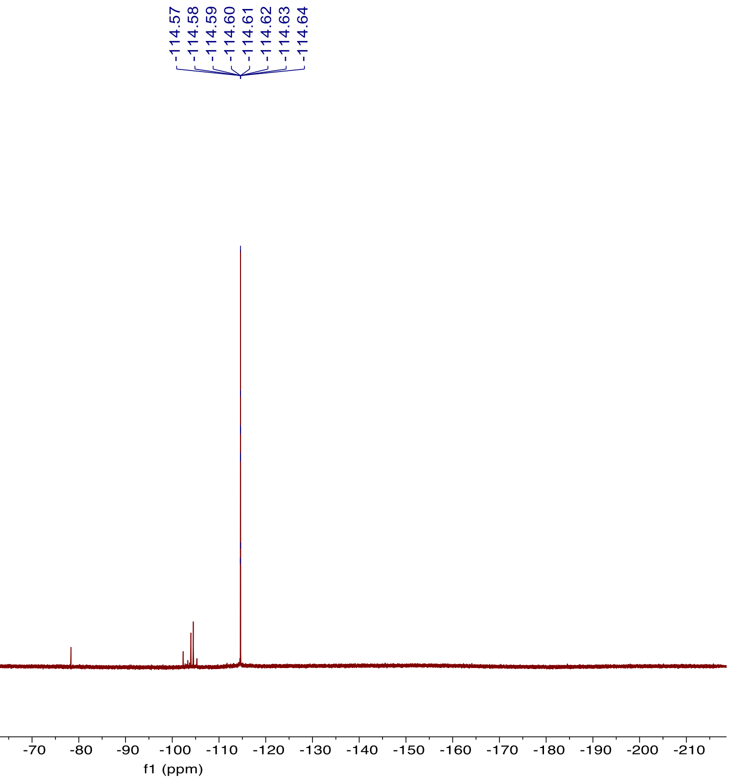

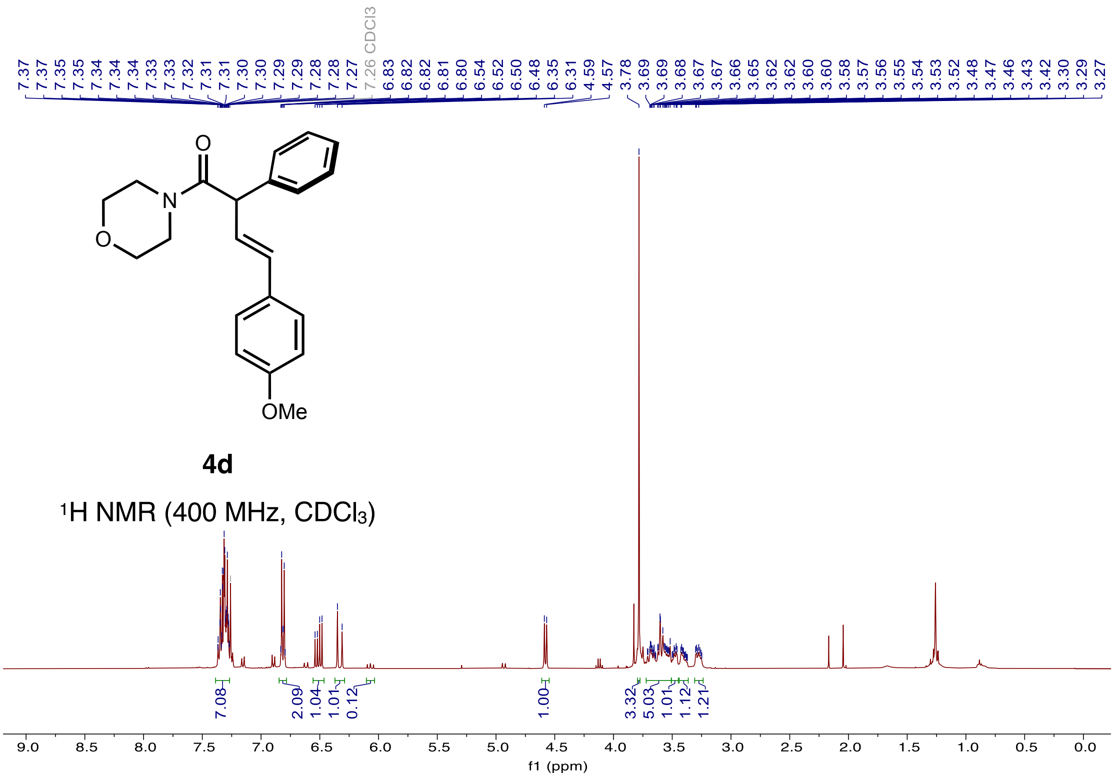
7.37 7.37 7.35 7.34 7.34 7.34 7.33 7.33 7.32 7.31 7.31 7.30 7.29 7.29 7.28 7.28 7.27 7.26 CDCl3 6.83 6.82 6.82 6.81 6.80 6.54 6.52 6.50 6.48 6.35 6.31 4.59 4.57 3.78 3.69 3.69 3.68 3.67 3.67 3.66 3.65 3.62 3.62 3.60 3.60 3.58 3.57 3.56 3.55 3.54 3.53 3.52 3.48 3.47 3.46 3.43 3.42 3.30 3.29 3.27
O
N
O
OMe
4d
^{1}H NMR (400 MHz, CDCl$_3$)
7.08 2.09 1.04 1.01 0.12 1.00 3.32 5.03 1.01 1.12 1.21
9.0 8.5 8.0 7.5 7.0 6.5 6.0 5.5 5.0 4.5 4.0 3.5 3.0 2.5 2.0 1.5 1.0 0.5 0.0
f1 (ppm)

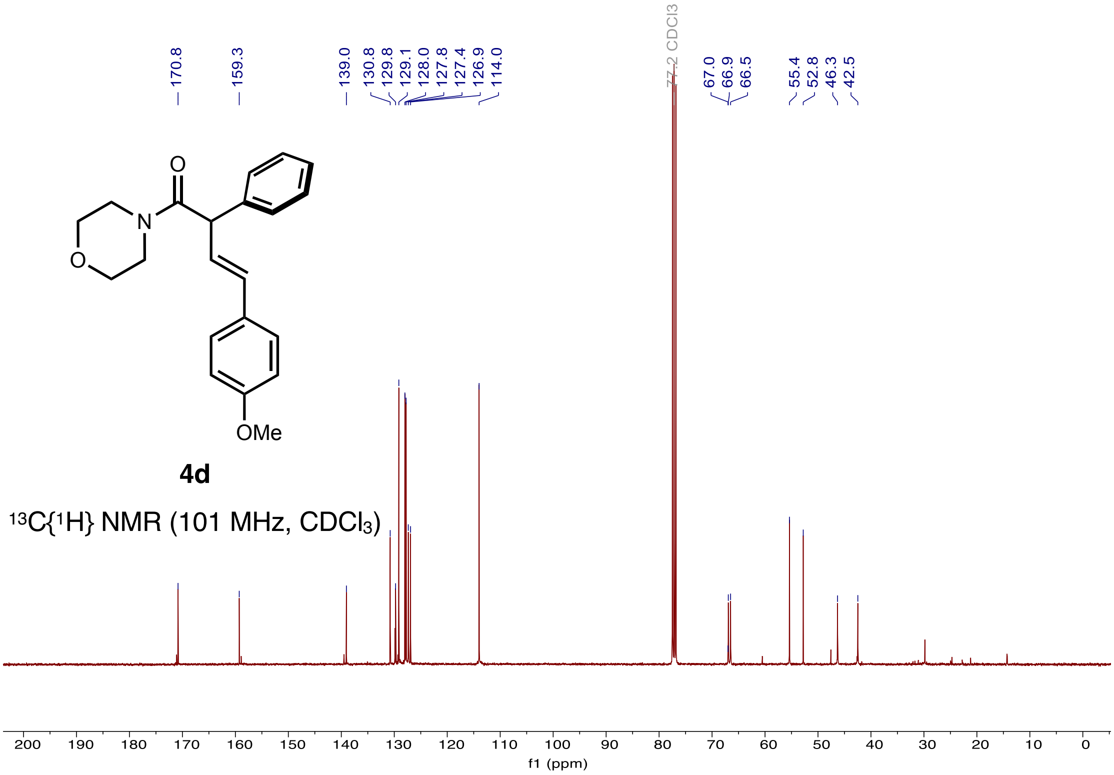
170.8 159.3 139.0 130.8 129.8 129.1 128.0 127.8 127.4 126.9 114.0 77.2 CDCl3 67.0 66.9 66.5 55.4 52.8 46.3 42.5
O
N
O
OMe
4d
^{13}C{^{1}H} NMR (101 MHz, CDCl$_3$)
200 190 180 170 160 150 140 130 120 110 100 90 80 70 60 50 40 30 20 10 0
f1 (ppm)

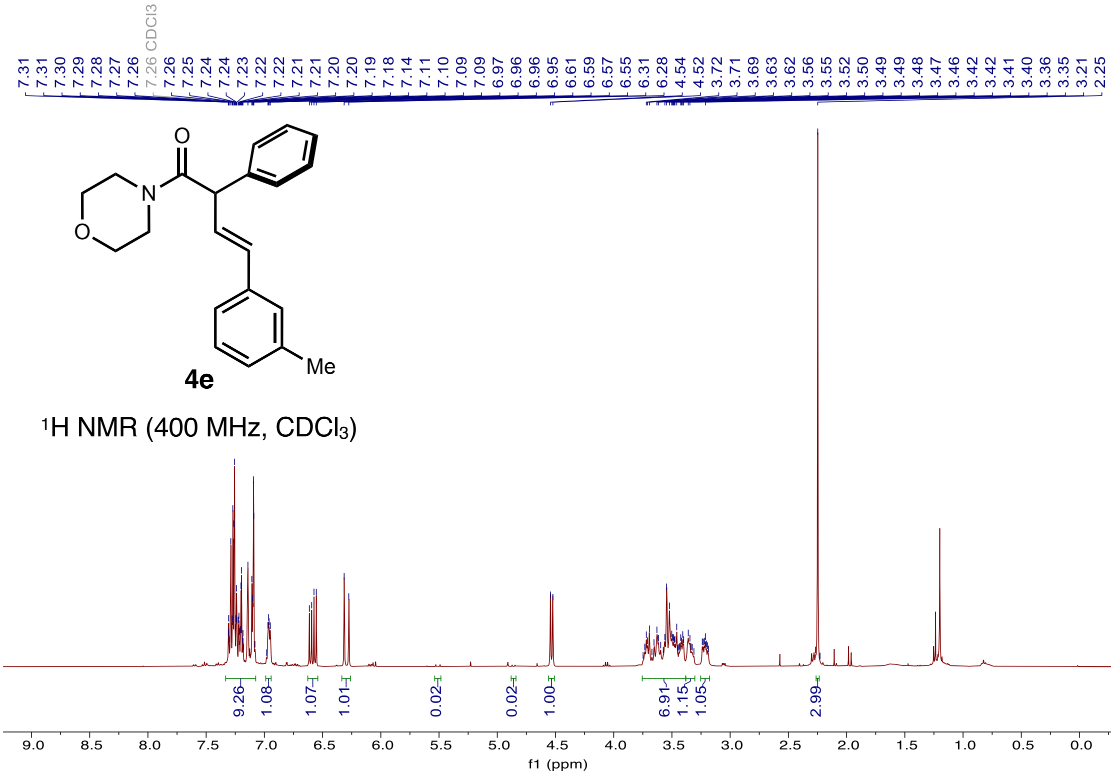
7.31 7.31 7.30 7.29 7.28 7.27 7.26 7.26 CDCl3 7.26 7.25 7.24 7.24 7.23 7.22 7.22 7.21 7.21 7.20 7.20 7.19 7.18 7.14 7.11 7.10 7.09 7.09 6.97 6.96 6.96 6.95 6.61 6.59 6.57 6.55 6.31 6.28 4.54 4.52 3.72 3.71 3.69 3.63 3.62 3.56 3.55 3.52 3.50 3.49 3.48 3.47 3.46 3.42 3.42 3.41 3.40 3.36 3.35 3.21 2.25
O
N
O
Me
4e
^{1}H NMR (400 MHz, CDCl$_3$)
9.26
1.08
1.07
1.01
0.02
0.02
1.00
6.91
1.15
1.05
2.99
9.0 8.5 8.0 7.5 7.0 6.5 6.0 5.5 5.0 4.5 4.0 3.5 3.0 2.5 2.0 1.5 1.0 0.5 0.0
f1 (ppm)

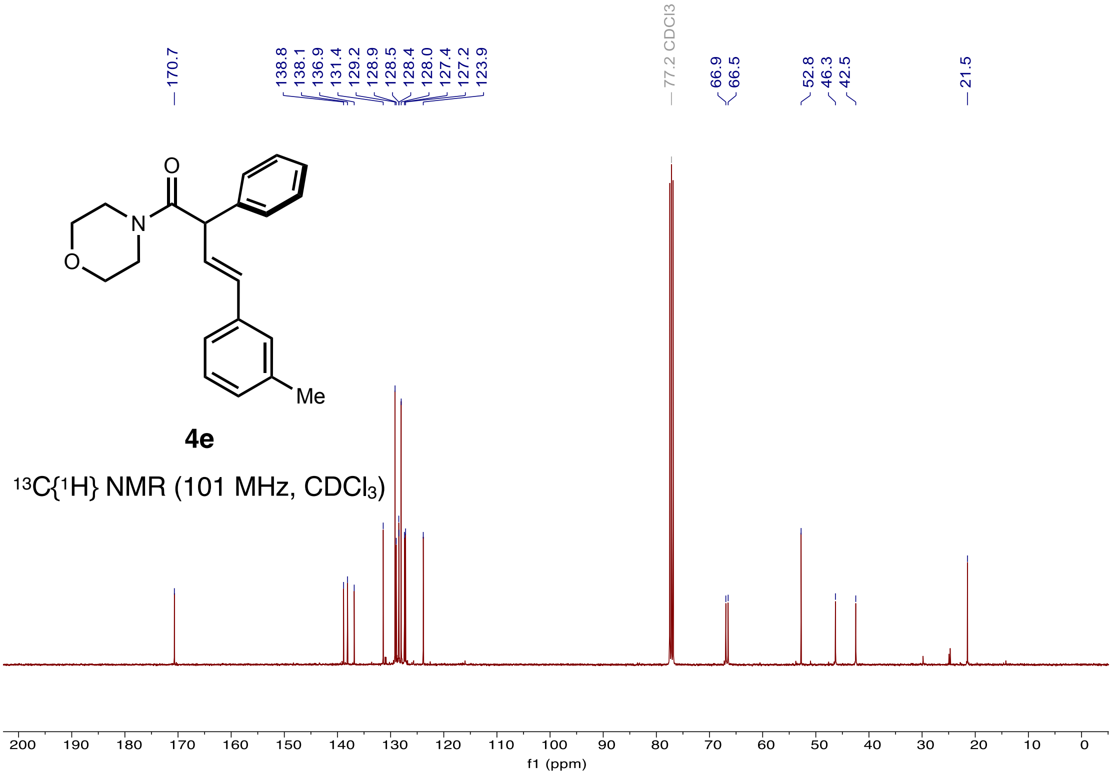
170.7
138.8 138.1 136.9 131.4 129.2 128.9 128.5 128.4 128.0 127.4 127.2 123.9
77.2 CDCl3
66.9 66.5
52.8 46.3 42.5
21.5
O
N
O
Me
4e
^{13}C{^{1}H} NMR (101 MHz, CDCl$_3$)
200 190 180 170 160 150 140 130 120 110 100 90 80 70 60 50 40 30 20 10 0
f1 (ppm)

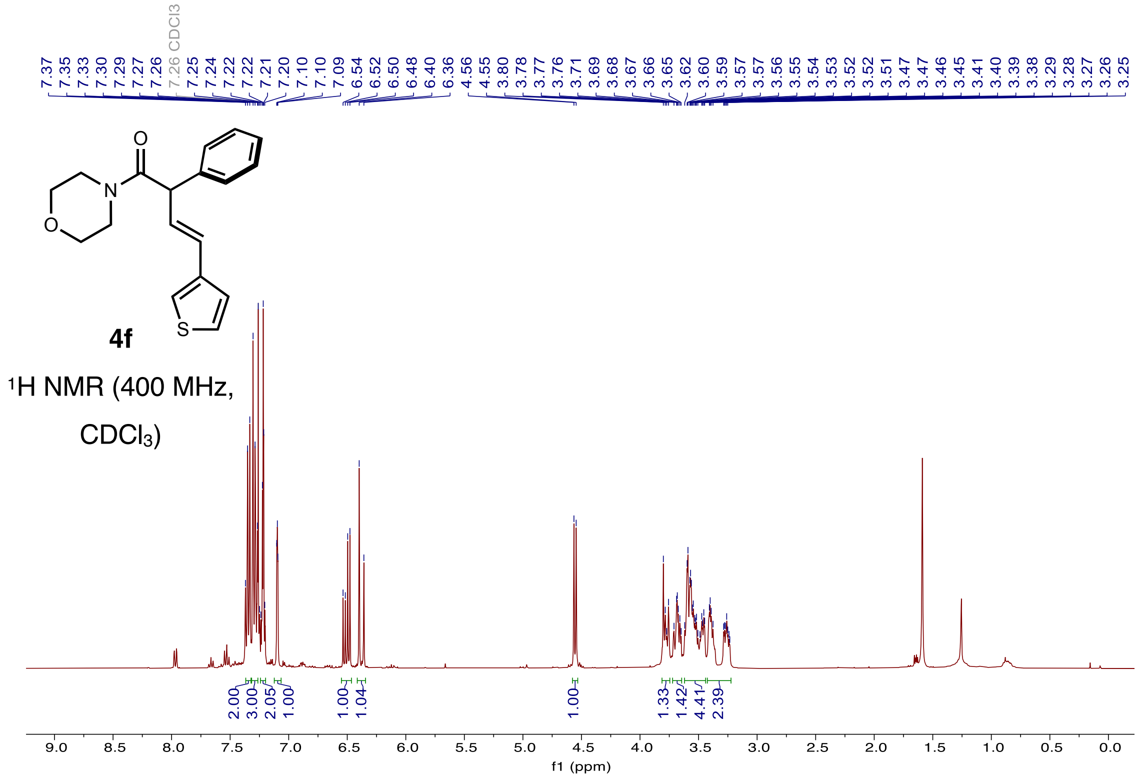
7.37 7.35 7.33 7.30 7.29 7.27 7.26 7.26 CDCl3 7.25 7.24 7.22 7.22 7.21 7.20 7.10 7.09 6.54 6.52 6.50 6.48 6.40 6.36 4.56 4.55 3.80 3.78 3.77 3.76 3.71 3.69 3.68 3.67 3.66 3.65 3.62 3.60 3.59 3.57 3.56 3.55 3.54 3.53 3.52 3.52 3.51 3.47 3.47 3.46 3.45 3.41 3.40 3.39 3.38 3.29 3.28 3.27 3.26 3.25
O
N
O
S
4f
1H NMR (400 MHz, CDCl3)
2.00 3.00 2.05 1.00 1.00 1.04 1.00 1.33 1.42 4.41 2.39
9.0 8.5 8.0 7.5 7.0 6.5 6.0 5.5 5.0 4.5 4.0 3.5 3.0 2.5 2.0 1.5 1.0 0.5 0.0
f1 (ppm)

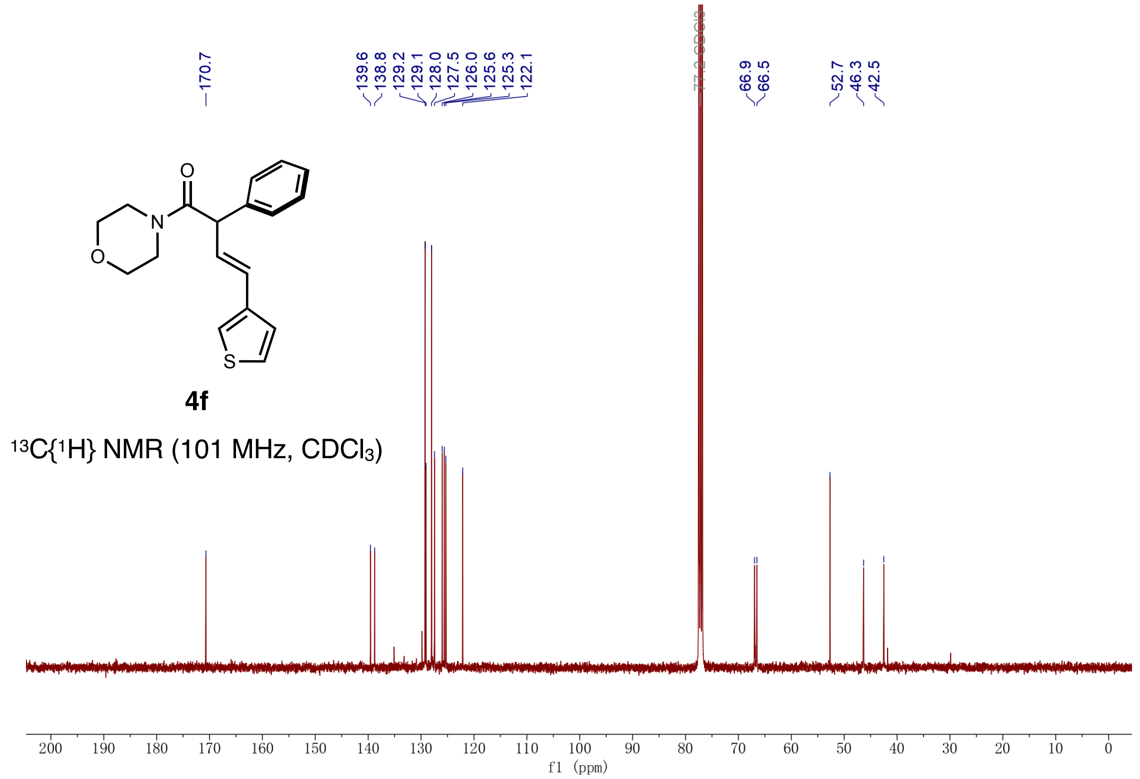
170.7
139.6 138.8 129.2 129.1 128.0 127.5 126.0 125.6 125.3 122.1
77.2 CDCl3
66.9 66.5
52.7 46.3 42.5
O
N
O
S
4f
13C{1H} NMR (101 MHz, CDCl3)
200 190 180 170 160 150 140 130 120 110 100 90 80 70 60 50 40 30 20 10 0
f1 (ppm)

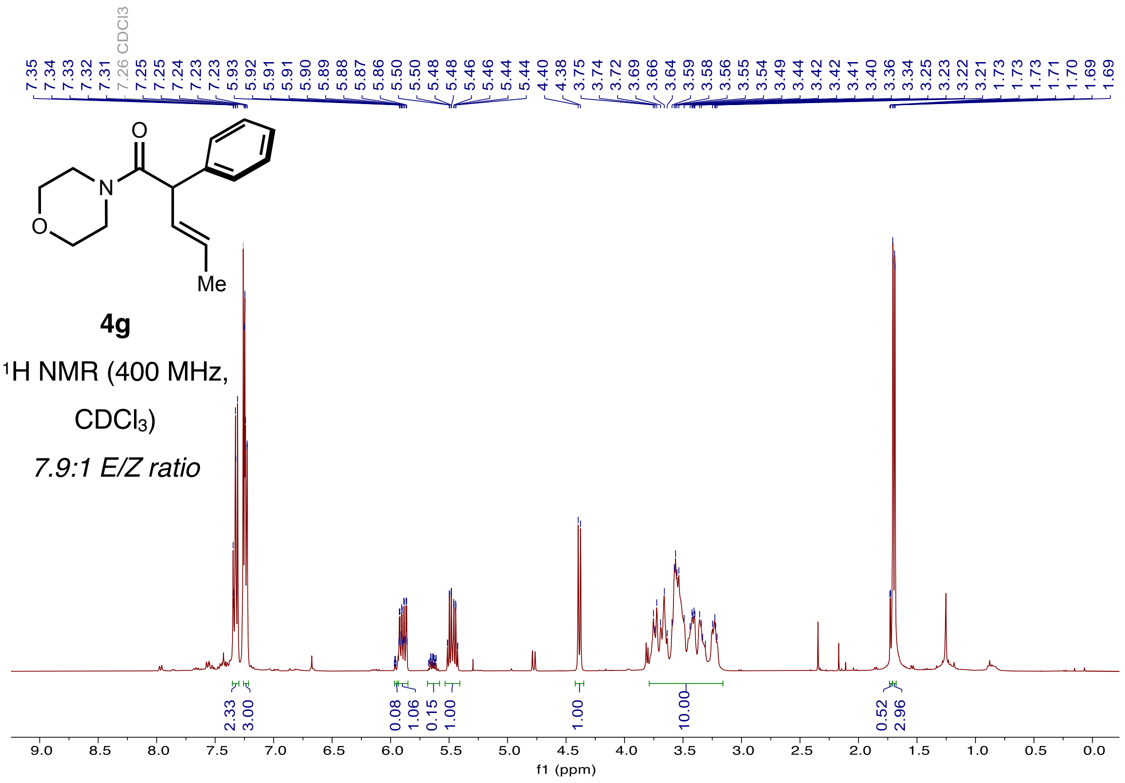
7.35 7.34 7.33 7.32 7.31 7.26 CDCl3 7.25 7.25 7.24 7.23 7.23 5.93 5.92 5.91 5.91 5.90 5.89 5.88 5.87 5.86 5.50 5.48 5.48 5.46 5.46 5.44 5.44 4.40 4.38 3.75 3.74 3.72 3.69 3.66 3.64 3.59 3.58 3.56 3.55 3.54 3.49 3.44 3.42 3.42 3.41 3.40 3.36 3.34 3.25 3.23 3.22 3.21 1.73 1.73 1.73 1.71 1.70 1.69 1.69
O
N
O
Me
4g
1H NMR (400 MHz, CDCl3)
7.9:1 E/Z ratio
2.33 3.00 0.08 1.06 0.15 1.00 1.00 10.00 0.52 2.96
9.0 8.5 8.0 7.5 7.0 6.5 6.0 5.5 5.0 4.5 4.0 3.5 3.0 2.5 2.0 1.5 1.0 0.5 0.0
f1 (ppm)

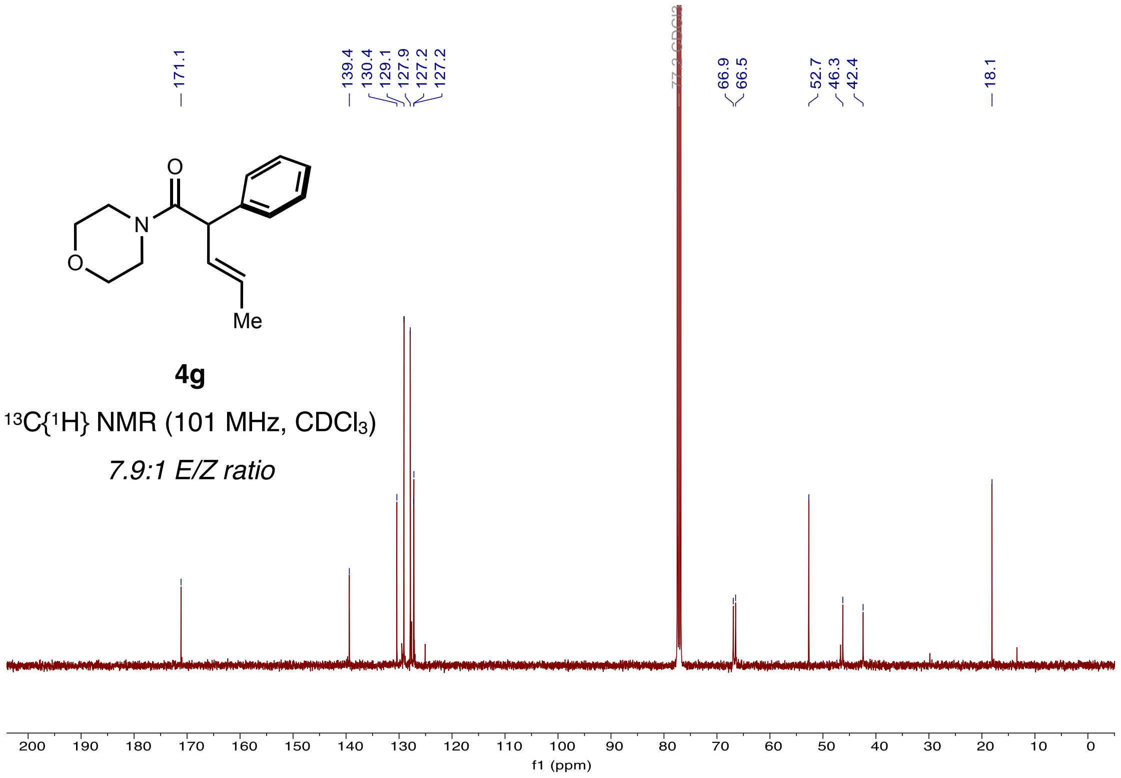
171.1 139.4 130.4 129.1 127.9 127.2 127.2 77.2 CDCl3 66.9 66.5 52.7 46.3 42.4 18.1
O
N
O
Me
4g
13C{1H} NMR (101 MHz, CDCl3)
7.9:1 E/Z ratio
200 190 180 170 160 150 140 130 120 110 100 90 80 70 60 50 40 30 20 10 0
f1 (ppm)

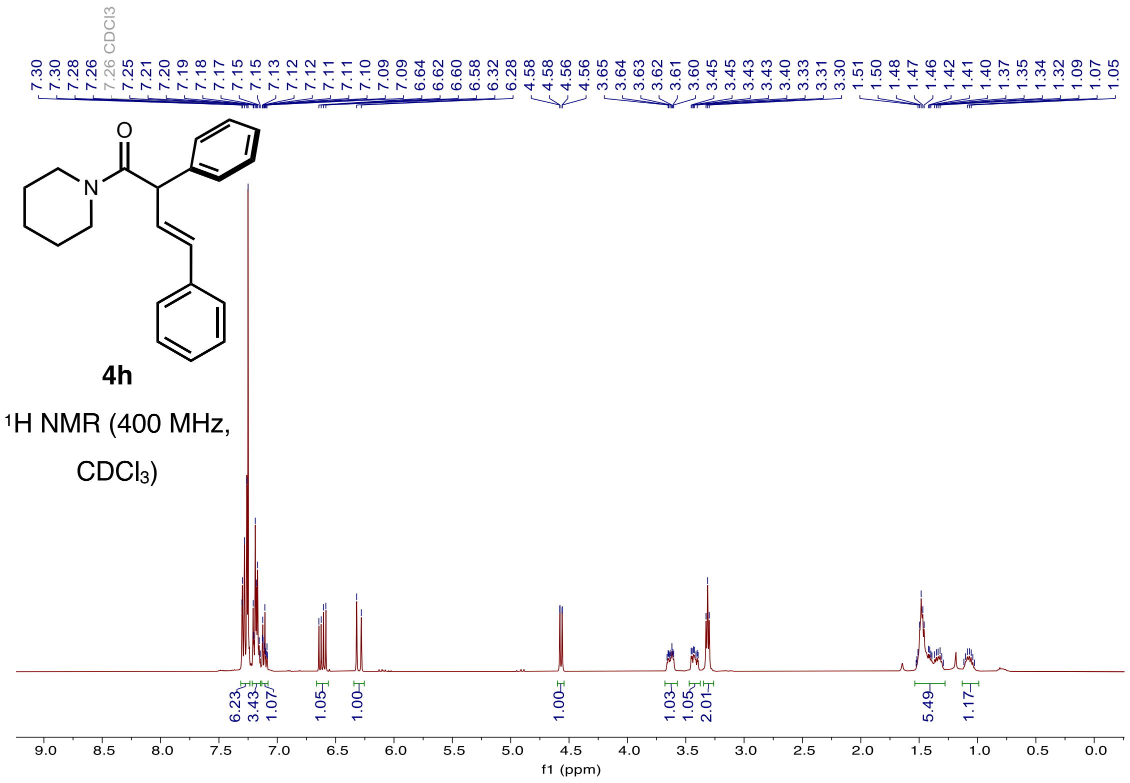

7.30 7.30 7.28 7.26 7.26 CDCl3 7.25 7.21 7.20 7.19 7.18 7.17 7.15 7.15 7.13 7.12 7.12 7.11 7.11 7.10 7.09 6.64 6.62 6.60 6.58 6.32 6.28 4.58 4.58 4.56 4.56 3.65 3.64 3.63 3.62 3.61 3.60 3.45 3.45 3.43 3.43 3.40 3.33 3.31 3.30 1.51 1.50 1.48 1.47 1.46 1.42 1.41 1.40 1.37 1.35 1.34 1.32 1.09 1.07 1.05
O
N
4h
¹H NMR (400 MHz, CDCl3)
6.23 3.43 1.07 1.05 1.00 1.00 1.03 1.05 2.01 5.49 1.17
f1 (ppm)

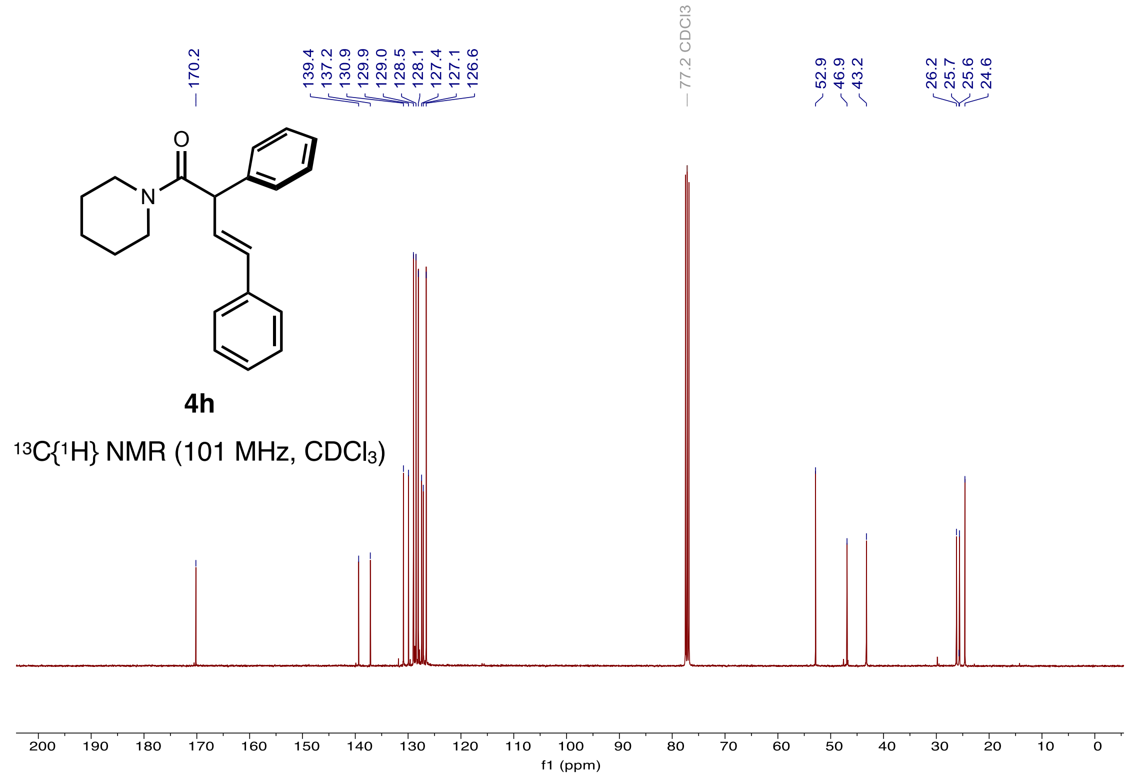

170.2
139.4 137.2 130.9 129.9 129.0 128.5 128.1 127.4 127.1 126.6
77.2 CDCl3
52.9 46.9 43.2
26.2 25.7 25.6 24.6
O
N
4h
¹³C{¹H} NMR (101 MHz, CDCl3)
f1 (ppm)

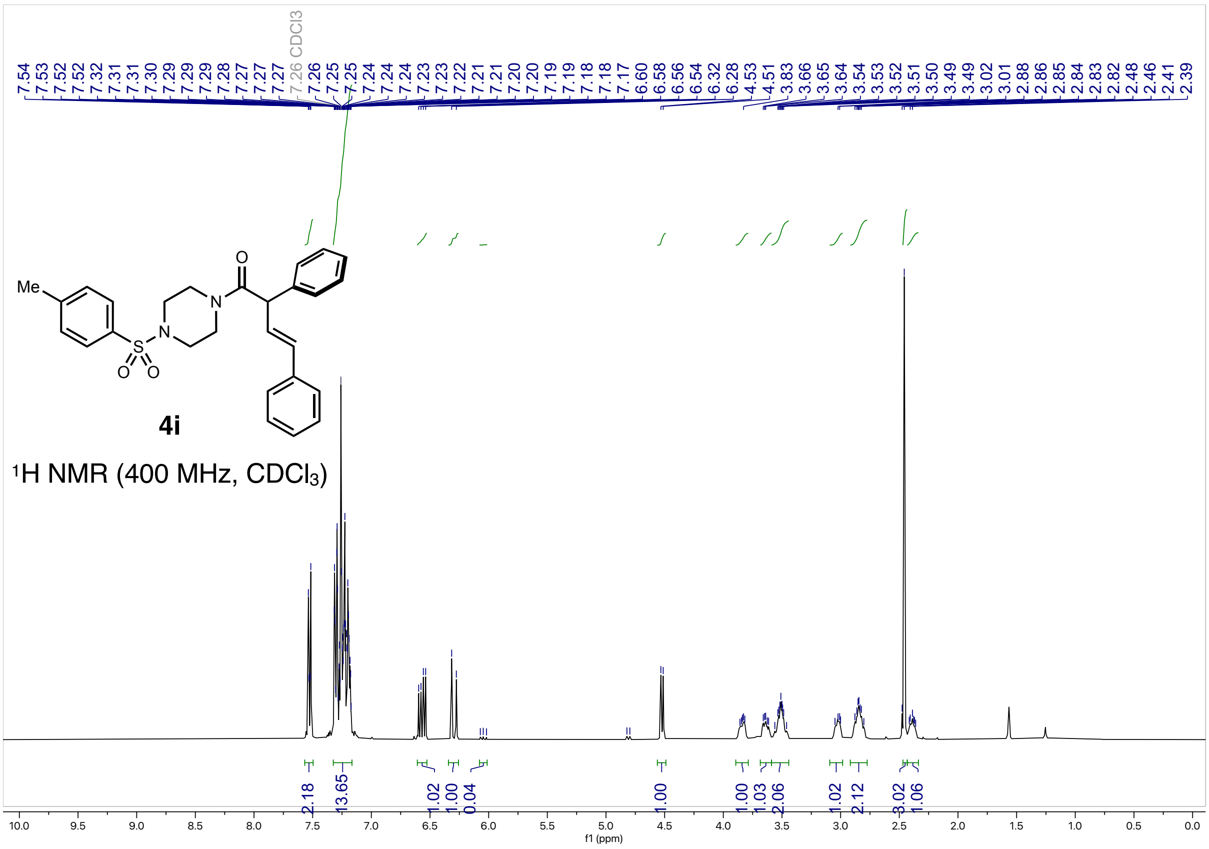
7.54 7.53 7.52 7.32 7.31 7.30 7.29 7.29 7.29 7.28 7.27 7.27 7.27 7.26 CDCl3 7.26 7.25 7.25 7.24 7.24 7.24 7.23 7.23 7.22 7.21 7.21 7.20 7.20 7.19 7.19 7.18 7.18 7.17 6.60 6.58 6.56 6.54 6.32 6.28 4.53 4.51 3.83 3.66 3.65 3.64 3.54 3.53 3.52 3.51 3.50 3.49 3.49 3.02 3.01 2.88 2.86 2.85 2.84 2.83 2.82 2.48 2.46 2.41 2.39
Me
N
N
O
S
O
O
4i
1H NMR (400 MHz, CDCl3)
2.18 13.65 1.02 1.00 0.04 1.00 1.00 1.03 2.06 1.02 2.12 3.02 1.06
10.0 9.5 9.0 8.5 8.0 7.5 7.0 6.5 6.0 5.5 5.0 4.5 4.0 3.5 3.0 2.5 2.0 1.5 1.0 0.5 0.0
f1 (ppm)

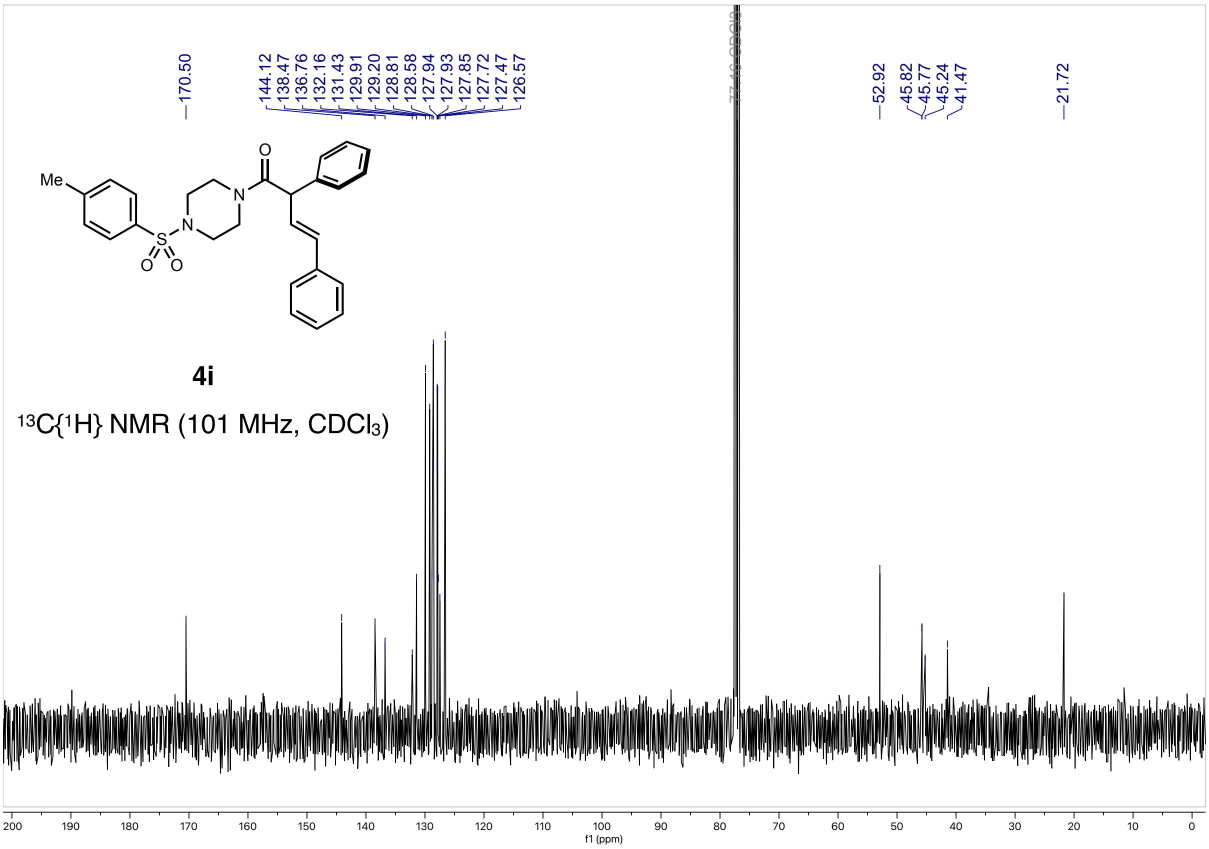
170.50
144.12 138.47 136.76 132.16 131.43 129.91 129.20 128.81 128.58 127.94 127.93 127.85 127.72 127.47 126.57
77.48 CDCl3
52.92 45.82 45.77 45.24 41.47
21.72
Me
N
N
O
S
O
O
4i
13C{1H} NMR (101 MHz, CDCl3)
200 190 180 170 160 150 140 130 120 110 100 90 80 70 60 50 40 30 20 10 0
f1 (ppm)

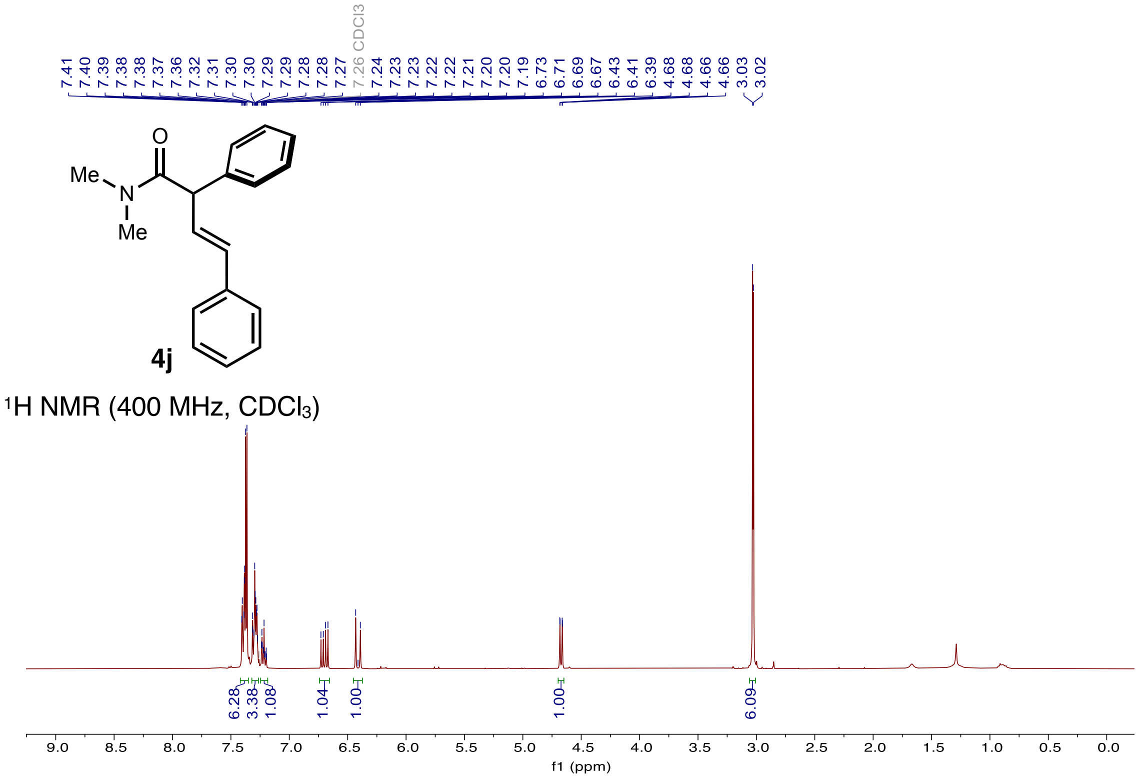

4j

^{1}H NMR (400 MHz, $CDCl_3$)

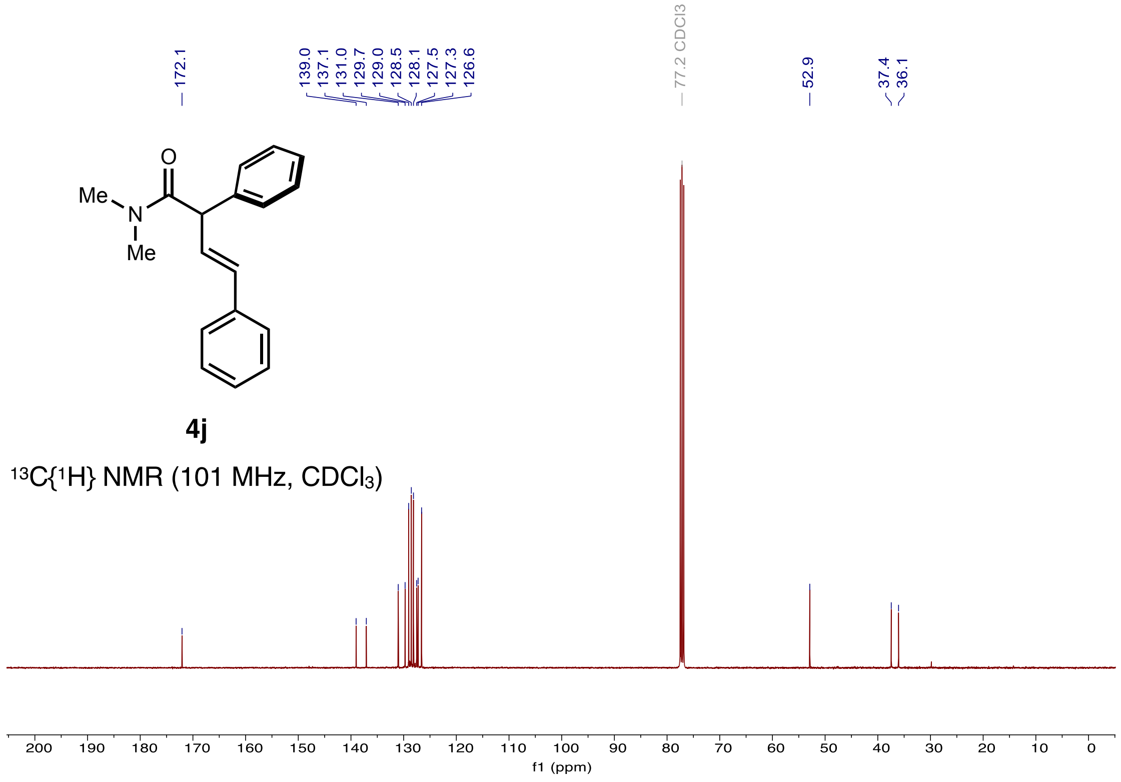

4j

$^{13}C\{^{1}H\}$ NMR (101 MHz, $CDCl_3$)

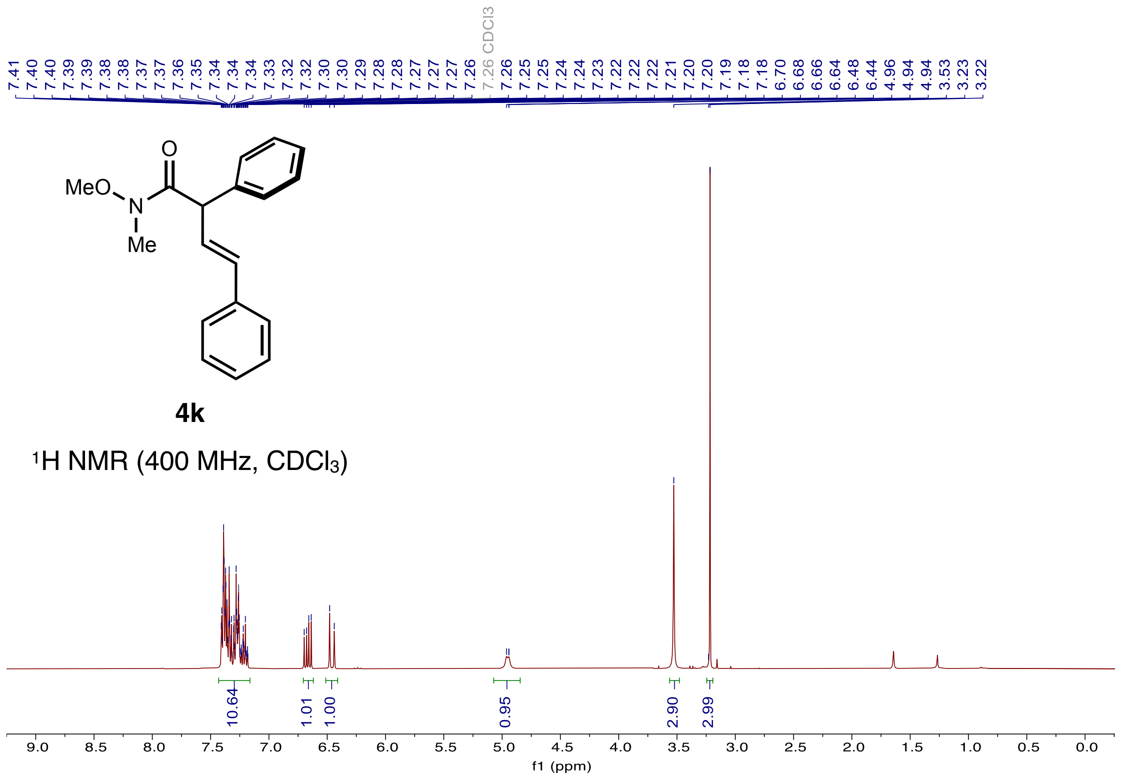
7.41 7.40 7.39 7.39 7.38 7.38 7.37 7.37 7.36 7.35 7.34 7.34 7.34 7.33 7.32 7.32 7.30 7.30 7.29 7.28 7.28 7.27 7.27 7.27 7.26 7.26 CDCl3 7.26 7.25 7.25 7.24 7.24 7.23 7.22 7.22 7.22 7.22 7.21 7.20 7.20 7.19 7.18 6.70 6.68 6.66 6.64 6.48 6.44 4.96 4.94 3.53 3.23 3.22
MeO
N
Me
O
4k
1H NMR (400 MHz, CDCl3)
10.64
1.01
1.00
0.95
2.90
2.99
9.0 8.5 8.0 7.5 7.0 6.5 6.0 5.5 5.0 4.5 4.0 3.5 3.0 2.5 2.0 1.5 1.0 0.5 0.0
f1 (ppm)

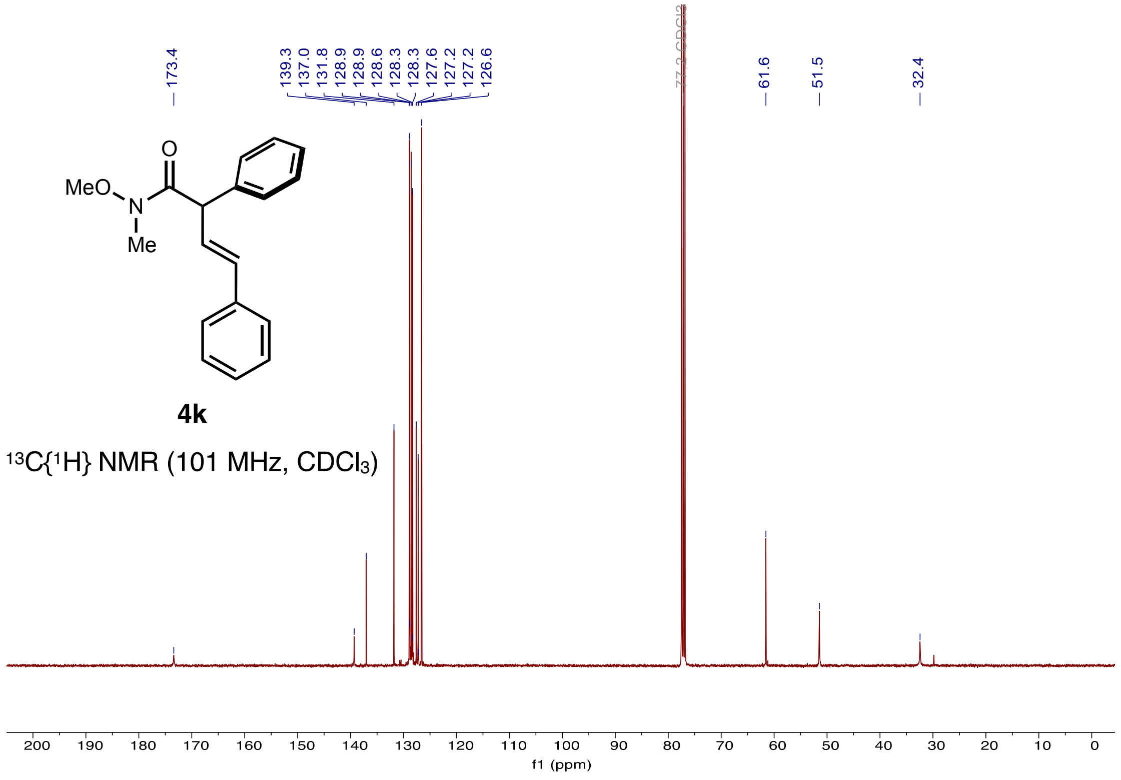
173.4
139.3 137.0 131.8 128.9 128.9 128.6 128.3 128.3 127.6 127.2 127.2 126.6
77.3 CDCl3
61.6
51.5
32.4
MeO
N
Me
O
4k
13C{1H} NMR (101 MHz, CDCl3)
200 190 180 170 160 150 140 130 120 110 100 90 80 70 60 50 40 30 20 10 0
f1 (ppm)

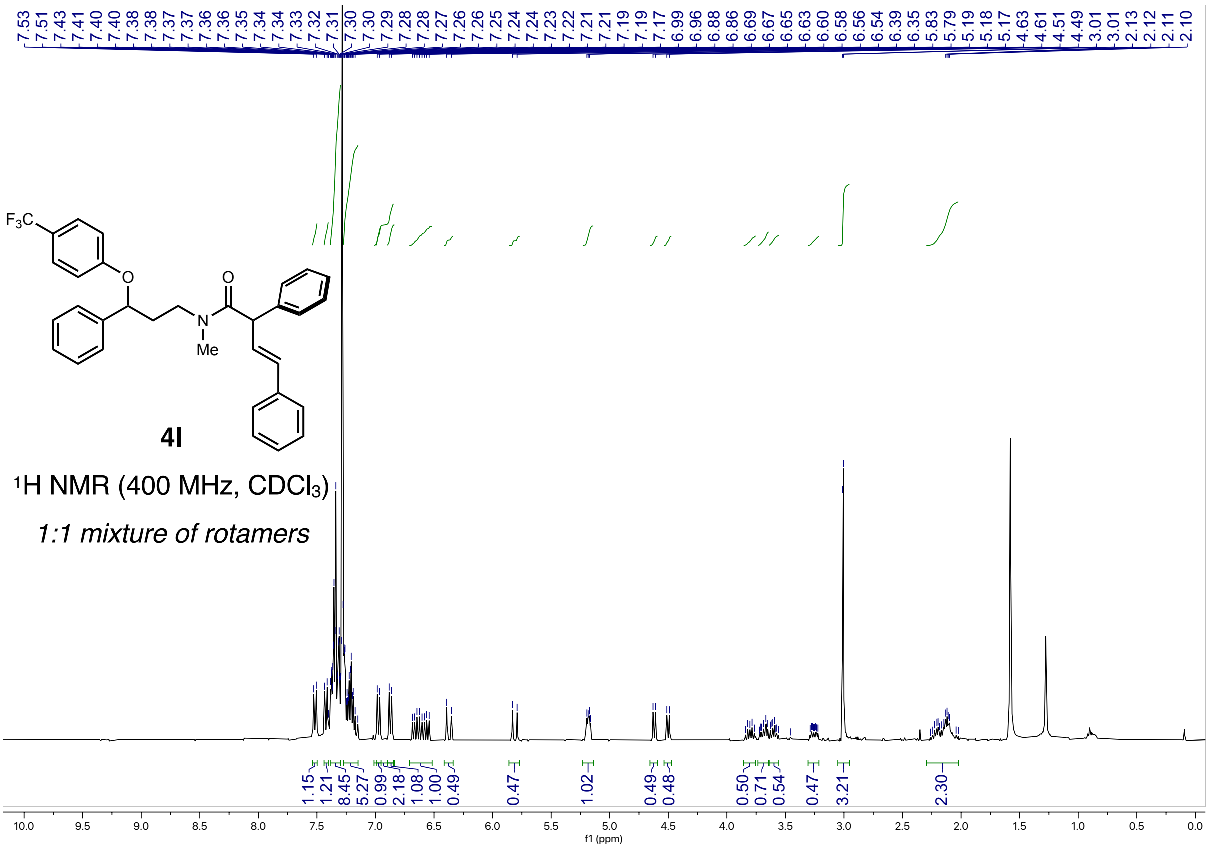
7.53 7.51 7.43 7.41 7.40 7.40 7.38 7.38 7.37 7.37 7.36 7.36 7.35 7.34 7.34 7.33 7.32 7.31 7.30 7.29 7.28 7.28 7.27 7.26 7.26 7.25 7.24 7.24 7.23 7.22 7.21 7.21 7.19 7.19 7.17 6.99 6.96 6.88 6.86 6.69 6.67 6.65 6.63 6.60 6.58 6.56 6.54 6.39 6.35 5.83 5.79 5.19 5.18 5.17 4.63 4.61 4.51 4.49 3.01 2.13 2.12 2.11 2.10
F_3C
O
O
N
Me
4l
¹H NMR (400 MHz, CDCl₃)
1:1 mixture of rotamers
1.15 1.21 8.45 5.27 0.99 2.18 1.08 1.00 0.49 0.47 1.02 0.49 0.48 0.50 0.71 0.54 0.47 3.21 2.30
f1 (ppm)

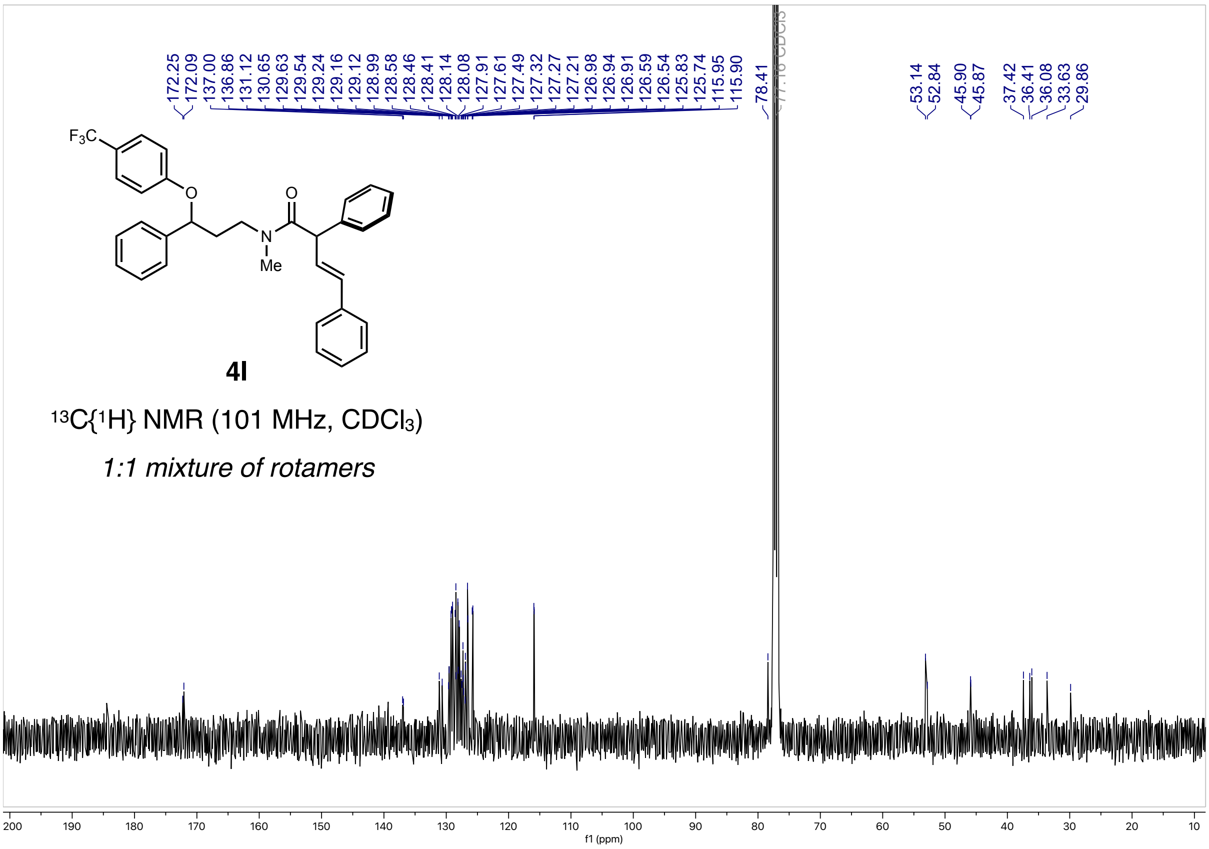
172.25 172.09 137.00 136.86 131.12 130.65 129.63 129.54 129.24 129.16 129.12 128.99 128.58 128.46 128.41 128.14 128.08 127.91 127.61 127.49 127.32 127.27 127.21 126.98 126.94 126.91 126.59 126.54 125.83 125.74 115.95 115.90 78.41 77.16 CDCl3 53.14 52.84 45.90 45.87 37.42 36.41 36.08 33.63 29.86
F_3C
O
O
N
Me
4l
¹³C{¹H} NMR (101 MHz, CDCl₃)
1:1 mixture of rotamers
f1 (ppm)

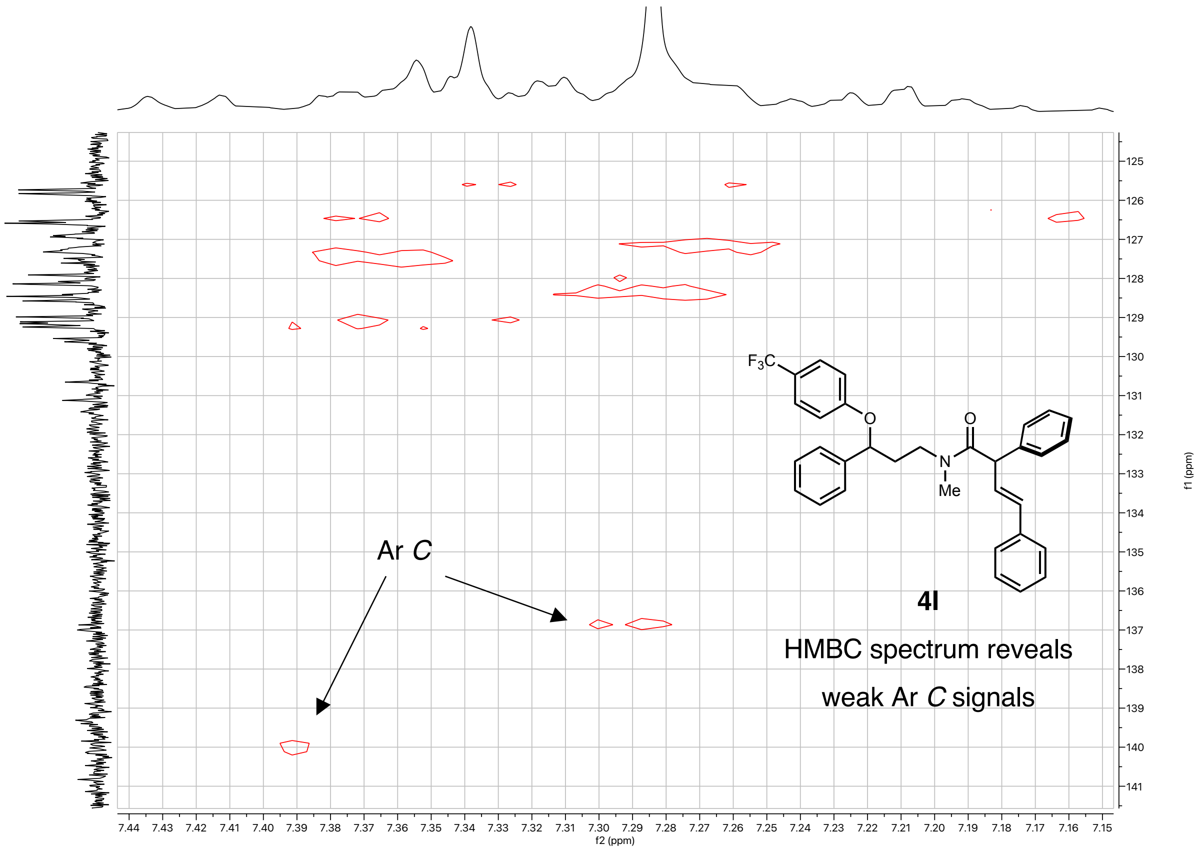
Ar C
F3C
O
O
N
Me
4l
HMBC spectrum reveals
weak Ar C signals
f2 (ppm)
f1 (ppm)

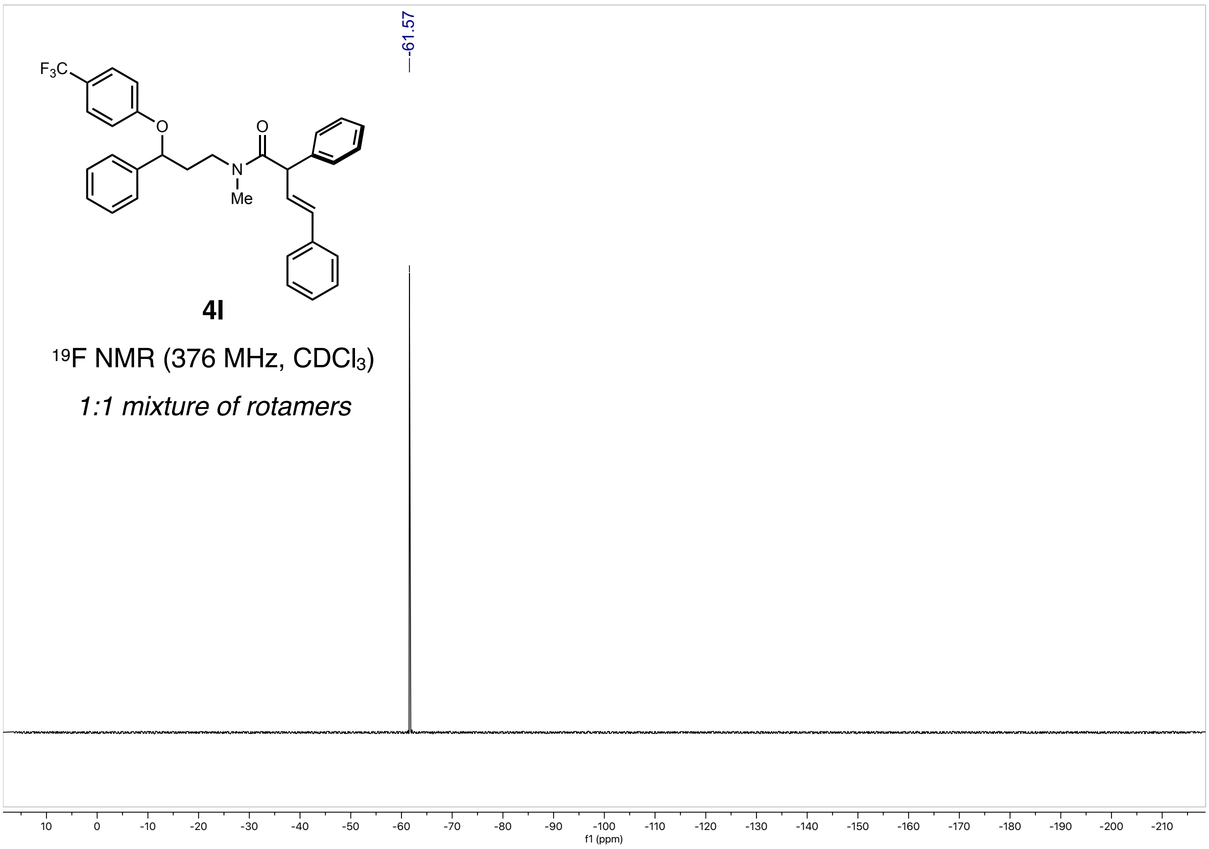
-61.57
F3C
O
O
N
Me
4l
19F NMR (376 MHz, CDCl3)
1:1 mixture of rotamers
f1 (ppm)

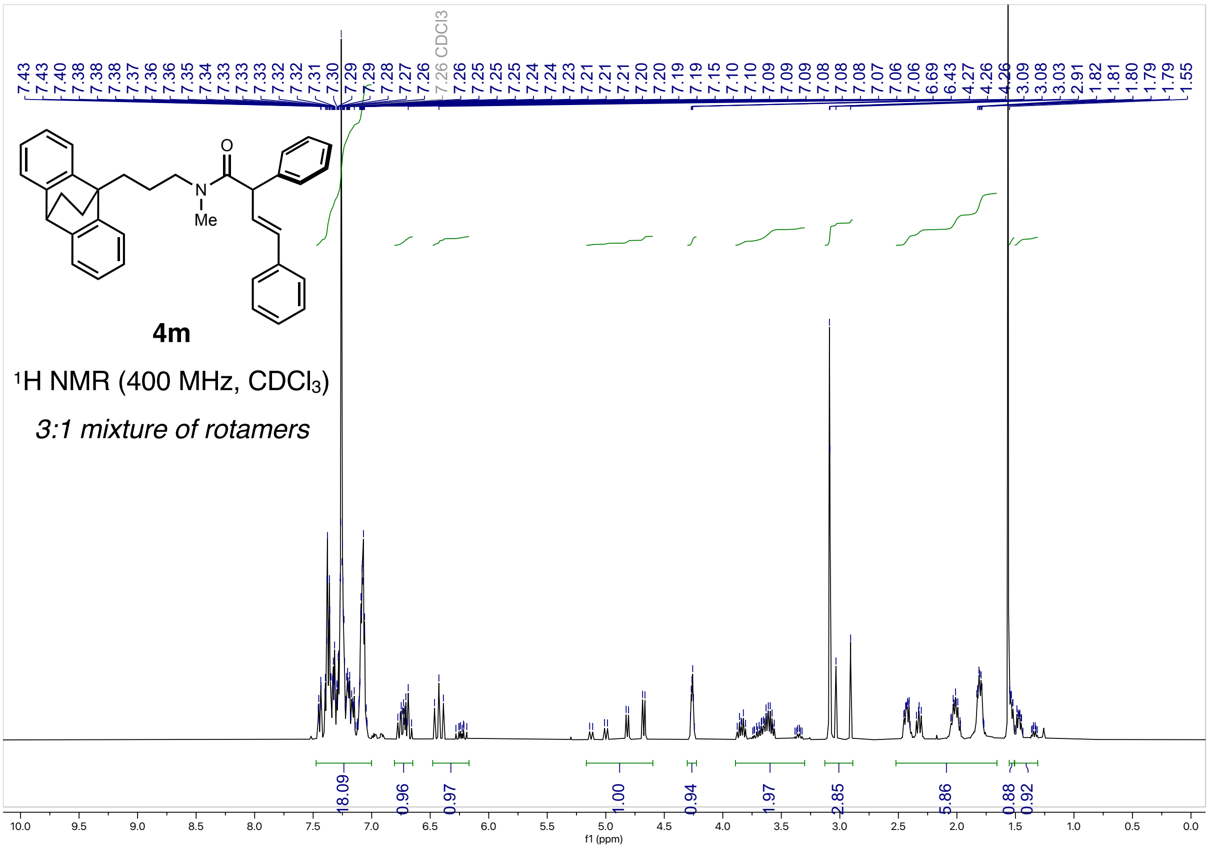

4m

^{1}H NMR (400 MHz, $CDCl_3$)

3:1 mixture of rotamers

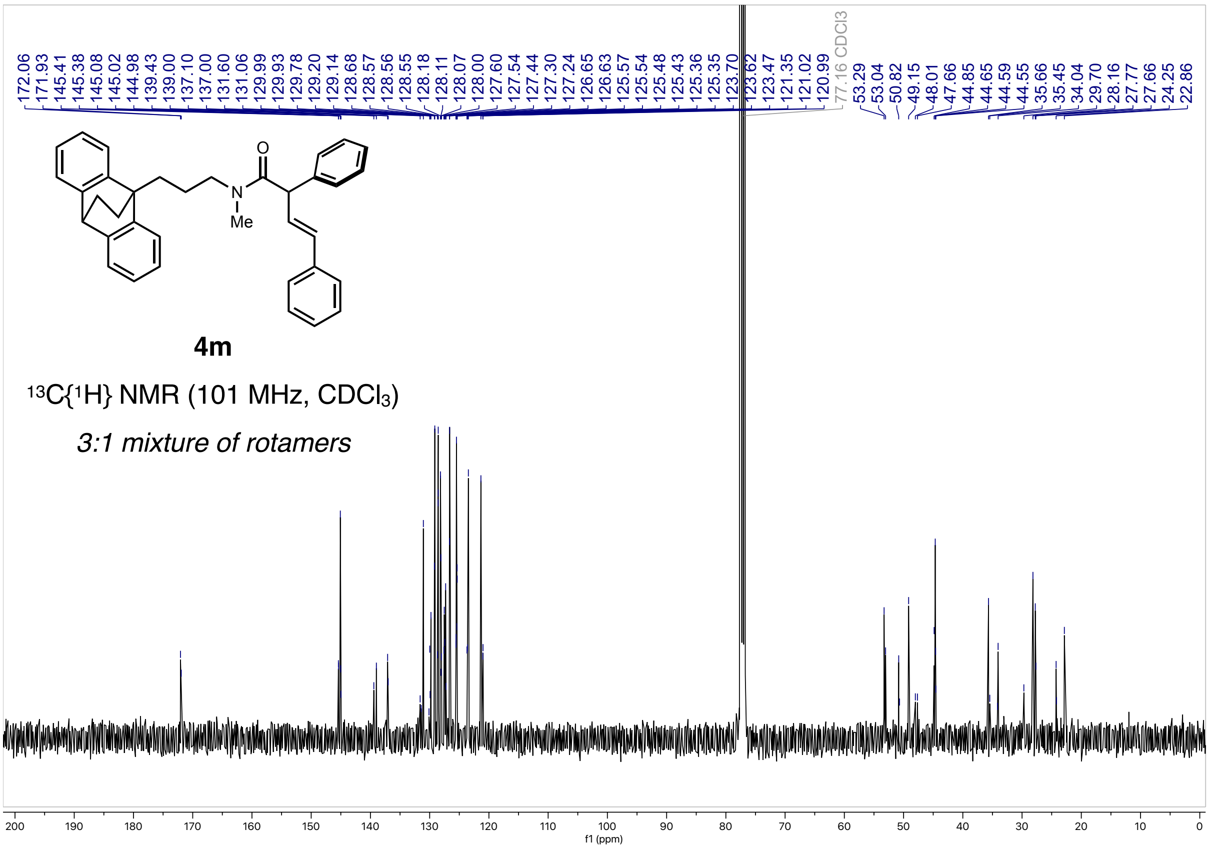

4m

$^{13}C\{^1H\}$ NMR (101 MHz, $CDCl_3$)

3:1 mixture of rotamers

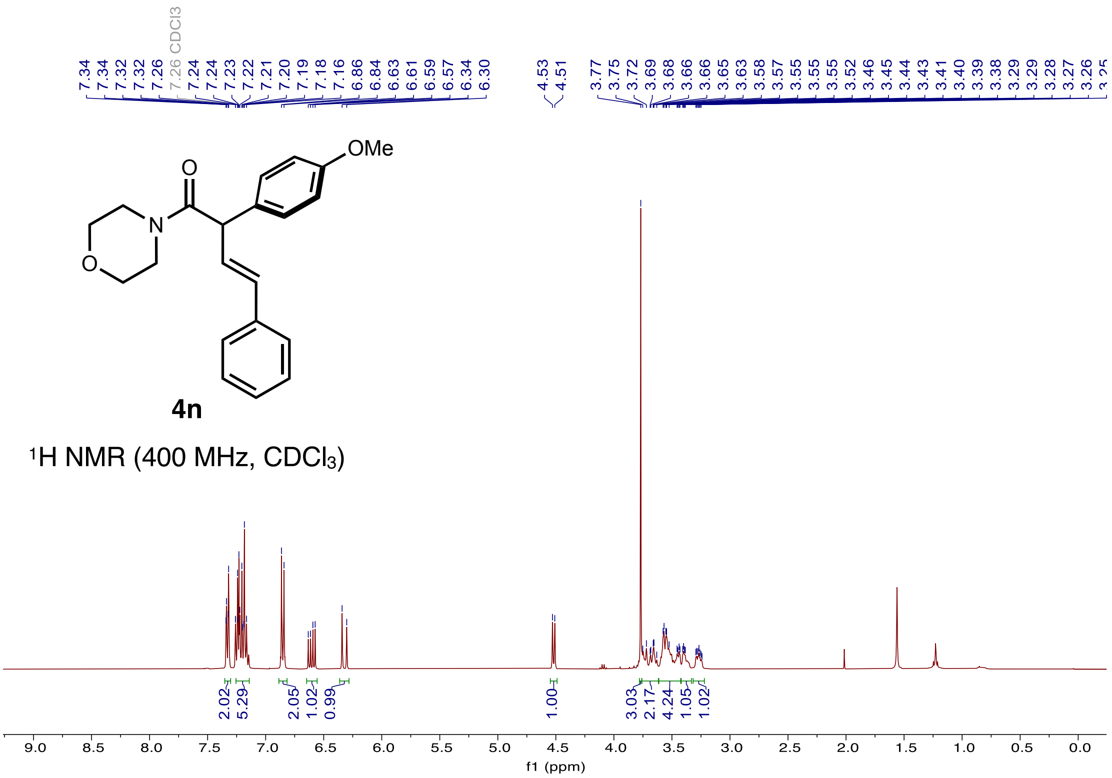
7.34
7.34
7.32
7.32
7.26
7.26 CDCl3
7.24
7.24
7.23
7.22
7.21
7.20
7.19
7.18
7.16
6.86
6.84
6.63
6.61
6.59
6.57
6.34
6.30
4.53
4.51
3.77
3.75
3.72
3.69
3.68
3.66
3.66
3.65
3.63
3.58
3.57
3.55
3.55
3.55
3.52
3.46
3.45
3.44
3.43
3.41
3.40
3.39
3.38
3.29
3.29
3.28
3.27
3.26
OMe
O
N
O
4n
^{1}H NMR (400 MHz, CDCl$_3$)
2.02
5.29
2.05
1.02
0.99
1.00
3.03
2.17
4.24
1.05
1.02
9.0 8.5 8.0 7.5 7.0 6.5 6.0 5.5 5.0 4.5 4.0 3.5 3.0 2.5 2.0 1.5 1.0 0.5 0.0
f1 (ppm)

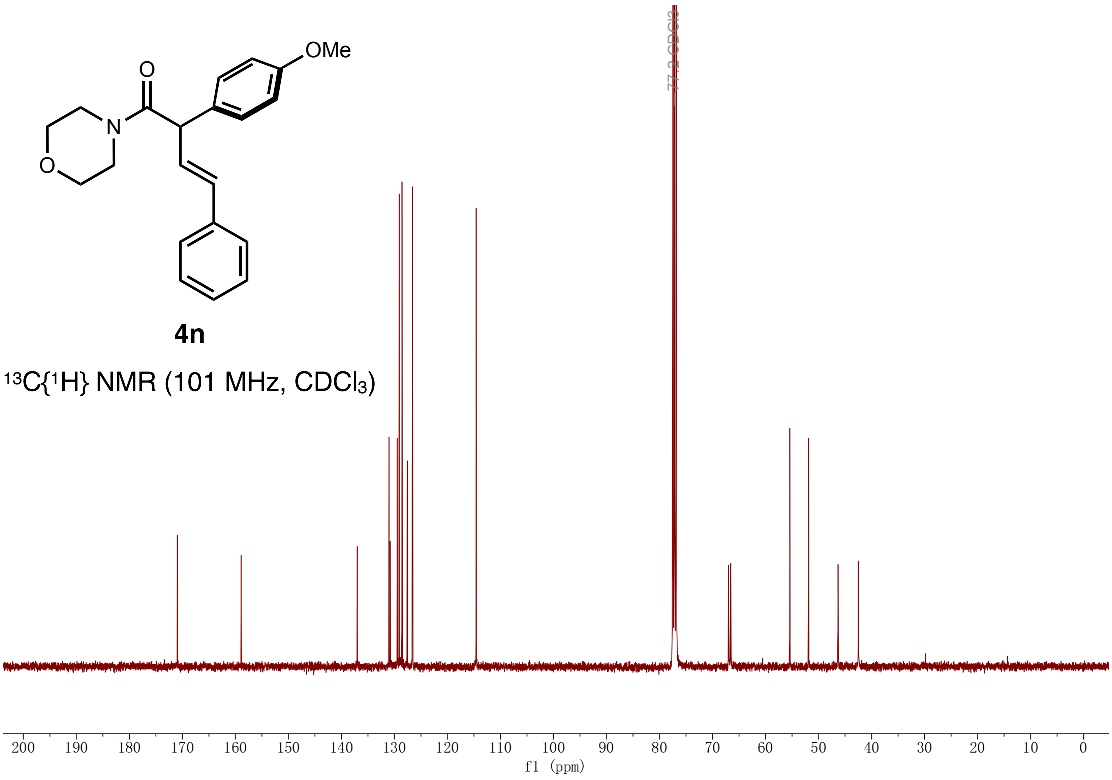
77.2 CDCl3
OMe
O
N
O
4n
^{13}C{^{1}H} NMR (101 MHz, CDCl$_3$)
200 190 180 170 160 150 140 130 120 110 100 90 80 70 60 50 40 30 20 10 0
f1 (ppm)

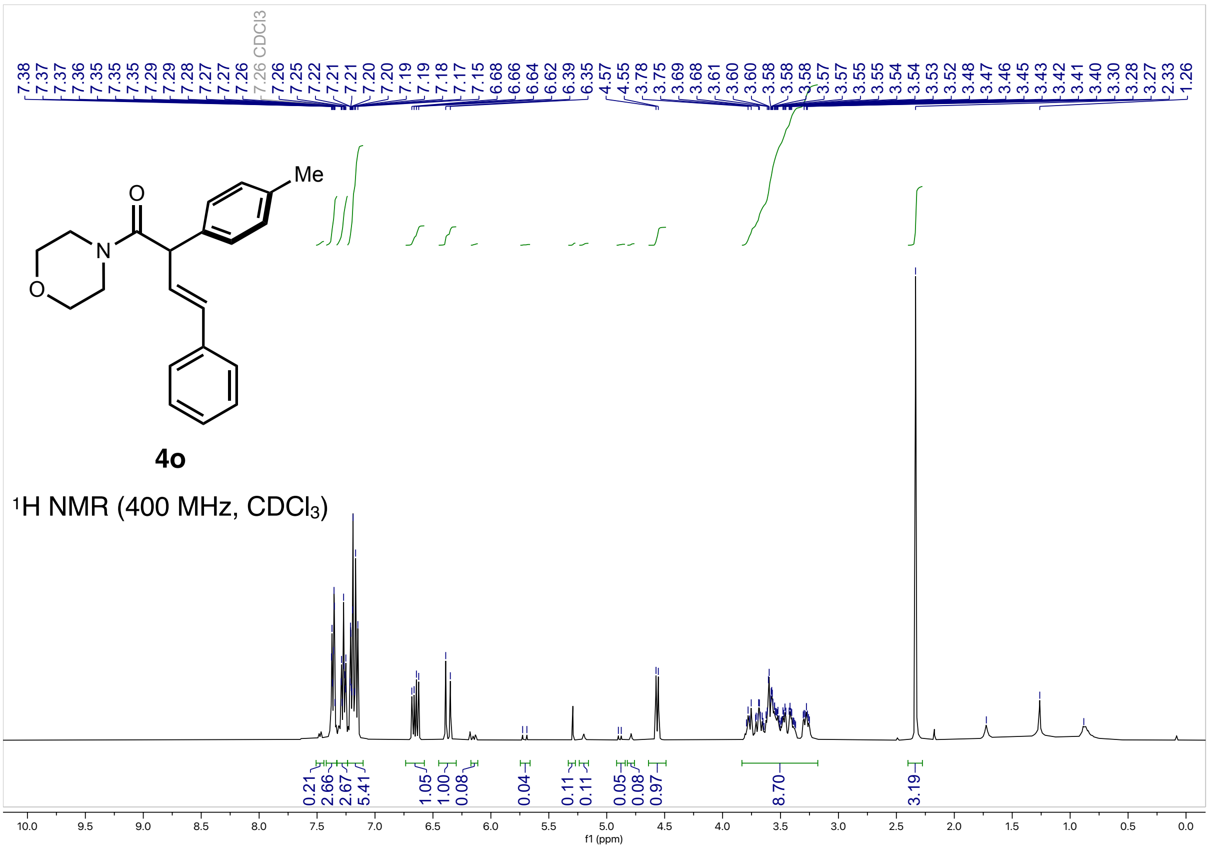

Me
O
N
O
4o
¹H NMR (400 MHz, CDCl₃)
7.26 CDCl3
f1 (ppm)

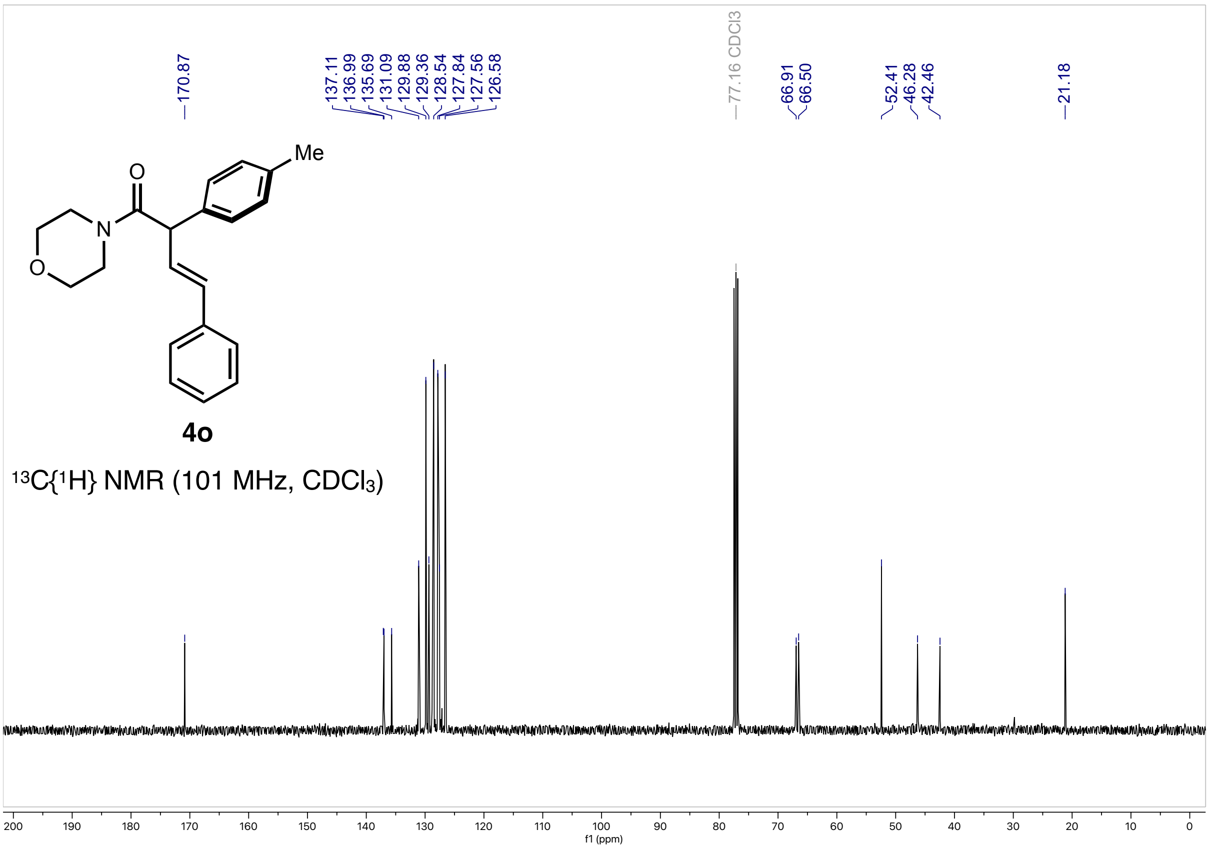

Me
O
N
O
4o
¹³C{¹H} NMR (101 MHz, CDCl₃)
170.87
137.11
136.99
135.69
131.09
129.88
129.36
128.54
127.84
127.56
126.58
77.16 CDCl3
66.91
66.50
52.41
46.28
42.46
21.18
f1 (ppm)

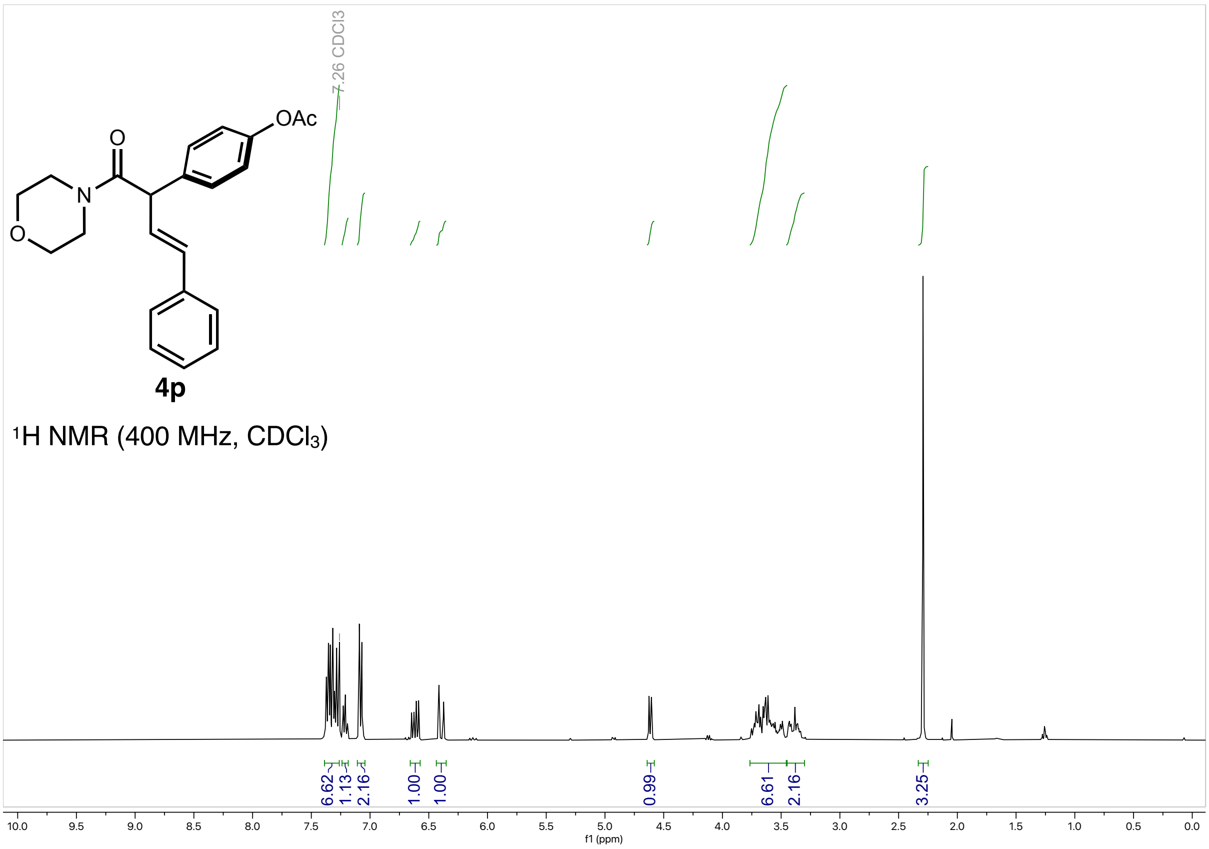

4p

^1H NMR (400 MHz, $CDCl_3$)

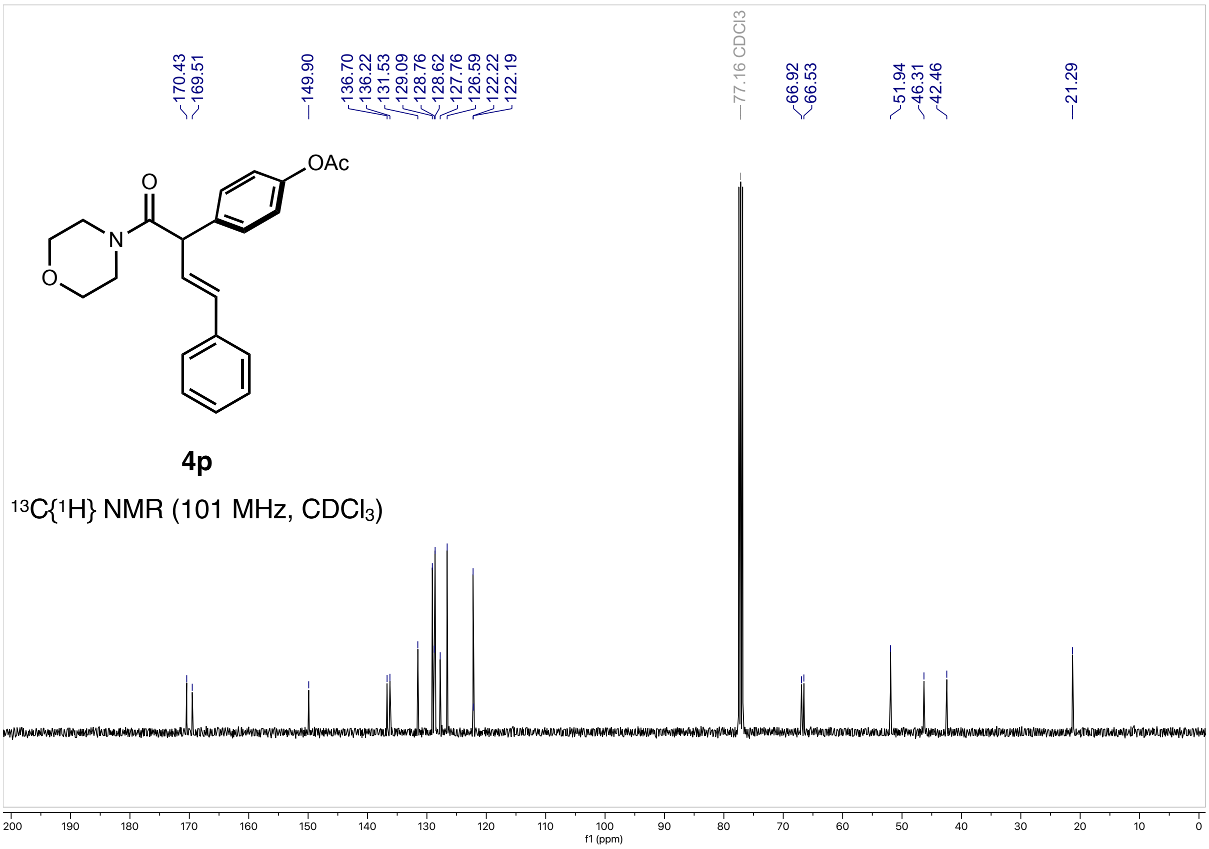

4p

$^{13}C\{^1H\}$ NMR (101 MHz, $CDCl_3$)

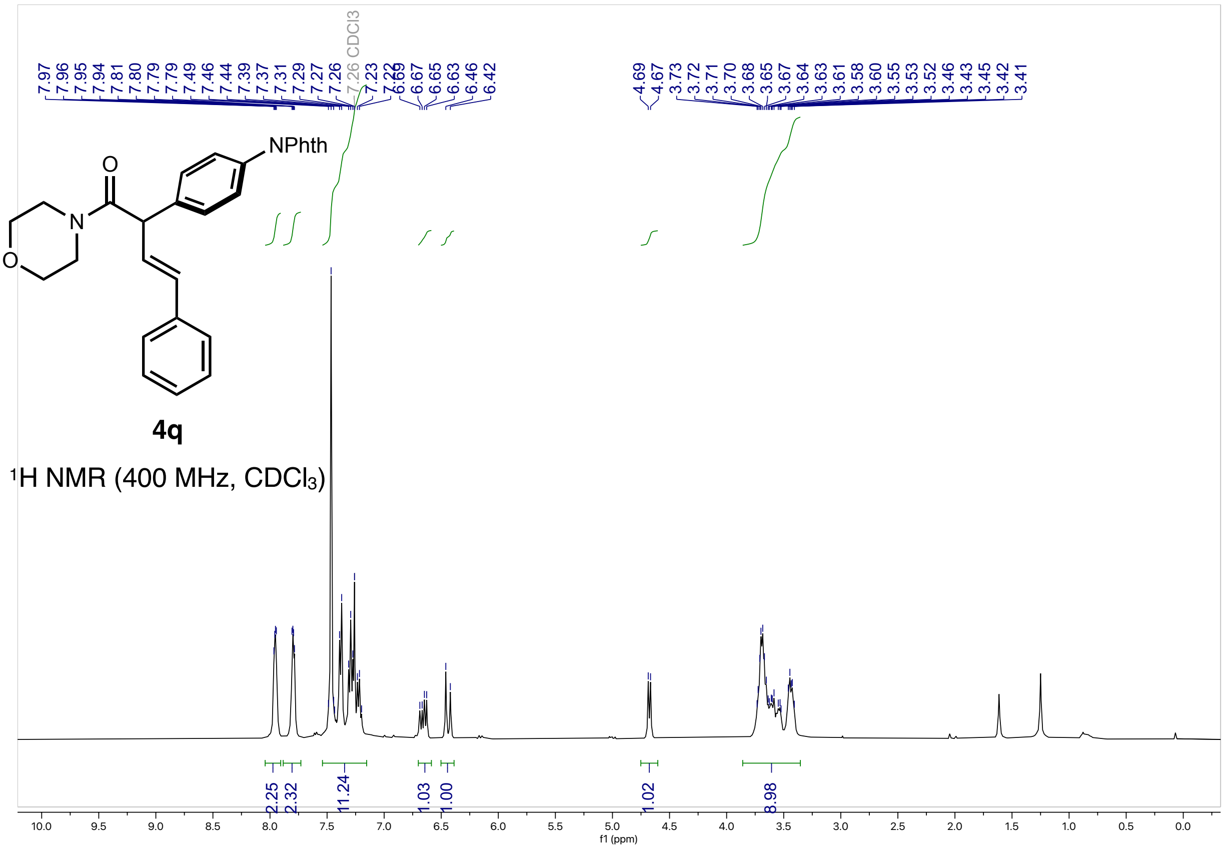

4q

^{1}H NMR (400 MHz, $CDCl_3$)

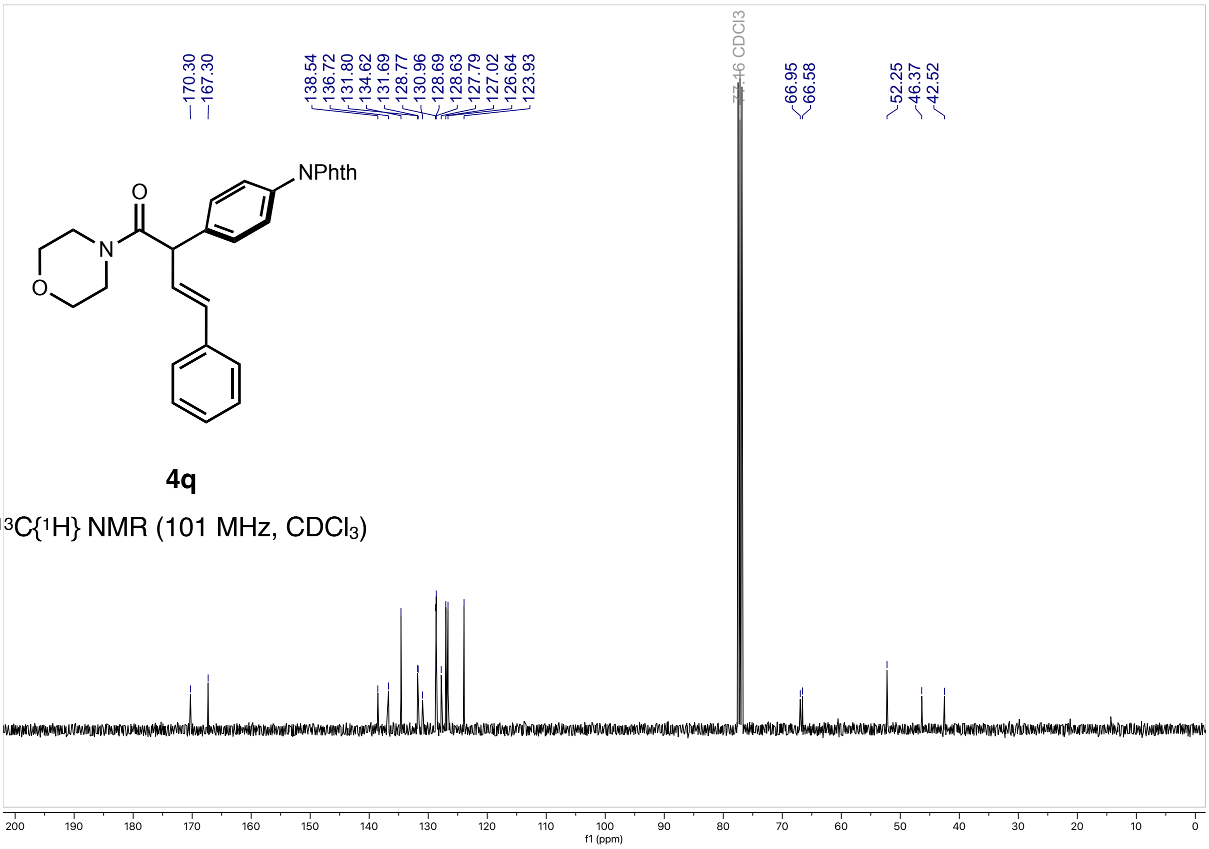

4q

^{13}C{^{1}H} NMR (101 MHz, $CDCl_3$)

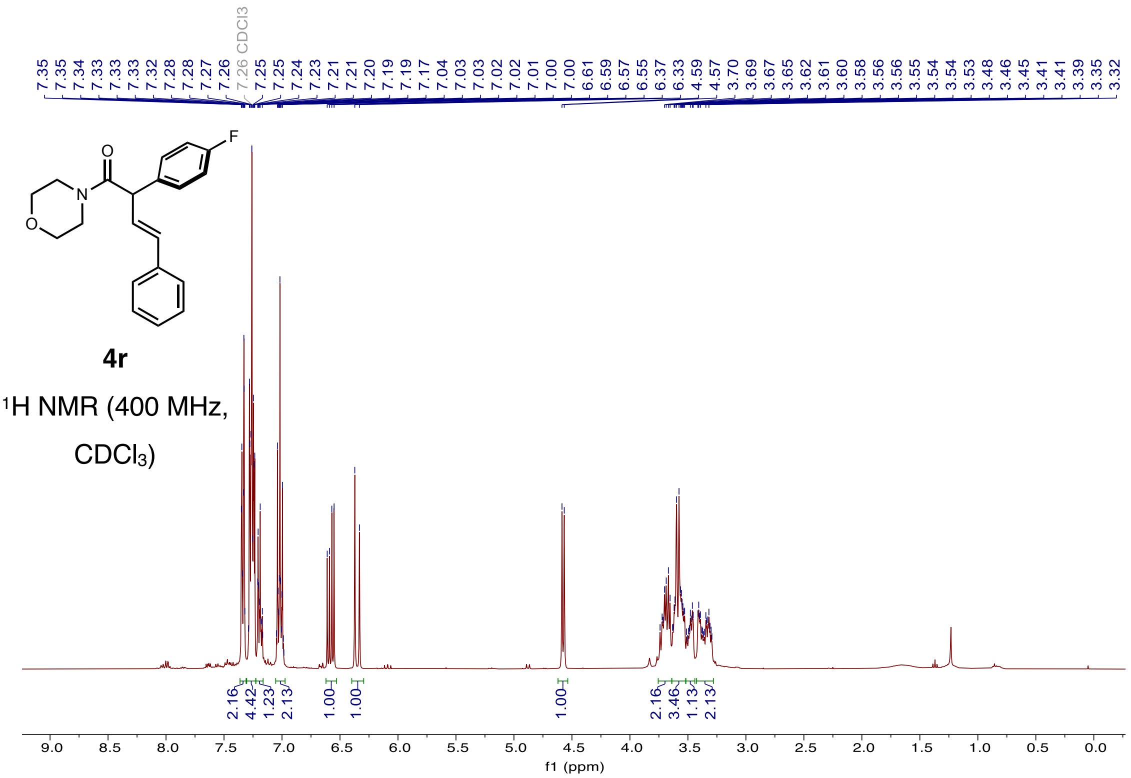
7.35 7.35 7.34 7.33 7.33 7.33 7.32 7.28 7.28 7.27 7.26 7.26 CDCl3 7.25 7.25 7.24 7.23 7.21 7.21 7.20 7.19 7.19 7.17 7.04 7.03 7.03 7.02 7.02 7.01 7.00 7.00 6.61 6.59 6.57 6.55 6.37 6.33 4.59 4.57 3.70 3.69 3.67 3.65 3.62 3.61 3.60 3.58 3.56 3.56 3.55 3.54 3.54 3.53 3.48 3.46 3.45 3.41 3.41 3.39 3.35 3.32
O
N
O
F
4r
¹H NMR (400 MHz, CDCl3)
2.16 4.42 1.23 2.13 1.00 1.00 1.00 2.16 3.46 1.13 2.13
9.0 8.5 8.0 7.5 7.0 6.5 6.0 5.5 5.0 4.5 4.0 3.5 3.0 2.5 2.0 1.5 1.0 0.5 0.0
f1 (ppm)

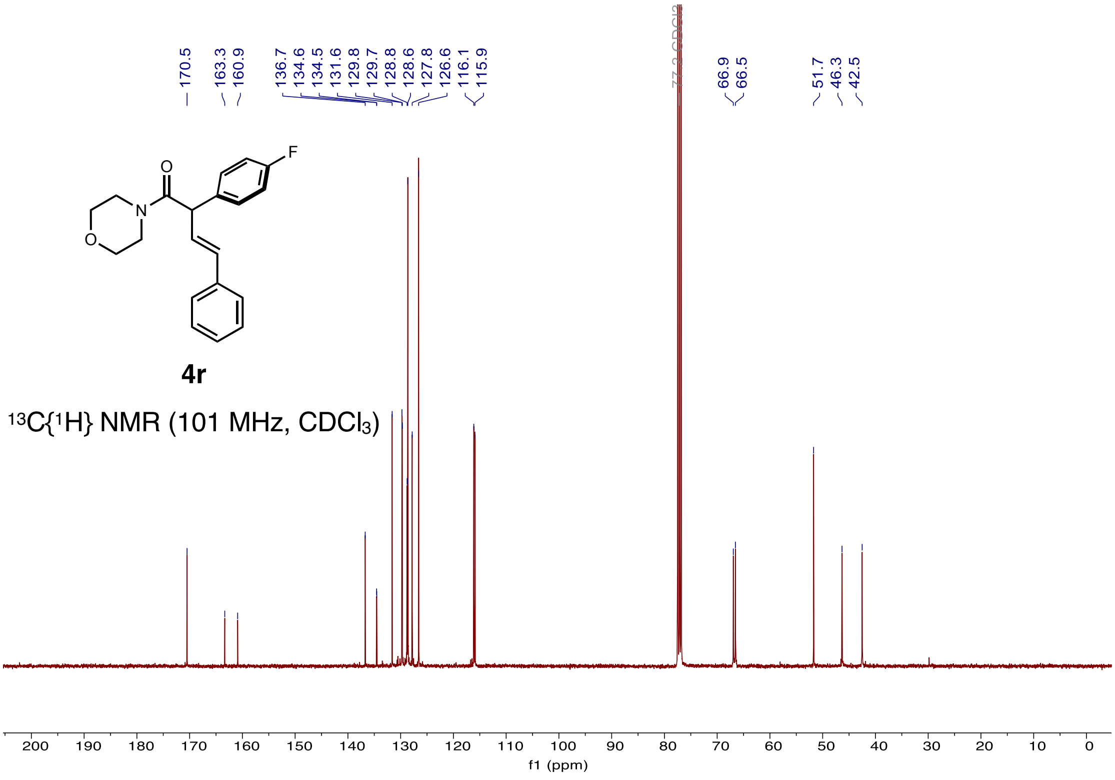
170.5 163.3 160.9 136.7 134.6 134.5 131.6 129.8 129.7 128.8 128.6 127.8 126.6 116.1 115.9 66.9 66.5 51.7 46.3 42.5
O
N
O
F
4r
¹³C{¹H} NMR (101 MHz, CDCl3)
200 190 180 170 160 150 140 130 120 110 100 90 80 70 60 50 40 30 20 10 0
f1 (ppm)

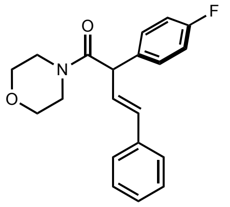

4r

^{19}F NMR (376 MHz, $CDCl_3$)

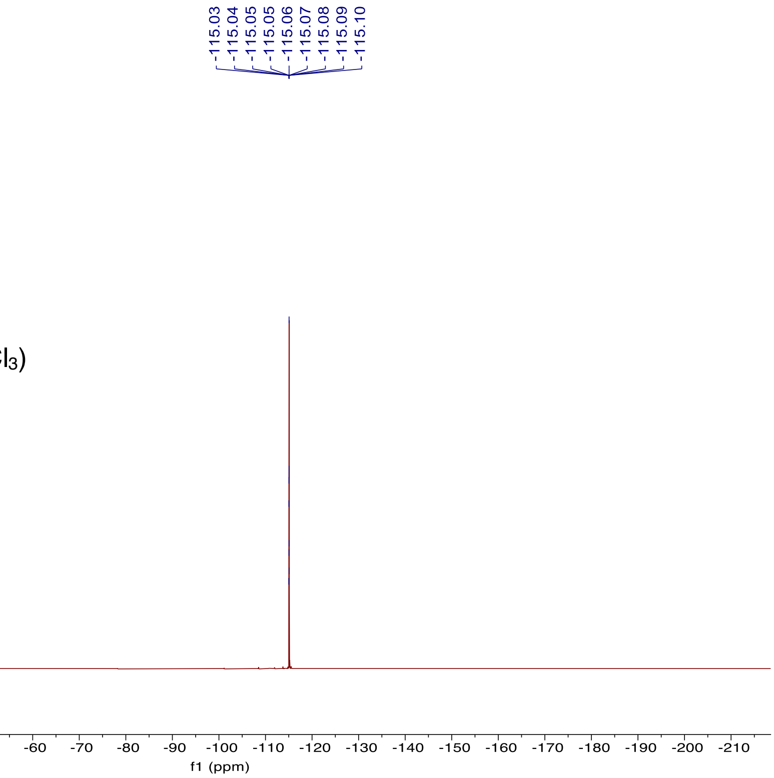

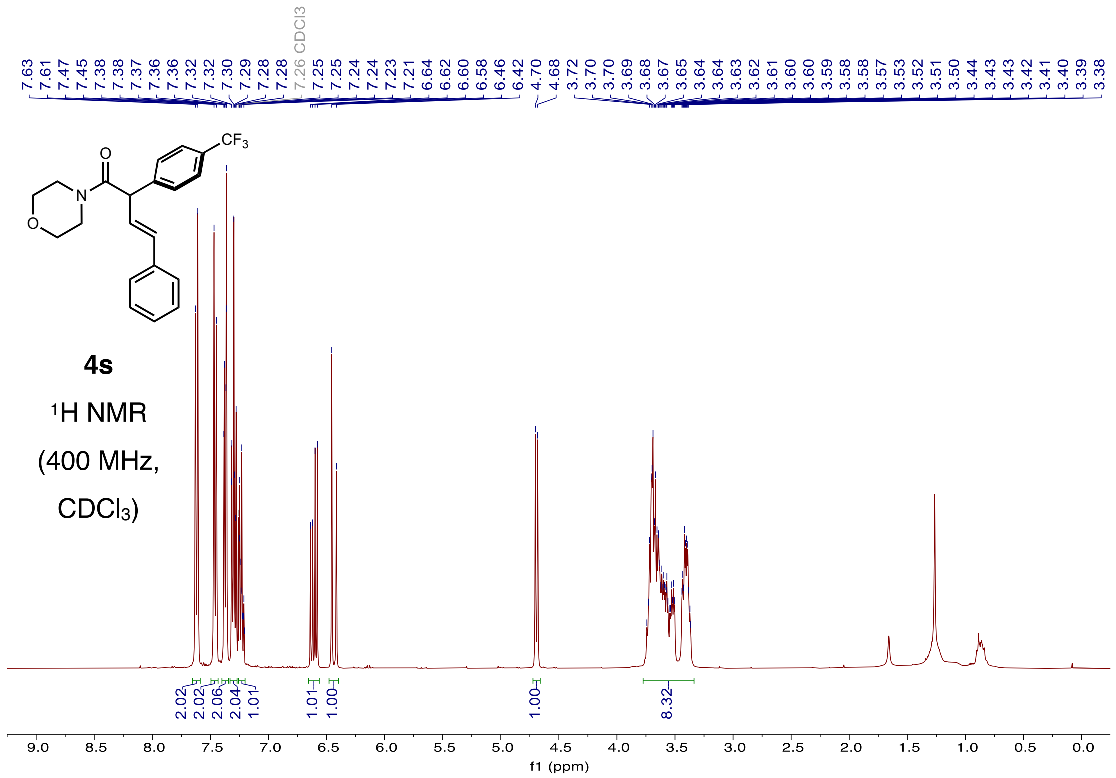

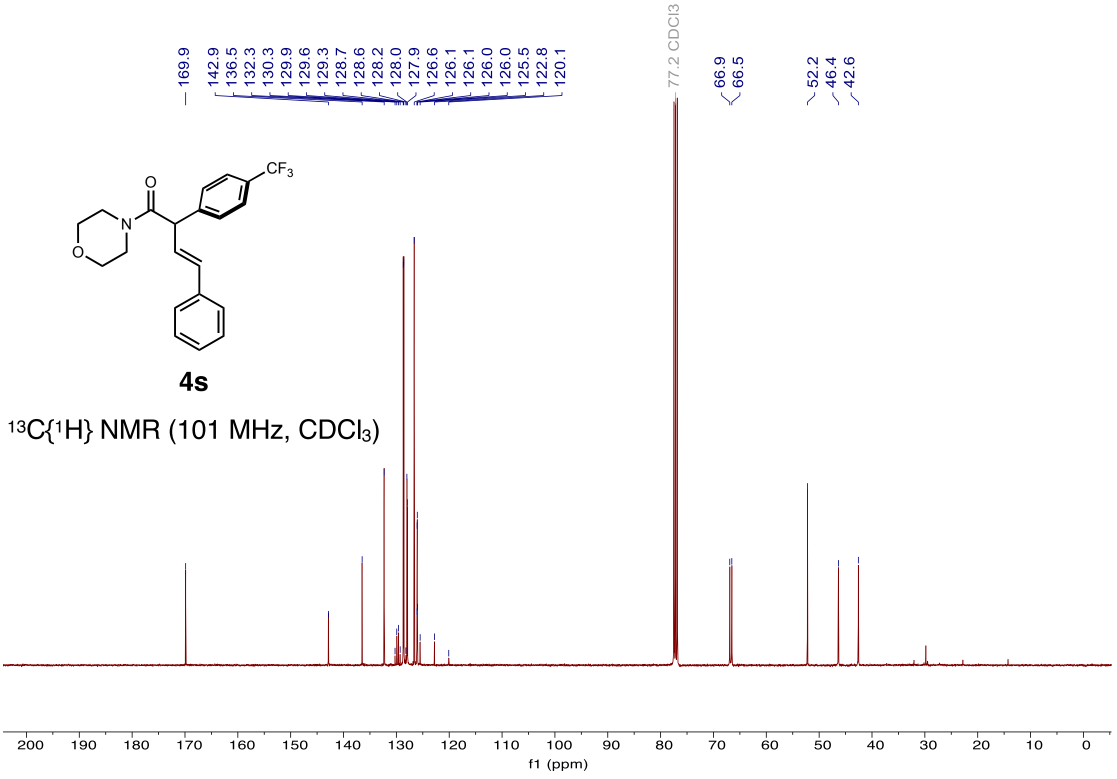

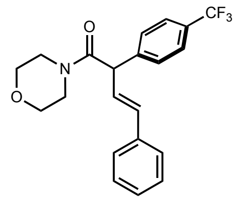

4s

^{19}F NMR (376 MHz, $CDCl_3$)

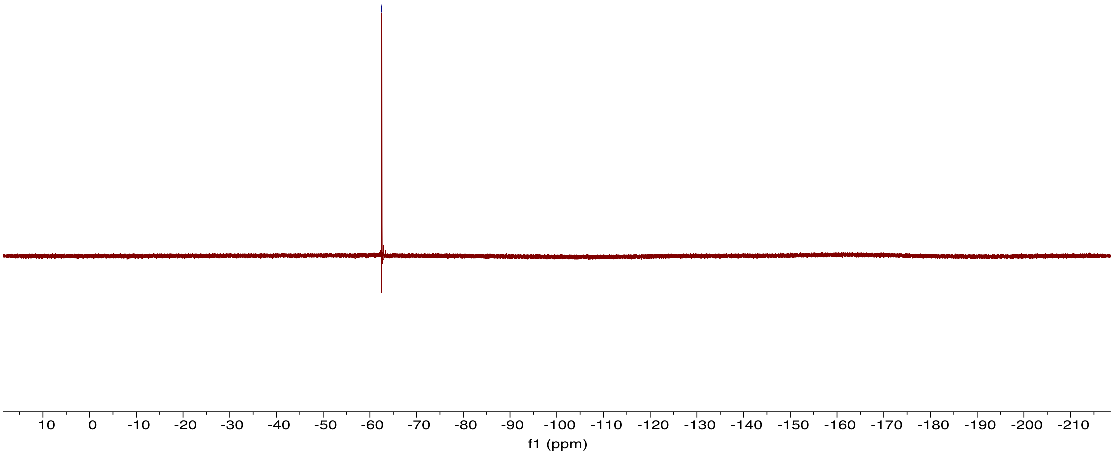

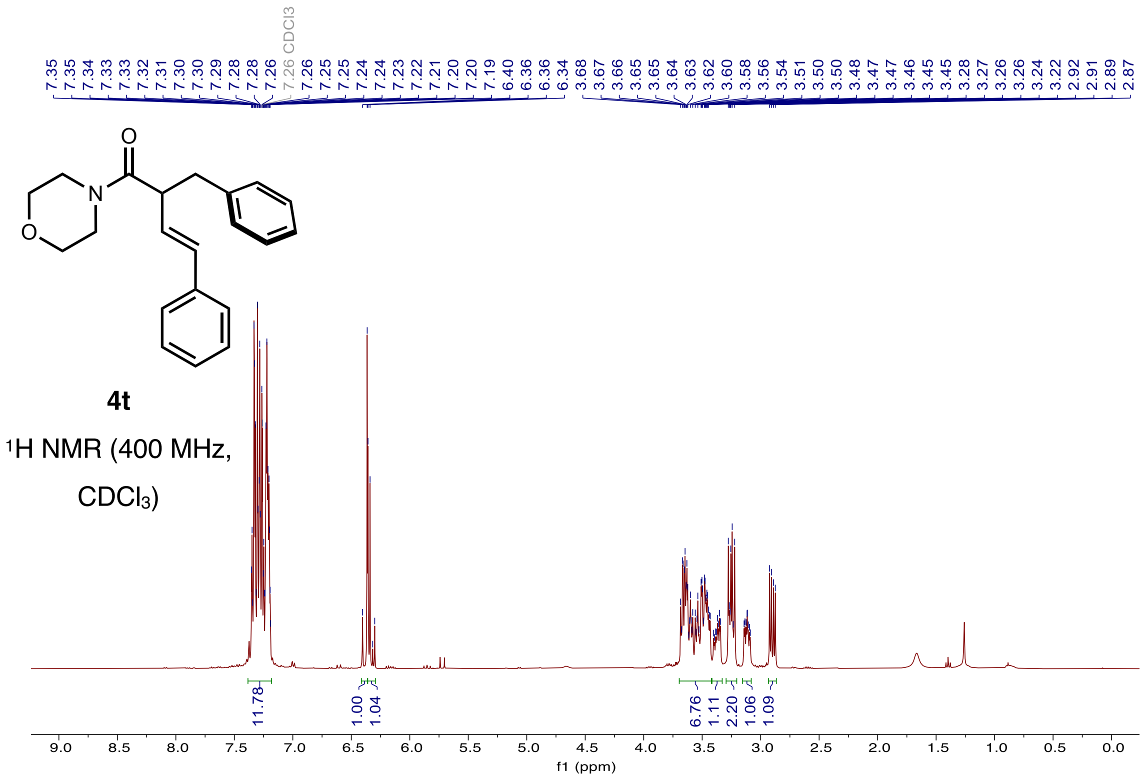
7.35 7.35 7.34 7.33 7.33 7.32 7.31 7.30 7.30 7.29 7.28 7.28 7.26 7.26 CDCl3 7.26 7.25 7.25 7.24 7.24 7.23 7.22 7.21 7.21 7.20 7.20 7.19 6.40 6.36 6.36 6.34 3.68 3.67 3.66 3.65 3.65 3.64 3.63 3.62 3.60 3.58 3.56 3.54 3.51 3.50 3.50 3.48 3.47 3.47 3.46 3.45 3.45 3.28 3.27 3.26 3.26 3.24 3.22 2.92 2.91 2.89 2.87
O
N
O
4t
¹H NMR (400 MHz, CDCl3)
11.78
1.00 1.04
6.76 1.11 2.20 1.06 1.09
9.0 8.5 8.0 7.5 7.0 6.5 6.0 5.5 5.0 4.5 4.0 3.5 3.0 2.5 2.0 1.5 1.0 0.5 0.0
f1 (ppm)

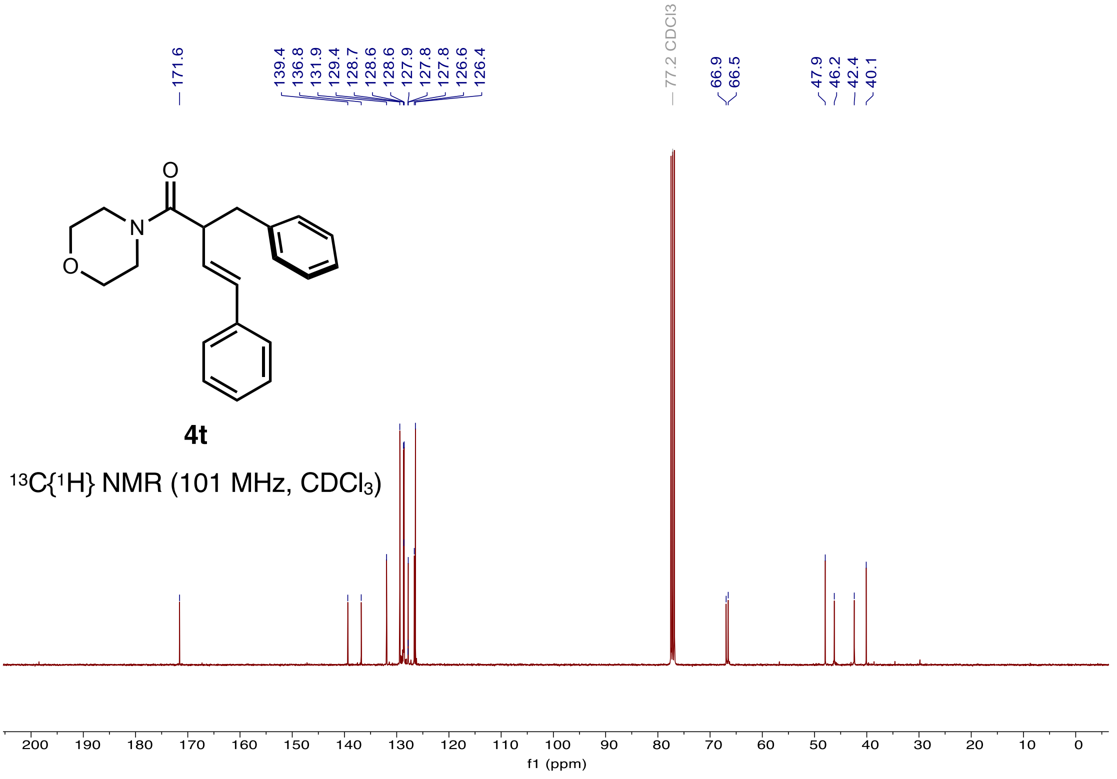
171.6
139.4 136.8 131.9 129.4 128.7 128.6 128.6 127.9 127.8 126.6 126.4
77.2 CDCl3
66.9 66.5
47.9 46.2 42.4 40.1
O
N
O
4t
¹³C{¹H} NMR (101 MHz, CDCl3)
200 190 180 170 160 150 140 130 120 110 100 90 80 70 60 50 40 30 20 10 0
f1 (ppm)

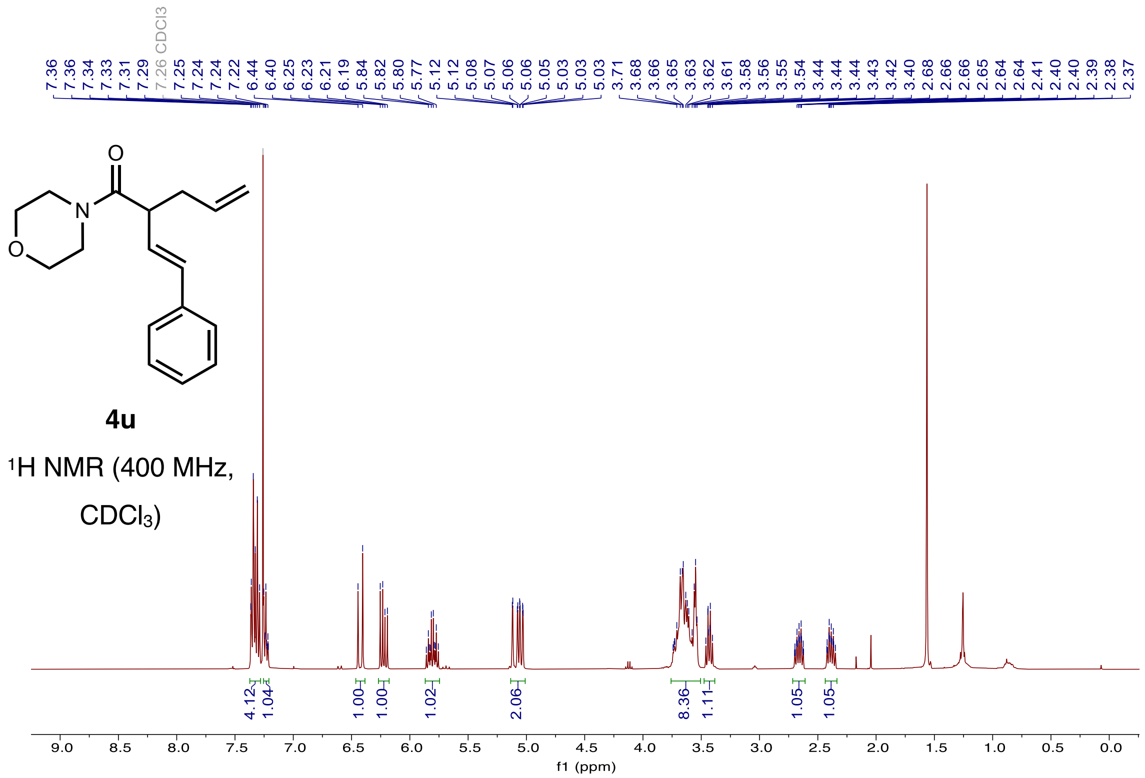

7.36 7.36 7.34 7.33 7.31 7.29 7.26 CDCl3 7.25 7.24 7.24 7.22 6.44 6.40 6.25 6.23 6.21 6.19 5.84 5.82 5.80 5.77 5.12 5.12 5.08 5.07 5.06 5.06 5.05 5.03 5.03 5.03 3.71 3.68 3.66 3.65 3.63 3.62 3.61 3.58 3.56 3.55 3.54 3.44 3.44 3.44 3.43 3.42 3.40 2.68 2.66 2.66 2.65 2.64 2.64 2.41 2.40 2.40 2.39 2.38 2.37
O
N
O
4u
¹H NMR (400 MHz, CDCl₃)
4.12 1.04 1.00 1.00 1.02 2.06 8.36 1.11 1.05 1.05
9.0 8.5 8.0 7.5 7.0 6.5 6.0 5.5 5.0 4.5 4.0 3.5 3.0 2.5 2.0 1.5 1.0 0.5 0.0
f1 (ppm)

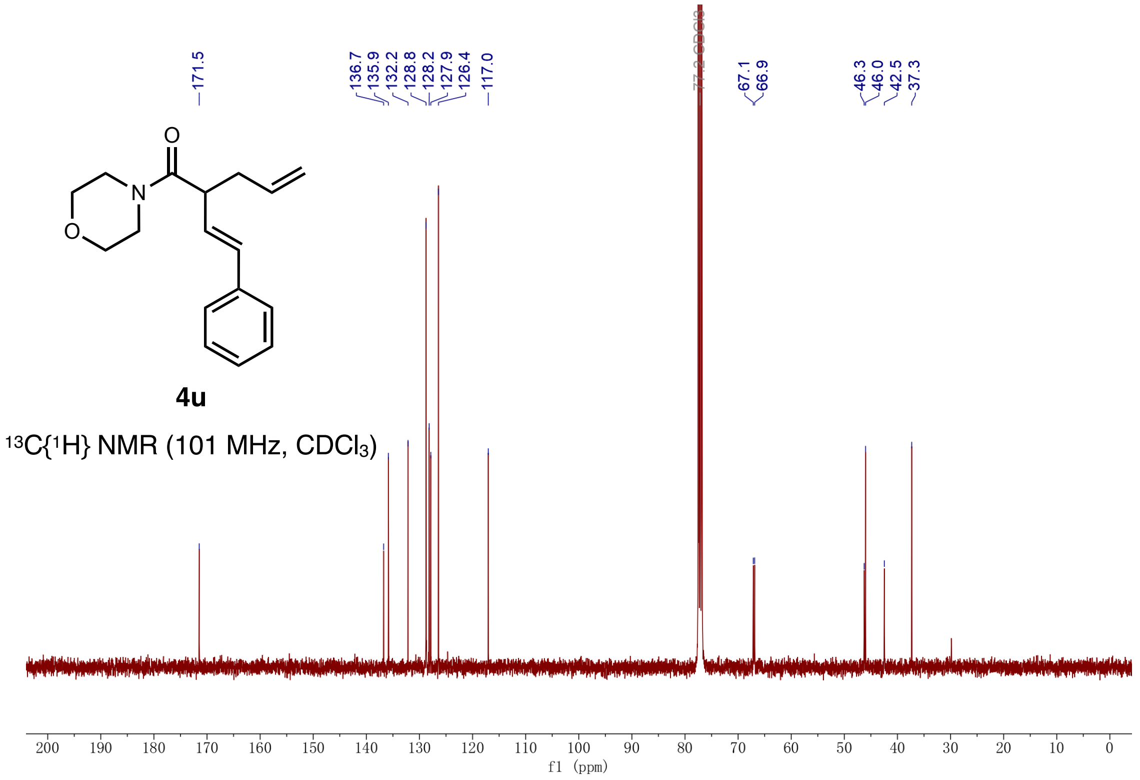

171.5
136.7 135.9 132.2 128.8 128.2 127.9 126.4 117.0
77.0 CDCl3
67.1 66.9
46.3 46.0 42.5 37.3
O
N
O
4u
¹³C{¹H} NMR (101 MHz, CDCl₃)
200 190 180 170 160 150 140 130 120 110 100 90 80 70 60 50 40 30 20 10 0
f1 (ppm)

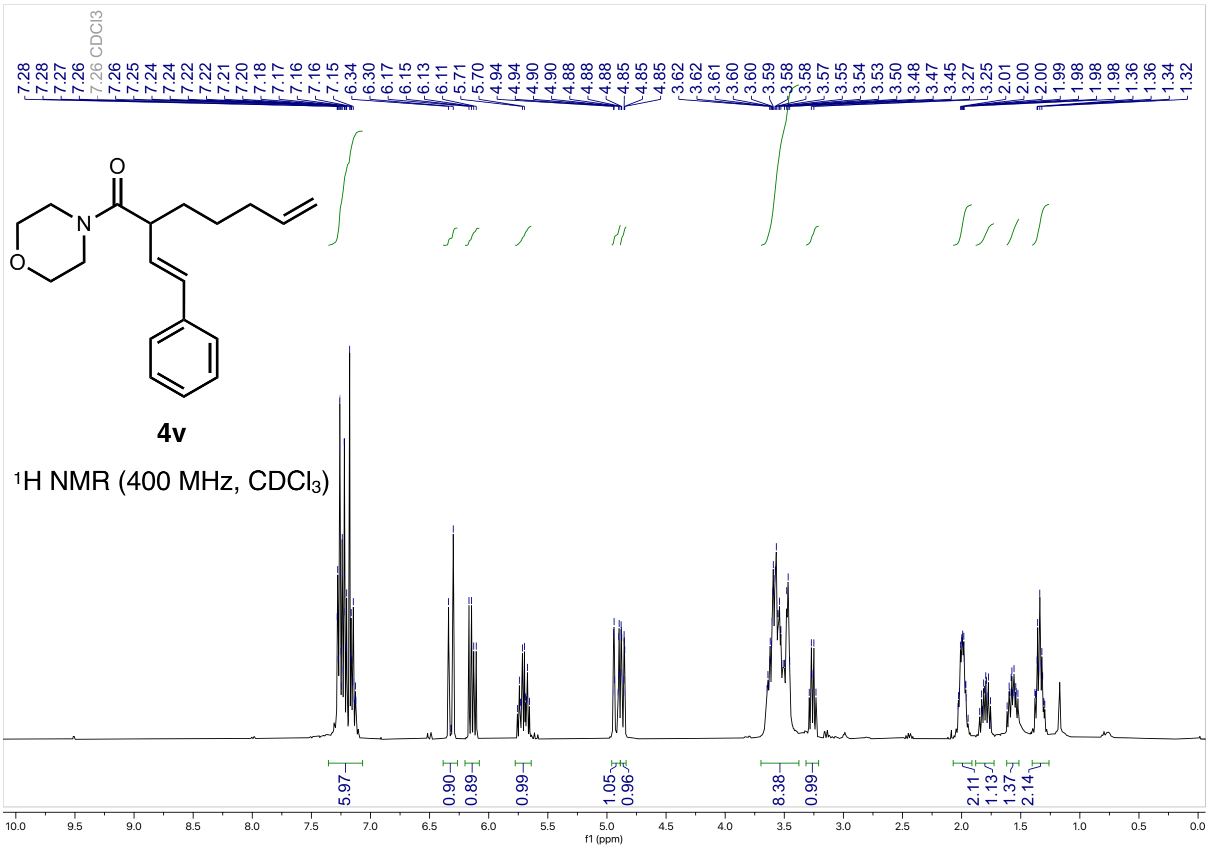
4v
^{1}H NMR (400 MHz, $CDCl_3$)
f1 (ppm)

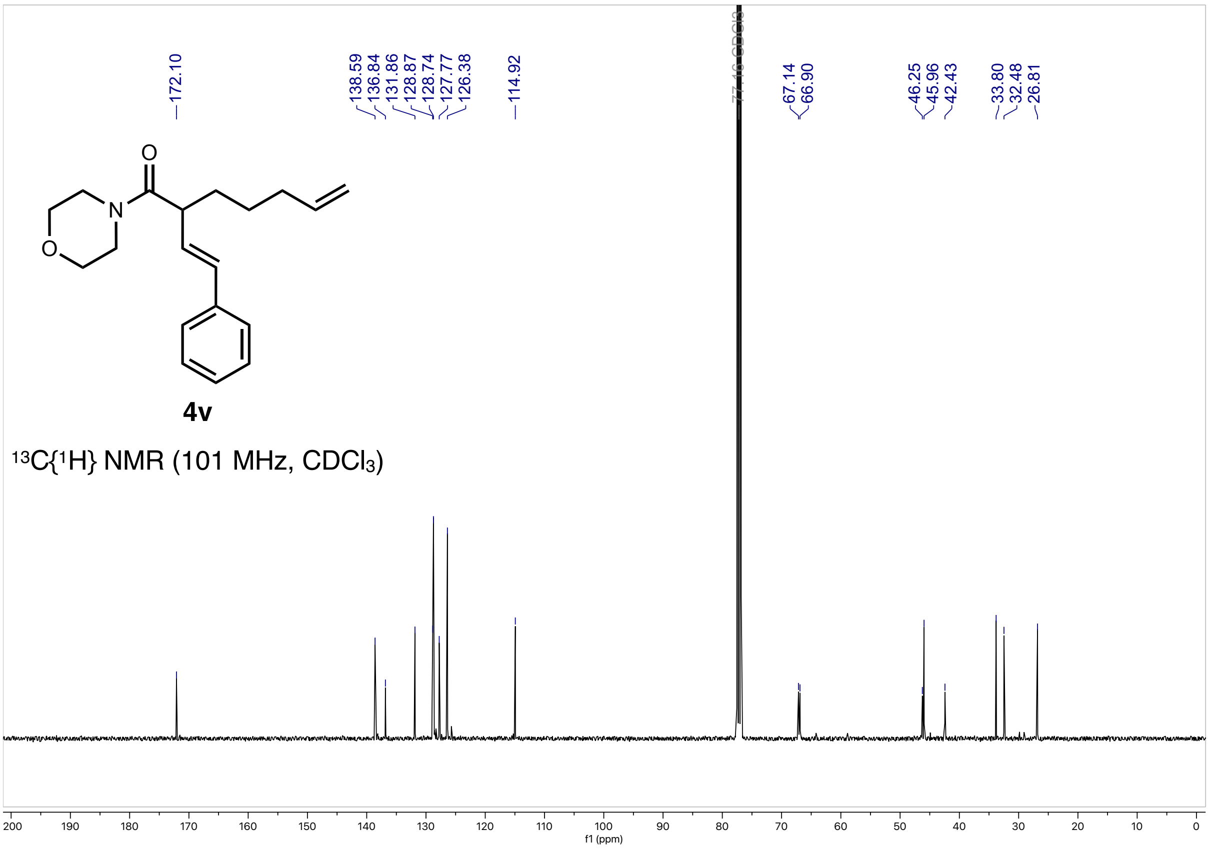
4v
$^{13}C\{^{1}H\}$ NMR (101 MHz, $CDCl_3$)
f1 (ppm)

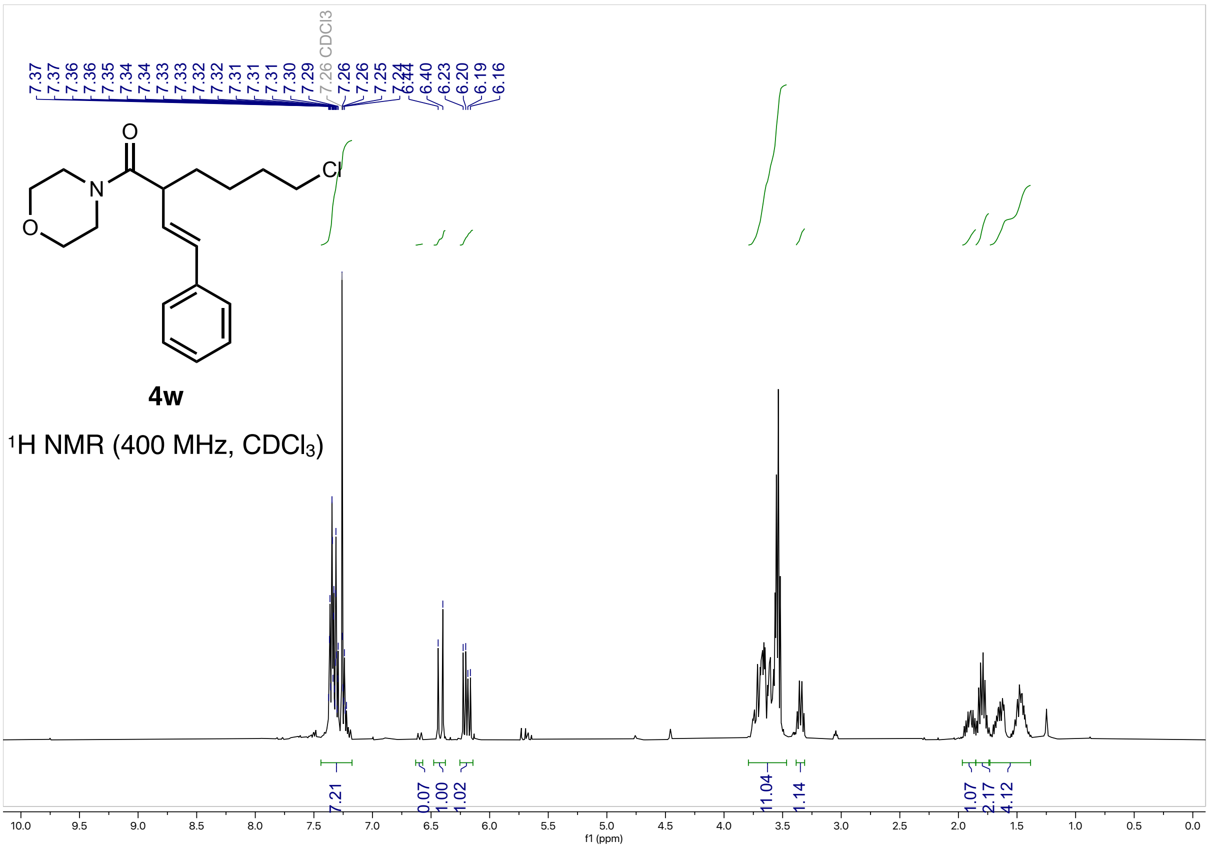

4w
^{1}H NMR (400 MHz, CDCl$_3$)
7.37 7.37 7.36 7.36 7.35 7.34 7.34 7.33 7.33 7.32 7.32 7.31 7.31 7.31 7.30 7.29 7.26 CDCl3 7.26 7.26 7.25 7.24 6.44 6.40 6.23 6.20 6.19 6.16
7.21 0.07 1.00 1.02 11.04 1.14 1.07 2.17 4.12
f1 (ppm)

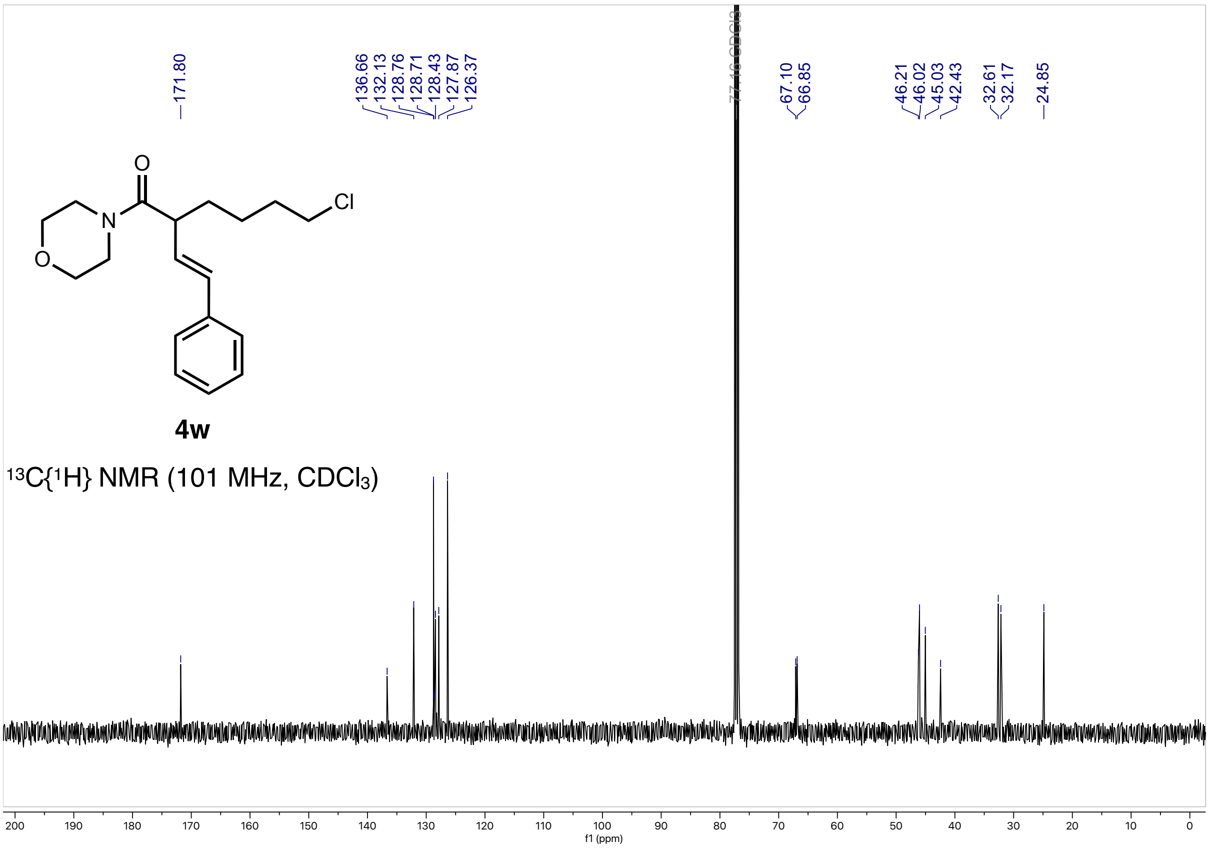

4w
^{13}C{^{1}H} NMR (101 MHz, CDCl$_3$)
171.80 136.66 132.13 128.76 128.71 128.43 127.87 126.37 77.16 CDCl3 67.10 66.85 46.21 46.02 45.03 42.43 32.61 32.17 24.85
f1 (ppm)

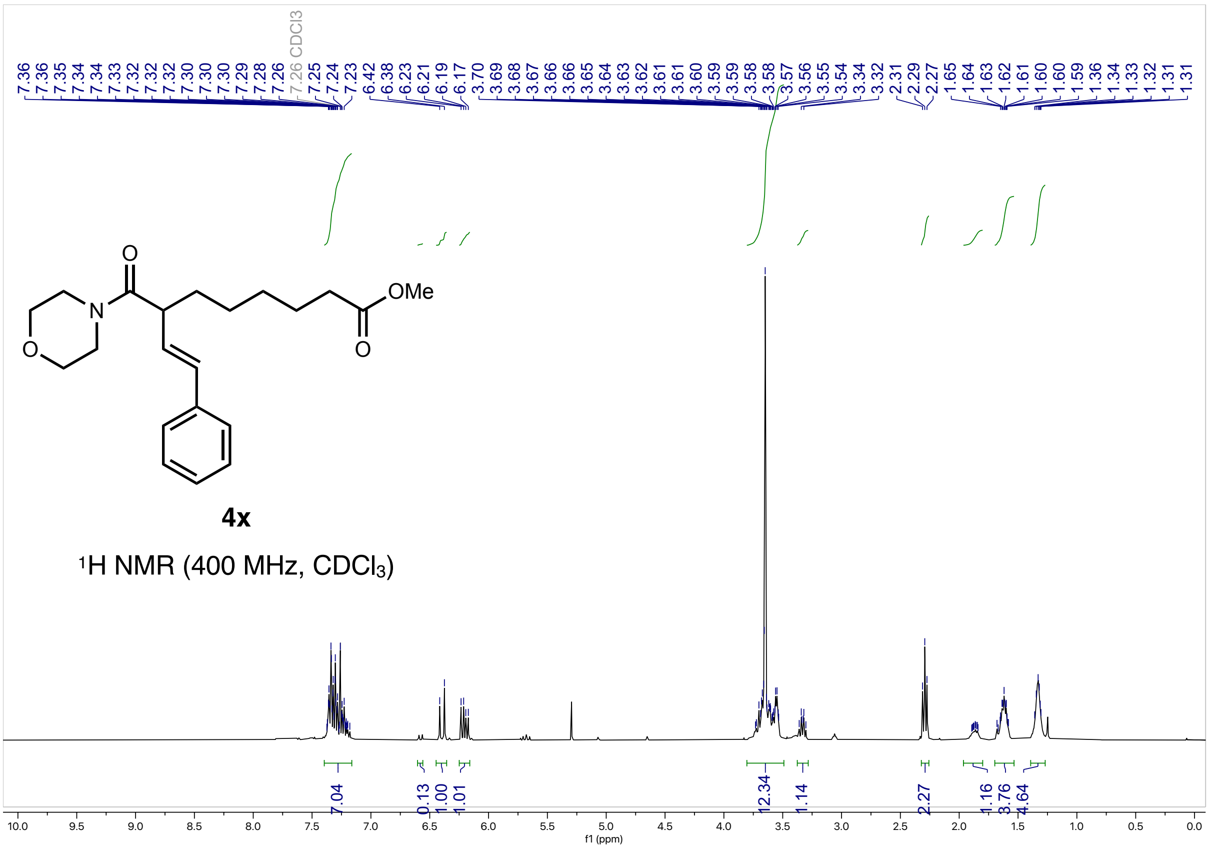

7.36 7.36 7.35 7.34 7.34 7.33 7.32 7.32 7.32 7.30 7.30 7.30 7.29 7.28 7.26 7.26 CDCl3 7.25 7.24 7.23 6.42 6.38 6.23 6.21 6.19 6.17 3.70 3.69 3.68 3.67 3.66 3.66 3.65 3.64 3.63 3.62 3.61 3.61 3.60 3.59 3.59 3.58 3.58 3.57 3.56 3.55 3.54 3.34 3.32 2.31 2.29 2.27 1.65 1.64 1.63 1.62 1.61 1.60 1.60 1.59 1.36 1.34 1.33 1.32 1.31 1.31
OMe
O
N
4x
¹H NMR (400 MHz, CDCl₃)
7.04 0.13 1.00 1.01 12.34 1.14 2.27 1.16 3.76 4.64
f1 (ppm)

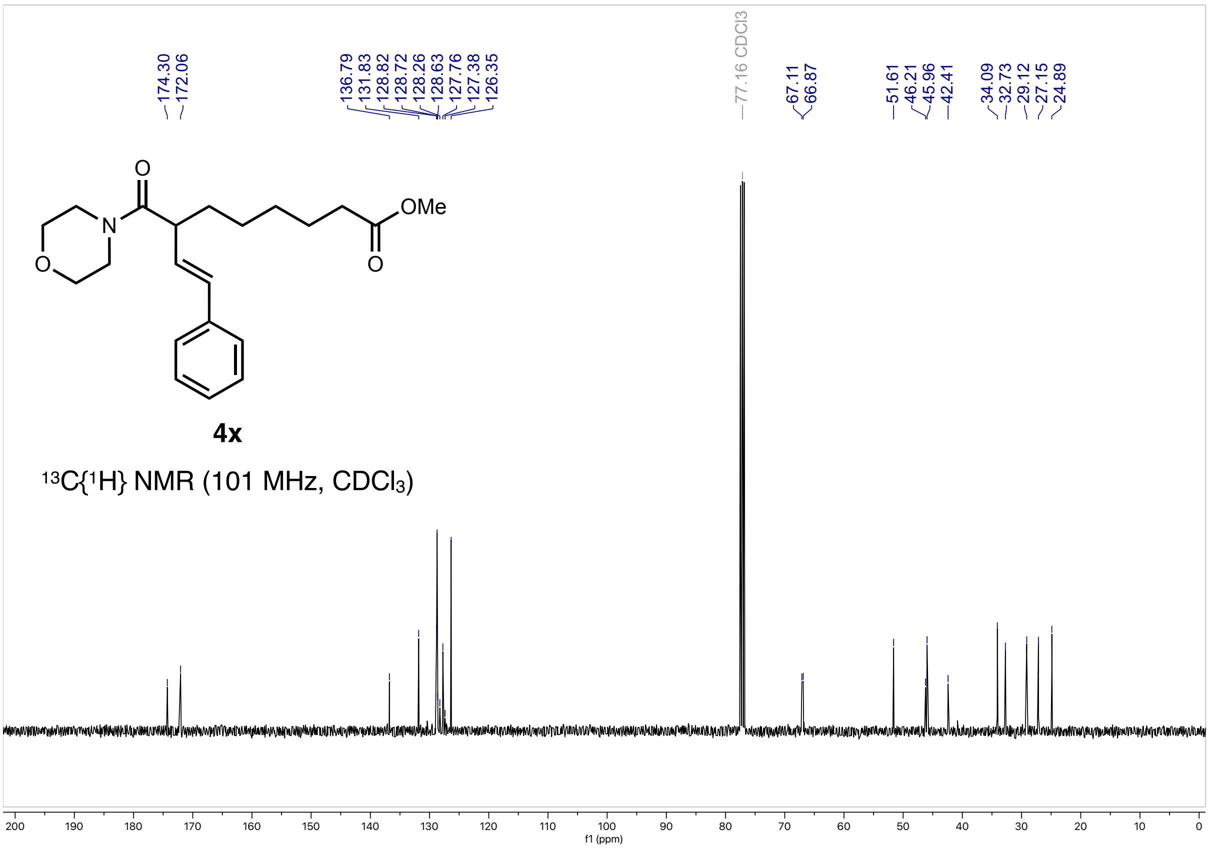

174.30 172.06 136.79 131.83 128.82 128.72 128.26 128.63 127.76 127.38 126.35 77.16 CDCl3 67.11 66.87 51.61 46.21 45.96 42.41 34.09 32.73 29.12 27.15 24.89
OMe
O
N
4x
¹³C{¹H} NMR (101 MHz, CDCl₃)
f1 (ppm)

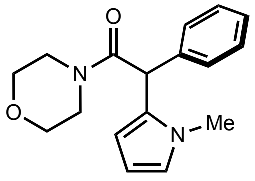

5a

^{1}H NMR (400 MHz, $CDCl_3$)

5.7:1 mixture of regioisomers

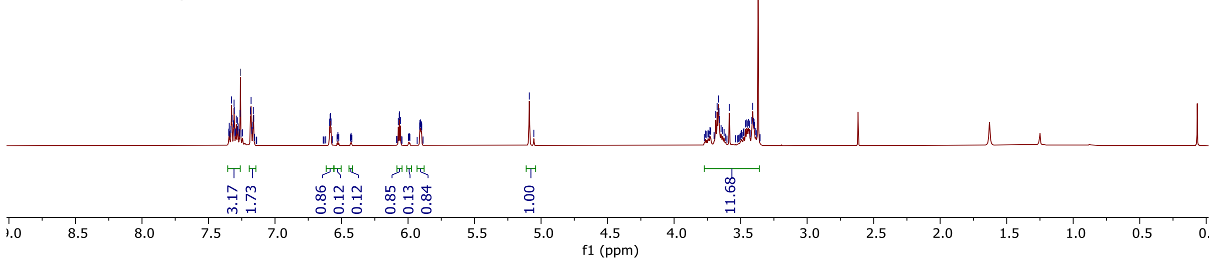

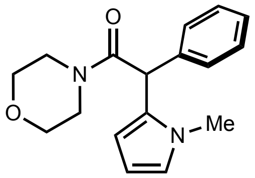

5a

$^{13}C\{^1H\}$ NMR (101 MHz, $CDCl_3$)

5.7:1 mixture of regioisomers

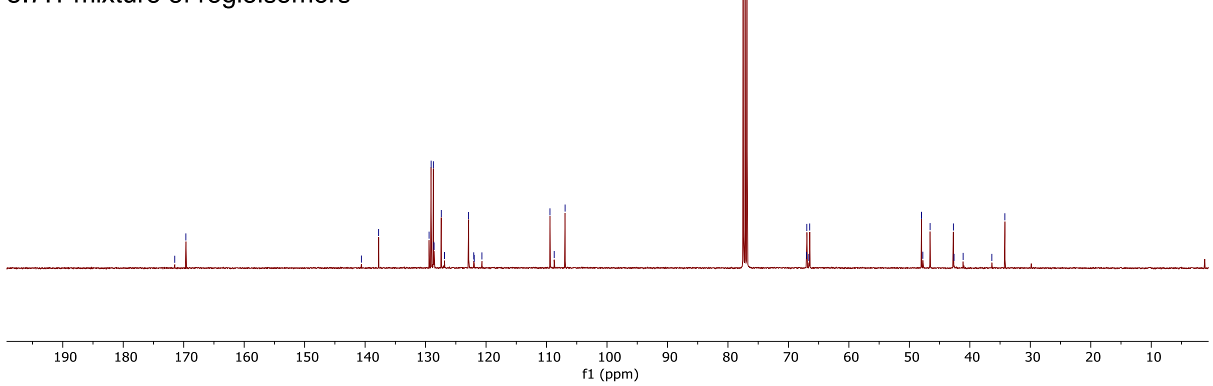

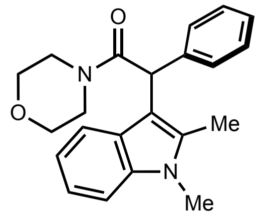

5b

^{1}H NMR (400 MHz, $CDCl_3$)

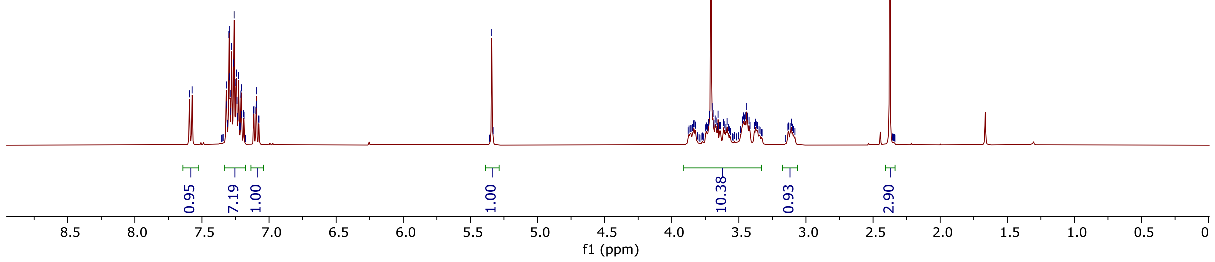

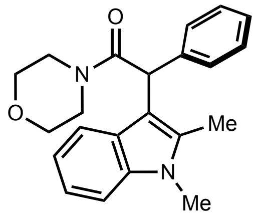

5b

^{13}C{^{1}H} NMR (101 MHz, $CDCl_3$)

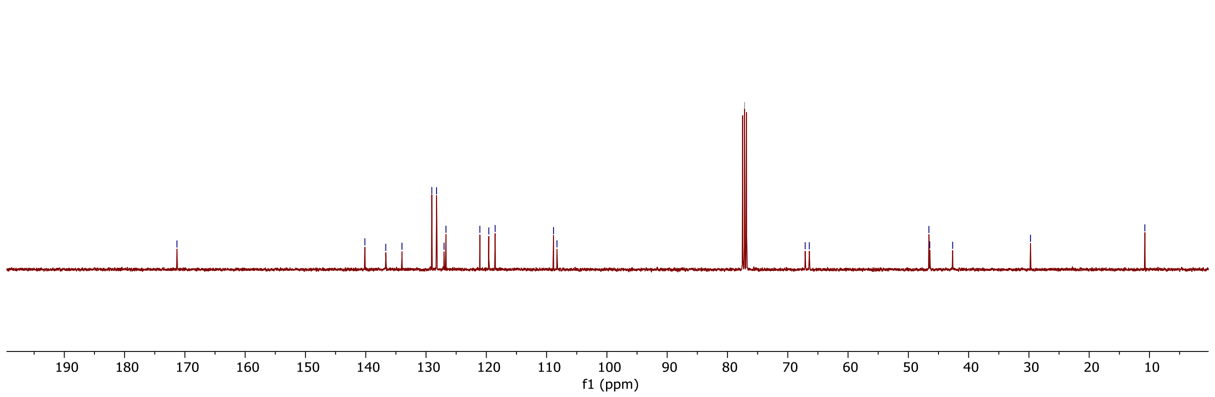

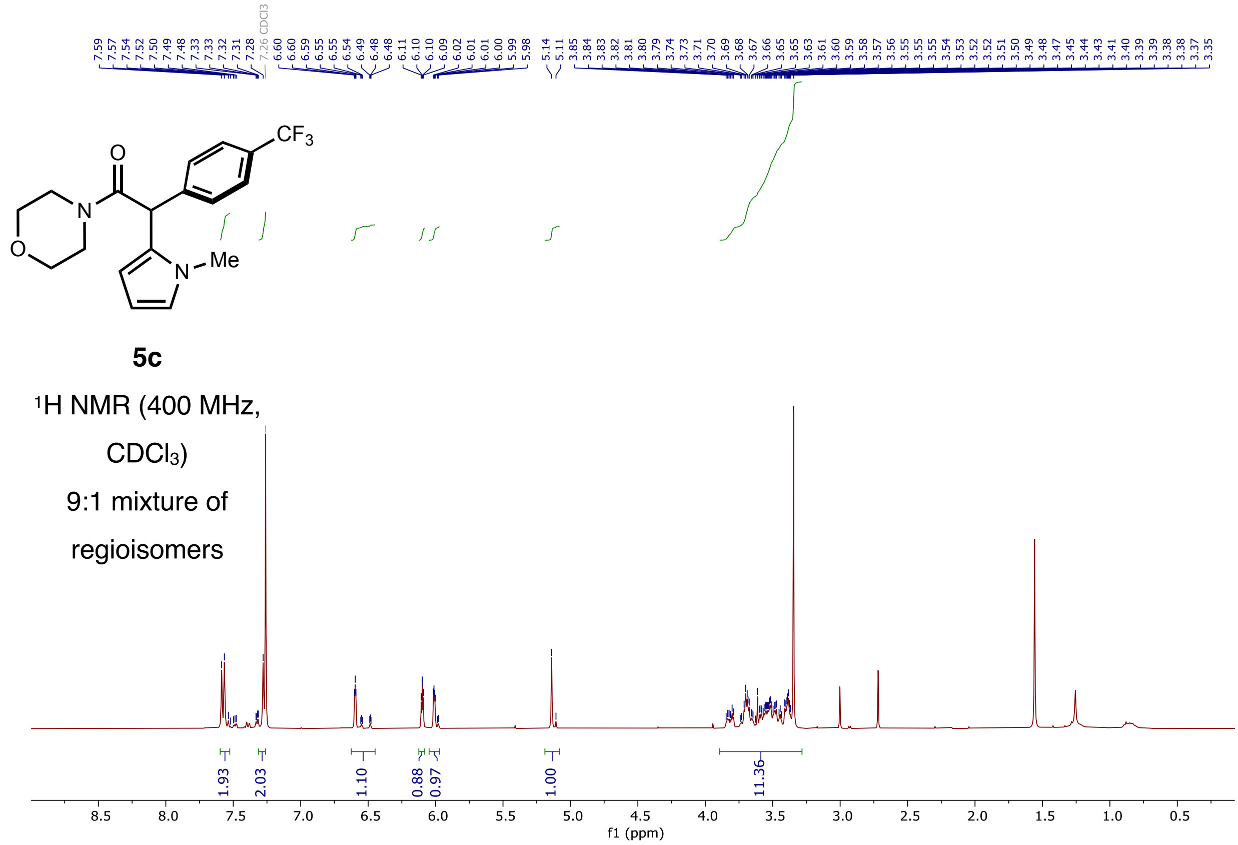

5c

^{1}H NMR (400 MHz, $CDCl_3$)

9:1 mixture of regioisomers

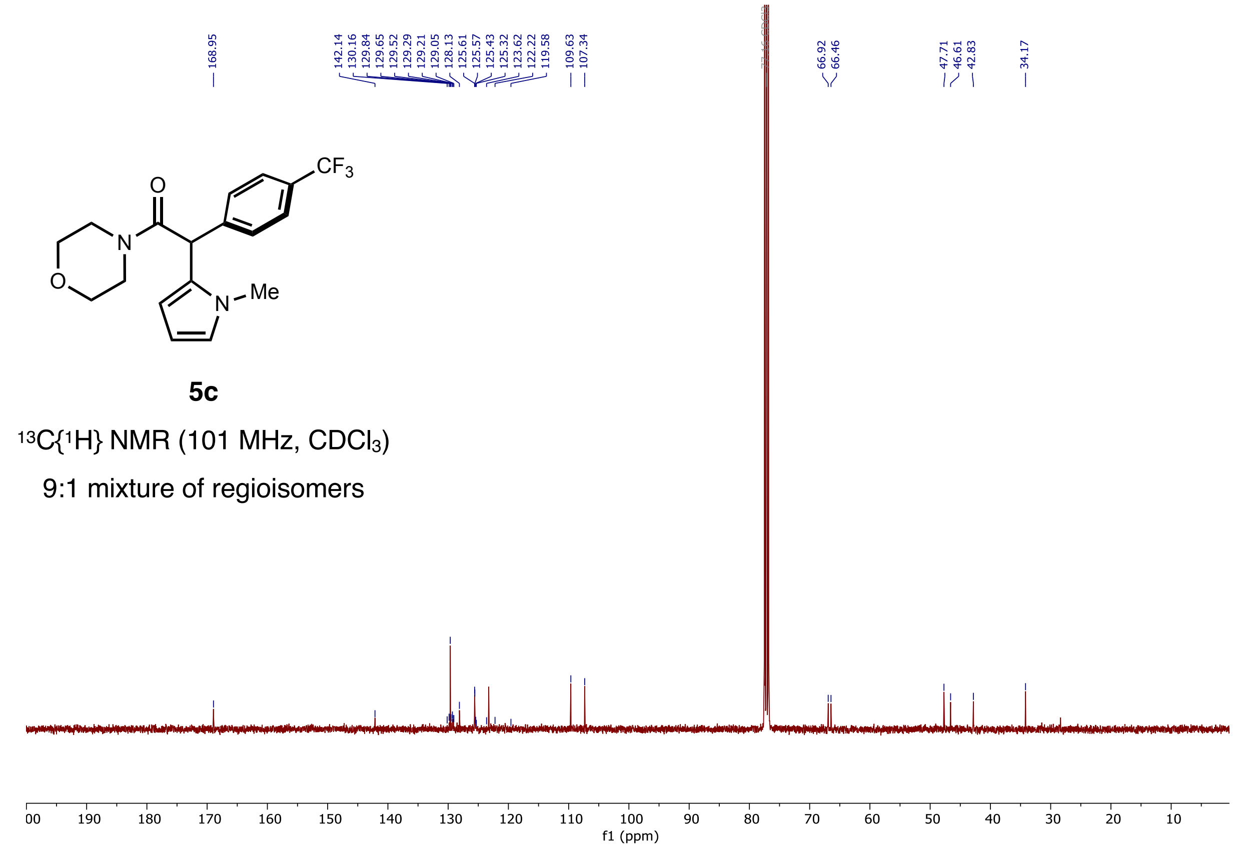

5c

$^{13}C\{^{1}H\}$ NMR (101 MHz, $CDCl_3$)

9:1 mixture of regioisomers

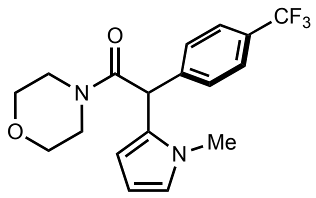

5c

^{19}F NMR (376 MHz, $CDCl_3$)

9:1 mixture of regioisomers

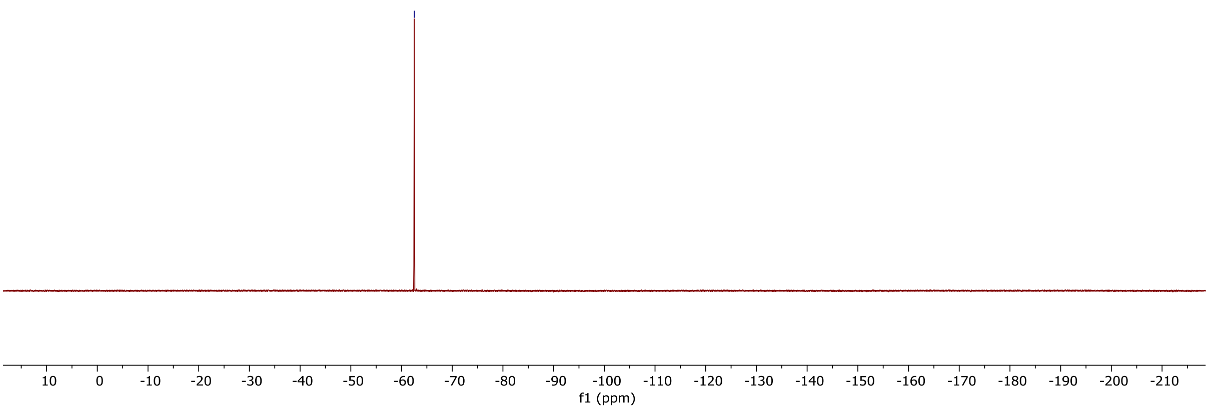

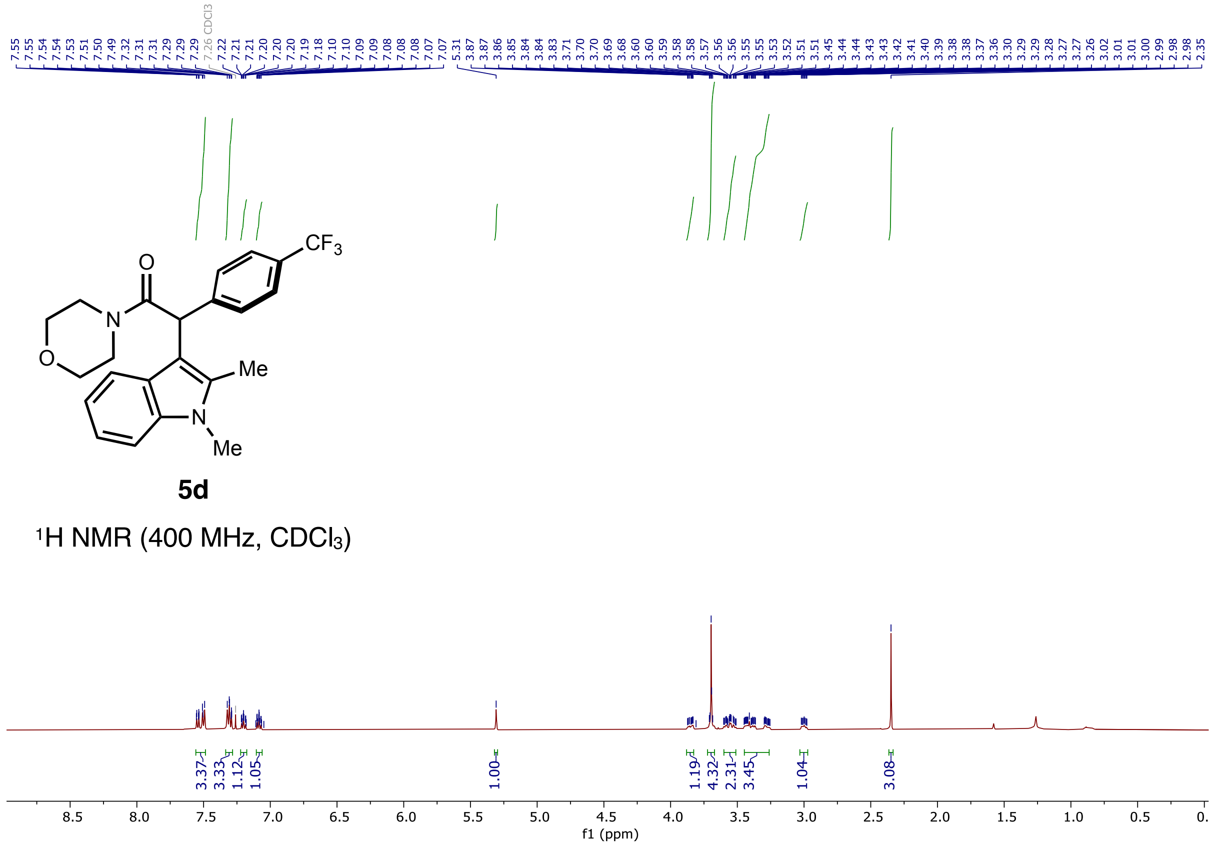
7.55 7.55 7.54 7.54 7.53 7.51 7.50 7.49 7.32 7.31 7.31 7.29 7.29 7.26 CDCl3 7.22 7.21 7.21 7.20 7.20 7.19 7.18 7.10 7.10 7.09 7.09 7.08 7.08 7.07 7.07 5.31 3.87 3.87 3.86 3.85 3.84 3.84 3.83 3.71 3.70 3.69 3.68 3.60 3.60 3.59 3.58 3.58 3.57 3.56 3.56 3.55 3.55 3.53 3.52 3.51 3.51 3.45 3.44 3.44 3.43 3.43 3.42 3.41 3.40 3.39 3.38 3.38 3.37 3.36 3.30 3.29 3.29 3.28 3.27 3.27 3.26 3.02 3.01 3.01 3.00 2.99 2.98 2.98 2.35
CF3
O
N
O
Me
N
Me
5d
1H NMR (400 MHz, CDCl3)
3.37 3.33 1.12 1.05 1.00 1.19 4.32 2.31 3.45 1.04 3.08
8.5 8.0 7.5 7.0 6.5 6.0 5.5 5.0 4.5 4.0 3.5 3.0 2.5 2.0 1.5 1.0 0.5 0.
f1 (ppm)

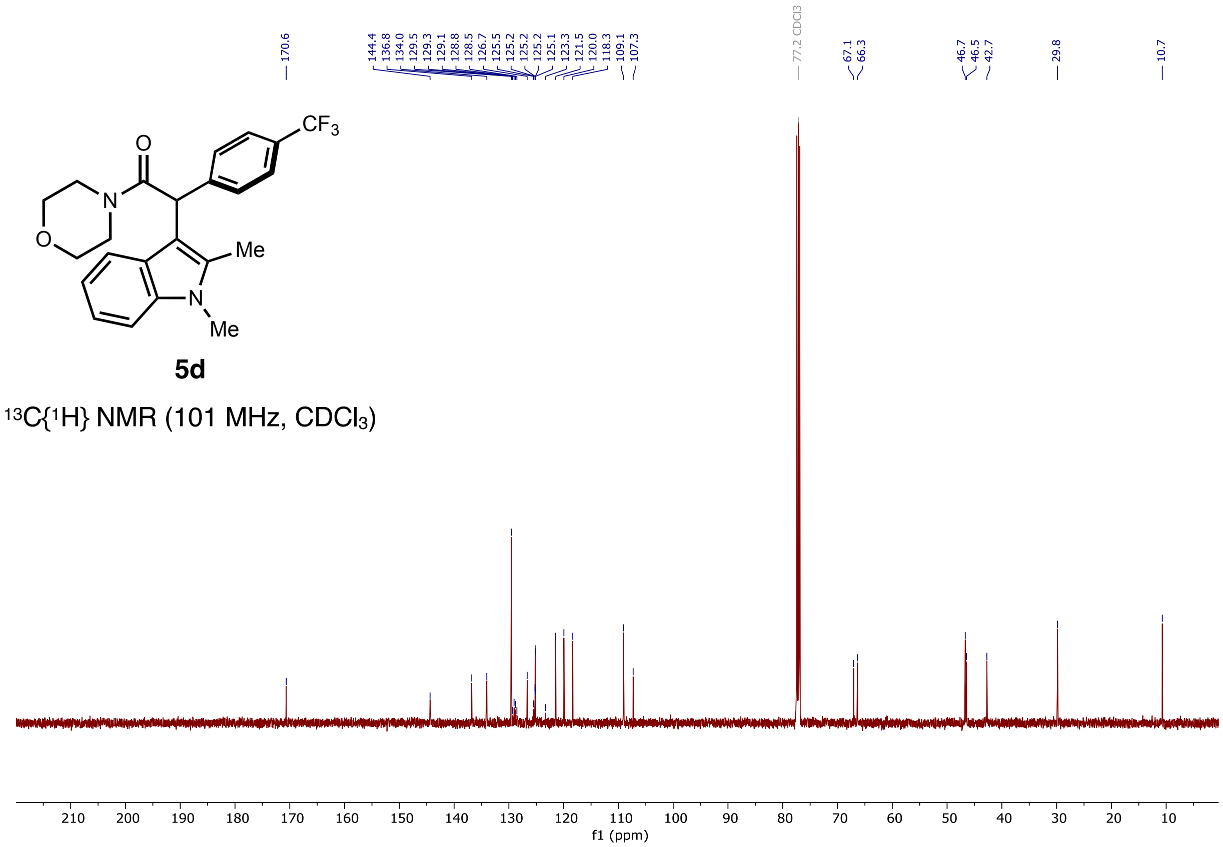
170.6
144.4 136.8 134.0 129.5 129.3 129.1 128.8 128.5 126.7 125.5 125.2 125.2 125.1 123.3 121.5 120.0 118.3 109.1 107.3
77.2 CDCl3
67.1 66.3
46.7 46.5 42.7
29.8
10.7
CF3
O
N
O
Me
N
Me
5d
13C{1H} NMR (101 MHz, CDCl3)
210 200 190 180 170 160 150 140 130 120 110 100 90 80 70 60 50 40 30 20 10
f1 (ppm)

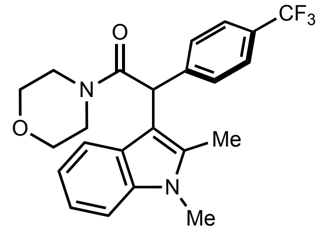

5d

^{19}F NMR (376 MHz, $CDCl_3$)

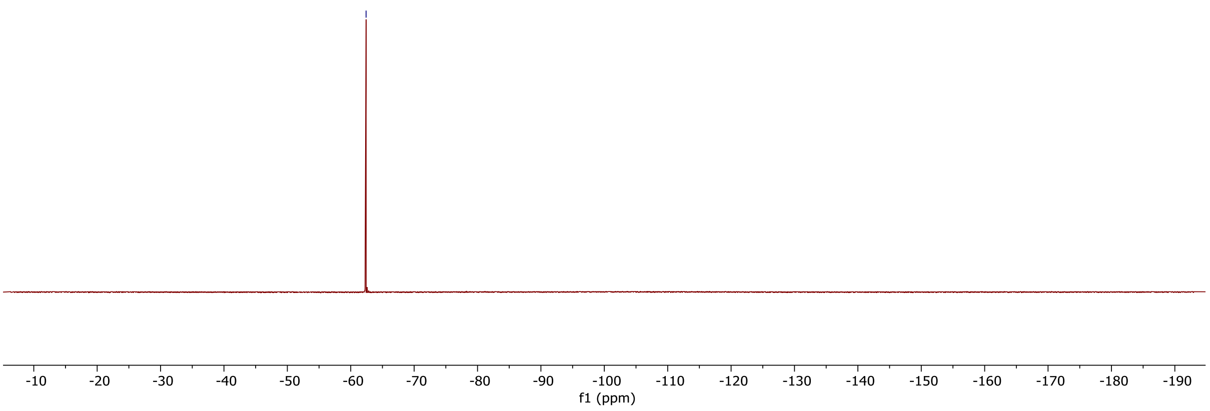

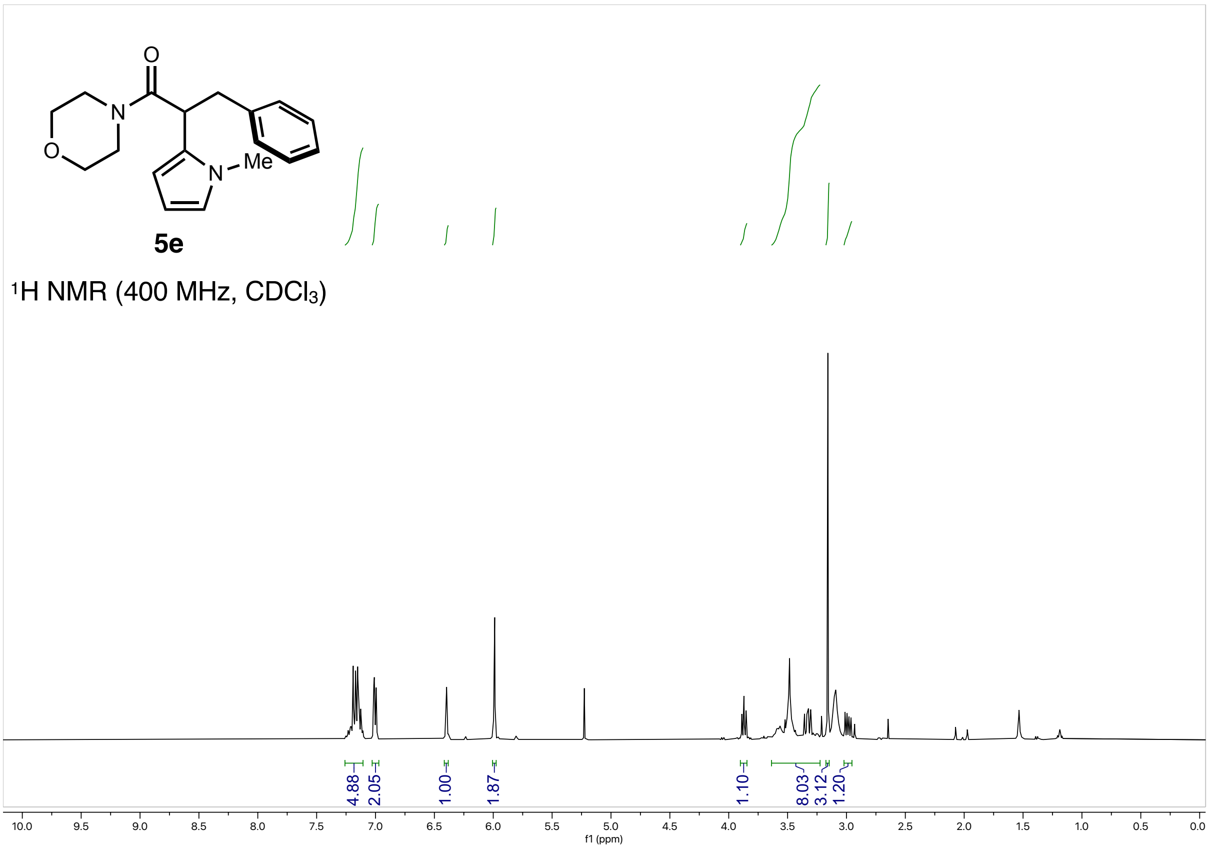
O
N
O
N
Me
5e
¹H NMR (400 MHz, CDCl₃)
4.88
2.05
1.00
1.87
1.10
8.03
3.12
1.20
10.0
9.5
9.0
8.5
8.0
7.5
7.0
6.5
6.0
5.5
5.0
4.5
4.0
3.5
3.0
2.5
2.0
1.5
1.0
0.5
0.0
f1 (ppm)

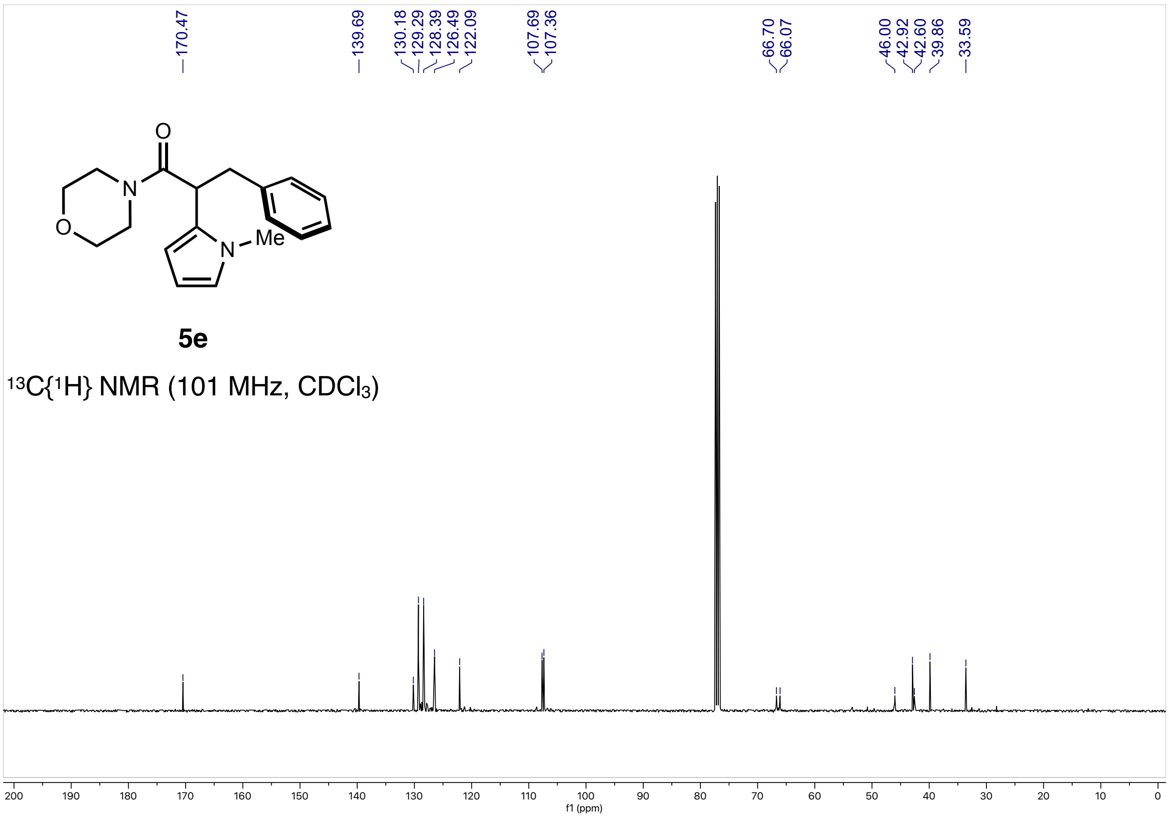
170.47
139.69
130.18
129.29
128.39
126.49
122.09
107.69
107.36
66.70
66.07
46.00
42.92
42.60
39.86
33.59
O
N
O
N
Me
5e
¹³C{¹H} NMR (101 MHz, CDCl₃)
200
190
180
170
160
150
140
130
120
110
100
90
80
70
60
50
40
30
20
10
0
f1 (ppm)

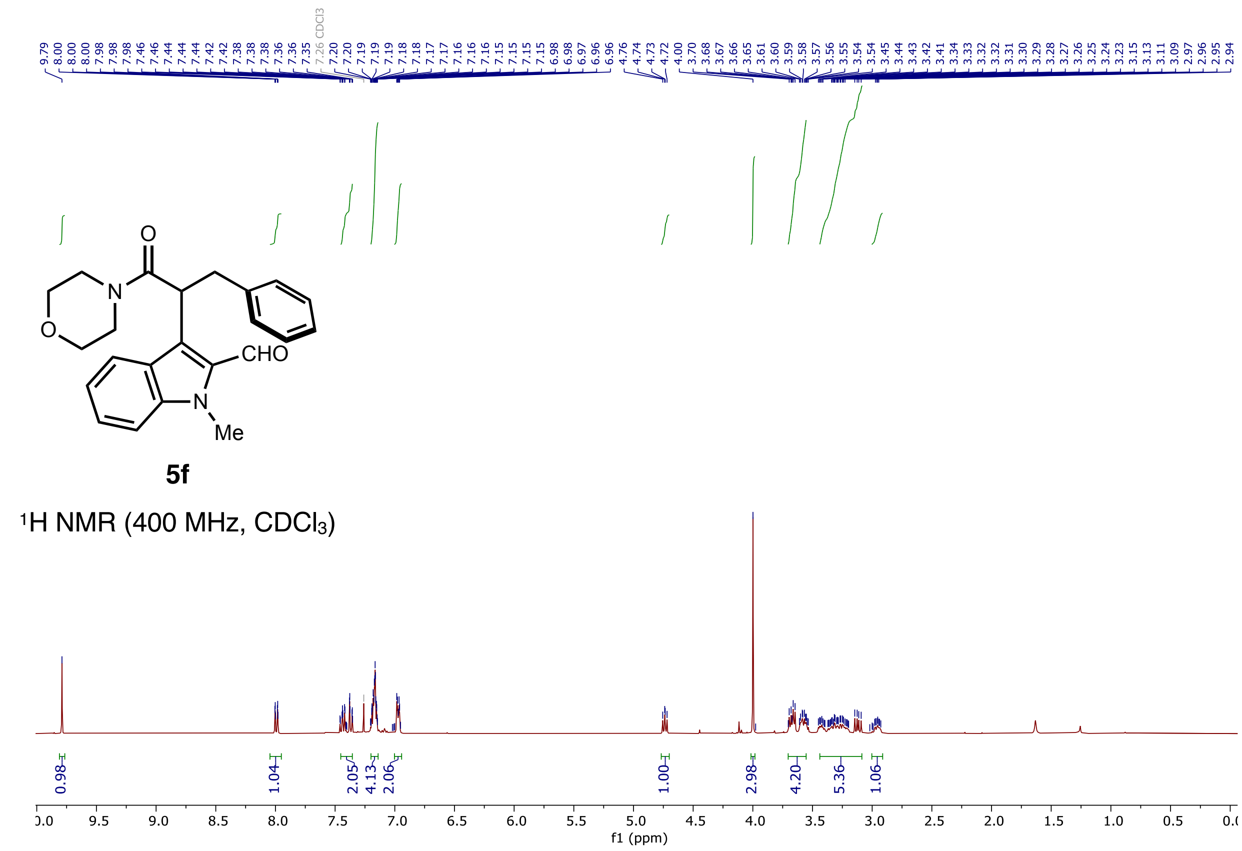
5f
^{1}H NMR (400 MHz, CDCl$_3$)
f1 (ppm)

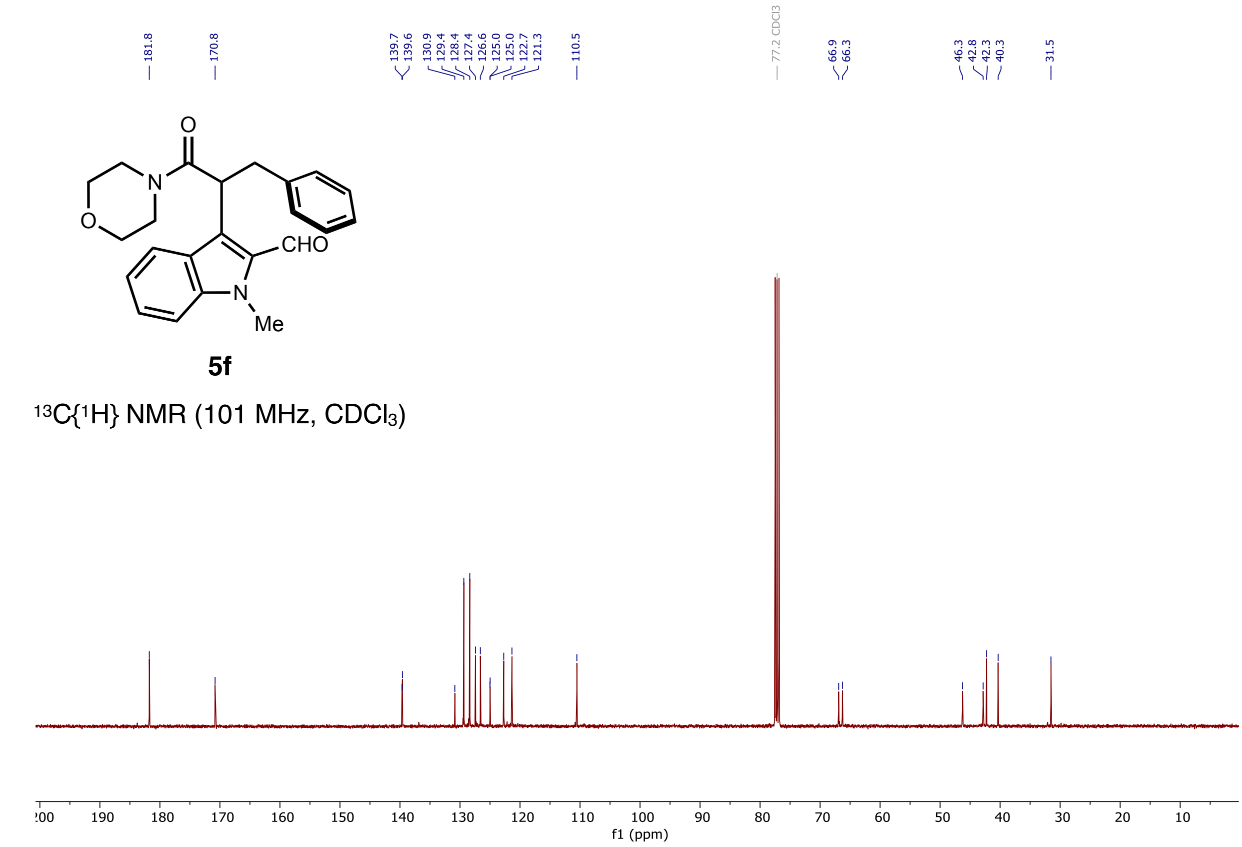
5f
^{13}C{^{1}H} NMR (101 MHz, CDCl$_3$)
f1 (ppm)

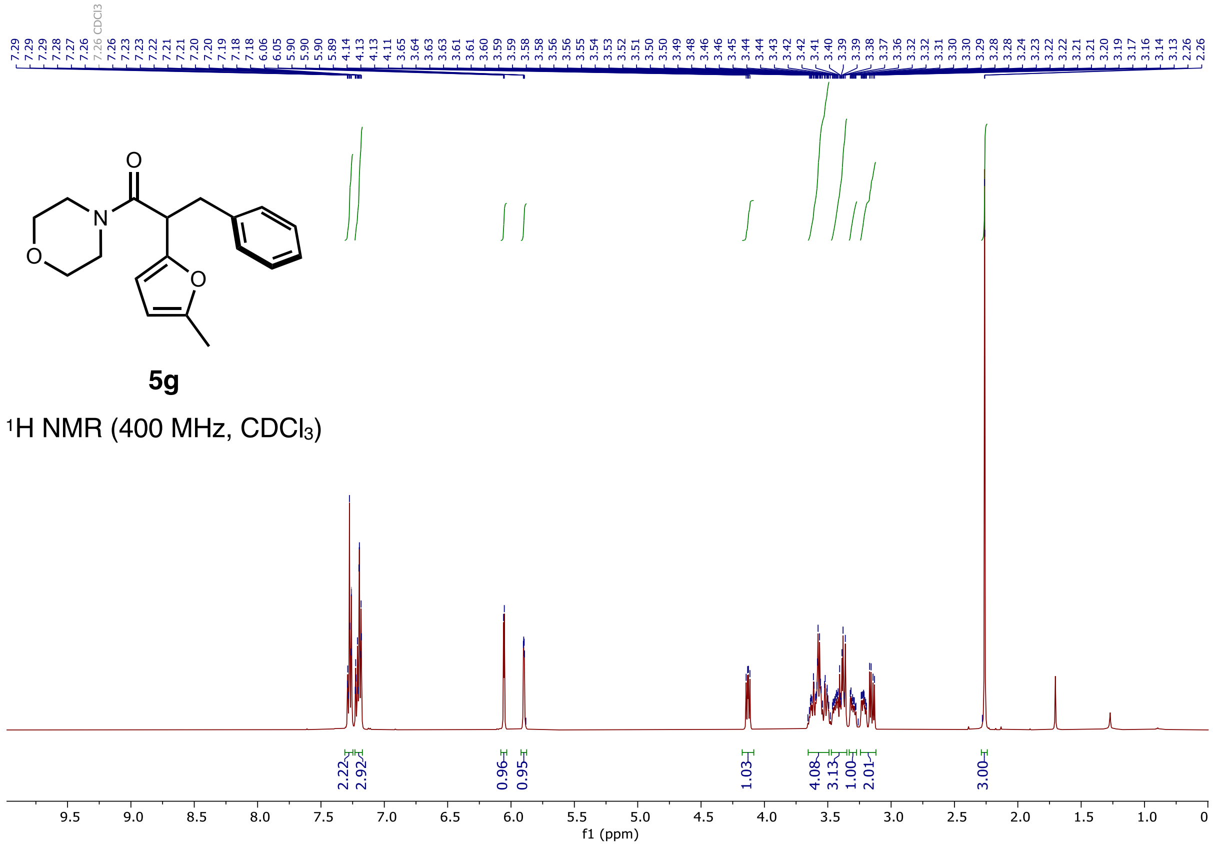

5g

^{1}H NMR (400 MHz, $CDCl_3$)

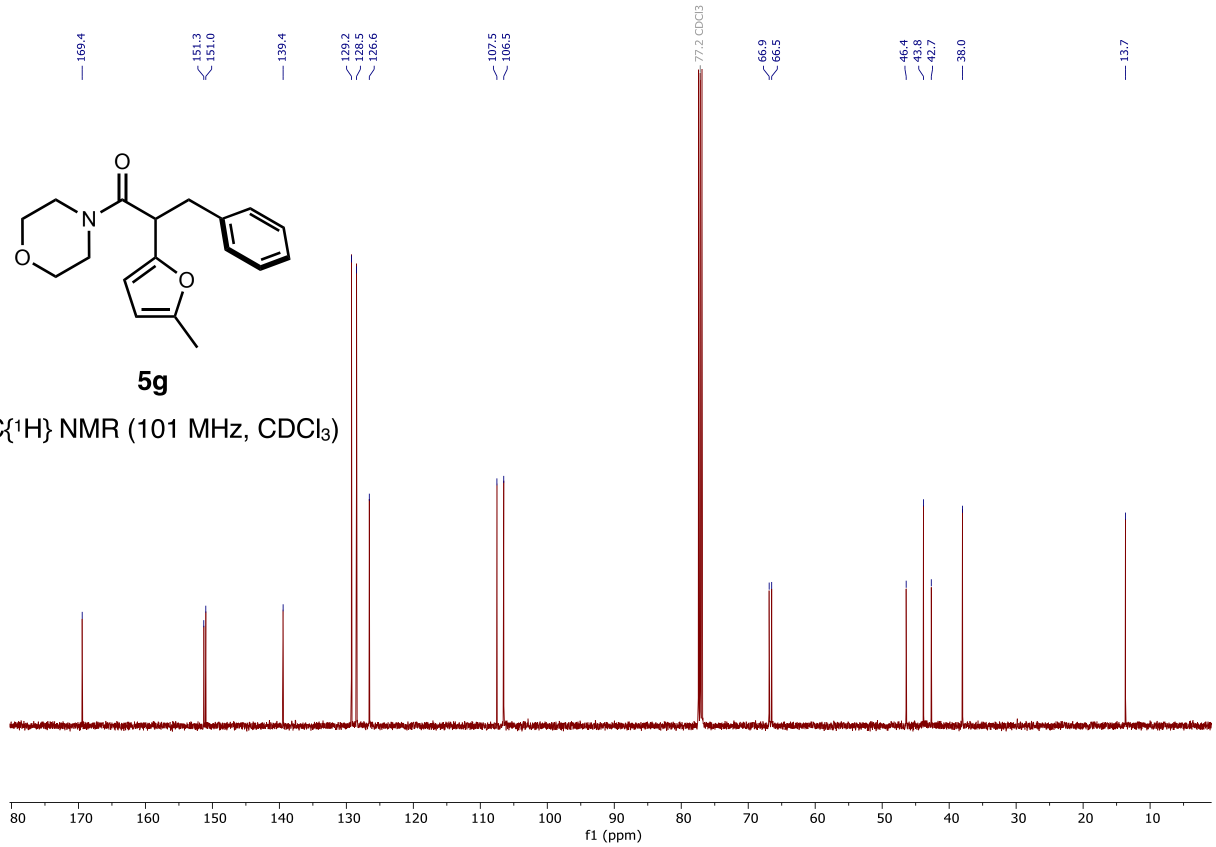

5g

$^{13}C\{^{1}H\}$ NMR (101 MHz, $CDCl_3$)

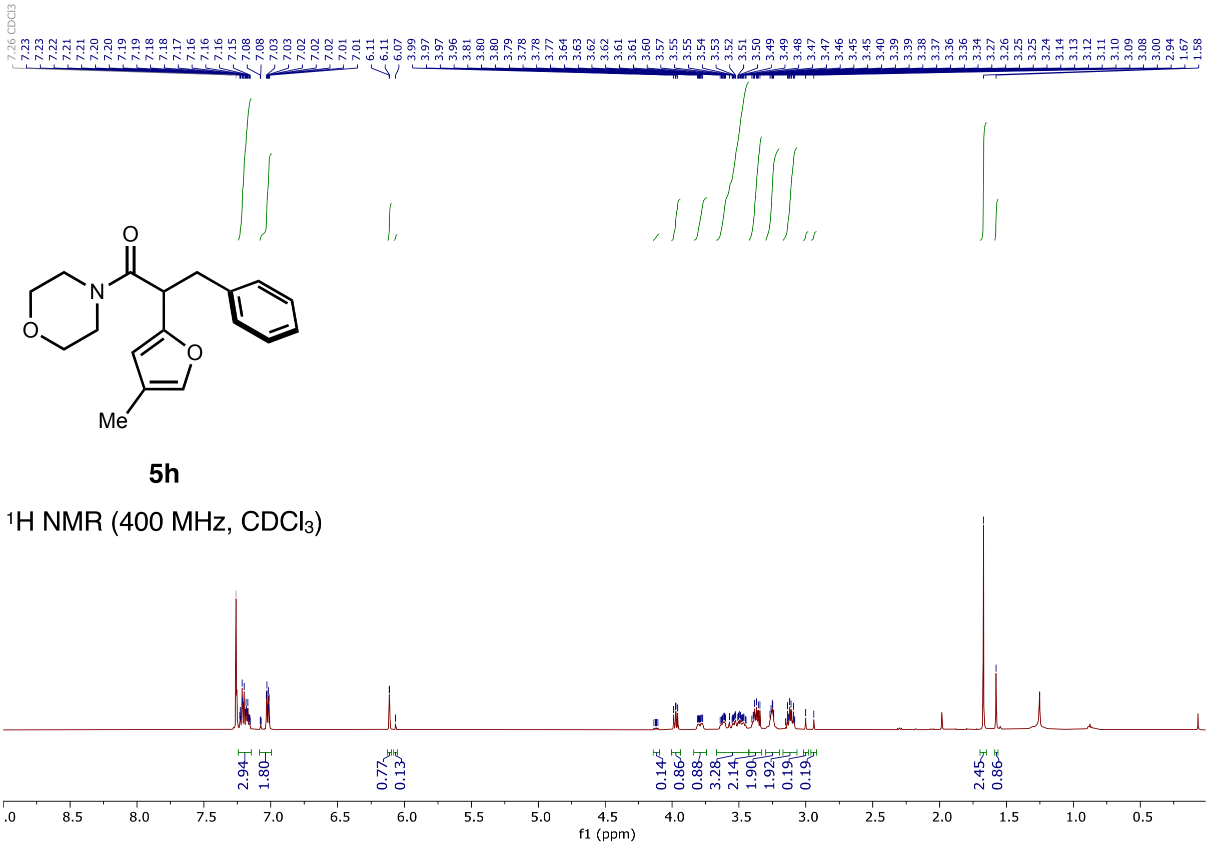

5h

^{1}H NMR (400 MHz, $CDCl_3$)

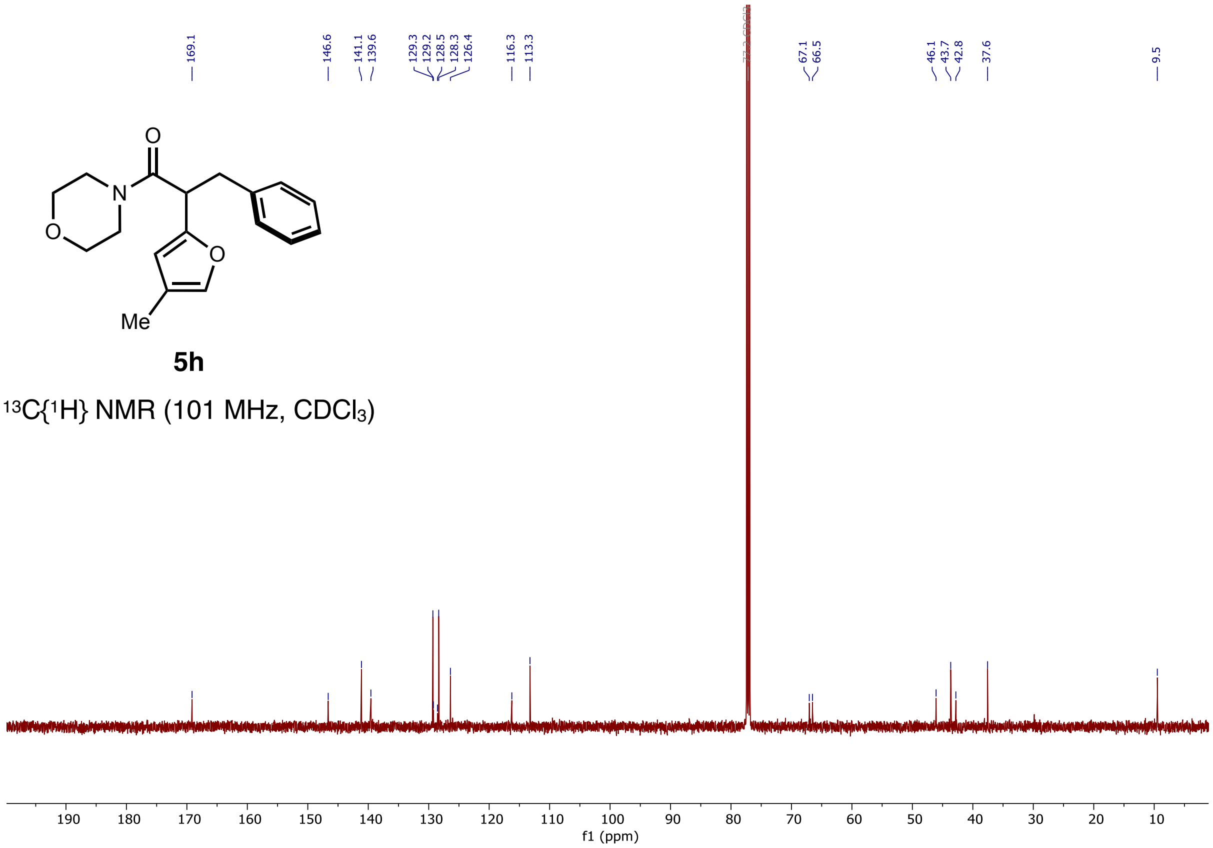

5h

$^{13}C\{^{1}H\}$ NMR (101 MHz, $CDCl_3$)

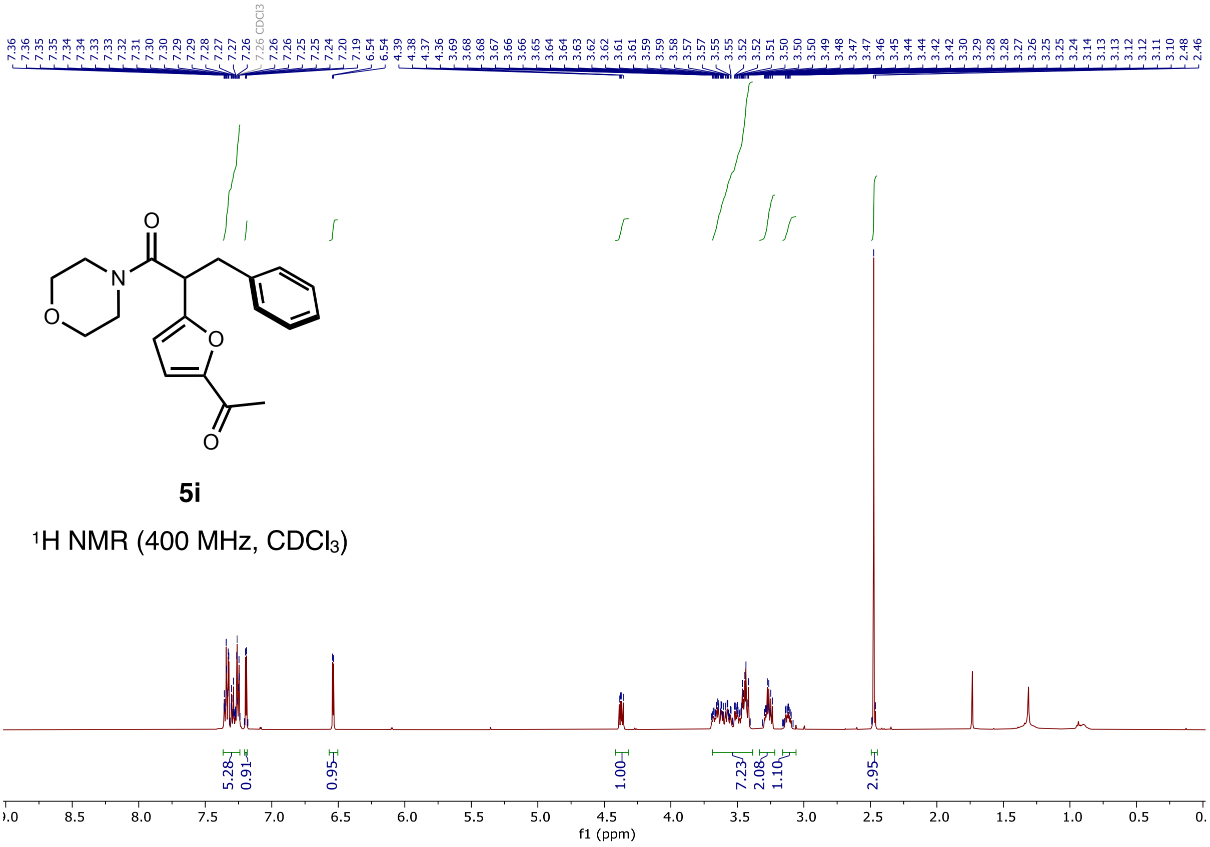

5i

^1H NMR (400 MHz, $CDCl_3$)

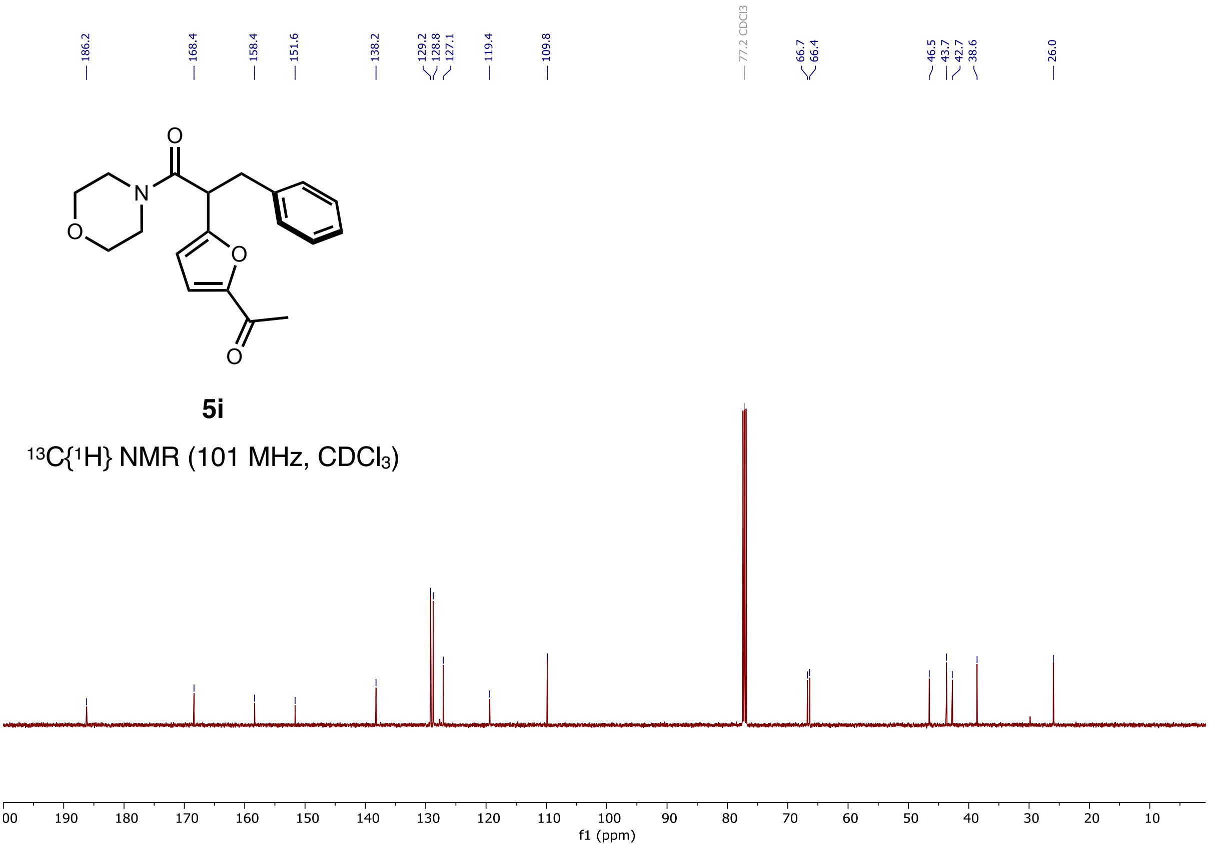

5i

$^{13}C\{^1H\}$ NMR (101 MHz, $CDCl_3$)

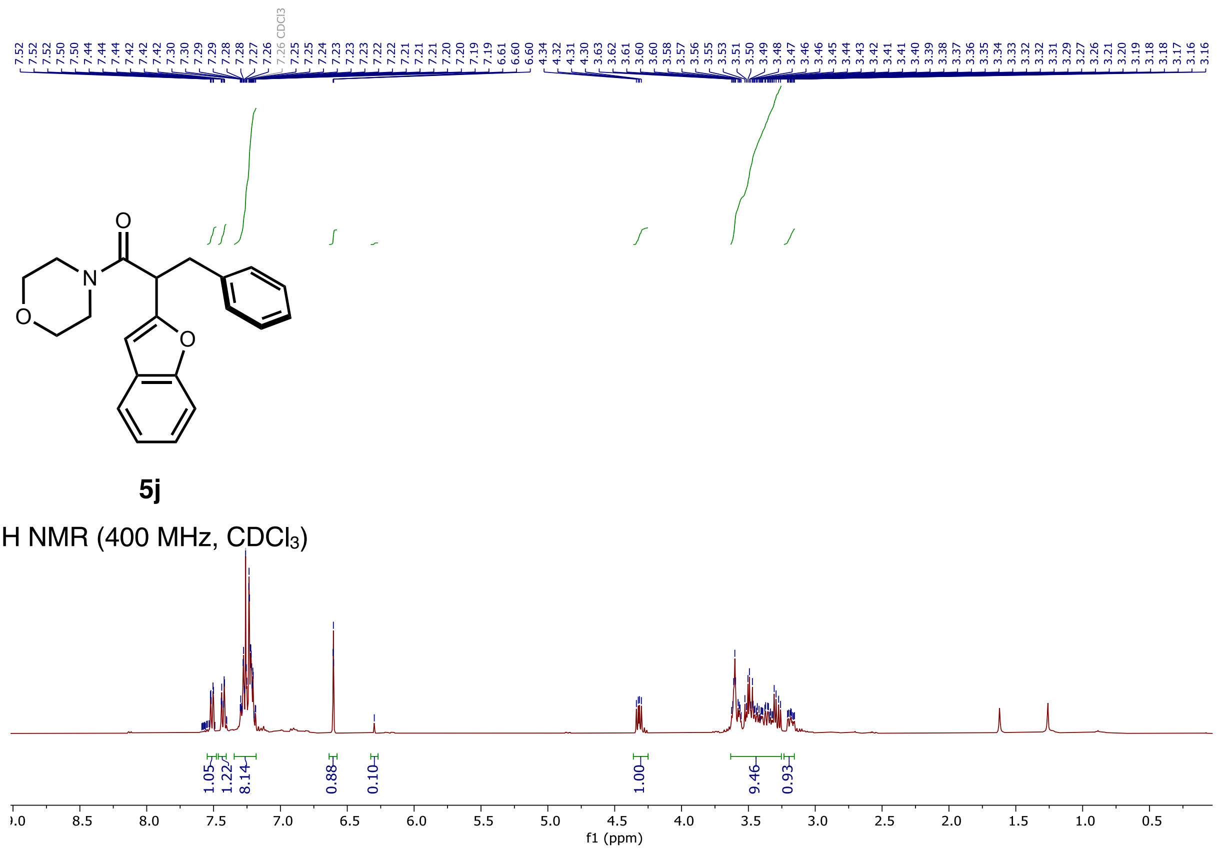
5j
¹H NMR (400 MHz, CDCl₃)
7.26 CDCl3
1.05
1.22
8.14
0.88
0.10
1.00
9.46
0.93
f1 (ppm)

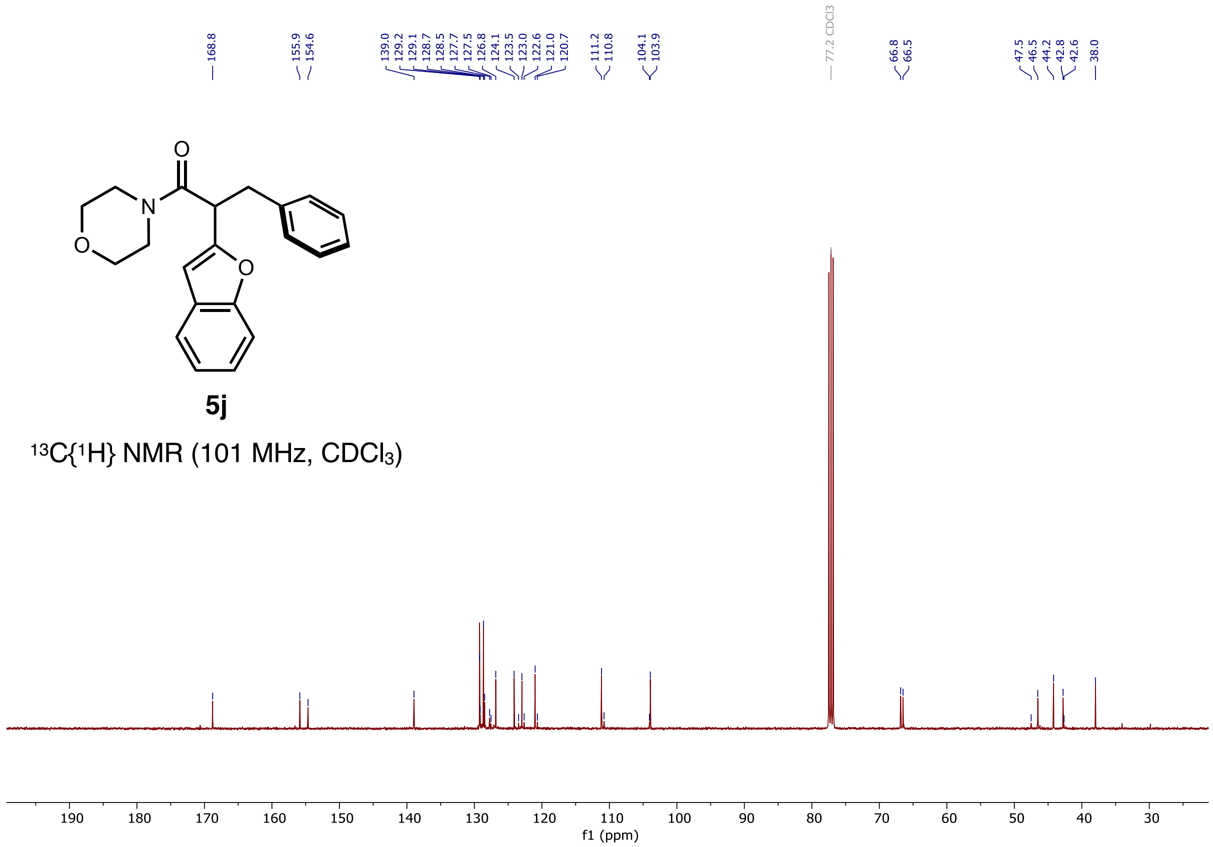
168.8
155.9
154.6
139.0
129.2
129.1
128.7
128.5
127.7
127.5
126.8
124.1
123.5
123.0
122.6
121.0
120.7
111.2
110.8
104.1
103.9
77.2 CDCl3
66.8
66.5
47.5
46.5
44.2
42.8
42.6
38.0
5j
¹³C{¹H} NMR (101 MHz, CDCl₃)
f1 (ppm)

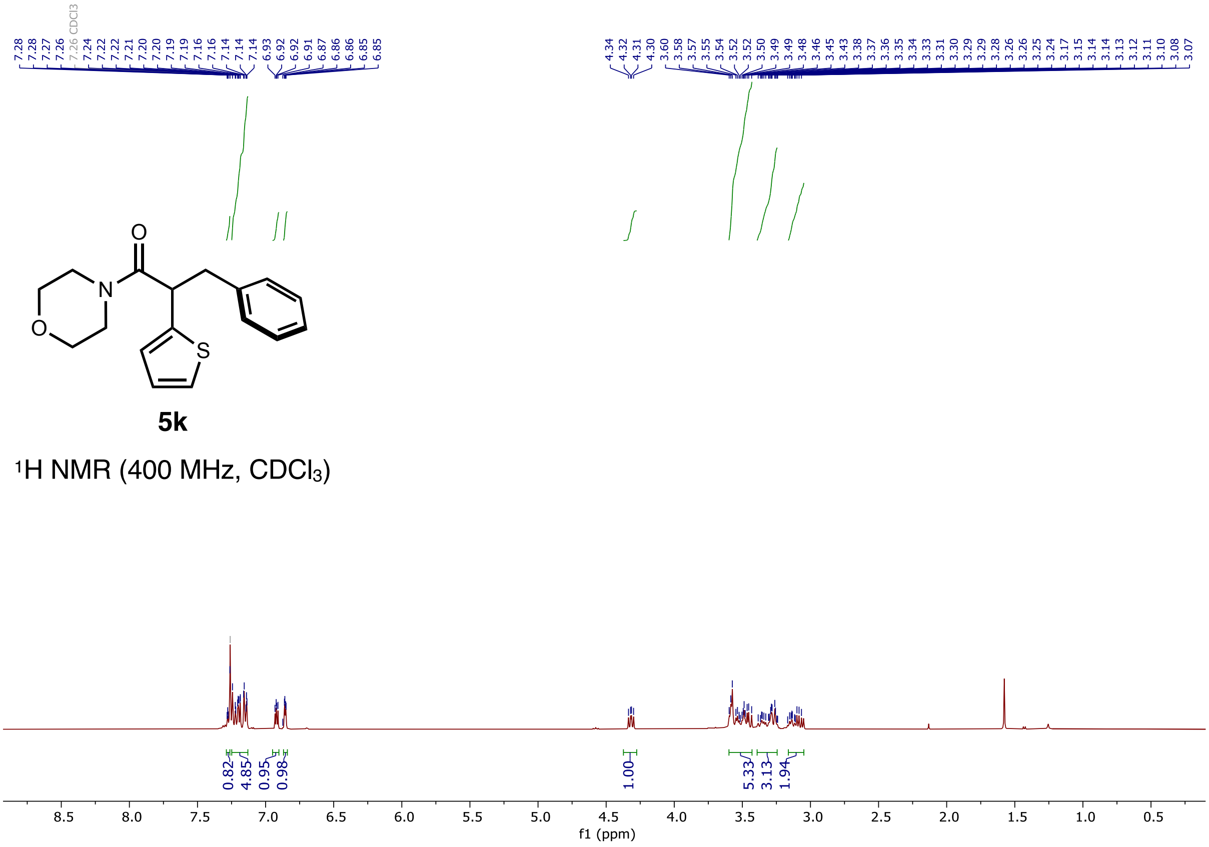
7.28 7.28 7.27 7.26 7.26 CDCl3 7.24 7.22 7.22 7.21 7.20 7.20 7.19 7.19 7.16 7.16 7.14 7.14 7.14 6.93 6.92 6.92 6.91 6.87 6.86 6.86 6.85 6.85
4.34 4.32 4.31 4.30 3.60 3.58 3.57 3.55 3.54 3.52 3.52 3.50 3.49 3.49 3.48 3.46 3.45 3.43 3.38 3.37 3.36 3.35 3.34 3.33 3.31 3.30 3.29 3.29 3.28 3.26 3.25 3.24 3.17 3.15 3.14 3.14 3.13 3.12 3.11 3.10 3.08 3.07
O
N
O
S
5k
1H NMR (400 MHz, CDCl3)
0.82 4.85 0.95 0.98 1.00 5.33 3.13 1.94
8.5 8.0 7.5 7.0 6.5 6.0 5.5 5.0 4.5 4.0 3.5 3.0 2.5 2.0 1.5 1.0 0.5
f1 (ppm)

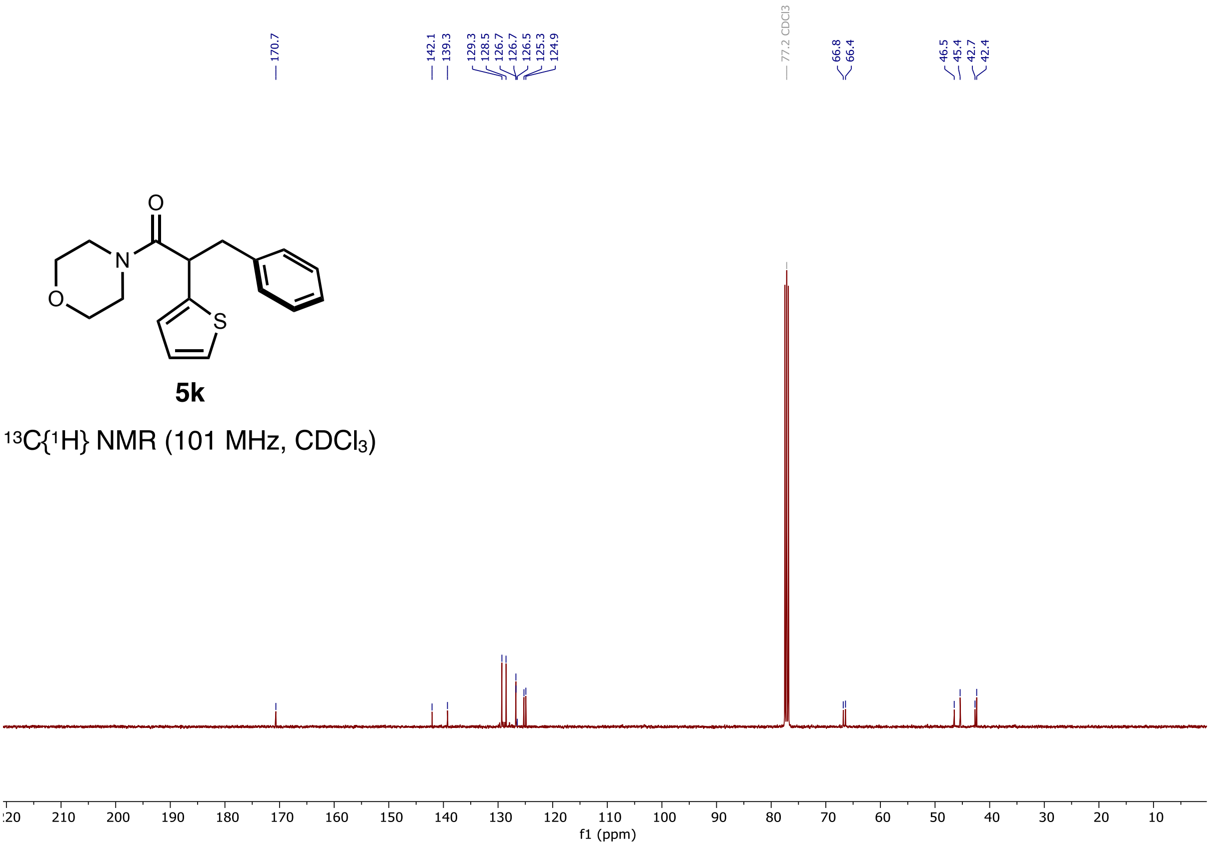
170.7 142.1 139.3 129.3 128.5 126.7 126.7 126.5 125.3 124.9 77.2 CDCl3 66.8 66.4 46.5 45.4 42.7 42.4
O
N
O
S
5k
13C{1H} NMR (101 MHz, CDCl3)
220 210 200 190 180 170 160 150 140 130 120 110 100 90 80 70 60 50 40 30 20 10
f1 (ppm)

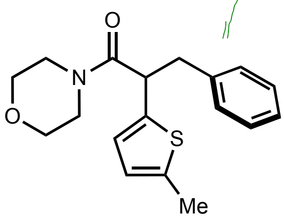

5l

^{1}H NMR (400 MHz, CDCl$_3$)

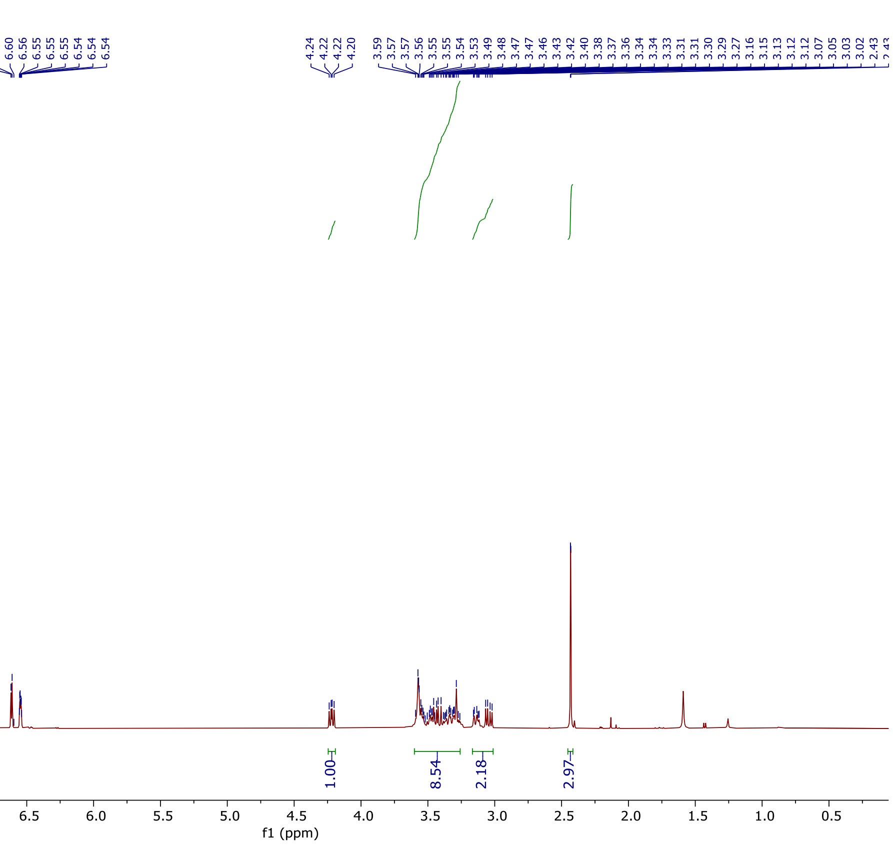

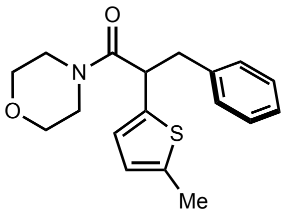

5l

^{13}C{^{1}H} NMR (101 MHz, CDCl$_3$)

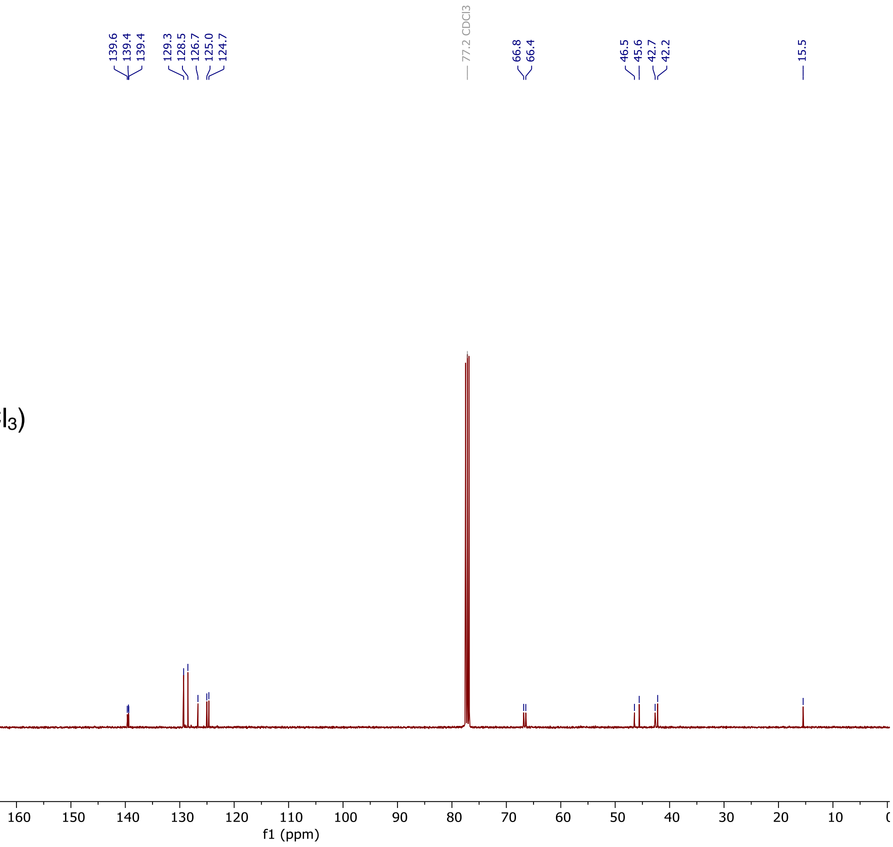

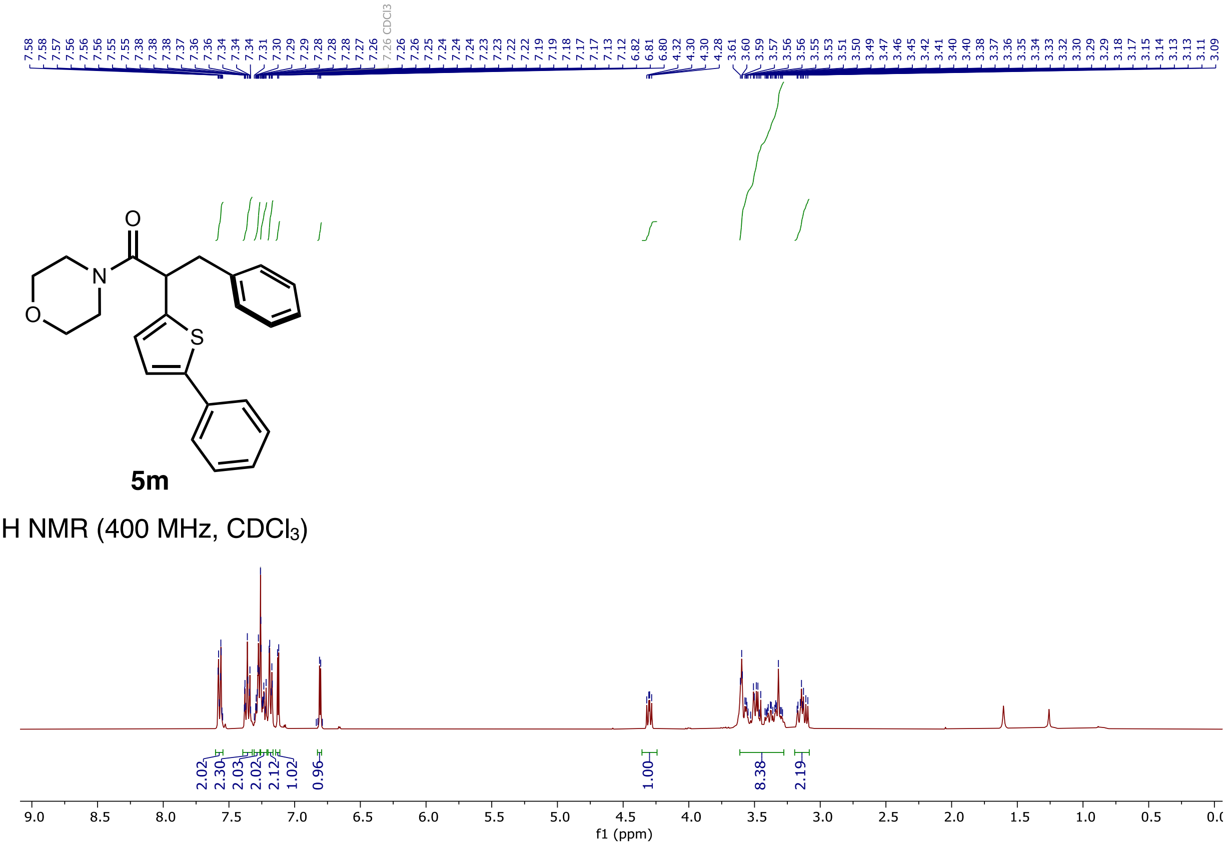

5m

^{1}H NMR (400 MHz, $CDCl_3$)

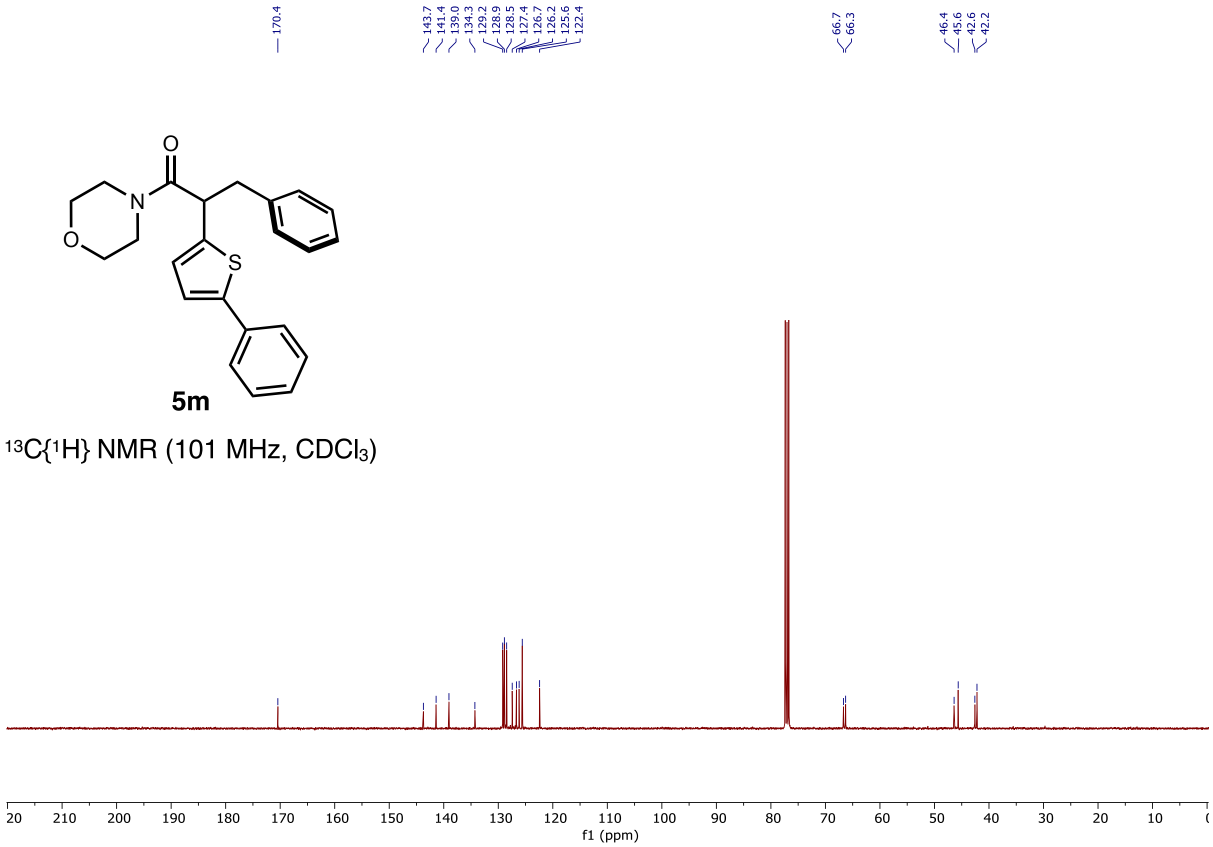

5m

$^{13}C\{^{1}H\}$ NMR (101 MHz, $CDCl_3$)

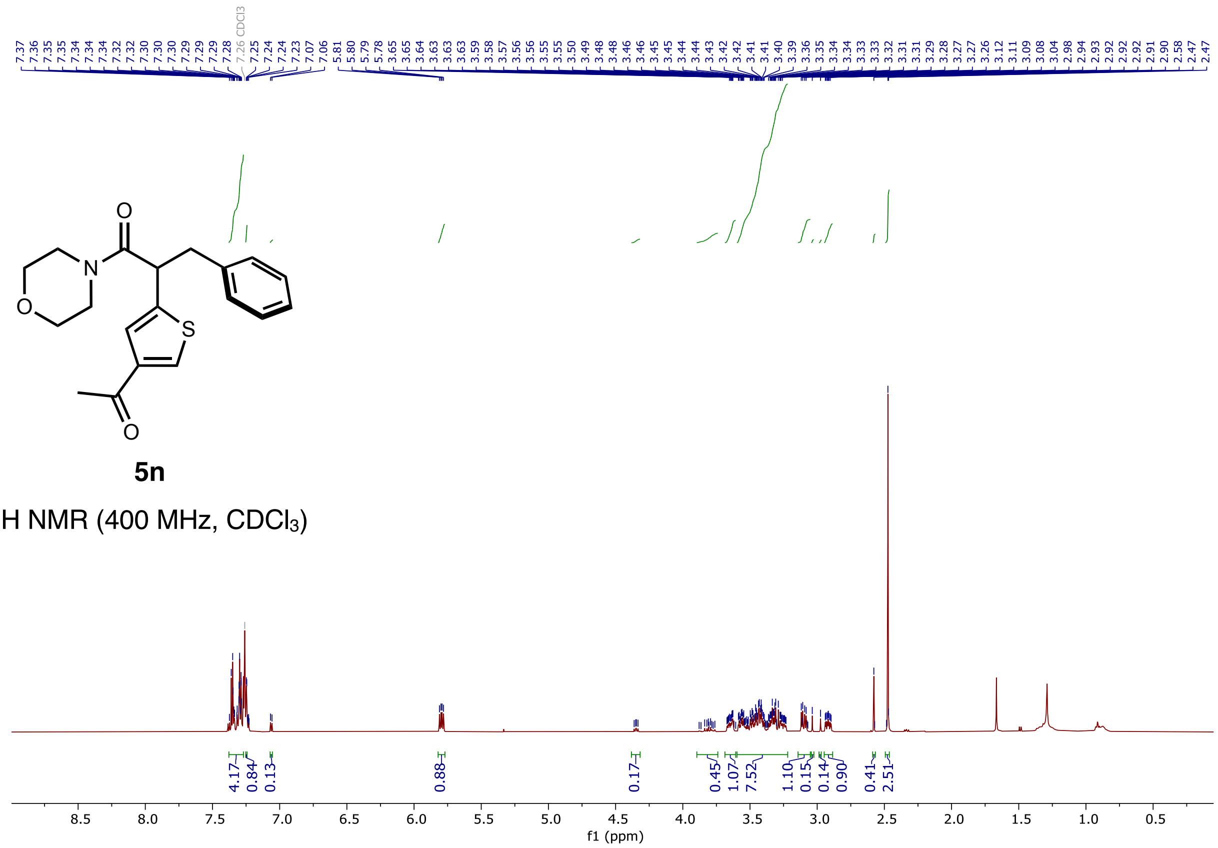
7.37 7.36 7.35 7.35 7.34 7.34 7.34 7.32 7.32 7.30 7.30 7.30 7.29 7.29 7.29 7.28 7.26 CDCl3 7.25 7.24 7.24 7.23 7.07 7.06 5.81 5.80 5.79 5.78 3.65 3.65 3.64 3.63 3.63 3.59 3.58 3.57 3.56 3.56 3.55 3.55 3.50 3.49 3.48 3.46 3.46 3.45 3.45 3.44 3.44 3.43 3.43 3.42 3.42 3.41 3.41 3.40 3.39 3.36 3.35 3.34 3.34 3.33 3.33 3.33 3.32 3.31 3.31 3.29 3.28 3.27 3.27 3.26 3.12 3.11 3.09 3.08 3.04 2.98 2.94 2.93 2.93 2.92 2.92 2.92 2.91 2.90 2.58 2.47 2.47
5n
1H NMR (400 MHz, CDCl3)
4.17 0.84 0.13 0.88 0.17 0.45 1.07 7.52 1.10 0.15 0.14 0.90 0.41 2.51
8.5 8.0 7.5 7.0 6.5 6.0 5.5 5.0 4.5 4.0 3.5 3.0 2.5 2.0 1.5 1.0 0.5
f1 (ppm)

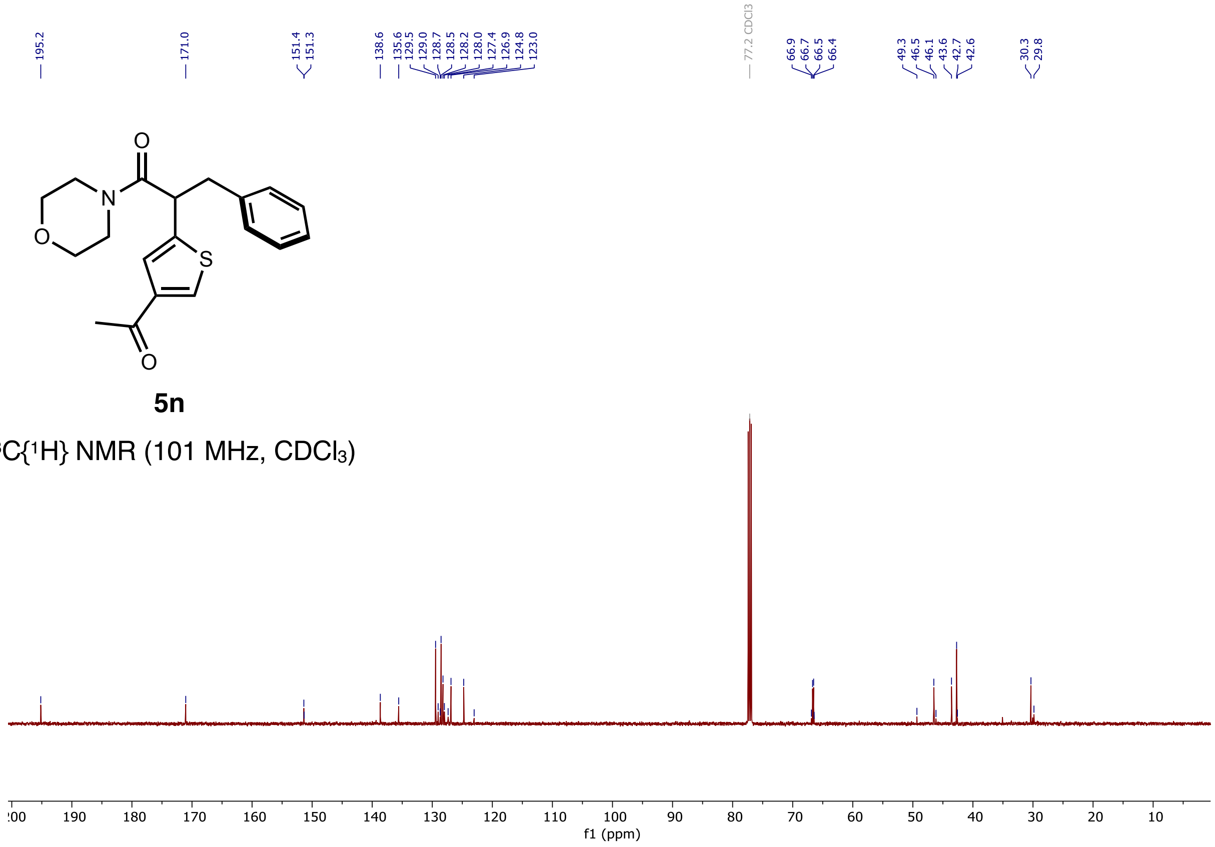
195.2 171.0 151.4 151.3 138.6 135.6 129.5 129.0 128.7 128.5 128.2 128.0 127.4 126.9 124.8 123.0 77.2 CDCl3 66.9 66.7 66.5 66.4 49.3 46.5 46.1 43.6 42.7 42.6 30.3 29.8
5n
13C{1H} NMR (101 MHz, CDCl3)
200 190 180 170 160 150 140 130 120 110 100 90 80 70 60 50 40 30 20 10
f1 (ppm)

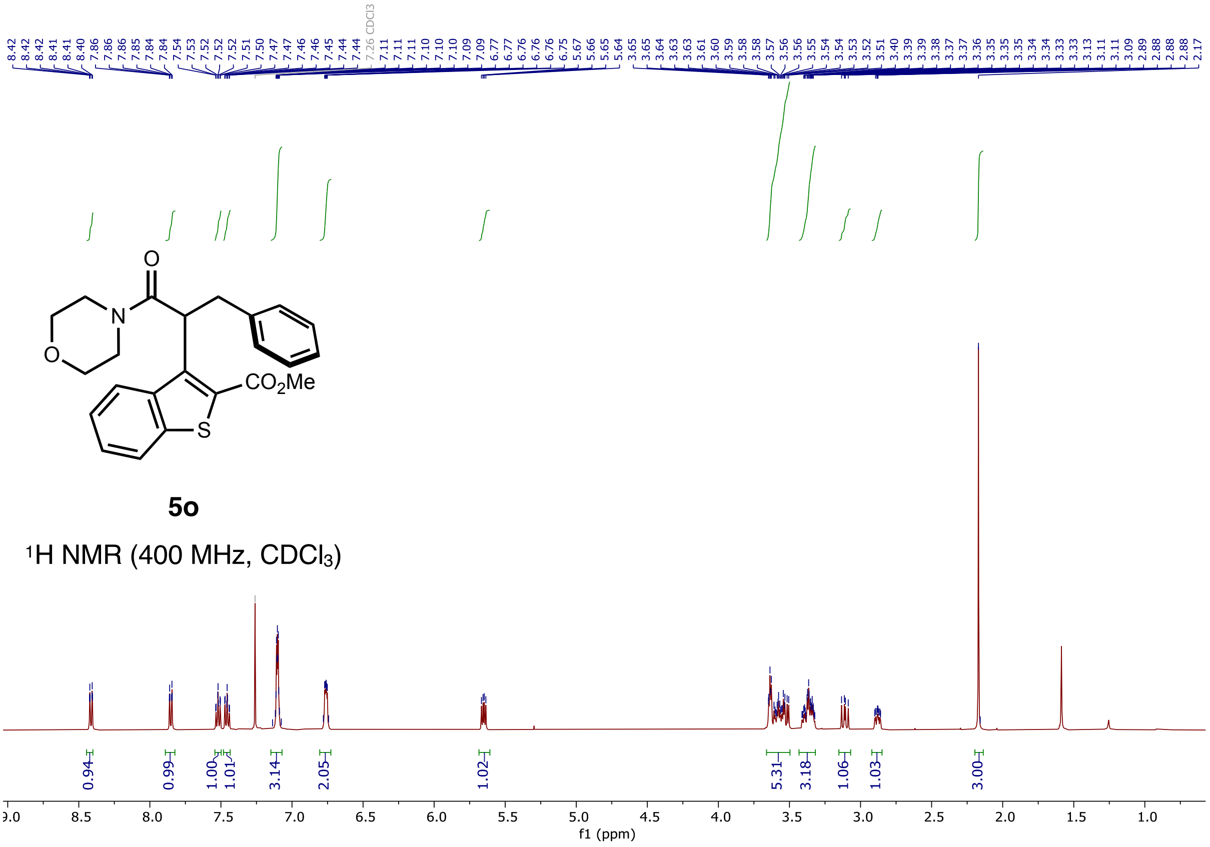

5o

^{1}H NMR (400 MHz, $CDCl_3$)

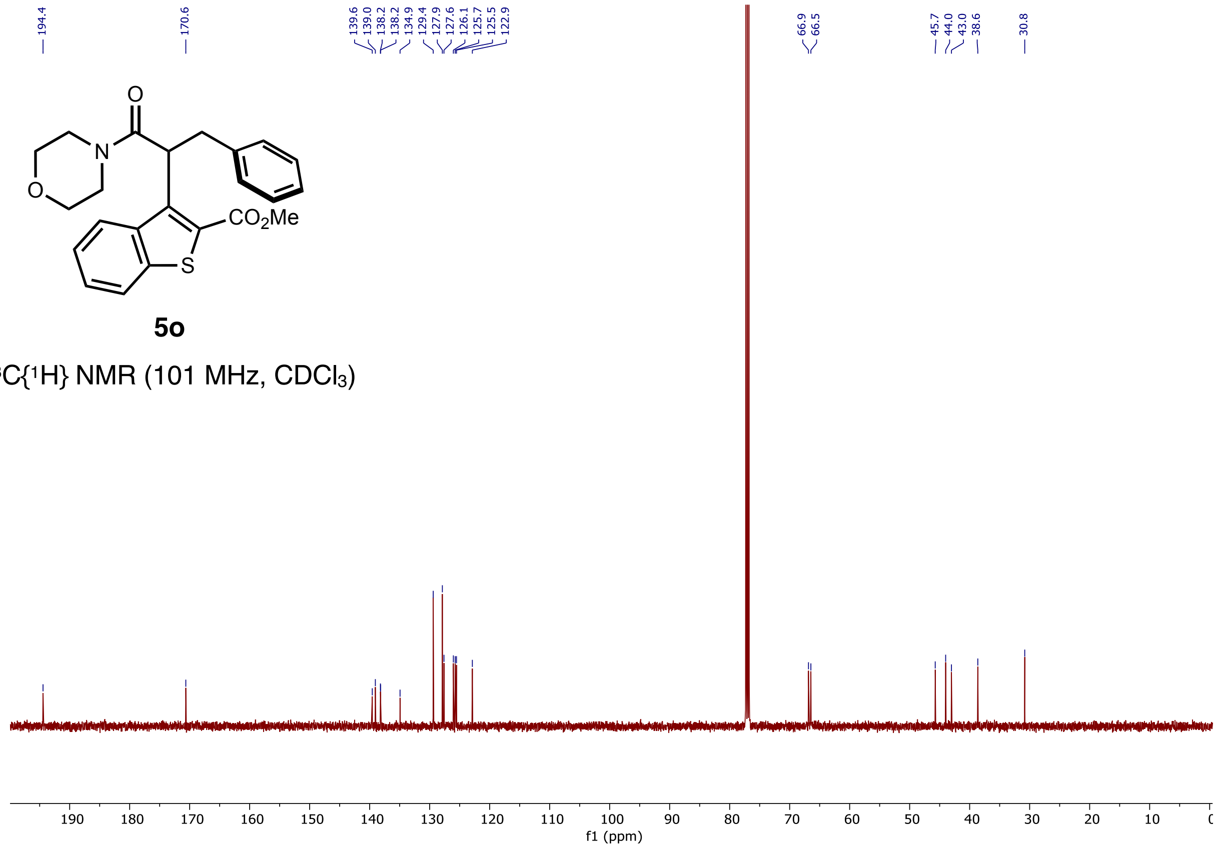

5o

$^{13}C\{^{1}H\}$ NMR (101 MHz, $CDCl_3$)

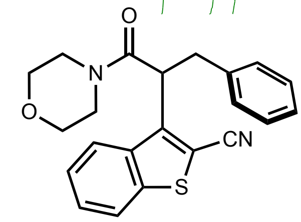

5p

^{1}H NMR (400 MHz, $CDCl_3$)

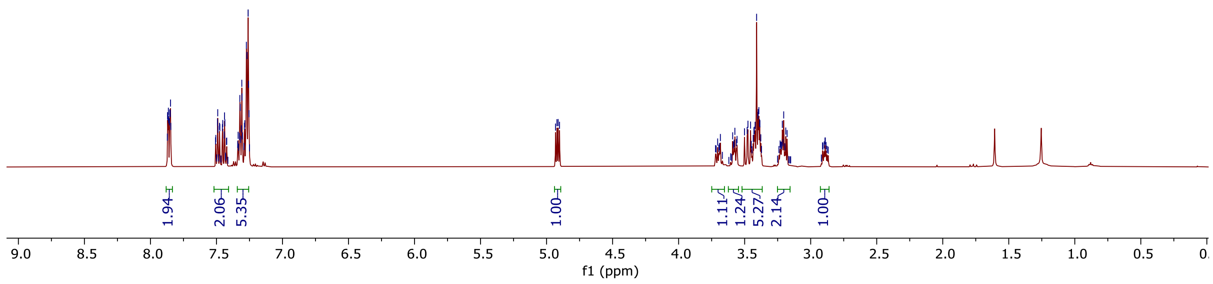

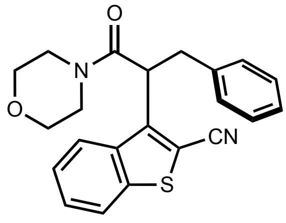

5p

$^{13}C\{^{1}H\}$ NMR (101 MHz, $CDCl_3$)

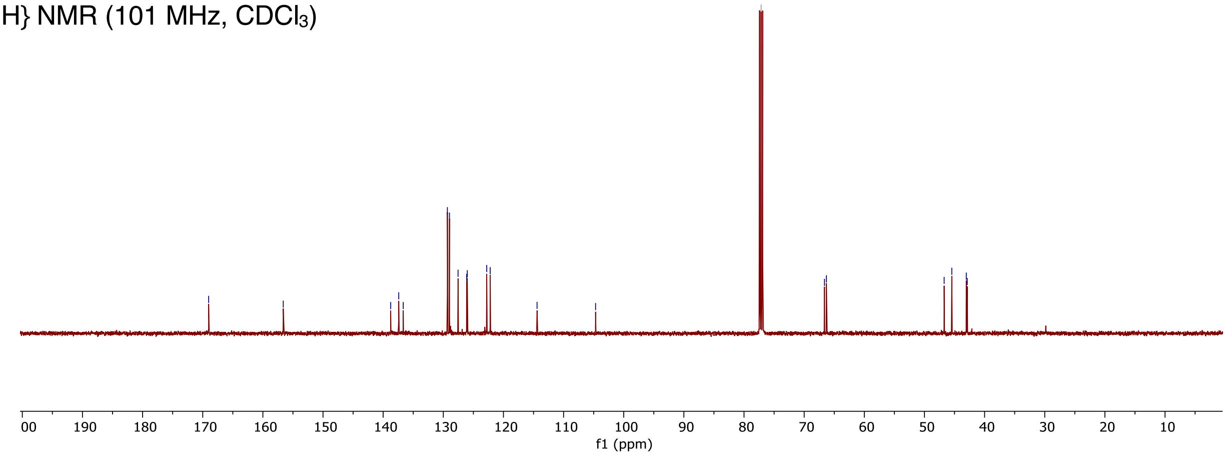

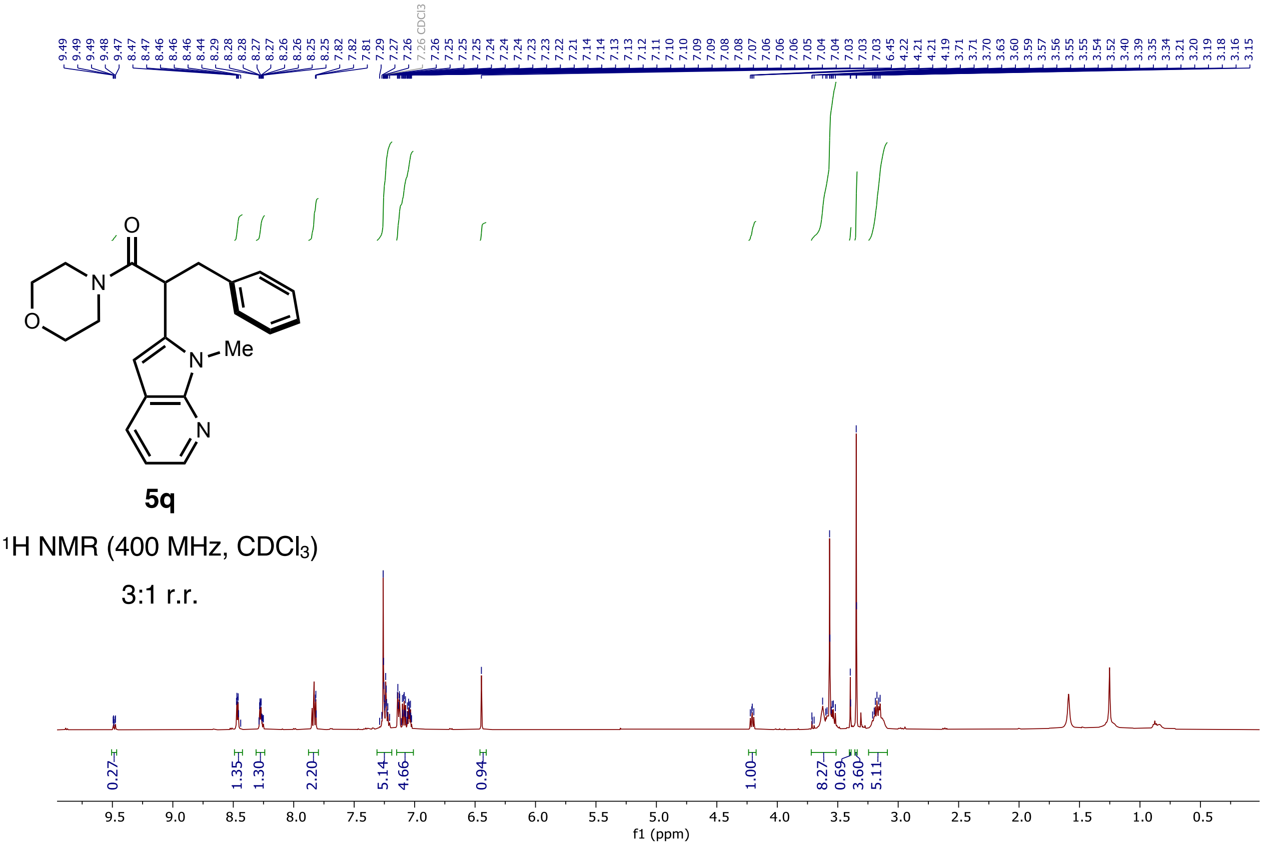

5q

^{1}H NMR (400 MHz, $CDCl_3$)

3:1 r.r.

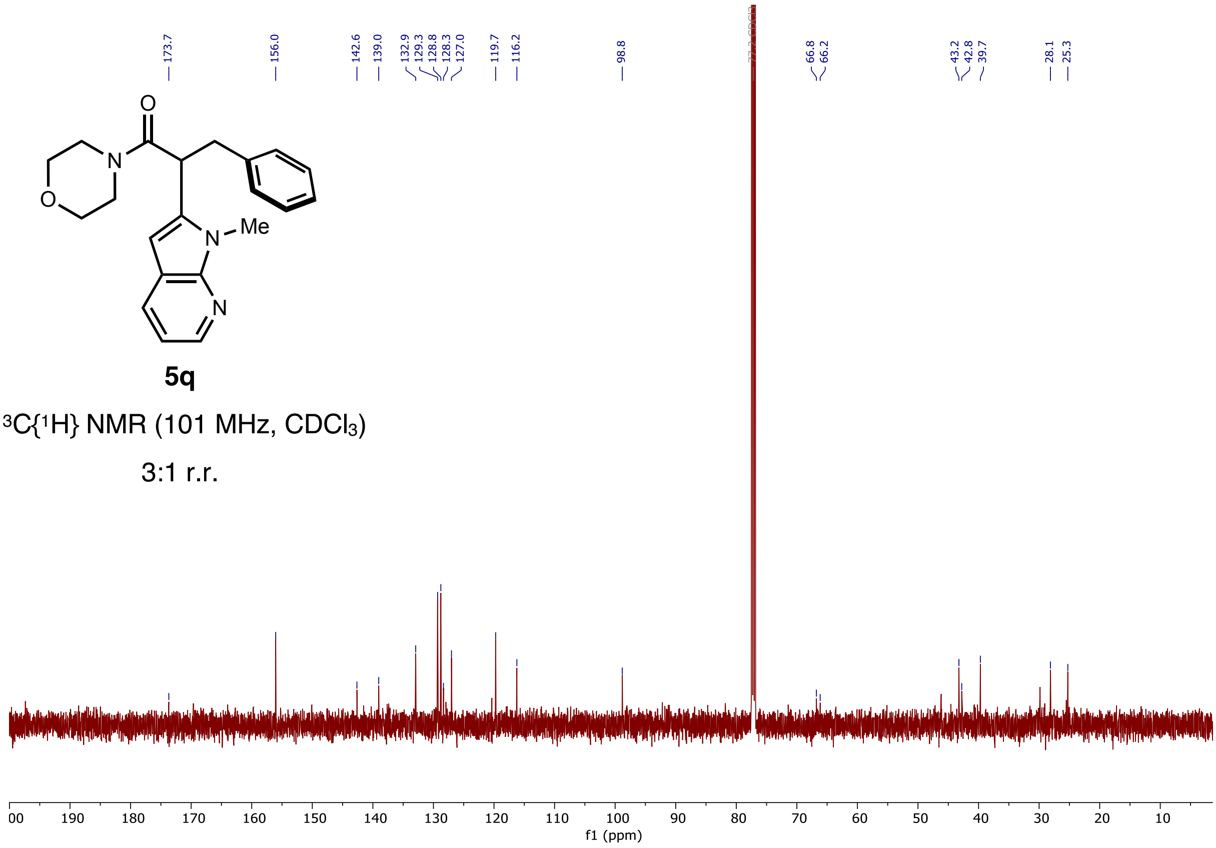

5q

$^{13}C\{^1H\}$ NMR (101 MHz, $CDCl_3$)

3:1 r.r.

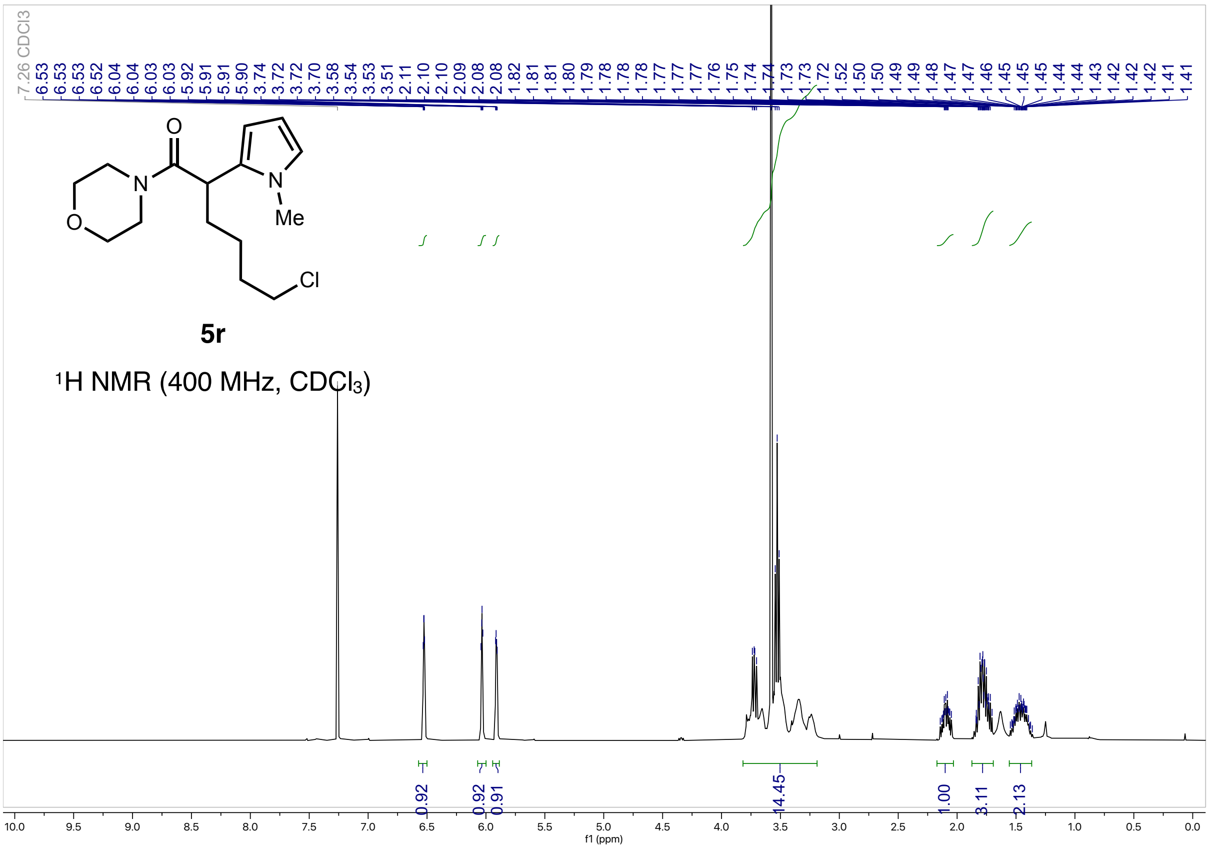
7.26 CDCl3
6.53 6.53 6.53 6.52 6.04 6.04 6.03 6.03 5.92 5.91 5.91 5.90 3.74 3.72 3.72 3.70 3.58 3.54 3.53 3.51 2.11 2.10 2.10 2.09 2.08 2.08 1.82 1.81 1.81 1.80 1.79 1.78 1.78 1.78 1.77 1.77 1.77 1.76 1.75 1.74 1.74 1.73 1.73 1.72 1.52 1.50 1.50 1.49 1.49 1.48 1.47 1.47 1.46 1.45 1.45 1.44 1.44 1.43 1.42 1.42 1.41 1.41
O
N
O
N
Me
Cl
5r
¹H NMR (400 MHz, CDCl₃)
0.92
0.92
0.91
14.45
1.00
3.11
2.13
f1 (ppm)

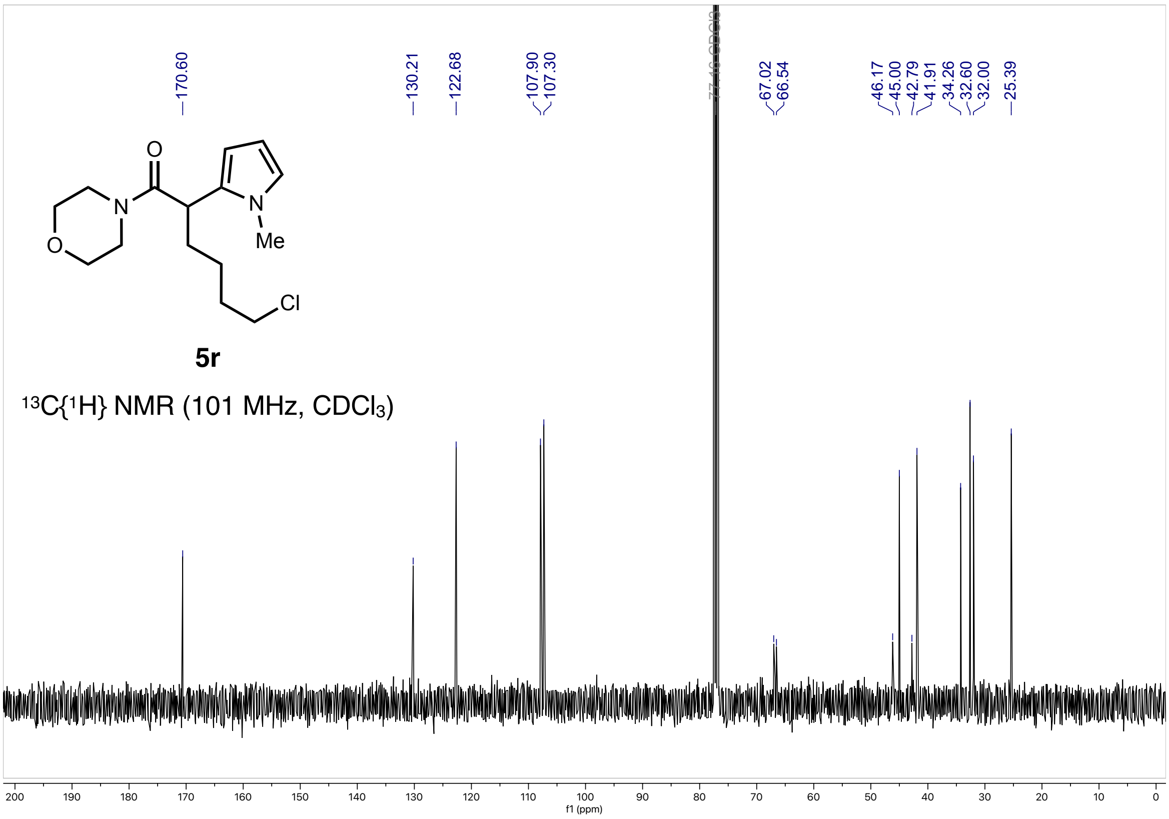
170.60
130.21
122.68
107.90
107.30
77.16 CDCl3
67.02
66.54
46.17
45.00
42.79
41.91
34.26
32.60
32.00
25.39
O
N
O
N
Me
Cl
5r
¹³C{¹H} NMR (101 MHz, CDCl₃)
f1 (ppm)
